# Transposable elements contribute to the genome plasticity of *Ralstonia solanacearum* species complex

**DOI:** 10.1099/mgen.0.000374

**Published:** 2020-05-07

**Authors:** Osiel Silva Gonçalves, Kiara França Campos, Jéssica Catarine Silva de Assis, Alexia Suellen Fernandes, Thamires Santos Souza, Luiz Guilherme do Carmo Rodrigues, Marisa Vieira de Queiroz, Mateus Ferreira Santana

**Affiliations:** ^1^​ Departamento de Microbiologia, Instituto de Biotecnologia Aplicada à Agropecuária (BIOAGRO), Universidade Federal de Viçosa, Viçosa, MG 36570-000, Brazil

**Keywords:** genome evolution, insertion sequence, mobile DNA, transposon

## Abstract

The extensive genetic diversity of *Ralstonia solanacearum *, a serious soil-borne phytopathogen, has led to the concept that *R. solanacearum* encompasses a species complex [* R. solanacearum* species complex (RSSC)]. Insertion sequences (ISs) are suggested to play an important role in the genome evolution of this pathogen. Here, we identified and analysed transposable elements (TEs), ISs and transposons, in 106 RSSC genomes and 15 *Ralstonia* spp. We mapped 10 259 IS elements in the complete genome of 62 representative RSSC strains and closely related *Ralstonia* spp. A unique set of 20 IS families was widespread across the strains, IS*5* and IS*3* being the most abundant. Our results showed six novel transposon sequences belonging to the Tn*3* family carrying passenger genes encoding antibiotic resistance and avirulence proteins. In addition, internal rearrangement events associated with ISs were demonstrated in *Ralstonia pseudosolanacearum* strains. We also mapped IS elements interrupting avirulence genes, which provided evidence that ISs plays an important role in virulence evolution of RSSC. Additionally, the activity of ISs was demonstrated by transcriptome analysis and DNA hybridization in *R. solanacearum* isolates. Altogether, we have provided collective data of TEs in RSSC genomes, opening a new path for understanding their evolutionary impact on the genome evolution and diversity of this important plant pathogen.

## Data Summary

Genome data analysed in this work are available in the National Center for Biotechnology Information database. Individual accession numbers are listed in Table S1 (available with the online version of this article).

**Impact Statement**

*
Ralstonia solanacearum* is one of the most devastating plant pathogenic bacteria found worldwide. This soil-borne pathogen is composed of a large-scale group of strains varying in geographical distribution and pathogenic behaviour, known as the *R. solanacearum* species complex (RSSC). The observation of this heterogeneous group has led to the hypothesis that the mobile genetic elements (MGEs) may play an important role in shaping the genetic diversity of RSSC. The genome of *R. solanacearum* is organized into two circular replicons, a chromosome and a megaplasmid. Both replicons have a mosaic structure containing several MGEs, which may play relevant functions in the genome and virulence evolution of the pathogen. Here, we analysed a representative subset of 121 *Ralstonia* spp. genomes, including RSSC strains and *Ralstonia pickettii *, *Ralstonia mannitolilytica* and *Ralstonia insidiosa *, to investigate the repertoire of insertion sequences (ISs) and transposons. A great diversity of transposable elements (TEs) was found in the *Ralstonia* spp. genomes. A unique set of IS families was highly widespread across the strains. These findings have expanded our knowledge of the genetic basis of RSSC diversified adaptation based on its repertoire of TEs, and further studies are required to fully understand the evolutionary impact on genome evolution and pathogenicity of this important plant pathogen.

## Introduction

Plant–pathogen interactions are intimate, complex and ancient, having developed from a never-ending war [1, 2]. Understanding how plant pathogenic bacteria are evolving to overcome plant resistance is crucial for designing disease control strategies. However, many evolutionary aspects of plan–pathogen interaction remain understudied. In order to form an association with hosts, some bacterial genomes undergo remarkable variations, such as insertions, duplications, inversions and translocations, until a stable long-term association is formed [3, 4]. To some extent, this process can be achieved by the accumulation of repetitive DNA, including transposable elements (TEs), prophages and paralogous genes; many of which have been recognized as non-functional sequences, which can play an important evolutionary role in specialized host adaptation [5].

TEs have garnered research interest as several pathogens possess a relatively high numbers of these mobile elements, which may be responsible for a bottlenecking relationship between pathogen and host [3]. The bacterial TEs, transposons and insertion sequences (ISs) are self-replicable intracellular mobile genetic elements (MGEs). Typically, ISs have single or multiple ORFs that encode a transposase protein, required for insertion into a new locus. In general, ISs have terminal inverted repeats (TIR) and are flanked by short direct repeats (DRs). These elements are distinguished from transposons because transposons carry cargo genes not involved in catalysing or regulating TE movement [6]. IS elements are typically the smallest TEs (<2 kb), and dramatically shape genome content by causing mutations, insertions, deletions, inversions of DNA and alterations of gene expression [7].

This process is believed to represent a great source of genomic diversification, allowing rapid evolution of pathogens or stimulating the emergence of new pathogenic races causing diseases in plants and animals [8]. ISs might play a crucial role in the genome evolution of the bacterium *Ralstonia solanacearum *, a serious soil-borne phytopathogen effecting agricultural production due to its extensive host range and aggressiveness [9]. However, a complete analysis of the TEs in the *R. solanacearum* genome has not been reported.

The genome of *R. solanacearum* is organized into two circular replicons, a chromosome and a megaplasmid; both encode housekeeping and accessory genes. They have similar genomic features (dinucleotide relative abundances, codon usage, and distribution and composition of simple sequence repeats), suggesting their co-evolution over a long time span [9, 10]. Genome comparisons of representative strains of *R. solanacearum* showed that genomic features, such as size, G+C content and number of genes, were conserved across the strains; however, many genomic rearrangements (e.g. inversion and translocation), as well as deletion and insertion of DNA, were also demonstrated among the strains [11, 12].

Owing to genome differentiation, *R. solanacearum* species complex (RSSC), which includes *Ralstonia syzygii* and blood disease bacteria (BDB), was proposed to encompasses three distinct species: *Ralstonia pseudosolanacearum* (formerly phylotypes I and III), *R. solanacearum* (IIA and IIB) and *R. syzygii* (formerly phylotypes IV and BDB) [13, 14]. To investigate the impact of TEs on the genome evolution of RSSC, we identified and analysed the MGEs present in the genomes of 106 RSSC strains and 15 *Ralstonia* spp. collected from diverse plant hosts and geographical origins.

## Methods

### Genome data and detection of TE sequences

The genomes of 106 RSSC and 15 *Ralstonia* spp. (* Ralstonia pickettii *, *Ralstonia mannitolilytica *, *Ralstonia insidiosa *) were downloaded from the National Center for Biotechnology Information (NCBI; www.ncbi.nlm.nih.gov/genome) database in December 2018 (Table S1). Three different programs were used to identify IS elements. First, ISs were predicted by blastn [15] alignment against the ISfinder database, using default parameters (*E* value ≤10^−5^) [16], and a minimum alignment coverage of 50 % and with at least 70 % identity was considered. Next, two semi-automatic programs were used: ISsaga (insertion sequence semi-automatic genome annotation; http://issaga.biotoul.fr/ISsaga2/issaga_index.php) [17] and oasis (optimized annotation system for insertion sequences; https://github.com/dgrtwo/OASIS) [18]. All originally annotated IS elements were recovered from each program. The DRs, and TIR were manually identified and annotated using Geneious 11.1.5 (Biomatters) based blastn searches against ISfinder to identify known IS elements. An extensive survey of the IS elements within the *Ralstonia* ssp. genomes was analysed followed the *Everyman’s Guide to Bacterial Insertion Sequences* to identify partial IS copies and providing general features for each family [19]. Transposon sequences were identified by screening our local database of ISs to search for IS derivatives of transposons. We identified six sequences belonging to the Tn*3* transposon family. Using the reference sequence, the predicted sequence was inspected for DR and TIR sequences that define the boundaries of the transposon. The complete nucleotide sequence was imported into Geneious in the GenBank format of corresponding records to help delimit genomic regions flanking the element. These six transposon sequences were registered in The Transposon Registry [20] as Tn*6768*, Tn*6769*, Tn*6770*, Tn*6771*, Tn*6772* and Tn*6773*.

### Virulence and antimicrobial-resistance-associated genes in TEs

Virulence and antimicrobial-resistance genes next to TEs were identified by performing a blastp search (using the following parameters: *E* value ≤10^−5^; amino acid identity >30 %; coverage >100 amino acids) on the Pathogen–Host Interactions database (PHI-base; www.phi-base.org) [21] and Ralsto TE3 [22], and by a standard blastn search against the Comprehensive Antibiotic Resistance Database (CARD; http://card.mcmaster.ca) [23]. To assess the impact of IS elements in the virulence genes, they were classified into three groups: IS insertions within a virulence ORF; impartial virulence ORF (less than 100 nt distant); and nearby ORF encoding a virulence genes.

### Phylogenetic tree

The 16S rRNA gene sequences were obtained from the NCBI database and a distance matrix was constructed using ClustalW [24]. Subsequently, all the sequences were aligned and a phylogenetic tree was reconstructed in mega x [25] using maximum likelihood (1000 bootstrap replicates) and the substitution model Tamura–Nei+gamma distribution+invariable [25]. The generated output file (.tree) was visualized and annotated with the Interactive Tree of Life (iTOL) interface v4 (https://iTOL.embl.de/) [26].

### Expression of ISs in the RSSC transcriptome

A transcriptome (61 Gbp) from *R. solanacearum* strain UW163 (accession numbers SRX1436103–SRX1436108, SRX1435115–SRX1435118, SRX1435038 and SRX1435071) [27] was retrieved in fastq format from the NCBI Sequence Read Archive (SRA) (www.ncbi.nlm.nih.gov/sra) [28]. The expression profile of this strain was compared in basic minimal medium (BMM), casamino acid-peptone-glucose (CPG) liquid media (containing 1 g casein l^−1^, 10 g peptone l^−1^ and 5 g glucose l^−1^), and plant hosts (tomato, banana and melon) [27]. A quality check of the raw sequencing data was performed using the FastQC (v0.11.5) program and the reads were trimmed with Trimmomatic [29]. The alignment of quality trimmed data was performed using Bowtie2 version 2.2.8 [30]. The reads were mapped against reference genomes and the values were normalized with the edgeR 3.6.2 [31] library in RStudio and the gene fold change was calculated as log_2_(treatment/control – minimal medium). The expression of IS families found in the genome of UW163 was verified in the transcriptomic datasets.

### Integration profile analysis

Seven *R. solanacearum* strains isolated from soil samples were selected, as detailed in Table S2. The isolates were cultured at 28 °C with a shaking speed of 150 r.p.m. in CPG medium. The genomic DNA was extracted using a Wizard genomic DNA purification kit (Promega) according to the manufacturer’s recommendations, checked for quality using a NanoDrop 2000 (Thermo Scientific) instrument and subjected to gel electrophoresis. Probes for IS*1021* and ISRso*10* were prepared and detected using a PCR DIG probe synthesis kit (Hoffmann–La Roche). For Southern hybridization, 10 µg genomic DNA was digested with *Eco*RI and incubated overnight at 37 °C. DNA denaturation, neutralization and transference were performed according to the Sambrook and Russel method [32].

### Comparison of chromosomal rearrangements

Genome sequences of the strains KACC10722, T110 and SEPPX05 were obtained from NCBI in .gbk format, richness of IS copies in the chromosome being the major selection criteria. Multiple genome alignments were performed with Mauve software (version 2.3.1) [33], with the following parameters: alignment with progressive Mauve (aligner: Muscle 3.6); default seed weight (15); full alignment (minimum island size 50, maximum backbone gap size 50, minimum backbone size 50); use of seed families, yes; iterative refinement, yes; determination of locally collinear blocks (LCBs), yes).

## Results

### Great diversity of IS elements in the *Ralstonia* spp. genomes

**Fig. 1.**
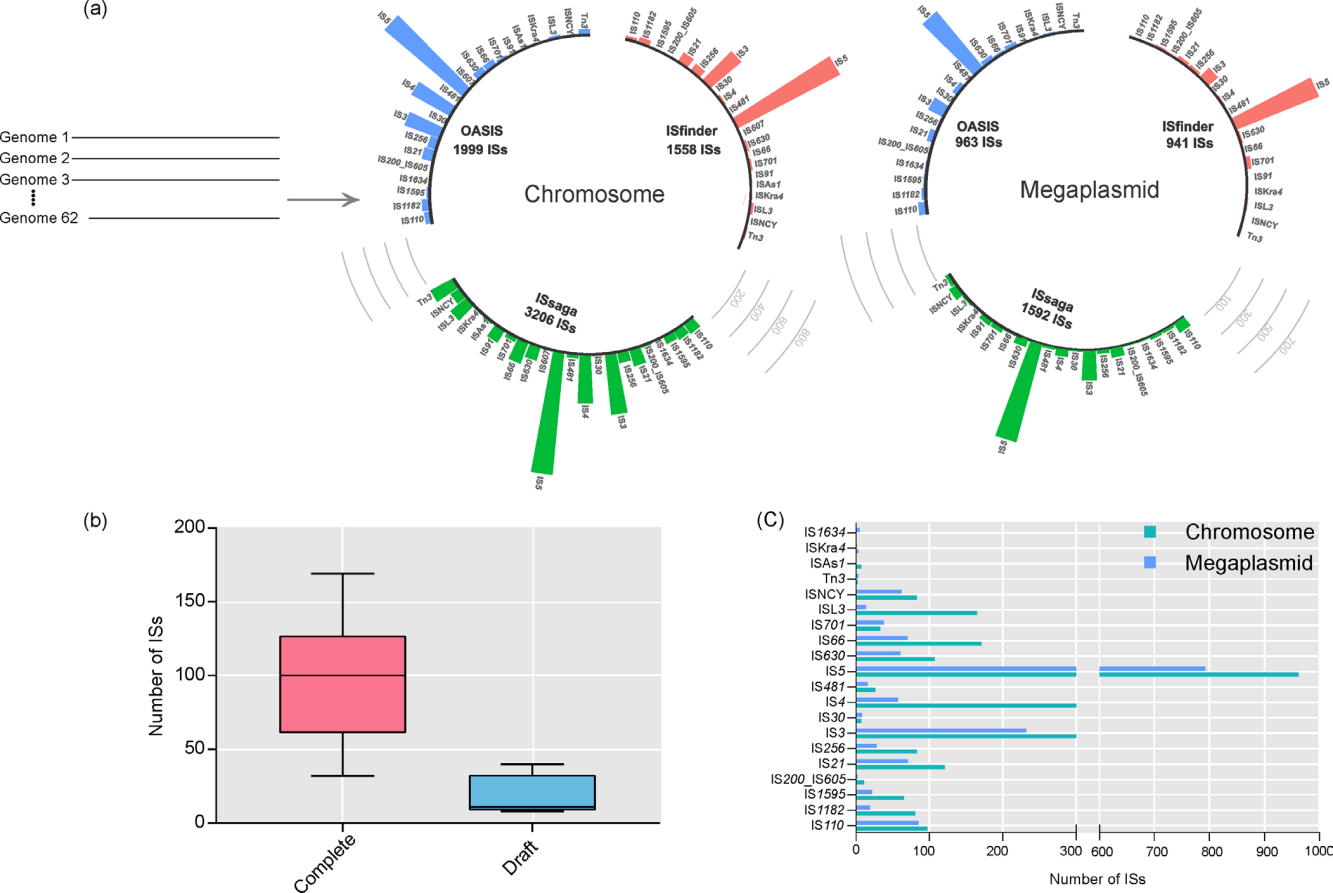
IS elements in *Ralstonia* spp. replicons. (a) A schematic diagram showing the IS numbers and families detected by the different computational tools. (b) Bar plot representing IS distributions in the complete and draft genomes. (c) Bar plot representing the distribution of IS families in the chromosome (green) and megaplasmid (blue).

Our analysis showed 10 259 IS elements in the chromosome and megaplasmid of 62 *Ralstonia* spp. complete genomes using ISsaga [17], ISfinder [16] and oasis [18] (Fig. 1a). An overview of IS distributions in the 60 draft genomes revealed the mean number to be lower than in the complete genomes (Fig. 1b), indicating the effect of genome assembly bias. Therefore, to avoid bias in the analysis, we opted to work only with complete genomes. The IS numbers and families detected varied according to the computational tool, ISsaga being efficient for automated annotation of a total of 3206 ISs in the chromosome and 1592 in the megaplasmid (Tables S3a/S3b/S3c and S4a/S4b/S4c, Fig. 1a). ISsaga found the greatest number of ISs, and also encompasses a set of IS families identified by ISfinder and oasis; therefore, our further analysis was performed with the ISsaga dataset. Details for each IS annotation computational tool are listed in Tables S3a, S3b, S3c, S4a, S4b and S4c. Subsequently, we computed the IS family distribution in the replicons of the complete genomes. Our results showed a unique set of 20 IS families across the chromosome and megaplasmid of the *Ralstonia* spp. (Fig. 1c).

### Description of the major IS families in *Ralstonia* spp.

The IS*5* family was the most abundant family found in the *Ralstonia* spp. genomes, followed by the IS*3*, IS*4*, IS*110* and IS*21* families (Table 1). A total of 1724 copies of IS*5* were found, of which 962 copies were identified in the chromosome and 762 in the megaplasmid sequence. A total of 256 copies for this family were identified as partial. The element sizes ranged from 850 to 1200 nt in length and have been divided into four subgroups (Table 1). The IS*3* family has 711 copies, of which 479 and 232 copies were found in the chromosome and megaplasmid, respectively. Within this family, 297 IS elements were identified as partial. The IS*3* family encompasses five subgroups ranging from 1000 to 1750 nt in length. At least one to three different IS*3* elements per genome were found. *Ralstonia* spp. genomes contain 436 copies of the IS*4* family, 379 in the chromosome and 57 in the megaplasmid. Fifteen elements were identified as partial. The IS*4* family encompasses only two subgroups (IS*4* and IS*50*) ranging from 1110 to 1359 nt in length. One to three different ISs were found per genome. In total, 162 copies were identified as belonging to the IS*110* family, of which 57 copies were in the chromosome and 105 in the megaplasmid. Also, 17 IS elements were identified as partial. IS*110* members encoded a single ORF with size ranging from 1200 to 1253 nt. This family encompasses one subgroup (IS*1111*). At least one to two different IS elements per genome were found within this family. Moreover, 167 copies of IS*21* family were identified, of which 121 were located in the chromosome and 46 in the megaplasmid. At least one to two different elements per genome were found in our dataset. Besides these five families describe here, another 13 families are listed in detail in Table 1.

**Table 1.** Characteristics of IS elements found in the *Ralstonia* spp. genomes

			**Number of copies**				
**Family**	**Subgroup**	**Size range (nt)**	**Chromosome**	**Megaplasmid**	**Different IS(s**)	**Partial**	**Total**
IS*21*	–	1700–1800	121	46	1–2	44	167
IS*3*	1000–1750	479	232	1–3	297	711
							IS*3*
							IS*407*
							IS*51*
							IS*150*
							IS*2*
IS*4*	1100–1359	379	57	1–2	15	436
							IS*4*
							IS*50*
IS*5*	IS*5*	850–1200	962	793	1–3	226	1755
	IS*1031*						
	IS*427*						
	IS*903*						
IS*110*	IS*1111*	1200–1253	57	105	1–2	17	162
IS*1182*	IS*1016*	1330–1578	57	18	1–3	14	75
ISH*4*
IS*1595*	ISPna*2*	700–1287	49	39	1–2	22	88
	ISSod*11*						
IS*256*	–	1200–1269	11	16	1–2	13	27
IS*701*	–	1200–1500	33	38	1–2	7	71
ISL*3*	–	1050–3000	165	14	1–2	35	179
ISNCY	IS*1202*	1400–2000	83	62	1–2	53	145
Tn*3*	–	1200–3000	201	30	1–2	225	231
ISAs*1*	–	1200–1500	7	–	1–3	–	7
ISKra*4*	–	1200–1500	3	1	1–3	2	4
IS*30*	–	102–1071	7	9	1	2	16
IS*481*	–	225–1968	19	17	1–2	7	36
IS*630*	–	237–1128	59	61	1–2	48	120
IS*66*	–	1515–2181	49	44	1–3	146	93

### IS families are widespread throughout the RSSC strains

**Fig. 2.**
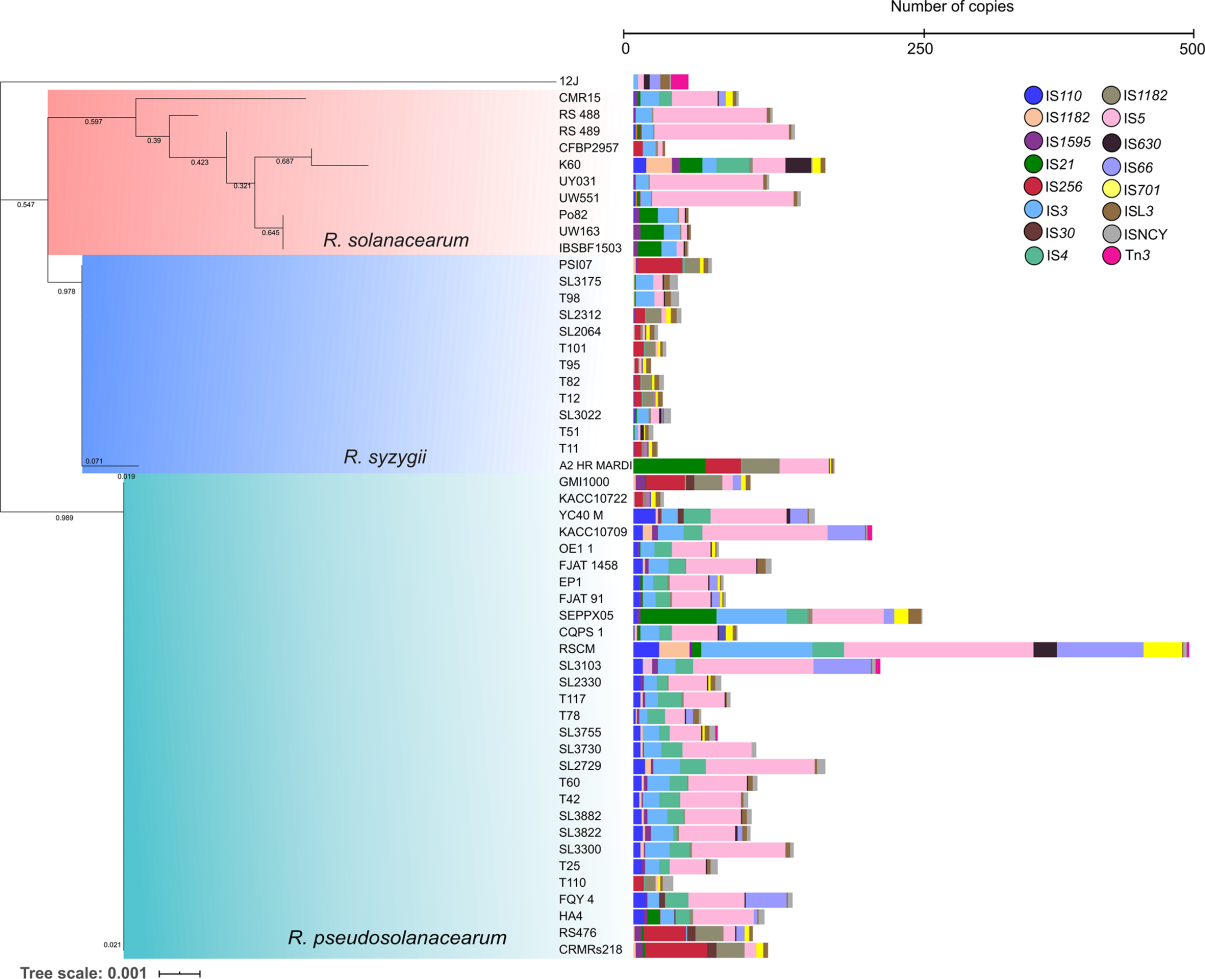
Representation of IS family distribution in a RSSC phylogenetic context based on the 16S rRNA gene. The phylogenetic tree was generated with the maximum-likelihood method using mega x software (1000 bootstrap replications) and the substitution model Tamura–Nei+gamma distribution+invariable. The tree is drawn to scale, with branch lengths in the same units as those of the evolutionary distances used to infer the phylogenetic tree. The tree was visualized and annotated using iTOL.

Comparisons of IS families between corresponding sets of *Ralstonia* ssp. complete genomes revealed the pattern of IS families among the RSSC strains (Fig. 2). The majority of IS families are widespread throughout the complex, the IS*5* and IS*3* families being shared by all RSSC genomes. Closely related strains tend to have similar patterns of ISs. However, several species-specific IS elements were noticed: such as IS*30* only shared among six *R. pseudosolanacearum* strains; the IS*4* and IS*701* families mostly found in *R. pseudosolanacearum* strains, only shared by one *R. solanacearum* strain K60; most *R. syzygii* strains lack a set of IS*110*, IS*256* and IS*66* families, only found in one genome. Altogether, *R. pseudosolanacearum* strains shared numerous and diverse IS elements (*n*=3912), followed in number by *R. solanacearum* (*n*=855) and *R. syzygii* strains (*n*=559). A set of IS families found in 62 genomes of *Ralstonia* spp. were characterized in detail (Table S5).

### Characterization of six novel putative transposons in *R. pseudosolanacearum* genomes

**Fig. 3.**
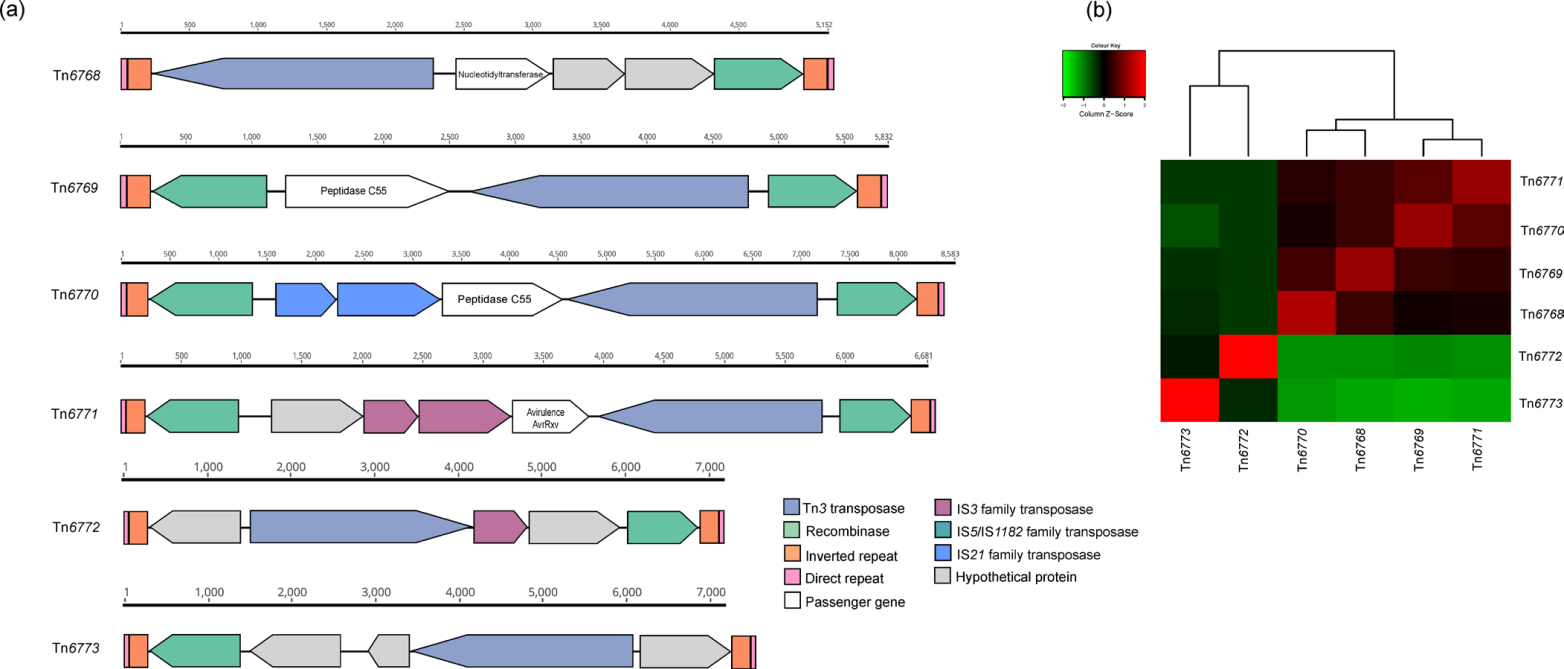
Characterization of six novel transposons. (a) Schematic representation of six transposons belonging to the Tn*3* family located in *R. pseudosolanacearum* strains RSSCM, HA4-1, KACC10709 and SL3103. Genes are indicated by coloured boxes, with the direction of transcription shown by the arrowheads. Transposition-related genes, passenger genes and terminal inverted repeats are as detailed in the key. (b) Heatmap of pairwise comparisons of the nucleotide sequences of the novel putative transposons. The colours represent the mean similarity values for the sequences, as shown in the key.

Tn*6768*, Tn*6769*, Tn*6770*, Tn*6771*, Tn*6772* and Tn*6773* are the novel putative transposons belonging to the Tn*3* family identified in four *R. pseudosolanacearum* strains (RSCM, HA4-1, KACC10729 and SL3103) (Fig. 3a). Tn*6768* and Tn*6769* were found in the RSCM chromosome and megaplasmid, respectively; Tn*6770* and Tn*6771* were found in the megaplasmid and plasmid of the strain HA4-1, respectively. Tn*6772* and Tn*6773* were identified in the chromosome and megaplasmid sequences of strains SL3103 and KACC10709. The length of the transposon sequences ranged from 5.1 to 8.5 kb (Fig. 3a). Together the six transposons identified here shared 24 to 86 % of sequence identity, and were exclusively found in these three Chinese strains and one Korean strain (Fig. 3b). Commonly, all the transposons encode the Tn*3* transposase family and recombinase proteins, which ensures the transposition process. Tn*6768*, Tn*6769*, Tn*6770* and Tn*6771* encode a serine recombinase family and they are flanked by a typical Tn*3* family IRL sequence of 51 bp long (GGGGCCGTCTCAGAAAACGGAAAAAATCGTACGCTAAGCCCGGGTTGATGC), an IRR sequence of 42 bp (GGGGTCGTCTCAGAAAACGGAAAAAATCGTACGCTAAGCTCG) and an 8 bp long DR (CAAGATGG). However, Tn*6772* and Tn*6773* encode a putative tyrosine recombinase XerC-like and they are flanked by an IRL sequence of 51 bp long (AGCGTCTCGTGCAGCGCGGGATGGTCGCGATTAATCTGAAGGGGCGATCTT), an IRR sequence of 51 bp long (CATTGAGTCATGATTTTGACGAGTTTTATGCCTTG ATGGAATAAAGACCGA) and an 8 bp long DR (CCATAAGC). The Tn*6768* includes two hypothetical proteins and nucleotidyltransferase genes as passengers. Tn*6769* contains an additional recombinase and a passenger gene encoding peptidase C55. Tn*6770*, in addition to an extra recombinase and passenger genes encoding peptidase C55, also contains the IS*21* family transposase. Tn*6771* carries a pair of IS*5* family transposases, a hypothetical protein gene and an additional passenger gene encoding avirulence effector protein, AviRxv (Fig. 3a). Tn*6772* and Tn*6773* include hypothetical proteins as passenger genes. We believe this is the first study reporting transposon elements in the RSSC genome.

### ISs mediate genomic rearrangements

**Fig. 4.**
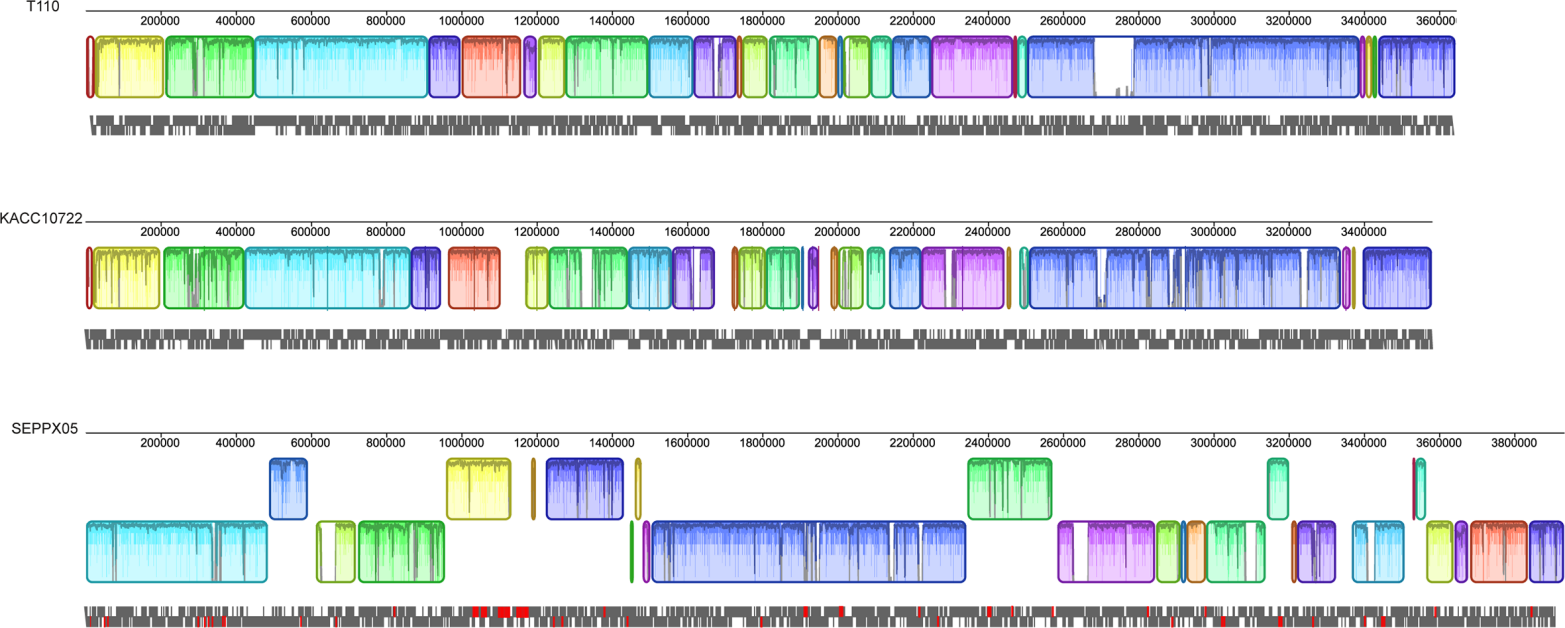
Mauve alignment of the three *R. pseudosolanacearum* genomes revealing numerous internal rearrangements in the strain SEPPX05. Coloured blocks represent co-linear blocks. Multiple genome alignments were performed by the Mauve software. IS*21* family annotations are indicated by the red boxes, where available.

IS elements can shape genomic rearrangements by causing insertions, deletions and inversions [34]. Three *R. pseudosolanacearum* strains (SEPPX05, KACC10722 and T110) were selected. KACC10722 had 17 complete IS copies, while T110 had 57 IS copies on the chromosome sequence. Notably, these two strains share collinear syntenic blocks. *R. pseudosolanacearum* strain SEPPX05 had 156 IS copies. In genomes possessing a higher number of IS copies, these elements might have a larger impact on the sequence. Our analysis revealed numerous internal rearrangements in *R. pseudosolanacearum* strain SEPPX05, with a subset being mostly associated with repeated IS*21* elements (Fig. 4), which is indicative that recombination between these ISs might be the cause of the rearrangements.

### TEs linked to virulence-encoding regions

**Fig. 5.**
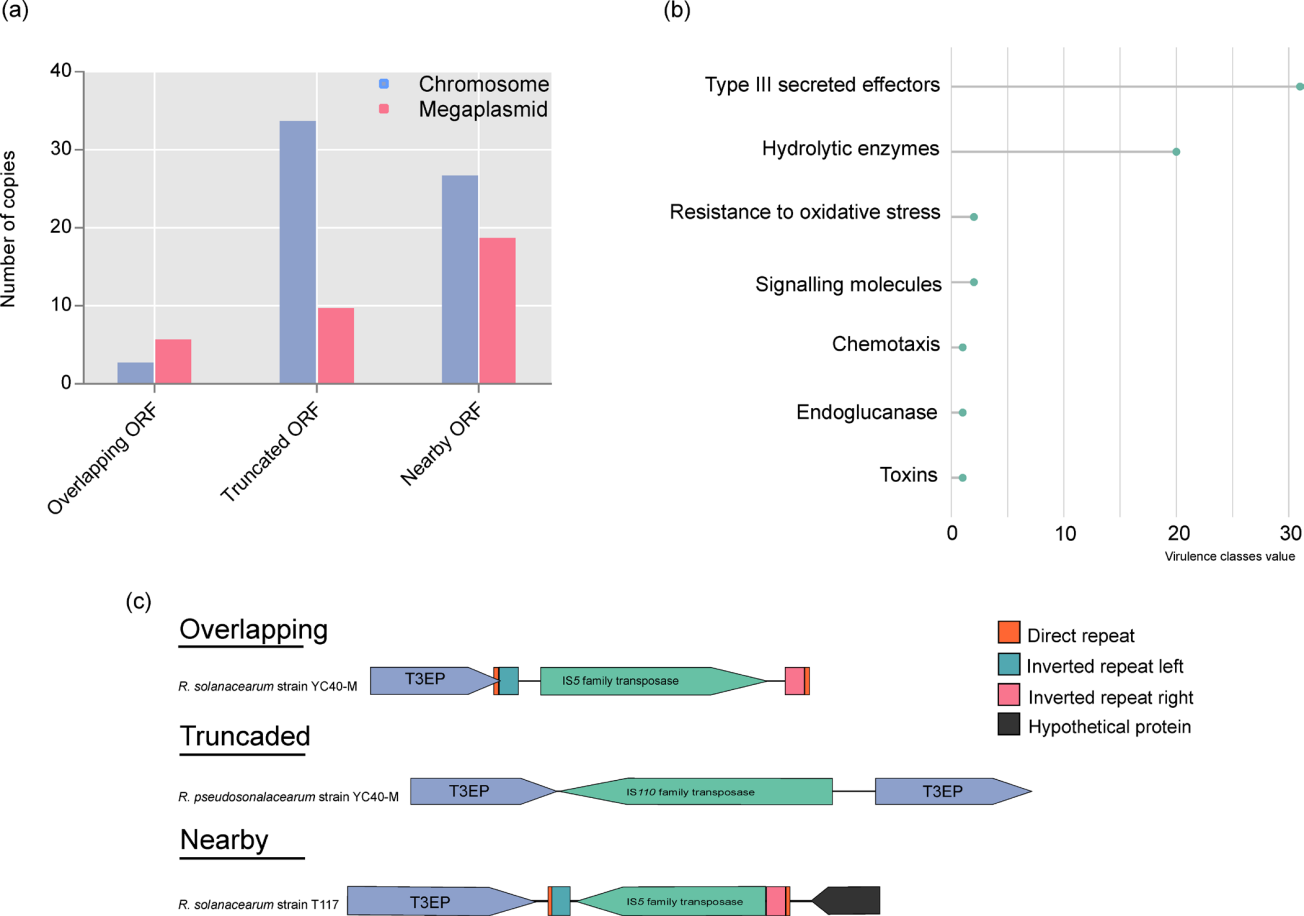
ISs in the intergenic regions of virulence genes. (a) Dot plot showing the insertion of these elements for the three classes across the chromosome and megaplasmid sequence. (b) Lollipop chart showing the virulence classes value. (c) Schematic representation of three examples, representing the different classes, mapped in two strains of RSSC.

It is believed that for pathogens under a bottlenecking relationship with hosts, IS elements have a strong effect on their genome [35]. Therefore, we paid special attention to the genome context for each annotated IS in intergenic regions of RSSC virulence factors. Most of the elements were found to be truncated, inserted nearby or overlapping genes of virulence factors mainly found in the chromosome sequence (Fig. 5a). Most flanking genes were within type III secreted effectors, including a number of genes encoding type III effector proteins (T3EPs), hydrolytic enzymes (haemagglutinin-related genes), resistance to oxidative stress, signalling molecules, chemotaxis, endoglucanase gene and toxins (Fig. 5b). Details of the flanking genes are found in Table S6. Analysis revealed that 49 % (*n*=31) of the T3EP genes may be affected by ISs. Fig. 5c illustrates three examples, representing the three classes, mapped across the RSSC genomes. In *R. pseudosolanacearum* strain YC40-M, an IS*5* is present within a T3EP gene, and an IS*110* element disrupts another T3EP gene. An intergenic region is present upstream of a T3EP gene and downstream of gene encoding a hypothetical protein in *R. pseudosolanacearum* strain T117. A T3EP gene disrupted by ISs represented the most common flanking gene. Subsequently, we performed a blastx analysis of T3EP against the PHI-database [21] and Ralsto T3E database [22] to characterize the genes. More than half of the T3EP genes were identified as avirulence genes (*avr*).

### Comparative transcriptomics for expression of IS elements *in planta* for host-adapted *R. solanacearum*

TEs have been reported to play an important role during plant–pathogen interactions, as these elements increase microbial genetic variability and rapidly adapt to environmental changes [36]. However, little is known about the similarity or difference of the effect of these elements on the bacterial transcriptome under *in vitro* and *in planta* conditions. To address this question, we investigated transcriptome patterns of IS sequences using an *in planta* comparative RNA-seq dataset [27]. Gene expression for the *R. solanacearum* strain UW163 was studied in rich media and under *in planta* conditions during the colonization of banana, melon and tomato (Fig. S1). We observed that even though all IS elements were expressed under *in vitro* and *in planta* conditions, more genes were up-regulated for the *in planta* dataset than in the rich media dataset. Clearer host genotype effects were observed for ISs up-regulated during tomato and banana infection, which were down-regulated in melon plants, indicating the role of IS elements in the host adaptation of *R. solanacearum* (Fig. S1).

### IS element activity in *R. solanacearum* isolates

*In silico* analysis indicated several IS copies. We searched for evidence of such elements *in vitro* in the *R. solanacearum* population. We selected seven *R. solanacearum* isolates from soil samples in Minas Gerais and Brasília, Brazil (Fig. S2a, Table S2), and performed Southern blotting using IS*5* family transposase IS*1021* and IS*3* family transposase ISRso*10* elements as probes (Fig. S2b). Most of the isolates showed the hybridization pattern for IS*1021* and ISRso*10* elements (Fig. S2c), suggesting the presence of these elements in the Brazilian *R. solanacearum* isolates. Within this population, we detected great polymorphism in the number of copies including isolates with no IS hybridization pattern.

## Discussion

We report a curated TE identification method in 121 genomes of the RSSC and closely related *Ralstonia* spp. (* R. pickettii *, *R. insidiosa *, *R. mannitolilytica *). Our analysis found the majority of TEs in the complete genome sequences. However, these elements were also mapped in a low number in the draft genomes, which might be related to genome assembly artifacts that tend to occur near repetitive genomic regions resulting in only one contig with the elements collapsed [37]. Therefore, to avoid bias in the analysis, we opted to work only with complete genomes. In total, 10 259 IS elements were mapped using ISsaga, ISfinder and oasis. We showed that *Ralstonia* spp. shared a unique set of IS families, mainly IS*5* and IS*3*. IS*5* is a relatively heterogeneous group, the majority of its members having a single transposase (Tpase), but also some members may express Tpase by frameshifting [19]. The IS*3* family has been found in 270 bacterial species, has over 554 members and is characterized by fusion ORF programmed translational frameshifting with 1200 and 1550 bp long sequences and inverted terminal repeats in the range of 20 to 40 bp [6, 19].

Especially for the RSSC, closely related strains tend to have similar patterns of IS elements. IS elements have demonstrated the ability to quickly multiply in genomes, resulting in a similar number of IS elements in closely related strains [37]. We noticed that *R. pseudosolanacearum* strains share numerous and diverse IS families. This study reflects on the coverage of these elements in their genome; for example, ISs constitute 3.9 % of the *R. pseudosolanacearum* strain SEPPX05 genome.

In addition to IS elements, we also report the presence of six novel transposon sequences that belonging to the Tn*3* family. These transposons were found in three Chinese *R. pseudosolanacearum* strains (RSSCM, HA4-1 and KACC10709) and one Korean strain (SL3103). Interestingly, these transposons were only found in these strains. The transposon Tn*6768* encodes the enzyme aminoglycoside nucleotidyltransferase in a passenger gene that confers resistance to a wide range of aminoglycosides, such as kanamycin A, and acts by transferring the nucleoside monophosphate group from a nucleotide to the 4′-hydroxyl group of kanamycin A [38, 39]. Although the wilt disease caused by *R. solanacearum* is not managed with antibiotics, our results showed acquisition of antibiotic resistance in this important phytopathogen. This is critical knowledge, because antibiotic-resistance genes are transferred by the mobile elements, which potentially might be acquired by other bacteria in the environment via horizontal gene transfer.

Putative *avr* genes were mapped as passengers in the sequence of Tn*6771*. The term ‘*avr* genes’ indicates an effector gene that encodes a determinant specifically to interact with the host [40]. Therefore, horizontal gene transfer of *avr* is recognized as a major epidemiological factor in new disease outbreaks [41], suggesting the role of these transposons in the pathogenicity of RSSC. Our findings demonstrated a collective data, showing the potential impact of these elements on *Ralstonia* host range. In this study, a large number of *avr* genes interrupted by ISs were found. As described by the gene-for-gene theory, *avr* genes are key determinants during plant–pathogen interactions [42]. The theory relies on the relationship between pathogen and host plant cultivars, this interaction occurs between an *avr* gene in the pathogen and an *R* (resistance) gene in the plant. When a pathogen possessing an *avr* attacks the plant that carries the corresponding *R* gene, resistance is induced in the plant, protecting it from the pathogen. Therefore, the inactivation of *avr* genes in bacteria can lead to virulence in a resistant host plant [40, 43]. Similarly, ISs have been found to interrupt *avr* genes in *Pseudomonas syringae* [44, 45] and be the mechanism of emergence of *Fusarium oxysporum* races as was demonstrated by Inami *et al*. [46]. In conclusion, these results provide evidence of MGEs as one of the driving forces for RSSC diversity.

As we demonstrated, in the genome of *R. pseudosolanacearum* strain SEPPX05, with a high number of IS copies, these elements have a strong influence on its organization, compared with two other *R. pseudosolanacearum* strains (KACC10722 and T110) with low numbers of copies. SEPPX05 had deletions, insertions and inversions, compared to most representative RSSC strains [47]. In addition, the strains KACC10722 and T110 are pathogenic to potato, and all three strains cause very high economic damage to crops in China [48].

Having shown the effect of IS elements in genome plasticity, we looked more closely at the impact of ISs in modulating RSSC virulence genes. Most IS elements were found in intergenic regions of genes encoding haemagglutinin-related protein, a class of adhesins produced by diverse pathogenic bacteria, responsible for the adhesion of bacteria during plant–pathogen interaction [49]. IS transposition is believed to activate the expression of a gene whose insertion creates an alternative promoter for the host gene or results in read-through transcription [34]. We mapped a few examples of insertions within virulence ORFs, such as ISs overlapping genes encoding haemolysin-like and type II secretion system family proteins, suggesting a possible interference of ISs in the transcription of these genes. Jeong and Timmis [50] reported transposition mediated by ISRso*4* (IS*5* family) in *R. solanacearum *, the inactivation of the global regulatory gene *phcA* modulated the expression of extracellular polysaccharides. Similarly, one IS was screened in the FJAT-1458 genome inserted into a *phcA* gene. FJAT-1458 is an avirulent strain and might be of use as a potential biocontrol agent as a plant vaccine for bacterial wilt [51].

Studies of the effect of IS transposition on phenotypic traits in bacteria have revealed a major modulation of IS expression under stress conditions [34, 37]. However, the mechanism of IS effects on pathogens under *in planta* conditions remains understudied. In this study, *in planta* bacteria RNA-seq data was used to link the expression of IS elements under *in planta* conditions. We found that while the IS elements were expressed both *in vitro* and *in planta*, the genes were up-regulated under *in planta* conditions compared with under rich media conditions. During plant–pathogen interactions, pathogens are challenged by abiotic and biotic stresses, such as reactive oxygen species, stress hormones, stress temperature, etc. [52]. A common feature of most ISs is that they are activated by stress and environmental factors [34]. Therefore, their transposition facilitates the establishment of the genetic variability that is required for adaptation [36]. This is the first evidence of IS activation in *R. solanacearum* under *in planta* conditions, suggesting the significant contribution of these elements to pathogen adaption.

The IS elements predicted *in silico* were assessed *in vitro* by analysing two IS elements in seven isolates of *R. solanacearum* from Brazil. The observed band polymorphism led to the hypothesis that these elements are involved in diversification [53]. Our analysis showed the widespread distribution of predicted IS elements *in silico* and *in vitro*, among *R. solanacearum* isolates. This might also indicate a recent activity of IS elements among the *R. solanacearum* population from Brazil. In conclusion, the research described here opens up new avenues for understanding the evolutionary impact of TEs on the genome evolution and diversity of the RSSC.

## Data Bibliography

1. Xu J, Zheng H, Liu L *et al*. GenBank assembly no. GCA_000215325.1 (2011).

2. Guarischi-Sousa R, Puigvert M, Coll NS *et al*. GenBank assembly no. GCA_001299555.1 (2016).

3. Remenant B, Coupat-Goutaland B, Guidot A *et al*. GenBank assembly no. GCA_000197855.1, GCA_000427195.1 and GCA_000283475.1 (2010).

4. Bocsanczy AM, Huguet-Tapia JC, Norman DJ. GenBank assembly no. GCA_000525615.1 (2014).

5. Hayes MM, MacIntyre AM, Allen C. GenBank assembly no. GCA_001696875.1, GCA_002251695.1 and GCA_000285815.1 (2017).

6. Yuan K, Cullis J, Lévesque CA *et al*. GenBank assembly no. GCA_000710135.3 and GCA_000710695.1 (2015).

7. Patil VU, Girimalla V, Sagar V *et al*. GenBank assembly no. GCA_001373295.1 (2017).

8. Kotorashvili A, Meparishvili G, Gogoladze G *et al*. GenBank assembly no. GCA_002029865.1, GCA_002029885.1 and GCA_002029895.1 (2017).

9. Salanoubat M, Genin S, Artiguenave F *et al*. GenBank assembly no. GCA_000009125.1 (2002).

10. Chen D, Liu B, Zhu Y *et al*. GenBank assembly no. GCA_001887535.1 (2017).

11. Li P, Wang D, Yan J *et al*. GenBank assembly no. GCA_001891105.1 (2016).

12. Chen D, Liu B, Zhu Y *et al*. GenBank assembly no. GCA_002155245.1 (2017).

13. Liu Y, Tang Y, Qin X *et al*. GenBank assembly no. GCA_002220465.1 (2016).

14. Cao Y, Tian B, Liu Y *et al*. GenBank assembly no. GCA_000348545.1 (2013).

15. Ramesh R, Gaitonde S, Achari G *et al*. GenBank assembly no. GCA_000671315.1 and GCA_000671335.1 (2014).

16. Li X, Huang X, Chen G *et al*. GenBank assembly no. GCA_002162015.1 (2018).

17. Cho H, Song E-S, Heu S *et al*. GenBank assembly no. GCA_003515205.1, GCA_003515205.1, GCA_003515225.1, GCA_003515245.1, GCA_003515285.1, GCA_003515305.1, GCA_003515345.1, GCA_003515365.1, GCA_003515405.1, GCA_003515465.1, GCA_003515545.1, GCA_003515565.1, GCA_003515585.1, GCA_003515605.1, GCA_003515165.1, GCA_003515185.1, GCA_003515265.1, GCA_003515325.1, GCA_003515385.1, GCA_003515425.1, GCA_003515445.1, GCA_003515485.1, GCA_003515505.1 and GCA_003515525.1 (2019).

18. Tan X, Qiu H, Li F *et al*. GenBank assembly no. GCF_003999725.1 (2019).

19. Shan W, Yang X, Ma W *et al*. GenBank assembly no. GCA_000430925.2 and GCA_001876985.1 (2013).

20. Zou C, Wang K, Meng J *et al*. GenBank assembly no. GCA_001484095.1 (2016).

21. Albuquerque GMR, Souza EB, Silva AMF *et al*. GenBank assembly no. GCA_003595305.1 and GCF_003612975.1 (2017).

22. Remenant B, Coupat-Goutaland B, Guidot A. GenBank assembly no. GCF_000197855.1, GCF_000283475.1 and GCF_000427195.1(2010).

23. Badrun R, Abu Bakar N, Laboh R *et al*. GenBank assembly no. GCA_002012345.1 (2017).

24. Remenant B, de Cambiaire J-C, Cellier G *et al*. GenBank project no. PRJNA369602 (2011).

25. Daligault HE, Davenport KW, Minogue TD *et al*. GenBank assembly no. GCA_000743455.1 (2014).

26. Vaz-Moreira I, Tamames J, Martínez JL, Manaia CM. GenBank assembly no. GCA_001699815.1 and GCA_001699795.1 (2016).

27. Paterson J, Gross H. GenBank assembly no. GCA_002516395.2 (2018).

28. Ohtsubo Y, Fujita N, Nagata Y *et al*. GenBank assembly no. GCA_000471925.1 (2013).

29. Xu J, Zheng H, Liu L *et al*. GenBank assembly no. GCA_001663855.1 and GCA_001653935.1 (2016).

## Supplementary Data

Supplementary material 1
