# Supplementary material for: Transposable elements contribute to the genome plasticity of Ralstonia solanacearum species complex
Source: Microb Genom. 2020 May 7;6(5):e000374. doi: 10.1099/mgen.0.000374 (PMC7371123; doi:10.1099/mgen.0.000374)
Supplement: Supplementary material 1 [file mgen-6-374-s001.pdf]

Transposable elements contribute to the genome plasticity of *Ralstonia solanacearum* Species Complex

Osiel Silva Gonçalves[1], Kiara França Campos[1], Jéssica Catarine Silva de Assis[1], Alexia Suellen Fernandes[1], Thamires Santos Souza[1], Luiz Guilherme do Carmo Rodrigues[1], Marisa Vieira de Queiroz[1], Mateus Ferreira Santana[1]*

[1]Departamento de Microbiologia, Instituto de Biotecnologia Aplicada à Agropecuária (BIOAGRO), Universidade Federal de Viçosa, Viçosa, MG, 36570-000; Brazil

*Corresponding author: Mateus Ferreira Santana

E-mail address: mateus.santana@ufv.br

Phone: +55 (31) 3612-2452

Supplementary figures legends

Fig. S1. Heat map of IS*401*, ISRso*19* and ISRso*10* transposase genes expressed between three plant hots and rich medium. Scaled expression values are color-coded according to the legend below.

Fig. S2. Evidence of IS elements activity in *R. solanacearum* isolates. (a) Seven *R. solanacearum* were isolated from soil samples in the state of Minas Gerais and Distrito Federal. (b) IS*1021* and ISRso*10* were used as probes. Arrows indicated the primers in the direction forward (F) and reverse (R). (c) Southern hybridization membrane for the probe IS*1021* and ISRso*10*.

Fig. S1.

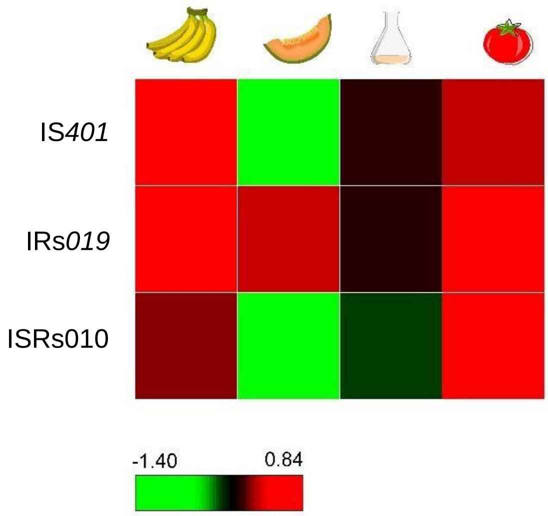

Fig. S2.

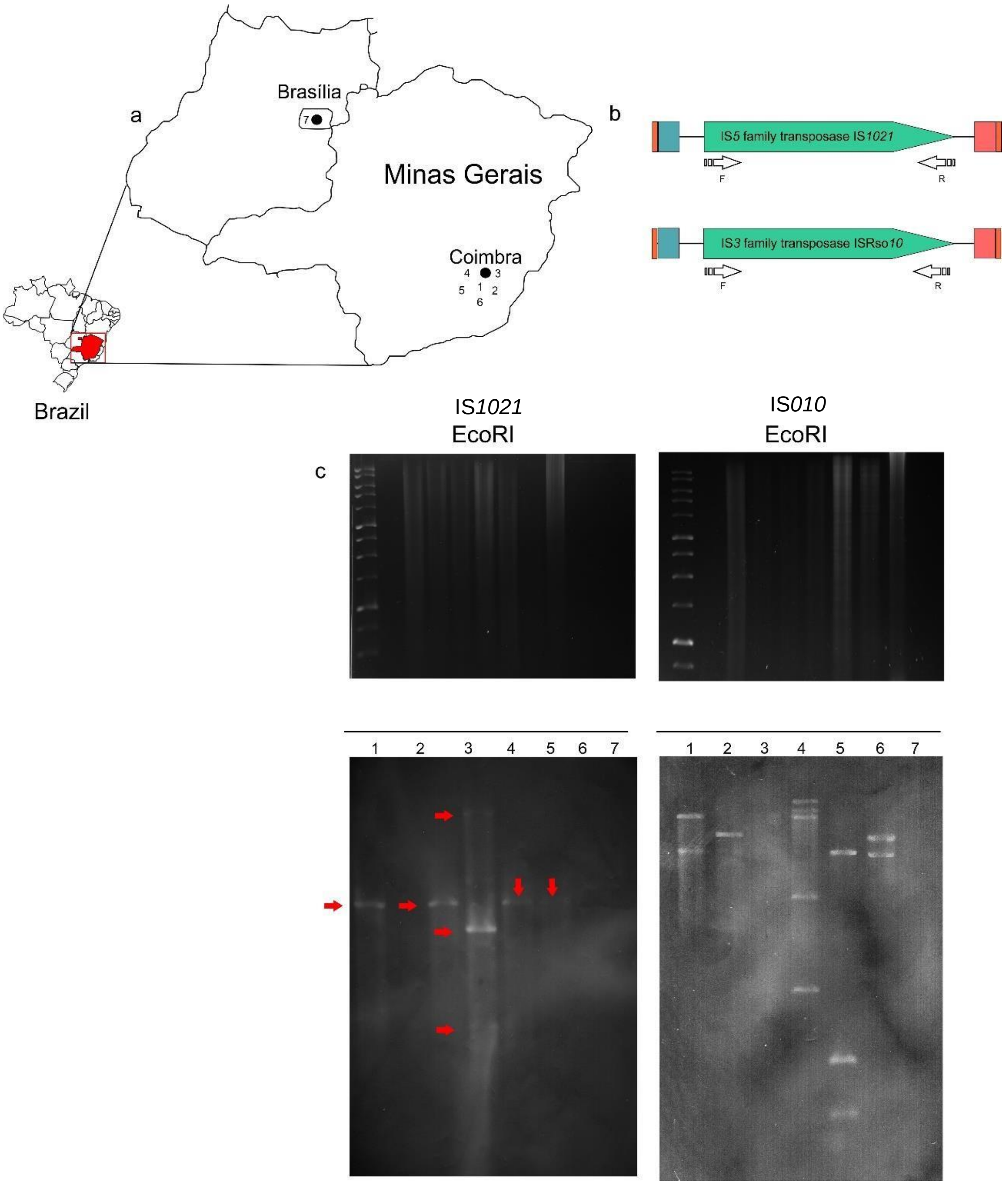
a
Brasília
7
Minas Gerais
Coimbra
4 3
5 1 2
6
Brazil
b
IS5 family transposase IS1021
F
R
IS3 family transposase ISRso10
F
R
IS1021
EcoRI
IS010
EcoRI
c
1 2 3 4 5 6 7
1 2 3 4 5 6 7

Table S1. *Ralstonia* spp. genomes used in this work.

Strain	Assembly	Status	Phylotype	Origin	Reference
Ralstonia solanacearum (phylotype IIA and IIB)					
Po82	GCA_000215325.1	Complete Genome	IIB	Mexico	Xu et al., 2011
UY031	GCA_001299555.1	Complete Genome	IIB	Uruguay	Guarischi-Sousa et al., 2016
UW163	GCA_001587135.1	Complete Genome	IIB	Peru	-
IBSBF1503	GCA_001587155.1	Complete Genome	IIB	Brazil	-
RS488	GCA_002501565.1	Complete Genome	II	Brazil	-
RS489	GCA_002549815.1	Complete Genome	II	Brazil	-
CFBP2957	GCA_000197855.1	Complete Genome	IIA	French West Indies	Remenant et al., 2010
MolK2	GCA_000212635.2	Draft	IIB	Filipina	-
P673	GCA_000525615.1	Draft	IIB	USA	Bocsanczy et al., 2014
23-10BR	GCA_000749995.1	Draft	IIB	Brazil	-
NCPPB 282	GCA_000750575.1	Draft	IIB	Colombia	-
POPS2	GCA_000750585.1	Draft	IIB	China	-
CIP120	GCA_001644795.1	Draft	IIA	Peru	-
P597	GCA_001644805.1	Draft	IIA	USA	-
CFBP6783	GCA_001644815.1	Draft	IIB	Martinica	-
UW491	GCA_001696845.1	Draft	II	Colombia	-
UW24	GCA_001696855.1	Draft	II	Israel	-
UW365	GCA_001696865.1	Draft	II	China	-
UW551	GCA_001696875.1	Complete Genome	IIB	USA	Hayes et al., 2017
UW25	GCA_002251695.1	Draft	II	USA	Hayes et al., 2017
K60	GCA_000285815.1	Complete Genome	IIA	USA	Hayes et al., 2017
Y45	GCA_000223115.2	Draft	IIB	China	-
IPO1609	GCA_001050995.1	Draft	IIB	Netherlands	-
CFIA906	GCA_000710135.3	Draft	II	Colombia	Yuan et al., 2015

NCPPB 909	GCA_000710695.1	Draft	IIB	Colombia	Yuan et al., 2015
B50	GCA_000825785.2	Draft	IIA	Peru	-
CIP417	GCA_000825825.2	Draft	IIB	Filipinas	-
Grenada 9-1	GCA_000825845.2	Draft	IIA	Granada	-
CFBP1416	GCA_000825925.2	Draft	IIB	Costa Rica	-
CFBP7014	GCA_001373255.1	Draft	IIB	Trindade	-
IBSBF1900	GCA_001373275.1	Draft	IIA	Brazil	-
RS2	GCA_001373295.1	Draft	IIB	India	Patil et al., 2017
GEO_99	GCA_002029865.1	Draft	-	USA	Kotorashvili et al., 2017
GEO_57	GCA_002029885.1	Draft	-	USA	Kotorashvili et al., 2017
GEO_96	GCA_002029895.1	Draft	-	USA	Kotorashvili et al., 2017
GEO_6	GCA_002894765.1	Draft	-	USA	-
GEO_304	GCA_002894775.1	Draft	-	USA	-
GEO_81	GCA_002894785.1	Draft	-	USA	-
GEO_230	GCA_002894795.1	Draft	-	USA	-
GEO_55	GCA_002894845.1	Draft	-	USA	-
UW181	GCA_001373315.1	Draft	IIA	Venezuela	-
58_RSOL	GCA_001065525.1	Draft	-	USA	-
UW179	GCA_000825805.2	Draft	IIA	Colombia	-
CFBP3858	GCA_001373335.1	Draft	IIB	Netherlands	-
Ralstonia pseudosolanacearum (Ralstonia solanacearum phylotype I/III*)*					
GMI1000	GCA_000009125.1	Complete Genome	I	French Guiana	Salanoubat et al. 2002
OE1-1	GCA_001879565.1	Complete Genome	I	Japan	-
FJAT-1458	GCA_001887535.1	Complete Genome	I	China	Chen et al., 2017
EP1	GCA_001891105.1	Complete Genome	I	China	Li et al., 2016
FJAT-91	GCA_002155245.1	Complete Genome	I	China	Chen et al., 2017
CQPS-1	GCA_002220465.1	Complete Genome	I	China	Liu et al., 2016

RSCM	GCA_002894285.1	Complete Genome	I	China	-
FQY_4	GCA_000348545.1	Complete Genome	I	China	Cao et al., 2013
Rs-10-244	GCA_000671315.1	Complete Genome	I	India	Ramesh et al., 2014
SEPPX05	GCA_002162015.1	Complete Genome	I	China	Li et al., 2018
Rs-09-161	GCA_000671335.1	Complete Genome	I	India	Ramesh et al., 2014
SL3103	GCA_003515205.1	Complete Genome	I	Korea	Cho et al., 2019
SL2330	GCA_003515225.1	Complete Genome	I	Korea	Cho et al., 2019
T117	GCA_003515245.1	Complete Genome	I	Korea	Cho et al., 2019
T78	GCA_003515285.1	Complete Genome	I	Korea	Cho et al., 2019
T25	GCA_003515305.1	Complete Genome	I	Korea	Cho et al., 2019
SL3755	GCA_003515345.1	Complete Genome	I	Korea	Cho et al., 2019
SL3730	GCA_003515365.1	Complete Genome	I	Korea	Cho et al., 2019
SL2729	GCA_003515405.1	Complete Genome	I	Korea	Cho et al., 2019
T110	GCA_003515465.1	Complete Genome	I	Korea	Cho et al., 2019
T60	GCA_003515545.1	Complete Genome	I	Korea	Cho et al., 2019
T42	GCA_003515565.1	Complete Genome	I	Korea	Cho et al., 2019
SL3882	GCA_003515585.1	Complete Genome	I	Korea	Cho et al., 2019
SL3822	GCA_003515605.1	Complete Genome	I	Korea	Cho et al., 2019
SL3300	GCA_003515625.1	Complete Genome	I	Korea	Cho et al., 2019
HA4-1	GCF_003999725.1	Complete Genome	I	China	Tan et al., 2019
P781	GCA_001644865.1	Draft	I	USA	-
KACC10709	GCA_001708525.1	Complete Genome	I	Korea	-
KACC 10722	GCA_001586135.1	Complete Genome	IIB	Korea	-
CaRs-Mep	GCA_001855495.1	Draft	I	India	-
PSS190	GCA_001870825.1	Draft	I	Taiwan	-
PSS216	GCA_001876975.1	Draft	I	Taiwan	-
RD15	GCA_001854265.1	Draft	-	Taiwan	-

PSS1308	GCA_001870805.1	Draft	-	Taiwan	-
SD54	GCA_000430925.2	Draft	I	China	Shan et al 2013
PSS4	GCA_001876985.1	Draft	I	Taiwan	Shan et al 2013
BBAC-C1	GCA_001920895.1	Draft	I	China	-
FJAT-452	GCA_001920905.1	Draft	I	China	-
CMR15	GCA_000427195.1	Complete Genome	III	Cameroon	Remenant et al., 2010
CFBP3059	GCA_001644855.1	Draft	III	Burkina Faso	-
Bg07	GCA_003256445.1	Draft	I	-	-
Rs-T02	GCA_001484095.1	Draft	I	China	Zou et al., 2016
Cq01	GCA_003256425.1	Draft	I	-	-
Fm03	GCA_003256405.1	Draft	I	-	-
RS 476	GCA_003595305.1	Complete Genome	I	Brazil	Albuquerque et al., 2017
CRMRs218	GCF_003612975.1	Complete Genome	I	Brazil	Albuquerque et al., 2017
YC40M	GCA_001663415.1	Complete Genome	I	China	-
Ralstonia syzygii (*Ralstonia solanacearum* phlylotype IV)					
PSI07 (subsp. *indonesiensis*)	GCA_000283475.1	Complete Genome	IV	Indonesia	Remenant et al., 2010
T51	GCA_003515145.1	Complete Genome	IV	Korea	Cho et al., 2019
T11	GCA_003515165.1	Complete Genome	IV	Korea	Cho et al., 2019
SL3175	GCA_003515185.1	Complete Genome	IV	Korea	Cho et al., 2019
T98	GCA_003515265.1	Complete Genome	IV	Korea	Cho et al., 2019
T12	GCA_003515325.1	Complete Genome	IV	Korea	Cho et al., 2019
SL3022	GCA_003515385.1	Complete Genome	IV	Korea	Cho et al., 2019
SL2312	GCA_003515425.1	Complete Genome	IV	Korea	Cho et al., 2019
SL2064	GCA_003515445.1	Complete Genome	IV	Korea	Cho et al., 2019
T101	GCA_003515485.1	Complete Genome	IV	Korea	Cho et al., 2019
T95	GCA_003515505.1	Complete Genome	IV	Korea	Cho et al., 2019
T82	GCA_003515525.1	Complete Genome	IV	Korea	Cho et al., 2019

A2-HR-MARDI (subsp. *celebesensis*)	GCA_002012345.1	Complete Genome	IV	-	Badrun et al., 2017
R229 (subsp. *celebesensis*)	PRJNA369602	Draft	IV	Malaysia	Remenant et al., 2011
R24 (subsp. *syzygii*)	PRJNA53879	Draft	IV	-	Remenant et al., 2011
Ralstonia pickettii					
12D	GCA_000023425.1	Complete Genome	-	USA	-
12J	GCA_000020205.1	Complete Genome	-	USA	-
FDAARGOS_410	GCA_002393485.1	Complete Genome	-	-	-
5_7_47FAA	GCA_000165085.1	Draft	-	Canada	-
52	GCA_002849525.1	Draft	-	-	-
ATCC 27511	GCA_000743455.1	Draft	-	-	Daligault et al., 2014
H2Cu5	GCA_001699815.1	Draft	-	Portugal	Vaz-Moreira et al., 2016
H2Cu2	GCA_001699795.1	Draft	-	Portugal	Vaz-Moreira et al., 2016
ICMP-8657	GCA_002516395.2	Draft	-	Germany	Paterson and Gross, 2018
NBRC 102503	GCA_001544155.1	Draft	-	-	-
OR214	GCA_000372665.1	Draft	-	-	-
SSH4	GCA_000607165.1	Draft	-	-	-
DTP0602	GCA_000471925.1	Complete Genome	-	-	Ohtsubo et al., 2013
Ralstonia mannitolilytica				-	
SN82F48	GCA_000954135.2	Complete Genome	-	-	-
SN83A39	GCA_001628775.1	Complete Genome	-	-	-
Ralstonia insidiosa					
ATCC 49129	GCA_001663855.1	Complete Genome	-	-	Xu et al., 2016
FC1138	GCA_001653935.1	Complete Genome	-	-	Xu et al., 2016

Table S2. Isolates of *Ralstonia solanacearum* used in this study

Isolated	Year of collection	Place	Sample	Phylotype
UFV 30	2015	Coimbra - MG	Soil	2
UFV 440	2015	Coimbra - MG	Soil	2
UFV 203	2015	Coimbra - MG	Soil	2
UFV 330	2016	Brasília -DF	Soil	2
UFV 336	2016	Brasília -DF	Soil	2
UFV 459	2017	Coimbra - MG	Soil	2
UFV 477	2017	Coimbra - MG	Soil	2

Table S3a. Characterization of Insertion sequences elements found in the chromosome of *Ralstonia* spp. with ISsaga

Strain	ORF Name	% of Similarity Aas	% of AAs Similarity	Identified IS Family	ORF Left End	ORF Right End	ORF Size (bp)
GMI1000	IS_f1ddcc0a_03235	49.48% ISHvo5_aa1	44.65% IS_f1ddcc0a_00905	IS1595 ssgr ISH4	3439165	3438377	789
GMI1000	IS_f1ddcc0a_00250	100% ISRso6_aa1	100% IS_f1ddcc0a_02374	IS21	275259	276302	1044
GMI1000	IS_f1ddcc0a_00251	100% ISRso6_aa2	100% IS_f1ddcc0a_02375	IS21	276299	277144	846
GMI1000	IS_f1ddcc0a_02374	100% ISRso6_aa1	100% IS_f1ddcc0a_00250	IS21	2531677	2532720	1044
GMI1000	IS_f1ddcc0a_02375	100% ISRso6_aa2	100% IS_f1ddcc0a_00251	IS21	2532717	2533562	846
GMI1000	IS_f1ddcc0a_00254	100% ISRso7_aa1	No hit	IS256	282838	284088	1251
GMI1000	IS_f1ddcc0a_00593	100% ISRso8_aa2	100% IS_f1ddcc0a_02306	IS3	623176	622286	891
GMI1000	IS_f1ddcc0a_00594	100% ISRso8_aa1	100% IS_f1ddcc0a_02307	IS3	623466	623173	294
GMI1000	IS_f1ddcc0a_01584	100% ISRso8_aa2	100% IS_f1ddcc0a_02306	IS3	1662654	1661764	891
GMI1000	IS_f1ddcc0a_01585	100% ISRso8_aa1	100% IS_f1ddcc0a_02307	IS3	1662944	1662651	294
GMI1000	IS_f1ddcc0a_02306	100% ISRso8_aa2	100% IS_f1ddcc0a_01584	IS3	2458962	2458072	891
GMI1000	IS_f1ddcc0a_02307	100% ISRso8_aa1	100% IS_f1ddcc0a_01585	IS3	2459252	2458959	294
GMI1000	IS_f1ddcc0a_01468	100% ISRso11_aa2	100% IS_f1ddcc0a_03144	IS3 ssgr IS150	1541024	1540188	837
GMI1000	IS_f1ddcc0a_01469	100% ISRso11_aa1	100% IS_f1ddcc0a_03145	IS3 ssgr IS150	1541554	1541021	534
GMI1000	IS_f1ddcc0a_02304	100% ISRso11_aa2	100% IS_f1ddcc0a_03144	IS3 ssgr IS150	2457510	2456674	837
GMI1000	IS_f1ddcc0a_02305	100% ISRso11_aa1	100% IS_f1ddcc0a_03145	IS3 ssgr IS150	2457935	2457507	429
GMI1000	IS_f1ddcc0a_02451	99.64% ISRso11_aa2	99.64% IS_f1ddcc0a_03144	IS3 ssgr IS150	2614089	2613253	837
GMI1000	IS_f1ddcc0a_02452	100% ISRso11_aa1	100% IS_f1ddcc0a_03145	IS3 ssgr IS150	2614619	2614086	534
GMI1000	IS_f1ddcc0a_03144	100% ISRso11_aa2	100% IS_f1ddcc0a_02304	IS3 ssgr IS150	3327278	3326442	837
GMI1000	IS_f1ddcc0a_03145	100% ISRso11_aa1	100% IS_f1ddcc0a_02452	IS3 ssgr IS150	3327808	3327275	534
GMI1000	IS_f1ddcc0a_01466	100% ISRso10_aa1	100% IS_f1ddcc0a_01873	IS3 ssgr IS2	1538857	1539246	390
GMI1000	IS_f1ddcc0a_01467	100% ISRso10_aa2	100% IS_f1ddcc0a_02372	IS3 ssgr IS2	1539243	1540091	849
GMI1000	IS_f1ddcc0a_01872	100% ISRso10_aa2	100% IS_f1ddcc0a_02372	IS3 ssgr IS2	2002393	2001545	849
GMI1000	IS_f1ddcc0a_01873	100% ISRso10_aa1	100% IS_f1ddcc0a_01466	IS3 ssgr IS2	2002779	2002390	390
GMI1000	IS_f1ddcc0a_02372	100% ISRso10_aa2	100% IS_f1ddcc0a_01872	IS3 ssgr IS2	2531315	2530467	849
GMI1000	IS_f1ddcc0a_02373	100% ISRso10_aa1	100% IS_f1ddcc0a_01873	IS3 ssgr IS2	2531557	2531312	246
GMI1000	IS_f1ddcc0a_02353	89.23% IS222_aa2	57.69% IS_f1ddcc0a_02306	IS3 ssgr IS3	2514893	2514222	672
GMI1000	IS_f1ddcc0a_02354	87.25% IS222_aa1	No hit	IS3 ssgr IS3	2515384	2515076	309
GMI1000	IS_f1ddcc0a_00642	87.67% ISSme1_aa3	80.28% IS_f1ddcc0a_02449	IS3 ssgr IS407	670988	670635	354
GMI1000	IS_f1ddcc0a_00845	100% ISRso14_aa2	100% IS_f1ddcc0a_02448	IS3 ssgr IS407	870517	869969	549
GMI1000	IS_f1ddcc0a_00846	100% ISRso14_aa1	100% IS_f1ddcc0a_02449	IS3 ssgr IS407	871086	870823	264

GMI1000	IS_f1ddcc0a_01470	100% ISRso12_aa1	74.11% IS_f1ddcc0a_02449	IS3 ssgr IS407	1541818	1542084	267
GMI1000	IS_f1ddcc0a_01471	100% ISRso12_aa2	100% IS_f1ddcc0a_02748	IS3 ssgr IS407	1542234	1542938	705
GMI1000	IS_f1ddcc0a_01525	100% ISRso14_aa2	100% IS_f1ddcc0a_02448	IS3 ssgr IS407	1597558	1597010	549
GMI1000	IS_f1ddcc0a_01526	100% ISRso14_aa1	100% IS_f1ddcc0a_02449	IS3 ssgr IS407	1598127	1597864	264
GMI1000	IS_f1ddcc0a_02448	100% ISRso14_aa2	100% IS_f1ddcc0a_01525	IS3 ssgr IS407	2612264	2611716	549
GMI1000	IS_f1ddcc0a_02449	100% ISRso14_aa1	100% IS_f1ddcc0a_01526	IS3 ssgr IS407	2612833	2612570	264
GMI1000	IS_f1ddcc0a_02748	100% ISRso12_aa2	100% IS_f1ddcc0a_01471	IS3 ssgr IS407	2911692	2910988	705
GMI1000	IS_f1ddcc0a_01583	76.34% ISBcen21_aa1	54.54% IS_f1ddcc0a_02307	IS3 ssgr IS51	1661338	1661625	288
GMI1000	IS_f1ddcc0a_01517	100% ISRso13_aa1	100% IS_f1ddcc0a_03290	IS4 ssgr IS4	1590766	1589432	1335
GMI1000	IS_f1ddcc0a_01907	100% ISRso13_aa1	100% IS_f1ddcc0a_03290	IS4 ssgr IS4	2047948	2049282	1335
GMI1000	IS_f1ddcc0a_03225	100% ISRso13_aa1	100% IS_f1ddcc0a_03290	IS4 ssgr IS4	3427557	3428891	1335
GMI1000	IS_f1ddcc0a_03290	100% ISRso13_aa1	100% IS_f1ddcc0a_03225	IS4 ssgr IS4	3500148	3498814	1335
GMI1000	IS_f1ddcc0a_00210	99.27% ISRso1_aa1	100% IS_f1ddcc0a_03401	IS5	231839	232738	900
GMI1000	IS_f1ddcc0a_01023	98.00% ISRso1_aa1	98.80% IS_f1ddcc0a_03401	IS5	1060272	1059517	756
GMI1000	IS_f1ddcc0a_03249	97.44% ISRso1_aa1	97.44% IS_f1ddcc0a_03401	IS5	3460622	3461446	825
GMI1000	IS_f1ddcc0a_03270	99.10% ISRso1_aa1	99.10% IS_f1ddcc0a_03401	IS5	3483498	3484175	678
GMI1000	IS_f1ddcc0a_03401	99.27% ISRso1_aa1	100% IS_f1ddcc0a_00210	IS5	3612864	3612040	825
GMI1000	IS_f1ddcc0a_00909	76.56% ISCARN14_aa1	51.10% IS_f1ddcc0a_00210	IS5 ssgr IS1031	940708	93953	1179
GMI1000	IS_f1ddcc0a_01711	100% IS1421_aa1	No hit	IS5 ssgr IS427	1793014	1792610	405
GMI1000	IS_f1ddcc0a_00859	100% ISRso9_aa1	100% IS_f1ddcc0a_03471	IS5 ssgr IS5	883334	884656	1323
GMI1000	IS_f1ddcc0a_01749	100% ISRso18_aa1	No hit	IS5 ssgr IS5	1821238	1820273	966
GMI1000	IS_f1ddcc0a_03179	100% ISRso9_aa1	100% IS_f1ddcc0a_03471	IS5 ssgr IS5	3367094	3365772	1323
GMI1000	IS_f1ddcc0a_03201	100% ISRso9_aa1	100% IS_f1ddcc0a_03471	IS5 ssgr IS5	3396682	3395360	1323
GMI1000	IS_f1ddcc0a_03471	100% ISRso9_aa1	100% IS_f1ddcc0a_03201	IS5 ssgr IS5	3697025	3695703	1323
GMI1000	IS_f1ddcc0a_00104	100% ISRso5_aa1	100% IS_f1ddcc0a_03444	IS630	117992	119083	1092
GMI1000	IS_f1ddcc0a_00110	99.72% ISRso5_aa1	99.72% IS_f1ddcc0a_03444	IS630	127903	128994	1092
GMI1000	IS_f1ddcc0a_00657	100% ISRso5_aa1	100% IS_f1ddcc0a_03444	IS630	683432	684523	1092
GMI1000	IS_f1ddcc0a_02214	100% ISRso5_aa1	100% IS_f1ddcc0a_03444	IS630	2360185	2361276	1092
GMI1000	IS_f1ddcc0a_03444	100% ISRso5_aa1	100% IS_f1ddcc0a_02214	IS630	3660475	3659384	1092
GMI1000	IS_f1ddcc0a_00054	43.33% ISMno24_aa2	52.12% IS_f1ddcc0a_02587	IS91	58345	57356	990
GMI1000	IS_f1ddcc0a_01589	40.16% ISShvi3_aa1	52.96% IS_f1ddcc0a_01696	IS91	1670803	1669739	1065
GMI1000	IS_f1ddcc0a_02587	48.21% ISTha3_aa2	51.50% IS_f1ddcc0a_00054	IS91	2752033	2752959	927
GMI1000	IS_f1ddcc0a_02616	100% ISRso15_aa1	No hit	ISL3	2780153	2781373	1221
GMI1000	IS_f1ddcc0a_02663	77.94% ISSm4_aa2	No hit	ISL3	2824603	2823536	1068
GMI1000	IS_f1ddcc0a_03447	38.97% ISKpn25_aa1	No hit	ISL3	3665746	3664301	1446

GMI1000	IS_f1ddcc0a_01886	64.16% ISKpn21_aa1	92.5% IS_f1ddcc0a_01887	ISNCY ssgr IS1202	2023647	2023255	393
GMI1000	IS_f1ddcc0a_01887	71.39% ISKpn21_aa1	92.5% IS_f1ddcc0a_01886	ISNCY ssgr IS1202	2025276	2023801	1476
GMI1000	IS_f1ddcc0a_00907	54.43% ISMpo10_aa3	72.74% IS_f1ddcc0a_03295	Tn3	937582	938964	1383
GMI1000	IS_f1ddcc0a_03228	61.81% ISMpo10_aa3	97.40% IS_f1ddcc0a_03295	Tn3	3430395	3431783	1389
GMI1000	IS_f1ddcc0a_03295	55.34% ISMpo10_aa3	97.40% IS_f1ddcc0a_03228	Tn3	3502812	3504200	1389
Po82	RSPO_c00521	54.35% ISHpa1_aa1	100% RSPO_c02362	IS1595 ssgr IS1016	540772	541551	780
Po82	RSPO_c02362	54.35% ISHpa1_aa1	100% RSPO_c00521	IS1595 ssgr IS1016	2472550	2473329	780
Po82	RSPO_c00288	51.40% ISPto1_aa1	No hit	IS1595 ssgr ISPna2	300425	301885	1461
Po82	RSPO_c00361	98.22% ISRso19_aa1	100% RSPO_c02740	IS21	380888	381913	1026
Po82	RSPO_c00362	99.23% ISRso19_aa2	100% RSPO_c02739	IS21	38191	382698	789
Po82	RSPO_c01120	98.22% ISRso19_aa1	100% RSPO_c02740	IS21	1171911	1172936	1026
Po82	RSPO_c01121	99.23% ISRso19_aa2	100% RSPO_c02739	IS21	1172933	1173721	789
Po82	RSPO_c01546	99.23% ISRso19_aa2	100% RSPO_c02739	IS21	1611633	1610845	789
Po82	RSPO_c01547	98.22% ISRso19_aa1	100% RSPO_c02740	IS21	1612655	1611630	1026
Po82	RSPO_c01626	98.22% ISRso19_aa1	100% RSPO_c02740	IS21	1695590	1696615	1026
Po82	RSPO_c01627	100% ISRso19_aa2	99.61% RSPO_c02531	IS21	1696612	1697400	789
Po82	RSPO_c02531	99.61% ISRso19_aa2	99.61% RSPO_c02739	IS21	2647444	2646656	789
Po82	RSPO_c02532	98.22% ISRso19_aa1	100% RSPO_c02740	IS21	2648466	2647441	1026
Po82	RSPO_c02739	99.23% ISRso19_aa2	100% RSPO_c01546	IS21	2855271	2854483	789
Po82	RSPO_c02740	98.22% ISRso19_aa1	100% RSPO_c02532	IS21	2856293	2855268	1026
Po82	RSPO_c00290	94.57% ISRso10_aa2	100% RSPO_c02817	IS3 ssgr IS2	304322	303435	888
Po82	RSPO_c00291	100% ISRso10_aa1	100% RSPO_c02818	IS3 ssgr IS2	304669	30428	390
Po82	RSPO_c01538	94.57% ISRso10_aa2	100% RSPO_c02817	IS3 ssgr IS2	1606012	1605125	888
Po82	RSPO_c01539	100% ISRso10_aa1	100% RSPO_c02818	IS3 ssgr IS2	1606335	1605970	366
Po82	RSPO_c01541	100% ISRso10_aa1	100% RSPO_c02818	IS3 ssgr IS2	1606697	1607062	366
Po82	RSPO_c01542	94.57% ISRso10_aa2	100% RSPO_c02817	IS3 ssgr IS2	1607020	1607907	888
Po82	RSPO_c02267	79.03% ISRso10_aa2	76.22% RSPO_c02817	IS3 ssgr IS2	2375945	2376586	642
Po82	RSPO_c02529	100% ISRso10_aa1	100% RSPO_c02818	IS3 ssgr IS2	2643607	2643996	390
Po82	RSPO_c02530	94.57% ISRso10_aa2	100% RSPO_c02817	IS3 ssgr IS2	2643954	2644841	888
Po82	RSPO_c02805	100% ISRso10_aa1	100% RSPO_c02818	IS3 ssgr IS2	2922102	2922491	390
Po82	RSPO_c02806	94.57% ISRso10_aa2	100% RSPO_c02817	IS3 ssgr IS2	2922449	2923336	888
Po82	RSPO_c02817	94.57% ISRso10_aa2	100% RSPO_c02806	IS3 ssgr IS2	2938575	2937688	888
Po82	RSPO_c02818	100% ISRso10_aa1	100% RSPO_c02805	IS3 ssgr IS2	2938922	2938533	390
Po82	RSPO_c01524	76.09% ISDet2_aa2	100% RSPO_c02809	IS3 ssgr IS407	1592457	1591633	825

Po82	RSPO_c01523	90.58% ISAtu5_aa1	100% RSPO_c02808	IS3 ssgr IS407	1592756	1592496	261
Po82	RSPO_c02732	90.58% ISAtu5_aa1	100% RSPO_c02808	IS3 ssgr IS407	2850322	2850582	261
Po82	RSPO_c02733	75.69% ISDet2_aa2	99.27% RSPO_c02809	IS3 ssgr IS407	2850621	2851445	825
Po82	RSPO_c02808	90.58% ISAtu5_aa1	100% RSPO_c01523	IS3 ssgr IS407	2924198	2924458	261
Po82	RSPO_c02809	76.09% ISDet2_aa2	100% RSPO_c01524	IS3 ssgr IS407	2924497	2925321	825
Po82	RSPO_c01278	96.26% IS401_aa1	100% RSPO_c02751	IS3 ssgr IS51	1356718	1357041	324
Po82	RSPO_c01279	96.71% IS401_aa2	100% RSPO_c02750	IS3 ssgr IS51	1357038	1357952	915
Po82	RSPO_c01537	96.26% IS401_aa1	100% RSPO_c02751	IS3 ssgr IS51	1604727	1605050	324
Po82	RSPO_c01540	94.73% IS401_aa2	100% RSPO_c02750	IS3 ssgr IS51	1606632	1606414	219
Po82	RSPO_c01543	96.95% IS401_aa2	100% RSPO_c01279	IS3 ssgr IS51	1607951	1608643	693
Po82	RSPO_c01624	95.32% IS401_aa1	99.06% RSPO_c02751	IS3 ssgr IS51	1694655	1694978	324
Po82	RSPO_c01625	96.72% IS401_aa2	99.45% RSPO_c02750	IS3 ssgr IS51	1694975	1695535	561
Po82	RSPO_c01628	95.90% IS401_aa2	100% RSPO_c01543	IS3 ssgr IS51	1697483	1697851	369
Po82	RSPO_c02750	96.71% IS401_aa2	100% RSPO_c01279	IS3 ssgr IS51	2867224	2866310	915
Po82	RSPO_c02751	96.26% IS401_aa1	100% RSPO_c01537	IS3 ssgr IS51	2867544	2867221	324
Po82	RSPO_c00907	47.98% ISMno23_aa1	51.21% RSPO_c03308	IS91	94845	947515	936
Po82	RSPO_c03308	48.25% ISWz1_aa1	51.21% RSPO_c00907	IS91	3434061	3435050	990
Po82	RSPO_c00042	41.51% ISSm4_aa1	No hit	ISL3	54684	56129	1446
Po82	RSPO_c01722	51.87% ISSm4_aa2	No hit	ISL3	1793320	1792442	879
Po82	RSPO_c00861	0% newcandidate	not_found	New_Family	907716	907579	138
Po82	RSPO_c01167	0% newcandidate	not_found	New_Family	1231935	1233212	1278
Po82	RSPO_c02883	0% newcandidate	not_found	New_Family	3013646	3014917	1272
Po82	RSPO_c00217	56.52% ISMpo10_aa3	71.83% RSPO_c02464	Tn3	231063	229684	138
Po82	RSPO_c00263	63.51% ISPsy30_aa1	No hit	Tn3	264394	263519	876
Po82	RSPO_c00738	53.33% TnShfr1_aa1	No hit	Tn3	771686	772912	1227
Po82	RSPO_c02464	53.04% ISMpo10_aa3	71.83% RSPO_c00217	Tn3	2578187	2576808	138
Po82	RSPO_c02813	68.47% ISYps3_aa2	48.71% RSPO_c00217	Tn3	2933810	2934427	618
UY031	RSUY_29420	96.54% ISBma3_aa1	No hit	IS110	3122091	3123047	957
UY031	RSUY_04240	98.36% ISRso10_aa1	100% RSUY_30360	IS3 ssgr IS2	489111	489503	393
UY031	RSUY_04250	97.70% ISRso10_aa2	100% RSUY_30350	IS3 ssgr IS2	489824	490348	525
UY031	RSUY_11740	98.36% ISRso10_aa1	100% RSUY_30360	IS3 ssgr IS2	1260631	1261023	393
UY031	RSUY_11750	97.70% ISRso10_aa2	100% RSUY_30350	IS3 ssgr IS2	1261344	1261868	525
UY031	RSUY_15190	82.95% ISRso10_aa2	79.54% RSUY_30350	IS3 ssgr IS2	1627376	1627038	339
UY031	RSUY_18780	98.36% ISRso10_aa1	100% RSUY_30360	IS3 ssgr IS2	2012988	2013380	393

UY031	RSUY_18790	97.70% ISRso10_aa2	100% RSUY_30350	IS3 ssgr IS2	2013701	2014225	525
UY031	RSUY_21360	97.70% ISRso10_aa2	100% RSUY_30350	IS3 ssgr IS2	2300802	2300278	525
UY031	RSUY_21370	98.36% ISRso10_aa1	100% RSUY_30360	IS3 ssgr IS2	2301515	2301123	393
UY031	RSUY_26390	98.36% ISRso10_aa1	100% RSUY_30360	IS3 ssgr IS2	2816389	2816781	393
UY031	RSUY_26400	97.70% ISRso10_aa2	100% RSUY_30350	IS3 ssgr IS2	2817102	2817626	525
UY031	RSUY_30350	97.70% ISRso10_aa2	100% RSUY_26400	IS3 ssgr IS2	3221043	3220519	525
UY031	RSUY_30360	98.36% ISRso10_aa1	100% RSUY_26390	IS3 ssgr IS2	3221756	3221364	393
UY031	RSUY_21320	100% ISRso20_aa2	100% RSUY_21430	IS3 ssgr IS3	2297602	2296760	843
UY031	RSUY_21330	100% ISRso20_aa1	100% RSUY_21420	IS3 ssgr IS3	2297943	2297647	297
UY031	RSUY_21420	100% ISRso20_aa1	100% RSUY_21330	IS3 ssgr IS3	2305957	2306253	297
UY031	RSUY_21430	100% ISRso20_aa2	100% RSUY_21320	IS3 ssgr IS3	2306298	2307140	843
UY031	RSUY_10520	38.55% ISGur11_aa1	No hit	IS481	1140550	1142232	1683
UY031	RSUY_00690	88.37% ISAzo23_aa1	100% RSUY_30650	IS5 ssgr IS1031	72573	71767	807
UY031	RSUY_07720	88.37% ISAzo23_aa1	100% RSUY_30650	IS5 ssgr IS1031	856004	855198	807
UY031	RSUY_21820	88.37% ISAzo23_aa1	100% RSUY_30650	IS5 ssgr IS1031	2344874	2345680	807
UY031	RSUY_30650	88.37% ISAzo23_aa1	100% RSUY_21820	IS5 ssgr IS1031	3253867	3254673	807
UY031	RSUY_00500	100% IS1021_aa1	100% RSUY_32060	IS5 ssgr IS5	50636	51622	987
UY031	RSUY_00560	100% IS1021_aa1	100% RSUY_32060	IS5 ssgr IS5	58608	57622	987
UY031	RSUY_00630	100% IS1021_aa1	100% RSUY_32060	IS5 ssgr IS5	65759	64773	987
UY031	RSUY_00710	100% IS1021_aa1	100% RSUY_32060	IS5 ssgr IS5	76002	75016	987
UY031	RSUY_02480	100% IS1021_aa1	100% RSUY_32060	IS5 ssgr IS5	269562	270548	987
UY031	RSUY_02720	100% IS1021_aa1	100% RSUY_32060	IS5 ssgr IS5	306964	305978	987
UY031	RSUY_02740	100% IS1021_aa1	100% RSUY_32060	IS5 ssgr IS5	308712	309698	987
UY031	RSUY_02760	100% IS1021_aa1	100% RSUY_32060	IS5 ssgr IS5	320244	32123	987
UY031	RSUY_02890	100% IS1021_aa1	100% RSUY_32060	IS5 ssgr IS5	344825	343839	987
UY031	RSUY_03650	100% IS1021_aa1	100% RSUY_32060	IS5 ssgr IS5	428821	429807	987
UY031	RSUY_08610	100% IS1021_aa1	100% RSUY_32060	IS5 ssgr IS5	950134	949148	987
UY031	RSUY_09620	100% IS1021_aa1	100% RSUY_32060	IS5 ssgr IS5	1051555	1052541	987
UY031	RSUY_10820	100% IS1021_aa1	100% RSUY_32060	IS5 ssgr IS5	1162863	1163849	987
UY031	RSUY_11190	100% IS1021_aa1	100% RSUY_32060	IS5 ssgr IS5	1204835	1203849	987
UY031	RSUY_11530	100% IS1021_aa1	100% RSUY_32060	IS5 ssgr IS5	1238767	1237781	987
UY031	RSUY_11670	100% IS1021_aa1	100% RSUY_32060	IS5 ssgr IS5	1254027	1253041	987
UY031	RSUY_11720	100% IS1021_aa1	100% RSUY_32060	IS5 ssgr IS5	1257120	1258106	987
UY031	RSUY_13380	100% IS1021_aa1	100% RSUY_32060	IS5 ssgr IS5	1439072	1438086	987
UY031	RSUY_19460	100% IS1021_aa1	100% RSUY_32060	IS5 ssgr IS5	2088456	2087470	987

UY031	RSUY_19480	100% IS1021_aa1	100% RSUY_32060	IS5 ssgr IS5	2089167	2090153	987
UY031	RSUY_20020	100% IS1021_aa1	100% RSUY_32060	IS5 ssgr IS5	2150144	2149158	987
UY031	RSUY_20050	100% IS1021_aa1	100% RSUY_32060	IS5 ssgr IS5	2152354	2151368	987
UY031	RSUY_21380	100% IS1021_aa1	100% RSUY_32060	IS5 ssgr IS5	2301689	2302675	987
UY031	RSUY_21810	100% IS1021_aa1	100% RSUY_32060	IS5 ssgr IS5	2344750	2343764	987
UY031	RSUY_22220	100% IS1021_aa1	100% RSUY_32060	IS5 ssgr IS5	2389012	2389998	987
UY031	RSUY_22270	100% IS1021_aa1	100% RSUY_32060	IS5 ssgr IS5	2393813	2392827	987
UY031	RSUY_22570	100% IS1021_aa1	100% RSUY_32060	IS5 ssgr IS5	2428298	2427312	987
UY031	RSUY_23430	100% IS1021_aa1	100% RSUY_32060	IS5 ssgr IS5	2514809	2515795	987
UY031	RSUY_24040	100% IS1021_aa1	100% RSUY_32060	IS5 ssgr IS5	2574317	2575303	987
UY031	RSUY_24090	100% IS1021_aa1	100% RSUY_32060	IS5 ssgr IS5	2581236	2580250	987
UY031	RSUY_25310	100% IS1021_aa1	100% RSUY_32060	IS5 ssgr IS5	2707863	2708849	987
UY031	RSUY_25530	100% IS1021_aa1	100% RSUY_32060	IS5 ssgr IS5	2733067	2734053	987
UY031	RSUY_26190	100% IS1021_aa1	100% RSUY_32060	IS5 ssgr IS5	2793864	2794850	987
UY031	RSUY_26290	100% IS1021_aa1	100% RSUY_32060	IS5 ssgr IS5	2805878	2806864	987
UY031	RSUY_26320	100% IS1021_aa1	100% RSUY_32060	IS5 ssgr IS5	2810917	2809931	987
UY031	RSUY_30050	100% IS1021_aa1	100% RSUY_32060	IS5 ssgr IS5	3187099	3186113	987
UY031	RSUY_30640	100% IS1021_aa1	100% RSUY_32060	IS5 ssgr IS5	3252699	3253685	987
UY031	RSUY_32060	100% IS1021_aa1	100% RSUY_30640	IS5 ssgr IS5	3406083	3405097	987
UY031	RSUY_26130	48.79% ISMno23_aa1	51.35% RSUY_31920	IS91	2789767	2790693	927
UY031	RSUY_31920	48.75% ISWz1_aa1	52.26% RSUY_26130	IS91	3389774	3390763	990
UY031	RSUY_00920	40.40% ISSm4_aa1	No hit	ISL3	102029	103474	1446
UY031	RSUY_10150	75.88% ISSm4_aa2	54.74% RSUY_20670	ISL3	1107505	1106465	1041
UY031	RSUY_20670	49.68% ISSm4_aa2	54.74% RSUY_10150	ISL3	2224129	2225190	1062
UY031	RSUY_19970	68.37% ISKpn21_aa1	No hit	ISNCY ssgr IS1202	2145825	2144959	867
UY031	RSUY_07040	53.33% TnShfr1_aa1	No hit	Tn3	77965	780876	1227
UY031	RSUY_11390	64.08% ISPa38_aa1	46.44% RSUY_21400	Tn3	1228556	1229146	591
UY031	RSUY_21400	52.63% ISSba14_aa1	46.44% RSUY_11390	Tn3	2303976	2304557	582

UW163	IS_dbbfd929_00933	54.35% ISHpa1_aa1	100% IS_dbbfd929_02372	IS1595 ssgr IS1016	98028	981059	780
UW163	IS_dbbfd929_01353	54.35% ISHpa1_aa1	100% IS_dbbfd929_02372	IS1595 ssgr IS1016	1421581	1422360	780
UW163	IS_dbbfd929_02372	54.35% ISHpa1_aa1	100% IS_dbbfd929_01353	IS1595 ssgr IS1016	2512266	2511487	780
UW163	IS_dbbfd929_01131	51.40% ISPto1_aa1	No hit	IS1595 ssgr ISPna2	1183551	1184984	1434
UW163	IS_dbbfd929_00237	99.61% ISRso19_aa2	100% IS_dbbfd929_01410	IS21	235881	235093	789
UW163	IS_dbbfd929_00238	98.18% ISRso19_aa1	100% IS_dbbfd929_03165	IS21	236873	235878	996

UW163	IS_dbbfd929_00308	97.88% ISRso19_aa1	99.69% IS_dbbfd929_03165	IS21	309932	310927	996
UW163	IS_dbbfd929_00309	99.23% ISRso19_aa2	100% IS_dbbfd929_03166	IS21	310924	311712	789
UW163	IS_dbbfd929_00321	98.18% ISRso19_aa1	100% IS_dbbfd929_03165	IS21	321767	322762	996
UW163	IS_dbbfd929_00322	99.23% ISRso19_aa2	100% IS_dbbfd929_03166	IS21	322759	323547	789
UW163	IS_dbbfd929_00872	97.88% ISRso19_aa1	100% IS_dbbfd929_03089	IS21	906479	907474	996
UW163	IS_dbbfd929_00873	99.61% ISRso19_aa2	100% IS_dbbfd929_03088	IS21	907471	908259	789
UW163	IS_dbbfd929_01192	98.18% ISRso19_aa1	100% IS_dbbfd929_03165	IS21	1261738	1262733	996
UW163	IS_dbbfd929_01193	99.23% ISRso19_aa2	100% IS_dbbfd929_03166	IS21	1262730	1263518	789
UW163	IS_dbbfd929_01410	99.61% ISRso19_aa2	100% IS_dbbfd929_00237	IS21	1480568	1479780	789
UW163	IS_dbbfd929_01411	98.18% ISRso19_aa1	100% IS_dbbfd929_03165	IS21	1481560	1480565	996
UW163	IS_dbbfd929_01938	98.18% ISRso19_aa1	100% IS_dbbfd929_03165	IS21	2054612	2055607	996
UW163	IS_dbbfd929_01939	99.23% ISRso19_aa2	100% IS_dbbfd929_03166	IS21	2055604	2056392	789
UW163	IS_dbbfd929_03088	99.61% ISRso19_aa2	100% IS_dbbfd929_00873	IS21	3284222	3283434	789
UW163	IS_dbbfd929_03089	97.88% ISRso19_aa1	100% IS_dbbfd929_00872	IS21	3285214	3284219	996
UW163	IS_dbbfd929_03100	98.18% ISRso19_aa1	100% IS_dbbfd929_03165	IS21	3297546	3298541	996
UW163	IS_dbbfd929_03101	99.23% ISRso19_aa2	100% IS_dbbfd929_03166	IS21	3298538	3299326	789
UW163	IS_dbbfd929_03165	98.18% ISRso19_aa1	100% IS_dbbfd929_03100	IS21	3371514	3372509	996
UW163	IS_dbbfd929_03166	99.23% ISRso19_aa2	100% IS_dbbfd929_01939	IS21	3372506	3373294	789
UW163	IS_dbbfd929_00038	100% ISRso10_aa1	100% IS_dbbfd929_03162	IS3 ssgr IS2	28839	29228	390
UW163	IS_dbbfd929_00039	96% ISRso10_aa2	100% IS_dbbfd929_03161	IS3 ssgr IS2	29471	30073	603
UW163	IS_dbbfd929_00303	100% ISRso10_aa1	100% IS_dbbfd929_03162	IS3 ssgr IS2	30267	303059	390
UW163	IS_dbbfd929_00304	96% ISRso10_aa2	100% IS_dbbfd929_03161	IS3 ssgr IS2	303302	303904	603
UW163	IS_dbbfd929_00317	96% ISRso10_aa2	100% IS_dbbfd929_03161	IS3 ssgr IS2	319625	319023	603
UW163	IS_dbbfd929_00318	100% ISRso10_aa1	100% IS_dbbfd929_03162	IS3 ssgr IS2	320257	319868	390
UW163	IS_dbbfd929_02342	96% ISRso10_aa2	100% IS_dbbfd929_03161	IS3 ssgr IS2	2487949	2487347	603
UW163	IS_dbbfd929_02343	100% ISRso10_aa1	100% IS_dbbfd929_03162	IS3 ssgr IS2	2488581	2488192	390
UW163	IS_dbbfd929_02463	80.86% ISRso10_aa2	77.39% IS_dbbfd929_03161	IS3 ssgr IS2	2608657	2608229	429
UW163	IS_dbbfd929_03161	96% ISRso10_aa2	100% IS_dbbfd929_02342	IS3 ssgr IS2	3369482	3368880	603
UW163	IS_dbbfd929_03162	100% ISRso10_aa1	100% IS_dbbfd929_02343	IS3 ssgr IS2	3370114	3369725	390
UW163	IS_dbbfd929_00231	90.58% ISAtu5_aa1	No hit	IS3 ssgr IS407	230939	231199	261
UW163	IS_dbbfd929_00232	75.69% ISDet2_aa2	No hit	IS3 ssgr IS407	231238	232062	825
UW163	IS_dbbfd929_00247	96.71% IS401_aa2	100% IS_dbbfd929_02350	IS3 ssgr IS51	247834	24692	915
UW163	IS_dbbfd929_00248	96.26% IS401_aa1	100% IS_dbbfd929_02339	IS3 ssgr IS51	248154	247831	324
UW163	IS_dbbfd929_02090	96.26% IS401_aa1	100% IS_dbbfd929_02339	IS3 ssgr IS51	2239387	2239710	324
UW163	IS_dbbfd929_02091	96.71% IS401_aa2	100% IS_dbbfd929_02350	IS3 ssgr IS51	2239707	2240621	915

UW163	IS_dbbfd929_02339	96.26% IS401_aa1	100% IS_dbbfd929_02090	IS3 ssgr IS51	2485361	2485684	324
UW163	IS_dbbfd929_02340	96.71% IS401_aa2	100% IS_dbbfd929_02350	IS3 ssgr IS51	2485681	2486595	915
UW163	IS_dbbfd929_02350	96.71% IS401_aa2	100% IS_dbbfd929_02340	IS3 ssgr IS51	2494036	2493122	915
UW163	IS_dbbfd929_02351	95.55% IS401_aa1	100% IS_dbbfd929_02339	IS3 ssgr IS51	2494305	2494033	273
UW163	IS_dbbfd929_03087	97.27% IS401_aa2	100% IS_dbbfd929_02350	IS3 ssgr IS51	3283315	3282983	333
UW163	IS_dbbfd929_03090	96.72% IS401_aa2	99.45% IS_dbbfd929_02350	IS3 ssgr IS51	3285859	3285299	561
UW163	IS_dbbfd929_03091	96.26% IS401_aa1	100% IS_dbbfd929_02339	IS3 ssgr IS51	3286179	3285856	324
UW163	IS_dbbfd929_00794	48.25% ISWz1_aa1	51.21% IS_dbbfd929_01725	IS91	817293	818282	990
UW163	IS_dbbfd929_01725	47.98% ISMno23_aa1	51.21% IS_dbbfd929_00794	IS91	1831214	1830279	936
UW163	IS_dbbfd929_03168	48.05% ISMno24_aa2	44.97% IS_dbbfd929_01725	IS91	3374623	3374030	594
UW163	IS_dbbfd929_00895	41.51% ISSm4_aa1	45.62% IS_dbbfd929_01056	ISL3	93739	938835	1446
UW163	IS_dbbfd929_02994	51.87% ISSm4_aa2	No hit	ISL3	3187516	3188394	879
UW163	IS_dbbfd929_00313	68.47% ISYps3_aa2	48.71% IS_dbbfd929_01059	Tn3	315186	315752	567
UW163	IS_dbbfd929_01059	61.81% ISMpo10_aa3	72.35% IS_dbbfd929_03265	Tn3	1113100	1111712	1389
UW163	IS_dbbfd929_01566	53.33% TnShfr1_aa1	No hit	Tn3	1654497	1655723	1227
UW163	IS_dbbfd929_03265	53.04% ISMpo10_aa3	72.35% IS_dbbfd929_01059	Tn3	3480885	3479506	138

IBSBF1503	IS_4388f2c8_01410	54.35% ISHpa1_aa1	100% IS_4388f2c8_02881	IS1595 ssgr IS1016	1549866	1549087	780
IBSBF1503	IS_4388f2c8_02881	54.35% ISHpa1_aa1	100% IS_4388f2c8_01410	IS1595 ssgr IS1016	3106711	3105932	780
IBSBF1503	IS_4388f2c8_01628	49.43% ISNe7_aa1	No hit	IS1595 ssgr ISPna2	1787941	1786508	1434
IBSBF1503	IS_4388f2c8_00393	100% ISRso19_aa2	99.23% IS_4388f2c8_02515	IS21	425798	42501	789
IBSBF1503	IS_4388f2c8_00394	97.88% ISRso19_aa1	99.69% IS_4388f2c8_02514	IS21	42679	425795	996
IBSBF1503	IS_4388f2c8_00468	98.12% ISRso19_aa1	100% IS_4388f2c8_02514	IS21	511128	512534	1407
IBSBF1503	IS_4388f2c8_00838	99.23% ISRso19_aa2	99.23% IS_4388f2c8_02515	IS21	921279	920491	789
IBSBF1503	IS_4388f2c8_00839	98.18% ISRso19_aa1	100% IS_4388f2c8_02514	IS21	922271	921276	996
IBSBF1503	IS_4388f2c8_01568	99.23% ISRso19_aa2	100% IS_4388f2c8_02515	IS21	1708714	1707926	789
IBSBF1503	IS_4388f2c8_01569	97.88% ISRso19_aa1	99.69% IS_4388f2c8_02514	IS21	1709706	1708711	996
IBSBF1503	IS_4388f2c8_02514	98.18% ISRso19_aa1	100% IS_4388f2c8_00839	IS21	2729877	2730872	996
IBSBF1503	IS_4388f2c8_02515	99.23% ISRso19_aa2	100% IS_4388f2c8_01568	IS21	2730869	2731657	789
IBSBF1503	IS_4388f2c8_00425	96% ISRso10_aa2	100% IS_4388f2c8_02716	IS3 ssgr IS2	466318	465716	603
IBSBF1503	IS_4388f2c8_00426	100% ISRso10_aa1	100% IS_4388f2c8_02717	IS3 ssgr IS2	46695	466561	390
IBSBF1503	IS_4388f2c8_02455	96% ISRso10_aa2	100% IS_4388f2c8_02716	IS3 ssgr IS2	2671696	2671094	603
IBSBF1503	IS_4388f2c8_02456	100% ISRso10_aa1	100% IS_4388f2c8_02717	IS3 ssgr IS2	2672328	2671939	390
IBSBF1503	IS_4388f2c8_02716	96% ISRso10_aa2	100% IS_4388f2c8_02455	IS3 ssgr IS2	2940279	2939677	603
IBSBF1503	IS_4388f2c8_02717	100% ISRso10_aa1	100% IS_4388f2c8_02456	IS3 ssgr IS2	2940911	2940522	390

IBSBF1503	IS_4388f2c8_02972	80.86% ISRso10_aa2	77.39% IS_4388f2c8_02716	IS3 ssgr IS2	3203102	3202674	429
IBSBF1503	IS_4388f2c8_02418	90.58% ISAtu5_aa1	100% IS_4388f2c8_02714	IS3 ssgr IS407	2633812	2634072	261
IBSBF1503	IS_4388f2c8_02419	76.09% ISDet2_aa2	100% IS_4388f2c8_02715	IS3 ssgr IS407	2634111	2634935	825
IBSBF1503	IS_4388f2c8_02520	75.69% ISDet2_aa2	99.27% IS_4388f2c8_02715	IS3 ssgr IS407	2735519	2734695	825
IBSBF1503	IS_4388f2c8_02521	90.58% ISAtu5_aa1	98.83% IS_4388f2c8_02714	IS3 ssgr IS407	2735818	2735558	261
IBSBF1503	IS_4388f2c8_02714	90.58% ISAtu5_aa1	100% IS_4388f2c8_02418	IS3 ssgr IS407	2938385	2938645	261
IBSBF1503	IS_4388f2c8_02715	76.09% ISDet2_aa2	100% IS_4388f2c8_02419	IS3 ssgr IS407	2938684	2939508	825
IBSBF1503	IS_4388f2c8_00392	97.27% IS401_aa2	100% IS_4388f2c8_00687	IS3 ssgr IS51	424891	424559	333
IBSBF1503	IS_4388f2c8_00395	96.72% IS401_aa2	99.45% IS_4388f2c8_00687	IS3 ssgr IS51	427435	426875	561
IBSBF1503	IS_4388f2c8_00396	94.39% IS401_aa1	98.13% IS_4388f2c8_00688	IS3 ssgr IS51	427755	427432	324
IBSBF1503	IS_4388f2c8_00687	96.71% IS401_aa2	99.45% IS_4388f2c8_00395	IS3 ssgr IS51	737165	736251	915
IBSBF1503	IS_4388f2c8_00688	96.26% IS401_aa1	98.13% IS_4388f2c8_00396	IS3 ssgr IS51	737485	737162	324
IBSBF1503	IS_4388f2c8_01041	47.98% ISMno23_aa1	51.21% IS_4388f2c8_01963	IS91	1142150	1143085	936
IBSBF1503	IS_4388f2c8_01963	48.25% ISWz1_aa1	51.21% IS_4388f2c8_01041	IS91	2154066	2153077	990
IBSBF1503	IS_4388f2c8_00298	51.87% ISSm4_aa2	No hit	ISL3	329092	32997	879
IBSBF1503	IS_4388f2c8_01860	41.51% ISSm4_aa1	No hit	ISL3	2030775	2029330	1446
IBSBF1503	IS_4388f2c8_01199	53.33% TnShfr1_aa1	No hit	Tn3	1318907	1317681	1227
IBSBF1503	IS_4388f2c8_01650	63.51% ISPsy30_aa1	No hit	Tn3	1820800	1821675	876
IBSBF1503	IS_4388f2c8_01696	61.81% ISMpo10_aa3	72.59% IS_4388f2c8_02779	Tn3	1854205	1855593	1389
IBSBF1503	IS_4388f2c8_02447	70.54% ISYps3_aa2	65.89% IS_4388f2c8_02450	Tn3	2664086	2664637	552
IBSBF1503	IS_4388f2c8_02450	84.57% ISSba14_aa1	65.89% IS_4388f2c8_02447	Tn3	2667110	2667709	600
IBSBF1503	IS_4388f2c8_02779	53.04% ISMpo10_aa3	72.59% IS_4388f2c8_01696	Tn3	3001074	3002453	138
RS488	CP021652_02918	96.54% ISBma3_aa1	No hit	IS110	3122089	3123045	957
RS488	CP021652_00417	98.34% ISRso10_aa1	100% CP021652_03012	IS3 ssgr IS2	489133	489498	366
RS488	CP021652_00418	97.70% ISRso10_aa2	100% CP021652_03011	IS3 ssgr IS2	489819	490343	525
RS488	CP021652_01163	98.34% ISRso10_aa1	100% CP021652_03012	IS3 ssgr IS2	1260650	1261015	366
RS488	CP021652_01164	97.70% ISRso10_aa2	100% CP021652_03011	IS3 ssgr IS2	1261336	1261860	525
RS488	CP021652_01503	82.95% ISRso10_aa2	79.54% CP021652_03011	IS3 ssgr IS2	1627369	1627031	339
RS488	CP021652_01857	98.34% ISRso10_aa1	100% CP021652_03012	IS3 ssgr IS2	2013005	2013370	366
RS488	CP021652_01858	97.70% ISRso10_aa2	100% CP021652_03011	IS3 ssgr IS2	2013691	2014215	525
RS488	CP021652_02116	97.70% ISRso10_aa2	100% CP021652_03011	IS3 ssgr IS2	2300792	2300268	525
RS488	CP021652_02117	98.34% ISRso10_aa1	100% CP021652_03012	IS3 ssgr IS2	2301478	2301113	366
RS488	CP021652_02618	98.34% ISRso10_aa1	100% CP021652_03012	IS3 ssgr IS2	2816405	2816770	366
RS488	CP021652_02619	97.70% ISRso10_aa2	100% CP021652_03011	IS3 ssgr IS2	2817091	2817615	525

RS488	CP021652_03011	97.70% ISRso10_aa2	100% CP021652_02619	IS3 ssgr IS2	3221043	3220519	525
RS488	CP021652_03012	98.34% ISRso10_aa1	100% CP021652_02618	IS3 ssgr IS2	3221729	3221364	366
RS488	CP021652_02112	100% ISRso20_aa2	100% CP021652_02123	IS3 ssgr IS3	2297592	2296750	843
RS488	CP021652_02113	100% ISRso20_aa1	100% CP021652_02122	IS3 ssgr IS3	2297933	2297637	297
RS488	CP021652_02122	100% ISRso20_aa1	100% CP021652_02113	IS3 ssgr IS3	2305947	2306243	297
RS488	CP021652_02123	100% ISRso20_aa2	100% CP021652_02112	IS3 ssgr IS3	2306288	2307130	843
RS488	CP021652_01042	38.55% ISGur11_aa1	No hit	IS481	1140542	1142224	1683
RS488	CP021652_00068	88.37% ISAzo23_aa1	100% CP021652_03041	IS5 ssgr IS1031	72573	71767	807
RS488	CP021652_00762	88.37% ISAzo23_aa1	100% CP021652_03041	IS5 ssgr IS1031	855996	85519	807
RS488	CP021652_02162	88.37% ISAzo23_aa1	100% CP021652_03041	IS5 ssgr IS1031	2344864	2345670	807
RS488	CP021652_03041	88.37% ISAzo23_aa1	100% CP021652_02162	IS5 ssgr IS1031	3253867	3254673	807
RS488	CP021652_00050	100% IS1021_aa1	100% CP021652_03180	IS5 ssgr IS5	50636	51622	987
RS488	CP021652_00056	100% IS1021_aa1	100% CP021652_03180	IS5 ssgr IS5	58608	57622	987
RS488	CP021652_00063	100% IS1021_aa1	100% CP021652_03180	IS5 ssgr IS5	65759	64773	987
RS488	CP021652_00070	100% IS1021_aa1	100% CP021652_03180	IS5 ssgr IS5	76002	75016	987
RS488	CP021652_00247	100% IS1021_aa1	100% CP021652_03180	IS5 ssgr IS5	269562	270548	987
RS488	CP021652_00270	100% IS1021_aa1	100% CP021652_03180	IS5 ssgr IS5	306964	305978	987
RS488	CP021652_00272	100% IS1021_aa1	100% CP021652_03180	IS5 ssgr IS5	308712	309698	987
RS488	CP021652_00274	100% IS1021_aa1	100% CP021652_03180	IS5 ssgr IS5	320244	32123	987
RS488	CP021652_00285	100% IS1021_aa1	100% CP021652_03180	IS5 ssgr IS5	344822	343836	987
RS488	CP021652_00361	100% IS1021_aa1	100% CP021652_03180	IS5 ssgr IS5	428818	429804	987
RS488	CP021652_00851	100% IS1021_aa1	100% CP021652_03180	IS5 ssgr IS5	950126	94914	987
RS488	CP021652_00952	100% IS1021_aa1	100% CP021652_03180	IS5 ssgr IS5	1051547	1052533	987
RS488	CP021652_01071	100% IS1021_aa1	100% CP021652_03180	IS5 ssgr IS5	1162855	1163841	987
RS488	CP021652_01108	100% IS1021_aa1	100% CP021652_03180	IS5 ssgr IS5	1204827	1203841	987
RS488	CP021652_01142	100% IS1021_aa1	100% CP021652_03180	IS5 ssgr IS5	1238759	1237773	987
RS488	CP021652_01156	100% IS1021_aa1	100% CP021652_03180	IS5 ssgr IS5	1254019	1253033	987
RS488	CP021652_01161	100% IS1021_aa1	100% CP021652_03180	IS5 ssgr IS5	1257112	1258098	987
RS488	CP021652_01325	100% IS1021_aa1	100% CP021652_03180	IS5 ssgr IS5	1439064	1438078	987
RS488	CP021652_01925	100% IS1021_aa1	100% CP021652_03180	IS5 ssgr IS5	2088446	2087460	987
RS488	CP021652_01927	100% IS1021_aa1	100% CP021652_03180	IS5 ssgr IS5	2089157	2090143	987
RS488	CP021652_01981	100% IS1021_aa1	100% CP021652_03180	IS5 ssgr IS5	2150134	2149148	987
RS488	CP021652_01984	100% IS1021_aa1	100% CP021652_03180	IS5 ssgr IS5	2152344	2151358	987
RS488	CP021652_02118	100% IS1021_aa1	100% CP021652_03180	IS5 ssgr IS5	2301679	2302665	987
RS488	CP021652_02161	100% IS1021_aa1	100% CP021652_03180	IS5 ssgr IS5	2344740	2343754	987

RS488	CP021652_02202	100% IS1021_aa1	100% CP021652_03180	IS5 ssgr IS5	2389002	2389988	987
RS488	CP021652_02207	100% IS1021_aa1	100% CP021652_03180	IS5 ssgr IS5	2393803	2392817	987
RS488	CP021652_02237	100% IS1021_aa1	100% CP021652_03180	IS5 ssgr IS5	2428288	2427302	987
RS488	CP021652_02323	100% IS1021_aa1	100% CP021652_03180	IS5 ssgr IS5	2514799	2515785	987
RS488	CP021652_02384	100% IS1021_aa1	100% CP021652_03180	IS5 ssgr IS5	2574307	2575293	987
RS488	CP021652_02389	100% IS1021_aa1	100% CP021652_03180	IS5 ssgr IS5	2581226	2580240	987
RS488	CP021652_02511	100% IS1021_aa1	100% CP021652_03180	IS5 ssgr IS5	2707852	2708838	987
RS488	CP021652_02533	100% IS1021_aa1	100% CP021652_03180	IS5 ssgr IS5	2733056	2734042	987
RS488	CP021652_02598	100% IS1021_aa1	100% CP021652_03180	IS5 ssgr IS5	2793853	2794839	987
RS488	CP021652_02608	100% IS1021_aa1	100% CP021652_03180	IS5 ssgr IS5	2805867	2806853	987
RS488	CP021652_02611	100% IS1021_aa1	100% CP021652_03180	IS5 ssgr IS5	2810906	2809920	987
RS488	CP021652_02981	100% IS1021_aa1	100% CP021652_03180	IS5 ssgr IS5	3187098	3186112	987
RS488	CP021652_03040	100% IS1021_aa1	100% CP021652_03180	IS5 ssgr IS5	3252699	3253685	987
RS488	CP021652_03180	100% IS1021_aa1	100% CP021652_03040	IS5 ssgr IS5	3406083	3405097	987
RS488	CP021652_02592	45.66% ISShvi3_aa1	50.75% CP021652_03166	IS91	2789756	2790682	927
RS488	CP021652_03166	48.75% ISWz1_aa1	51.66% CP021652_02592	IS91	3389774	3390763	990
RS488	CP021652_00091	40.40% ISSm4_aa1	No hit	ISL3	102029	103474	1446
RS488	CP021652_01005	75.88% ISSm4_aa2	54.74% CP021652_02047	ISL3	1107497	1106457	1041
RS488	CP021652_02047	49.68% ISSm4_aa2	54.74% CP021652_01005	ISL3	2224119	2225180	1062
RS488	CP021652_01976	68.37% ISKpn21_aa1	No hit	ISNCY ssgr IS1202	2145815	2144949	867
RS488	CP021652_00696	53.33% TnShfr1_aa1	No hit	Tn3	779642	780868	1227
RS488	CP021652_01128	64.08% ISPa38_aa1	46.44% CP021652_02120	Tn3	1228548	1229138	591
RS488	CP021652_02120	52.63% ISSba14_aa1	46.44% CP021652_01128	Tn3	2303966	2304547	582
RS488	CP021652_02644	53.84% ISMpo10_aa1	58.20% CP021652_03073	Tn3	2841185	2840613	573
CFBP2957	RCFBP_10042	98.92% ISRso7_aa1	100% RCFBP_20548	IS256	41922	42767	846
CFBP2957	RCFBP_10043	97.96% ISRso7_aa1	100% RCFBP_21270	IS256	42556	4317	615
CFBP2957	RCFBP_11130	90.24% ISRso7_aa1	90.24% RCFBP_21270	IS256	1170682	1170344	339
CFBP2957	RCFBP_11131	70.58% ISRso7_aa1	72.94% RCFBP_20548	IS256	1170866	1170600	267
CFBP2957	RCFBP_11132	87.43% ISRso7_aa1	89.37% RCFBP_21270	IS256	1171589	1170918	672
CFBP2957	RCFBP_11589	97.35% ISRso7_aa1	99.27% RCFBP_21270	IS256	1636082	1634832	1251
CFBP2957	RCFBP_20548	98.31% ISRso7_aa1	98.79% RCFBP_21270	IS256	2505916	2507166	1251
CFBP2957	RCFBP_21270	98.07% ISRso7_aa1	99.27% RCFBP_11589	IS256	3218228	3219478	1251
CFBP2957	RCFBP_11104	95.74% ISRso10_aa2	100% RCFBP_20551	IS3 ssgr IS2	1142836	1141988	849
CFBP2957	RCFBP_11105	98.36% ISRso10_aa1	100% RCFBP_20550	IS3 ssgr IS2	1143228	1142833	396

CFBP2957	RCFBP_20390	67.53% ISRso10_aa2	70.68% RCFBP_20551	IS3 ssgr IS2	2356915	2357181	267
CFBP2957	RCFBP_20550	98.36% ISRso10_aa1	100% RCFBP_11105	IS3 ssgr IS2	2507519	2507908	390
CFBP2957	RCFBP_20551	95.74% ISRso10_aa2	100% RCFBP_11104	IS3 ssgr IS2	2507905	2508753	849
CFBP2957	RCFBP_10007	97.72% ISRso16_aa1	96.59% RCFBP_11107	IS3 ssgr IS407	7134	74	267
CFBP2957	RCFBP_10006	98.03% ISRso16_aa2	67.10% RCFBP_21243	IS3 ssgr IS407	7421	7897	477
CFBP2957	RCFBP_10008	92.30% ISRso16_aa2	64.46% RCFBP_10364	IS3 ssgr IS407	7884	8276	393
CFBP2957	RCFBP_10016	82.95% ISAeme6_aa1	68.49% RCFBP_21244	IS3 ssgr IS407	19841	20107	267
CFBP2957	RCFBP_10017	84.58% ISXac2_aa3	59.92% RCFBP_21243	IS3 ssgr IS407	20128	20928	801
CFBP2957	RCFBP_10364	76% ISDet2_aa2	100% RCFBP_21243	IS3 ssgr IS407	378385	377558	828
CFBP2957	RCFBP_10365	89.41% ISSme1_aa1	100% RCFBP_21244	IS3 ssgr IS407	378681	378421	261
CFBP2957	RCFBP_11107	96.59% ISRso16_aa1	96.59% RCFBP_10007	IS3 ssgr IS407	1144570	1144836	267
CFBP2957	RCFBP_11108	99.01% ISRso16_aa2	97.40% RCFBP_10006	IS3 ssgr IS407	1144869	1145333	465
CFBP2957	RCFBP_11109	97.14% ISRso16_aa2	65.07% RCFBP_21243	IS3 ssgr IS407	1145197	1145595	399
CFBP2957	RCFBP_11910	80.79% ISDet2_aa2	100% RCFBP_21243	IS3 ssgr IS407	1949294	1948794	501
CFBP2957	RCFBP_20543	76% ISDet2_aa2	100% RCFBP_10364	IS3 ssgr IS407	2503406	2502582	825
CFBP2957	RCFBP_20544	89.41% ISSme1_aa1	100% RCFBP_21244	IS3 ssgr IS407	2503705	2503445	261
CFBP2957	RCFBP_21243	76% ISDet2_aa2	100% RCFBP_10364	IS3 ssgr IS407	3186132	3185308	825
CFBP2957	RCFBP_21244	89.41% ISSme1_aa1	100% RCFBP_20544	IS3 ssgr IS407	3186431	3186171	261
CFBP2957	RCFBP_11695	44.22% ISAzs36_aa2	No hit	IS481	1753241	1751952	129
CFBP2957	RCFBP_11696	41.32% ISAzs36_aa1	No hit	IS481	1755037	1753238	18
CFBP2957	RCFBP_10953	88.61% IS1421_aa1	100% RCFBP_20595	IS5 ssgr IS427	988807	989211	405
CFBP2957	RCFBP_10954	81.63% IS1421_aa2	67.44% RCFBP_11524	IS5 ssgr IS427	989316	989612	297
CFBP2957	RCFBP_11524	67.85% IS1421_aa2	67.44% RCFBP_10954	IS5 ssgr IS427	1562265	1562047	219
CFBP2957	RCFBP_20595	93.58% IS1421_aa1	100% RCFBP_10953	IS5 ssgr IS427	2560445	2560714	270
CFBP2957	RCFBP_10852	47.96% ISMno23_aa1	51.76% RCFBP_21393	IS91	891054	890128	927
CFBP2957	RCFBP_21393	48.25% ISWz1_aa1	52.42% RCFBP_10852	IS91	3356530	3357519	990
CFBP2957	RCFBP_10068	41.25% ISSm4_aa1	No hit	ISL3	7122	72665	1446
CFBP2957	RCFBP_11648	49.37% ISSm4_aa2	No hit	ISL3	1701079	1700018	1062
CFBP2957	RCFBP_20621	61.76% ISSm4_aa2	No hit	ISL3	2580690	2581004	315
CFBP2957	RCFBP_20622	78.33% ISSm4_aa2	55.96% RCFBP_11648	ISL3	2580977	2581729	753
CFBP2957	RCFBP_10484	0% newcandidate	not_found	New_Family	499726	500451	726
CFBP2957	RCFBP_10807	0% newcandidate	not_found	New_Family	850271	850068	204
CFBP2957	RCFBP_20813	0% newcandidate	not_found	New_Family	2766373	2766441	69
CFBP2957	RCFBP_10234	53.70% ISMpo10_aa3	58.82% RCFBP_10259	Tn3	246075	244735	1341
CFBP2957	RCFBP_11588	48.38% TnAs1_aa5	No hit	Tn3	1634140	1634727	588

CFBP2957	RCFBP_20883	53.84% ISMpo10_aa1	56.33% RCFBP_21306	Tn3	2821971	2821393	579
UW551	96.09% ISBma3_aa1	No hit	IS110	38724	385988	1253	1
UW551	41.04% ISFsp3_aa2	55.86% B7R79_02845	IS21	1635609	1637326	1718	1
UW551	94.61% ISRso10_aa3	79.54% B7R79_08870	IS3 ssgr IS2	286086	287296	1211	1
UW551	94.61% ISRso10_aa3	79.54% B7R79_08870	IS3 ssgr IS2	692625	691415	1211	1
UW551	94.61% ISRso10_aa3	79.54% B7R79_08870	IS3 ssgr IS2	1206620	1205410	1211	1
UW551	94.61% ISRso10_aa3	79.54% B7R79_08870	IS3 ssgr IS2	1494250	1493040	1211	1
UW551	82.95% ISRso10_aa2	No hit	IS3 ssgr IS2	1879856	1880194	339	
UW551	94.61% ISRso10_aa3	79.54% B7R79_08870	IS3 ssgr IS2	2246568	2245358	1211	1
UW551	94.61% ISRso10_aa3	79.54% B7R79_08870	IS3 ssgr IS2	3016866	3015656	1211	1
UW551	100% ISRso20_aa3	No hit	IS3 ssgr IS3	1203075	1201892	1184	
UW551	100% ISRso20_aa3	No hit	IS3 ssgr IS3	1211536	1212719	1184	
UW551	83.33% ISRso16_aa2	No hit	IS3 ssgr IS407	701837	701607	231	
UW551	38.55% ISGur11_aa1	No hit	IS481	2365463	2363781	1683	1
UW551	88.37% ISAzo23_aa1	100% B7R79_16240	IS5 ssgr IS1031	3218	4024	807	1
UW551	88.37% ISAzo23_aa1	100% B7R79_16240	IS5 ssgr IS1031	20612	21418	807	1
UW551	88.37% ISAzo23_aa1	100% B7R79_16240	IS5 ssgr IS1031	251452	250646	807	1
UW551	88.37% ISAzo23_aa1	100% B7R79_16240	IS5 ssgr IS1031	1164158	1163352	807	1
UW551	88.37% ISAzo23_aa1	100% B7R79_16240	IS5 ssgr IS1031	2650004	2650810	807	1
UW551	88.37% ISAzo23_aa1	100% B7R79_16240	IS5 ssgr IS1031	3433454	3434260	807	1
UW551	88.37% ISAzo23_aa1	100% B7R79_16240	IS5 ssgr IS1031	3450847	3451653	807	1
UW551	88.37% ISAzo23_aa1	100% B7R79_16160	IS5 ssgr IS1031	3468239	3469045	807	1
UW551	78.14% ISAzba7_aa3	No hit	IS5 ssgr IS427	249022	248217	806	1
UW551	86.56% IS1421_aa1	No hit	IS5 ssgr IS427	1420228	1420428	201	
UW551	78.14% ISAzba7_aa3	No hit	IS5 ssgr IS427	1692876	1692071	806	1
UW551	91.66% ISBmu23_aa1	100% B7R79_16225	IS5 ssgr IS5	3	775	773	
UW551	100% IS1021_aa1	100% B7R79_10885	IS5 ssgr IS5	10032	11018	987	1
UW551	100% IS1021_aa1	100% B7R79_10885	IS5 ssgr IS5	17183	18169	987	1
UW551	100% IS1021_aa1	100% B7R79_10885	IS5 ssgr IS5	27425	28411	987	1
UW551	100% IS1021_aa1	100% B7R79_10885	IS5 ssgr IS5	34576	35562	987	1
UW551	100% IS1021_aa1	100% B7R79_10885	IS5 ssgr IS5	42548	41562	987	1
UW551	100% IS1021_aa1	100% B7R79_10885	IS5 ssgr IS5	99237	100223	987	1
UW551	100% IS1021_aa1	100% B7R79_10885	IS5 ssgr IS5	25262	251634	987	1
UW551	100% IS1021_aa1	100% B7R79_10885	IS5 ssgr IS5	255117	254131	987	1

UW551	100% IS1021_aa1	100% B7R79_10885	IS5 ssgr IS5	320724	32171	987	1
UW551	100% IS1021_aa1	100% B7R79_10885	IS5 ssgr IS5	372833	371847	987	1
UW551	100% IS1021_aa1	100% B7R79_10885	IS5 ssgr IS5	698123	699109	987	1
UW551	100% IS1021_aa1	100% B7R79_10885	IS5 ssgr IS5	703162	702176	987	1
UW551	100% IS1021_aa1	100% B7R79_10885	IS5 ssgr IS5	715176	71419	987	1
UW551	100% IS1021_aa1	100% B7R79_10885	IS5 ssgr IS5	774233	773247	987	1
UW551	100% IS1021_aa1	100% B7R79_10885	IS5 ssgr IS5	777185	776199	987	1
UW551	100% IS1021_aa1	100% B7R79_10885	IS5 ssgr IS5	802389	801403	987	1
UW551	100% IS1021_aa1	100% B7R79_10885	IS5 ssgr IS5	934721	933735	987	1
UW551	100% IS1021_aa1	100% B7R79_10885	IS5 ssgr IS5	994229	993243	987	1
UW551	100% IS1021_aa1	100% B7R79_10885	IS5 ssgr IS5	1080736	1081722	987	1
UW551	100% IS1021_aa1	100% B7R79_10885	IS5 ssgr IS5	1115220	1116206	987	1
UW551	100% IS1021_aa1	100% B7R79_10885	IS5 ssgr IS5	1120021	1119035	987	1
UW551	100% IS1021_aa1	100% B7R79_10885	IS5 ssgr IS5	1164282	1165268	987	1
UW551	100% IS1021_aa1	100% B7R79_10885	IS5 ssgr IS5	1206821	1207807	987	1
UW551	100% IS1021_aa1	100% B7R79_10885	IS5 ssgr IS5	1357124	1358110	987	1
UW551	100% IS1021_aa1	100% B7R79_10885	IS5 ssgr IS5	1418099	1417113	987	1
UW551	100% IS1021_aa1	100% B7R79_10885	IS5 ssgr IS5	1418810	1419796	987	1
UW551	100% IS1021_aa1	100% B7R79_10885	IS5 ssgr IS5	2068158	2069144	987	1
UW551	100% IS1021_aa1	100% B7R79_10885	IS5 ssgr IS5	2251986	2252972	987	1
UW551	100% IS1021_aa1	100% B7R79_10885	IS5 ssgr IS5	2267246	2268232	987	1
UW551	100% IS1021_aa1	100% B7R79_16225	IS5 ssgr IS5	2300755	2302164	141	
UW551	100% IS1021_aa1	100% B7R79_10885	IS5 ssgr IS5	2343150	2342164	987	1
UW551	100% IS1021_aa1	100% B7R79_10885	IS5 ssgr IS5	2454457	2453471	987	1
UW551	100% IS1021_aa1	100% B7R79_10885	IS5 ssgr IS5	2555875	2556861	987	1
UW551	100% IS1021_aa1	100% B7R79_10885	IS5 ssgr IS5	3077183	3076197	987	1
UW551	100% IS1021_aa1	100% B7R79_10885	IS5 ssgr IS5	3161178	3162164	987	1
UW551	100% IS1021_aa1	100% B7R79_10885	IS5 ssgr IS5	3198583	3197597	987	1
UW551	100% IS1021_aa1	100% B7R79_10885	IS5 ssgr IS5	3200331	3201317	987	1
UW551	100% IS1021_aa1	100% B7R79_10885	IS5 ssgr IS5	3211862	3212848	987	1
UW551	100% IS1021_aa1	100% B7R79_10885	IS5 ssgr IS5	3236465	3235479	987	1
UW551	100% IS1021_aa1	100% B7R79_10885	IS5 ssgr IS5	3430025	3431011	987	1
UW551	100% IS1021_aa1	100% B7R79_10885	IS5 ssgr IS5	3440267	3441253	987	1
UW551	100% IS1021_aa1	100% B7R79_10885	IS5 ssgr IS5	3447418	3448404	987	1
UW551	100% IS1021_aa1	100% B7R79_10885	IS5 ssgr IS5	3457660	3458646	987	1

UW551	100% IS1021_aa1	100% B7R79_10885	IS5 ssgr IS5	3464811	3465797	987	1
UW551	48.75% ISWz1_aa1	52.26% B7R79_03430	IS91	115545	114556	990	1
UW551	48.79% ISMno23_aa1	51.35% B7R79_00530	IS91	719273	718347	927	1
UW551	49.68% ISSm4_aa2	54.74% B7R79_11385	ISL3	1285349	1284288	1062	1
UW551	75.88% ISSm4_aa2	54.74% B7R79_06140	ISL3	2398507	2399547	1041	1
UW551	40.40% ISSm4_aa1	No hit	ISL3	3403998	3402553	1446	1
UW551	70.07% ISKpn21_aa1	No hit	ISNCY ssgr IS1202	1361039	1362309	1271	
UW551	0% newcandidate	not_found	New_Family	2366579	2365467	1113	1
UW551	0% newcandidate	not_found	New_Family	2593689	2593892	204	
UW551	0% newcandidate	not_found	New_Family	2941190	2940465	726	
UW551	52.63% ISSba14_aa1	46.44% B7R79_10780	Tn3	1209555	1210136	582	
UW551	64.08% ISPa38_aa1	46.44% B7R79_05805	Tn3	2277457	2276867	591	
UW551	54.13% TnShfr1_aa1	45.96% B7R79_14525	Tn3	2726356	2725131	1226	

RS489	CDC59_14595	96.09% ISBma3_aa1	No hit	IS110	3121620	3122872	1253
RS489	CDC59_02090	95.53% ISRso10_aa3	100% CDC59_15085	IS3 ssgr IS2	489244	490454	1211
RS489	CDC59_05815	95.53% ISRso10_aa3	100% CDC59_15085	IS3 ssgr IS2	1260688	1261898	1211
RS489	CDC59_07540	49.41% IS1417_aa2	50.68% CDC59_15085	IS3 ssgr IS2	1627661	1627467	195
RS489	CDC59_09315	95.53% ISRso10_aa3	100% CDC59_15085	IS3 ssgr IS2	2013027	2014237	1211
RS489	CDC59_10610	95.53% ISRso10_aa3	100% CDC59_15085	IS3 ssgr IS2	2301497	2300287	1211
RS489	CDC59_13110	95.53% ISRso10_aa3	100% CDC59_15085	IS3 ssgr IS2	2816324	2817534	1211
RS489	CDC59_15085	95.53% ISRso10_aa3	100% CDC59_13110	IS3 ssgr IS2	3221556	3220346	1211
RS489	CDC59_10590	99.23% ISRso20_aa3	100% CDC59_10640	IS3 ssgr IS3	2297952	2296769	1184
RS489	CDC59_10640	99.23% ISRso20_aa3	100% CDC59_10590	IS3 ssgr IS3	2305966	2307149	1184
RS489	CDC59_13055	83.33% ISRso16_aa2	No hit	IS3 ssgr IS407	2807034	2807462	429
RS489	CDC59_05220	38.55% ISGur11_aa1	No hit	IS481	1140572	1142254	1683
RS489	CDC59_00345	88.37% ISAzo23_aa1	100% CDC59_15230	IS5 ssgr IS1031	72603	71797	807
RS489	CDC59_03815	88.37% ISAzo23_aa1	100% CDC59_15230	IS5 ssgr IS1031	856021	855215	807
RS489	CDC59_10840	88.37% ISAzo23_aa1	100% CDC59_15230	IS5 ssgr IS1031	2344867	2345673	807
RS489	CDC59_15230	88.37% ISAzo23_aa1	100% CDC59_10840	IS5 ssgr IS1031	3253695	3254501	807
RS489	CDC59_08425	85.82% IS1421_aa3	99.62% CDC59_15245	IS5 ssgr IS427	1814423	1815228	806
RS489	CDC59_09655	86.56% IS1421_aa1	100% CDC59_08425	IS5 ssgr IS427	2087061	2086699	363
RS489	CDC59_15245	86.19% IS1421_aa3	99.62% CDC59_08425	IS5 ssgr IS427	3256125	3256930	806
RS489	CDC59_00250	98.78% IS1021_aa1	99.69% CDC59_05535	IS5 ssgr IS5	50662	51648	987
RS489	CDC59_00280	98.78% IS1021_aa1	99.69% CDC59_05535	IS5 ssgr IS5	58634	57648	987

RS489	CDC59_00315	98.47% IS1021_aa1	99.39% CDC59_05535	IS5 ssgr IS5	65788	64802	987
RS489	CDC59_00360	99.08% IS1021_aa1	100% CDC59_05535	IS5 ssgr IS5	76027	75041	987
RS489	CDC59_01225	99.08% IS1021_aa1	100% CDC59_05535	IS5 ssgr IS5	269638	270624	987
RS489	CDC59_01360	98.78% IS1021_aa1	99.69% CDC59_05535	IS5 ssgr IS5	30704	306054	987
RS489	CDC59_01370	99.08% IS1021_aa1	100% CDC59_05535	IS5 ssgr IS5	308788	309774	987
RS489	CDC59_01380	99.08% IS1021_aa1	100% CDC59_05535	IS5 ssgr IS5	320318	321304	987
RS489	CDC59_01435	98.78% IS1021_aa1	99.69% CDC59_05535	IS5 ssgr IS5	344918	343932	987
RS489	CDC59_01805	98.47% IS1021_aa1	99.39% CDC59_05535	IS5 ssgr IS5	428902	429888	987
RS489	CDC59_04265	99.08% IS1021_aa1	100% CDC59_05535	IS5 ssgr IS5	950171	949185	987
RS489	CDC59_04780	99.08% IS1021_aa1	100% CDC59_05535	IS5 ssgr IS5	1051572	1052558	987
RS489	CDC59_05365	98.47% IS1021_aa1	99.39% CDC59_05535	IS5 ssgr IS5	1162885	1163871	987
RS489	CDC59_05535	99.08% IS1021_aa1	100% CDC59_15225	IS5 ssgr IS5	1205290	1203881	141
RS489	CDC59_05710	98.47% IS1021_aa1	99.39% CDC59_05535	IS5 ssgr IS5	1238797	1237811	987
RS489	CDC59_05775	98.78% IS1021_aa1	99.69% CDC59_05535	IS5 ssgr IS5	1254057	1253071	987
RS489	CDC59_05800	98.47% IS1021_aa1	99.39% CDC59_05535	IS5 ssgr IS5	1257150	1258136	987
RS489	CDC59_06635	98.78% IS1021_aa1	99.69% CDC59_05535	IS5 ssgr IS5	1439167	1438181	987
RS489	CDC59_09660	98.78% IS1021_aa1	99.69% CDC59_05535	IS5 ssgr IS5	2088479	2087493	987
RS489	CDC59_09670	98.78% IS1021_aa1	99.69% CDC59_05535	IS5 ssgr IS5	2089190	2090176	987
RS489	CDC59_09940	98.78% IS1021_aa1	99.69% CDC59_05535	IS5 ssgr IS5	2150125	2149139	987
RS489	CDC59_09950	99.08% IS1021_aa1	100% CDC59_05535	IS5 ssgr IS5	2152335	2151349	987
RS489	CDC59_10615	98.78% IS1021_aa1	99.69% CDC59_05535	IS5 ssgr IS5	2301698	2302684	987
RS489	CDC59_10835	98.47% IS1021_aa1	99.39% CDC59_05535	IS5 ssgr IS5	2344743	2343757	987
RS489	CDC59_11055	98.47% IS1021_aa1	99.39% CDC59_05535	IS5 ssgr IS5	2388980	2389966	987
RS489	CDC59_11080	98.78% IS1021_aa1	99.69% CDC59_05535	IS5 ssgr IS5	2393781	2392795	987
RS489	CDC59_11230	98.78% IS1021_aa1	99.69% CDC59_05535	IS5 ssgr IS5	2428228	2427242	987
RS489	CDC59_11655	98.78% IS1021_aa1	99.69% CDC59_05535	IS5 ssgr IS5	2514740	2515726	987
RS489	CDC59_11955	98.47% IS1021_aa1	99.39% CDC59_05535	IS5 ssgr IS5	2574247	2575233	987
RS489	CDC59_11980	98.78% IS1021_aa1	99.69% CDC59_05535	IS5 ssgr IS5	2581166	2580180	987
RS489	CDC59_12580	98.47% IS1021_aa1	99.39% CDC59_05535	IS5 ssgr IS5	2707811	2708797	987
RS489	CDC59_12690	98.47% IS1021_aa1	99.39% CDC59_05535	IS5 ssgr IS5	2733008	2733994	987
RS489	CDC59_13000	98.47% IS1021_aa1	99.39% CDC59_05535	IS5 ssgr IS5	2793773	2794759	987
RS489	CDC59_13050	98.47% IS1021_aa1	99.39% CDC59_05535	IS5 ssgr IS5	2805778	2806764	987
RS489	CDC59_13075	98.78% IS1021_aa1	99.69% CDC59_05535	IS5 ssgr IS5	2810817	2809831	987
RS489	CDC59_14920	98.78% IS1021_aa1	99.69% CDC59_05535	IS5 ssgr IS5	3186931	3185945	987
RS489	CDC59_15225	99.08% IS1021_aa1	100% CDC59_05535	IS5 ssgr IS5	3252527	3253513	987

RS489	CDC59_15925	98.78% IS1021_aa1	99.69% CDC59_05535	IS5 ssgr IS5	3405902	3404916	987
RS489	CDC59_12970	48.37% ISMno23_aa1	52.09% CDC59_15855	IS91	2789679	2790605	927
RS489	CDC59_15855	48.25% ISWz1_aa1	52.75% CDC59_12970	IS91	3389590	3390579	990
RS489	CDC59_00455	40.26% ISSm4_aa1	No hit	ISL3	102059	103504	1446
RS489	CDC59_05040	75.88% ISSm4_aa2	55.48% CDC59_10265	ISL3	1107505	1106465	1041
RS489	CDC59_10265	49.37% ISSm4_aa2	55.48% CDC59_05040	ISL3	2224142	2225203	1062
RS489	CDC59_09915	70.07% ISKpn21_aa1	No hit	ISNCY ssgr IS1202	2146210	2144940	1271
RS489	CDC59_02490	0% newcandidate	not_found	New_Family	564911	565636	726
RS489	CDC59_04065	0% newcandidate	not_found	New_Family	91235	912147	204
RS489	CDC59_05215	0% newcandidate	not_found	New_Family	1139456	1140568	1113
RS489	CDC59_05635	64.08% ISPa38_aa1	46.44% CDC59_10625	Tn3	1228589	1229179	591
RS489	CDC59_10625	52.63% ISSba14_aa1	46.44% CDC59_05635	Tn3	2303985	2304566	582
RS489	CDC59_13225	53.84% ISMpo10_aa1	56.33% CDC59_15395	Tn3	2841026	2840505	522

OE1	IS_fe1a9e3d_01853	95.51% ISBma3_aa1	44.33% IS_fe1a9e3d_00127	IS110	2022809	2024014	1206
OE1	IS_fe1a9e3d_00127	88.75% ISBcen4_aa1	No hit	IS110 ssgr IS1111	149284	150303	102
OE1	IS_fe1a9e3d_02571	53.80% ISHpa1_aa1	No hit	IS1595 ssgr IS1016	2783122	2782343	780
OE1	IS_fe1a9e3d_02995	80.30% ISAav1_aa2	No hit	IS21	3240310	3239513	798
OE1	IS_fe1a9e3d_02996	75.93% ISAav1_aa1	No hit	IS21	3241796	3240300	1497
OE1	IS_fe1a9e3d_02637	99.28% ISRso11_aa2	50.22% IS_fe1a9e3d_02334	IS3 ssgr IS150	2851113	2850277	837
OE1	IS_fe1a9e3d_02638	100% ISRso11_aa1	No hit	IS3 ssgr IS150	2851643	2851110	534
OE1	IS_fe1a9e3d_03275	93.52% ISButh1_aa2	40.22% IS_fe1a9e3d_02637	IS3 ssgr IS2	3540292	3539456	837
OE1	IS_fe1a9e3d_03276	97.72% ISButh1_aa1	No hit	IS3 ssgr IS2	3540690	3540289	402
OE1	IS_fe1a9e3d_00888	89.23% IS222_aa2	98.65% IS_fe1a9e3d_02334	IS3 ssgr IS3	924515	923844	672
OE1	IS_fe1a9e3d_00889	88.23% IS222_aa1	100% IS_fe1a9e3d_02335	IS3 ssgr IS3	925006	924698	309
OE1	IS_fe1a9e3d_02334	88.78% IS222_aa2	98.65% IS_fe1a9e3d_00888	IS3 ssgr IS3	2535828	2535157	672
OE1	IS_fe1a9e3d_02335	82.81% ISPsy24_aa1	100% IS_fe1a9e3d_00889	IS3 ssgr IS3	2536318	2536061	258
OE1	IS_fe1a9e3d_00660	83.13% ISSme1_aa1	91.66% IS_fe1a9e3d_03535	IS3 ssgr IS407	704352	703963	390
OE1	IS_fe1a9e3d_03535	88.23% ISAtu5_aa1	91.66% IS_fe1a9e3d_00660	IS3 ssgr IS407	3816737	3816997	261
OE1	IS_fe1a9e3d_03536	76% ISDet2_aa2	44.92% IS_fe1a9e3d_02637	IS3 ssgr IS407	3817036	3817860	825
OE1	IS_fe1a9e3d_02346	80.43% ISAisp2_aa1	No hit	IS3 ssgr IS51	2546922	2547200	279
OE1	IS_fe1a9e3d_02347	86.77% ISAisp2_aa2	52.34% IS_fe1a9e3d_02637	IS3 ssgr IS51	2547197	2547988	792
OE1	IS_fe1a9e3d_00028	61.68% ISCro3_aa1	100% IS_fe1a9e3d_03538	IS4	31754	30426	1329
OE1	IS_fe1a9e3d_00034	61.68% ISCro3_aa1	100% IS_fe1a9e3d_03538	IS4	39825	38497	1329
OE1	IS_fe1a9e3d_00313	61.68% ISCro3_aa1	100% IS_fe1a9e3d_03538	IS4	35664	355312	1329

OE1	IS_fe1a9e3d_00807	61.68% ISCro3_aa1	100% IS_fe1a9e3d_03057	IS4	847209	848537	1329
OE1	IS_fe1a9e3d_01103	61.68% ISCro3_aa1	100% IS_fe1a9e3d_03538	IS4	1155120	1153792	1329
OE1	IS_fe1a9e3d_01158	61.68% ISCro3_aa1	100% IS_fe1a9e3d_03538	IS4	1210349	1211677	1329
OE1	IS_fe1a9e3d_01727	61.68% ISCro3_aa1	100% IS_fe1a9e3d_03538	IS4	1885904	1884576	1329
OE1	IS_fe1a9e3d_02250	61.68% ISCro3_aa1	100% IS_fe1a9e3d_03538	IS4	2436244	2437572	1329
OE1	IS_fe1a9e3d_03057	61.68% ISCro3_aa1	100% IS_fe1a9e3d_00807	IS4	3303519	3304847	1329
OE1	IS_fe1a9e3d_03245	61.68% ISCro3_aa1	100% IS_fe1a9e3d_03538	IS4	3503734	3502406	1329
OE1	IS_fe1a9e3d_03538	61.68% ISCro3_aa1	100% IS_fe1a9e3d_03245	IS4	3820394	3819066	1329
OE1	IS_fe1a9e3d_00220	98.90% ISRso1_aa1	100% IS_fe1a9e3d_01855	IS5	253495	254319	825
OE1	IS_fe1a9e3d_01855	98.90% ISRso1_aa1	100% IS_fe1a9e3d_00220	IS5	2025702	2026526	825
OE1	IS_fe1a9e3d_02744	98.17% ISRso1_aa1	98.54% IS_fe1a9e3d_01855	IS5	2956686	2955862	825
OE1	IS_fe1a9e3d_03264	98.90% ISRso1_aa1	99.27% IS_fe1a9e3d_01855	IS5	3528645	3527821	825
OE1	IS_fe1a9e3d_00253	86.06% IS1421_aa1	100% IS_fe1a9e3d_03456	IS5 ssgr IS427	286034	285627	864
OE1	IS_fe1a9e3d_00573	86.06% IS1421_aa1	100% IS_fe1a9e3d_03456	IS5 ssgr IS427	616696	616289	864
OE1	IS_fe1a9e3d_00653	84.03% IS1421_aa1	98.48% IS_fe1a9e3d_03456	IS5 ssgr IS427	697788	697351	864
OE1	IS_fe1a9e3d_00900	86.06% IS1421_aa1	100% IS_fe1a9e3d_03456	IS5 ssgr IS427	94702	946613	864
OE1	IS_fe1a9e3d_00908	86.06% IS1421_aa1	100% IS_fe1a9e3d_03456	IS5 ssgr IS427	954899	955306	864
OE1	IS_fe1a9e3d_01341	86.06% IS1421_aa1	100% IS_fe1a9e3d_03456	IS5 ssgr IS427	1398106	1397699	864
OE1	IS_fe1a9e3d_02333	86.06% IS1421_aa1	100% IS_fe1a9e3d_03456	IS5 ssgr IS427	2533542	2533949	864
OE1	IS_fe1a9e3d_02386	86.06% IS1421_aa1	100% IS_fe1a9e3d_03456	IS5 ssgr IS427	2593878	2593471	864
OE1	IS_fe1a9e3d_03257	86.06% IS1421_aa1	100% IS_fe1a9e3d_03456	IS5 ssgr IS427	3522257	3521850	864
OE1	IS_fe1a9e3d_03456	86.06% IS1421_aa1	100% IS_fe1a9e3d_03257	IS5 ssgr IS427	3720815	3721222	864
OE1	IS_fe1a9e3d_00668	94.20% IS1021_aa1	72.13% IS_fe1a9e3d_03307	IS5 ssgr IS5	71232	711334	987
OE1	IS_fe1a9e3d_01587	99.37% IS1405_aa1	100% IS_fe1a9e3d_03307	IS5 ssgr IS5	1672323	1673288	966
OE1	IS_fe1a9e3d_01856	99.37% IS1405_aa1	100% IS_fe1a9e3d_03307	IS5 ssgr IS5	2026748	2027713	966
OE1	IS_fe1a9e3d_01956	99.37% IS1405_aa1	100% IS_fe1a9e3d_03307	IS5 ssgr IS5	2126641	2127606	966
OE1	IS_fe1a9e3d_02379	99.37% IS1405_aa1	100% IS_fe1a9e3d_03307	IS5 ssgr IS5	2582285	2581320	966
OE1	IS_fe1a9e3d_02742	99.06% IS1405_aa1	99.68% IS_fe1a9e3d_03307	IS5 ssgr IS5	2954341	2955306	966
OE1	IS_fe1a9e3d_03209	99.37% IS1405_aa1	100% IS_fe1a9e3d_03307	IS5 ssgr IS5	3458940	3459905	966
OE1	IS_fe1a9e3d_03287	85.93% ISAau3_aa1	80% IS_fe1a9e3d_03307	IS5 ssgr IS5	3552076	3551807	270
OE1	IS_fe1a9e3d_03289	99.37% IS1405_aa1	100% IS_fe1a9e3d_03307	IS5 ssgr IS5	3553411	3552446	966
OE1	IS_fe1a9e3d_03307	99.37% IS1405_aa1	100% IS_fe1a9e3d_03289	IS5 ssgr IS5	3579893	3580858	966
OE1	IS_fe1a9e3d_01715	68.75% ISCARN39_aa2	No hit	IS630	1870086	1869886	201
OE1	IS_fe1a9e3d_00762	56.17% ISRsp1_aa4	No hit	IS66	801984	802532	549
OE1	IS_fe1a9e3d_01854	100% ISRso17_aa1	No hit	IS701	2024245	2025576	1332

OE1	IS_fe1a9e3d_00055	43.33% ISMno24_aa2	52.12% IS_fe1a9e3d_02627	IS91	6111	60121	990
OE1	IS_fe1a9e3d_00763	50% ISWz1_aa1	43.91% IS_fe1a9e3d_02627	IS91	803822	802677	1146
OE1	IS_fe1a9e3d_02627	47.5% ISTha3_aa2	51.50% IS_fe1a9e3d_00055	IS91	2839288	2840214	927
OE1	IS_fe1a9e3d_03501	38.97% ISKpn25_aa1	No hit	ISL3	3772943	3771498	1446
OE1	IS_fe1a9e3d_01604	64.60% ISKpn21_aa1	No hit	ISNCY ssgr IS1202	1690066	1690467	402
OE1	IS_fe1a9e3d_00906	55.06% ISMpo10_aa3	94.34% IS_fe1a9e3d_00986	Tn3	952716	954098	1383
OE1	IS_fe1a9e3d_00986	54.43% ISMpo10_aa3	94.34% IS_fe1a9e3d_00906	Tn3	1032490	1033869	138
OE1	IS_fe1a9e3d_03271	61.81% ISMpo10_aa3	96.96% IS_fe1a9e3d_03351	Tn3	3535804	3537192	1389
OE1	IS_fe1a9e3d_03351	61.81% ISMpo10_aa3	96.96% IS_fe1a9e3d_03271	Tn3	3611072	3612460	1389
K60	NCTK01000001_00210	95.51% ISBma3_aa1	47.36% NCTK01000001_00851	IS110	212113	213318	1206
K60	NCTK01000001_00850	78.78% ISBcen3_aa1	67.85% NCTK01000001_03240	IS110 ssgr IS1111	874019	874594	576
K60	NCTK01000001_00851	65.82% ISCARN80_aa1	67.77% NCTK01000001_03239	IS110 ssgr IS1111	87457	875064	495
K60	NCTK01000001_02633	93.04% ISPath1_aa1	98.47% NCTK01000001_03240	IS110 ssgr IS1111	2836327	2836722	396
K60	NCTK01000001_03239	95.03% ISPath1_aa1	96.07% NCTK01000001_02634	IS110 ssgr IS1111	3503598	3503089	510
K60	NCTK01000001_03240	92.24% ISPath1_aa1	98.47% NCTK01000001_02633	IS110 ssgr IS1111	3504016	3503621	396
K60	NCTK01000001_00218	91.30% ISBdo1_aa1	100% NCTK01000001_02652	IS1182	222084	221683	402
K60	NCTK01000001_00219	90.85% ISBma2_aa1	99.71% NCTK01000001_02652	IS1182	223133	222081	1053
K60	NCTK01000001_00374	91.30% ISBdo1_aa1	99.24% NCTK01000001_02652	IS1182	388308	387907	402
K60	NCTK01000001_00375	90.85% ISBma2_aa1	99.71% NCTK01000001_02652	IS1182	389357	388305	1053
K60	NCTK01000001_00521	91.24% ISBma2_aa1	100% NCTK01000001_02652	IS1182	551572	550088	1485
K60	NCTK01000001_00793	91.24% ISBma2_aa1	100% NCTK01000001_02652	IS1182	811458	812942	1485
K60	NCTK01000001_01122	90.85% ISBma2_aa1	99.71% NCTK01000001_02652	IS1182	1157223	1158275	1053
K60	NCTK01000001_01123	91.30% ISBdo1_aa1	100% NCTK01000001_02652	IS1182	1158272	1158673	402
K60	NCTK01000001_01390	91.02% ISBma2_aa1	99.79% NCTK01000001_02652	IS1182	1447832	1446348	1485
K60	NCTK01000001_01823	91.30% ISBdo1_aa1	100% NCTK01000001_02652	IS1182	1949074	1948673	402
K60	NCTK01000001_01824	93.02% ISBma2_aa1	100% NCTK01000001_02652	IS1182	1949460	1949071	390
K60	NCTK01000001_02262	91.24% ISBma2_aa1	100% NCTK01000001_02652	IS1182	2419102	2417618	1485
K60	NCTK01000001_02467	91.24% ISBma2_aa1	100% NCTK01000001_02652	IS1182	2646095	2647579	1485
K60	NCTK01000001_02652	91.24% ISBma2_aa1	100% NCTK01000001_02467	IS1182	2854309	2852825	1485
K60	NCTK01000001_02780	90.85% ISBma2_aa1	99.71% NCTK01000001_02652	IS1182	3000891	3001943	1053
K60	NCTK01000001_02781	91.30% ISBdo1_aa1	100% NCTK01000001_02652	IS1182	3001940	3002341	402
K60	NCTK01000001_03166	91.30% ISBdo1_aa1	100% NCTK01000001_02652	IS1182	3421941	3421540	402
K60	NCTK01000001_03167	90.85% ISBma2_aa1	99.71% NCTK01000001_02652	IS1182	3422990	3421938	1053
K60	NCTK01000001_03353	91.30% ISBdo1_aa1	100% NCTK01000001_02652	IS1182	3620658	3620257	402

K60	NCTK01000001_03354	90.85% ISBma2_aa1	99.71% NCTK01000001_02652	IS1182	3621707	3620655	1053
K60	NCTK01000001_03468	91.30% ISBdo1_aa1	100% NCTK01000001_02652	IS1182	3732982	3732581	402
K60	NCTK01000001_03469	90.85% ISBma2_aa1	99.71% NCTK01000001_02652	IS1182	3734031	3732979	1053
K60	NCTK01000001_00090	69.34% ISLsp2_aa1	100% NCTK01000001_03015	IS1595 ssgr ISPna2	84983	85981	999
K60	NCTK01000001_00359	69.34% ISLsp2_aa1	100% NCTK01000001_03015	IS1595 ssgr ISPna2	372992	371994	999
K60	NCTK01000001_02050	41.13% ISPto1_aa1	49.75% NCTK01000001_02698	IS1595 ssgr ISPna2	2189279	2190712	1434
K60	NCTK01000001_02698	51.45% ISLsp2_aa1	47.30% NCTK01000001_02050	IS1595 ssgr ISPna2	2912539	2913816	1278
K60	NCTK01000001_03015	69.34% ISLsp2_aa1	100% NCTK01000001_00359	IS1595 ssgr ISPna2	3273275	3272277	999
K60	NCTK01000001_03334	69.74% ISLsp2_aa1	100% NCTK01000001_03015	IS1595 ssgr ISPna2	3601970	3602923	954
K60	NCTK01000001_00861	81.08% IS1600_aa1	100% NCTK01000001_03376	IS21	888056	889582	1527
K60	NCTK01000001_00862	98.08% ISRme4_aa2	100% NCTK01000001_03375	IS21	889572	890357	786
K60	NCTK01000001_01536	82.88% ISCARN95_aa1	100% NCTK01000001_03253	IS21	1608906	1610030	1125
K60	NCTK01000001_01537	85.14% ISCARN95_aa2	99.60% NCTK01000001_03254	IS21	1610043	1610810	768
K60	NCTK01000001_01541	70.25% ISRme9_aa1	65.08% NCTK01000001_03253	IS21	1613541	1615058	1518
K60	NCTK01000001_01542	75.91% ISBmu3_aa2	65.69% NCTK01000001_01537	IS21	1615076	1615816	741
K60	NCTK01000001_01735	100% ISRso19_aa2	100% NCTK01000001_03371	IS21	1845067	1844279	789
K60	NCTK01000001_01736	97.04% ISRso19_aa1	100% NCTK01000001_03372	IS21	1846080	1845064	1017
K60	NCTK01000001_02022	98.08% ISRme4_aa2	100% NCTK01000001_03375	IS21	2150772	2149987	786
K60	NCTK01000001_02023	81.08% IS1600_aa1	100% NCTK01000001_03376	IS21	2152288	2150762	1527
K60	NCTK01000001_02319	100% ISRso19_aa2	100% NCTK01000001_03371	IS21	2476518	2475730	789
K60	NCTK01000001_02320	97.42% ISRso19_aa1	99.67% NCTK01000001_03372	IS21	2477453	2476515	939
K60	NCTK01000001_02321	81.08% IS1600_aa1	100% NCTK01000001_03376	IS21	2477608	2479134	1527
K60	NCTK01000001_02322	98.08% ISRme4_aa2	100% NCTK01000001_03375	IS21	2479124	2479909	786
K60	NCTK01000001_02454	81.08% IS1600_aa1	100% NCTK01000001_03376	IS21	2628629	2630155	1527
K60	NCTK01000001_02455	98.08% ISRme4_aa2	100% NCTK01000001_03375	IS21	2630145	2630930	786
K60	NCTK01000001_02888	90.56% ISRso19_aa1	92.92% NCTK01000001_03372	IS21	3127058	3126426	633
K60	NCTK01000001_03253	81.65% ISCARN95_aa1	100% NCTK01000001_01536	IS21	3513102	3514625	1524
K60	NCTK01000001_03254	84.73% ISCARN95_aa2	99.60% NCTK01000001_01537	IS21	3514638	3515405	768
K60	NCTK01000001_03371	100% ISRso19_aa2	100% NCTK01000001_02319	IS21	3636746	3635958	789
K60	NCTK01000001_03372	97.04% ISRso19_aa1	100% NCTK01000001_01736	IS21	3637759	3636743	1017
K60	NCTK01000001_03375	98.08% ISRme4_aa2	100% NCTK01000001_02455	IS21	3639816	3639031	786
K60	NCTK01000001_03376	81.08% IS1600_aa1	100% NCTK01000001_02454	IS21	3641332	3639806	1527
K60	NCTK01000001_00633	68.18% ISRso10_aa2	No hit	IS3 ssgr IS2	64875	649091	342
K60	NCTK01000001_03266	92.70% ISRso20_aa2	56% NCTK01000001_03328	IS3 ssgr IS3	3522096	3522800	705
K60	NCTK01000001_03252	91.52% ISRso16_aa2	67.08% NCTK01000001_03255	IS3 ssgr IS407	3512927	3512568	360

K60	NCTK01000001_03255	74.07% ISBam2_aa3	67.08% NCTK01000001_03252	IS3 ssgr IS407	3515604	3515900	297
K60	NCTK01000001_00821	89.28% IS401_aa1	87.5% NCTK01000001_00843	IS3 ssgr IS51	842672	842848	177
K60	NCTK01000001_00842	97.03% IS401_aa2	100% NCTK01000001_03328	IS3 ssgr IS51	860612	859698	915
K60	NCTK01000001_00843	96.26% IS401_aa1	100% NCTK01000001_03329	IS3 ssgr IS51	860932	860609	324
K60	NCTK01000001_03328	97.03% IS401_aa2	100% NCTK01000001_00842	IS3 ssgr IS51	3599260	3598346	915
K60	NCTK01000001_03329	96.26% IS401_aa1	100% NCTK01000001_00843	IS3 ssgr IS51	3599580	3599257	324
K60	NCTK01000001_00075	81.92% ISAzo5_aa1	99.76% NCTK01000001_02451	IS4 ssgr IS50	67028	65769	126
K60	NCTK01000001_00155	81.92% ISAzo5_aa1	99.52% NCTK01000001_01496	IS4 ssgr IS50	152624	153883	126
K60	NCTK01000001_01060	76.52% ISAzo5_aa1	98.64% NCTK01000001_02148	IS4 ssgr IS50	1092605	1093933	1329
K60	NCTK01000001_01303	76% ISAzo5_aa1	76% NCTK01000001_02451	IS4 ssgr IS50	1357286	1356768	519
K60	NCTK01000001_01304	87.93% ISAzo5_aa1	100% NCTK01000001_01528	IS4 ssgr IS50	1357498	1357286	213
K60	NCTK01000001_01312	77.06% ISAzo5_aa1	99.54% NCTK01000001_02148	IS4 ssgr IS50	1366935	1365607	1329
K60	NCTK01000001_01313	82.16% ISAzo5_aa1	100% NCTK01000001_02451	IS4 ssgr IS50	1368983	1367724	126
K60	NCTK01000001_01314	81.92% ISAzo5_aa1	100% NCTK01000001_01496	IS4 ssgr IS50	1373889	1372630	126
K60	NCTK01000001_01496	81.92% ISAzo5_aa1	100% NCTK01000001_01314	IS4 ssgr IS50	1555430	1556689	126
K60	NCTK01000001_01528	73.26% ISAzo5_aa1	72.28% NCTK01000001_01312	IS4 ssgr IS50	1600037	1600891	855
K60	NCTK01000001_01529	85.06% ISAzo5_aa1	81.29% NCTK01000001_02451	IS4 ssgr IS50	1600895	1601362	468
K60	NCTK01000001_02148	76.59% ISAzo5_aa1	99.54% NCTK01000001_01312	IS4 ssgr IS50	2305059	2306387	1329
K60	NCTK01000001_02451	82.16% ISAzo5_aa1	100% NCTK01000001_01313	IS4 ssgr IS50	2624558	2625817	126
K60	NCTK01000001_03009	81.68% ISAzo5_aa1	99.52% NCTK01000001_02451	IS4 ssgr IS50	3268008	3266749	126
K60	NCTK01000001_03250	77.06% ISAzo5_aa1	99.54% NCTK01000001_01312	IS4 ssgr IS50	3510427	3511755	1329
K60	NCTK01000001_03265	81.68% ISAzo5_aa1	99.76% NCTK01000001_01496	IS4 ssgr IS50	3520487	3521746	126
K60	NCTK01000001_00578	39.36% ISChy6_aa2	41.77% NCTK01000001_01731	IS481	606723	608972	225
K60	NCTK01000001_01731	39.14% ISGur11_aa1	40.18% NCTK01000001_00578	IS481	1836750	1838714	1965
K60	NCTK01000001_01994	97.08% ISRso1_aa1	No hit	IS5	2126065	2125241	825
K60	NCTK01000001_00866	65% ISCaa13_aa2	100% NCTK01000001_02132	IS5 ssgr IS427	893345	89298	366
K60	NCTK01000001_01315	65.28% ISCaa13_aa2	100% NCTK01000001_03369	IS5 ssgr IS427	1374864	1375229	366
K60	NCTK01000001_01349	65.28% ISCaa13_aa2	100% NCTK01000001_03369	IS5 ssgr IS427	1409326	1408961	366
K60	NCTK01000001_01843	65.28% ISCaa13_aa2	100% NCTK01000001_03369	IS5 ssgr IS427	1966678	1967043	366
K60	NCTK01000001_02132	65% ISCaa13_aa2	100% NCTK01000001_00866	IS5 ssgr IS427	2290108	2290473	366
K60	NCTK01000001_02723	65.28% ISCaa13_aa2	100% NCTK01000001_03369	IS5 ssgr IS427	2937621	2937256	366
K60	NCTK01000001_03008	88.78% ISCaa13_aa1	No hit	IS5 ssgr IS427	3266228	3266596	369
K60	NCTK01000001_03011	65.28% ISCaa13_aa2	100% NCTK01000001_03369	IS5 ssgr IS427	3269710	3270075	366
K60	NCTK01000001_03057	65.28% ISCaa13_aa2	100% NCTK01000001_03011	IS5 ssgr IS427	3314061	3313696	366
K60	NCTK01000001_03107	65.28% ISCaa13_aa2	100% NCTK01000001_03369	IS5 ssgr IS427	3366458	3366093	366

K60	NCTK01000001_03369	65.28% ISCaa13_aa2	100% NCTK01000001_03107	IS5 ssgr IS427	3634894	3635259	366
K60	NCTK01000001_03438	65% ISCaa13_aa2	99.17% NCTK01000001_03369	IS5 ssgr IS427	3699511	3699146	366
K60	NCTK01000001_00750	87.65% ISBmu20_aa1	42.00% NCTK01000001_00822	IS5 ssgr IS5	77067	769927	744
K60	NCTK01000001_00822	61.32% ISAzo11_aa1	42.00% NCTK01000001_00750	IS5 ssgr IS5	842972	844042	1071
K60	NCTK01000001_02192	97.51% ISRso9_aa1	No hit	IS5 ssgr IS5	2354368	2354907	540
K60	NCTK01000001_00233	89.97% ISCARN25_aa1	99.70% NCTK01000001_00847	IS630	233661	23468	102
K60	NCTK01000001_00841	92.26% ISAzo32_aa1	100% NCTK01000001_01733	IS630	858335	859312	978
K60	NCTK01000001_00847	89.67% ISCARN25_aa1	99.70% NCTK01000001_00233	IS630	862834	863853	102
K60	NCTK01000001_01183	85.63% ISAzo9_aa2	99.73% NCTK01000001_01306	IS630	1223647	1222520	1128
K60	NCTK01000001_01306	85.83% ISAzo9_aa2	99.73% NCTK01000001_01183	IS630	1358388	1359515	1128
K60	NCTK01000001_01733	92.26% ISAzo32_aa1	100% NCTK01000001_00841	IS630	1840353	1841330	978
K60	NCTK01000001_03383	81.05% ISRso5_aa1	44.02% NCTK01000001_01306	IS630	3650316	3649240	1077
K60	NCTK01000001_03261	81.88% ISPpu19_aa3	No hit	IS66	3518692	3519120	429
K60	NCTK01000001_00840	66.90% ISMmg4_aa1	100% NCTK01000001_02459	IS701	85691	858124	1215
K60	NCTK01000001_01153	72.76% ISPosp2_aa1	91.66% NCTK01000001_02459	IS701	1189707	1190801	1095
K60	NCTK01000001_01289	67.23% ISMmg4_aa1	99.48% NCTK01000001_00840	IS701	1339885	1338713	1173
K60	NCTK01000001_02052	66.90% ISMmg4_aa1	100% NCTK01000001_02459	IS701	2194262	2195476	1215
K60	NCTK01000001_02459	66.90% ISMmg4_aa1	100% NCTK01000001_02052	IS701	2635616	2634402	1215
K60	NCTK01000001_03333	67% ISMmg4_aa1	100% NCTK01000001_02459	IS701	3600976	3602016	1041
K60	NCTK01000001_00890	64.51% ISSm4_aa2	No hit	ISL3	921043	921357	315
K60	NCTK01000001_00891	76.27% ISSm4_aa2	57.26% NCTK01000001_03496	ISL3	921354	922082	729
K60	NCTK01000001_01777	41.25% ISSm4_aa1	No hit	ISL3	1900111	1901556	1446
K60	NCTK01000001_03496	49.37% ISSm4_aa2	57.26% NCTK01000001_00891	ISL3	3763260	3762199	1062
FJAT1458	IS_9b9ce816_03326	61.67% ISMno14_aa1	45.45% IS_9b9ce816_03639	IS110 ssgr IS1111	3514230	3513217	1014
FJAT1458	IS_9b9ce816_03639	88.46% ISBcen4_aa1	45.45% IS_9b9ce816_03326	IS110 ssgr IS1111	3823429	3822410	102
FJAT1458	IS_9b9ce816_03097	83.75% ISBusp4_aa1	No hit	IS1182	3274969	3273524	1446
FJAT1458	IS_9b9ce816_01013	54.76% ISHpa1_aa1	No hit	IS1595 ssgr IS1016	1067840	1068619	780
FJAT1458	IS_9b9ce816_00259	49.48% ISHvo5_aa1	99.61% IS_9b9ce816_02855	IS1595 ssgr ISH4	273215	274003	789
FJAT1458	IS_9b9ce816_02855	49.48% ISHvo5_aa1	99.61% IS_9b9ce816_00259	IS1595 ssgr ISH4	3025506	3024718	789
FJAT1458	IS_9b9ce816_00081	100% ISRso11_aa1	100% IS_9b9ce816_02114	IS3 ssgr IS150	94149	94682	534
FJAT1458	IS_9b9ce816_00082	99.64% ISRso11_aa2	100% IS_9b9ce816_02113	IS3 ssgr IS150	94679	95515	837
FJAT1458	IS_9b9ce816_02113	99.64% ISRso11_aa2	100% IS_9b9ce816_00082	IS3 ssgr IS150	2254025	2253189	837
FJAT1458	IS_9b9ce816_02114	100% ISRso11_aa1	100% IS_9b9ce816_00081	IS3 ssgr IS150	2254555	2254022	534
FJAT1458	IS_9b9ce816_01165	88.23% IS222_aa1	60.91% IS_9b9ce816_01121	IS3 ssgr IS3	1229164	1229472	309

FJAT1458	IS_9b9ce816_01166	89.23% IS222_aa2	56.22% IS_9b9ce816_01120	IS3 ssgr IS3	1229655	1230326	672
FJAT1458	IS_9b9ce816_03114	85.71% ISAtu5_aa3	No hit	IS3 ssgr IS407	3290906	3291295	390
FJAT1458	IS_9b9ce816_00191	81.52% ISAisp2_aa1	100% IS_9b9ce816_03561	IS3 ssgr IS51	20691	207188	279
FJAT1458	IS_9b9ce816_00192	89.34% ISAisp2_aa2	100% IS_9b9ce816_03562	IS3 ssgr IS51	207185	20806	876
FJAT1458	IS_9b9ce816_01120	89.34% ISAisp2_aa2	98.28% IS_9b9ce816_03562	IS3 ssgr IS51	1183190	1182315	876
FJAT1458	IS_9b9ce816_01121	81.52% ISAisp2_aa1	97.82% IS_9b9ce816_03561	IS3 ssgr IS51	1183465	1183187	279
FJAT1458	IS_9b9ce816_01154	86.38% ISAisp2_aa2	94.94% IS_9b9ce816_01120	IS3 ssgr IS51	1218800	1218009	792
FJAT1458	IS_9b9ce816_01155	80.43% ISAisp2_aa1	96.73% IS_9b9ce816_03561	IS3 ssgr IS51	1219075	1218797	279
FJAT1458	IS_9b9ce816_02891	89.34% ISAisp2_aa2	100% IS_9b9ce816_03562	IS3 ssgr IS51	3071153	3070278	876
FJAT1458	IS_9b9ce816_02892	81.52% ISAisp2_aa1	100% IS_9b9ce816_03561	IS3 ssgr IS51	3071428	3071150	279
FJAT1458	IS_9b9ce816_03561	81.52% ISAisp2_aa1	100% IS_9b9ce816_02892	IS3 ssgr IS51	3749495	3749773	279
FJAT1458	IS_9b9ce816_03562	89.34% ISAisp2_aa2	100% IS_9b9ce816_02891	IS3 ssgr IS51	3749770	3750645	876
FJAT1458	IS_9b9ce816_00280	61.68% ISCro3_aa1	100% IS_9b9ce816_03737	IS4	301471	302799	1329
FJAT1458	IS_9b9ce816_00350	61.68% ISCro3_aa1	100% IS_9b9ce816_03737	IS4	384121	382793	1329
FJAT1458	IS_9b9ce816_00556	61.68% ISCro3_aa1	100% IS_9b9ce816_03737	IS4	575551	574223	1329
FJAT1458	IS_9b9ce816_00595	61.68% ISCro3_aa1	100% IS_9b9ce816_03737	IS4	614051	615379	1329
FJAT1458	IS_9b9ce816_00845	61.44% ISCro3_aa1	100% IS_9b9ce816_01251	IS4	898646	897318	1329
FJAT1458	IS_9b9ce816_01071	61.68% ISCro3_aa1	100% IS_9b9ce816_03737	IS4	1128939	1127611	1329
FJAT1458	IS_9b9ce816_01251	61.44% ISCro3_aa1	100% IS_9b9ce816_00845	IS4	1328069	1329397	1329
FJAT1458	IS_9b9ce816_01906	62.82% ISCro3_aa1	100% IS_9b9ce816_03737	IS4	1979548	1980483	936
FJAT1458	IS_9b9ce816_02543	61.44% ISCro3_aa1	100% IS_9b9ce816_02656	IS4	2716556	2715228	1329
FJAT1458	IS_9b9ce816_02656	61.44% ISCro3_aa1	100% IS_9b9ce816_02543	IS4	2821826	2820498	1329
FJAT1458	IS_9b9ce816_02996	61.44% ISCro3_aa1	100% IS_9b9ce816_03737	IS4	3174731	3173403	1329
FJAT1458	IS_9b9ce816_03737	61.68% ISCro3_aa1	100% IS_9b9ce816_01071	IS4	3941087	3939759	1329
FJAT1458	IS_9b9ce816_01907	65% ISCro6_aa1	100% IS_9b9ce816_02996	IS4 ssgr IS4	1980520	1980876	357
FJAT1458	IS_9b9ce816_02860	78.03% ISAzo5_aa1	No hit	IS4 ssgr IS50	3032229	3033554	1326
FJAT1458	IS_9b9ce816_00260	93.43% ISRso1_aa1	94.16% IS_9b9ce816_01920	IS5	274725	275546	822
FJAT1458	IS_9b9ce816_00262	98.17% ISRso1_aa1	98.54% IS_9b9ce816_01920	IS5	277439	278263	825
FJAT1458	IS_9b9ce816_01920	97.44% ISRso1_aa1	98.90% IS_9b9ce816_02873	IS5	1996902	1998149	1248
FJAT1458	IS_9b9ce816_02873	97.81% ISRso1_aa1	98.90% IS_9b9ce816_01920	IS5	3050777	3051601	825
FJAT1458	IS_9b9ce816_00213	99.37% IS1405_aa1	100% IS_9b9ce816_02862	IS5 ssgr IS5	224094	223129	966
FJAT1458	IS_9b9ce816_00255	85.93% ISAau3_aa1	80% IS_9b9ce816_02862	IS5 ssgr IS5	26704	267309	270
FJAT1458	IS_9b9ce816_00285	99.06% IS1405_aa1	100% IS_9b9ce816_03509	IS5 ssgr IS5	307339	308304	966
FJAT1458	IS_9b9ce816_01196	94.51% IS1021_aa1	100% IS_9b9ce816_03511	IS5 ssgr IS5	1266028	1267014	987
FJAT1458	IS_9b9ce816_01343	99.06% IS1405_aa1	100% IS_9b9ce816_03509	IS5 ssgr IS5	1426150	1427115	966

FJAT1458	IS_9b9ce816_01351	94.51% IS1021_aa1	100% IS_9b9ce816_03511	IS5 ssgr IS5	1433245	1432259	987
FJAT1458	IS_9b9ce816_01372	99.06% IS1405_aa1	100% IS_9b9ce816_03509	IS5 ssgr IS5	1458635	1457670	966
FJAT1458	IS_9b9ce816_01534	94.51% IS1021_aa1	100% IS_9b9ce816_03511	IS5 ssgr IS5	1623941	1622955	987
FJAT1458	IS_9b9ce816_01538	94.51% IS1021_aa1	100% IS_9b9ce816_03511	IS5 ssgr IS5	1629161	1630147	987
FJAT1458	IS_9b9ce816_01668	99.06% IS1405_aa1	100% IS_9b9ce816_03509	IS5 ssgr IS5	1759784	1758819	966
FJAT1458	IS_9b9ce816_01808	94.51% IS1021_aa1	100% IS_9b9ce816_03511	IS5 ssgr IS5	1900997	1900011	987
FJAT1458	IS_9b9ce816_01824	99.06% IS1405_aa1	100% IS_9b9ce816_03509	IS5 ssgr IS5	1914572	1915537	966
FJAT1458	IS_9b9ce816_01848	94.51% IS1021_aa1	100% IS_9b9ce816_03511	IS5 ssgr IS5	1931172	1930186	987
FJAT1458	IS_9b9ce816_01893	94.51% IS1021_aa1	100% IS_9b9ce816_03511	IS5 ssgr IS5	1964524	1963538	987
FJAT1458	IS_9b9ce816_02008	94.51% IS1021_aa1	100% IS_9b9ce816_03511	IS5 ssgr IS5	2131295	2132281	987
FJAT1458	IS_9b9ce816_02579	99.06% IS1405_aa1	100% IS_9b9ce816_03509	IS5 ssgr IS5	2743663	2742698	966
FJAT1458	IS_9b9ce816_02584	94.51% IS1021_aa1	100% IS_9b9ce816_03511	IS5 ssgr IS5	2749073	2748087	987
FJAT1458	IS_9b9ce816_02605	94.51% IS1021_aa1	100% IS_9b9ce816_03511	IS5 ssgr IS5	2770289	2771275	987
FJAT1458	IS_9b9ce816_02862	99.37% IS1405_aa1	100% IS_9b9ce816_00213	IS5 ssgr IS5	3035899	3034934	966
FJAT1458	IS_9b9ce816_03471	94.51% IS1021_aa1	100% IS_9b9ce816_03511	IS5 ssgr IS5	3650652	3651638	987
FJAT1458	IS_9b9ce816_03509	99.06% IS1405_aa1	100% IS_9b9ce816_02579	IS5 ssgr IS5	3694584	3695549	966
FJAT1458	IS_9b9ce816_03511	94.51% IS1021_aa1	100% IS_9b9ce816_03471	IS5 ssgr IS5	3696816	3695830	987
FJAT1458	IS_9b9ce816_00956	47.5% ISTha3_aa2	51.50% IS_9b9ce816_03710	IS91	1009808	1008882	927
FJAT1458	IS_9b9ce816_03710	43.33% ISMno24_aa2	52.12% IS_9b9ce816_00956	IS91	3911942	3912931	990
FJAT1458	IS_9b9ce816_00034	38.97% ISKpn25_aa1	No hit	ISL3	40783	42228	1446
FJAT1458	IS_9b9ce816_00792	100% ISRso21_aa2	No hit	ISL3	829347	830768	1422
FJAT1458	IS_9b9ce816_02037	62.5% ISKpn21_aa1	93.33% IS_9b9ce816_02038	ISNCY ssgr IS1202	2178495	2178094	402
FJAT1458	IS_9b9ce816_02038	72.27% ISKpn21_aa1	100% IS_9b9ce816_02052	ISNCY ssgr IS1202	2180124	2178649	1476
FJAT1458	IS_9b9ce816_02052	73.70% ISKpn21_aa1	100% IS_9b9ce816_02038	ISNCY ssgr IS1202	2189784	2188675	111
FJAT1458	IS_9b9ce816_00189	61.81% ISMpo10_aa3	72.38% IS_9b9ce816_02773	Tn3	205666	204278	1389
FJAT1458	IS_9b9ce816_01671	50% ISArsp6_aa1	No hit	Tn3	1762465	1763130	666
FJAT1458	IS_9b9ce816_02773	54.43% ISMpo10_aa3	95% IS_9b9ce816_02853	Tn3	2943261	2941882	138
FJAT1458	IS_9b9ce816_02853	51.83% ISMpo10_aa3	95% IS_9b9ce816_02773	Tn3	3023210	3021828	1383
EP1	IS_4f0ea434_01713	95.51% ISBma3_aa1	44.33% IS_4f0ea434_03567	IS110	1807441	1806236	1206
EP1	IS_4f0ea434_03567	88.75% ISBcen4_aa1	No hit	IS110 ssgr IS1111	3800437	3799418	102
EP1	IS_4f0ea434_00983	53.80% ISHpa1_aa1	No hit	IS1595 ssgr IS1016	1056134	1056913	780
EP1	IS_4f0ea434_02784	98.85% ISRso19_aa2	No hit	IS21	2993443	2992655	789
EP1	IS_4f0ea434_02785	98.52% ISRso19_aa1	No hit	IS21	2994456	2993440	1017
EP1	IS_4f0ea434_00916	100% ISRso11_aa1	No hit	IS3 ssgr IS150	987613	988146	534

EP1	IS_4f0ea434_00917	99.28% ISRso11_aa2	50.22% IS_4f0ea434_01222	IS3 ssgr IS150	988143	988979	837
EP1	IS_4f0ea434_00264	97.72% ISButh1_aa1	No hit	IS3 ssgr IS2	282649	28305	402
EP1	IS_4f0ea434_00265	93.52% ISButh1_aa2	40.22% IS_4f0ea434_00917	IS3 ssgr IS2	283047	283883	837
EP1	IS_4f0ea434_01222	88.78% IS222_aa2	98.65% IS_4f0ea434_02808	IS3 ssgr IS3	1293866	1294537	672
EP1	IS_4f0ea434_02807	88.23% IS222_aa1	No hit	IS3 ssgr IS3	3027040	3027348	309
EP1	IS_4f0ea434_02808	89.23% IS222_aa2	98.65% IS_4f0ea434_01222	IS3 ssgr IS3	3027531	3028202	672
EP1	IS_4f0ea434_00005	76% ISDet2_aa2	44.92% IS_4f0ea434_00917	IS3 ssgr IS407	7478	6654	825
EP1	IS_4f0ea434_00006	88.23% ISAtu5_aa1	91.66% IS_4f0ea434_03035	IS3 ssgr IS407	7777	7517	261
EP1	IS_4f0ea434_03035	83.13% ISSme1_aa1	91.66% IS_4f0ea434_00006	IS3 ssgr IS407	3247704	3248093	390
EP1	IS_4f0ea434_01210	86.77% ISAisp2_aa2	52.34% IS_4f0ea434_00917	IS3 ssgr IS51	1282497	1281706	792
EP1	IS_4f0ea434_01211	80.43% ISAisp2_aa1	No hit	IS3 ssgr IS51	1282772	1282494	279
EP1	IS_4f0ea434_00296	61.68% ISCro3_aa1	100% IS_4f0ea434_03666	IS4	319605	320933	1329
EP1	IS_4f0ea434_00484	61.68% ISCro3_aa1	100% IS_4f0ea434_02888	IS4	519816	518488	1329
EP1	IS_4f0ea434_01307	61.68% ISCro3_aa1	100% IS_4f0ea434_02888	IS4	1392583	1391255	1329
EP1	IS_4f0ea434_01567	61.68% ISCro3_aa1	100% IS_4f0ea434_03666	IS4	1663877	1665205	1329
EP1	IS_4f0ea434_01892	61.68% ISCro3_aa1	100% IS_4f0ea434_03666	IS4	1983268	1984596	1329
EP1	IS_4f0ea434_02469	61.68% ISCro3_aa1	100% IS_4f0ea434_02888	IS4	2666989	2665661	1329
EP1	IS_4f0ea434_02526	61.68% ISCro3_aa1	100% IS_4f0ea434_03666	IS4	2723396	2724724	1329
EP1	IS_4f0ea434_02888	61.68% ISCro3_aa1	100% IS_4f0ea434_02469	IS4	3104847	3103519	1329
EP1	IS_4f0ea434_03660	61.68% ISCro3_aa1	100% IS_4f0ea434_03666	IS4	3909263	3910591	1329
EP1	IS_4f0ea434_03666	61.68% ISCro3_aa1	100% IS_4f0ea434_03660	IS4	3917334	3918662	1329
EP1	IS_4f0ea434_00276	98.90% ISRso1_aa1	99.27% IS_4f0ea434_03474	IS5	294694	295518	825
EP1	IS_4f0ea434_00803	96.38% ISRso1_aa1	100% IS_4f0ea434_02741	IS5	875414	875716	303
EP1	IS_4f0ea434_00807	98.87% ISRso1_aa1	100% IS_4f0ea434_00276	IS5	879262	879816	555
EP1	IS_4f0ea434_01711	98.90% ISRso1_aa1	100% IS_4f0ea434_03474	IS5	1804548	1803724	825
EP1	IS_4f0ea434_02741	97.44% ISRso1_aa1	97.08% IS_4f0ea434_03474	IS5	2950816	2951640	825
EP1	IS_4f0ea434_03474	98.90% ISRso1_aa1	100% IS_4f0ea434_01711	IS5	3696226	3695402	825
EP1	IS_4f0ea434_00086	86.06% IS1421_aa1	100% IS_4f0ea434_03440	IS5 ssgr IS427	103702	103295	408
EP1	IS_4f0ea434_00283	86.06% IS1421_aa1	100% IS_4f0ea434_03440	IS5 ssgr IS427	301082	301489	408
EP1	IS_4f0ea434_01203	86.06% IS1421_aa1	100% IS_4f0ea434_03440	IS5 ssgr IS427	1275385	1275792	408
EP1	IS_4f0ea434_02276	86.06% IS1421_aa1	100% IS_4f0ea434_03440	IS5 ssgr IS427	2469761	2470168	408
EP1	IS_4f0ea434_02721	86.06% IS1421_aa1	100% IS_4f0ea434_03440	IS5 ssgr IS427	2923715	2923308	408
EP1	IS_4f0ea434_02795	86.06% IS1421_aa1	100% IS_4f0ea434_03440	IS5 ssgr IS427	3005026	3005433	408
EP1	IS_4f0ea434_03042	84.03% IS1421_aa1	98.48% IS_4f0ea434_03440	IS5 ssgr IS427	3254282	3254719	438
EP1	IS_4f0ea434_03440	86.06% IS1421_aa1	100% IS_4f0ea434_02795	IS5 ssgr IS427	3663695	3664102	408

EP1	IS_4f0ea434_00251	99.37% IS1405_aa1	100% IS_4f0ea434_02763	IS5 ssgr IS5	269928	270893	966
EP1	IS_4f0ea434_00253	85.93% ISAau3_aa1	80% IS_4f0ea434_02763	IS5 ssgr IS5	271263	271532	270
EP1	IS_4f0ea434_00332	99.37% IS1405_aa1	100% IS_4f0ea434_02763	IS5 ssgr IS5	364399	363434	966
EP1	IS_4f0ea434_00812	99.06% IS1405_aa1	99.68% IS_4f0ea434_02763	IS5 ssgr IS5	884915	88395	966
EP1	IS_4f0ea434_01607	99.37% IS1405_aa1	100% IS_4f0ea434_02763	IS5 ssgr IS5	1703612	1702647	966
EP1	IS_4f0ea434_01710	99.37% IS1405_aa1	100% IS_4f0ea434_02763	IS5 ssgr IS5	1803502	1802537	966
EP1	IS_4f0ea434_02520	99.37% IS1405_aa1	100% IS_4f0ea434_02763	IS5 ssgr IS5	2717576	2718541	966
EP1	IS_4f0ea434_02763	99.37% IS1405_aa1	100% IS_4f0ea434_02520	IS5 ssgr IS5	2972597	2973562	966
EP1	IS_4f0ea434_03027	94.20% IS1021_aa1	72.13% IS_4f0ea434_02763	IS5 ssgr IS5	3239736	3240722	987
EP1	IS_4f0ea434_00805	59.09% ISAeh1_aa2	100% IS_4f0ea434_00809	IS66	878315	876759	1557
EP1	IS_4f0ea434_00806	74.13% ISRm2_aa2	100% IS_4f0ea434_00808	IS66	878701	878348	354
EP1	IS_4f0ea434_00808	74.13% ISRm2_aa2	100% IS_4f0ea434_00806	IS66	880402	880755	354
EP1	IS_4f0ea434_00809	59.09% ISAeh1_aa2	100% IS_4f0ea434_00805	IS66	880788	882344	1557
EP1	IS_4f0ea434_02933	56.17% ISRsp1_aa4	No hit	IS66	3150072	3149524	549
EP1	IS_4f0ea434_01712	100% ISRso17_aa1	No hit	IS701	1806005	1804674	1332
EP1	IS_4f0ea434_00927	47.5% ISTha3_aa2	51.50% IS_4f0ea434_03639	IS91	999968	999042	927
EP1	IS_4f0ea434_02409	44.15% ISTha3_aa2	53.28% IS_4f0ea434_01664	IS91	2610342	2611448	1107
EP1	IS_4f0ea434_02932	50% ISWz1_aa1	40.59% IS_4f0ea434_02409	IS91	3148234	3149379	1146
EP1	IS_4f0ea434_03639	43.33% ISMno24_aa2	52.12% IS_4f0ea434_00927	IS91	3887948	3888937	990
EP1	IS_4f0ea434_00040	38.97% ISKpn25_aa1	No hit	ISL3	51571	53016	1446
EP1	IS_4f0ea434_02015	64.60% ISKpn21_aa1	No hit	ISNCY ssgr IS1202	2178979	2178578	402
EP1	IS_4f0ea434_01904	0% newcandidate	not_found	New_Family	1998995	1999150	156
EP1	IS_4f0ea434_00191	61.81% ISMpo10_aa3	96.96% IS_4f0ea434_00269	Tn3	213445	212057	1389
EP1	IS_4f0ea434_00269	61.81% ISMpo10_aa3	96.96% IS_4f0ea434_00191	Tn3	287535	286147	1389
EP1	IS_4f0ea434_01185	64.61% ISPa38_aa1	68.67% IS_4f0ea434_00269	Tn3	1262204	1262641	438
EP1	IS_4f0ea434_02643	54.43% ISMpo10_aa3	93.85% IS_4f0ea434_02789	Tn3	2846039	2844660	138
EP1	IS_4f0ea434_02723	54.43% ISMpo10_aa3	97.60% IS_4f0ea434_02789	Tn3	2925898	2924516	1383
EP1	IS_4f0ea434_02789	53.65% ISMpo10_aa3	97.60% IS_4f0ea434_02723	Tn3	2999330	2997948	1383
CQPS_1	CP016914_00545	66.66% ISBj4_aa1	No hit	IS110 ssgr IS1111	561941	560898	1044
CQPS_1	CP016914_02370	53.80% ISHpa1_aa1	No hit	IS1595 ssgr IS1016	2458996	2459775	780
CQPS_1	CP016914_00362	49.48% ISHvo5_aa1	44.65% CP016914_00480	IS1595 ssgr ISH4	366602	365814	789
CQPS_1	CP016914_00539	88.09% ISRme20_aa1	100% CP016914_00981	IS21	555457	556488	1032
CQPS_1	CP016914_00540	93.58% ISRme20_aa2	100% CP016914_00980	IS21	556488	557297	810
CQPS_1	CP016914_00980	93.58% ISRme20_aa2	100% CP016914_00540	IS21	990834	990025	810

CQPS_1	CP016914_00981	88.09% ISRme20_aa1	100% CP016914_00539	IS21	991865	990834	1032
CQPS_1	CP016914_02302	100% ISRso11_aa1	100% CP016914_02411	IS3 ssgr IS150	2390483	2391016	534
CQPS_1	CP016914_02303	99.28% ISRso11_aa2	100% CP016914_02410	IS3 ssgr IS150	2391013	2391849	837
CQPS_1	CP016914_02410	99.28% ISRso11_aa2	100% CP016914_02303	IS3 ssgr IS150	2503181	2502345	837
CQPS_1	CP016914_02411	100% ISRso11_aa1	100% CP016914_02302	IS3 ssgr IS150	2503711	2503178	534
CQPS_1	CP016914_02198	93.52% ISButh1_aa2	40.22% CP016914_02410	IS3 ssgr IS2	2287536	2286700	837
CQPS_1	CP016914_02199	97.72% ISButh1_aa1	No hit	IS3 ssgr IS2	2287934	2287533	402
CQPS_1	CP016914_00546	89.68% IS222_aa2	97.30% CP016914_02593	IS3 ssgr IS3	562236	562907	672
CQPS_1	CP016914_02592	82.81% ISPsy24_aa1	No hit	IS3 ssgr IS3	2681808	2682065	258
CQPS_1	CP016914_02593	88.78% IS222_aa2	97.30% CP016914_00546	IS3 ssgr IS3	2682298	2682969	672
CQPS_1	CP016914_00756	86.74% ISAtu5_aa1	79.51% CP016914_01410	IS3 ssgr IS407	760848	761237	390
CQPS_1	CP016914_00968	93.50% ISRso16_aa2	No hit	IS3 ssgr IS407	978972	978673	300
CQPS_1	CP016914_00972	100% ISRso14_aa1	100% CP016914_01410	IS3 ssgr IS407	982544	982807	264
CQPS_1	CP016914_00973	98.90% ISRso14_aa2	100% CP016914_01409	IS3 ssgr IS407	983113	983661	549
CQPS_1	CP016914_01251	100% ISRso14_aa1	100% CP016914_01410	IS3 ssgr IS407	1266604	1266867	264
CQPS_1	CP016914_01252	98.90% ISRso14_aa2	100% CP016914_01409	IS3 ssgr IS407	1267173	1267721	549
CQPS_1	CP016914_01409	98.90% ISRso14_aa2	100% CP016914_01252	IS3 ssgr IS407	1445803	1445255	549
CQPS_1	CP016914_01410	100% ISRso14_aa1	100% CP016914_01251	IS3 ssgr IS407	1446372	1446109	264
CQPS_1	CP016914_00544	78.04% ISAisp2_aa1	96.34% CP016914_02581	IS3 ssgr IS51	560394	560771	378
CQPS_1	CP016914_02580	89.34% ISAisp2_aa2	56.22% CP016914_00546	IS3 ssgr IS51	2670929	2670054	876
CQPS_1	CP016914_02581	81.52% ISAisp2_aa1	96.34% CP016914_00544	IS3 ssgr IS51	2671204	2670926	279
CQPS_1	CP016914_00072	61.68% ISCro3_aa1	100% CP016914_03215	IS4	7536	76688	1329
CQPS_1	CP016914_00182	61.68% ISCro3_aa1	100% CP016914_03215	IS4	183278	18195	1329
CQPS_1	CP016914_00239	61.68% ISCro3_aa1	100% CP016914_03215	IS4	238503	239831	1329
CQPS_1	CP016914_00446	61.68% ISCro3_aa1	100% CP016914_03215	IS4	461945	463273	1329
CQPS_1	CP016914_01408	72.27% ISCro3_aa1	100% CP016914_03215	IS4	1444852	1445223	372
CQPS_1	CP016914_01411	59.68% ISCro3_aa1	100% CP016914_03215	IS4	1446468	1447418	951
CQPS_1	CP016914_01417	61.68% ISCro3_aa1	100% CP016914_03215	IS4	1455404	1454076	1329
CQPS_1	CP016914_01669	61.68% ISCro3_aa1	100% CP016914_03215	IS4	1735839	1737167	1329
CQPS_1	CP016914_02178	61.68% ISCro3_aa1	100% CP016914_03215	IS4	2257159	2255831	1329
CQPS_1	CP016914_02680	61.68% ISCro3_aa1	100% CP016914_03215	IS4	2781902	2780574	1329
CQPS_1	CP016914_03215	61.68% ISCro3_aa1	100% CP016914_02680	IS4	3330028	3331356	1329
CQPS_1	CP016914_00376	97.08% ISRso1_aa1	96.71% CP016914_03085	IS5	389211	390035	825
CQPS_1	CP016914_01647	98.90% ISRso1_aa1	99.27% CP016914_03085	IS5	1709754	1710578	825
CQPS_1	CP016914_03085	98.90% ISRso1_aa1	99.27% CP016914_01647	IS5	3193161	3192337	825

CQPS_1	CP016914_00358	97.54% IS1421_aa1	86.25% CP016914_02594	IS5 ssgr IS427	361367	361771	405
CQPS_1	CP016914_00850	86.06% IS1421_aa1	100% CP016914_02594	IS5 ssgr IS427	857566	857159	408
CQPS_1	CP016914_01197	86.06% IS1421_aa1	100% CP016914_02594	IS5 ssgr IS427	1214667	1215074	408
CQPS_1	CP016914_01527	85.44% IS1421_aa3	99.12% CP016914_02594	IS5 ssgr IS427	1586880	1587683	804
CQPS_1	CP016914_02556	86.06% IS1421_aa1	100% CP016914_02594	IS5 ssgr IS427	2647585	2647178	408
CQPS_1	CP016914_02594	86.06% IS1421_aa1	100% CP016914_02556	IS5 ssgr IS427	2684584	2684177	408
CQPS_1	CP016914_00417	99.37% IS1405_aa1	100% CP016914_03618	IS5 ssgr IS5	43063	429665	966
CQPS_1	CP016914_00462	99.37% IS1405_aa1	100% CP016914_03618	IS5 ssgr IS5	480234	479269	966
CQPS_1	CP016914_00476	94.51% IS1021_aa1	99.39% CP016914_00748	IS5 ssgr IS5	490564	49155	987
CQPS_1	CP016914_00593	99.37% IS1405_aa1	100% CP016914_03618	IS5 ssgr IS5	605481	606446	966
CQPS_1	CP016914_00748	94.20% IS1021_aa1	99.39% CP016914_00476	IS5 ssgr IS5	75288	753866	987
CQPS_1	CP016914_01012	99.37% IS1405_aa1	100% CP016914_03618	IS5 ssgr IS5	1033170	1034135	966
CQPS_1	CP016914_01253	99.37% IS1405_aa1	100% CP016914_03618	IS5 ssgr IS5	1268329	1269294	966
CQPS_1	CP016914_01630	99.37% IS1405_aa1	100% CP016914_03618	IS5 ssgr IS5	1692941	1691976	966
CQPS_1	CP016914_01655	99.37% IS1405_aa1	100% CP016914_03618	IS5 ssgr IS5	1718040	1719005	966
CQPS_1	CP016914_02195	99.37% IS1405_aa1	100% CP016914_03618	IS5 ssgr IS5	2275750	2276715	966
CQPS_1	CP016914_03040	99.37% IS1405_aa1	100% CP016914_03618	IS5 ssgr IS5	3143888	3142923	966
CQPS_1	CP016914_03618	99.37% IS1405_aa1	100% CP016914_03040	IS5 ssgr IS5	3823208	3822243	966
CQPS_1	CP016914_02997	95.31% ISRso5_aa1	No hit	IS630	3104337	3103246	1092
CQPS_1	CP016914_00971	60.6% ISPpu13_aa2	100% CP016914_01174	IS66	982268	980682	1587
CQPS_1	CP016914_00974	72.88% ISRm2_aa2	100% CP016914_01175	IS66	983892	983707	186
CQPS_1	CP016914_00975	56.79% ISRm2_aa1	100% CP016914_01176	IS66	984365	983889	477
CQPS_1	CP016914_01174	60.6% ISPpu13_aa2	100% CP016914_00971	IS66	1193101	1191515	1587
CQPS_1	CP016914_01175	74.56% ISBmu30_aa1	100% CP016914_00974	IS66	1193487	1193134	354
CQPS_1	CP016914_01176	56.79% ISRm2_aa1	100% CP016914_00975	IS66	1193960	1193484	477
CQPS_1	CP016914_00475	100% ISRso17_aa1	100% CP016914_01249	IS701	489063	490394	1332
CQPS_1	CP016914_01249	100% ISRso17_aa1	100% CP016914_00475	IS701	1261906	1263237	1332
CQPS_1	CP016914_00468	45.61% ISTha3_aa2	100% CP016914_00504	IS91	485069	48405	102
CQPS_1	CP016914_00471	45.76% ISWz1_aa1	100% CP016914_00502	IS91	487211	485964	1248
CQPS_1	CP016914_00502	45.76% ISWz1_aa1	100% CP016914_00471	IS91	524369	525616	1248
CQPS_1	CP016914_00504	45.61% ISTha3_aa2	100% CP016914_00468	IS91	526512	527531	102
CQPS_1	CP016914_01386	43.33% ISMno24_aa2	52.12% CP016914_02313	IS91	1423573	1424562	990
CQPS_1	CP016914_02313	47.5% ISTha3_aa2	51.50% CP016914_01386	IS91	2402836	2401910	927
CQPS_1	CP016914_01453	40.10% ISKpn25_aa3	No hit	ISL3	1498863	1495573	3291
CQPS_1	CP016914_01455	44.86% ISKpn25_aa1	39.71% CP016914_01482	ISL3	1501828	1500209	162

CQPS_1	CP016914_01482	38.97% ISKpn25_aa1	40.43% CP016914_01455	ISL3	1535507	1536952	1446
CQPS_1	CP016914_03343	65.48% ISKpn21_aa1	No hit	ISNCY ssgr IS1202	3525701	3525309	393
CQPS_1	CP016914_03227	0% newcandidate	not_found	New_Family	3345760	3345915	156
CQPS_1	CP016914_00360	54.43% ISMpo10_aa3	97.17% CP016914_00478	Tn3	364306	362924	1383
CQPS_1	CP016914_00420	61.81% ISMpo10_aa3	97.18% CP016914_01640	Tn3	433002	43439	1389
CQPS_1	CP016914_00478	53.65% ISMpo10_aa3	97.17% CP016914_00360	Tn3	494445	493063	1383
CQPS_1	CP016914_00495	79.65% ISPsy30_aa1	No hit	Tn3	519445	519999	555
CQPS_1	CP016914_01640	61.81% ISMpo10_aa3	97.18% CP016914_00420	Tn3	1702595	1701207	1389
CQPS_1	CP016914_02536	61.87% ISPa38_aa1	54.90% CP016914_01640	Tn3	2627475	2628065	591
FJAT91	IS_45c6ab84_01713	95.51% ISBma3_aa1	44.33% IS_45c6ab84_03567	IS110	1807441	1806236	1206
FJAT91	IS_45c6ab84_03567	88.75% ISBcen4_aa1	No hit	IS110 ssgr IS1111	3800437	3799418	102
FJAT91	IS_45c6ab84_00983	53.80% ISHpa1_aa1	No hit	IS1595 ssgr IS1016	1056134	1056913	780
FJAT91	IS_45c6ab84_02784	98.85% ISRso19_aa2	No hit	IS21	2993443	2992655	789
FJAT91	IS_45c6ab84_02785	98.52% ISRso19_aa1	No hit	IS21	2994456	2993440	1017
FJAT91	IS_45c6ab84_00916	100% ISRso11_aa1	No hit	IS3 ssgr IS150	987613	988146	534
FJAT91	IS_45c6ab84_00917	99.28% ISRso11_aa2	50.22% IS_45c6ab84_01222	IS3 ssgr IS150	988143	988979	837
FJAT91	IS_45c6ab84_00264	97.72% ISButh1_aa1	No hit	IS3 ssgr IS2	282649	28305	402
FJAT91	IS_45c6ab84_00265	93.52% ISButh1_aa2	40.22% IS_45c6ab84_00917	IS3 ssgr IS2	283047	283883	837
FJAT91	IS_45c6ab84_01222	88.78% IS222_aa2	98.65% IS_45c6ab84_02808	IS3 ssgr IS3	1293866	1294537	672
FJAT91	IS_45c6ab84_02807	88.23% IS222_aa1	No hit	IS3 ssgr IS3	3027040	3027348	309
FJAT91	IS_45c6ab84_02808	89.23% IS222_aa2	98.65% IS_45c6ab84_01222	IS3 ssgr IS3	3027531	3028202	672
FJAT91	IS_45c6ab84_00005	76% ISDet2_aa2	44.92% IS_45c6ab84_00917	IS3 ssgr IS407	7478	6654	825
FJAT91	IS_45c6ab84_00006	88.23% ISAtu5_aa1	91.66% IS_45c6ab84_03035	IS3 ssgr IS407	7777	7517	261
FJAT91	IS_45c6ab84_03035	83.13% ISSme1_aa1	91.66% IS_45c6ab84_00006	IS3 ssgr IS407	3247704	3248093	390
FJAT91	IS_45c6ab84_01210	86.77% ISAisp2_aa2	52.34% IS_45c6ab84_00917	IS3 ssgr IS51	1282497	1281706	792
FJAT91	IS_45c6ab84_01211	80.43% ISAisp2_aa1	No hit	IS3 ssgr IS51	1282772	1282494	279
FJAT91	IS_45c6ab84_00296	61.68% ISCro3_aa1	100% IS_45c6ab84_03666	IS4	319605	320933	1329
FJAT91	IS_45c6ab84_00484	61.68% ISCro3_aa1	100% IS_45c6ab84_02888	IS4	519816	518488	1329
FJAT91	IS_45c6ab84_01307	61.68% ISCro3_aa1	100% IS_45c6ab84_02888	IS4	1392583	1391255	1329
FJAT91	IS_45c6ab84_01567	61.68% ISCro3_aa1	100% IS_45c6ab84_03666	IS4	1663877	1665205	1329
FJAT91	IS_45c6ab84_01892	61.68% ISCro3_aa1	100% IS_45c6ab84_03666	IS4	1983268	1984596	1329
FJAT91	IS_45c6ab84_02469	61.68% ISCro3_aa1	100% IS_45c6ab84_02888	IS4	2666989	2665661	1329
FJAT91	IS_45c6ab84_02526	61.68% ISCro3_aa1	100% IS_45c6ab84_03666	IS4	2723396	2724724	1329
FJAT91	IS_45c6ab84_02888	61.68% ISCro3_aa1	100% IS_45c6ab84_02469	IS4	3104847	3103519	1329

FJAT91	IS_45c6ab84_03660	61.68% ISCro3_aa1	100% IS_45c6ab84_03666	IS4	3909263	3910591	1329
FJAT91	IS_45c6ab84_03666	61.68% ISCro3_aa1	100% IS_45c6ab84_03660	IS4	3917334	3918662	1329
FJAT91	IS_45c6ab84_00276	98.90% ISRso1_aa1	99.27% IS_45c6ab84_03474	IS5	294694	295518	825
FJAT91	IS_45c6ab84_00803	96.38% ISRso1_aa1	100% IS_45c6ab84_02741	IS5	875414	875716	303
FJAT91	IS_45c6ab84_00807	98.87% ISRso1_aa1	100% IS_45c6ab84_00276	IS5	879262	879816	555
FJAT91	IS_45c6ab84_01711	98.90% ISRso1_aa1	100% IS_45c6ab84_03474	IS5	1804548	1803724	825
FJAT91	IS_45c6ab84_02741	97.44% ISRso1_aa1	97.08% IS_45c6ab84_03474	IS5	2950816	2951640	825
FJAT91	IS_45c6ab84_03474	98.90% ISRso1_aa1	100% IS_45c6ab84_01711	IS5	3696226	3695402	825
FJAT91	IS_45c6ab84_00086	86.06% IS1421_aa1	100% IS_45c6ab84_03440	IS5 ssgr IS427	103702	103295	408
FJAT91	IS_45c6ab84_00283	86.06% IS1421_aa1	100% IS_45c6ab84_03440	IS5 ssgr IS427	301082	301489	408
FJAT91	IS_45c6ab84_01203	86.06% IS1421_aa1	100% IS_45c6ab84_03440	IS5 ssgr IS427	1275385	1275792	408
FJAT91	IS_45c6ab84_02276	86.06% IS1421_aa1	100% IS_45c6ab84_03440	IS5 ssgr IS427	2469761	2470168	408
FJAT91	IS_45c6ab84_02721	86.06% IS1421_aa1	100% IS_45c6ab84_03440	IS5 ssgr IS427	2923715	2923308	408
FJAT91	IS_45c6ab84_02795	86.06% IS1421_aa1	100% IS_45c6ab84_03440	IS5 ssgr IS427	3005026	3005433	408
FJAT91	IS_45c6ab84_03042	84.03% IS1421_aa1	98.48% IS_45c6ab84_03440	IS5 ssgr IS427	3254282	3254719	438
FJAT91	IS_45c6ab84_03440	86.06% IS1421_aa1	100% IS_45c6ab84_02795	IS5 ssgr IS427	3663695	3664102	408
FJAT91	IS_45c6ab84_00251	99.37% IS1405_aa1	100% IS_45c6ab84_02763	IS5 ssgr IS5	269928	270893	966
FJAT91	IS_45c6ab84_00253	85.93% ISAau3_aa1	80% IS_45c6ab84_02763	IS5 ssgr IS5	271263	271532	270
FJAT91	IS_45c6ab84_00332	99.37% IS1405_aa1	100% IS_45c6ab84_02763	IS5 ssgr IS5	364399	363434	966
FJAT91	IS_45c6ab84_00812	99.06% IS1405_aa1	99.68% IS_45c6ab84_02763	IS5 ssgr IS5	884915	88395	966
FJAT91	IS_45c6ab84_01607	99.37% IS1405_aa1	100% IS_45c6ab84_02763	IS5 ssgr IS5	1703612	1702647	966
FJAT91	IS_45c6ab84_01710	99.37% IS1405_aa1	100% IS_45c6ab84_02763	IS5 ssgr IS5	1803502	1802537	966
FJAT91	IS_45c6ab84_02520	99.37% IS1405_aa1	100% IS_45c6ab84_02763	IS5 ssgr IS5	2717576	2718541	966
FJAT91	IS_45c6ab84_02763	99.37% IS1405_aa1	100% IS_45c6ab84_02520	IS5 ssgr IS5	2972597	2973562	966
FJAT91	IS_45c6ab84_03027	94.20% IS1021_aa1	72.13% IS_45c6ab84_02763	IS5 ssgr IS5	3239736	3240722	987
FJAT91	IS_45c6ab84_00805	59.09% ISAeh1_aa2	100% IS_45c6ab84_00809	IS66	878315	876759	1557
FJAT91	IS_45c6ab84_00806	74.13% ISRm2_aa2	100% IS_45c6ab84_00808	IS66	878701	878348	354
FJAT91	IS_45c6ab84_00808	74.13% ISRm2_aa2	100% IS_45c6ab84_00806	IS66	880402	880755	354
FJAT91	IS_45c6ab84_00809	59.09% ISAeh1_aa2	100% IS_45c6ab84_00805	IS66	880788	882344	1557
FJAT91	IS_45c6ab84_02933	56.17% ISRsp1_aa4	No hit	IS66	3150072	3149524	549
FJAT91	IS_45c6ab84_01712	100% ISRso17_aa1	No hit	IS701	1806005	1804674	1332
FJAT91	IS_45c6ab84_00927	47.5% ISTha3_aa2	51.50% IS_45c6ab84_03639	IS91	999968	999042	927
FJAT91	IS_45c6ab84_02409	44.15% ISTha3_aa2	53.28% IS_45c6ab84_01664	IS91	2610342	2611448	1107
FJAT91	IS_45c6ab84_02932	50% ISWz1_aa1	40.59% IS_45c6ab84_02409	IS91	3148234	3149379	1146
FJAT91	IS_45c6ab84_03639	43.33% ISMno24_aa2	52.12% IS_45c6ab84_00927	IS91	3887948	3888937	990

FJAT91	IS_45c6ab84_00040	38.97% ISKpn25_aa1	No hit	ISL3	51571	53016	1446
FJAT91	IS_45c6ab84_02015	64.60% ISKpn21_aa1	No hit	ISNCY ssgr IS1202	2178979	2178578	402
FJAT91	IS_45c6ab84_01904	0% newcandidate	not_found	New_Family	1998995	1999150	156
FJAT91	IS_45c6ab84_00191	61.81% ISMpo10_aa3	96.96% IS_45c6ab84_00269	Tn3	213445	212057	1389
FJAT91	IS_45c6ab84_00269	61.81% ISMpo10_aa3	96.96% IS_45c6ab84_00191	Tn3	287535	286147	1389
FJAT91	IS_45c6ab84_01185	64.61% ISPa38_aa1	68.67% IS_45c6ab84_00269	Tn3	1262204	1262641	438
FJAT91	IS_45c6ab84_02643	54.43% ISMpo10_aa3	93.85% IS_45c6ab84_02789	Tn3	2846039	2844660	138
FJAT91	IS_45c6ab84_02723	54.43% ISMpo10_aa3	97.60% IS_45c6ab84_02789	Tn3	2925898	2924516	1383
FJAT91	IS_45c6ab84_02789	53.65% ISMpo10_aa3	97.60% IS_45c6ab84_02723	Tn3	2999330	2997948	1383
FQY_4	IS_9e96eacd_00633	95.76% ISBma3_aa1	100% IS_9e96eacd_02839	IS110	662273	663478	1206
FQY_4	IS_9e96eacd_02839	95.76% ISBma3_aa1	100% IS_9e96eacd_00633	IS110	3080268	3081473	1206
FQY_4	IS_9e96eacd_00110	82.67% IS1383_aa1	100% IS_9e96eacd_03020	IS110 ssgr IS1111	127125	126124	1002
FQY_4	IS_9e96eacd_00130	88.75% ISBcen4_aa1	72.72% IS_9e96eacd_03020	IS110 ssgr IS1111	149455	150474	102
FQY_4	IS_9e96eacd_00189	82.67% IS1383_aa1	100% IS_9e96eacd_03020	IS110 ssgr IS1111	20951	208509	1002
FQY_4	IS_9e96eacd_03020	82.67% IS1383_aa1	100% IS_9e96eacd_00189	IS110 ssgr IS1111	3263234	3262233	1002
FQY_4	IS_9e96eacd_00707	99.64% ISRso11_aa2	100% IS_9e96eacd_03121	IS3 ssgr IS150	3263234	3262233	837
FQY_4	IS_9e96eacd_00708	100% ISRso11_aa1	100% IS_9e96eacd_03122	IS3 ssgr IS150	737451	736918	534
FQY_4	IS_9e96eacd_01463	100% ISRso11_aa1	100% IS_9e96eacd_03122	IS3 ssgr IS150	1552142	1552675	534
FQY_4	IS_9e96eacd_01464	99.64% ISRso11_aa2	100% IS_9e96eacd_03121	IS3 ssgr IS150	1552672	1553508	837
FQY_4	IS_9e96eacd_03121	99.64% ISRso11_aa2	100% IS_9e96eacd_01464	IS3 ssgr IS150	3349134	3348298	837
FQY_4	IS_9e96eacd_03122	100% ISRso11_aa1	100% IS_9e96eacd_01463	IS3 ssgr IS150	3349664	3349131	534
FQY_4	IS_9e96eacd_02251	89.68% IS222_aa2	56.22% IS_9e96eacd_02297	IS3 ssgr IS3	2455126	2454455	672
FQY_4	IS_9e96eacd_02252	87.25% IS222_aa1	59.77% IS_9e96eacd_02296	IS3 ssgr IS3	2455617	2455309	309
FQY_4	IS_9e96eacd_02263	80.43% ISAisp2_aa1	96.73% IS_9e96eacd_02296	IS3 ssgr IS51	2466383	2466661	279
FQY_4	IS_9e96eacd_02264	86.38% ISAisp2_aa2	94.94% IS_9e96eacd_02297	IS3 ssgr IS51	2466658	2467449	792
FQY_4	IS_9e96eacd_02296	81.52% ISAisp2_aa1	96.73% IS_9e96eacd_02263	IS3 ssgr IS51	2501933	2502211	279
FQY_4	IS_9e96eacd_02297	89.34% ISAisp2_aa2	94.94% IS_9e96eacd_02264	IS3 ssgr IS51	2502208	2503083	876
FQY_4	IS_9e96eacd_00263	65.95% IS1382_aa1	100% IS_9e96eacd_00821	IS30	285642	286661	102
FQY_4	IS_9e96eacd_00821	65.95% IS1382_aa1	100% IS_9e96eacd_00263	IS30	848913	847894	102
FQY_4	IS_9e96eacd_00303	61.68% ISCro3_aa1	100% IS_9e96eacd_02915	IS4	329942	328614	1329
FQY_4	IS_9e96eacd_00739	61.68% ISCro3_aa1	100% IS_9e96eacd_02915	IS4	767174	765846	1329
FQY_4	IS_9e96eacd_00880	61.68% ISCro3_aa1	100% IS_9e96eacd_03155	IS4	923078	924406	1329
FQY_4	IS_9e96eacd_00915	61.68% ISCro3_aa1	100% IS_9e96eacd_03155	IS4	956846	955518	1329
FQY_4	IS_9e96eacd_00950	61.68% ISCro3_aa1	100% IS_9e96eacd_03155	IS4	99327	991942	1329

FQY_4	IS_9e96eacd_00958	61.68% ISCro3_aa1	100% IS_9e96eacd_03155	IS4	1000476	1001804	1329
FQY_4	IS_9e96eacd_00976	61.68% ISCro3_aa1	100% IS_9e96eacd_02915	IS4	1022722	1021394	1329
FQY_4	IS_9e96eacd_00978	61.68% ISCro3_aa1	100% IS_9e96eacd_03155	IS4	1023939	1025267	1329
FQY_4	IS_9e96eacd_01020	61.68% ISCro3_aa1	100% IS_9e96eacd_02915	IS4	1067264	1068592	1329
FQY_4	IS_9e96eacd_01649	61.44% ISCro3_aa1	99.77% IS_9e96eacd_03155	IS4	1811460	1810132	1329
FQY_4	IS_9e96eacd_02167	61.65% ISCro3_aa1	96.32% IS_9e96eacd_01649	IS4	2356668	2357993	1326
FQY_4	IS_9e96eacd_02606	61.68% ISCro3_aa1	100% IS_9e96eacd_03155	IS4	2823501	2822173	1329
FQY_4	IS_9e96eacd_02915	61.68% ISCro3_aa1	100% IS_9e96eacd_01020	IS4	3156527	3155199	1329
FQY_4	IS_9e96eacd_03155	61.68% ISCro3_aa1	100% IS_9e96eacd_02606	IS4	3391538	3390210	1329
FQY_4	IS_9e96eacd_01103	76.86% ISAzo5_aa1	100% IS_9e96eacd_03167	IS4 ssgr IS50	1149313	1150620	1308
FQY_4	IS_9e96eacd_01788	76.86% ISAzo5_aa1	100% IS_9e96eacd_03167	IS4 ssgr IS50	1954220	1952913	1308
FQY_4	IS_9e96eacd_02316	76.86% ISAzo5_aa1	100% IS_9e96eacd_03167	IS4 ssgr IS50	2523699	2525006	1308
FQY_4	IS_9e96eacd_03167	76.86% ISAzo5_aa1	100% IS_9e96eacd_02316	IS4 ssgr IS50	3408930	3410237	1308
FQY_4	IS_9e96eacd_00836	97.44% ISRso1_aa1	74.87% IS_9e96eacd_01800	IS5	868085	867261	825
FQY_4	IS_9e96eacd_00847	97.29% ISRso1_aa1	95.94% IS_9e96eacd_00836	IS5	876011	876283	273
FQY_4	IS_9e96eacd_01800	96.29% ISRso1_aa1	74.87% IS_9e96eacd_00836	IS5	1969502	1968012	1491
FQY_4	IS_9e96eacd_00111	100% IS1421_aa1	100% IS_9e96eacd_03439	IS5 ssgr IS427	128062	127658	405
FQY_4	IS_9e96eacd_00190	100% IS1421_aa1	100% IS_9e96eacd_03439	IS5 ssgr IS427	210447	210043	405
FQY_4	IS_9e96eacd_03021	100% IS1421_aa1	100% IS_9e96eacd_03439	IS5 ssgr IS427	3264171	3263767	405
FQY_4	IS_9e96eacd_03439	100% IS1421_aa1	100% IS_9e96eacd_03021	IS5 ssgr IS427	3691957	3692361	405
FQY_4	IS_9e96eacd_00224	99.37% IS1405_aa1	100% IS_9e96eacd_03061	IS5 ssgr IS5	240984	241949	966
FQY_4	IS_9e96eacd_00259	99.06% IS1405_aa1	100% IS_9e96eacd_02846	IS5 ssgr IS5	278133	277168	966
FQY_4	IS_9e96eacd_00278	94.51% IS1021_aa1	100% IS_9e96eacd_02831	IS5 ssgr IS5	304019	303033	987
FQY_4	IS_9e96eacd_00820	99.06% IS1405_aa1	100% IS_9e96eacd_02846	IS5 ssgr IS5	846894	845929	966
FQY_4	IS_9e96eacd_00838	99.06% IS1405_aa1	100% IS_9e96eacd_02846	IS5 ssgr IS5	869669	870634	966
FQY_4	IS_9e96eacd_01855	99.06% IS1405_aa1	100% IS_9e96eacd_02846	IS5 ssgr IS5	2031325	2032290	966
FQY_4	IS_9e96eacd_02068	99.06% IS1405_aa1	100% IS_9e96eacd_02846	IS5 ssgr IS5	2253890	2254855	966
FQY_4	IS_9e96eacd_02433	94.63% IS1021_aa1	100% IS_9e96eacd_02831	IS5 ssgr IS5	2648040	2647219	822
FQY_4	IS_9e96eacd_02573	94.51% IS1021_aa1	100% IS_9e96eacd_02831	IS5 ssgr IS5	2789434	2788448	987
FQY_4	IS_9e96eacd_02591	94.51% IS1021_aa1	100% IS_9e96eacd_02831	IS5 ssgr IS5	2804251	2805237	987
FQY_4	IS_9e96eacd_02831	94.51% IS1021_aa1	100% IS_9e96eacd_02591	IS5 ssgr IS5	3071711	3070725	987
FQY_4	IS_9e96eacd_02846	99.06% IS1405_aa1	100% IS_9e96eacd_02068	IS5 ssgr IS5	3086712	3085747	966
FQY_4	IS_9e96eacd_03061	99.37% IS1405_aa1	100% IS_9e96eacd_00224	IS5 ssgr IS5	3291933	3292898	966
FQY_4	IS_9e96eacd_03432	91.66% IS1021_aa1	100% IS_9e96eacd_02831	IS5 ssgr IS5	3686124	3685426	699
FQY_4	IS_9e96eacd_01361	85.13% ISAzo9_aa2	No hit	IS630	1435833	1436960	1128

FQY_4	IS_9e96eacd_01107	56.95% ISRm2_aa1	100% IS_9e96eacd_03436	IS66	1152442	1152924	483
FQY_4	IS_9e96eacd_01108	73.27% ISRm2_aa2	100% IS_9e96eacd_03435	IS66	1152921	1153274	354
FQY_4	IS_9e96eacd_01109	59.58% ISAeh1_aa2	100% IS_9e96eacd_02560	IS66	1156402	1154222	2181
FQY_4	IS_9e96eacd_01110	73.27% ISRm2_aa2	100% IS_9e96eacd_03435	IS66	1156788	1156435	354
FQY_4	IS_9e96eacd_01111	56.95% ISRm2_aa1	100% IS_9e96eacd_03436	IS66	1157267	1156785	483
FQY_4	IS_9e96eacd_01794	56.95% ISRm2_aa1	100% IS_9e96eacd_03436	IS66	1960272	1960754	483
FQY_4	IS_9e96eacd_01795	73.27% ISRm2_aa2	100% IS_9e96eacd_03435	IS66	1960751	1961104	354
FQY_4	IS_9e96eacd_01796	61.53% ISPpu13_aa2	100% IS_9e96eacd_01963	IS66	1961137	1962693	1557
FQY_4	IS_9e96eacd_01961	58.27% ISRm2_aa1	99.37% IS_9e96eacd_03436	IS66	2140844	2141326	483
FQY_4	IS_9e96eacd_01962	73.27% ISRm2_aa2	100% IS_9e96eacd_03435	IS66	2141323	2141676	354
FQY_4	IS_9e96eacd_01963	61.53% ISPpu13_aa2	100% IS_9e96eacd_01796	IS66	2141709	2143265	1557
FQY_4	IS_9e96eacd_02558	56.95% ISRm2_aa1	100% IS_9e96eacd_03436	IS66	2777331	2777813	483
FQY_4	IS_9e96eacd_02559	73.27% ISRm2_aa2	100% IS_9e96eacd_03435	IS66	2777810	2778163	354
FQY_4	IS_9e96eacd_02560	59.58% ISAeh1_aa2	100% IS_9e96eacd_03434	IS66	2778196	2779809	1614
FQY_4	IS_9e96eacd_02818	59.14% ISAeh1_aa2	100% IS_9e96eacd_01963	IS66	3057425	3055656	177
FQY_4	IS_9e96eacd_02819	73.27% ISRm2_aa2	100% IS_9e96eacd_03435	IS66	3057811	3057458	354
FQY_4	IS_9e96eacd_02820	56.95% ISRm2_aa1	100% IS_9e96eacd_03436	IS66	3058290	3057808	483
FQY_4	IS_9e96eacd_03217	60.98% ISPpu13_aa2	100% IS_9e96eacd_01963	IS66	3463096	3461504	1593
FQY_4	IS_9e96eacd_03218	73.27% ISRm2_aa2	100% IS_9e96eacd_03435	IS66	3463482	3463129	354
FQY_4	IS_9e96eacd_03219	56.95% ISRm2_aa1	100% IS_9e96eacd_03436	IS66	3463961	3463479	483
FQY_4	IS_9e96eacd_03434	59.58% ISAeh1_aa2	100% IS_9e96eacd_02560	IS66	3688786	3687149	1638
FQY_4	IS_9e96eacd_03435	73.27% ISRm2_aa2	100% IS_9e96eacd_03218	IS66	3689172	3688819	354
FQY_4	IS_9e96eacd_03436	56.95% ISRm2_aa1	100% IS_9e96eacd_03219	IS66	3689651	3689169	483
FQY_4	IS_9e96eacd_00057	43.33% ISMno24_aa2	52.12% IS_9e96eacd_02460	IS91	62416	61427	990
FQY_4	IS_9e96eacd_01779	45.41% ISTha3_aa2	53.80% IS_9e96eacd_01792	IS91	1943876	1944931	1056
FQY_4	IS_9e96eacd_02460	47.5% ISTha3_aa2	51.50% IS_9e96eacd_00057	IS91	2676081	2677007	927
FQY_4	IS_9e96eacd_03410	38.97% ISKpn25_aa1	No hit	ISL3	3662678	3661233	1446
FQY_4	IS_9e96eacd_03449	46.61% ISPa4_aa1	No hit	ISNCY	3706879	3708969	2091
FQY_4	IS_9e96eacd_01526	72.27% ISKpn21_aa1	95% IS_9e96eacd_01527	ISNCY ssgr IS1202	1617415	1618890	1476
FQY_4	IS_9e96eacd_01527	63.33% ISKpn21_aa1	95% IS_9e96eacd_01526	ISNCY ssgr IS1202	1619044	1619445	402
FQY_4	IS_9e96eacd_00854	54.43% ISMpo10_aa3	73.79% IS_9e96eacd_03258	Tn3	892361	893743	1383
FQY_4	IS_9e96eacd_01785	75.93% ISSba14_aa1	47.23% IS_9e96eacd_00854	Tn3	1950689	1950111	579
FQY_4	IS_9e96eacd_03258	61.81% ISMpo10_aa3	73.79% IS_9e96eacd_00854	Tn3	3501609	3502997	1389
RSCM	CP025985_00201	61.67% ISMno14_aa1	100% CP025985_03174	IS110 ssgr IS1111	211094	210081	1014

RSCM	CP025985_00636	82.67% IS1383_aa1	100% CP025985_03449	IS110 ssgr IS1111	643784	644785	1002
RSCM	CP025985_01258	61.67% ISMno14_aa1	100% CP025985_03174	IS110 ssgr IS1111	1314966	1313953	1014
RSCM	CP025985_01791	61.67% ISMno14_aa1	100% CP025985_03174	IS110 ssgr IS1111	1904278	1903265	1014
RSCM	CP025985_02396	61.67% ISMno14_aa1	100% CP025985_03174	IS110 ssgr IS1111	2572473	2573486	1014
RSCM	CP025985_02560	82.67% IS1383_aa1	100% CP025985_03449	IS110 ssgr IS1111	2746117	2745116	1002
RSCM	CP025985_02888	61.67% ISMno14_aa1	100% CP025985_03174	IS110 ssgr IS1111	3078347	3079360	1014
RSCM	CP025985_03017	61.67% ISMno14_aa1	100% CP025985_03174	IS110 ssgr IS1111	3209883	3210896	1014
RSCM	CP025985_03174	61.67% ISMno14_aa1	100% CP025985_03017	IS110 ssgr IS1111	3371686	3372699	1014
RSCM	CP025985_03449	82.67% IS1383_aa1	100% CP025985_02560	IS110 ssgr IS1111	3643823	3644824	1002
RSCM	CP025985_00604	83.02% ISBusp4_aa1	100% CP025985_03451	IS1182	616073	614622	1452
RSCM	CP025985_00872	83.02% ISBusp4_aa1	100% CP025985_03451	IS1182	905326	903875	1452
RSCM	CP025985_01454	83.02% ISBusp4_aa1	100% CP025985_03451	IS1182	1541223	1539772	1452
RSCM	CP025985_01702	81.45% ISBusp4_aa1	100% CP025985_03451	IS1182	1805101	1804274	828
RSCM	CP025985_02020	83.02% ISBusp4_aa1	100% CP025985_03451	IS1182	2198958	2197507	1452
RSCM	CP025985_02355	83.02% ISBusp4_aa1	100% CP025985_03451	IS1182	2528784	2530235	1452
RSCM	CP025985_02874	83.02% ISBusp4_aa1	100% CP025985_03451	IS1182	3067708	3069159	1452
RSCM	CP025985_03117	83.02% ISBusp4_aa1	100% CP025985_03451	IS1182	3314494	3315945	1452
RSCM	CP025985_03149	83.02% ISBusp4_aa1	100% CP025985_03451	IS1182	3343637	3345088	1452
RSCM	CP025985_03451	83.02% ISBusp4_aa1	100% CP025985_03149	IS1182	3646197	3647648	1452
RSCM	CP025985_02710	53.80% ISHpa1_aa1	No hit	IS1595 ssgr IS1016	2900175	2899396	780
RSCM	CP025985_01247	49.48% ISHvo5_aa1	No hit	IS1595 ssgr ISH4	1303440	1304228	789
RSCM	CP025985_00274	95.91% ISRso7_aa1	99.75% CP025985_02943	IS256	28276	28401	1251
RSCM	CP025985_00397	96.15% ISRso7_aa1	100% CP025985_02943	IS256	418858	417608	1251
RSCM	CP025985_01039	96.14% ISRso7_aa1	100% CP025985_02966	IS256	1089426	1088257	117
RSCM	CP025985_02047	96.15% ISRso7_aa1	100% CP025985_02943	IS256	2230550	2229300	1251
RSCM	CP025985_02943	96.15% ISRso7_aa1	100% CP025985_02047	IS256	3133431	3134681	1251
RSCM	CP025985_02966	96.14% ISRso7_aa1	100% CP025985_01039	IS256	3158493	3157324	117
RSCM	CP025985_01051	100% ISRso11_aa1	100% CP025985_03187	IS3 ssgr IS150	1099416	1099949	534
RSCM	CP025985_01052	99.28% ISRso11_aa2	100% CP025985_03186	IS3 ssgr IS150	1099946	1100782	837
RSCM	CP025985_01804	99.28% ISRso11_aa2	100% CP025985_03186	IS3 ssgr IS150	1916245	1915409	837
RSCM	CP025985_01805	99.43% ISRso11_aa1	99.43% CP025985_03187	IS3 ssgr IS150	1916775	1916242	534
RSCM	CP025985_02779	99.28% ISRso11_aa2	100% CP025985_03186	IS3 ssgr IS150	2971495	2970659	837
RSCM	CP025985_02780	100% ISRso11_aa1	100% CP025985_03187	IS3 ssgr IS150	2972025	2971492	534
RSCM	CP025985_03186	99.28% ISRso11_aa2	100% CP025985_02779	IS3 ssgr IS150	3386115	3385279	837
RSCM	CP025985_03187	100% ISRso11_aa1	100% CP025985_02780	IS3 ssgr IS150	3386645	3386112	534

RSCM	CP025985_01257	94.87% ISButh1_aa2	100% CP025985_03175	IS3 ssgr IS2	1313453	1313100	354
RSCM	CP025985_01259	89.33% ISButh1_aa2	100% CP025985_03173	IS3 ssgr IS2	1315233	1314997	237
RSCM	CP025985_01260	97.72% ISButh1_aa1	100% CP025985_03172	IS3 ssgr IS2	1315631	1315230	402
RSCM	CP025985_02394	97.72% ISButh1_aa1	100% CP025985_03172	IS3 ssgr IS2	2571808	2572209	402
RSCM	CP025985_02395	89.33% ISButh1_aa2	100% CP025985_03173	IS3 ssgr IS2	2572206	2572442	237
RSCM	CP025985_02397	94.87% ISButh1_aa2	100% CP025985_03175	IS3 ssgr IS2	2573986	2574339	354
RSCM	CP025985_02886	97.72% ISButh1_aa1	100% CP025985_03172	IS3 ssgr IS2	3077682	3078083	402
RSCM	CP025985_02887	89.33% ISButh1_aa2	100% CP025985_03173	IS3 ssgr IS2	3078080	3078316	237
RSCM	CP025985_02894	94.87% ISButh1_aa2	100% CP025985_03175	IS3 ssgr IS2	3083575	3083928	354
RSCM	CP025985_03172	97.72% ISButh1_aa1	100% CP025985_02886	IS3 ssgr IS2	3371021	3371422	402
RSCM	CP025985_03173	89.33% ISButh1_aa2	100% CP025985_02887	IS3 ssgr IS2	3371419	3371655	237
RSCM	CP025985_03175	94.87% ISButh1_aa2	100% CP025985_02894	IS3 ssgr IS2	3373199	3373552	354
RSCM	CP025985_01165	89.23% IS222_aa2	98.65% CP025985_02524	IS3 ssgr IS3	1207705	1207034	672
RSCM	CP025985_01166	88.23% IS222_aa1	100% CP025985_02525	IS3 ssgr IS3	1208196	1207888	309
RSCM	CP025985_02524	88.78% IS222_aa2	98.65% CP025985_01165	IS3 ssgr IS3	2713587	2712916	672
RSCM	CP025985_02525	82.81% ISPsy24_aa1	100% CP025985_01166	IS3 ssgr IS3	2714077	2713820	258
RSCM	CP025985_03019	82.60% ISHar2_aa2	65.93% CP025985_03180	IS3 ssgr IS3	3212351	3211770	582
RSCM	CP025985_00011	100% ISRso14_aa2	100% CP025985_01189	IS3 ssgr IS407	14827	14	828
RSCM	CP025985_00012	100% ISRso14_aa1	100% CP025985_03016	IS3 ssgr IS407	15117	14854	264
RSCM	CP025985_00200	100% ISRso14_aa2	100% CP025985_01189	IS3 ssgr IS407	209767	209219	549
RSCM	CP025985_00202	100% ISRso14_aa1	100% CP025985_03016	IS3 ssgr IS407	211633	21137	264
RSCM	CP025985_00329	100% ISRso14_aa2	100% CP025985_01189	IS3 ssgr IS407	351438	350611	828
RSCM	CP025985_00330	100% ISRso14_aa1	100% CP025985_03016	IS3 ssgr IS407	351728	351465	264
RSCM	CP025985_00600	100% ISRso14_aa2	100% CP025985_01189	IS3 ssgr IS407	611916	611089	828
RSCM	CP025985_00601	100% ISRso14_aa1	100% CP025985_03016	IS3 ssgr IS407	612206	611943	264
RSCM	CP025985_01188	100% ISRso14_aa1	100% CP025985_03016	IS3 ssgr IS407	1238090	1238293	204
RSCM	CP025985_01189	100% ISRso14_aa2	100% CP025985_00600	IS3 ssgr IS407	1238320	1239147	828
RSCM	CP025985_01792	100% ISRso14_aa1	100% CP025985_03016	IS3 ssgr IS407	1904817	1904554	264
RSCM	CP025985_03016	100% ISRso14_aa1	100% CP025985_01792	IS3 ssgr IS407	3209344	3209607	264
RSCM	CP025985_03018	100% ISRso14_aa2	100% CP025985_01189	IS3 ssgr IS407	3211210	3211758	549
RSCM	CP025985_03363	94.80% ISRso16_aa2	64.61% CP025985_01189	IS3 ssgr IS407	3555155	3555454	300
RSCM	CP025985_00008	89.34% ISAisp2_aa2	100% CP025985_03180	IS3 ssgr IS51	12543	11668	876
RSCM	CP025985_00009	81.52% ISAisp2_aa1	100% CP025985_03179	IS3 ssgr IS51	12818	1254	279
RSCM	CP025985_00203	98.68% IS401_aa2	100% CP025985_02545	IS3 ssgr IS51	212649	211735	915
RSCM	CP025985_00204	99.06% IS401_aa1	100% CP025985_02546	IS3 ssgr IS51	212969	212646	324

RSCM	CP025985_00902	81.52% ISAisp2_aa1	100% CP025985_03179	IS3 ssgr IS51	943033	943311	279
RSCM	CP025985_00903	98.68% IS401_aa2	100% CP025985_02545	IS3 ssgr IS51	944257	943343	915
RSCM	CP025985_00904	99.06% IS401_aa1	100% CP025985_02546	IS3 ssgr IS51	944577	944254	324
RSCM	CP025985_00905	89.34% ISAisp2_aa2	100% CP025985_03180	IS3 ssgr IS51	94463	945505	876
RSCM	CP025985_00935	99.06% IS401_aa1	100% CP025985_02546	IS3 ssgr IS51	981648	981971	324
RSCM	CP025985_00936	98.68% IS401_aa2	100% CP025985_02545	IS3 ssgr IS51	981968	982882	915
RSCM	CP025985_01033	98.68% IS401_aa2	100% CP025985_02545	IS3 ssgr IS51	1082925	1082011	915
RSCM	CP025985_01034	99.06% IS401_aa1	100% CP025985_02546	IS3 ssgr IS51	1083245	1082922	324
RSCM	CP025985_01191	98.68% IS401_aa2	100% CP025985_02545	IS3 ssgr IS51	1241093	1240179	915
RSCM	CP025985_01192	99.06% IS401_aa1	100% CP025985_02546	IS3 ssgr IS51	1241413	1241090	324
RSCM	CP025985_01261	98.68% IS401_aa2	100% CP025985_02545	IS3 ssgr IS51	1316797	1315883	915
RSCM	CP025985_01262	99.06% IS401_aa1	100% CP025985_02546	IS3 ssgr IS51	1317117	1316794	324
RSCM	CP025985_01395	99.06% IS401_aa1	100% CP025985_02546	IS3 ssgr IS51	1474291	1474614	324
RSCM	CP025985_01396	98.68% IS401_aa2	100% CP025985_02545	IS3 ssgr IS51	1474611	1475525	915
RSCM	CP025985_01703	89.34% ISAisp2_aa2	100% CP025985_03180	IS3 ssgr IS51	1806148	1805273	876
RSCM	CP025985_01704	81.52% ISAisp2_aa1	100% CP025985_03179	IS3 ssgr IS51	1806423	1806145	279
RSCM	CP025985_01705	99.06% IS401_aa1	100% CP025985_02546	IS3 ssgr IS51	1806980	1807303	324
RSCM	CP025985_01706	98.68% IS401_aa2	100% CP025985_02545	IS3 ssgr IS51	1807300	1808214	915
RSCM	CP025985_01801	98.68% IS401_aa2	100% CP025985_02545	IS3 ssgr IS51	1914759	1913845	915
RSCM	CP025985_01802	99.06% IS401_aa1	100% CP025985_02546	IS3 ssgr IS51	1915079	1914756	324
RSCM	CP025985_01916	89.62% ISAisp2_aa2	100% CP025985_03180	IS3 ssgr IS51	2069193	2068783	411
RSCM	CP025985_01920	88.14% ISAisp2_aa2	100% CP025985_03180	IS3 ssgr IS51	2072216	2071752	465
RSCM	CP025985_01921	81.52% ISAisp2_aa1	100% CP025985_03179	IS3 ssgr IS51	2072491	2072213	279
RSCM	CP025985_01962	89.34% ISAisp2_aa2	100% CP025985_03180	IS3 ssgr IS51	2127293	2126418	876
RSCM	CP025985_01963	81.52% ISAisp2_aa1	100% CP025985_03179	IS3 ssgr IS51	2127568	2127290	279
RSCM	CP025985_02419	99.06% IS401_aa1	100% CP025985_02546	IS3 ssgr IS51	2594298	2594621	324
RSCM	CP025985_02420	98.68% IS401_aa2	100% CP025985_02545	IS3 ssgr IS51	2594618	2595532	915
RSCM	CP025985_02539	89.34% ISAisp2_aa2	100% CP025985_03180	IS3 ssgr IS51	2726589	2725714	876
RSCM	CP025985_02540	81.52% ISAisp2_aa1	100% CP025985_03179	IS3 ssgr IS51	2726864	2726586	279
RSCM	CP025985_02545	98.68% IS401_aa2	100% CP025985_02420	IS3 ssgr IS51	2730787	2729873	915
RSCM	CP025985_02546	99.06% IS401_aa1	100% CP025985_02419	IS3 ssgr IS51	2731107	2730784	324
RSCM	CP025985_03015	95.52% ISMlo4_aa2	76.11% CP025985_01916	IS3 ssgr IS51	3209174	3208959	216
RSCM	CP025985_03020	89.58% ISMlo4_aa1	63.15% CP025985_03179	IS3 ssgr IS51	3212641	3212348	294
RSCM	CP025985_03179	81.52% ISAisp2_aa1	100% CP025985_02540	IS3 ssgr IS51	3376236	3376514	279
RSCM	CP025985_03180	89.34% ISAisp2_aa2	100% CP025985_02539	IS3 ssgr IS51	3376511	3377386	876

RSCM	CP025985_00294	61.68% ISCro3_aa1	100% CP025985_03545	IS4	307369	308697	1329
RSCM	CP025985_00543	61.68% ISCro3_aa1	100% CP025985_03545	IS4	554668	55334	1329
RSCM	CP025985_00727	61.68% ISCro3_aa1	100% CP025985_03545	IS4	740823	739495	1329
RSCM	CP025985_00757	61.68% ISCro3_aa1	100% CP025985_03545	IS4	779003	780331	1329
RSCM	CP025985_01083	61.91% ISCro3_aa1	99.77% CP025985_03545	IS4	1129251	1130579	1329
RSCM	CP025985_01520	61.68% ISCro3_aa1	100% CP025985_03545	IS4	1602634	1603962	1329
RSCM	CP025985_02032	61.68% ISCro3_aa1	100% CP025985_03545	IS4	2214807	2213479	1329
RSCM	CP025985_02217	61.68% ISCro3_aa1	100% CP025985_03545	IS4	2390223	2388895	1329
RSCM	CP025985_02272	61.68% ISCro3_aa1	100% CP025985_03545	IS4	2445467	2446795	1329
RSCM	CP025985_02349	61.68% ISCro3_aa1	100% CP025985_03545	IS4	2524595	2525923	1329
RSCM	CP025985_02439	61.68% ISCro3_aa1	100% CP025985_03545	IS4	2614825	2616153	1329
RSCM	CP025985_02883	61.68% ISCro3_aa1	100% CP025985_03545	IS4	3073946	3075274	1329
RSCM	CP025985_03224	61.68% ISCro3_aa1	100% CP025985_03545	IS4	3424093	3425421	1329
RSCM	CP025985_03279	61.68% ISCro3_aa1	100% CP025985_03545	IS4	3479650	3478322	1329
RSCM	CP025985_03419	61.68% ISCro3_aa1	100% CP025985_03545	IS4	3615499	3616827	1329
RSCM	CP025985_03539	61.68% ISCro3_aa1	100% CP025985_03545	IS4	3747404	3748732	1329
RSCM	CP025985_03545	61.68% ISCro3_aa1	100% CP025985_03539	IS4	3756718	3755390	1329
RSCM	CP025985_00272	97.29% ISRso1_aa1	96.05% CP025985_00931	IS5	271335	271607	273
RSCM	CP025985_00931	98.90% ISRso1_aa1	99.26% CP025985_01903	IS5	974482	976977	2496
RSCM	CP025985_01668	98.90% ISRso1_aa1	100% CP025985_00931	IS5	1758215	1757391	825
RSCM	CP025985_01709	98.90% ISRso1_aa1	100% CP025985_00931	IS5	1811753	1812577	825
RSCM	CP025985_02542	97.44% ISRso1_aa1	97.08% CP025985_00931	IS5	2727610	2728434	825
RSCM	CP025985_02972	98.90% ISRso1_aa1	100% CP025985_00931	IS5	3162501	3163325	825
RSCM	CP025985_03450	98.90% ISRso1_aa1	100% CP025985_00931	IS5	3645161	3645985	825
RSCM	CP025985_00004	100% IS1421_aa1	100% CP025985_03448	IS5 ssgr IS427	6446	6042	405
RSCM	CP025985_00010	100% IS1421_aa1	100% CP025985_03448	IS5 ssgr IS427	12922	13326	405
RSCM	CP025985_00280	87.15% IS1421_aa1	100% CP025985_03518	IS5 ssgr IS427	288876	289247	372
RSCM	CP025985_00635	100% IS1421_aa1	100% CP025985_03448	IS5 ssgr IS427	643601	643197	405
RSCM	CP025985_00895	100% IS1421_aa1	100% CP025985_03448	IS5 ssgr IS427	933725	933321	405
RSCM	CP025985_00960	100% IS1421_aa1	100% CP025985_03448	IS5 ssgr IS427	1011414	1011818	405
RSCM	CP025985_01254	97.54% IS1421_aa1	97.54% CP025985_03448	IS5 ssgr IS427	1311233	1310829	405
RSCM	CP025985_01386	100% IS1421_aa1	100% CP025985_03448	IS5 ssgr IS427	1464737	1465120	384
RSCM	CP025985_01912	100% IS1421_aa1	100% CP025985_03448	IS5 ssgr IS427	2063561	2063157	405
RSCM	CP025985_01922	100% IS1421_aa1	100% CP025985_03448	IS5 ssgr IS427	2072595	2072999	405
RSCM	CP025985_01932	100% IS1421_aa1	100% CP025985_03448	IS5 ssgr IS427	2081304	2081708	405

RSCM	CP025985_02335	87.15% IS1421_aa1	100% CP025985_03518	IS5 ssgr IS427	2513324	2513695	372
RSCM	CP025985_02529	87.15% IS1421_aa1	100% CP025985_03518	IS5 ssgr IS427	2716065	2715694	372
RSCM	CP025985_02543	100% IS1421_aa1	100% CP025985_03448	IS5 ssgr IS427	2729262	2728858	405
RSCM	CP025985_02561	100% IS1421_aa1	100% CP025985_03448	IS5 ssgr IS427	2747053	2746649	405
RSCM	CP025985_02562	87.15% IS1421_aa1	100% CP025985_03518	IS5 ssgr IS427	2747200	2747571	372
RSCM	CP025985_03014	87.15% IS1421_aa1	100% CP025985_03518	IS5 ssgr IS427	3207789	3207418	372
RSCM	CP025985_03118	89.89% IS1421_aa1	98.26% CP025985_03518	IS5 ssgr IS427	3316039	3316416	378
RSCM	CP025985_03148	100% IS1421_aa1	100% CP025985_03448	IS5 ssgr IS427	3342723	3343127	405
RSCM	CP025985_03448	100% IS1421_aa1	100% CP025985_02543	IS5 ssgr IS427	3642887	3643291	405
RSCM	CP025985_03462	87.15% IS1421_aa1	100% CP025985_03518	IS5 ssgr IS427	3661402	3661773	372
RSCM	CP025985_03518	87.15% IS1421_aa1	100% CP025985_03462	IS5 ssgr IS427	3727621	3727992	372
RSCM	CP025985_00033	99.37% IS1405_aa1	100% CP025985_02967	IS5 ssgr IS5	37326	36361	966
RSCM	CP025985_00300	91.76% IS1021_aa1	100% CP025985_03199	IS5 ssgr IS5	314596	315582	987
RSCM	CP025985_00332	99.37% IS1405_aa1	100% CP025985_02967	IS5 ssgr IS5	353916	352951	966
RSCM	CP025985_00334	91.76% IS1021_aa1	100% CP025985_03199	IS5 ssgr IS5	356265	355279	987
RSCM	CP025985_00395	91.76% IS1021_aa1	100% CP025985_03199	IS5 ssgr IS5	416525	415539	987
RSCM	CP025985_00623	99.37% IS1405_aa1	100% CP025985_02967	IS5 ssgr IS5	631623	630658	966
RSCM	CP025985_00728	91.76% IS1021_aa1	100% CP025985_03199	IS5 ssgr IS5	741962	740976	987
RSCM	CP025985_00937	91.76% IS1021_aa1	100% CP025985_03199	IS5 ssgr IS5	984456	98347	987
RSCM	CP025985_00943	99.37% IS1405_aa1	100% CP025985_02967	IS5 ssgr IS5	99078	991745	966
RSCM	CP025985_01037	99.37% IS1405_aa1	100% CP025985_02967	IS5 ssgr IS5	1086045	1087010	966
RSCM	CP025985_01041	99.37% IS1405_aa1	100% CP025985_02967	IS5 ssgr IS5	1090558	1089593	966
RSCM	CP025985_01167	99.37% IS1405_aa1	100% CP025985_02967	IS5 ssgr IS5	1208297	1209262	966
RSCM	CP025985_01170	99.37% IS1405_aa1	100% CP025985_02967	IS5 ssgr IS5	1211636	1210671	966
RSCM	CP025985_01176	91.76% IS1021_aa1	100% CP025985_03199	IS5 ssgr IS5	1216320	1217306	987
RSCM	CP025985_01267	91.76% IS1021_aa1	100% CP025985_03199	IS5 ssgr IS5	1321680	1322666	987
RSCM	CP025985_01281	91.76% IS1021_aa1	100% CP025985_03199	IS5 ssgr IS5	1355386	1354400	987
RSCM	CP025985_01365	91.76% IS1021_aa1	100% CP025985_03199	IS5 ssgr IS5	1445846	1444860	987
RSCM	CP025985_01419	99.37% IS1405_aa1	100% CP025985_02967	IS5 ssgr IS5	1503442	1504407	966
RSCM	CP025985_01429	99.37% IS1405_aa1	100% CP025985_02967	IS5 ssgr IS5	1514055	1513090	966
RSCM	CP025985_01590	99.37% IS1405_aa1	100% CP025985_02967	IS5 ssgr IS5	1674237	1675202	966
RSCM	CP025985_01592	91.76% IS1021_aa1	100% CP025985_03199	IS5 ssgr IS5	1676470	1675484	987
RSCM	CP025985_01595	91.76% IS1021_aa1	100% CP025985_03199	IS5 ssgr IS5	1679486	1680472	987
RSCM	CP025985_01666	99.37% IS1405_aa1	100% CP025985_02967	IS5 ssgr IS5	1756988	1756023	966
RSCM	CP025985_01782	99.37% IS1405_aa1	100% CP025985_02967	IS5 ssgr IS5	1895292	1894327	966

RSCM	CP025985_02015	99.37% IS1405_aa1	100% CP025985_02967	IS5 ssgr IS5	2191938	2192903	966
RSCM	CP025985_02324	91.76% IS1021_aa1	100% CP025985_03199	IS5 ssgr IS5	2503529	2502543	987
RSCM	CP025985_02526	99.06% IS1405_aa1	100% CP025985_03547	IS5 ssgr IS5	2714188	2715153	966
RSCM	CP025985_02565	99.06% IS1405_aa1	100% CP025985_03547	IS5 ssgr IS5	2749078	2748113	966
RSCM	CP025985_02575	99.37% IS1405_aa1	100% CP025985_02967	IS5 ssgr IS5	2756229	2757194	966
RSCM	CP025985_02681	91.76% IS1021_aa1	100% CP025985_03199	IS5 ssgr IS5	2868817	2867831	987
RSCM	CP025985_02766	91.76% IS1021_aa1	100% CP025985_03199	IS5 ssgr IS5	2958175	2957189	987
RSCM	CP025985_02967	99.37% IS1405_aa1	100% CP025985_02575	IS5 ssgr IS5	3158584	3159549	966
RSCM	CP025985_03157	91.76% IS1021_aa1	100% CP025985_03199	IS5 ssgr IS5	3354028	3353042	987
RSCM	CP025985_03199	91.76% IS1021_aa1	100% CP025985_03157	IS5 ssgr IS5	3399553	3398567	987
RSCM	CP025985_03547	99.06% IS1405_aa1	100% CP025985_02565	IS5 ssgr IS5	3757843	3756878	966
RSCM	CP025985_00333	80.88% ISRso5_aa1	100% CP025985_02890	IS630	355065	353983	1083
RSCM	CP025985_00642	86.30% ISRso5_aa1	100% CP025985_02890	IS630	64983	650336	507
RSCM	CP025985_00929	80.88% ISRso5_aa1	100% CP025985_02890	IS630	972532	97145	1083
RSCM	CP025985_00938	80.88% ISRso5_aa1	100% CP025985_02890	IS630	985624	984542	1083
RSCM	CP025985_01119	80.88% ISRso5_aa1	100% CP025985_02890	IS630	1163509	1164591	1083
RSCM	CP025985_01175	80.88% ISRso5_aa1	100% CP025985_02890	IS630	1216156	1215074	1083
RSCM	CP025985_01266	80.88% ISRso5_aa1	100% CP025985_02890	IS630	1320512	1321594	1083
RSCM	CP025985_01364	80.88% ISRso5_aa1	100% CP025985_02890	IS630	1444646	1443564	1083
RSCM	CP025985_01589	80.88% ISRso5_aa1	100% CP025985_02890	IS630	1673088	1674170	1083
RSCM	CP025985_01707	80.88% ISRso5_aa1	100% CP025985_02890	IS630	1810131	1809049	1083
RSCM	CP025985_01905	80.88% ISRso5_aa1	100% CP025985_02890	IS630	2058032	2056950	1083
RSCM	CP025985_02019	69.33% ISCARN39_aa2	No hit	IS630	2197413	2197141	273
RSCM	CP025985_02890	80.88% ISRso5_aa1	100% CP025985_01905	IS630	3081016	3082098	1083
RSCM	CP025985_03142	76.04% ISRso5_aa1	100% CP025985_02890	IS630	3336954	3336370	585
RSCM	CP025985_00195	58.21% ISRm2_aa1	100% CP025985_03141	IS66	20481	205274	465
RSCM	CP025985_00196	75% ISBmu30_aa1	100% CP025985_03140	IS66	205274	205624	351
RSCM	CP025985_00197	58.13% ISAeh1_aa2	100% CP025985_03139	IS66	205656	207227	1572
RSCM	CP025985_00639	58.21% ISRm2_aa1	100% CP025985_03141	IS66	647351	647815	465
RSCM	CP025985_00640	75% ISBmu30_aa1	100% CP025985_03140	IS66	647815	648165	351
RSCM	CP025985_00641	58.13% ISAeh1_aa2	100% CP025985_03139	IS66	648197	649768	1572
RSCM	CP025985_00897	58.13% ISAeh1_aa2	100% CP025985_03139	IS66	940028	938457	1572
RSCM	CP025985_00898	75% ISBmu30_aa1	100% CP025985_03140	IS66	94041	94006	351
RSCM	CP025985_00899	58.21% ISRm2_aa1	100% CP025985_03141	IS66	940874	94041	465
RSCM	CP025985_01248	58.13% ISAeh1_aa2	100% CP025985_03139	IS66	1306413	1304842	1572

RSCM	CP025985_01249	75% ISBmu30_aa1	100% CP025985_03140	IS66	1306795	1306445	351
RSCM	CP025985_01250	58.21% ISRm2_aa1	100% CP025985_03141	IS66	1307259	1306795	465
RSCM	CP025985_01296	58.13% ISAeh1_aa2	100% CP025985_03139	IS66	1369663	1368092	1572
RSCM	CP025985_01297	75% ISBmu30_aa1	100% CP025985_03140	IS66	1370045	1369695	351
RSCM	CP025985_01298	58.21% ISRm2_aa1	100% CP025985_03141	IS66	1370509	1370045	465
RSCM	CP025985_01788	58.21% ISRm2_aa1	100% CP025985_03141	IS66	1900678	1901142	465
RSCM	CP025985_01789	75% ISBmu30_aa1	100% CP025985_03140	IS66	1901142	1901492	351
RSCM	CP025985_01790	58.13% ISAeh1_aa2	100% CP025985_03139	IS66	1901524	1903095	1572
RSCM	CP025985_01796	58.21% ISRm2_aa1	100% CP025985_03141	IS66	1910779	1911243	465
RSCM	CP025985_01797	75% ISBmu30_aa1	100% CP025985_03140	IS66	1911243	1911593	351
RSCM	CP025985_01798	58.13% ISAeh1_aa2	100% CP025985_03139	IS66	1911625	1913196	1572
RSCM	CP025985_01917	58.21% ISRm2_aa1	100% CP025985_03141	IS66	2069349	2069813	465
RSCM	CP025985_01918	75% ISBmu30_aa1	100% CP025985_03140	IS66	2069813	2070163	351
RSCM	CP025985_01919	58.13% ISAeh1_aa2	100% CP025985_03139	IS66	2070195	2071766	1572
RSCM	CP025985_02100	58.21% ISRm2_aa1	100% CP025985_03141	IS66	2268105	2268569	465
RSCM	CP025985_02101	75% ISBmu30_aa1	100% CP025985_03140	IS66	2268569	2268919	351
RSCM	CP025985_02102	58.13% ISAeh1_aa2	100% CP025985_03139	IS66	2268951	2270522	1572
RSCM	CP025985_02409	58.21% ISRm2_aa1	100% CP025985_03141	IS66	2585720	2586184	465
RSCM	CP025985_02410	75% ISBmu30_aa1	100% CP025985_03140	IS66	2586184	2586534	351
RSCM	CP025985_02411	58.13% ISAeh1_aa2	100% CP025985_03139	IS66	2586566	2588137	1572
RSCM	CP025985_02571	58.21% ISRm2_aa1	100% CP025985_03141	IS66	2753110	2753574	465
RSCM	CP025985_02572	75% ISBmu30_aa1	100% CP025985_03140	IS66	2753574	2753924	351
RSCM	CP025985_02573	58.13% ISAeh1_aa2	100% CP025985_03139	IS66	2753956	2755527	1572
RSCM	CP025985_02889	61.07% ISAeh1_aa2	100% CP025985_03139	IS66	3080885	3079530	1356
RSCM	CP025985_02892	75% ISBmu30_aa1	100% CP025985_03140	IS66	3082640	3082290	351
RSCM	CP025985_02893	58.21% ISRm2_aa1	100% CP025985_03141	IS66	3083104	3082640	465
RSCM	CP025985_03048	58.21% ISRm2_aa1	100% CP025985_03141	IS66	3245545	3246009	465
RSCM	CP025985_03049	75% ISBmu30_aa1	100% CP025985_03140	IS66	3246009	3246359	351
RSCM	CP025985_03050	58.13% ISAeh1_aa2	100% CP025985_03139	IS66	3246391	3247962	1572
RSCM	CP025985_03139	58.13% ISAeh1_aa2	100% CP025985_03050	IS66	3335453	3333882	1572
RSCM	CP025985_03140	75% ISBmu30_aa1	100% CP025985_03049	IS66	3335835	3335485	351
RSCM	CP025985_03141	58.21% ISRm2_aa1	100% CP025985_03048	IS66	3336299	3335835	465
RSCM	CP025985_00208	100% ISRso17_aa1	100% CP025985_02971	IS701	215168	216499	1332
RSCM	CP025985_00939	100% ISRso17_aa1	100% CP025985_03120	IS701	987187	985826	1362
RSCM	CP025985_01280	100% ISRso17_aa1	100% CP025985_02971	IS701	1354190	1353243	948

RSCM	CP025985_01282	100% ISRso17_aa1	100% CP025985_03120	IS701	1355815	1355423	393
RSCM	CP025985_01669	100% ISRso17_aa1	100% CP025985_02971	IS701	1758324	1759655	1332
RSCM	CP025985_01708	100% ISRso17_aa1	100% CP025985_02971	IS701	1811644	1810313	1332
RSCM	CP025985_01785	100% ISRso17_aa1	100% CP025985_02971	IS701	1897845	1899176	1332
RSCM	CP025985_02971	100% ISRso17_aa1	100% CP025985_01785	IS701	3162392	3161061	1332
RSCM	CP025985_03120	100% ISRso17_aa1	100% CP025985_00939	IS701	3318493	3317132	1362
RSCM	CP025985_00598	46.49% ISShvi3_aa1	78.65% CP025985_02425	IS91	609626	610672	1047
RSCM	CP025985_02769	47.5% ISTha3_aa2	51.57% CP025985_03516	IS91	2959670	2960596	927
RSCM	CP025985_03516	44.02% ISMno24_aa2	52.21% CP025985_02769	IS91	3725254	3726243	990
RSCM	CP025985_00034	38.97% ISKpn25_aa1	No hit	ISL3	37627	38814	1188
RSCM	CP025985_01867	64.60% ISKpn21_aa1	No hit	ISNCY ssgr IS1202	1980076	1980477	402
RSCM	CP025985_00214	54.71% ISMpo10_aa3	72.83% CP025985_01252	Tn3	221943	220555	1389
RSCM	CP025985_00643	95.44% TnShfr1_aa4	46.81% CP025985_01903	Tn3	65357	650604	2967
RSCM	CP025985_00646	98.91% ISPa38_aa1	50.63% CP025985_00214	Tn3	654846	655403	558
RSCM	CP025985_00932	77.09% ISPa43_aa2	99.77% CP025985_01903	Tn3	976974	978353	138
RSCM	CP025985_01187	54.43% ISMpo10_aa3	91.42% CP025985_01252	Tn3	1237048	1238130	1083
RSCM	CP025985_01252	54.43% ISMpo10_aa3	92.19% CP025985_01187	Tn3	1308294	1309676	1383
RSCM	CP025985_01903	65.71% ISPa43_aa2	99.26% CP025985_00931	Tn3	2055078	2052094	2985
T60	CP022768_03200	61.67% ISMno14_aa1	45.80% CP022768_03537	IS110 ssgr IS1111	3466372	3465359	1014
T60	CP022768_03537	88.75% ISBcen4_aa1	45.80% CP022768_03200	IS110 ssgr IS1111	3797692	3796673	102
T60	CP022768_02993	83.75% ISBusp4_aa1	No hit	IS1182	3249823	3248378	1446
T60	CP022768_00254	49.48% ISHvo5_aa1	100% CP022768_02674	IS1595 ssgr ISH4	279711	280499	789
T60	CP022768_02674	49.48% ISHvo5_aa1	100% CP022768_00254	IS1595 ssgr ISH4	2914211	2913423	789
T60	CP022768_00984	100% ISRso11_aa1	100% CP022768_01984	IS3 ssgr IS150	1067739	1068272	534
T60	CP022768_00985	99.64% ISRso11_aa2	100% CP022768_01983	IS3 ssgr IS150	1068269	1069105	837
T60	CP022768_01983	99.64% ISRso11_aa2	100% CP022768_00985	IS3 ssgr IS150	2186372	2185536	837
T60	CP022768_01984	100% ISRso11_aa1	100% CP022768_00984	IS3 ssgr IS150	2186902	2186369	534
T60	CP022768_01219	88.23% IS222_aa1	60.91% CP022768_01159	IS3 ssgr IS3	1307643	1307951	309
T60	CP022768_01220	88.78% IS222_aa2	54.83% CP022768_02707	IS3 ssgr IS3	1308134	1308805	672
T60	CP022768_01052	89.74% ISAisp2_aa2	97.93% CP022768_02707	IS3 ssgr IS51	1138905	1138195	711
T60	CP022768_01054	89.34% ISAisp2_aa2	100% CP022768_02707	IS3 ssgr IS51	1142098	1141223	876
T60	CP022768_01055	81.52% ISAisp2_aa1	100% CP022768_02708	IS3 ssgr IS51	1142373	1142095	279
T60	CP022768_01158	88.88% ISAisp2_aa2	98.61% CP022768_02707	IS3 ssgr IS51	1251695	1251249	447
T60	CP022768_01159	81.52% ISAisp2_aa1	97.82% CP022768_02708	IS3 ssgr IS51	1251970	1251692	279

T60	CP022768_01207	86.38% ISAisp2_aa2	95.71% CP022768_02707	IS3 ssgr IS51	1296602	1295811	792
T60	CP022768_01208	80.43% ISAisp2_aa1	96.73% CP022768_02708	IS3 ssgr IS51	1296877	1296599	279
T60	CP022768_02707	89.34% ISAisp2_aa2	100% CP022768_01054	IS3 ssgr IS51	2956100	2955225	876
T60	CP022768_02708	81.52% ISAisp2_aa1	100% CP022768_01055	IS3 ssgr IS51	2956375	2956097	279
T60	CP022768_00341	61.68% ISCro3_aa1	100% CP022768_02859	IS4	375137	376465	1329
T60	CP022768_00410	61.68% ISCro3_aa1	100% CP022768_02859	IS4	455754	454426	1329
T60	CP022768_00530	61.68% ISCro3_aa1	100% CP022768_02859	IS4	573464	572136	1329
T60	CP022768_00708	61.44% ISCro3_aa1	100% CP022768_01306	IS4	76293	764258	1329
T60	CP022768_01111	61.68% ISCro3_aa1	100% CP022768_02859	IS4	1201293	1199965	1329
T60	CP022768_01306	61.44% ISCro3_aa1	100% CP022768_00708	IS4	1406221	1407549	1329
T60	CP022768_01795	61.68% ISCro3_aa1	100% CP022768_03631	IS4	1924143	1925471	1329
T60	CP022768_02415	61.68% ISCro3_aa1	100% CP022768_03631	IS4	2651551	2650223	1329
T60	CP022768_02470	61.68% ISCro3_aa1	100% CP022768_02859	IS4	2708009	2706681	1329
T60	CP022768_02565	61.68% ISCro3_aa1	100% CP022768_03631	IS4	2802005	2803333	1329
T60	CP022768_02859	61.68% ISCro3_aa1	100% CP022768_02470	IS4	3108695	3110023	1329
T60	CP022768_03631	61.68% ISCro3_aa1	100% CP022768_02565	IS4	3906443	3905115	1329
T60	CP022768_01223	98.66% ISRso1_aa1	100% CP022768_03556	IS5	1310077	1310754	678
T60	CP022768_02073	97.81% ISRso1_aa1	100% CP022768_03556	IS5	2288479	2289303	825
T60	CP022768_02689	97.44% ISRso1_aa1	98.17% CP022768_03556	IS5	2935724	2936548	825
T60	CP022768_02994	97.81% ISRso1_aa1	100% CP022768_03556	IS5	3250323	3251147	825
T60	CP022768_03556	97.81% ISRso1_aa1	100% CP022768_02994	IS5	3821069	3820245	825
T60	CP022768_00005	99.06% IS1405_aa1	100% CP022768_03451	IS5 ssgr IS5	7027	6062	966
T60	CP022768_00250	85.93% ISAau3_aa1	80% CP022768_00305	IS5 ssgr IS5	273536	273805	270
T60	CP022768_00305	99.37% IS1405_aa1	99.68% CP022768_03451	IS5 ssgr IS5	322642	321677	966
T60	CP022768_00329	94.51% IS1021_aa1	100% CP022768_03408	IS5 ssgr IS5	358208	357222	987
T60	CP022768_00346	98.75% IS1405_aa1	99.68% CP022768_03451	IS5 ssgr IS5	381005	38197	966
T60	CP022768_00412	99.06% IS1405_aa1	100% CP022768_03451	IS5 ssgr IS5	456879	455914	966
T60	CP022768_00621	94.51% IS1021_aa1	100% CP022768_03408	IS5 ssgr IS5	669728	668742	987
T60	CP022768_00881	99.06% IS1405_aa1	100% CP022768_03451	IS5 ssgr IS5	967049	966084	966
T60	CP022768_01431	99.06% IS1405_aa1	100% CP022768_03451	IS5 ssgr IS5	1543236	1542271	966
T60	CP022768_02082	94.51% IS1021_aa1	100% CP022768_03408	IS5 ssgr IS5	2298367	2297381	987
T60	CP022768_02290	94.51% IS1021_aa1	100% CP022768_03408	IS5 ssgr IS5	2532703	2531717	987
T60	CP022768_02311	94.51% IS1021_aa1	100% CP022768_03408	IS5 ssgr IS5	2552512	2551526	987
T60	CP022768_02493	99.06% IS1405_aa1	100% CP022768_03451	IS5 ssgr IS5	2732372	2731407	966
T60	CP022768_02738	99.06% IS1405_aa1	100% CP022768_03451	IS5 ssgr IS5	2996445	2997410	966

T60	CP022768_03359	99.06% IS1405_aa1	100% CP022768_03451	IS5 ssgr IS5	3612313	3613278	966
T60	CP022768_03408	94.51% IS1021_aa1	100% CP022768_02311	IS5 ssgr IS5	3664567	3663581	987
T60	CP022768_03451	99.06% IS1405_aa1	100% CP022768_03359	IS5 ssgr IS5	3715897	3716862	966
T60	CP022768_01595	99.68% IS1420_aa1	No hit	IS5 ssgr IS903	1710791	1709835	957
T60	CP022768_00585	46.25% ISShvi3_aa1	45.16% CP022768_00995	IS91	628568	629491	924
T60	CP022768_00995	47.5% ISTha3_aa2	51.50% CP022768_03608	IS91	1080094	1079168	927
T60	CP022768_03608	43.33% ISMno24_aa2	52.12% CP022768_00995	IS91	3883894	3884883	990
T60	CP022768_00038	38.97% ISKpn25_aa1	No hit	ISL3	50979	52424	1446
T60	CP022768_00635	53.43% ISSm4_aa1	No hit	ISL3	681651	684044	2394
T60	CP022768_00636	43.97% ISSm4_aa3	No hit	ISL3	684044	685447	1404
T60	CP022768_00637	49.68% ISSm4_aa4	No hit	ISL3	685434	68843	2997
T60	CP022768_01921	62.5% ISKpn21_aa1	93.33% CP022768_01922	ISNCY ssgr IS1202	2120870	2120469	402
T60	CP022768_01922	72.27% ISKpn21_aa1	93.33% CP022768_01921	ISNCY ssgr IS1202	2122499	2121024	1476
T60	CP022768_00192	61.81% ISMpo10_aa3	97.83% CP022768_00259	Tn3	213223	211835	1389
T60	CP022768_00259	61.81% ISMpo10_aa3	97.83% CP022768_00192	Tn3	285427	284039	1389
T60	CP022768_01191	61.87% ISPa38_aa1	54.90% CP022768_00259	Tn3	1286105	1286695	591
T60	CP022768_02591	54.43% ISMpo10_aa3	94.56% CP022768_02732	Tn3	2832048	2830669	138
T60	CP022768_02672	54.43% ISMpo10_aa3	98.91% CP022768_02732	Tn3	2911998	2910616	1383
T60	CP022768_02732	54.43% ISMpo10_aa3	98.91% CP022768_02672	Tn3	2980247	2978865	1383
SL3882	CP022778_03198	61.67% ISMno14_aa1	45.80% CP022778_03532	IS110 ssgr IS1111	3465201	3464188	1014
SL3882	CP022778_03532	88.75% ISBcen4_aa1	45.80% CP022778_03198	IS110 ssgr IS1111	3796537	3795518	102
SL3882	CP022778_02991	83.75% ISBusp4_aa1	No hit	IS1182	3248652	3247207	1446
SL3882	CP022778_00254	49.48% ISHvo5_aa1	100% CP022778_02672	IS1595 ssgr ISH4	278536	279324	789
SL3882	CP022778_02672	49.48% ISHvo5_aa1	100% CP022778_00254	IS1595 ssgr ISH4	2913037	2912249	789
SL3882	CP022778_00986	100% ISRso11_aa1	100% CP022778_01985	IS3 ssgr IS150	1066575	1067108	534
SL3882	CP022778_00987	99.64% ISRso11_aa2	100% CP022778_01984	IS3 ssgr IS150	1067105	1067941	837
SL3882	CP022778_01984	99.64% ISRso11_aa2	100% CP022778_00987	IS3 ssgr IS150	2185194	2184358	837
SL3882	CP022778_01985	100% ISRso11_aa1	100% CP022778_00986	IS3 ssgr IS150	2185724	2185191	534
SL3882	CP022778_01221	88.23% IS222_aa1	60.91% CP022778_01161	IS3 ssgr IS3	1306480	1306788	309
SL3882	CP022778_01222	88.78% IS222_aa2	54.83% CP022778_02704	IS3 ssgr IS3	1306971	1307642	672
SL3882	CP022778_01054	89.74% ISAisp2_aa2	97.93% CP022778_02704	IS3 ssgr IS51	1137741	1137031	711
SL3882	CP022778_01056	89.34% ISAisp2_aa2	100% CP022778_02704	IS3 ssgr IS51	1140935	1140060	876
SL3882	CP022778_01057	81.52% ISAisp2_aa1	100% CP022778_02705	IS3 ssgr IS51	1141210	1140932	279
SL3882	CP022778_01160	88.88% ISAisp2_aa2	98.61% CP022778_02704	IS3 ssgr IS51	1250532	1250086	447

SL3882	CP022778_01161	81.52% ISAisp2_aa1	97.82% CP022778_02705	IS3 ssgr IS51	1250807	1250529	279
SL3882	CP022778_01209	86.38% ISAisp2_aa2	95.71% CP022778_02704	IS3 ssgr IS51	1295439	1294648	792
SL3882	CP022778_01210	80.43% ISAisp2_aa1	96.73% CP022778_02705	IS3 ssgr IS51	1295714	1295436	279
SL3882	CP022778_02704	89.34% ISAisp2_aa2	100% CP022778_01056	IS3 ssgr IS51	2954929	2954054	876
SL3882	CP022778_02705	81.52% ISAisp2_aa1	100% CP022778_01057	IS3 ssgr IS51	2955204	2954926	279
SL3882	CP022778_00342	61.68% ISCro3_aa1	100% CP022778_02856	IS4	373963	375291	1329
SL3882	CP022778_00411	61.68% ISCro3_aa1	100% CP022778_02856	IS4	45458	453252	1329
SL3882	CP022778_00531	61.68% ISCro3_aa1	100% CP022778_02856	IS4	57229	570962	1329
SL3882	CP022778_00709	61.44% ISCro3_aa1	100% CP022778_01309	IS4	761765	763093	1329
SL3882	CP022778_01113	61.68% ISCro3_aa1	100% CP022778_02856	IS4	1200130	1198802	1329
SL3882	CP022778_01309	61.44% ISCro3_aa1	100% CP022778_00709	IS4	1405057	1406385	1329
SL3882	CP022778_01798	61.68% ISCro3_aa1	100% CP022778_03626	IS4	1922974	1924302	1329
SL3882	CP022778_02415	61.68% ISCro3_aa1	100% CP022778_03626	IS4	2650372	2649044	1329
SL3882	CP022778_02470	61.68% ISCro3_aa1	100% CP022778_02856	IS4	2706830	2705502	1329
SL3882	CP022778_02565	61.68% ISCro3_aa1	100% CP022778_03626	IS4	2800830	2802158	1329
SL3882	CP022778_02856	61.68% ISCro3_aa1	100% CP022778_02470	IS4	3107524	3108852	1329
SL3882	CP022778_03626	61.68% ISCro3_aa1	100% CP022778_02565	IS4	3905288	3903960	1329
SL3882	CP022778_01225	98.66% ISRso1_aa1	100% CP022778_03551	IS5	1308914	1309591	678
SL3882	CP022778_02074	97.81% ISRso1_aa1	100% CP022778_03551	IS5	2287301	2288125	825
SL3882	CP022778_02686	97.44% ISRso1_aa1	98.17% CP022778_03551	IS5	2934550	2935374	825
SL3882	CP022778_02992	97.81% ISRso1_aa1	100% CP022778_03551	IS5	3249152	3249976	825
SL3882	CP022778_03551	97.81% ISRso1_aa1	100% CP022778_02992	IS5	3819914	3819090	825
SL3882	CP022778_00249	85.93% ISAau3_aa1	80% CP022778_00306	IS5 ssgr IS5	272361	27263	270
SL3882	CP022778_00306	99.37% IS1405_aa1	99.68% CP022778_03446	IS5 ssgr IS5	321468	320503	966
SL3882	CP022778_00330	94.51% IS1021_aa1	100% CP022778_03403	IS5 ssgr IS5	357034	356048	987
SL3882	CP022778_00347	98.75% IS1405_aa1	99.68% CP022778_03446	IS5 ssgr IS5	379831	380796	966
SL3882	CP022778_00413	99.06% IS1405_aa1	100% CP022778_03446	IS5 ssgr IS5	455705	45474	966
SL3882	CP022778_00622	94.51% IS1021_aa1	100% CP022778_03403	IS5 ssgr IS5	668563	667577	987
SL3882	CP022778_00884	99.06% IS1405_aa1	100% CP022778_03446	IS5 ssgr IS5	965884	964919	966
SL3882	CP022778_01434	99.06% IS1405_aa1	100% CP022778_03446	IS5 ssgr IS5	1542071	1541106	966
SL3882	CP022778_02083	94.51% IS1021_aa1	100% CP022778_03403	IS5 ssgr IS5	2297189	2296203	987
SL3882	CP022778_02291	94.51% IS1021_aa1	100% CP022778_03403	IS5 ssgr IS5	2531525	2530539	987
SL3882	CP022778_02311	94.51% IS1021_aa1	100% CP022778_03403	IS5 ssgr IS5	2551333	2550347	987
SL3882	CP022778_02493	99.06% IS1405_aa1	100% CP022778_03446	IS5 ssgr IS5	2731193	2730228	966
SL3882	CP022778_02735	99.06% IS1405_aa1	100% CP022778_03446	IS5 ssgr IS5	2995274	2996239	966

SL3882	CP022778_03354	99.06% IS1405_aa1	100% CP022778_03446	IS5 ssgr IS5	3611158	3612123	966
SL3882	CP022778_03403	94.51% IS1021_aa1	100% CP022778_02311	IS5 ssgr IS5	3663412	3662426	987
SL3882	CP022778_03446	99.06% IS1405_aa1	100% CP022778_03354	IS5 ssgr IS5	3714742	3715707	966
SL3882	CP022778_01599	99.68% IS1420_aa1	No hit	IS5 ssgr IS903	1709626	1708670	957
SL3882	CP022778_00586	46.25% ISShvi3_aa1	45.16% CP022778_00997	IS91	627394	628317	924
SL3882	CP022778_00997	47.5% ISTha3_aa2	51.50% CP022778_03603	IS91	1078930	1078004	927
SL3882	CP022778_03603	43.33% ISMno24_aa2	52.12% CP022778_00997	IS91	3882739	3883728	990
SL3882	CP022778_00036	38.97% ISKpn25_aa1	No hit	ISL3	49802	51247	1446
SL3882	CP022778_00636	53.43% ISSm4_aa1	No hit	ISL3	680486	682879	2394
SL3882	CP022778_00637	43.97% ISSm4_aa3	No hit	ISL3	682879	684282	1404
SL3882	CP022778_00638	49.68% ISSm4_aa4	No hit	ISL3	684269	687265	2997
SL3882	CP022778_01924	62.5% ISKpn21_aa1	93.33% CP022778_01925	ISNCY ssgr IS1202	2119701	2119300	402
SL3882	CP022778_01925	73.84% ISKpn21_aa1	93.33% CP022778_01924	ISNCY ssgr IS1202	2121329	2119842	1488
SL3882	CP022778_00190	61.81% ISMpo10_aa3	97.83% CP022778_00259	Tn3	212047	210659	1389
SL3882	CP022778_00259	61.81% ISMpo10_aa3	97.83% CP022778_00190	Tn3	284252	282864	1389
SL3882	CP022778_01193	61.87% ISPa38_aa1	54.90% CP022778_00259	Tn3	1284942	1285532	591
SL3882	CP022778_02590	54.43% ISMpo10_aa3	94.56% CP022778_02729	Tn3	2830874	2829495	138
SL3882	CP022778_02670	54.43% ISMpo10_aa3	98.91% CP022778_02729	Tn3	2910824	2909442	1383
SL3882	CP022778_02729	54.43% ISMpo10_aa3	98.91% CP022778_02670	Tn3	2979076	2977694	1383
T42	CP022772_02970	61.67% ISMno14_aa1	45.80% CP022772_03313	IS110 ssgr IS1111	3231151	3230138	1014
T42	CP022772_03313	88.75% ISBcen4_aa1	45.80% CP022772_02970	IS110 ssgr IS1111	3568459	3567440	102
T42	CP022772_00948	83.75% ISBusp4_aa1	100% CP022772_02749	IS1182	1029140	1030585	1446
T42	CP022772_02749	83.75% ISBusp4_aa1	100% CP022772_00948	IS1182	2999590	2998145	1446
T42	CP022772_00957	54.76% ISHpa1_aa1	No hit	IS1595 ssgr IS1016	1041129	1041908	780
T42	CP022772_00874	100% ISRso11_aa1	100% CP022772_01935	IS3 ssgr IS150	951938	952471	534
T42	CP022772_00875	99.64% ISRso11_aa2	100% CP022772_01934	IS3 ssgr IS150	952468	953304	837
T42	CP022772_01934	99.64% ISRso11_aa2	100% CP022772_00875	IS3 ssgr IS150	2146646	2145810	837
T42	CP022772_01935	100% ISRso11_aa1	100% CP022772_00874	IS3 ssgr IS150	2147176	2146643	534
T42	CP022772_01157	88.23% IS222_aa1	60.91% CP022772_01065	IS3 ssgr IS3	1258639	1258947	309
T42	CP022772_01158	88.78% IS222_aa2	55.76% CP022772_01064	IS3 ssgr IS3	1259130	1259801	672
T42	CP022772_00649	81.52% ISAisp2_aa1	100% CP022772_01065	IS3 ssgr IS51	706898	707176	279
T42	CP022772_00650	89.34% ISAisp2_aa2	100% CP022772_01064	IS3 ssgr IS51	707173	708048	876
T42	CP022772_00954	81.52% ISAisp2_aa1	100% CP022772_01065	IS3 ssgr IS51	1037929	1038207	279
T42	CP022772_00955	89.34% ISAisp2_aa2	100% CP022772_01064	IS3 ssgr IS51	1038204	1039079	876

T42	CP022772_01064	89.34% ISAisp2_aa2	100% CP022772_00955	IS3 ssgr IS51	1156478	1155603	876
T42	CP022772_01065	81.52% ISAisp2_aa1	100% CP022772_00954	IS3 ssgr IS51	1156753	1156475	279
T42	CP022772_01145	86.38% ISAisp2_aa2	94.94% CP022772_01064	IS3 ssgr IS51	1247598	1246807	792
T42	CP022772_01146	80.43% ISAisp2_aa1	96.73% CP022772_03063	IS3 ssgr IS51	1247873	1247595	279
T42	CP022772_03062	89.34% ISAisp2_aa2	98.28% CP022772_01064	IS3 ssgr IS51	3316524	3315649	876
T42	CP022772_03063	81.52% ISAisp2_aa1	97.82% CP022772_01065	IS3 ssgr IS51	3316799	3316521	279
T42	CP022772_00273	61.68% ISCro3_aa1	100% CP022772_02646	IS4	299464	300792	1329
T42	CP022772_00343	61.68% ISCro3_aa1	100% CP022772_02646	IS4	382139	380811	1329
T42	CP022772_00461	61.68% ISCro3_aa1	100% CP022772_02646	IS4	498665	497337	1329
T42	CP022772_00580	61.44% ISCro3_aa1	100% CP022772_01242	IS4	621808	623136	1329
T42	CP022772_01015	61.68% ISCro3_aa1	100% CP022772_02646	IS4	1102227	1100899	1329
T42	CP022772_01242	61.44% ISCro3_aa1	100% CP022772_00580	IS4	1356328	1357656	1329
T42	CP022772_01742	61.68% ISCro3_aa1	100% CP022772_03406	IS4	1884438	1885766	1329
T42	CP022772_02321	61.68% ISCro3_aa1	100% CP022772_03406	IS4	2567540	2566212	1329
T42	CP022772_02376	61.68% ISCro3_aa1	99.77% CP022772_02646	IS4	2623998	2622670	1329
T42	CP022772_02403	61.68% ISCro3_aa1	100% CP022772_02646	IS4	2653169	2651841	1329
T42	CP022772_02472	61.68% ISCro3_aa1	100% CP022772_03406	IS4	2719441	2720769	1329
T42	CP022772_02646	61.68% ISCro3_aa1	100% CP022772_02403	IS4	2897491	2898819	1329
T42	CP022772_03406	61.68% ISCro3_aa1	100% CP022772_02472	IS4	3678981	3677653	1329
T42	CP022772_00261	78.03% ISAzo5_aa1	100% CP022772_00914	IS4 ssgr IS50	281763	283088	1326
T42	CP022772_00644	78.03% ISAzo5_aa1	100% CP022772_00914	IS4 ssgr IS50	699047	700372	1326
T42	CP022772_00914	78.03% ISAzo5_aa1	100% CP022772_00644	IS4 ssgr IS50	992489	993814	1326
T42	CP022772_01363	97.81% ISRso1_aa1	100% CP022772_02025	IS5	1487022	1486198	825
T42	CP022772_02025	97.81% ISRso1_aa1	100% CP022772_01363	IS5	2248780	2249604	825
T42	CP022772_00236	99.37% IS1405_aa1	99.68% CP022772_03187	IS5 ssgr IS5	246699	245734	966
T42	CP022772_00278	99.06% IS1405_aa1	100% CP022772_03187	IS5 ssgr IS5	305332	306297	966
T42	CP022772_00663	94.51% IS1021_aa1	100% CP022772_03249	IS5 ssgr IS5	724186	725172	987
T42	CP022772_00770	94.51% IS1021_aa1	100% CP022772_03249	IS5 ssgr IS5	849177	848191	987
T42	CP022772_01361	99.06% IS1405_aa1	100% CP022772_03187	IS5 ssgr IS5	1484513	1483548	966
T42	CP022772_01528	99.06% IS1405_aa1	100% CP022772_03187	IS5 ssgr IS5	1650698	1649733	966
T42	CP022772_02399	99.06% IS1405_aa1	100% CP022772_03187	IS5 ssgr IS5	2648361	2647396	966
T42	CP022772_02504	99.06% IS1405_aa1	100% CP022772_03187	IS5 ssgr IS5	2764870	2765835	966
T42	CP022772_02522	99.06% IS1405_aa1	100% CP022772_03187	IS5 ssgr IS5	2783798	2782833	966
T42	CP022772_03059	94.51% IS1021_aa1	100% CP022772_03249	IS5 ssgr IS5	3313460	3312474	987
T42	CP022772_03068	99.06% IS1405_aa1	100% CP022772_03187	IS5 ssgr IS5	3322253	3321288	966

T42	CP022772_03087	94.51% IS1021_aa1	100% CP022772_03249	IS5 ssgr IS5	3335636	3336622	987
T42	CP022772_03134	99.06% IS1405_aa1	100% CP022772_03187	IS5 ssgr IS5	3381943	3382908	966
T42	CP022772_03187	99.06% IS1405_aa1	100% CP022772_03134	IS5 ssgr IS5	3442953	3441988	966
T42	CP022772_03249	94.51% IS1021_aa1	100% CP022772_03087	IS5 ssgr IS5	3505797	3506783	987
T42	CP022772_00008	100% IS1420_aa1	100% CP022772_03147	IS5 ssgr IS903	15896	1494	957
T42	CP022772_00643	100% IS1420_aa1	100% CP022772_03147	IS5 ssgr IS903	697114	69807	957
T42	CP022772_00768	100% IS1420_aa1	100% CP022772_03147	IS5 ssgr IS903	838681	839637	957
T42	CP022772_02753	100% IS1420_aa1	100% CP022772_03147	IS5 ssgr IS903	3002990	3002034	957
T42	CP022772_02755	100% IS1420_aa1	100% CP022772_03147	IS5 ssgr IS903	3004663	3005619	957
T42	CP022772_02793	100% IS1420_aa1	100% CP022772_03147	IS5 ssgr IS903	3044497	3045453	957
T42	CP022772_03147	100% IS1420_aa1	100% CP022772_02793	IS5 ssgr IS903	3393255	3394211	957
T42	CP022772_00886	47.5% ISTha3_aa2	51.50% CP022772_03383	IS91	964292	963366	927
T42	CP022772_01625	40.65% ISShvi3_aa1	51.94% CP022772_01611	IS91	1760815	1759751	1065
T42	CP022772_03383	43.33% ISMno24_aa2	52.12% CP022772_00886	IS91	3656432	3657421	990
T42	CP022772_00038	38.97% ISKpn25_aa1	No hit	ISL3	5096	52405	1446
T42	CP022772_01870	62.5% ISKpn21_aa1	93.33% CP022772_01871	ISNCY ssgr IS1202	2081147	2080746	402
T42	CP022772_01871	72.27% ISKpn21_aa1	93.33% CP022772_01870	ISNCY ssgr IS1202	2082776	2081301	1476
T42	CP022772_00194	61.81% ISMpo10_aa3	73.17% CP022772_02497	Tn3	214357	212969	1389
T42	CP022772_01615	75.40% ISPa40_aa4	54.09% CP022772_00194	Tn3	1747055	1747651	597
T42	CP022772_02497	55.06% ISMpo10_aa3	73.17% CP022772_00194	Tn3	2749488	2748106	1383

SL3300	CP022786_03134	61.67% ISMno14_aa1	45.80% CP022786_03474	IS110 ssgr IS1111	3385111	3384098	1014
SL3300	CP022786_03474	88.75% ISBcen4_aa1	45.80% CP022786_03134	IS110 ssgr IS1111	3718056	3717037	102
SL3300	CP022786_01001	83.75% ISBusp4_aa1	100% CP022786_02926	IS1182	1086904	1088349	1446
SL3300	CP022786_02926	83.75% ISBusp4_aa1	100% CP022786_01001	IS1182	3168255	3166810	1446
SL3300	CP022786_02920	54.76% ISHpa1_aa1	No hit	IS1595 ssgr IS1016	3157500	3156721	780
SL3300	CP022786_00927	100% ISRso11_aa1	100% CP022786_01894	IS3 ssgr IS150	1008422	1008955	534
SL3300	CP022786_00928	99.64% ISRso11_aa2	100% CP022786_01895	IS3 ssgr IS150	1008952	1009788	837
SL3300	CP022786_01894	100% ISRso11_aa1	100% CP022786_00927	IS3 ssgr IS150	2021792	2022325	534
SL3300	CP022786_01895	99.64% ISRso11_aa2	100% CP022786_00928	IS3 ssgr IS150	2022322	2023158	837
SL3300	CP022786_02764	88.78% IS222_aa2	55.76% CP022786_02812	IS3 ssgr IS3	2992584	2991913	672
SL3300	CP022786_02765	88.23% IS222_aa1	60.91% CP022786_02811	IS3 ssgr IS3	2993075	2992767	309
SL3300	CP022786_00706	89.34% ISAisp2_aa2	100% CP022786_02812	IS3 ssgr IS51	767406	766531	876
SL3300	CP022786_00707	81.52% ISAisp2_aa1	100% CP022786_02811	IS3 ssgr IS51	767681	767403	279
SL3300	CP022786_02260	89.34% ISAisp2_aa2	100% CP022786_03225	IS3 ssgr IS51	2454389	2453514	876

SL3300	CP022786_02261	81.52% ISAisp2_aa1	100% CP022786_03226	IS3 ssgr IS51	2454664	2454386	279
SL3300	CP022786_02777	80.43% ISAisp2_aa1	96.73% CP022786_03226	IS3 ssgr IS51	3005053	3005331	279
SL3300	CP022786_02778	86.38% ISAisp2_aa2	94.94% CP022786_02812	IS3 ssgr IS51	3005328	3006119	792
SL3300	CP022786_02811	81.52% ISAisp2_aa1	100% CP022786_00707	IS3 ssgr IS51	3040663	3040941	279
SL3300	CP022786_02812	89.34% ISAisp2_aa2	100% CP022786_00706	IS3 ssgr IS51	3040938	3041813	876
SL3300	CP022786_03225	89.34% ISAisp2_aa2	100% CP022786_02260	IS3 ssgr IS51	3469271	3468396	876
SL3300	CP022786_03226	81.52% ISAisp2_aa1	100% CP022786_02261	IS3 ssgr IS51	3469546	3469268	279
SL3300	CP022786_00301	61.68% ISCro3_aa1	100% CP022786_03570	IS4	316009	317337	1329
SL3300	CP022786_00401	61.68% ISCro3_aa1	100% CP022786_03570	IS4	443302	44463	1329
SL3300	CP022786_00514	61.68% ISCro3_aa1	100% CP022786_03570	IS4	554745	553417	1329
SL3300	CP022786_00633	61.44% ISCro3_aa1	100% CP022786_02679	IS4	677888	679216	1329
SL3300	CP022786_01134	61.68% ISCro3_aa1	100% CP022786_03570	IS4	1221972	1220644	1329
SL3300	CP022786_01311	61.68% ISCro3_aa1	100% CP022786_02083	IS4	1406719	1405391	1329
SL3300	CP022786_01406	61.68% ISCro3_aa1	99.77% CP022786_03570	IS4	1500715	1502043	1329
SL3300	CP022786_01461	61.68% ISCro3_aa1	100% CP022786_02083	IS4	1557173	1558501	1329
SL3300	CP022786_02083	61.68% ISCro3_aa1	100% CP022786_01461	IS4	2282907	2281579	1329
SL3300	CP022786_02679	61.44% ISCro3_aa1	100% CP022786_00633	IS4	2893631	2892303	1329
SL3300	CP022786_02862	61.68% ISCro3_aa1	100% CP022786_03570	IS4	3096401	3097729	1329
SL3300	CP022786_03570	61.68% ISCro3_aa1	100% CP022786_02862	IS4	3831038	3829710	1329
SL3300	CP022786_00966	78.03% ISAzo5_aa1	100% CP022786_03576	IS4 ssgr IS50	1048985	1050310	1326
SL3300	CP022786_03576	78.03% ISAzo5_aa1	100% CP022786_00966	IS4 ssgr IS50	3838072	3839397	1326
SL3300	CP022786_00014	40.42% ISGur11_aa1	45.59% CP022786_01027	IS481	17601	19568	1968
SL3300	CP022786_01260	97.81% ISRso1_aa1	100% CP022786_02762	IS5	1339854	1338850	1005
SL3300	CP022786_01804	97.81% ISRso1_aa1	100% CP022786_01260	IS5	1920197	1919373	825
SL3300	CP022786_02762	97.81% ISRso1_aa1	100% CP022786_01260	IS5	2989659	2990483	825
SL3300	CP022786_00117	99.25% IS1421_aa1	100% CP022786_03384	IS5 ssgr IS427	143148	143552	405
SL3300	CP022786_00135	99.25% IS1421_aa1	100% CP022786_03384	IS5 ssgr IS427	158949	158545	405
SL3300	CP022786_00696	99.25% IS1421_aa1	100% CP022786_03384	IS5 ssgr IS427	754315	753911	405
SL3300	CP022786_00716	99.25% IS1421_aa1	100% CP022786_03384	IS5 ssgr IS427	782251	782655	405
SL3300	CP022786_02763	99.25% IS1421_aa1	100% CP022786_03384	IS5 ssgr IS427	2990558	2990962	405
SL3300	CP022786_03384	99.25% IS1421_aa1	100% CP022786_02763	IS5 ssgr IS427	3630278	3630682	405
SL3300	CP022786_00021	99.06% IS1405_aa1	100% CP022786_03295	IS5 ssgr IS5	28598	27633	966
SL3300	CP022786_00024	94.51% IS1021_aa1	100% CP022786_03514	IS5 ssgr IS5	35526	3454	987
SL3300	CP022786_00028	99.06% IS1405_aa1	100% CP022786_03295	IS5 ssgr IS5	40907	41872	966
SL3300	CP022786_00063	94.51% IS1021_aa1	100% CP022786_03514	IS5 ssgr IS5	74878	73892	987

SL3300	CP022786_00288	94.51% IS1021_aa1	100% CP022786_03514	IS5 ssgr IS5	301614	3026	987
SL3300	CP022786_00365	94.51% IS1021_aa1	100% CP022786_03514	IS5 ssgr IS5	391796	392782	987
SL3300	CP022786_00406	99.06% IS1405_aa1	100% CP022786_03295	IS5 ssgr IS5	44917	450135	966
SL3300	CP022786_00408	94.51% IS1021_aa1	100% CP022786_03514	IS5 ssgr IS5	451402	450416	987
SL3300	CP022786_00697	94.51% IS1021_aa1	100% CP022786_03514	IS5 ssgr IS5	755803	754817	987
SL3300	CP022786_00699	99.06% IS1405_aa1	100% CP022786_03295	IS5 ssgr IS5	758989	759954	966
SL3300	CP022786_00712	94.51% IS1021_aa1	100% CP022786_03514	IS5 ssgr IS5	775589	774603	987
SL3300	CP022786_00821	94.51% IS1021_aa1	100% CP022786_03514	IS5 ssgr IS5	905657	904671	987
SL3300	CP022786_00997	94.51% IS1021_aa1	100% CP022786_03514	IS5 ssgr IS5	1081396	1082382	987
SL3300	CP022786_01216	94.51% IS1021_aa1	100% CP022786_03514	IS5 ssgr IS5	1300999	1301985	987
SL3300	CP022786_01217	94.51% IS1021_aa1	100% CP022786_03514	IS5 ssgr IS5	1303381	1302395	987
SL3300	CP022786_01218	94.51% IS1021_aa1	100% CP022786_03514	IS5 ssgr IS5	1304487	1305473	987
SL3300	CP022786_01261	99.37% IS1405_aa1	99.68% CP022786_03295	IS5 ssgr IS5	1340816	1339851	966
SL3300	CP022786_01278	99.06% IS1405_aa1	100% CP022786_03295	IS5 ssgr IS5	1358703	1357738	966
SL3300	CP022786_01383	99.06% IS1405_aa1	100% CP022786_03295	IS5 ssgr IS5	1476352	1477317	966
SL3300	CP022786_01552	94.51% IS1021_aa1	100% CP022786_03514	IS5 ssgr IS5	1643581	1642595	987
SL3300	CP022786_01567	99.06% IS1405_aa1	100% CP022786_03295	IS5 ssgr IS5	1657490	1656525	966
SL3300	CP022786_01588	94.51% IS1021_aa1	100% CP022786_03514	IS5 ssgr IS5	1677154	1678140	987
SL3300	CP022786_02110	99.06% IS1405_aa1	100% CP022786_03295	IS5 ssgr IS5	2307035	2308000	966
SL3300	CP022786_02259	94.36% IS1021_aa1	100% CP022786_03514	IS5 ssgr IS5	2452651	2453517	867
SL3300	CP022786_02263	94.51% IS1021_aa1	100% CP022786_03514	IS5 ssgr IS5	2455098	2456084	987
SL3300	CP022786_02271	94.51% IS1021_aa1	100% CP022786_03514	IS5 ssgr IS5	2466056	2467042	987
SL3300	CP022786_02544	98.75% IS1405_aa1	100% CP022786_02968	IS5 ssgr IS5	2748330	2749295	966
SL3300	CP022786_02565	94.51% IS1021_aa1	100% CP022786_03514	IS5 ssgr IS5	2774096	2775082	987
SL3300	CP022786_02610	99.06% IS1405_aa1	100% CP022786_03295	IS5 ssgr IS5	2817924	2818889	966
SL3300	CP022786_02770	94.51% IS1021_aa1	100% CP022786_03514	IS5 ssgr IS5	2998623	2997637	987
SL3300	CP022786_02814	94.51% IS1021_aa1	100% CP022786_03514	IS5 ssgr IS5	3043863	3042877	987
SL3300	CP022786_02968	98.75% IS1405_aa1	100% CP022786_02544	IS5 ssgr IS5	3211158	3210193	966
SL3300	CP022786_03228	94.51% IS1021_aa1	100% CP022786_03514	IS5 ssgr IS5	3470494	3471480	987
SL3300	CP022786_03295	99.06% IS1405_aa1	100% CP022786_02610	IS5 ssgr IS5	3531776	3532741	966
SL3300	CP022786_03310	94.51% IS1021_aa1	100% CP022786_03514	IS5 ssgr IS5	3548070	3549056	987
SL3300	CP022786_03401	94.51% IS1021_aa1	100% CP022786_03514	IS5 ssgr IS5	3649258	3648272	987
SL3300	CP022786_03495	94.51% IS1021_aa1	100% CP022786_03514	IS5 ssgr IS5	3744254	3743268	987
SL3300	CP022786_03514	94.51% IS1021_aa1	100% CP022786_03495	IS5 ssgr IS5	3768419	3769405	987
SL3300	CP022786_00938	47.5% ISTha3_aa2	51.50% CP022786_03547	IS91	1020777	1019851	927

SL3300	CP022786_02256	40.65% ISShvi3_aa1	51.94% CP022786_02277	IS91	2450074	2451138	1065
SL3300	CP022786_03547	43.33% ISMno24_aa2	52.12% CP022786_00938	IS91	3808489	3809478	990
SL3300	CP022786_00088	38.97% ISKpn25_aa1	No hit	ISL3	104777	106222	1446
SL3300	CP022786_01956	62.5% ISKpn21_aa1	No hit	ISNCY ssgr IS1202	2086195	2086596	402
SL3300	CP022786_00245	61.81% ISMpo10_aa3	73.64% CP022786_01285	Tn3	269938	26855	1389
SL3300	CP022786_01285	54.43% ISMpo10_aa3	73.64% CP022786_00245	Tn3	1374754	1376136	1383
SL3300	CP022786_02273	75.40% ISPa40_aa4	54.09% CP022786_00245	Tn3	2468705	2468109	597
SL3822	CP022780_03024	61.67% ISMno14_aa1	45.80% CP022780_03377	IS110 ssgr IS1111	3316528	3315515	1014
SL3822	CP022780_03377	88.75% ISBcen4_aa1	45.80% CP022780_03024	IS110 ssgr IS1111	3688829	3687810	102
SL3822	CP022780_02818	83.75% ISBusp4_aa1	No hit	IS1182	3100867	3099422	1446
SL3822	CP022780_00969	54.76% ISHpa1_aa1	No hit	IS1595 ssgr IS1016	1054928	1055707	780
SL3822	CP022780_00252	49.48% ISHvo5_aa1	100% CP022780_02559	IS1595 ssgr ISH4	279315	280103	789
SL3822	CP022780_02559	49.48% ISHvo5_aa1	100% CP022780_00252	IS1595 ssgr ISH4	2840599	2839811	789
SL3822	CP022780_00889	100% ISRso11_aa1	100% CP022780_01937	IS3 ssgr IS150	967801	968334	534
SL3822	CP022780_00890	99.64% ISRso11_aa2	100% CP022780_01936	IS3 ssgr IS150	968331	969167	837
SL3822	CP022780_01936	99.64% ISRso11_aa2	100% CP022780_00890	IS3 ssgr IS150	2164621	2163785	837
SL3822	CP022780_01937	100% ISRso11_aa1	100% CP022780_00889	IS3 ssgr IS150	2165151	2164618	534
SL3822	CP022780_01167	88.23% IS222_aa1	60.91% CP022780_01079	IS3 ssgr IS3	1280292	1280600	309
SL3822	CP022780_01168	88.78% IS222_aa2	55.76% CP022780_01078	IS3 ssgr IS3	1280783	1281454	672
SL3822	CP022780_00526	97.84% ISBp1_aa2	40.35% CP022780_01936	IS3 ssgr IS407	575494	574793	702
SL3822	CP022780_00527	100% ISBp1_aa1	No hit	IS3 ssgr IS407	575904	575641	264
SL3822	CP022780_00965	89.34% ISAisp2_aa2	100% CP022780_01078	IS3 ssgr IS51	1050202	1049327	876
SL3822	CP022780_00966	81.52% ISAisp2_aa1	100% CP022780_01079	IS3 ssgr IS51	1050477	1050199	279
SL3822	CP022780_01078	89.34% ISAisp2_aa2	100% CP022780_00965	IS3 ssgr IS51	1171505	1170630	876
SL3822	CP022780_01079	81.52% ISAisp2_aa1	100% CP022780_00966	IS3 ssgr IS51	1171780	1171502	279
SL3822	CP022780_01153	86.38% ISAisp2_aa2	94.94% CP022780_01078	IS3 ssgr IS51	1268073	1267282	792
SL3822	CP022780_01154	80.43% ISAisp2_aa1	96.73% CP022780_02533	IS3 ssgr IS51	1268348	1268070	279
SL3822	CP022780_02532	89.34% ISAisp2_aa2	98.28% CP022780_01078	IS3 ssgr IS51	2814157	2813282	876
SL3822	CP022780_02533	81.52% ISAisp2_aa1	97.82% CP022780_01079	IS3 ssgr IS51	2814432	2814154	279
SL3822	CP022780_00273	61.68% ISCro3_aa1	100% CP022780_02715	IS4	307571	308899	1329
SL3822	CP022780_00342	61.68% ISCro3_aa1	100% CP022780_02715	IS4	388186	386858	1329
SL3822	CP022780_00460	61.68% ISCro3_aa1	100% CP022780_02715	IS4	504712	503384	1329
SL3822	CP022780_00617	61.44% ISCro3_aa1	100% CP022780_01252	IS4	668654	669982	1329
SL3822	CP022780_00787	61.44% ISCro3_aa1	100% CP022780_01252	IS4	867553	866225	1329

SL3822	CP022780_01027	61.68% ISCro3_aa1	100% CP022780_02715	IS4	1116026	1114698	1329
SL3822	CP022780_01252	61.44% ISCro3_aa1	100% CP022780_00787	IS4	1377983	1379311	1329
SL3822	CP022780_01747	61.68% ISCro3_aa1	100% CP022780_03470	IS4	1904031	1905359	1329
SL3822	CP022780_02321	61.68% ISCro3_aa1	100% CP022780_03470	IS4	2585521	2584193	1329
SL3822	CP022780_02376	61.68% ISCro3_aa1	99.77% CP022780_02715	IS4	2641979	2640651	1329
SL3822	CP022780_02471	61.68% ISCro3_aa1	100% CP022780_03470	IS4	2735995	2737323	1329
SL3822	CP022780_02715	61.68% ISCro3_aa1	100% CP022780_01027	IS4	2998768	3000096	1329
SL3822	CP022780_03470	61.68% ISCro3_aa1	100% CP022780_02471	IS4	3799417	3798089	1329
SL3822	CP022780_01074	80.13% ISAzo5_aa1	99.65% CP022780_02563	IS4 ssgr IS50	1167177	1166107	1071
SL3822	CP022780_02496	78.03% ISAzo5_aa1	100% CP022780_02563	IS4 ssgr IS50	2767458	2766133	1326
SL3822	CP022780_02563	78.03% ISAzo5_aa1	100% CP022780_02496	IS4 ssgr IS50	2846660	2847985	1326
SL3822	CP022780_00253	93.43% ISRso1_aa1	94.52% CP022780_02026	IS5	280825	281646	822
SL3822	CP022780_00255	98.17% ISRso1_aa1	98.90% CP022780_02026	IS5	283539	284363	825
SL3822	CP022780_02026	97.81% ISRso1_aa1	98.17% CP022780_02514	IS5	2266764	2267588	825
SL3822	CP022780_02514	97.44% ISRso1_aa1	98.17% CP022780_02026	IS5	2793781	2794605	825
SL3822	CP022780_00248	85.93% ISAau3_aa1	80% CP022780_03179	IS5 ssgr IS5	27314	273409	270
SL3822	CP022780_00278	99.06% IS1405_aa1	100% CP022780_03179	IS5 ssgr IS5	313439	314404	966
SL3822	CP022780_00783	99.06% IS1405_aa1	100% CP022780_03179	IS5 ssgr IS5	855382	854417	966
SL3822	CP022780_00903	94.51% IS1021_aa1	100% CP022780_03230	IS5 ssgr IS5	982686	9817	987
SL3822	CP022780_01162	99.06% IS1405_aa1	100% CP022780_03179	IS5 ssgr IS5	1275798	1274833	966
SL3822	CP022780_01369	99.06% IS1405_aa1	100% CP022780_03179	IS5 ssgr IS5	1506169	1505204	966
SL3822	CP022780_02399	99.06% IS1405_aa1	100% CP022780_03179	IS5 ssgr IS5	2666342	2665377	966
SL3822	CP022780_02512	94.51% IS1021_aa1	100% CP022780_03230	IS5 ssgr IS5	2792176	2791190	987
SL3822	CP022780_02622	99.06% IS1405_aa1	100% CP022780_03179	IS5 ssgr IS5	2908955	2909920	966
SL3822	CP022780_02626	94.51% IS1021_aa1	100% CP022780_03230	IS5 ssgr IS5	2911611	2912597	987
SL3822	CP022780_03179	99.06% IS1405_aa1	100% CP022780_02622	IS5 ssgr IS5	3462483	3463448	966
SL3822	CP022780_03194	94.51% IS1021_aa1	100% CP022780_03230	IS5 ssgr IS5	3478777	3479763	987
SL3822	CP022780_03230	94.51% IS1021_aa1	100% CP022780_03194	IS5 ssgr IS5	3515745	3514759	987
SL3822	CP022780_02504	100% IS1420_aa1	100% CP022780_02629	IS5 ssgr IS903	2785400	2786356	957
SL3822	CP022780_02564	100% IS1420_aa1	100% CP022780_02629	IS5 ssgr IS903	2849039	2848083	957
SL3822	CP022780_02629	100% IS1420_aa1	100% CP022780_02564	IS5 ssgr IS903	2915023	2914067	957
SL3822	CP022780_00531	64.65% ISBcen19_aa1	No hit	IS66	577195	57759	396
SL3822	CP022780_00532	79.27% ISSal1_aa2	No hit	IS66	577578	577922	345
SL3822	CP022780_00533	87.18% ISBcen19_aa3	No hit	IS66	577988	57952	1533
SL3822	CP022780_00900	47.5% ISTha3_aa2	51.50% CP022780_03447	IS91	980156	97923	927

SL3822	CP022780_01629	40.65% ISShvi3_aa1	51.94% CP022780_01615	IS91	1780408	1779344	1065
SL3822	CP022780_03447	43.33% ISMno24_aa2	52.12% CP022780_00900	IS91	3776868	3777857	990
SL3822	CP022780_00036	38.97% ISKpn25_aa1	No hit	ISL3	49819	51264	1446
SL3822	CP022780_00543	53.43% ISSm4_aa1	No hit	ISL3	58739	589783	2394
SL3822	CP022780_00544	43.97% ISSm4_aa3	No hit	ISL3	589783	591186	1404
SL3822	CP022780_00545	49.68% ISSm4_aa4	No hit	ISL3	591173	594169	2997
SL3822	CP022780_01875	62.5% ISKpn21_aa1	No hit	ISNCY ssgr IS1202	2100747	2100346	402
SL3822	CP022780_00189	61.81% ISMpo10_aa3	73.20% CP022780_02498	Tn3	210684	209296	1389
SL3822	CP022780_01619	75.40% ISPa40_aa4	54.09% CP022780_00189	Tn3	1766648	1767244	597
SL3822	CP022780_02498	55.06% ISMpo10_aa3	98.26% CP022780_02557	Tn3	2769442	2768060	1383
SL3822	CP022780_02557	51.83% ISMpo10_aa3	98.26% CP022780_02498	Tn3	2838304	2836922	1383
HA4_1	CP022481_01079	95.51% ISBma3_aa1	100% CP022481_02860	IS110	1149563	1148358	1206
HA4_1	CP022481_02860	95.51% ISBma3_aa1	100% CP022481_01079	IS110	3096458	3095253	1206
HA4_1	CP022481_03014	95.51% ISBma3_aa1	99.00% CP022481_02860	IS110	3278277	3279482	1206
HA4_1	CP022481_02243	67.26% ISBj4_aa1	59.88% CP022481_03031	IS110 ssgr IS1111	2468829	2469872	1044
HA4_1	CP022481_03031	88.75% ISBcen4_aa1	59.88% CP022481_02243	IS110 ssgr IS1111	3295940	3294921	102
HA4_1	CP022481_00533	54.76% ISHpa1_aa1	No hit	IS1595 ssgr IS1016	564486	565265	780
HA4_1	CP022481_02207	49.48% ISHvo5_aa1	44.65% CP022481_02273	IS1595 ssgr ISH4	2423523	2422735	789
HA4_1	CP022481_00672	81.85% ISCARN95_aa1	100% CP022481_03176	IS21	712505	714028	1524
HA4_1	CP022481_00673	85.14% ISCARN95_aa2	100% CP022481_03175	IS21	714041	714808	768
HA4_1	CP022481_01896	85.14% ISCARN95_aa2	100% CP022481_03175	IS21	2106618	2105851	768
HA4_1	CP022481_01897	81.85% ISCARN95_aa1	100% CP022481_03176	IS21	2108154	2106631	1524
HA4_1	CP022481_02951	85.14% ISCARN95_aa2	100% CP022481_03175	IS21	3203772	3203005	768
HA4_1	CP022481_02952	81.85% ISCARN95_aa1	100% CP022481_03176	IS21	3205308	3203785	1524
HA4_1	CP022481_02965	93.61% ISRme9_aa1	100% CP022481_03368	IS21	3222523	3224076	1554
HA4_1	CP022481_02966	94.42% ISRme9_aa2	100% CP022481_03367	IS21	3224085	3224843	759
HA4_1	CP022481_03175	85.14% ISCARN95_aa2	100% CP022481_02951	IS21	3472618	3471851	768
HA4_1	CP022481_03176	81.85% ISCARN95_aa1	100% CP022481_02952	IS21	3474154	3472631	1524
HA4_1	CP022481_03367	94.42% ISRme9_aa2	100% CP022481_02966	IS21	3684971	3684213	759
HA4_1	CP022481_03368	93.61% ISRme9_aa1	100% CP022481_02965	IS21	3686533	3684980	1554
HA4_1	CP022481_02151	94.31% ISBcen18_aa1	No hit	IS256	2373147	2371870	1278
HA4_1	CP022481_00467	100% ISRso11_aa1	100% CP022481_01458	IS3 ssgr IS150	495916	496449	534
HA4_1	CP022481_00468	100% ISRso11_aa2	100% CP022481_01457	IS3 ssgr IS150	496683	497282	600
HA4_1	CP022481_00594	98.99% ISRso11_aa2	98.99% CP022481_01457	IS3 ssgr IS150	628662	628063	600

HA4_1	CP022481_00595	100% ISRso11_aa1	100% CP022481_01458	IS3 ssgr IS150	629429	628896	534
HA4_1	CP022481_00763	99.64% ISRso11_aa2	100% CP022481_01457	IS3 ssgr IS150	818246	81741	837
HA4_1	CP022481_00764	100% ISRso11_aa1	100% CP022481_01458	IS3 ssgr IS150	818776	818243	534
HA4_1	CP022481_01457	99.64% ISRso11_aa2	100% CP022481_00763	IS3 ssgr IS150	1625937	1625101	837
HA4_1	CP022481_01458	100% ISRso11_aa1	100% CP022481_00764	IS3 ssgr IS150	1626467	1625934	534
HA4_1	CP022481_02309	99.57% ISRso12_aa2	45.68% CP022481_01457	IS3 ssgr IS407	2541912	2541208	705
HA4_1	CP022481_02311	100% ISRso12_aa1	77.41% CP022481_02713	IS3 ssgr IS407	2543195	2542929	267
HA4_1	CP022481_02712	93.50% ISRso16_aa2	84.31% CP022481_02309	IS3 ssgr IS407	2953147	2952848	300
HA4_1	CP022481_02242	89.34% ISAisp2_aa2	52% CP022481_01457	IS3 ssgr IS51	2468732	2467857	876
HA4_1	CP022481_02244	78.04% ISAisp2_aa1	No hit	IS3 ssgr IS51	2470376	2469999	378
HA4_1	CP022481_00073	61.68% ISCro3_aa1	100% CP022481_03131	IS4	69491	68163	1329
HA4_1	CP022481_00112	61.44% ISCro3_aa1	99.77% CP022481_03131	IS4	107991	109319	1329
HA4_1	CP022481_00367	61.68% ISCro3_aa1	100% CP022481_03131	IS4	395631	394303	1329
HA4_1	CP022481_01027	61.44% ISCro3_aa1	100% CP022481_02797	IS4	1096662	1095334	1329
HA4_1	CP022481_01269	61.44% ISCro3_aa1	100% CP022481_02797	IS4	1359074	1357746	1329
HA4_1	CP022481_02004	61.68% ISCro3_aa1	100% CP022481_03131	IS4	2215991	2217319	1329
HA4_1	CP022481_02391	61.68% ISCro3_aa1	100% CP022481_03131	IS4	2624715	2623387	1329
HA4_1	CP022481_02767	61.68% ISCro3_aa1	100% CP022481_03131	IS4	3005130	3006458	1329
HA4_1	CP022481_02797	61.44% ISCro3_aa1	100% CP022481_01269	IS4	3031576	3032904	1329
HA4_1	CP022481_02852	61.68% ISCro3_aa1	100% CP022481_03131	IS4	3087156	3085828	1329
HA4_1	CP022481_03131	61.68% ISCro3_aa1	100% CP022481_02767	IS4	3414249	3412921	1329
HA4_1	CP022481_01534	87.30% IS1419_aa1	100% CP022481_01892	IS481	1713863	1712814	105
HA4_1	CP022481_01892	87.30% IS1419_aa1	100% CP022481_01534	IS481	2101548	2102597	105
HA4_1	CP022481_03171	39.80% ISGur11_aa1	No hit	IS481	3466291	3468237	1947
HA4_1	CP022481_01144	98.41% ISRso1_aa1	98.80% CP022481_01908	IS5	1223687	1224445	759
HA4_1	CP022481_01150	98.90% ISRso1_aa1	99.27% CP022481_03379	IS5	1234037	1233213	825
HA4_1	CP022481_01908	98.17% ISRso1_aa1	98.54% CP022481_02222	IS5	2120657	2121481	825
HA4_1	CP022481_02222	97.44% ISRso1_aa1	98.54% CP022481_01908	IS5	2446300	2447124	825
HA4_1	CP022481_02277	98.67% ISRso1_aa1	100% CP022481_01144	IS5	2514793	2514113	681
HA4_1	CP022481_02280	95.95% ISRso1_aa1	96.53% CP022481_01908	IS5	2516625	2516104	522
HA4_1	CP022481_03379	99.63% ISRso1_aa1	99.27% CP022481_01150	IS5	3698914	3699738	825
HA4_1	CP022481_03527	99.11% ISRso1_aa1	100% CP022481_01908	IS5	3850566	3849889	678
HA4_1	CP022481_00860	100% IS1421_aa1	100% CP022481_02948	IS5 ssgr IS427	922048	921644	405
HA4_1	CP022481_01030	86.06% IS1421_aa1	86.06% CP022481_02948	IS5 ssgr IS427	1097714	1097307	408
HA4_1	CP022481_01895	100% IS1421_aa1	100% CP022481_02948	IS5 ssgr IS427	2105591	2105187	405

HA4_1	CP022481_02236	100% IS1421_aa1	100% CP022481_02948	IS5 ssgr IS427	2463355	2462951	405
HA4_1	CP022481_02310	99.25% IS1421_aa1	99.25% CP022481_00860	IS5 ssgr IS427	2542120	2542524	405
HA4_1	CP022481_02629	100% IS1421_aa1	100% CP022481_02948	IS5 ssgr IS427	2866118	2866501	384
HA4_1	CP022481_02948	100% IS1421_aa1	100% CP022481_02236	IS5 ssgr IS427	3201451	3201855	405
HA4_1	CP022481_00300	94.51% IS1021_aa1	100% CP022481_01890	IS5 ssgr IS5	315047	314061	987
HA4_1	CP022481_00362	94.51% IS1021_aa1	100% CP022481_01890	IS5 ssgr IS5	384013	383027	987
HA4_1	CP022481_01051	94.51% IS1021_aa1	100% CP022481_01890	IS5 ssgr IS5	1117995	1118981	987
HA4_1	CP022481_01331	94.51% IS1021_aa1	100% CP022481_01890	IS5 ssgr IS5	1443569	1442583	987
HA4_1	CP022481_01890	94.51% IS1021_aa1	100% CP022481_02956	IS5 ssgr IS5	2098543	2100132	159
HA4_1	CP022481_01899	94.51% IS1021_aa1	100% CP022481_01890	IS5 ssgr IS5	2110186	2109200	987
HA4_1	CP022481_02233	94.51% IS1021_aa1	100% CP022481_01890	IS5 ssgr IS5	2461024	2460038	987
HA4_1	CP022481_02269	94.51% IS1021_aa1	100% CP022481_01890	IS5 ssgr IS5	2494665	2493679	987
HA4_1	CP022481_02588	94.51% IS1021_aa1	100% CP022481_01890	IS5 ssgr IS5	2820903	2821889	987
HA4_1	CP022481_02863	94.51% IS1021_aa1	100% CP022481_01890	IS5 ssgr IS5	3101670	3102656	987
HA4_1	CP022481_02942	94.51% IS1021_aa1	100% CP022481_01890	IS5 ssgr IS5	3193037	3192051	987
HA4_1	CP022481_02956	94.51% IS1021_aa1	100% CP022481_01890	IS5 ssgr IS5	3208429	3209415	987
HA4_1	CP022481_02212	99.45% IS1420_aa1	99.45% CP022481_02900	IS5 ssgr IS903	2430703	2431260	558
HA4_1	CP022481_02900	100% IS1420_aa1	99.45% CP022481_02212	IS5 ssgr IS903	3148340	3147384	957
HA4_1	CP022481_00478	47.5% ISTha3_aa2	51.50% CP022481_03100	IS91	508271	507345	927
HA4_1	CP022481_03100	43.33% ISMno24_aa2	52.12% CP022481_00478	IS91	3380976	3381965	990
HA4_1	CP022481_03216	38.97% ISKpn25_aa1	No hit	ISL3	3522766	3524211	1446
HA4_1	CP022481_03168	50.94% ISPa4_aa1	No hit	ISNCY	3460564	3462339	1776
HA4_1	CP022481_01395	65.48% ISKpn21_aa1	83.33% CP022481_01396	ISNCY ssgr IS1202	1560307	1559915	393
HA4_1	CP022481_01396	72.27% ISKpn21_aa1	83.33% CP022481_01395	ISNCY ssgr IS1202	1561933	1560458	1476
HA4_1	CP022481_00667	61.87% ISPa38_aa1	54.90% CP022481_03373	Tn3	706863	707453	591
HA4_1	CP022481_02121	54.43% ISMpo10_aa3	94.51% CP022481_02271	Tn3	2338629	2337250	138
HA4_1	CP022481_02205	54.43% ISMpo10_aa3	98.47% CP022481_02271	Tn3	2421217	2419835	1383
HA4_1	CP022481_02271	54.43% ISMpo10_aa3	98.47% CP022481_02205	Tn3	2497845	2496463	1383
HA4_1	CP022481_03373	55.34% ISMpo10_aa3	72.59% CP022481_02271	Tn3	3691755	3690367	1389
KACC10709	IS_3588bd79_00251	61.67% ISMno14_aa1	45.80% IS_3588bd79_03191	IS110 ssgr IS1111	245288	246301	1014
KACC10709	IS_3588bd79_02825	88.75% ISBcen4_aa1	100% IS_3588bd79_03191	IS110 ssgr IS1111	3004655	3003636	102
KACC10709	IS_3588bd79_03191	88.75% ISBcen4_aa1	100% IS_3588bd79_02825	IS110 ssgr IS1111	3393872	3394891	102
KACC10709	IS_3588bd79_00156	83.75% ISBusp4_aa1	100% IS_3588bd79_01994	IS1182	159001	157556	1446
KACC10709	IS_3588bd79_00463	83.75% ISBusp4_aa1	100% IS_3588bd79_01994	IS1182	464235	46568	1446

KACC10709	IS_3588bd79_00880	83.75% ISBusp4_aa1	100% IS_3588bd79_01994	IS1182	898875	89743	1446
KACC10709	IS_3588bd79_01686	82.65% ISBusp4_aa1	100% IS_3588bd79_01994	IS1182	1769849	1768599	1251
KACC10709	IS_3588bd79_01994	83.75% ISBusp4_aa1	100% IS_3588bd79_00880	IS1182	2104232	2105677	1446
KACC10709	IS_3588bd79_02098	54.76% ISHpa1_aa1	No hit	IS1595 ssgr IS1016	2216077	2215298	780
KACC10709	IS_3588bd79_02794	50.44% ISLsp2_aa1	48.34% IS_3588bd79_00676	IS1595 ssgr ISPna2	2962457	2961180	1278
KACC10709	IS_3588bd79_02164	99.64% ISRso11_aa2	100% IS_3588bd79_02732	IS3 ssgr IS150	2283796	2282960	837
KACC10709	IS_3588bd79_02165	100% ISRso11_aa1	100% IS_3588bd79_02733	IS3 ssgr IS150	2284326	2283793	534
KACC10709	IS_3588bd79_02732	99.64% ISRso11_aa2	100% IS_3588bd79_02164	IS3 ssgr IS150	2886521	2885685	837
KACC10709	IS_3588bd79_02733	100% ISRso11_aa1	100% IS_3588bd79_02165	IS3 ssgr IS150	2887051	2886518	534
KACC10709	IS_3588bd79_02824	99.64% ISRso10_aa2	40% IS_3588bd79_02732	IS3 ssgr IS2	3003260	3002412	849
KACC10709	IS_3588bd79_02826	97.87% ISRso10_aa1	96.96% IS_3588bd79_03190	IS3 ssgr IS2	3005010	3004630	381
KACC10709	IS_3588bd79_01954	91.46% IS222_aa2	55.22% IS_3588bd79_02732	IS3 ssgr IS3	2064537	2064067	471
KACC10709	IS_3588bd79_01990	81.52% ISAisp2_aa1	No hit	IS3 ssgr IS51	2101614	2101892	279
KACC10709	IS_3588bd79_01991	89% ISAisp2_aa2	51.70% IS_3588bd79_02732	IS3 ssgr IS51	2101889	2102551	663
KACC10709	IS_3588bd79_00210	61.68% ISCro3_aa1	100% IS_3588bd79_03095	IS4	206954	208282	1329
KACC10709	IS_3588bd79_00565	61.68% ISCro3_aa1	100% IS_3588bd79_03101	IS4	565645	564317	1329
KACC10709	IS_3588bd79_00796	61.68% ISCro3_aa1	100% IS_3588bd79_03095	IS4	810486	811814	1329
KACC10709	IS_3588bd79_00854	59.68% ISCro3_aa1	100% IS_3588bd79_03095	IS4	8685	86945	951
KACC10709	IS_3588bd79_01339	61.68% ISCro3_aa1	100% IS_3588bd79_03101	IS4	1417785	1416457	1329
KACC10709	IS_3588bd79_01873	61.68% ISCro3_aa1	100% IS_3588bd79_03095	IS4	1968269	1969597	1329
KACC10709	IS_3588bd79_02040	61.68% ISCro3_aa1	100% IS_3588bd79_03095	IS4	2154965	2156293	1329
KACC10709	IS_3588bd79_02523	61.68% ISCro3_aa1	100% IS_3588bd79_03095	IS4	2675585	2674257	1329
KACC10709	IS_3588bd79_02562	61.68% ISCro3_aa1	100% IS_3588bd79_03101	IS4	2714085	2715413	1329
KACC10709	IS_3588bd79_02698	61.68% ISCro3_aa1	100% IS_3588bd79_03095	IS4	2847143	2848471	1329
KACC10709	IS_3588bd79_02771	61.68% ISCro3_aa1	100% IS_3588bd79_03095	IS4	2932519	2931191	1329
KACC10709	IS_3588bd79_03064	61.68% ISCro3_aa1	100% IS_3588bd79_03101	IS4	3241014	3242342	1329
KACC10709	IS_3588bd79_03095	61.68% ISCro3_aa1	100% IS_3588bd79_02771	IS4	3279370	3280698	1329
KACC10709	IS_3588bd79_03101	61.68% ISCro3_aa1	100% IS_3588bd79_03064	IS4	3288684	3287356	1329
KACC10709	IS_3588bd79_00853	66.66% ISCro6_aa1	100% IS_3588bd79_03095	IS4 ssgr IS4	868122	8684	279
KACC10709	IS_3588bd79_02783	97.81% ISRso1_aa1	100% IS_3588bd79_02805	IS5	2948917	2949741	825
KACC10709	IS_3588bd79_02805	97.81% ISRso1_aa1	100% IS_3588bd79_02783	IS5	2983391	2982567	825
KACC10709	IS_3588bd79_00031	94.81% IS1021_aa1	100% IS_3588bd79_03059	IS5 ssgr IS5	32054	31068	987
KACC10709	IS_3588bd79_00071	99.37% IS1405_aa1	100% IS_3588bd79_02766	IS5 ssgr IS5	78016	77051	966
KACC10709	IS_3588bd79_00145	94.81% IS1021_aa1	100% IS_3588bd79_03059	IS5 ssgr IS5	145678	146664	987
KACC10709	IS_3588bd79_00152	99.37% IS1405_aa1	100% IS_3588bd79_02766	IS5 ssgr IS5	152398	153363	966

KACC10709	IS_3588bd79_00248	94.81% IS1021_aa1	100% IS_3588bd79_03059	IS5 ssgr IS5	244096	24311	987
KACC10709	IS_3588bd79_00368	99.06% IS1405_aa1	100% IS_3588bd79_03255	IS5 ssgr IS5	367898	368863	966
KACC10709	IS_3588bd79_00462	99.37% IS1405_aa1	100% IS_3588bd79_02766	IS5 ssgr IS5	463711	462746	966
KACC10709	IS_3588bd79_00646	99.37% IS1405_aa1	100% IS_3588bd79_02766	IS5 ssgr IS5	645329	644364	966
KACC10709	IS_3588bd79_00852	99.06% IS1405_aa1	100% IS_3588bd79_03255	IS5 ssgr IS5	867975	86701	966
KACC10709	IS_3588bd79_01756	98.75% IS1405_aa1	99.68% IS_3588bd79_03255	IS5 ssgr IS5	1841327	1842292	966
KACC10709	IS_3588bd79_02688	93.50% IS1021_aa1	100% IS_3588bd79_03059	IS5 ssgr IS5	2838485	2838252	234
KACC10709	IS_3588bd79_02766	99.37% IS1405_aa1	100% IS_3588bd79_00646	IS5 ssgr IS5	2926651	2925686	966
KACC10709	IS_3588bd79_02786	99.06% IS1405_aa1	100% IS_3588bd79_03255	IS5 ssgr IS5	2951726	2950761	966
KACC10709	IS_3588bd79_02793	92.07% ISRso9_aa1	No hit	IS5 ssgr IS5	2960814	2960509	306
KACC10709	IS_3588bd79_02833	99.06% IS1405_aa1	100% IS_3588bd79_03255	IS5 ssgr IS5	3009258	3008293	966
KACC10709	IS_3588bd79_03059	94.81% IS1021_aa1	100% IS_3588bd79_00248	IS5 ssgr IS5	3235159	3236145	987
KACC10709	IS_3588bd79_03255	99.06% IS1405_aa1	100% IS_3588bd79_02833	IS5 ssgr IS5	3455728	3454763	966
KACC10709	IS_3588bd79_00016	100% IS1420_aa1	100% IS_3588bd79_03173	IS5 ssgr IS903	12793	13749	957
KACC10709	IS_3588bd79_00419	100% IS1420_aa1	100% IS_3588bd79_03173	IS5 ssgr IS903	421039	420083	957
KACC10709	IS_3588bd79_00666	99.68% IS1420_aa1	99.68% IS_3588bd79_03173	IS5 ssgr IS903	666876	66592	957
KACC10709	IS_3588bd79_00674	100% IS1420_aa1	100% IS_3588bd79_03173	IS5 ssgr IS903	68264	683596	957
KACC10709	IS_3588bd79_00774	100% IS1420_aa1	100% IS_3588bd79_03173	IS5 ssgr IS903	787229	786273	957
KACC10709	IS_3588bd79_01589	100% IS1420_aa1	100% IS_3588bd79_03173	IS5 ssgr IS903	1672018	1672974	957
KACC10709	IS_3588bd79_01668	100% IS1420_aa1	100% IS_3588bd79_03173	IS5 ssgr IS903	1753152	1752196	957
KACC10709	IS_3588bd79_02762	100% IS1420_aa1	100% IS_3588bd79_03173	IS5 ssgr IS903	2922806	2923660	855
KACC10709	IS_3588bd79_02764	100% IS1420_aa1	100% IS_3588bd79_03173	IS5 ssgr IS903	2925029	2924073	957
KACC10709	IS_3588bd79_02834	100% IS1420_aa1	100% IS_3588bd79_03173	IS5 ssgr IS903	3010638	3009682	957
KACC10709	IS_3588bd79_03061	100% IS1420_aa1	100% IS_3588bd79_03173	IS5 ssgr IS903	3236673	3237629	957
KACC10709	IS_3588bd79_03173	100% IS1420_aa1	100% IS_3588bd79_03061	IS5 ssgr IS903	3371747	3370791	957
KACC10709	IS_3588bd79_00139	56.95% ISRm2_aa1	100% IS_3588bd79_02819	IS66	14048	140962	483
KACC10709	IS_3588bd79_00140	73.27% ISRm2_aa2	100% IS_3588bd79_02818	IS66	140959	141312	354
KACC10709	IS_3588bd79_00141	59.79% ISAeh1_aa2	100% IS_3588bd79_02817	IS66	141345	142901	1557
KACC10709	IS_3588bd79_00654	59.79% ISAeh1_aa2	100% IS_3588bd79_02817	IS66	65383	652274	1557
KACC10709	IS_3588bd79_00655	73.27% ISRm2_aa2	100% IS_3588bd79_02818	IS66	654216	653863	354
KACC10709	IS_3588bd79_00656	56.95% ISRm2_aa1	100% IS_3588bd79_02819	IS66	654695	654213	483
KACC10709	IS_3588bd79_01506	59.79% ISAeh1_aa2	100% IS_3588bd79_02817	IS66	1583016	1581460	1557
KACC10709	IS_3588bd79_01507	73.27% ISRm2_aa2	100% IS_3588bd79_02818	IS66	1583402	1583049	354
KACC10709	IS_3588bd79_01508	56.95% ISRm2_aa1	100% IS_3588bd79_02819	IS66	1583881	1583399	483
KACC10709	IS_3588bd79_02269	56.95% ISRm2_aa1	100% IS_3588bd79_02819	IS66	2386813	2387295	483

KACC10709	IS_3588bd79_02270	73.27% ISRm2_aa2	100% IS_3588bd79_02818	IS66	2387292	2387645	354
KACC10709	IS_3588bd79_02271	59.79% ISAeh1_aa2	100% IS_3588bd79_02817	IS66	2387678	2389234	1557
KACC10709	IS_3588bd79_02812	60.71% ISAba28_aa4	No hit	IS66	2989040	2989351	312
KACC10709	IS_3588bd79_02813	77.95% IS883_aa3	54.19% IS_3588bd79_02817	IS66	2989796	2989332	465
KACC10709	IS_3588bd79_02814	72.46% ISEc8_aa3	43.72% IS_3588bd79_02817	IS66	2990426	2989800	627
KACC10709	IS_3588bd79_02815	63.38% IS883_aa3	No hit	IS66	2990935	2990366	570
KACC10709	IS_3588bd79_02816	86.84% IS883_aa2	53.98% IS_3588bd79_02818	IS66	2991391	2990975	417
KACC10709	IS_3588bd79_02817	59.79% ISAeh1_aa2	100% IS_3588bd79_02271	IS66	2992920	2991364	1557
KACC10709	IS_3588bd79_02818	73.27% ISRm2_aa2	100% IS_3588bd79_02270	IS66	2993306	2992953	354
KACC10709	IS_3588bd79_02819	56.95% ISRm2_aa1	100% IS_3588bd79_02269	IS66	2993785	2993303	483
KACC10709	IS_3588bd79_01274	42.78% ISWz1_aa1	100% IS_3588bd79_02377	IS91	1339363	1337690	1674
KACC10709	IS_3588bd79_02154	47.5% ISTha3_aa2	51.50% IS_3588bd79_03123	IS91	2271981	2272907	927
KACC10709	IS_3588bd79_02377	42.78% ISWz1_aa1	100% IS_3588bd79_01274	IS91	2511389	2513062	1674
KACC10709	IS_3588bd79_03123	43.33% ISMno24_aa2	52.12% IS_3588bd79_02154	IS91	3309969	3308980	990
KACC10709	IS_3588bd79_03037	38.97% ISKpn25_aa1	No hit	ISL3	3212046	3210601	1446
KACC10709	IS_3588bd79_00678	55.06% ISMpo10_aa3	73.20% IS_3588bd79_02883	Tn3	68798	689362	1383
KACC10709	IS_3588bd79_01277	75.15% ISPa42_aa1	100% IS_3588bd79_02374	Tn3	1343964	1341019	2946
KACC10709	IS_3588bd79_02374	75.15% ISPa42_aa1	100% IS_3588bd79_01277	Tn3	2506789	2509734	2946
KACC10709	IS_3588bd79_02883	54.08% ISMpo10_aa3	73.20% IS_3588bd79_00678	Tn3	3046321	3047709	1389
CMR15	IS_8710be73_01399	89.09% ISPath1_aa1	96.36% IS_8710be73_03080	IS110 ssgr IS1111	1511207	1510506	702
CMR15	IS_8710be73_02825	92.85% ISPath1_aa1	100% IS_8710be73_03080	IS110 ssgr IS1111	3015884	3014802	1083
CMR15	IS_8710be73_03080	92.85% ISPath1_aa1	100% IS_8710be73_02825	IS110 ssgr IS1111	3282645	3283727	1083
CMR15	IS_8710be73_03229	92.13% ISPath1_aa1	100% IS_8710be73_03080	IS110 ssgr IS1111	3444359	3444895	537
CMR15	IS_8710be73_03230	91.03% ISAeca3_aa1	100% IS_8710be73_03080	IS110 ssgr IS1111	3444905	3445387	483
CMR15	IS_8710be73_00283	91.46% ISBma2_aa1	100% IS_8710be73_03346	IS1182	335351	333867	1485
CMR15	IS_8710be73_00477	91.68% ISBma2_aa1	100% IS_8710be73_01872	IS1182	528232	526748	1485
CMR15	IS_8710be73_00678	90.67% ISBma2_aa1	100% IS_8710be73_03336	IS1182	741975	742748	774
CMR15	IS_8710be73_00679	92.07% ISBma2_aa1	100% IS_8710be73_00824	IS1182	742741	74346	720
CMR15	IS_8710be73_00824	91.68% ISBma2_aa1	100% IS_8710be73_01872	IS1182	908391	906868	1524
CMR15	IS_8710be73_00833	91.68% ISBma2_aa1	100% IS_8710be73_03336	IS1182	914681	916165	1485
CMR15	IS_8710be73_00861	91.68% ISBma2_aa1	100% IS_8710be73_03336	IS1182	943399	944883	1485
CMR15	IS_8710be73_00913	91.68% ISBma2_aa1	100% IS_8710be73_03336	IS1182	993049	994533	1485
CMR15	IS_8710be73_00939	91.46% ISBma2_aa1	98.65% IS_8710be73_01872	IS1182	1019231	1020796	1566
CMR15	IS_8710be73_01062	92.20% ISBma2_aa1	100% IS_8710be73_02138	IS1182	1152126	1151551	576

CMR15	IS_8710be73_01063	91.18% ISBma2_aa1	99.61% IS_8710be73_02138	IS1182	1153037	1152126	912
CMR15	IS_8710be73_01850	91.68% ISBma2_aa1	100% IS_8710be73_03336	IS1182	2017452	2018936	1485
CMR15	IS_8710be73_01857	91.68% ISBma2_aa1	100% IS_8710be73_03336	IS1182	2026702	2025218	1485
CMR15	IS_8710be73_01872	91.68% ISBma2_aa1	98.65% IS_8710be73_00939	IS1182	2042867	2044444	1578
CMR15	IS_8710be73_01905	91.46% ISBma2_aa1	100% IS_8710be73_03346	IS1182	2077989	2079473	1485
CMR15	IS_8710be73_02138	91.68% ISBma2_aa1	100% IS_8710be73_01872	IS1182	2292728	2291244	1485
CMR15	IS_8710be73_02284	91.46% ISBma2_aa1	99.59% IS_8710be73_00824	IS1182	2458962	2457478	1485
CMR15	IS_8710be73_02444	91.24% ISBma2_aa1	99.59% IS_8710be73_03336	IS1182	2627696	2629180	1485
CMR15	IS_8710be73_02673	91.46% ISBma2_aa1	100% IS_8710be73_03346	IS1182	2855409	2856893	1485
CMR15	IS_8710be73_02804	91.68% ISBma2_aa1	100% IS_8710be73_03336	IS1182	2994993	2993509	1485
CMR15	IS_8710be73_03336	91.68% ISBma2_aa1	100% IS_8710be73_01857	IS1182	3563054	3561570	1485
CMR15	IS_8710be73_03346	91.46% ISBma2_aa1	100% IS_8710be73_02673	IS1182	3572759	3574243	1485
CMR15	IS_8710be73_02802	66.66% ISNme3_aa1	No hit	IS1595 ssgr IS1016	2992855	2992604	252
CMR15	IS_8710be73_00398	60.75% ISCro3_aa1	100% IS_8710be73_03114	IS4	445962	44464	1323
CMR15	IS_8710be73_00531	60.75% ISCro3_aa1	100% IS_8710be73_03114	IS4	579673	580995	1323
CMR15	IS_8710be73_00943	60.75% ISCro3_aa1	100% IS_8710be73_03114	IS4	1024684	1026006	1323
CMR15	IS_8710be73_01113	60.75% ISCro3_aa1	100% IS_8710be73_03114	IS4	1212079	1210757	1323
CMR15	IS_8710be73_01360	61.20% ISCro3_aa1	100% IS_8710be73_02593	IS4	1466893	1465565	1329
CMR15	IS_8710be73_01897	61.20% ISCro3_aa1	100% IS_8710be73_02593	IS4	2069571	2070899	1329
CMR15	IS_8710be73_02593	61.20% ISCro3_aa1	100% IS_8710be73_01897	IS4	2781667	2780339	1329
CMR15	IS_8710be73_03114	60.75% ISCro3_aa1	100% IS_8710be73_01113	IS4	3315369	3316691	1323
CMR15	IS_8710be73_00203	97.42% ISRso1_aa1	55% IS_8710be73_01552	IS5	236254	235622	633
CMR15	IS_8710be73_01552	88.37% ISAzo23_aa1	55% IS_8710be73_00203	IS5 ssgr IS1031	1679841	1679035	807
CMR15	IS_8710be73_03042	74.13% ISRm2_aa2	No hit	IS66	3239379	3239732	354
CMR15	IS_8710be73_03043	60.74% ISPpu13_aa2	67.39% IS_8710be73_01577	IS66	3239765	3241321	1557
CMR15	IS_8710be73_00793	48.22% ISTha3_aa2	52.05% IS_8710be73_03304	IS91	871533	870598	936
CMR15	IS_8710be73_03304	43.53% ISMno24_aa2	52.05% IS_8710be73_00793	IS91	3528345	3529334	990
CMR15	IS_8710be73_00012	74.17% ISSpu3_aa1	100% IS_8710be73_03056	ISAs1	15602	14508	1095
CMR15	IS_8710be73_00015	74.17% ISSpu3_aa1	100% IS_8710be73_03056	ISAs1	19308	20402	1095
CMR15	IS_8710be73_01211	74.17% ISSpu3_aa1	100% IS_8710be73_03056	ISAs1	1316251	1315157	1095
CMR15	IS_8710be73_01381	74.17% ISSpu3_aa1	100% IS_8710be73_03056	ISAs1	1488096	1487002	1095
CMR15	IS_8710be73_02685	74.17% ISSpu3_aa1	100% IS_8710be73_03056	ISAs1	2873717	2874811	1095
CMR15	IS_8710be73_03056	74.17% ISSpu3_aa1	100% IS_8710be73_02685	ISAs1	3258453	3257359	1095
CMR15	IS_8710be73_00017	38.87% ISKpn25_aa3	36.99% IS_8710be73_03046	ISL3	24481	21191	3291
CMR15	IS_8710be73_00019	44.32% ISKpn25_aa1	44% IS_8710be73_03048	ISL3	27461	25851	1611

CMR15	IS_8710be73_00063	38.58% ISKpn25_aa1	38.84% IS_8710be73_00019	ISL3	8294	84385	1446
CMR15	IS_8710be73_02523	92% ISSm4_aa2	No hit	ISL3	2711825	2711409	417
CMR15	IS_8710be73_03046	68.59% ISKpn25_aa3	36.99% IS_8710be73_00017	ISL3	3246718	3243503	3216
CMR15	IS_8710be73_03047	50.88% ISKpn25_aa2	No hit	ISL3	3248052	3246715	1338
CMR15	IS_8710be73_03048	65.36% ISKpn25_aa1	43.87% IS_8710be73_00019	ISL3	3249974	3248049	1926
CMR15	IS_8710be73_02486	49.28% ISKpn21_aa1	No hit	ISNCY ssgr IS1202	2675516	2675860	345
CMR15	IS_8710be73_00626	52.59% TnShfr1_aa1	56.62% IS_8710be73_00298	Tn3	687084	688304	1221
CMR15	IS_8710be73_01013	62.43% ISPa38_aa1	56.20% IS_8710be73_01794	Tn3	1102435	1103025	591
CMR15	IS_8710be73_01794	53.65% ISMpo10_aa3	97.33% IS_8710be73_02548	Tn3	1960772	1962154	1383
RS476	CP021762_03231	49.48% ISHvo5_aa1	44.65% CP021762_00902	IS1595 ssgr ISH4	3439175	3438387	789
RS476	CP021762_00250	100% ISRso6_aa1	100% CP021762_02370	IS21	275253	276296	1.044
RS476	CP021762_00251	100% ISRso6_aa2	100% CP021762_02371	IS21	276293	277138	846
RS476	CP021762_02370	100% ISRso6_aa1	100% CP021762_00250	IS21	2531684	2532727	1.044
RS476	CP021762_02371	100% ISRso6_aa2	100% CP021762_00251	IS21	2532724	2533569	846
RS476	CP021762_00254	100% ISRso7_aa1	No hit	IS256	282832	284082	1.251
RS476	CP021762_00593	100% ISRso8_aa2	100% CP021762_02302	IS3	623171	622281	891
RS476	CP021762_00594	100% ISRso8_aa1	100% CP021762_02303	IS3	623461	623168	294
RS476	CP021762_01580	100% ISRso8_aa2	100% CP021762_02302	IS3	1662653	1661763	891
RS476	CP021762_01581	100% ISRso8_aa1	100% CP021762_02303	IS3	1662943	1662650	294
RS476	CP021762_02302	100% ISRso8_aa2	100% CP021762_01580	IS3	2458969	2458079	891
RS476	CP021762_02303	100% ISRso8_aa1	100% CP021762_01581	IS3	2459259	2458966	294
RS476	CP021762_01464	100% ISRso11_aa2	100% CP021762_03140	IS3 ssgr IS150	1541023	1540187	837
RS476	CP021762_01465	100% ISRso11_aa1	100% CP021762_03141	IS3 ssgr IS150	1541553	1541020	534
RS476	CP021762_02300	100% ISRso11_aa2	100% CP021762_03140	IS3 ssgr IS150	2457517	2456681	837
RS476	CP021762_02301	100% ISRso11_aa1	100% CP021762_03141	IS3 ssgr IS150	2457942	2457514	429
RS476	CP021762_02447	99.64% ISRso11_aa2	99.64% CP021762_03140	IS3 ssgr IS150	2614096	2613260	837
RS476	CP021762_02448	100% ISRso11_aa1	100% CP021762_03141	IS3 ssgr IS150	2614626	2614093	534
RS476	CP021762_03140	100% ISRso11_aa2	100% CP021762_02300	IS3 ssgr IS150	3327289	3326453	837
RS476	CP021762_03141	100% ISRso11_aa1	100% CP021762_02448	IS3 ssgr IS150	3327819	3327286	534
RS476	CP021762_01462	100% ISRso10_aa1	100% CP021762_01869	IS3 ssgr IS2	1538856	1539245	390
RS476	CP021762_01463	100% ISRso10_aa2	100% CP021762_02368	IS3 ssgr IS2	1539242	1540090	849
RS476	CP021762_01868	100% ISRso10_aa2	100% CP021762_02368	IS3 ssgr IS2	2002392	2001544	849
RS476	CP021762_01869	100% ISRso10_aa1	100% CP021762_01462	IS3 ssgr IS2	2002778	2002389	390
RS476	CP021762_02368	100% ISRso10_aa2	100% CP021762_01868	IS3 ssgr IS2	2531322	2530474	849

RS476	CP021762_02369	100% ISRso10_aa1	100% CP021762_01869	IS3 ssgr IS2	2531564	2531319	246
RS476	CP021762_02349	89.23% IS222_aa2	57.69% CP021762_02302	IS3 ssgr IS3	2514900	2514229	672
RS476	CP021762_02350	87.25% IS222_aa1	No hit	IS3 ssgr IS3	2515391	2515083	309
RS476	CP021762_00641	87.67% ISSme1_aa3	80.28% CP021762_02445	IS3 ssgr IS407	670983	67063	354
RS476	CP021762_00842	100% ISRso14_aa2	100% CP021762_02444	IS3 ssgr IS407	870513	869965	549
RS476	CP021762_00843	100% ISRso14_aa1	100% CP021762_02445	IS3 ssgr IS407	871082	870819	264
RS476	CP021762_01466	100% ISRso12_aa1	74.11% CP021762_02445	IS3 ssgr IS407	1541817	1542083	267
RS476	CP021762_01467	100% ISRso12_aa2	100% CP021762_02744	IS3 ssgr IS407	1542233	1542937	705
RS476	CP021762_01522	100% ISRso14_aa2	100% CP021762_02444	IS3 ssgr IS407	1597556	1597008	549
RS476	CP021762_01523	100% ISRso14_aa1	100% CP021762_02445	IS3 ssgr IS407	1598125	1597862	264
RS476	CP021762_02444	100% ISRso14_aa2	100% CP021762_01522	IS3 ssgr IS407	2612271	2611723	549
RS476	CP021762_02445	100% ISRso14_aa1	100% CP021762_01523	IS3 ssgr IS407	2612840	2612577	264
RS476	CP021762_02744	100% ISRso12_aa2	100% CP021762_01467	IS3 ssgr IS407	2911702	2910998	705
RS476	CP021762_01579	75.26% ISBcen21_aa1	54.54% CP021762_02303	IS3 ssgr IS51	1661337	1661624	288
RS476	CP021762_01513	100% ISRso13_aa1	100% CP021762_03286	IS4 ssgr IS4	1590765	1589431	1.335
RS476	CP021762_01903	100% ISRso13_aa1	100% CP021762_03286	IS4 ssgr IS4	2047953	2049287	1.335
RS476	CP021762_03221	100% ISRso13_aa1	100% CP021762_03286	IS4 ssgr IS4	3427567	3428901	1.335
RS476	CP021762_03286	100% ISRso13_aa1	100% CP021762_03221	IS4 ssgr IS4	3500158	3498824	1.335
RS476	CP021762_00210	99.27% ISRso1_aa1	100% CP021762_03397	IS5	231833	232732	900
RS476	CP021762_01020	98.00% ISRso1_aa1	98.80% CP021762_03397	IS5	1060268	1059513	756
RS476	CP021762_03245	97.44% ISRso1_aa1	97.44% CP021762_03397	IS5	3460632	3461456	825
RS476	CP021762_03266	99.10% ISRso1_aa1	99.10% CP021762_03397	IS5	3483508	3484185	678
RS476	CP021762_03397	99.27% ISRso1_aa1	100% CP021762_00210	IS5	3612874	3612050	825
RS476	CP021762_00906	76.56% ISCARN14_aa1	51.10% CP021762_00210	IS5 ssgr IS1031	940704	939526	1.179
RS476	CP021762_01707	100% IS1421_aa1	No hit	IS5 ssgr IS427	1793013	1792609	405
RS476	CP021762_00856	100% ISRso9_aa1	100% CP021762_03467	IS5 ssgr IS5	88333	884652	1.323
RS476	CP021762_01745	100% ISRso18_aa1	No hit	IS5 ssgr IS5	1821237	1820272	966
RS476	CP021762_03175	100% ISRso9_aa1	100% CP021762_03467	IS5 ssgr IS5	3367104	3365782	1.323
RS476	CP021762_03197	100% ISRso9_aa1	100% CP021762_03467	IS5 ssgr IS5	3396692	3395370	1.323
RS476	CP021762_03467	100% ISRso9_aa1	100% CP021762_03197	IS5 ssgr IS5	3697034	3695712	1.323
RS476	CP021762_00104	100% ISRso5_aa1	100% CP021762_03440	IS630	117986	119077	1.092
RS476	CP021762_00110	99.72% ISRso5_aa1	99.72% CP021762_03440	IS630	127897	128988	1.092
RS476	CP021762_00655	100% ISRso5_aa1	100% CP021762_03440	IS630	683427	684518	1.092
RS476	CP021762_02210	100% ISRso5_aa1	100% CP021762_03440	IS630	2360192	2361283	1.092
RS476	CP021762_03440	100% ISRso5_aa1	100% CP021762_02210	IS630	3660484	3659393	1.092

RS476	CP021762_00054	43.33% ISMno24_aa2	52.12% CP021762_02583	IS91	58345	57356	990
RS476	CP021762_01585	40.16% ISShvi3_aa1	52.96% CP021762_01692	IS91	1670802	1669738	1.065
RS476	CP021762_02583	48.21% ISTha3_aa2	51.50% CP021762_00054	IS91	2752052	2752978	927
RS476	CP021762_02612	100% ISRso15_aa1	No hit	ISL3	2780172	2781392	1.221
RS476	CP021762_02659	77.94% ISSm4_aa2	No hit	ISL3	2824613	2823546	1.068
RS476	CP021762_03443	38.97% ISKpn25_aa1	No hit	ISL3	3665755	3664310	1.446
RS476	CP021762_01882	64.16% ISKpn21_aa1	92.5% CP021762_01883	ISNCY ssgr IS1202	2023646	2023254	393
RS476	CP021762_01883	71.39% ISKpn21_aa1	92.5% CP021762_01882	ISNCY ssgr IS1202	2025275	2023800	1.476
RS476	CP021762_00904	54.43% ISMpo10_aa3	72.74% CP021762_03291	Tn3	937578	93896	1.383
RS476	CP021762_03224	61.81% ISMpo10_aa3	97.40% CP021762_03291	Tn3	3430405	3431793	1.389
RS476	CP021762_03291	55.34% ISMpo10_aa3	97.40% CP021762_03224	Tn3	3502822	3504210	1.389

CRMRs218	CP021764_03249	49.48% ISHvo5_aa1	44.65% CP021764_00907	IS1595 ssgr ISH4	3439477	3438689	789
CRMRs218	CP021764_00250	100% ISRso6_aa1	100% CP021764_02381	IS21	275316	276359	1.044
CRMRs218	CP021764_00251	100% ISRso6_aa2	100% CP021764_02382	IS21	276356	277201	846
CRMRs218	CP021764_02381	100% ISRso6_aa1	100% CP021764_00250	IS21	2531806	2532849	1.044
CRMRs218	CP021764_02382	100% ISRso6_aa2	100% CP021764_00251	IS21	2532846	2533691	846
CRMRs218	CP021764_00255	95.43% ISRso7_aa1	No hit	IS256	282895	284145	1.251
CRMRs218	CP021764_00592	100% ISRso8_aa2	100% CP021764_02313	IS3	623236	622346	891
CRMRs218	CP021764_00593	100% ISRso8_aa1	100% CP021764_02314	IS3	623526	623233	294
CRMRs218	CP021764_01590	100% ISRso8_aa2	100% CP021764_02313	IS3	1662560	1661670	891
CRMRs218	CP021764_01591	100% ISRso8_aa1	100% CP021764_02314	IS3	1662850	1662557	294
CRMRs218	CP021764_02313	100% ISRso8_aa2	100% CP021764_01590	IS3	2459097	2458207	891
CRMRs218	CP021764_02314	100% ISRso8_aa1	100% CP021764_01591	IS3	2459387	2459094	294
CRMRs218	CP021764_01474	100% ISRso11_aa2	100% CP021764_03154	IS3 ssgr IS150	1540951	1540115	837
CRMRs218	CP021764_01475	100% ISRso11_aa1	100% CP021764_03155	IS3 ssgr IS150	1541481	1540948	534
CRMRs218	CP021764_02311	100% ISRso11_aa2	100% CP021764_03154	IS3 ssgr IS150	2457645	2456809	837
CRMRs218	CP021764_02312	100% ISRso11_aa1	100% CP021764_03155	IS3 ssgr IS150	2458070	2457642	429
CRMRs218	CP021764_02459	99.64% ISRso11_aa2	99.64% CP021764_03154	IS3 ssgr IS150	2614254	2613418	837
CRMRs218	CP021764_02460	100% ISRso11_aa1	100% CP021764_03155	IS3 ssgr IS150	2614784	2614251	534
CRMRs218	CP021764_03154	100% ISRso11_aa2	100% CP021764_02311	IS3 ssgr IS150	3327542	3326706	837
CRMRs218	CP021764_03155	100% ISRso11_aa1	100% CP021764_02460	IS3 ssgr IS150	3328072	3327539	534
CRMRs218	CP021764_01472	98.36% ISRso10_aa1	100% CP021764_01880	IS3 ssgr IS2	1538781	1539173	393
CRMRs218	CP021764_01473	99.64% ISRso10_aa2	99.64% CP021764_01879	IS3 ssgr IS2	1539170	1540018	849
CRMRs218	CP021764_01879	99.29% ISRso10_aa2	99.64% CP021764_01473	IS3 ssgr IS2	2002403	2001555	849

CRMRs218	CP021764_01880	98.36% ISRso10_aa1	100% CP021764_01472	IS3 ssgr IS2	2002792	2002400	393
CRMRs218	CP021764_02379	98.58% ISRso10_aa2	98.93% CP021764_01473	IS3 ssgr IS2	2531444	2530596	849
CRMRs218	CP021764_02380	98.86% ISRso10_aa1	97.72% CP021764_01880	IS3 ssgr IS2	2531737	2531441	297
CRMRs218	CP021764_02360	89.23% IS222_aa2	57.69% CP021764_02313	IS3 ssgr IS3	2515013	2514342	672
CRMRs218	CP021764_02361	87.25% IS222_aa1	No hit	IS3 ssgr IS3	2515504	2515196	309
CRMRs218	CP021764_00429	96.10% ISRso16_aa2	85.18% CP021764_02755	IS3 ssgr IS407	451724	452023	300
CRMRs218	CP021764_00641	88.09% ISAtu5_aa1	80.23% CP021764_02457	IS3 ssgr IS407	671045	670785	261
CRMRs218	CP021764_00846	98.90% ISRso14_aa2	100% CP021764_02456	IS3 ssgr IS407	870532	869984	549
CRMRs218	CP021764_00847	100% ISRso14_aa1	100% CP021764_02457	IS3 ssgr IS407	871101	870838	264
CRMRs218	CP021764_01476	100% ISRso12_aa1	74.69% CP021764_00641	IS3 ssgr IS407	1541746	1542012	267
CRMRs218	CP021764_01477	100% ISRso12_aa2	100% CP021764_02755	IS3 ssgr IS407	1542162	1542866	705
CRMRs218	CP021764_01531	98.90% ISRso14_aa2	100% CP021764_02456	IS3 ssgr IS407	1597471	1596923	549
CRMRs218	CP021764_01532	100% ISRso14_aa1	100% CP021764_02457	IS3 ssgr IS407	1598040	1597777	264
CRMRs218	CP021764_02456	98.90% ISRso14_aa2	100% CP021764_01531	IS3 ssgr IS407	2612429	2611881	549
CRMRs218	CP021764_02457	100% ISRso14_aa1	100% CP021764_01532	IS3 ssgr IS407	2612998	2612735	264
CRMRs218	CP021764_02755	100% ISRso12_aa2	100% CP021764_01477	IS3 ssgr IS407	2911959	2911255	705
CRMRs218	CP021764_01589	76.34% ISBcen21_aa1	54.54% CP021764_02314	IS3 ssgr IS51	1661244	1661531	288
CRMRs218	CP021764_01522	100% ISRso13_aa1	100% CP021764_03306	IS4 ssgr IS4	1590586	1589345	1.242
CRMRs218	CP021764_01915	100% ISRso13_aa1	100% CP021764_03306	IS4 ssgr IS4	2047952	2049286	1.335
CRMRs218	CP021764_03237	100% ISRso13_aa1	100% CP021764_03306	IS4 ssgr IS4	3427869	3429203	1.335
CRMRs218	CP021764_03306	100% ISRso13_aa1	100% CP021764_03237	IS4 ssgr IS4	3500459	3499125	1.335
CRMRs218	CP021764_00210	95.62% ISRso1_aa1	99.27% CP021764_03417	IS5	231889	232788	900
CRMRs218	CP021764_01026	97.21% ISRso1_aa1	95.21% CP021764_03417	IS5	1060261	1059506	756
CRMRs218	CP021764_03264	96.35% ISRso1_aa1	96.35% CP021764_03417	IS5	3460936	3461760	825
CRMRs218	CP021764_03274	88.15% ISRso1_aa1	89.47% CP021764_03264	IS5	3472497	3473306	810
CRMRs218	CP021764_03284	94.87% ISRso1_aa1	97.84% CP021764_00210	IS5	3483646	3484488	843
CRMRs218	CP021764_03417	95.98% ISRso1_aa1	99.27% CP021764_00210	IS5	3613190	3612366	825
CRMRs218	CP021764_00911	76.56% ISCARN14_aa1	49.26% CP021764_00210	IS5 ssgr IS1031	940716	939538	1.179
CRMRs218	CP021764_00861	100% ISRso9_aa1	100% CP021764_03488	IS5 ssgr IS5	883345	884667	1.323
CRMRs218	CP021764_01754	99.68% ISRso18_aa1	No hit	IS5 ssgr IS5	1821225	1820260	966
CRMRs218	CP021764_03191	100% ISRso9_aa1	100% CP021764_03488	IS5 ssgr IS5	3367383	3366061	1.323
CRMRs218	CP021764_03214	100% ISRso9_aa1	100% CP021764_03488	IS5 ssgr IS5	3396972	3395650	1.323
CRMRs218	CP021764_03488	100% ISRso9_aa1	100% CP021764_03214	IS5 ssgr IS5	3697368	3696046	1.323
CRMRs218	CP021764_00103	100% ISRso5_aa1	100% CP021764_03461	IS630	118044	119135	1.092
CRMRs218	CP021764_00109	99.72% ISRso5_aa1	99.72% CP021764_03461	IS630	127969	12906	1.092

CRMRs218	CP021764_00655	100% ISRso5_aa1	100% CP021764_03461	IS630	683464	684555	1.092
CRMRs218	CP021764_02223	100% ISRso5_aa1	100% CP021764_03461	IS630	2360302	2361393	1.092
CRMRs218	CP021764_03461	100% ISRso5_aa1	100% CP021764_02223	IS630	3660831	3659740	1.092
CRMRs218	CP021764_00053	48.25% ISWz1_aa1	53.14% CP021764_02595	IS91	5836	57371	990
CRMRs218	CP021764_01595	40.16% ISShvi3_aa1	52.96% CP021764_01702	IS91	1670709	1669645	1.065
CRMRs218	CP021764_02595	48.98% ISMno23_aa1	52.10% CP021764_00053	IS91	2752251	2753177	927
CRMRs218	CP021764_02624	100% ISRso15_aa1	No hit	ISL3	2780421	2781641	1.221
CRMRs218	CP021764_02671	77.94% ISSm4_aa2	No hit	ISL3	2824871	2823804	1.068
CRMRs218	CP021764_03464	38.94% ISKpn25_aa1	No hit	ISL3	3666102	3664657	1.446
CRMRs218	CP021764_01894	64.16% ISKpn21_aa1	92.5% CP021764_01895	ISNCY ssgr IS1202	2023666	2023274	393
CRMRs218	CP021764_01895	71.39% ISKpn21_aa1	92.5% CP021764_01894	ISNCY ssgr IS1202	2025295	2023820	1.476
CRMRs218	CP021764_00605	52.74% ISMpo10_aa1	54.92% CP021764_00149	Tn3	636592	637098	507
CRMRs218	CP021764_00909	53.79% ISMpo10_aa3	71.09% CP021764_03311	Tn3	937596	938975	1.38
CRMRs218	CP021764_03240	61.81% ISMpo10_aa3	96.96% CP021764_03311	Tn3	3430707	3432095	1.389
CRMRs218	CP021764_03311	55.34% ISMpo10_aa3	96.96% CP021764_03240	Tn3	3503119	3504507	1.389

YC40M	IS_96ce018c_00602	95.76% ISBma3_aa1	100% IS_96ce018c_02930	IS110	62152	620315	1.206
YC40M	IS_96ce018c_00689	95.76% ISBma3_aa1	100% IS_96ce018c_02930	IS110	717324	716119	1.206
YC40M	IS_96ce018c_01658	95.76% ISBma3_aa1	100% IS_96ce018c_02930	IS110	1748602	1749807	1.206
YC40M	IS_96ce018c_02626	95.76% ISBma3_aa1	100% IS_96ce018c_02930	IS110	2848322	2847117	1.206
YC40M	IS_96ce018c_02930	95.76% ISBma3_aa1	100% IS_96ce018c_02626	IS110	3158590	3157385	1.206
YC40M	IS_96ce018c_00416	82.67% IS1383_aa1	100% IS_96ce018c_03480	IS110 ssgr IS1111	435207	436208	1.002
YC40M	IS_96ce018c_02694	82.67% IS1383_aa1	100% IS_96ce018c_03480	IS110 ssgr IS1111	2921145	2920144	1.002
YC40M	IS_96ce018c_02726	82.67% IS1383_aa1	100% IS_96ce018c_03480	IS110 ssgr IS1111	2957862	2956861	1.002
YC40M	IS_96ce018c_03398	82.67% IS1383_aa1	100% IS_96ce018c_03480	IS110 ssgr IS1111	3636094	3637095	1.002
YC40M	IS_96ce018c_03441	82.67% IS1383_aa1	100% IS_96ce018c_03480	IS110 ssgr IS1111	3680405	3679404	1.002
YC40M	IS_96ce018c_03460	88.75% ISBcen4_aa1	72.72% IS_96ce018c_03480	IS110 ssgr IS1111	3698428	3697409	1.02
YC40M	IS_96ce018c_03480	82.67% IS1383_aa1	100% IS_96ce018c_03441	IS110 ssgr IS1111	3720776	3721777	1.002
YC40M	IS_96ce018c_02666	83.75% ISBusp4_aa1	No hit	IS1182	2880151	2878706	1.446
YC40M	IS_96ce018c_02673	49.48% ISHvo5_aa1	43.39% IS_96ce018c_02602	IS1595 ssgr ISH4	2888850	2888062	789
YC40M	IS_96ce018c_00315	100% ISRso11_aa1	100% IS_96ce018c_02855	IS3 ssgr IS150	348778	349311	534
YC40M	IS_96ce018c_00316	99.64% ISRso11_aa2	100% IS_96ce018c_02856	IS3 ssgr IS150	349308	350144	837
YC40M	IS_96ce018c_01985	99.64% ISRso11_aa2	100% IS_96ce018c_02856	IS3 ssgr IS150	2150718	2149882	837
YC40M	IS_96ce018c_01986	100% ISRso11_aa1	100% IS_96ce018c_02855	IS3 ssgr IS150	2151248	2150715	534
YC40M	IS_96ce018c_02855	100% ISRso11_aa1	100% IS_96ce018c_01986	IS3 ssgr IS150	3083925	3084458	534

YC40M	IS_96ce018c_02856	99.64% ISRso11_aa2	100% IS_96ce018c_01985	IS3 ssgr IS150	3084455	3085291	837
YC40M	IS_96ce018c_01194	87.25% IS222_aa1	59.77% IS_96ce018c_01150	IS3 ssgr IS3	1249279	1249587	309
YC40M	IS_96ce018c_01195	89.68% IS222_aa2	56.22% IS_96ce018c_01149	IS3 ssgr IS3	1249770	1250441	672
YC40M	IS_96ce018c_01149	89.34% ISAisp2_aa2	94.94% IS_96ce018c_01182	IS3 ssgr IS51	1202688	1201813	876
YC40M	IS_96ce018c_01150	81.52% ISAisp2_aa1	96.73% IS_96ce018c_01183	IS3 ssgr IS51	1202963	1202685	279
YC40M	IS_96ce018c_01182	86.38% ISAisp2_aa2	94.94% IS_96ce018c_01149	IS3 ssgr IS51	1238238	1237447	792
YC40M	IS_96ce018c_01183	80.43% ISAisp2_aa1	96.73% IS_96ce018c_01150	IS3 ssgr IS51	1238513	1238235	279
YC40M	IS_96ce018c_02678	65.95% IS1382_aa1	100% IS_96ce018c_03325	IS30	2897794	2896775	1.02
YC40M	IS_96ce018c_02740	65.95% IS1382_aa1	100% IS_96ce018c_03325	IS30	2972463	2973482	1.02
YC40M	IS_96ce018c_03325	65.95% IS1382_aa1	100% IS_96ce018c_02740	IS30	3559939	3558920	1.02
YC40M	IS_96ce018c_00146	61.68% ISCro3_aa1	100% IS_96ce018c_02574	IS4	164937	163609	1.329
YC40M	IS_96ce018c_00282	61.68% ISCro3_aa1	100% IS_96ce018c_02574	IS4	306904	308232	1.329
YC40M	IS_96ce018c_00525	61.44% ISCro3_aa1	99.77% IS_96ce018c_03284	IS4	543818	545146	1.329
YC40M	IS_96ce018c_00564	61.68% ISCro3_aa1	100% IS_96ce018c_02574	IS4	584806	583478	1.329
YC40M	IS_96ce018c_00837	61.68% ISCro3_aa1	100% IS_96ce018c_02574	IS4	879891	881219	1.329
YC40M	IS_96ce018c_01279	61.89% ISCro3_aa1	96.32% IS_96ce018c_03284	IS4	1348219	1346894	1.326
YC40M	IS_96ce018c_01791	61.68% ISCro3_aa1	100% IS_96ce018c_02574	IS4	1885428	1886756	1.329
YC40M	IS_96ce018c_02431	61.68% ISCro3_aa1	100% IS_96ce018c_03284	IS4	2634391	2633063	1.329
YC40M	IS_96ce018c_02476	61.68% ISCro3_aa1	100% IS_96ce018c_02574	IS4	2678894	2677566	1.329
YC40M	IS_96ce018c_02478	61.68% ISCro3_aa1	100% IS_96ce018c_03284	IS4	2680111	2681439	1.329
YC40M	IS_96ce018c_02496	61.68% ISCro3_aa1	100% IS_96ce018c_02574	IS4	2702357	2701029	1.329
YC40M	IS_96ce018c_02504	61.68% ISCro3_aa1	100% IS_96ce018c_02574	IS4	2709563	2710891	1.329
YC40M	IS_96ce018c_02539	61.68% ISCro3_aa1	100% IS_96ce018c_02574	IS4	2745987	2747315	1.329
YC40M	IS_96ce018c_02574	61.68% ISCro3_aa1	100% IS_96ce018c_02539	IS4	2779755	2778427	1.329
YC40M	IS_96ce018c_02823	61.68% ISCro3_aa1	100% IS_96ce018c_03284	IS4	3054202	3055530	1.329
YC40M	IS_96ce018c_03284	61.68% ISCro3_aa1	100% IS_96ce018c_02823	IS4	3515639	3516967	1.329
YC40M	IS_96ce018c_00270	76.86% ISAzo5_aa1	100% IS_96ce018c_02348	IS4 ssgr IS50	289512	288205	1.308
YC40M	IS_96ce018c_01130	76.86% ISAzo5_aa1	100% IS_96ce018c_02348	IS4 ssgr IS50	1181197	1179890	1.308
YC40M	IS_96ce018c_01660	76.86% ISAzo5_aa1	100% IS_96ce018c_02348	IS4 ssgr IS50	1751230	1752537	1.308
YC40M	IS_96ce018c_02348	76.86% ISAzo5_aa1	100% IS_96ce018c_01660	IS4 ssgr IS50	2552342	2551035	1.308
YC40M	IS_96ce018c_01643	97.81% ISRso1_aa1	98.17% IS_96ce018c_02691	IS5	1732903	1733727	825
YC40M	IS_96ce018c_02691	97.44% ISRso1_aa1	98.17% IS_96ce018c_01643	IS5	2917913	2918737	825
YC40M	IS_96ce018c_00020	100% IS1421_aa1	100% IS_96ce018c_03479	IS5 ssgr IS427	26943	26539	405
YC40M	IS_96ce018c_00415	100% IS1421_aa1	100% IS_96ce018c_03479	IS5 ssgr IS427	43427	434674	405
YC40M	IS_96ce018c_02695	100% IS1421_aa1	100% IS_96ce018c_03479	IS5 ssgr IS427	2922082	2921678	405

YC40M	IS_96ce018c_02727	100% IS1421_aa1	100% IS_96ce018c_03479	IS5 ssgr IS427	2958046	2958450	405
YC40M	IS_96ce018c_03397	100% IS1421_aa1	100% IS_96ce018c_03479	IS5 ssgr IS427	3635157	3635561	405
YC40M	IS_96ce018c_03442	100% IS1421_aa1	100% IS_96ce018c_03479	IS5 ssgr IS427	3681342	3680938	405
YC40M	IS_96ce018c_03479	100% IS1421_aa1	100% IS_96ce018c_03442	IS5 ssgr IS427	3719839	3720243	405
YC40M	IS_96ce018c_00027	94.51% IS1021_aa1	100% IS_96ce018c_03309	IS5 ssgr IS5	32771	33757	987
YC40M	IS_96ce018c_00375	99.37% IS1405_aa1	100% IS_96ce018c_03363	IS5 ssgr IS5	406501	405536	966
YC40M	IS_96ce018c_00595	99.06% IS1405_aa1	100% IS_96ce018c_03328	IS5 ssgr IS5	615076	616041	966
YC40M	IS_96ce018c_00610	94.51% IS1021_aa1	100% IS_96ce018c_03309	IS5 ssgr IS5	630077	631063	987
YC40M	IS_96ce018c_00853	94.51% IS1021_aa1	100% IS_96ce018c_03309	IS5 ssgr IS5	899141	898155	987
YC40M	IS_96ce018c_00871	94.51% IS1021_aa1	100% IS_96ce018c_03309	IS5 ssgr IS5	913958	914944	987
YC40M	IS_96ce018c_00925	99.06% IS1405_aa1	100% IS_96ce018c_03328	IS5 ssgr IS5	968226	969191	966
YC40M	IS_96ce018c_01013	94.51% IS1021_aa1	100% IS_96ce018c_03309	IS5 ssgr IS5	1056525	1057511	987
YC40M	IS_96ce018c_01378	99.06% IS1405_aa1	100% IS_96ce018c_03328	IS5 ssgr IS5	1450997	1450032	966
YC40M	IS_96ce018c_01588	99.06% IS1405_aa1	100% IS_96ce018c_03328	IS5 ssgr IS5	1671002	1670037	966
YC40M	IS_96ce018c_01655	91.66% IS1021_aa1	100% IS_96ce018c_03309	IS5 ssgr IS5	1746954	1746664	291
YC40M	IS_96ce018c_01656	97.59% IS1021_aa1	100% IS_96ce018c_03309	IS5 ssgr IS5	1747267	1747007	261
YC40M	IS_96ce018c_01669	94.51% IS1021_aa1	100% IS_96ce018c_03309	IS5 ssgr IS5	1759512	1758526	987
YC40M	IS_96ce018c_02447	99.06% IS1405_aa1	100% IS_96ce018c_03328	IS5 ssgr IS5	2647896	2646931	966
YC40M	IS_96ce018c_02624	94.51% IS1021_aa1	100% IS_96ce018c_03309	IS5 ssgr IS5	2846261	2845275	987
YC40M	IS_96ce018c_02649	99.06% IS1405_aa1	100% IS_96ce018c_03328	IS5 ssgr IS5	2864189	2863224	966
YC40M	IS_96ce018c_02689	99.06% IS1405_aa1	100% IS_96ce018c_03328	IS5 ssgr IS5	2916329	2915364	966
YC40M	IS_96ce018c_02735	94.51% IS1021_aa1	100% IS_96ce018c_03309	IS5 ssgr IS5	2965305	2966291	987
YC40M	IS_96ce018c_02736	94.51% IS1021_aa1	100% IS_96ce018c_03309	IS5 ssgr IS5	2967840	2966854	987
YC40M	IS_96ce018c_02741	99.06% IS1405_aa1	100% IS_96ce018c_03328	IS5 ssgr IS5	2974482	2975447	966
YC40M	IS_96ce018c_03098	99.37% IS1405_aa1	100% IS_96ce018c_03363	IS5 ssgr IS5	3334314	3333349	966
YC40M	IS_96ce018c_03309	94.51% IS1021_aa1	100% IS_96ce018c_02736	IS5 ssgr IS5	3541562	3542548	987
YC40M	IS_96ce018c_03328	99.06% IS1405_aa1	100% IS_96ce018c_02741	IS5 ssgr IS5	3567471	3568436	966
YC40M	IS_96ce018c_03363	99.37% IS1405_aa1	100% IS_96ce018c_03098	IS5 ssgr IS5	3604620	3603655	966
YC40M	IS_96ce018c_02089	85.13% ISAzo9_aa2	No hit	IS630	2267869	2266742	1.128
YC40M	IS_96ce018c_00023	56.95% ISRm2_aa1	100% IS_96ce018c_02707	IS66	29249	29731	483
YC40M	IS_96ce018c_00024	73.27% ISRm2_aa2	100% IS_96ce018c_02706	IS66	29728	30081	354
YC40M	IS_96ce018c_00025	61.53% ISPpu13_aa2	100% IS_96ce018c_02705	IS66	30114	3167	1.557
YC40M	IS_96ce018c_00621	56.95% ISRm2_aa1	100% IS_96ce018c_02707	IS66	643498	64398	483
YC40M	IS_96ce018c_00622	73.27% ISRm2_aa2	100% IS_96ce018c_02706	IS66	643977	64433	354
YC40M	IS_96ce018c_00623	61.53% ISPpu13_aa2	100% IS_96ce018c_02705	IS66	644363	645919	1.557

YC40M	IS_96ce018c_00884	61.53% ISPpu13_aa2	100% IS_96ce018c_02705	IS66	925191	923635	1.557
YC40M	IS_96ce018c_00885	73.27% ISRm2_aa2	100% IS_96ce018c_02706	IS66	925577	925224	354
YC40M	IS_96ce018c_00886	56.95% ISRm2_aa1	100% IS_96ce018c_02707	IS66	926056	925574	483
YC40M	IS_96ce018c_01649	61.53% ISPpu13_aa2	100% IS_96ce018c_02705	IS66	1741651	1740095	1.557
YC40M	IS_96ce018c_01650	73.27% ISRm2_aa2	100% IS_96ce018c_02706	IS66	1742037	1741684	354
YC40M	IS_96ce018c_01651	56.95% ISRm2_aa1	100% IS_96ce018c_02707	IS66	1742516	1742034	483
YC40M	IS_96ce018c_02342	61.53% ISPpu13_aa2	100% IS_96ce018c_02705	IS66	2548348	2546792	1.557
YC40M	IS_96ce018c_02343	73.27% ISRm2_aa2	100% IS_96ce018c_02706	IS66	2548734	2548381	354
YC40M	IS_96ce018c_02344	56.95% ISRm2_aa1	100% IS_96ce018c_02707	IS66	2549213	2548731	483
YC40M	IS_96ce018c_02705	61.53% ISPpu13_aa2	100% IS_96ce018c_02342	IS66	2936456	2934900	1.557
YC40M	IS_96ce018c_02706	73.27% ISRm2_aa2	100% IS_96ce018c_02343	IS66	2936842	2936489	354
YC40M	IS_96ce018c_02707	56.95% ISRm2_aa1	100% IS_96ce018c_02344	IS66	2937321	2936839	483
YC40M	IS_96ce018c_00986	47.5% ISTha3_aa2	51.50% IS_96ce018c_03533	IS91	1028484	1027558	927
YC40M	IS_96ce018c_01671	45.41% ISTha3_aa2	53.80% IS_96ce018c_01653	IS91	1762786	1761731	1.056
YC40M	IS_96ce018c_03533	43.33% ISMno24_aa2	52.12% IS_96ce018c_00986	IS91	3785484	3786473	990
YC40M	IS_96ce018c_00049	38.97% ISKpn25_aa1	No hit	ISL3	56505	5795	1.446
YC40M	IS_96ce018c_00010	46.61% ISPa4_aa1	No hit	ISNCY	11679	9589	2.091
YC40M	IS_96ce018c_01913	62.5% ISKpn21_aa1	93.33% IS_96ce018c_01914	ISNCY ssgr IS1202	2077872	2077471	402
YC40M	IS_96ce018c_01914	72.27% ISKpn21_aa1	93.33% IS_96ce018c_01913	ISNCY ssgr IS1202	2079501	2078026	1.476
YC40M	IS_96ce018c_00202	61.81% ISMpo10_aa3	73.55% IS_96ce018c_02600	Tn3	219017	217629	1.389
YC40M	IS_96ce018c_01663	75.93% ISSba14_aa1	47.23% IS_96ce018c_02600	Tn3	1754761	1755339	579
YC40M	IS_96ce018c_02600	54.43% ISMpo10_aa3	96.73% IS_96ce018c_02671	Tn3	2810471	2809089	1.383
YC40M	IS_96ce018c_02671	53.04% ISMpo10_aa3	96.73% IS_96ce018c_02600	Tn3	2886554	2885172	1.383

SN82F48	IS_98f194dd_01524	75.64% IS5708_aa1	No hit	IS110 ssgr IS1111	1608510	1608887	378
SN82F48	IS_98f194dd_00787	75.93% ISAav1_aa1	100% IS_98f194dd_00807	IS21	83214	833636	1.497
SN82F48	IS_98f194dd_00788	80.30% ISAav1_aa2	100% IS_98f194dd_00806	IS21	833626	834423	798
SN82F48	IS_98f194dd_00806	80.30% ISAav1_aa2	100% IS_98f194dd_00788	IS21	85449	853693	798
SN82F48	IS_98f194dd_00807	75.93% ISAav1_aa1	100% IS_98f194dd_00787	IS21	855976	85448	1.497
SN82F48	IS_98f194dd_01578	92.85% ISCARN17_aa2	49.02% IS_98f194dd_00806	IS21	1656416	1655652	765
SN82F48	IS_98f194dd_01579	81.85% ISCARN17_aa1	40.84% IS_98f194dd_00807	IS21	1658050	1656413	1.638
SN82F48	IS_98f194dd_00208	98.05% ISMca5_aa1	100% IS_98f194dd_03049	IS256	213079	211814	1.266
SN82F48	IS_98f194dd_00443	98.05% ISMca5_aa1	100% IS_98f194dd_03049	IS256	455171	453906	1.266
SN82F48	IS_98f194dd_00978	98.05% ISMca5_aa1	100% IS_98f194dd_03049	IS256	1019553	1020818	1.266
SN82F48	IS_98f194dd_00987	98.05% ISMca5_aa1	100% IS_98f194dd_03049	IS256	1027684	1028949	1.266

SN82F48	IS_98f194dd_01636	98.05% ISMca5_aa1	100% IS_98f194dd_03049	IS256	1716062	1714797	1.266
SN82F48	IS_98f194dd_02603	98.05% ISMca5_aa1	100% IS_98f194dd_03049	IS256	2746809	2748074	1.266
SN82F48	IS_98f194dd_02614	98.05% ISMca5_aa1	100% IS_98f194dd_03049	IS256	2761922	2760657	1.266
SN82F48	IS_98f194dd_02743	98.05% ISMca5_aa1	100% IS_98f194dd_03049	IS256	2894412	2895677	1.266
SN82F48	IS_98f194dd_03049	98.05% ISMca5_aa1	100% IS_98f194dd_02743	IS256	3231407	3230142	1.266
SN82F48	IS_98f194dd_02055	85.65% ISBcen7_aa2	98.05% IS_98f194dd_02076	IS3 ssgr IS3	2157163	2156441	723
SN82F48	IS_98f194dd_02076	87.41% ISBcen7_aa2	98.05% IS_98f194dd_02055	IS3 ssgr IS3	2176264	2175800	465
SN82F48	IS_98f194dd_00372	93.33% IS401_aa2	52.59% IS_98f194dd_02055	IS3 ssgr IS51	389565	389023	543
SN82F48	IS_98f194dd_02739	80.74% ISBxe3_aa1	No hit	IS481	2891863	2890967	897
SN82F48	IS_98f194dd_00375	91.92% ISPa54_aa1	76.66% IS_98f194dd_01600	IS5 ssgr IS5	393697	392717	981
SN82F48	IS_98f194dd_01600	78.33% ISPsp3_aa1	76.66% IS_98f194dd_00375	IS5 ssgr IS5	1677127	1676933	195
SN82F48	IS_98f194dd_00720	80.89% ISBcen19_aa3	100% IS_98f194dd_03303	IS66	758893	757364	1.53
SN82F48	IS_98f194dd_00721	77.08% ISSal1_aa2	100% IS_98f194dd_03304	IS66	759246	758956	291
SN82F48	IS_98f194dd_00722	65.38% ISBcen19_aa1	100% IS_98f194dd_03305	IS66	759659	759288	372
SN82F48	IS_98f194dd_01200	65.38% ISBcen19_aa1	100% IS_98f194dd_03305	IS66	1259055	1259426	372
SN82F48	IS_98f194dd_01201	77.08% ISSal1_aa2	100% IS_98f194dd_03304	IS66	1259468	1259758	291
SN82F48	IS_98f194dd_01202	80.89% ISBcen19_aa3	100% IS_98f194dd_03303	IS66	1259821	1261350	1.53
SN82F48	IS_98f194dd_01356	65.38% ISBcen19_aa1	100% IS_98f194dd_03305	IS66	1431037	1431408	372
SN82F48	IS_98f194dd_01357	77.08% ISSal1_aa2	100% IS_98f194dd_03304	IS66	1431450	1431740	291
SN82F48	IS_98f194dd_01358	80.89% ISBcen19_aa3	100% IS_98f194dd_03303	IS66	1431803	1433332	1.53
SN82F48	IS_98f194dd_01525	71.90% ISPpu19_aa3	70% IS_98f194dd_03303	IS66	1609372	1609001	372
SN82F48	IS_98f194dd_02544	80.89% ISBcen19_aa3	100% IS_98f194dd_03303	IS66	2679042	2677513	1.53
SN82F48	IS_98f194dd_02545	77.08% ISSal1_aa2	100% IS_98f194dd_03304	IS66	2679395	2679105	291
SN82F48	IS_98f194dd_02546	65.38% ISBcen19_aa1	100% IS_98f194dd_03305	IS66	2679808	2679437	372
SN82F48	IS_98f194dd_02605	52.34% ISRsp1_aa4	No hit	IS66	2751545	2751955	411
SN82F48	IS_98f194dd_03303	80.89% ISBcen19_aa3	100% IS_98f194dd_02544	IS66	3497723	3496194	1.53
SN82F48	IS_98f194dd_03304	77.08% ISSal1_aa2	100% IS_98f194dd_02545	IS66	3498076	3497786	291
SN82F48	IS_98f194dd_03305	65.38% ISBcen19_aa1	100% IS_98f194dd_02546	IS66	3498489	3498118	372
SN82F48	IS_98f194dd_00651	48.75% ISWz1_aa1	52.72% IS_98f194dd_03201	IS91	68138	682366	987
SN82F48	IS_98f194dd_01361	43.75% ISShvi3_aa1	60.05% IS_98f194dd_02551	IS91	1438066	1437020	1.047
SN82F48	IS_98f194dd_02532	46.70% ISTha3_aa2	51.24% IS_98f194dd_01361	IS91	2666452	2667537	1.086
SN82F48	IS_98f194dd_03201	46.07% ISTha3_aa2	53.17% IS_98f194dd_00651	IS91	3385615	3386550	936
SN82F48	IS_98f194dd_00381	56.09% ISKpn31_aa1	No hit	ISAs1	398379	39887	492
SN82F48	IS_98f194dd_00314	77.85% ISIde1_aa1	100% IS_98f194dd_00801	ISL3	32767	328965	1.296
SN82F48	IS_98f194dd_00363	77.85% ISIde1_aa1	100% IS_98f194dd_00801	ISL3	379541	380836	1.296

SN82F48	IS_98f194dd_00694	77.85% ISIde1_aa1	100% IS_98f194dd_00801	ISL3	724452	725747	1.296
SN82F48	IS_98f194dd_00723	57.68% ISKpn25_aa3	No hit	ISL3	762633	759712	2.922
SN82F48	IS_98f194dd_00725	47.98% ISKpn25_aa2	No hit	ISL3	764953	763616	1.338
SN82F48	IS_98f194dd_00726	56.82% ISKpn25_aa1	No hit	ISL3	766791	76495	1.842
SN82F48	IS_98f194dd_00782	77.85% ISIde1_aa1	100% IS_98f194dd_00801	ISL3	827163	825868	1.296
SN82F48	IS_98f194dd_00801	77.85% ISIde1_aa1	100% IS_98f194dd_00782	ISL3	847471	846176	1.296
SN82F48	IS_98f194dd_00825	77.85% ISIde1_aa1	100% IS_98f194dd_02924	ISL3	871259	869964	1.296
SN82F48	IS_98f194dd_01169	78.08% ISIde1_aa1	100% IS_98f194dd_03160	ISL3	1221277	1222572	1.296
SN82F48	IS_98f194dd_01173	78.08% ISIde1_aa1	100% IS_98f194dd_03160	ISL3	1229047	1227752	1.296
SN82F48	IS_98f194dd_01345	78.08% ISIde1_aa1	100% IS_98f194dd_03160	ISL3	1421693	1420398	1.296
SN82F48	IS_98f194dd_01496	77.85% ISIde1_aa1	100% IS_98f194dd_02924	ISL3	1580298	1579003	1.296
SN82F48	IS_98f194dd_01500	77.85% ISIde1_aa1	100% IS_98f194dd_02924	ISL3	1583610	1582315	1.296
SN82F48	IS_98f194dd_01540	78.08% ISIde1_aa1	100% IS_98f194dd_03160	ISL3	1622550	1623845	1.296
SN82F48	IS_98f194dd_01580	77.85% ISIde1_aa1	100% IS_98f194dd_02924	ISL3	1659520	1658225	1.296
SN82F48	IS_98f194dd_01588	78.08% ISIde1_aa1	100% IS_98f194dd_03160	ISL3	1666750	1665455	1.296
SN82F48	IS_98f194dd_02437	50.47% ISSm4_aa2	No hit	ISL3	2571428	2572438	1.011
SN82F48	IS_98f194dd_02924	77.85% ISIde1_aa1	100% IS_98f194dd_01580	ISL3	3088598	3089893	1.296
SN82F48	IS_98f194dd_03011	78.08% ISIde1_aa1	100% IS_98f194dd_03160	ISL3	3185794	3187089	1.296
SN82F48	IS_98f194dd_03160	78.08% ISIde1_aa1	100% IS_98f194dd_01588	ISL3	3346746	3348041	1.296
SN82F48	IS_98f194dd_00192	65.81% IS882_aa1	No hit	Tn3	19075	193683	2.934
SN82F48	IS_98f194dd_00193	75.55% ISPa43_aa1	No hit	Tn3	194769	19381	960
SN82F48	IS_98f194dd_00233	78.08% TnShfr1_aa1	45.13% IS_98f194dd_00256	Tn3	238657	239631	975
SN82F48	IS_98f194dd_00406	62.72% ISMpo10_aa3	No hit	Tn3	419254	420633	1.38
SN82F48	IS_98f194dd_01413	53.84% ISMpo10_aa1	45.98% IS_98f194dd_00281	Tn3	1492524	1493042	519
SN83A39	IS_dabb08e8_00018	99.19% ISBmu3_aa2	100% IS_dabb08e8_02008	IS21	15063	14314	750
SN83A39	IS_dabb08e8_00019	98.25% ISBmu3_aa1	No hit	IS21	16629	15079	1.551
SN83A39	IS_dabb08e8_02008	99.15% ISBmu3_aa2	100% IS_dabb08e8_00018	IS21	2115896	2116252	357
SN83A39	IS_dabb08e8_02339	78.90% ISSpwi2_aa1	100% IS_dabb08e8_03248	IS256	2451021	2452289	1.269
SN83A39	IS_dabb08e8_03236	79.16% ISBcen18_aa1	99.54% IS_dabb08e8_02339	IS256	3366829	3366173	657
SN83A39	IS_dabb08e8_03248	81.14% ISSpwi2_aa1	100% IS_dabb08e8_02339	IS256	3379264	3378164	1.101
SN83A39	IS_dabb08e8_00010	79.23% ISPsp3_aa1	100% IS_dabb08e8_02016	IS5 ssgr IS5	7374	8363	990
SN83A39	IS_dabb08e8_01591	93.02% ISUnCu2_aa1	No hit	IS5 ssgr IS5	1680705	1680145	561
SN83A39	IS_dabb08e8_01599	94.44% ISUnCu2_aa1	No hit	IS5 ssgr IS5	1688446	1687958	489
SN83A39	IS_dabb08e8_02016	79.23% ISPsp3_aa1	100% IS_dabb08e8_00010	IS5 ssgr IS5	2123192	2122203	990

SN83A39	IS_dabb08e8_00023	59.27% ISCARN56_aa2	No hit	IS607	18661	19206	546
SN83A39	IS_dabb08e8_00003	43.25% ISShvi3_aa1	43.70% IS_dabb08e8_01834	IS91	2472	1153	1.32
SN83A39	IS_dabb08e8_00967	46.07% ISTha3_aa2	52.61% IS_dabb08e8_01834	IS91	1017468	1018403	936
SN83A39	IS_dabb08e8_01834	48.75% ISWz1_aa1	52.16% IS_dabb08e8_00967	IS91	1938484	1939470	987
SN83A39	IS_dabb08e8_00226	50.47% ISSm4_aa2	No hit	ISL3	241162	242223	1.062
SN83A39	IS_dabb08e8_01541	53.77% ISSm4_aa1	No hit	ISL3	1634702	1637074	2.373
SN83A39	IS_dabb08e8_01542	44.71% ISKpn25_aa2	No hit	ISL3	1637074	1638471	1.398
SN83A39	IS_dabb08e8_01543	49.15% ISSm4_aa4	No hit	ISL3	1638458	1641457	3
SN83A39	IS_dabb08e8_00112	69.29% ISKpn21_aa1	No hit	ISNCY ssgr IS1202	11009	108597	1.494
SN83A39	IS_dabb08e8_00336	53.50% ISThsp9_aa1	74% IS_dabb08e8_01588	Tn3	354833	353454	1.38
SN83A39	IS_dabb08e8_01588	50% ISThsp9_aa1	74% IS_dabb08e8_00336	Tn3	1679049	1677682	1.368
SN83A39	IS_dabb08e8_02379	53.84% ISMpo10_aa1	47.44% IS_dabb08e8_01465	Tn3	2495655	2496173	519
SN83A39	IS_dabb08e8_02606	50% ISThsp9_aa1	73.70% IS_dabb08e8_01588	Tn3	2726688	2728028	1.341
SEPPX05	IS_67e77903_00988	67.55% ISBj4_aa1	100% IS_67e77903_02516	IS110 ssgr IS1111	1021322	1020279	1.044
SEPPX05	IS_67e77903_02076	67.85% ISBj4_aa1	100% IS_67e77903_02516	IS110 ssgr IS1111	2134839	2135615	777
SEPPX05	IS_67e77903_02516	67.55% ISBj4_aa1	100% IS_67e77903_00988	IS110 ssgr IS1111	2578331	2579374	1.044
SEPPX05	IS_67e77903_01784	53.80% ISHpa1_aa1	100% IS_67e77903_03660	IS1595 ssgr IS1016	1846091	1846870	780
SEPPX05	IS_67e77903_03660	53.80% ISHpa1_aa1	100% IS_67e77903_01784	IS1595 ssgr IS1016	3837565	3836786	780
SEPPX05	IS_67e77903_00855	50.51% ISHvo5_aa1	No hit	IS1595 ssgr ISH4	883971	883183	789
SEPPX05	IS_67e77903_00079	99.23% ISRso19_aa2	100% IS_67e77903_03731	IS21	8068	79892	789
SEPPX05	IS_67e77903_00080	97.58% ISRso19_aa1	100% IS_67e77903_03732	IS21	81672	80677	996
SEPPX05	IS_67e77903_00312	97.58% ISRso19_aa1	100% IS_67e77903_03732	IS21	335865	33686	996
SEPPX05	IS_67e77903_00313	99.23% ISRso19_aa2	100% IS_67e77903_03731	IS21	336857	337645	789
SEPPX05	IS_67e77903_00328	94.44% ISRme9_aa2	100% IS_67e77903_03365	IS21	347998	34724	759
SEPPX05	IS_67e77903_00329	93.42% ISRme9_aa1	100% IS_67e77903_03364	IS21	34956	348007	1.554
SEPPX05	IS_67e77903_00342	97.58% ISRso19_aa1	100% IS_67e77903_03732	IS21	358792	359787	996
SEPPX05	IS_67e77903_00343	99.23% ISRso19_aa2	100% IS_67e77903_03731	IS21	359784	360572	789
SEPPX05	IS_67e77903_00473	99.23% ISRso19_aa2	100% IS_67e77903_03731	IS21	484979	484191	789
SEPPX05	IS_67e77903_00474	97.58% ISRso19_aa1	100% IS_67e77903_03732	IS21	485971	484976	996
SEPPX05	IS_67e77903_00571	97.58% ISRso19_aa1	100% IS_67e77903_03732	IS21	592408	593403	996
SEPPX05	IS_67e77903_00572	99.23% ISRso19_aa2	100% IS_67e77903_03731	IS21	5934	594188	789
SEPPX05	IS_67e77903_00591	97.58% ISRso19_aa1	100% IS_67e77903_03732	IS21	6083	609295	996
SEPPX05	IS_67e77903_00592	99.23% ISRso19_aa2	100% IS_67e77903_03731	IS21	609292	61008	789
SEPPX05	IS_67e77903_00847	99.23% ISRso19_aa2	100% IS_67e77903_03731	IS21	875621	874833	789

SEPPX05	IS_67e77903_00848	97.58% ISRso19_aa1	100% IS_67e77903_03732	IS21	876613	875618	996
SEPPX05	IS_67e77903_01093	100% ISRso19_aa1	98.68% IS_67e77903_03732	IS21	1127361	1127828	468
SEPPX05	IS_67e77903_01094	93.42% ISRme9_aa1	100% IS_67e77903_03364	IS21	1128072	1129625	1.554
SEPPX05	IS_67e77903_01095	93.70% ISRme9_aa2	100% IS_67e77903_03365	IS21	1129634	1130065	432
SEPPX05	IS_67e77903_01098	95.45% ISRme9_aa2	100% IS_67e77903_03365	IS21	1131274	1131606	333
SEPPX05	IS_67e77903_01099	96.04% ISRso19_aa1	100% IS_67e77903_01886	IS21	1131729	1132262	534
SEPPX05	IS_67e77903_01100	99.23% ISRso19_aa2	100% IS_67e77903_03731	IS21	1132259	1133047	789
SEPPX05	IS_67e77903_01169	97.58% ISRso19_aa1	100% IS_67e77903_03732	IS21	1195276	1196271	996
SEPPX05	IS_67e77903_01170	99.23% ISRso19_aa2	100% IS_67e77903_03731	IS21	1196268	1197056	789
SEPPX05	IS_67e77903_01173	94.44% ISRme9_aa2	100% IS_67e77903_03365	IS21	1200710	1199952	759
SEPPX05	IS_67e77903_01174	93.42% ISRme9_aa1	100% IS_67e77903_03364	IS21	1202272	1200719	1.554
SEPPX05	IS_67e77903_01189	99.20% ISRso19_aa2	100% IS_67e77903_03731	IS21	1219390	1218632	759
SEPPX05	IS_67e77903_01190	97.58% ISRso19_aa1	100% IS_67e77903_03732	IS21	1220382	1219387	996
SEPPX05	IS_67e77903_01296	97.58% ISRso19_aa1	100% IS_67e77903_03732	IS21	1332339	1333334	996
SEPPX05	IS_67e77903_01297	99.23% ISRso19_aa2	100% IS_67e77903_03731	IS21	1333331	1334119	789
SEPPX05	IS_67e77903_01388	99.29% ISRso19_aa2	100% IS_67e77903_03731	IS21	1440527	1440069	459
SEPPX05	IS_67e77903_01391	93.42% ISRme9_aa1	100% IS_67e77903_03364	IS21	1441887	1443440	1.554
SEPPX05	IS_67e77903_01392	94.44% ISRme9_aa2	100% IS_67e77903_03365	IS21	1443449	1444207	759
SEPPX05	IS_67e77903_01401	97.58% ISRso19_aa1	100% IS_67e77903_03732	IS21	1451108	1452103	996
SEPPX05	IS_67e77903_01402	99.23% ISRso19_aa2	100% IS_67e77903_03731	IS21	1452100	1452888	789
SEPPX05	IS_67e77903_01488	97.58% ISRso19_aa1	100% IS_67e77903_03732	IS21	1552624	1553619	996
SEPPX05	IS_67e77903_01489	99.23% ISRso19_aa2	100% IS_67e77903_03731	IS21	1553616	1554404	789
SEPPX05	IS_67e77903_01787	99.23% ISRso19_aa2	100% IS_67e77903_03731	IS21	1850430	1849642	789
SEPPX05	IS_67e77903_01788	97.58% ISRso19_aa1	100% IS_67e77903_03732	IS21	1851422	1850427	996
SEPPX05	IS_67e77903_01864	94.44% ISRme9_aa2	100% IS_67e77903_03365	IS21	1930716	1929958	759
SEPPX05	IS_67e77903_01865	93.42% ISRme9_aa1	100% IS_67e77903_03364	IS21	1932278	1930725	1.554
SEPPX05	IS_67e77903_01883	100% ISRso19_aa1	100% IS_67e77903_03732	IS21	1947606	1947824	219
SEPPX05	IS_67e77903_01886	96.92% ISRso19_aa1	100% IS_67e77903_03732	IS21	1949054	1949836	783
SEPPX05	IS_67e77903_01887	99.23% ISRso19_aa2	100% IS_67e77903_03731	IS21	1949833	1950621	789
SEPPX05	IS_67e77903_01992	97.58% ISRso19_aa1	100% IS_67e77903_03732	IS21	2053635	2054630	996
SEPPX05	IS_67e77903_01993	99.23% ISRso19_aa2	100% IS_67e77903_03731	IS21	2054627	2055415	789
SEPPX05	IS_67e77903_02077	99.23% ISRso19_aa2	100% IS_67e77903_03731	IS21	2136462	2135674	789
SEPPX05	IS_67e77903_02078	97.58% ISRso19_aa1	100% IS_67e77903_03732	IS21	2137454	2136459	996
SEPPX05	IS_67e77903_02093	99.23% ISRso19_aa2	100% IS_67e77903_03731	IS21	2148593	2147805	789
SEPPX05	IS_67e77903_02094	97.58% ISRso19_aa1	100% IS_67e77903_03732	IS21	2149585	2148590	996

SEPPX05	IS_67e77903_02157	99.23% ISRso19_aa2	100% IS_67e77903_03731	IS21	2215421	2214633	789
SEPPX05	IS_67e77903_02158	97.58% ISRso19_aa1	100% IS_67e77903_03732	IS21	2216413	2215418	996
SEPPX05	IS_67e77903_02285	93.42% ISRme9_aa1	100% IS_67e77903_03364	IS21	2340168	2341721	1.554
SEPPX05	IS_67e77903_02286	94.44% ISRme9_aa2	100% IS_67e77903_03365	IS21	2341730	2342488	759
SEPPX05	IS_67e77903_02358	97.58% ISRso19_aa1	100% IS_67e77903_03732	IS21	2417348	2418343	996
SEPPX05	IS_67e77903_02359	99.23% ISRso19_aa2	100% IS_67e77903_03731	IS21	2418340	2419128	789
SEPPX05	IS_67e77903_02421	97.58% ISRso19_aa1	100% IS_67e77903_03732	IS21	2487793	2488788	996
SEPPX05	IS_67e77903_02422	99.23% ISRso19_aa2	100% IS_67e77903_03731	IS21	2488785	2489573	789
SEPPX05	IS_67e77903_02493	97.58% ISRso19_aa1	100% IS_67e77903_03732	IS21	2560267	2561262	996
SEPPX05	IS_67e77903_02494	99.23% ISRso19_aa2	100% IS_67e77903_03731	IS21	2561259	2562047	789
SEPPX05	IS_67e77903_02603	97.58% ISRso19_aa1	100% IS_67e77903_03732	IS21	2655318	2656313	996
SEPPX05	IS_67e77903_02604	99.23% ISRso19_aa2	100% IS_67e77903_03731	IS21	2656310	2657098	789
SEPPX05	IS_67e77903_02779	99.23% ISRso19_aa2	100% IS_67e77903_03731	IS21	2846326	2845538	789
SEPPX05	IS_67e77903_02780	97.58% ISRso19_aa1	100% IS_67e77903_03732	IS21	2847318	2846323	996
SEPPX05	IS_67e77903_02836	93.42% ISRme9_aa1	100% IS_67e77903_03364	IS21	2905255	2906808	1.554
SEPPX05	IS_67e77903_02837	94.44% ISRme9_aa2	100% IS_67e77903_03365	IS21	2906817	2907575	759
SEPPX05	IS_67e77903_02842	99.23% ISRso19_aa2	100% IS_67e77903_03731	IS21	2911275	2910487	789
SEPPX05	IS_67e77903_02843	97.58% ISRso19_aa1	100% IS_67e77903_03732	IS21	2912267	2911272	996
SEPPX05	IS_67e77903_02953	99.23% ISRso19_aa2	100% IS_67e77903_03731	IS21	3095083	3094295	789
SEPPX05	IS_67e77903_02954	97.58% ISRso19_aa1	100% IS_67e77903_03732	IS21	3096075	3095080	996
SEPPX05	IS_67e77903_02962	93.42% ISRme9_aa1	100% IS_67e77903_03364	IS21	3103741	3105294	1.554
SEPPX05	IS_67e77903_02963	94.44% ISRme9_aa2	100% IS_67e77903_03365	IS21	3105303	3106061	759
SEPPX05	IS_67e77903_02989	97.58% ISRso19_aa1	100% IS_67e77903_03732	IS21	3137844	3138839	996
SEPPX05	IS_67e77903_02990	99.23% ISRso19_aa2	100% IS_67e77903_03731	IS21	3138836	3139624	789
SEPPX05	IS_67e77903_03047	97.58% ISRso19_aa1	100% IS_67e77903_03732	IS21	3202827	3203822	996
SEPPX05	IS_67e77903_03048	99.23% ISRso19_aa2	100% IS_67e77903_03731	IS21	3203819	3204607	789
SEPPX05	IS_67e77903_03112	99.54% ISRso19_aa1	100% IS_67e77903_03732	IS21	3268450	3269151	702
SEPPX05	IS_67e77903_03113	94.44% ISRme9_aa2	100% IS_67e77903_03365	IS21	3269966	3269208	759
SEPPX05	IS_67e77903_03114	93.42% ISRme9_aa1	100% IS_67e77903_03364	IS21	3271528	3269975	1.554
SEPPX05	IS_67e77903_03115	93.80% ISRso19_aa1	100% IS_67e77903_01886	IS21	3271775	3272137	363
SEPPX05	IS_67e77903_03116	99.23% ISRso19_aa2	100% IS_67e77903_03731	IS21	3272134	3272922	789
SEPPX05	IS_67e77903_03184	97.58% ISRso19_aa1	100% IS_67e77903_03732	IS21	3338971	3339966	996
SEPPX05	IS_67e77903_03185	99.23% ISRso19_aa2	100% IS_67e77903_03731	IS21	3339963	3340751	789
SEPPX05	IS_67e77903_03257	99.23% ISRso19_aa2	100% IS_67e77903_03731	IS21	3409119	3408331	789
SEPPX05	IS_67e77903_03258	97.58% ISRso19_aa1	100% IS_67e77903_03732	IS21	3410111	3409116	996

SEPPX05	IS_67e77903_03364	93.42% ISRme9_aa1	100% IS_67e77903_03114	IS21	3514756	3516309	1.554
SEPPX05	IS_67e77903_03365	94.44% ISRme9_aa2	100% IS_67e77903_03113	IS21	3516318	3517076	759
SEPPX05	IS_67e77903_03368	97.58% ISRso19_aa1	100% IS_67e77903_03732	IS21	3518573	3519568	996
SEPPX05	IS_67e77903_03369	99.23% ISRso19_aa2	100% IS_67e77903_03731	IS21	3519565	3520353	789
SEPPX05	IS_67e77903_03500	97.58% ISRso19_aa1	100% IS_67e77903_03732	IS21	3670755	3671750	996
SEPPX05	IS_67e77903_03501	99.23% ISRso19_aa2	100% IS_67e77903_03731	IS21	3671747	3672535	789
SEPPX05	IS_67e77903_03731	99.23% ISRso19_aa2	100% IS_67e77903_03501	IS21	3910436	3909648	789
SEPPX05	IS_67e77903_03732	97.58% ISRso19_aa1	100% IS_67e77903_03500	IS21	3911428	3910433	996
SEPPX05	IS_67e77903_00318	46.57% ISAzs33_aa2	100% IS_67e77903_01884	IS3 ssgr IS150	339779	3406	822
SEPPX05	IS_67e77903_00340	46.20% ISAzs33_aa2	100% IS_67e77903_02950	IS3 ssgr IS150	357028	357849	822
SEPPX05	IS_67e77903_00575	46.20% ISAzs33_aa2	100% IS_67e77903_02950	IS3 ssgr IS150	595175	595996	822
SEPPX05	IS_67e77903_00587	46.20% ISAzs33_aa2	100% IS_67e77903_02950	IS3 ssgr IS150	606756	605935	822
SEPPX05	IS_67e77903_00606	46.20% ISAzs33_aa2	100% IS_67e77903_02950	IS3 ssgr IS150	625951	62513	822
SEPPX05	IS_67e77903_00846	46.57% ISAzs33_aa2	100% IS_67e77903_01884	IS3 ssgr IS150	873824	874645	822
SEPPX05	IS_67e77903_01096	46.20% ISAzs33_aa2	100% IS_67e77903_02950	IS3 ssgr IS150	1130910	1130089	822
SEPPX05	IS_67e77903_01180	46.20% ISAzs33_aa2	100% IS_67e77903_02950	IS3 ssgr IS150	1207304	1208125	822
SEPPX05	IS_67e77903_01273	46.57% ISAzs33_aa2	100% IS_67e77903_01884	IS3 ssgr IS150	1309216	1308395	822
SEPPX05	IS_67e77903_01390	44.44% ISAzs33_aa2	100% IS_67e77903_02950	IS3 ssgr IS150	1440860	1441636	777
SEPPX05	IS_67e77903_01884	46.57% ISAzs33_aa2	100% IS_67e77903_01273	IS3 ssgr IS150	1948669	1947848	822
SEPPX05	IS_67e77903_02082	46.20% ISAzs33_aa2	100% IS_67e77903_02950	IS3 ssgr IS150	2138998	2139819	822
SEPPX05	IS_67e77903_02090	46.20% ISAzs33_aa2	100% IS_67e77903_02950	IS3 ssgr IS150	2146818	2145997	822
SEPPX05	IS_67e77903_02781	100% ISRso11_aa1	100% IS_67e77903_03729	IS3 ssgr IS150	2847918	2847403	516
SEPPX05	IS_67e77903_02950	46.20% ISAzs33_aa2	100% IS_67e77903_02090	IS3 ssgr IS150	3089071	3089892	822
SEPPX05	IS_67e77903_02991	98.92% ISRso11_aa2	100% IS_67e77903_03730	IS3 ssgr IS150	3139736	3140572	837
SEPPX05	IS_67e77903_03595	100% ISRso11_aa1	100% IS_67e77903_03729	IS3 ssgr IS150	3770279	3770812	534
SEPPX05	IS_67e77903_03596	98.92% ISRso11_aa2	100% IS_67e77903_03730	IS3 ssgr IS150	3770809	3771645	837
SEPPX05	IS_67e77903_03729	100% ISRso11_aa1	100% IS_67e77903_03595	IS3 ssgr IS150	3908201	3908734	534
SEPPX05	IS_67e77903_03730	98.92% ISRso11_aa2	100% IS_67e77903_03596	IS3 ssgr IS150	3908731	3909567	837
SEPPX05	IS_67e77903_00069	92.80% ISButh1_aa2	100% IS_67e77903_03363	IS3 ssgr IS2	73012	72176	837
SEPPX05	IS_67e77903_00070	95.48% ISButh1_aa1	100% IS_67e77903_02998	IS3 ssgr IS2	7341	73009	402
SEPPX05	IS_67e77903_00315	95.48% ISButh1_aa1	100% IS_67e77903_02998	IS3 ssgr IS2	338753	339154	402
SEPPX05	IS_67e77903_00316	90% ISButh1_aa2	100% IS_67e77903_03363	IS3 ssgr IS2	339151	339423	273
SEPPX05	IS_67e77903_00338	94.25% ISButh1_aa1	100% IS_67e77903_02998	IS3 ssgr IS2	356406	356672	267
SEPPX05	IS_67e77903_00341	92.92% ISButh1_aa2	99.55% IS_67e77903_03363	IS3 ssgr IS2	358018	358707	690
SEPPX05	IS_67e77903_00344	91.07% ISButh1_aa2	100% IS_67e77903_03363	IS3 ssgr IS2	360646	360816	171

SEPPX05	IS_67e77903_00877	98.48% ISButh1_aa1	100% IS_67e77903_03698	IS3 ssgr IS2	916928	917329	402
SEPPX05	IS_67e77903_00878	93.52% ISButh1_aa2	99.64% IS_67e77903_03699	IS3 ssgr IS2	917326	918162	837
SEPPX05	IS_67e77903_01185	92.80% ISButh1_aa2	100% IS_67e77903_03363	IS3 ssgr IS2	1216475	1215639	837
SEPPX05	IS_67e77903_01186	95.48% ISButh1_aa1	100% IS_67e77903_02998	IS3 ssgr IS2	1216873	1216472	402
SEPPX05	IS_67e77903_01271	92.80% ISButh1_aa2	100% IS_67e77903_03363	IS3 ssgr IS2	1308125	1307289	837
SEPPX05	IS_67e77903_01272	97.53% ISButh1_aa1	100% IS_67e77903_02998	IS3 ssgr IS2	1308367	1308122	246
SEPPX05	IS_67e77903_01405	92.80% ISButh1_aa2	100% IS_67e77903_03363	IS3 ssgr IS2	1455290	1454454	837
SEPPX05	IS_67e77903_01406	95.48% ISButh1_aa1	100% IS_67e77903_02998	IS3 ssgr IS2	1455688	1455287	402
SEPPX05	IS_67e77903_01446	95.48% ISButh1_aa1	100% IS_67e77903_02998	IS3 ssgr IS2	1504379	1504780	402
SEPPX05	IS_67e77903_01447	92.80% ISButh1_aa2	100% IS_67e77903_03363	IS3 ssgr IS2	1504777	1505613	837
SEPPX05	IS_67e77903_01875	95.48% ISButh1_aa1	100% IS_67e77903_02998	IS3 ssgr IS2	1941258	1941659	402
SEPPX05	IS_67e77903_01876	91.26% ISButh1_aa2	100% IS_67e77903_03363	IS3 ssgr IS2	1941656	1942036	381
SEPPX05	IS_67e77903_01879	94.15% ISButh1_aa3	99.35% IS_67e77903_03363	IS3 ssgr IS2	1943216	1943731	516
SEPPX05	IS_67e77903_01968	92.80% ISButh1_aa2	100% IS_67e77903_03363	IS3 ssgr IS2	2031795	2030959	837
SEPPX05	IS_67e77903_01969	95.48% ISButh1_aa1	100% IS_67e77903_02998	IS3 ssgr IS2	2032193	2031792	402
SEPPX05	IS_67e77903_02083	95.48% ISButh1_aa1	100% IS_67e77903_02998	IS3 ssgr IS2	2139982	2140383	402
SEPPX05	IS_67e77903_02084	92.80% ISButh1_aa2	100% IS_67e77903_03363	IS3 ssgr IS2	2140380	2141216	837
SEPPX05	IS_67e77903_02172	98.37% ISButh1_aa1	100% IS_67e77903_03698	IS3 ssgr IS2	2222738	2223112	375
SEPPX05	IS_67e77903_02173	93.88% ISButh1_aa2	100% IS_67e77903_03699	IS3 ssgr IS2	2223109	2223945	837
SEPPX05	IS_67e77903_02507	95.48% ISButh1_aa1	100% IS_67e77903_02998	IS3 ssgr IS2	2572460	2572861	402
SEPPX05	IS_67e77903_02508	92.80% ISButh1_aa2	100% IS_67e77903_03363	IS3 ssgr IS2	2572858	2573694	837
SEPPX05	IS_67e77903_02838	95.48% ISButh1_aa1	100% IS_67e77903_02998	IS3 ssgr IS2	2907698	2908099	402
SEPPX05	IS_67e77903_02839	92.80% ISButh1_aa2	100% IS_67e77903_03363	IS3 ssgr IS2	2908096	2908932	837
SEPPX05	IS_67e77903_02997	92.80% ISButh1_aa2	100% IS_67e77903_03363	IS3 ssgr IS2	3148400	3147564	837
SEPPX05	IS_67e77903_02998	95.48% ISButh1_aa1	100% IS_67e77903_02838	IS3 ssgr IS2	3148798	3148397	402
SEPPX05	IS_67e77903_03354	92.80% ISButh1_aa2	100% IS_67e77903_03363	IS3 ssgr IS2	3508144	3507308	837
SEPPX05	IS_67e77903_03355	95.96% ISButh1_aa1	100% IS_67e77903_03362	IS3 ssgr IS2	3508515	3508141	375
SEPPX05	IS_67e77903_03362	95.96% ISButh1_aa1	100% IS_67e77903_03355	IS3 ssgr IS2	3513145	3513519	375
SEPPX05	IS_67e77903_03363	92.80% ISButh1_aa2	100% IS_67e77903_03354	IS3 ssgr IS2	3513516	3514352	837
SEPPX05	IS_67e77903_03698	98.48% ISButh1_aa1	100% IS_67e77903_00877	IS3 ssgr IS2	3876031	3876432	402
SEPPX05	IS_67e77903_03699	93.88% ISButh1_aa2	100% IS_67e77903_02173	IS3 ssgr IS2	3876429	3877265	837
SEPPX05	IS_67e77903_00581	54.16% ISBce13_aa2	100% IS_67e77903_02950	IS3 ssgr IS3	600429	601091	663
SEPPX05	IS_67e77903_00589	85.39% IS222_aa1	100% IS_67e77903_02080	IS3 ssgr IS3	607388	607783	396
SEPPX05	IS_67e77903_02075	88.78% IS222_aa2	50.71% IS_67e77903_03730	IS3 ssgr IS3	2134544	2133873	672
SEPPX05	IS_67e77903_02080	85.39% IS222_aa1	100% IS_67e77903_00589	IS3 ssgr IS3	2138366	2137971	396

SEPPX05	IS_67e77903_00573	100% ISRso14_aa1	100% IS_67e77903_03374	IS3 ssgr IS407	594302	594565	264
SEPPX05	IS_67e77903_00576	100% ISRso14_aa2	100% IS_67e77903_03375	IS3 ssgr IS407	596069	596617	549
SEPPX05	IS_67e77903_00582	100% ISRso14_aa2	100% IS_67e77903_03375	IS3 ssgr IS407	601164	601712	549
SEPPX05	IS_67e77903_00928	100% ISRso14_aa1	100% IS_67e77903_03374	IS3 ssgr IS407	95583	956093	264
SEPPX05	IS_67e77903_00929	100% ISRso14_aa2	100% IS_67e77903_03375	IS3 ssgr IS407	956399	956947	549
SEPPX05	IS_67e77903_01192	100% ISRso14_aa2	100% IS_67e77903_03375	IS3 ssgr IS407	1220978	1221526	549
SEPPX05	IS_67e77903_01636	93.18% ISRso16_aa2	No hit	IS3 ssgr IS407	1692647	1692979	333
SEPPX05	IS_67e77903_01856	85.71% ISAtu5_aa3	79.51% IS_67e77903_03374	IS3 ssgr IS407	1922734	1922345	390
SEPPX05	IS_67e77903_01877	100% ISRso14_aa1	100% IS_67e77903_03374	IS3 ssgr IS407	1942102	1942365	264
SEPPX05	IS_67e77903_01878	100% ISRso14_aa2	100% IS_67e77903_03375	IS3 ssgr IS407	1942671	1943219	549
SEPPX05	IS_67e77903_02089	100% ISRso14_aa2	100% IS_67e77903_03375	IS3 ssgr IS407	2145924	2145376	549
SEPPX05	IS_67e77903_02092	100% ISRso14_aa1	100% IS_67e77903_03374	IS3 ssgr IS407	2147691	2147428	264
SEPPX05	IS_67e77903_02165	100% ISRso14_aa1	100% IS_67e77903_03374	IS3 ssgr IS407	2219699	2219962	264
SEPPX05	IS_67e77903_02166	100% ISRso14_aa2	100% IS_67e77903_03375	IS3 ssgr IS407	2220268	2220816	549
SEPPX05	IS_67e77903_02510	100% ISRso14_aa2	100% IS_67e77903_03375	IS3 ssgr IS407	2574934	2574386	549
SEPPX05	IS_67e77903_02511	100% ISRso14_aa1	100% IS_67e77903_03374	IS3 ssgr IS407	2575503	2575240	264
SEPPX05	IS_67e77903_03105	100% ISRso14_aa2	100% IS_67e77903_03375	IS3 ssgr IS407	3262422	3261874	549
SEPPX05	IS_67e77903_03106	100% ISRso14_aa1	100% IS_67e77903_03374	IS3 ssgr IS407	3262991	3262728	264
SEPPX05	IS_67e77903_03374	100% ISRso14_aa1	100% IS_67e77903_03106	IS3 ssgr IS407	3522036	3522299	264
SEPPX05	IS_67e77903_03375	100% ISRso14_aa2	100% IS_67e77903_03105	IS3 ssgr IS407	3522605	3523153	549
SEPPX05	IS_67e77903_00402	61.68% ISCro3_aa1	100% IS_67e77903_03600	IS4	418986	420314	1.329
SEPPX05	IS_67e77903_00593	62.03% ISCro3_aa1	96.50% IS_67e77903_03600	IS4	611381	610104	1.278
SEPPX05	IS_67e77903_00760	61.44% ISCro3_aa1	99.77% IS_67e77903_03600	IS4	772226	770898	1.329
SEPPX05	IS_67e77903_00767	61.68% ISCro3_aa1	100% IS_67e77903_03600	IS4	778981	777653	1.329
SEPPX05	IS_67e77903_00835	61.68% ISCro3_aa1	100% IS_67e77903_03600	IS4	858063	859391	1.329
SEPPX05	IS_67e77903_01355	61.68% ISCro3_aa1	100% IS_67e77903_03600	IS4	1400712	1399384	1.329
SEPPX05	IS_67e77903_01442	61.68% ISCro3_aa1	100% IS_67e77903_03600	IS4	1496346	1495018	1.329
SEPPX05	IS_67e77903_01479	61.68% ISCro3_aa1	100% IS_67e77903_03600	IS4	1540349	1539021	1.329
SEPPX05	IS_67e77903_01499	61.68% ISCro3_aa1	100% IS_67e77903_03600	IS4	1563079	1564407	1.329
SEPPX05	IS_67e77903_01991	63.78% ISCro3_aa1	100% IS_67e77903_03600	IS4	2052436	2053539	1.104
SEPPX05	IS_67e77903_02119	61.68% ISCro3_aa1	100% IS_67e77903_03600	IS4	2178371	2179699	1.329
SEPPX05	IS_67e77903_02231	61.68% ISCro3_aa1	100% IS_67e77903_03600	IS4	2285584	2284256	1.329
SEPPX05	IS_67e77903_02289	61.68% ISCro3_aa1	100% IS_67e77903_03600	IS4	2343518	2344846	1.329
SEPPX05	IS_67e77903_03419	61.68% ISCro3_aa1	100% IS_67e77903_03600	IS4	3565525	3564197	1.329
SEPPX05	IS_67e77903_03600	61.68% ISCro3_aa1	100% IS_67e77903_03419	IS4	3776841	3775513	1.329

SEPPX05	IS_67e77903_01349	58.27% ISCro6_aa1	100% IS_67e77903_03600	IS4 ssgr IS4	1391824	1391324	501
SEPPX05	IS_67e77903_03172	45.84% ISAzs36_aa2	No hit	IS481	3331658	3330372	1.287
SEPPX05	IS_67e77903_03173	41.66% ISAzs36_aa1	No hit	IS481	3333457	3331658	1.8
SEPPX05	IS_67e77903_00660	100% IS1421_aa1	100% IS_67e77903_03622	IS5 ssgr IS427	664501	664097	405
SEPPX05	IS_67e77903_00853	98.50% IS1421_aa1	100% IS_67e77903_01848	IS5 ssgr IS427	88144	881036	405
SEPPX05	IS_67e77903_00932	100% IS1421_aa1	100% IS_67e77903_03622	IS5 ssgr IS427	958198	958602	405
SEPPX05	IS_67e77903_00933	86.06% IS1421_aa1	100% IS_67e77903_03122	IS5 ssgr IS427	959062	959469	408
SEPPX05	IS_67e77903_01334	86.06% IS1421_aa1	100% IS_67e77903_03122	IS5 ssgr IS427	1378364	1378771	408
SEPPX05	IS_67e77903_01356	100% IS1421_aa1	100% IS_67e77903_03622	IS5 ssgr IS427	1401057	1401461	405
SEPPX05	IS_67e77903_01394	86.06% IS1421_aa1	100% IS_67e77903_03122	IS5 ssgr IS427	1445431	1445024	408
SEPPX05	IS_67e77903_01395	100% IS1421_aa1	100% IS_67e77903_03622	IS5 ssgr IS427	1446126	1446530	405
SEPPX05	IS_67e77903_01720	86.06% IS1421_aa1	100% IS_67e77903_03122	IS5 ssgr IS427	1779720	1779313	408
SEPPX05	IS_67e77903_01755	86.06% IS1421_aa1	100% IS_67e77903_03122	IS5 ssgr IS427	1815773	1815366	408
SEPPX05	IS_67e77903_01763	86.06% IS1421_aa1	100% IS_67e77903_03122	IS5 ssgr IS427	1825270	1825677	408
SEPPX05	IS_67e77903_01848	98.50% IS1421_aa1	100% IS_67e77903_00853	IS5 ssgr IS427	1912077	1912481	405
SEPPX05	IS_67e77903_02391	86.06% IS1421_aa1	100% IS_67e77903_03122	IS5 ssgr IS427	2452017	2452424	408
SEPPX05	IS_67e77903_02710	86.06% IS1421_aa1	100% IS_67e77903_03122	IS5 ssgr IS427	2765978	2766385	408
SEPPX05	IS_67e77903_02927	100% IS1421_aa1	100% IS_67e77903_03622	IS5 ssgr IS427	3063522	3063926	405
SEPPX05	IS_67e77903_03122	86.06% IS1421_aa1	100% IS_67e77903_02710	IS5 ssgr IS427	3282407	3282000	408
SEPPX05	IS_67e77903_03622	100% IS1421_aa1	100% IS_67e77903_02927	IS5 ssgr IS427	3797586	3797182	405
SEPPX05	IS_67e77903_00475	100% IS1405_aa1	100% IS_67e77903_01490	IS5 ssgr IS5	486602	486153	450
SEPPX05	IS_67e77903_00715	100% IS1405_aa1	100% IS_67e77903_01490	IS5 ssgr IS5	721088	720639	450
SEPPX05	IS_67e77903_00717	98.81% IS1405_aa1	100% IS_67e77903_03049	IS5 ssgr IS5	723454	722792	663
SEPPX05	IS_67e77903_00859	85.71% ISAau3_aa1	80.28% IS_67e77903_01443	IS5 ssgr IS5	89017	889877	294
SEPPX05	IS_67e77903_01443	99.06% IS1405_aa1	100% IS_67e77903_03049	IS5 ssgr IS5	1497427	1496462	966
SEPPX05	IS_67e77903_01486	98.81% IS1405_aa1	100% IS_67e77903_03049	IS5 ssgr IS5	1550209	1550871	663
SEPPX05	IS_67e77903_01490	100% IS1405_aa1	100% IS_67e77903_00715	IS5 ssgr IS5	1554537	1554986	450
SEPPX05	IS_67e77903_03049	98.81% IS1405_aa1	100% IS_67e77903_01486	IS5 ssgr IS5	3205467	3204805	663
SEPPX05	IS_67e77903_01184	89.37% ISBcen14_aa3	100% IS_67e77903_01880	IS66	1215574	1214810	765
SEPPX05	IS_67e77903_01187	80.14% ISBcen14_aa3	100% IS_67e77903_01880	IS66	1217745	1216915	831
SEPPX05	IS_67e77903_01188	93.04% ISBcen14_aa2	100% IS_67e77903_01881	IS66	1218144	1217797	348
SEPPX05	IS_67e77903_01880	84.45% ISBcen14_aa3	100% IS_67e77903_01187	IS66	1945437	1943836	1.602
SEPPX05	IS_67e77903_01881	93.04% ISBcen14_aa2	100% IS_67e77903_01188	IS66	1945836	1945489	348
SEPPX05	IS_67e77903_01836	100% ISRso17_aa1	100% IS_67e77903_02345	IS701	1901982	1900651	1.332
SEPPX05	IS_67e77903_02345	100% ISRso17_aa1	100% IS_67e77903_01836	IS701	2404300	2405631	1.332

SEPPX05	IS_67e77903_00347	47.48% ISShvi3_aa1	50.41% IS_67e77903_02522	IS91	364025	362979	1.047
SEPPX05	IS_67e77903_00884	45.76% ISWz1_aa1	100% IS_67e77903_01137	IS91	921774	923021	1.248
SEPPX05	IS_67e77903_00886	45.61% ISTha3_aa2	100% IS_67e77903_01139	IS91	923917	924936	1.02
SEPPX05	IS_67e77903_01137	45.76% ISWz1_aa1	100% IS_67e77903_00884	IS91	1162349	1163596	1.248
SEPPX05	IS_67e77903_01139	45.61% ISTha3_aa2	100% IS_67e77903_00886	IS91	1164492	1165511	1.02
SEPPX05	IS_67e77903_01326	43% ISMno24_aa2	52.21% IS_67e77903_03719	IS91	1369267	1370256	990
SEPPX05	IS_67e77903_02522	47.12% ISTha3_aa2	50.41% IS_67e77903_00347	IS91	2584852	2583710	1.143
SEPPX05	IS_67e77903_03719	47.5% ISTha3_aa2	51.57% IS_67e77903_01326	IS91	3897203	3898129	927
SEPPX05	IS_67e77903_00406	99.75% ISRso15_aa1	100% IS_67e77903_02381	ISL3	42363	42241	1.221
SEPPX05	IS_67e77903_00569	99.75% ISRso15_aa1	100% IS_67e77903_02381	ISL3	59162	5904	1.221
SEPPX05	IS_67e77903_00943	38.97% ISKpn25_aa1	No hit	ISL3	968753	970198	1.446
SEPPX05	IS_67e77903_01411	99.75% ISRso15_aa1	100% IS_67e77903_02381	ISL3	1459297	1460517	1.221
SEPPX05	IS_67e77903_01430	99.75% ISRso15_aa1	100% IS_67e77903_02381	ISL3	1480783	1479563	1.221
SEPPX05	IS_67e77903_01847	99.75% ISRso15_aa1	100% IS_67e77903_02381	ISL3	1910572	1911792	1.221
SEPPX05	IS_67e77903_02100	99.75% ISRso15_aa1	100% IS_67e77903_02381	ISL3	2158092	2156872	1.221
SEPPX05	IS_67e77903_02381	99.75% ISRso15_aa1	100% IS_67e77903_02100	ISL3	2439703	2438483	1.221
SEPPX05	IS_67e77903_02844	64.60% ISKpn21_aa1	No hit	ISNCY ssgr IS1202	2913156	2913548	393
SEPPX05	IS_67e77903_00579	56.29% ISMpo10_aa3	100% IS_67e77903_02086	Tn3	59971	598526	1.185
SEPPX05	IS_67e77903_00585	51.83% ISMpo10_aa3	100% IS_67e77903_02086	Tn3	605003	603621	1.383
SEPPX05	IS_67e77903_00926	54.71% ISMpo10_aa3	96.32% IS_67e77903_01104	Tn3	953542	95493	1.389
SEPPX05	IS_67e77903_01104	61.81% ISMpo10_aa3	96.32% IS_67e77903_00926	Tn3	1136955	1135567	1.389
SEPPX05	IS_67e77903_02086	51.83% ISMpo10_aa3	100% IS_67e77903_00585	Tn3	2142085	2143467	1.383
SEPPX05	IS_67e77903_02164	83.75% ISSba14_aa1	100% IS_67e77903_03377	Tn3	2219509	2219264	246
SEPPX05	IS_67e77903_02167	83.75% ISYps3_aa2	100% IS_67e77903_03377	Tn3	2221104	2220862	243
SEPPX05	IS_67e77903_02965	66.77% ISPa43_aa2	No hit	Tn3	3106780	3109503	2.724
SEPPX05	IS_67e77903_03111	75.15% ISPa40_aa4	62.96% IS_67e77903_03516	Tn3	3268309	3267809	501
SEPPX05	IS_67e77903_03377	83.41% ISSba14_aa1	100% IS_67e77903_02167	Tn3	3524872	3524288	585
SEPPX05	IS_67e77903_03516	61.87% ISPa38_aa1	52.57% IS_67e77903_03377	Tn3	3682634	3682044	591
Rs_10_244	IS_78626702_03173	87.92% ISAisp1_aa1	No hit	IS1595 ssgr ISSod11	3393218	3392247	972
Rs_10_244	IS_78626702_03410	100% ISRso11_aa2	52% IS_78626702_00182	IS3 ssgr IS150	3651311	3650475	837
Rs_10_244	IS_78626702_03411	100% ISRso11_aa1	No hit	IS3 ssgr IS150	3651841	3651308	534
Rs_10_244	IS_78626702_00193	94.38% ISButh1_aa2	94.01% IS_78626702_00835	IS3 ssgr IS2	211459	210869	591
Rs_10_244	IS_78626702_00194	89.33% ISButh1_aa2	87.17% IS_78626702_00834	IS3 ssgr IS2	211966	21173	237
Rs_10_244	IS_78626702_00195	95.96% ISButh1_aa1	94.35% IS_78626702_00833	IS3 ssgr IS2	212337	211963	375

Rs_10_244	IS_78626702_00833	98.48% ISButh1_aa1	95.12% IS_78626702_00195	IS3 ssgr IS2	861784	862185	402
Rs_10_244	IS_78626702_00834	89.33% ISButh1_aa2	87.17% IS_78626702_00194	IS3 ssgr IS2	862182	862418	237
Rs_10_244	IS_78626702_00835	94.87% ISButh1_aa2	94.01% IS_78626702_00193	IS3 ssgr IS2	862921	863274	354
Rs_10_244	IS_78626702_02100	63.15% ISAve4_aa1	No hit	IS3 ssgr IS3	2257768	2257583	186
Rs_10_244	IS_78626702_00429	91.80% ISRso16_aa2	No hit	IS3 ssgr IS407	447936	448127	192
Rs_10_244	IS_78626702_00631	75.80% ISSme1_aa1	69.84% IS_78626702_01545	IS3 ssgr IS407	657071	656874	198
Rs_10_244	IS_78626702_01545	100% ISRso14_aa1	69.84% IS_78626702_00631	IS3 ssgr IS407	1614687	1614424	264
Rs_10_244	IS_78626702_03394	99.45% ISRso14_aa2	41.07% IS_78626702_03410	IS3 ssgr IS407	3631226	3630678	549
Rs_10_244	IS_78626702_00181	81.52% ISAisp2_aa1	No hit	IS3 ssgr IS51	20136	201638	279
Rs_10_244	IS_78626702_00182	89.34% ISAisp2_aa2	52% IS_78626702_03410	IS3 ssgr IS51	201635	20251	876
Rs_10_244	IS_78626702_00337	46.29% ISAcp2_aa1	No hit	IS481	360334	362235	1.902
Rs_10_244	IS_78626702_03128	97.44% ISRso1_aa1	97.08% IS_78626702_03377	IS5	3348700	3349524	825
Rs_10_244	IS_78626702_03368	96.96% ISRso1_aa1	96.96% IS_78626702_03377	IS5	3603756	3603556	201
Rs_10_244	IS_78626702_03377	97.44% ISRso1_aa1	97.08% IS_78626702_03128	IS5	3612649	3611825	825
Rs_10_244	IS_78626702_01677	68.75% ISCARN39_aa2	No hit	IS630	1757264	1757464	201
Rs_10_244	IS_78626702_00053	47.76% ISWz1_aa1	51.73% IS_78626702_02458	IS91	58474	57485	990
Rs_10_244	IS_78626702_01861	44.8% ISTha3_aa2	57.69% IS_78626702_01463	IS91	2008545	2007535	1.011
Rs_10_244	IS_78626702_02458	47.5% ISTha3_aa2	51.09% IS_78626702_00053	IS91	2649850	2650770	921
Rs_10_244	IS_78626702_03323	38.97% ISKpn25_aa1	No hit	ISL3	3554502	3553057	1.446
Rs_10_244	IS_78626702_01799	63.71% ISKpn21_aa1	No hit	ISNCY ssgr IS1202	1941929	1941540	390
Rs_10_244	IS_78626702_00884	54.43% ISMpo10_aa3	91.27% IS_78626702_03392	Tn3	915685	917067	1.383
Rs_10_244	IS_78626702_01111	85.18% ISYps3_aa2	53.59% IS_78626702_03366	Tn3	1146337	1145747	591
Rs_10_244	IS_78626702_03168	54.71% ISMpo10_aa3	73.03% IS_78626702_00884	Tn3	3387306	3388694	1.389
Rs_10_244	IS_78626702_03366	84.15% ISPa40_aa4	53.59% IS_78626702_01111	Tn3	3602988	3602425	564
Rs_10_244	IS_78626702_03392	51.46% ISMpo10_aa3	91.27% IS_78626702_00884	Tn3	3627164	3628537	1.374
Rs_09_16	IS_ef62fa1c_03191	50.51% ISHvo5_aa1	47.05% IS_ef62fa1c_00890	IS1595 ssgr ISH4	3402043	3401255	789
Rs_09_16	IS_ef62fa1c_00890	50.44% ISLsp2_aa1	48.10% IS_ef62fa1c_00911	IS1595 ssgr ISPna2	90834	909626	1.287
Rs_09_16	IS_ef62fa1c_02220	93.16% ISButh1_aa2	No hit	IS3 ssgr IS2	2354191	2355027	837
Rs_09_16	IS_ef62fa1c_00828	90.13% IS222_aa2	No hit	IS3 ssgr IS3	856142	855471	672
Rs_09_16	IS_ef62fa1c_00630	85.54% ISAtu5_aa1	68.29% IS_ef62fa1c_00905	IS3 ssgr IS407	661165	660776	390
Rs_09_16	IS_ef62fa1c_00905	100% ISRso12_aa1	100% IS_ef62fa1c_01915	IS3 ssgr IS407	925989	926255	267
Rs_09_16	IS_ef62fa1c_01915	100% ISRso12_aa1	100% IS_ef62fa1c_00905	IS3 ssgr IS407	2044513	2044779	267
Rs_09_16	IS_ef62fa1c_00891	81.03% ISBcen26_aa1	No hit	IS481	910121	909771	351
Rs_09_16	IS_ef62fa1c_03412	97.44% ISRso1_aa1	No hit	IS5	3659985	3660809	825

Rs_09_16	IS_ef62fa1c_00893	81.35% ISCARN39_aa3	No hit	IS630	912483	911947	537
Rs_09_16	IS_ef62fa1c_01000	77.77% ISRso5_aa1	No hit	IS630	1023090	1023635	546
Rs_09_16	IS_ef62fa1c_01001	92.75% ISRso5_aa1	No hit	IS630	1023807	1024451	645
Rs_09_16	IS_ef62fa1c_01742	67.18% ISCARN39_aa2	No hit	IS630	1804293	1804493	201
Rs_09_16	IS_ef62fa1c_00054	43.33% ISMno24_aa2	52.12% IS_ef62fa1c_02566	IS91	58624	57635	990
Rs_09_16	IS_ef62fa1c_02566	47.5% ISTha3_aa2	51.50% IS_ef62fa1c_00054	IS91	2736982	2737908	927
Rs_09_16	IS_ef62fa1c_02931	45% ISShvi3_aa1	51.26% IS_ef62fa1c_01136	IS91	3126842	3125931	912
Rs_09_16	IS_ef62fa1c_03344	38.97% ISKpn25_aa1	No hit	ISL3	3567115	3565670	1.446
Rs_09_16	IS_ef62fa1c_01861	64.60% ISKpn21_aa1	No hit	ISNCY ssgr IS1202	1988333	1987932	402
Rs_09_16	IS_ef62fa1c_00913	54.43% ISMpo10_aa3	73.09% IS_ef62fa1c_03194	Tn3	932306	933688	1.383
Rs_09_16	IS_ef62fa1c_02370	61.87% ISPa38_aa1	54.90% IS_ef62fa1c_03194	Tn3	2526528	2525938	591
Rs_09_16	IS_ef62fa1c_02758	52.59% TnShfr1_aa1	No hit	Tn3	2941354	2940317	1.038
Rs_09_16	IS_ef62fa1c_03094	44.44% ISNpu13_aa2	44.37% IS_ef62fa1c_02931	Tn3	3287856	3286774	1.083
Rs_09_16	IS_ef62fa1c_03194	54.71% ISMpo10_aa3	73.09% IS_ef62fa1c_00913	Tn3	3404954	3406342	1.389
SL3103	CP022790_00221	88.75% ISBcen4_aa1	100% CP022790_03171	IS110 ssgr IS1111	229897	230916	1.02
SL3103	CP022790_02829	61.67% ISMno14_aa1	45.80% CP022790_03171	IS110 ssgr IS1111	3005859	3004846	1.014
SL3103	CP022790_03171	88.75% ISBcen4_aa1	100% CP022790_00221	IS110 ssgr IS1111	3337224	3336205	1.02
SL3103	CP022790_01036	83.75% ISBusp4_aa1	100% CP022790_02619	IS1182	1107005	1105560	1.446
SL3103	CP022790_01356	82.73% ISBusp4_aa1	100% CP022790_02619	IS1182	1454545	1455801	1.257
SL3103	CP022790_02170	83.75% ISBusp4_aa1	100% CP022790_02619	IS1182	2327195	2328640	1.446
SL3103	CP022790_02619	83.75% ISBusp4_aa1	100% CP022790_02170	IS1182	2788087	2786642	1.446
SL3103	CP022790_00934	54.76% ISHpa1_aa1	No hit	IS1595 ssgr IS1016	995158	995937	780
SL3103	CP022790_00256	50.44% ISLsp2_aa1	48.34% CP022790_02372	IS1595 ssgr ISPna2	278406	279683	1.278
SL3103	CP022790_00316	100% ISRso11_aa1	No hit	IS3 ssgr IS150	352743	353276	534
SL3103	CP022790_00317	99.64% ISRso11_aa2	100% CP022790_00868	IS3 ssgr IS150	353273	354109	837
SL3103	CP022790_00868	100% ISRso11_aa2	100% CP022790_00317	IS3 ssgr IS150	927716	928315	600
SL3103	CP022790_00220	97.87% ISRso10_aa1	96.96% CP022790_03172	IS3 ssgr IS2	229542	229922	381
SL3103	CP022790_00222	99.64% ISRso10_aa2	40% CP022790_00317	IS3 ssgr IS2	231292	23214	849
SL3103	CP022790_01086	89.06% IS222_aa2	No hit	IS3 ssgr IS3	1160203	1160397	195
SL3103	CP022790_01039	89% ISAisp2_aa2	95.21% CP022790_03083	IS3 ssgr IS51	1109348	1108686	663
SL3103	CP022790_01040	81.52% ISAisp2_aa1	97.82% CP022790_03084	IS3 ssgr IS51	1109623	1109345	279
SL3103	CP022790_01078	86.38% ISAisp2_aa2	95.71% CP022790_03083	IS3 ssgr IS51	1154028	1153237	792
SL3103	CP022790_01079	80.43% ISAisp2_aa1	96.73% CP022790_03084	IS3 ssgr IS51	1154303	1154025	279
SL3103	CP022790_03083	89.34% ISAisp2_aa2	95.71% CP022790_01078	IS3 ssgr IS51	3254670	3253795	876

SL3103	CP022790_03084	81.52% ISAisp2_aa1	97.82% CP022790_01040	IS3 ssgr IS51	3254945	3254667	279
SL3103	CP022790_00280	61.68% ISCro3_aa1	100% CP022790_03269	IS4	308415	309743	1.329
SL3103	CP022790_00341	61.68% ISCro3_aa1	100% CP022790_03269	IS4	381571	382899	1.329
SL3103	CP022790_00352	61.68% ISCro3_aa1	100% CP022790_03269	IS4	394092	392764	1.329
SL3103	CP022790_00470	61.68% ISCro3_aa1	100% CP022790_03269	IS4	510636	509308	1.329
SL3103	CP022790_00510	61.68% ISCro3_aa1	100% CP022790_03269	IS4	549134	550462	1.329
SL3103	CP022790_00991	61.68% ISCro3_aa1	100% CP022790_03269	IS4	1056274	1054946	1.329
SL3103	CP022790_01167	61.68% ISCro3_aa1	100% CP022790_03269	IS4	1256131	1254803	1.329
SL3103	CP022790_01707	61.68% ISCro3_aa1	100% CP022790_03263	IS4	1807470	1808798	1.329
SL3103	CP022790_02196	61.68% ISCro3_aa1	100% CP022790_03269	IS4	2357948	2356620	1.329
SL3103	CP022790_02252	61.68% ISCro3_aa1	100% CP022790_03263	IS4	2414423	2413095	1.329
SL3103	CP022790_02516	61.68% ISCro3_aa1	100% CP022790_03269	IS4	2686670	2687998	1.329
SL3103	CP022790_03263	61.68% ISCro3_aa1	100% CP022790_02252	IS4	3443076	3444404	1.329
SL3103	CP022790_03269	61.68% ISCro3_aa1	100% CP022790_02516	IS4	3452390	3451062	1.329
SL3103	CP022790_00238	97.81% ISRso1_aa1	100% CP022790_01675	IS5	248601	249425	825
SL3103	CP022790_00267	97.81% ISRso1_aa1	100% CP022790_01675	IS5	291149	291973	825
SL3103	CP022790_01675	97.81% ISRso1_aa1	100% CP022790_00267	IS5	1781209	1780385	825
SL3103	CP022790_03087	98.66% ISRso1_aa1	100% CP022790_01675	IS5	3257010	3256333	678
SL3103	CP022790_02421	100% IS1421_aa1	No hit	IS5 ssgr IS427	2594379	2594762	384
SL3103	CP022790_00008	99.06% IS1405_aa1	100% CP022790_03108	IS5 ssgr IS5	13611	14576	966
SL3103	CP022790_00022	94.81% IS1021_aa1	100% CP022790_02434	IS5 ssgr IS5	26792	25806	987
SL3103	CP022790_00196	99.06% IS1405_aa1	100% CP022790_03108	IS5 ssgr IS5	210527	211492	966
SL3103	CP022790_00257	92.07% ISRso9_aa1	No hit	IS5 ssgr IS5	280049	280354	306
SL3103	CP022790_00264	99.06% IS1405_aa1	100% CP022790_03108	IS5 ssgr IS5	289137	290102	966
SL3103	CP022790_00285	99.37% IS1405_aa1	100% CP022790_02925	IS5 ssgr IS5	314282	315247	966
SL3103	CP022790_00759	94.81% IS1021_aa1	100% CP022790_02434	IS5 ssgr IS5	820768	821754	987
SL3103	CP022790_01285	99.06% IS1405_aa1	100% CP022790_03108	IS5 ssgr IS5	1383074	1382109	966
SL3103	CP022790_02407	99.37% IS1405_aa1	100% CP022790_02925	IS5 ssgr IS5	2581104	2582069	966
SL3103	CP022790_02434	94.81% IS1021_aa1	100% CP022790_00759	IS5 ssgr IS5	2605447	2606433	987
SL3103	CP022790_02435	99.37% IS1405_aa1	100% CP022790_02925	IS5 ssgr IS5	2606988	2607953	966
SL3103	CP022790_02712	99.37% IS1405_aa1	100% CP022790_02925	IS5 ssgr IS5	2883235	2882270	966
SL3103	CP022790_02925	99.37% IS1405_aa1	100% CP022790_02712	IS5 ssgr IS5	3094410	3093445	966
SL3103	CP022790_02932	94.69% IS1021_aa1	100% CP022790_02434	IS5 ssgr IS5	3100889	3100143	747
SL3103	CP022790_03005	99.06% IS1405_aa1	100% CP022790_03108	IS5 ssgr IS5	3168817	3167852	966
SL3103	CP022790_03108	99.06% IS1405_aa1	100% CP022790_03005	IS5 ssgr IS5	3275316	3276281	966

SL3103	CP022790_00006	100% IS1420_aa1	100% CP022790_03189	IS5 ssgr IS903	10262	9306	957
SL3103	CP022790_00015	100% IS1420_aa1	100% CP022790_03189	IS5 ssgr IS903	20502	19546	957
SL3103	CP022790_00195	100% IS1420_aa1	100% CP022790_03189	IS5 ssgr IS903	209147	210103	957
SL3103	CP022790_00287	100% IS1420_aa1	100% CP022790_03189	IS5 ssgr IS903	315904	31686	957
SL3103	CP022790_01374	100% IS1420_aa1	100% CP022790_03189	IS5 ssgr IS903	1471254	1472210	957
SL3103	CP022790_01452	100% IS1420_aa1	100% CP022790_03189	IS5 ssgr IS903	1552388	1551432	957
SL3103	CP022790_01784	100% IS1420_aa1	100% CP022790_03189	IS5 ssgr IS903	1897753	1898709	957
SL3103	CP022790_02274	100% IS1420_aa1	100% CP022790_03189	IS5 ssgr IS903	2437680	2438636	957
SL3103	CP022790_02374	100% IS1420_aa1	100% CP022790_03189	IS5 ssgr IS903	2542255	2541299	957
SL3103	CP022790_02386	99.68% IS1420_aa1	99.68% CP022790_03189	IS5 ssgr IS903	2559558	2560514	957
SL3103	CP022790_02661	100% IS1420_aa1	100% CP022790_03189	IS5 ssgr IS903	2830105	2831061	957
SL3103	CP022790_03058	100% IS1420_aa1	100% CP022790_03189	IS5 ssgr IS903	3231792	3230836	957
SL3103	CP022790_03189	100% IS1420_aa1	100% CP022790_03058	IS5 ssgr IS903	3359349	3360305	957
SL3103	CP022790_00011	56.95% ISRm2_aa1	100% CP022790_02936	IS66	15592	16074	483
SL3103	CP022790_00012	73.27% ISRm2_aa2	100% CP022790_02935	IS66	16071	16424	354
SL3103	CP022790_00013	59.79% ISAeh1_aa2	100% CP022790_02934	IS66	16457	18013	1.557
SL3103	CP022790_00226	70.19% IS883_aa1	No hit	IS66	240269	240676	408
SL3103	CP022790_00227	86.95% IS883_aa2	53.15% CP022790_02935	IS66	240673	241017	345
SL3103	CP022790_00228	63.38% IS883_aa3	No hit	IS66	241057	241626	570
SL3103	CP022790_00229	72.46% ISEc8_aa3	43.72% CP022790_01781	IS66	241566	242192	627
SL3103	CP022790_00230	78.74% IS883_aa3	54.19% CP022790_02428	IS66	242196	24266	465
SL3103	CP022790_00231	60.71% ISAba28_aa4	No hit	IS66	242952	242641	312
SL3103	CP022790_00760	59.79% ISAeh1_aa2	100% CP022790_02934	IS66	823594	822038	1.557
SL3103	CP022790_00761	73.27% ISRm2_aa2	100% CP022790_02935	IS66	82398	823627	354
SL3103	CP022790_00762	56.95% ISRm2_aa1	100% CP022790_02936	IS66	824459	823977	483
SL3103	CP022790_01535	56.95% ISRm2_aa1	100% CP022790_02936	IS66	1641372	1641854	483
SL3103	CP022790_01536	73.27% ISRm2_aa2	100% CP022790_02935	IS66	1641851	1642204	354
SL3103	CP022790_01537	59.79% ISAeh1_aa2	100% CP022790_02934	IS66	1642237	1643793	1.557
SL3103	CP022790_01781	62.68% ISAeh1_aa2	100% CP022790_02934	IS66	1894849	1896069	1.221
SL3103	CP022790_02397	56.95% ISRm2_aa1	100% CP022790_02936	IS66	2571738	2572220	483
SL3103	CP022790_02398	73.27% ISRm2_aa2	100% CP022790_02935	IS66	2572217	2572570	354
SL3103	CP022790_02399	59.79% ISAeh1_aa2	100% CP022790_02934	IS66	2572603	2574159	1.557
SL3103	CP022790_02428	60.75% ISPpu13_aa2	95.41% CP022790_02934	IS66	2601446	2599857	1.59
SL3103	CP022790_02429	73.27% ISRm2_aa2	96.58% CP022790_02935	IS66	2601832	2601479	354
SL3103	CP022790_02430	55.55% ISRm2_aa1	69.56% CP022790_02936	IS66	2602293	2601829	465

SL3103	CP022790_02934	59.79% ISAeh1_aa2	100% CP022790_02399	IS66	3104449	3102893	1.557
SL3103	CP022790_02935	73.27% ISRm2_aa2	100% CP022790_02398	IS66	3104835	3104482	354
SL3103	CP022790_02936	56.95% ISRm2_aa1	100% CP022790_02397	IS66	3105314	3104832	483
SL3103	CP022790_00246	42.78% ISWz1_aa1	100% CP022790_01775	IS91	258494	260167	1.674
SL3103	CP022790_00879	51.30% ISMno23_aa1	55.04% CP022790_03241	IS91	938997	938332	666
SL3103	CP022790_01775	42.78% ISWz1_aa1	100% CP022790_00246	IS91	1889233	1887560	1.674
SL3103	CP022790_03241	43.33% ISMno24_aa2	55.93% CP022790_00879	IS91	3421792	3422781	990
SL3103	CP022790_00044	38.97% ISKpn25_aa1	No hit	ISL3	49904	51349	1.446
SL3103	CP022790_01786	62.5% ISAba32_aa1	70% CP022790_01787	ISNCY ssgr IS1202	1900108	1899893	216
SL3103	CP022790_01787	72.27% ISKpn21_aa1	70% CP022790_01786	ISNCY ssgr IS1202	1901726	1900251	1.476
SL3103	CP022790_00201	61.81% ISMpo10_aa3	72.38% CP022790_02370	Tn3	214736	213348	1.389
SL3103	CP022790_00242	75.15% ISPa42_aa1	100% CP022790_01779	Tn3	253894	256839	2.946
SL3103	CP022790_01770	75.15% ISPa42_aa1	100% CP022790_01779	Tn3	1881875	1884820	2.946
SL3103	CP022790_01779	75.15% ISPa42_aa1	100% CP022790_01770	Tn3	1893833	1890888	2.946
SL3103	CP022790_02370	55.06% ISMpo10_aa3	72.38% CP022790_00201	Tn3	2536915	2535533	1.383
T117	CP022755_02995	61.67% ISMno14_aa1	45.80% CP022755_03327	IS110 ssgr IS1111	3266855	3265842	1.014
T117	CP022755_03327	88.75% ISBcen4_aa1	45.80% CP022755_02995	IS110 ssgr IS1111	3597725	3596706	1.02
T117	CP022755_02747	83.75% ISBusp4_aa1	No hit	IS1182	3000041	2998596	1.446
T117	CP022755_00959	54.76% ISHpa1_aa1	No hit	IS1595 ssgr IS1016	1038141	1038920	780
T117	CP022755_00889	100% ISRso11_aa1	100% CP022755_01764	IS3 ssgr IS150	964444	964977	534
T117	CP022755_00890	99.64% ISRso11_aa2	100% CP022755_01765	IS3 ssgr IS150	964974	96581	837
T117	CP022755_01764	100% ISRso11_aa1	100% CP022755_00889	IS3 ssgr IS150	1900872	1901405	534
T117	CP022755_01765	99.64% ISRso11_aa2	100% CP022755_00890	IS3 ssgr IS150	1901402	1902238	837
T117	CP022755_02517	88.78% IS222_aa2	54.83% CP022755_01168	IS3 ssgr IS3	2767155	2766484	672
T117	CP022755_02518	88.23% IS222_aa1	60.91% CP022755_02563	IS3 ssgr IS3	2767646	2767338	309
T117	CP022755_00957	89.74% ISAisp2_aa2	97.93% CP022755_01168	IS3 ssgr IS51	1035610	1034900	711
T117	CP022755_01167	81.52% ISAisp2_aa1	97.82% CP022755_02563	IS3 ssgr IS51	1251560	1251838	279
T117	CP022755_01168	89.34% ISAisp2_aa2	95.71% CP022755_02530	IS3 ssgr IS51	1251835	1252710	876
T117	CP022755_02529	80.43% ISAisp2_aa1	96.73% CP022755_01167	IS3 ssgr IS51	2778412	2778690	279
T117	CP022755_02530	86.38% ISAisp2_aa2	95.71% CP022755_01168	IS3 ssgr IS51	2778687	2779478	792
T117	CP022755_02563	81.52% ISAisp2_aa1	97.82% CP022755_01167	IS3 ssgr IS51	2814022	2814300	279
T117	CP022755_02564	88.88% ISAisp2_aa2	98.61% CP022755_01168	IS3 ssgr IS51	2814297	2814743	447
T117	CP022755_00306	61.68% ISCro3_aa1	100% CP022755_02611	IS4	340963	342291	1.329
T117	CP022755_00376	61.68% ISCro3_aa1	100% CP022755_02611	IS4	421575	420247	1.329

T117	CP022755_00494	61.68% ISCro3_aa1	100% CP022755_02611	IS4	538101	536773	1.329
T117	CP022755_00613	61.44% ISCro3_aa1	100% CP022755_02433	IS4	661244	662572	1.329
T117	CP022755_01017	61.68% ISCro3_aa1	100% CP022755_02611	IS4	1099240	1097912	1.329
T117	CP022755_01230	61.68% ISCro3_aa1	100% CP022755_03421	IS4	1330036	1328708	1.329
T117	CP022755_01325	61.68% ISCro3_aa1	100% CP022755_02611	IS4	1424048	1425376	1.329
T117	CP022755_01380	61.68% ISCro3_aa1	100% CP022755_03421	IS4	1480524	1481852	1.329
T117	CP022755_01952	61.68% ISCro3_aa1	100% CP022755_03421	IS4	2161993	2160665	1.329
T117	CP022755_02433	61.44% ISCro3_aa1	100% CP022755_00613	IS4	2669955	2668627	1.329
T117	CP022755_02611	61.68% ISCro3_aa1	100% CP022755_01325	IS4	2864699	2866027	1.329
T117	CP022755_03421	61.68% ISCro3_aa1	100% CP022755_01952	IS4	3707953	3706625	1.329
T117	CP022755_00677	78.03% ISAzo5_aa1	100% CP022755_03347	IS4 ssgr IS50	737547	738872	1.326
T117	CP022755_01205	78.03% ISAzo5_aa1	100% CP022755_03347	IS4 ssgr IS50	1298573	1299898	1.326
T117	CP022755_03347	78.03% ISAzo5_aa1	100% CP022755_01205	IS4 ssgr IS50	3622566	3621241	1.326
T117	CP022755_00014	40.42% ISGur11_aa1	44.96% CP022755_02729	IS481	17636	19603	1.968
T117	CP022755_01186	97.44% ISRso1_aa1	98.17% CP022755_03346	IS5	1272211	1271387	825
T117	CP022755_01675	97.81% ISRso1_aa1	100% CP022755_03346	IS5	1799277	1798453	825
T117	CP022755_03346	97.81% ISRso1_aa1	100% CP022755_01675	IS5	3621096	3620272	825
T117	CP022755_00271	99.37% IS1405_aa1	99.68% CP022755_03151	IS5 ssgr IS5	289681	288716	966
T117	CP022755_00311	98.75% IS1405_aa1	99.68% CP022755_03151	IS5 ssgr IS5	346831	347796	966
T117	CP022755_01138	99.06% IS1405_aa1	100% CP022755_03151	IS5 ssgr IS5	1211490	1210525	966
T117	CP022755_01189	99.06% IS1405_aa1	100% CP022755_03151	IS5 ssgr IS5	1274836	1273871	966
T117	CP022755_01190	94.51% IS1021_aa1	100% CP022755_03200	IS5 ssgr IS5	1274994	1275980	987
T117	CP022755_01302	99.06% IS1405_aa1	100% CP022755_03151	IS5 ssgr IS5	1399685	1400650	966
T117	CP022755_02316	99.06% IS1405_aa1	100% CP022755_03151	IS5 ssgr IS5	2541768	2542733	966
T117	CP022755_02726	94.38% IS1021_aa1	95.14% CP022755_03200	IS5 ssgr IS5	2979565	2978756	810
T117	CP022755_03151	99.06% IS1405_aa1	100% CP022755_02316	IS5 ssgr IS5	3412800	3413765	966
T117	CP022755_03200	94.51% IS1021_aa1	100% CP022755_01190	IS5 ssgr IS5	3465054	3464068	987
T117	CP022755_00900	47.5% ISTha3_aa2	51.50% CP022755_03398	IS91	976799	975873	927
T117	CP022755_03398	43.33% ISMno24_aa2	52.12% CP022755_00900	IS91	3685404	3686393	990
T117	CP022755_00073	38.97% ISKpn25_aa1	No hit	ISL3	91174	92619	1.446
T117	CP022755_01826	62.5% ISKpn21_aa1	No hit	ISNCY ssgr IS1202	1965275	1965676	402
T117	CP022755_00226	54.71% ISMpo10_aa3	73.09% CP022755_01203	Tn3	253417	252029	1.389
T117	CP022755_01144	54.43% ISMpo10_aa3	97.39% CP022755_01203	Tn3	1227688	1229070	1.383
T117	CP022755_01203	55.06% ISMpo10_aa3	97.39% CP022755_01144	Tn3	1296589	1297971	1.383

SL2330	CP022794_01555	95.76% ISBma3_aa1	44.82% CP022794_03215	IS110	1703000	1701795	1.206
SL2330	CP022794_03215	88.75% ISBcen4_aa1	No hit	IS110 ssgr IS1111	3526309	3525290	1.02
SL2330	CP022794_00277	49.48% ISHvo5_aa1	100% CP022794_02441	IS1595 ssgr ISH4	310436	311224	789
SL2330	CP022794_02441	49.48% ISHvo5_aa1	100% CP022794_00277	IS1595 ssgr ISH4	2714617	2713829	789
SL2330	CP022794_00334	100% ISRso11_aa1	No hit	IS3 ssgr IS150	383817	38435	534
SL2330	CP022794_00335	99.64% ISRso11_aa2	52% CP022794_03142	IS3 ssgr IS150	384347	385183	837
SL2330	CP022794_01102	88.23% IS222_aa1	100% CP022794_02516	IS3 ssgr IS3	1196813	1197121	309
SL2330	CP022794_01103	89.23% IS222_aa2	97.75% CP022794_02517	IS3 ssgr IS3	1197304	1197975	672
SL2330	CP022794_02516	88.23% IS222_aa1	100% CP022794_01102	IS3 ssgr IS3	2816540	2816848	309
SL2330	CP022794_02517	89.23% IS222_aa2	97.75% CP022794_01103	IS3 ssgr IS3	2817031	2817702	672
SL2330	CP022794_02714	85.71% ISAtu5_aa3	79.51% CP022794_03148	IS3 ssgr IS407	3011601	3011990	390
SL2330	CP022794_03148	100% ISRso14_aa1	79.51% CP022794_02714	IS3 ssgr IS407	3459468	3459731	264
SL2330	CP022794_03149	98.90% ISRso14_aa2	41.07% CP022794_00335	IS3 ssgr IS407	3460037	3460585	549
SL2330	CP022794_01089	86.84% ISAisp2_aa2	96.56% CP022794_03142	IS3 ssgr IS51	1184905	1183970	936
SL2330	CP022794_01090	80.43% ISAisp2_aa1	96.73% CP022794_03141	IS3 ssgr IS51	1185180	1184902	279
SL2330	CP022794_02486	89.34% ISAisp2_aa2	100% CP022794_03142	IS3 ssgr IS51	2770532	2769657	876
SL2330	CP022794_02487	81.52% ISAisp2_aa1	100% CP022794_03141	IS3 ssgr IS51	2770807	2770529	279
SL2330	CP022794_03141	81.52% ISAisp2_aa1	100% CP022794_02487	IS3 ssgr IS51	3453408	3453686	279
SL2330	CP022794_03142	89.34% ISAisp2_aa2	100% CP022794_02486	IS3 ssgr IS51	3453683	3454558	876
SL2330	CP022794_00485	61.68% ISCro3_aa1	100% CP022794_03065	IS4	53801	539338	1.329
SL2330	CP022794_00524	61.68% ISCro3_aa1	100% CP022794_03065	IS4	579001	577673	1.329
SL2330	CP022794_01185	61.91% ISCro3_aa1	99.77% CP022794_03065	IS4	1305039	1303711	1.329
SL2330	CP022794_01207	61.68% ISCro3_aa1	100% CP022794_03065	IS4	1330023	1328695	1.329
SL2330	CP022794_01684	61.68% ISCro3_aa1	100% CP022794_03065	IS4	1843522	1842194	1.329
SL2330	CP022794_02320	61.68% ISCro3_aa1	100% CP022794_03065	IS4	2587852	2589180	1.329
SL2330	CP022794_02596	61.68% ISCro3_aa1	100% CP022794_01185	IS4	2897194	2895866	1.329
SL2330	CP022794_03065	61.68% ISCro3_aa1	100% CP022794_02320	IS4	3365234	3366562	1.329
SL2330	CP022794_03313	61.68% ISCro3_aa1	99.77% CP022794_03065	IS4	3641127	3642455	1.329
SL2330	CP022794_00017	39.67% ISGur11_aa1	No hit	IS481	25644	27611	1.968
SL2330	CP022794_01212	97.81% ISRso1_aa1	100% CP022794_03143	IS5	1337877	1337053	825
SL2330	CP022794_01550	99.27% ISRso1_aa1	98.54% CP022794_03143	IS5	1696661	1695837	825
SL2330	CP022794_01745	97.81% ISRso1_aa1	100% CP022794_03143	IS5	1915071	1915895	825
SL2330	CP022794_02455	97.44% ISRso1_aa1	98.17% CP022794_03143	IS5	2736130	2736954	825
SL2330	CP022794_02493	97.81% ISRso1_aa1	100% CP022794_03143	IS5	2781609	2780785	825
SL2330	CP022794_02494	98.67% ISRso1_aa1	99.11% CP022794_03143	IS5	2781951	2782631	681

SL2330	CP022794_02505	97.81% ISRso1_aa1	100% CP022794_03143	IS5	2804282	2805106	825
SL2330	CP022794_02507	95.91% ISRso1_aa1	96.42% CP022794_03143	IS5	2806870	2806277	594
SL2330	CP022794_03143	97.81% ISRso1_aa1	100% CP022794_02505	IS5	3454706	3455530	825
SL2330	CP022794_00287	86.06% IS1421_aa1	100% CP022794_02813	IS5 ssgr IS427	322069	322476	408
SL2330	CP022794_00985	100% IS1421_aa1	83.96% CP022794_02813	IS5 ssgr IS427	1068075	1068479	405
SL2330	CP022794_01101	86.06% IS1421_aa1	100% CP022794_02813	IS5 ssgr IS427	1195967	1196374	408
SL2330	CP022794_02805	86.06% IS1421_aa1	100% CP022794_02813	IS5 ssgr IS427	3105101	3104694	408
SL2330	CP022794_02813	86.06% IS1421_aa1	100% CP022794_02805	IS5 ssgr IS427	3114598	3115005	408
SL2330	CP022794_00004	99.37% IS1405_aa1	100% CP022794_00300	IS5 ssgr IS5	6075	704	966
SL2330	CP022794_00272	85.71% ISAau3_aa1	80.28% CP022794_00300	IS5 ssgr IS5	304237	30453	294
SL2330	CP022794_00300	99.37% IS1405_aa1	100% CP022794_00004	IS5 ssgr IS5	341785	34275	966
SL2330	CP022794_01696	67.18% ISCARN39_aa2	No hit	IS630	1858096	1858296	201
SL2330	CP022794_02735	100% ISRso17_aa1	100% CP022794_03144	IS701	3031355	3032686	1.332
SL2330	CP022794_03144	100% ISRso17_aa1	100% CP022794_02735	IS701	3455612	3456943	1.332
SL2330	CP022794_00541	47.48% ISShvi3_aa1	52.67% CP022794_01565	IS91	595659	596705	1.047
SL2330	CP022794_00890	47.5% ISTha3_aa2	51.50% CP022794_03286	IS91	970958	970032	927
SL2330	CP022794_03286	43.33% ISMno24_aa2	52.12% CP022794_00890	IS91	3613206	3614195	990
SL2330	CP022794_00007	50.25% ISKpn25_aa1	No hit	ISL3	776	10114	2.355
SL2330	CP022794_00009	65.68% ISKpn25_aa2	No hit	ISL3	11955	13256	1.302
SL2330	CP022794_00010	49.95% ISKpn25_aa3	No hit	ISL3	13265	16225	2.961
SL2330	CP022794_00061	38.97% ISKpn25_aa1	No hit	ISL3	77576	79021	1.446
SL2330	CP022794_01809	64.60% ISKpn21_aa1	77.61% CP022794_01811	ISNCY ssgr IS1202	2042806	2042405	402
SL2330	CP022794_01810	74.02% ISKpn21_aa1	No hit	ISNCY ssgr IS1202	2043901	2042957	945
SL2330	CP022794_01811	62.5% ISKpn21_aa1	77.61% CP022794_01809	ISNCY ssgr IS1202	2044434	2043901	534
SL2330	CP022794_00213	54.08% ISMpo10_aa3	97.40% CP022794_00283	Tn3	243001	241613	1.389
SL2330	CP022794_00283	61.81% ISMpo10_aa3	97.40% CP022794_00213	Tn3	317607	316219	1.389
SL2330	CP022794_01559	77.00% ISSba14_aa1	46.25% CP022794_02501	Tn3	1707316	1707885	570
SL2330	CP022794_02439	51.83% ISMpo10_aa3	98.69% CP022794_02501	Tn3	2712404	2711022	1.383
SL2330	CP022794_02501	54.43% ISMpo10_aa3	98.69% CP022794_02439	Tn3	2800042	2801424	1.383
SL3755	CP022782_01563	95.76% ISBma3_aa1	44.82% CP022782_03363	IS110	1679531	1678326	1.206
SL3755	CP022782_03363	88.75% ISBcen4_aa1	No hit	IS110 ssgr IS1111	3640230	3639211	1.02
SL3755	CP022782_02564	49.48% ISHvo5_aa1	44.65% CP022782_02624	IS1595 ssgr ISH4	2808012	2807224	789
SL3755	CP022782_00320	100% ISRso11_aa1	No hit	IS3 ssgr IS150	352383	352916	534
SL3755	CP022782_00321	99.64% ISRso11_aa2	52% CP022782_02611	IS3 ssgr IS150	352913	353749	837

SL3755	CP022782_01104	88.23% IS222_aa1	100% CP022782_02641	IS3 ssgr IS3	1174096	1174404	309
SL3755	CP022782_01105	89.23% IS222_aa2	97.75% CP022782_02642	IS3 ssgr IS3	1174587	1175258	672
SL3755	CP022782_02641	88.23% IS222_aa1	100% CP022782_01104	IS3 ssgr IS3	2913810	2914118	309
SL3755	CP022782_02642	89.23% IS222_aa2	97.75% CP022782_01105	IS3 ssgr IS3	2914301	2914972	672
SL3755	CP022782_02839	85.71% ISAtu5_aa3	79.51% CP022782_03295	IS3 ssgr IS407	3108920	3109309	390
SL3755	CP022782_03295	100% ISRso14_aa1	79.51% CP022782_02839	IS3 ssgr IS407	3573390	3573653	264
SL3755	CP022782_03296	98.90% ISRso14_aa2	41.07% CP022782_00321	IS3 ssgr IS407	3573959	3574507	549
SL3755	CP022782_01092	86.84% ISAisp2_aa2	96.56% CP022782_02611	IS3 ssgr IS51	1163055	1162120	936
SL3755	CP022782_01093	80.43% ISAisp2_aa1	96.73% CP022782_02612	IS3 ssgr IS51	1163330	1163052	279
SL3755	CP022782_02611	89.34% ISAisp2_aa2	96.56% CP022782_01092	IS3 ssgr IS51	2867808	2866933	876
SL3755	CP022782_02612	81.52% ISAisp2_aa1	96.73% CP022782_01093	IS3 ssgr IS51	2868083	2867805	279
SL3755	CP022782_00471	61.68% ISCro3_aa1	100% CP022782_03214	IS4	506573	507901	1.329
SL3755	CP022782_00513	61.68% ISCro3_aa1	100% CP022782_03214	IS4	547561	546233	1.329
SL3755	CP022782_01188	61.91% ISCro3_aa1	99.77% CP022782_03214	IS4	1282318	1280990	1.329
SL3755	CP022782_01210	61.68% ISCro3_aa1	100% CP022782_03214	IS4	1307302	1305974	1.329
SL3755	CP022782_01692	61.68% ISCro3_aa1	100% CP022782_03214	IS4	1820050	1818722	1.329
SL3755	CP022782_02382	61.68% ISCro3_aa1	100% CP022782_03214	IS4	2606857	2608185	1.329
SL3755	CP022782_02721	61.68% ISCro3_aa1	100% CP022782_01188	IS4	2994484	2993156	1.329
SL3755	CP022782_03214	61.68% ISCro3_aa1	100% CP022782_02382	IS4	3480401	3481729	1.329
SL3755	CP022782_03463	61.68% ISCro3_aa1	99.77% CP022782_03214	IS4	3755037	3756365	1.329
SL3755	CP022782_00259	97.29% ISRso1_aa1	98.79% CP022782_02632	IS5	264616	264888	273
SL3755	CP022782_01215	97.81% ISRso1_aa1	100% CP022782_03290	IS5	1315156	1314332	825
SL3755	CP022782_01558	99.27% ISRso1_aa1	98.54% CP022782_03290	IS5	1673193	1672369	825
SL3755	CP022782_01753	97.81% ISRso1_aa1	100% CP022782_03290	IS5	1891555	1892379	825
SL3755	CP022782_02580	97.44% ISRso1_aa1	98.17% CP022782_03290	IS5	2833407	2834231	825
SL3755	CP022782_02618	97.44% ISRso1_aa1	99.63% CP022782_03290	IS5	2878880	2878056	825
SL3755	CP022782_02629	98.67% ISRso1_aa1	99.11% CP022782_03290	IS5	2901210	2900530	681
SL3755	CP022782_02630	97.81% ISRso1_aa1	100% CP022782_03290	IS5	2901552	2902376	825
SL3755	CP022782_02632	95.91% ISRso1_aa1	96.42% CP022782_03290	IS5	2904140	2903547	594
SL3755	CP022782_03290	97.81% ISRso1_aa1	100% CP022782_02630	IS5	3568628	3569452	825
SL3755	CP022782_00271	99.06% IS1405_aa1	100% CP022782_02506	IS5 ssgr IS5	287304	288269	966
SL3755	CP022782_00286	99.37% IS1405_aa1	99.68% CP022782_02506	IS5 ssgr IS5	310351	311316	966
SL3755	CP022782_02506	99.06% IS1405_aa1	100% CP022782_00271	IS5 ssgr IS5	2745636	2746601	966
SL3755	CP022782_01704	67.18% ISCARN39_aa2	No hit	IS630	1834624	1834824	201
SL3755	CP022782_02860	100% ISRso17_aa1	100% CP022782_03291	IS701	3128674	3130005	1.332

SL3755	CP022782_03291	100% ISRso17_aa1	100% CP022782_02860	IS701	3570882	3569551	1.332
SL3755	CP022782_00530	47.48% ISShvi3_aa1	52.67% CP022782_01573	IS91	564219	565265	1.047
SL3755	CP022782_00882	47.5% ISTha3_aa2	51.50% CP022782_03436	IS91	939519	938593	927
SL3755	CP022782_02519	45.61% ISTha3_aa2	49% CP022782_00882	IS91	2756804	2755785	1.02
SL3755	CP022782_02521	45.76% ISWz1_aa1	51.39% CP022782_00882	IS91	2758947	2757700	1.248
SL3755	CP022782_03436	43.33% ISMno24_aa2	52.12% CP022782_00882	IS91	3727116	3728105	990
SL3755	CP022782_00007	36.11% ISSm4_aa4	No hit	ISL3	13464	1018	3.285
SL3755	CP022782_00009	47.46% ISKpn25_aa1	No hit	ISL3	1552	14747	774
SL3755	CP022782_00010	44.19% ISSm4_aa1	40.14% CP022782_00043	ISL3	16366	15581	786
SL3755	CP022782_00043	38.97% ISKpn25_aa1	40.14% CP022782_00010	ISL3	51062	52507	1.446
SL3755	CP022782_01820	64.60% ISKpn21_aa1	77.61% CP022782_01822	ISNCY ssgr IS1202	2019318	2018917	402
SL3755	CP022782_01821	74.02% ISKpn21_aa1	No hit	ISNCY ssgr IS1202	2020413	2019469	945
SL3755	CP022782_01822	62.5% ISKpn21_aa1	77.61% CP022782_01820	ISNCY ssgr IS1202	2020946	2020413	534
SL3755	CP022782_00197	61.81% ISMpo10_aa3	98.48% CP022782_00269	Tn3	213203	211815	1.389
SL3755	CP022782_00269	61.81% ISMpo10_aa3	98.48% CP022782_00197	Tn3	285863	284475	1.389
SL3755	CP022782_01075	61.87% ISPa38_aa1	55.17% CP022782_01567	Tn3	1151776	1152366	591
SL3755	CP022782_01567	77.00% ISSba14_aa1	55.17% CP022782_01075	Tn3	1683847	1684416	570
SL3755	CP022782_02500	54.43% ISMpo10_aa3	98.47% CP022782_02622	Tn3	2729475	2728093	1.383
SL3755	CP022782_02562	54.16% ISMpo10_aa3	100% CP022782_02622	Tn3	2805799	2805257	543
SL3755	CP022782_02622	54.43% ISMpo10_aa3	98.47% CP022782_02500	Tn3	2883119	2881737	1.383
T25	CP023014_01628	95.5% ISBma3_aa1	99.75% CP023014_01633	IS110	1677380	1675938	1.443
T25	CP023014_01633	95.76% ISBma3_aa1	99.75% CP023014_01628	IS110	1683417	1682212	1.206
T25	CP023014_03513	88.75% ISBcen4_aa1	No hit	IS110 ssgr IS1111	3567445	3566426	1.02
T25	CP023014_00272	49.48% ISHvo5_aa1	100% CP023014_02681	IS1595 ssgr ISH4	285226	286014	789
T25	CP023014_02681	49.48% ISHvo5_aa1	100% CP023014_00272	IS1595 ssgr ISH4	2739163	2738375	789
T25	CP023014_00331	100% ISRso11_aa1	No hit	IS3 ssgr IS150	357728	358261	534
T25	CP023014_00332	99.64% ISRso11_aa2	52% CP023014_02732	IS3 ssgr IS150	358258	359094	837
T25	CP023014_01139	88.23% IS222_aa1	100% CP023014_02768	IS3 ssgr IS3	1170131	1170439	309
T25	CP023014_01140	89.23% IS222_aa2	97.75% CP023014_02769	IS3 ssgr IS3	1170622	1171293	672
T25	CP023014_02768	88.23% IS222_aa1	100% CP023014_01139	IS3 ssgr IS3	2842240	2842548	309
T25	CP023014_02769	89.23% IS222_aa2	97.75% CP023014_01140	IS3 ssgr IS3	2842731	2843402	672
T25	CP023014_02970	85.71% ISAtu5_aa3	79.51% CP023014_03443	IS3 ssgr IS407	3037310	3037699	390
T25	CP023014_03443	100% ISRso14_aa1	79.51% CP023014_02970	IS3 ssgr IS407	3500624	3500887	264
T25	CP023014_03444	99.45% ISRso14_aa2	39.87% CP023014_00332	IS3 ssgr IS407	3501193	3501741	549

T25	CP023014_01127	86.84% ISAisp2_aa2	96.56% CP023014_02732	IS3 ssgr IS51	1159090	1158155	936
T25	CP023014_01128	80.43% ISAisp2_aa1	96.73% CP023014_02733	IS3 ssgr IS51	1159365	1159087	279
T25	CP023014_02732	89.34% ISAisp2_aa2	96.56% CP023014_01127	IS3 ssgr IS51	2796249	2795374	876
T25	CP023014_02733	81.52% ISAisp2_aa1	96.73% CP023014_01128	IS3 ssgr IS51	2796524	2796246	279
T25	CP023014_00488	61.68% ISCro3_aa1	100% CP023014_03358	IS4	511898	513226	1.329
T25	CP023014_00530	61.68% ISCro3_aa1	100% CP023014_03358	IS4	552884	551556	1.329
T25	CP023014_01230	61.91% ISCro3_aa1	99.77% CP023014_03358	IS4	1278336	1277008	1.329
T25	CP023014_01254	61.68% ISCro3_aa1	100% CP023014_03358	IS4	1303316	1301988	1.329
T25	CP023014_01775	61.68% ISCro3_aa1	100% CP023014_03358	IS4	1823899	1822571	1.329
T25	CP023014_02550	63.32% ISCro3_aa1	94.73% CP023014_01775	IS4	2612442	2613779	1.338
T25	CP023014_02847	61.68% ISCro3_aa1	100% CP023014_01230	IS4	2922897	2921569	1.329
T25	CP023014_03358	61.68% ISCro3_aa1	100% CP023014_01254	IS4	3409035	3410363	1.329
T25	CP023014_03620	61.68% ISCro3_aa1	99.77% CP023014_03358	IS4	3682215	3683543	1.329
T25	CP023014_01259	97.81% ISRso1_aa1	100% CP023014_03439	IS5	1311169	1310345	825
T25	CP023014_01622	98.87% ISRso1_aa1	99.46% CP023014_03439	IS5	1669605	1669042	564
T25	CP023014_01844	97.81% ISRso1_aa1	100% CP023014_03439	IS5	1895375	1896199	825
T25	CP023014_02696	97.44% ISRso1_aa1	98.17% CP023014_03439	IS5	2760673	2761497	825
T25	CP023014_02739	97.44% ISRso1_aa1	99.63% CP023014_03439	IS5	2807316	2806492	825
T25	CP023014_02756	98.67% ISRso1_aa1	99.11% CP023014_03439	IS5	2829644	2828964	681
T25	CP023014_02757	97.81% ISRso1_aa1	100% CP023014_03439	IS5	2829985	2830809	825
T25	CP023014_02759	95.91% ISRso1_aa1	96.42% CP023014_03439	IS5	2832573	2831980	594
T25	CP023014_03439	97.81% ISRso1_aa1	100% CP023014_02757	IS5	3497337	3498161	825
T25	CP023014_01630	86.44% IS1421_aa1	95.04% CP023014_03072	IS5 ssgr IS427	1679703	1679335	369
T25	CP023014_03063	86.06% IS1421_aa1	99.12% CP023014_03072	IS5 ssgr IS427	3129297	3128890	408
T25	CP023014_03072	87.05% IS1421_aa3	95.04% CP023014_01630	IS5 ssgr IS427	3138789	3139376	588
T25	CP023014_00268	85.71% ISAau3_aa1	80.28% CP023014_02718	IS5 ssgr IS5	279028	279321	294
T25	CP023014_00295	98.91% IS1405_aa1	100% CP023014_02718	IS5 ssgr IS5	315696	316292	597
T25	CP023014_00296	100% IS1405_aa1	100% CP023014_02718	IS5 ssgr IS5	31625	31666	411
T25	CP023014_01627	99.37% IS1405_aa1	100% CP023014_02718	IS5 ssgr IS5	1675075	1676040	966
T25	CP023014_02718	99.37% IS1405_aa1	100% CP023014_01627	IS5 ssgr IS5	2783497	2782532	966
T25	CP023014_01788	67.18% ISCARN39_aa2	No hit	IS630	1838469	1838669	201
T25	CP023014_00918	51.30% ISMno23_aa1	55.04% CP023014_03593	IS91	944523	943858	666
T25	CP023014_03593	43.33% ISMno24_aa2	55.93% CP023014_00918	IS91	3654314	3655303	990
T25	CP023014_00009	36.11% ISSm4_aa4	No hit	ISL3	13472	10188	3.285
T25	CP023014_00011	44.54% ISKpn25_aa1	38.86% CP023014_00047	ISL3	16374	14755	1.62

T25	CP023014_00047	38.97% ISKpn25_aa1	38.62% CP023014_00011	ISL3	51675	5312	1.446
T25	CP023014_01938	66.66% ISRel10_aa1	83.33% CP023014_01940	ISNCY ssgr IS1202	2023105	2022857	249
T25	CP023014_01939	74.02% ISKpn21_aa1	No hit	ISNCY ssgr IS1202	2024200	2023256	945
T25	CP023014_01940	62.5% ISKpn21_aa1	83.33% CP023014_01938	ISNCY ssgr IS1202	2024733	2024200	534
T25	CP023014_00205	54.08% ISMpo10_aa3	97.40% CP023014_00278	Tn3	217177	215789	1.389
T25	CP023014_00278	61.81% ISMpo10_aa3	97.40% CP023014_00205	Tn3	292397	291009	1.389
T25	CP023014_01637	77.00% ISSba14_aa1	46.25% CP023014_02743	Tn3	1687729	1688298	570
T25	CP023014_02679	54.43% ISMpo10_aa3	98.91% CP023014_02743	Tn3	2736950	2735568	1.383
T25	CP023014_02743	54.43% ISMpo10_aa3	98.91% CP023014_02679	Tn3	2811556	2810174	1.383

T78	CP022765_03216	61.67% ISMno14_aa1	45.80% CP022765_03594	IS110 ssgr IS1111	3435899	3434886	1.014
T78	CP022765_03594	88.75% ISBcen4_aa1	45.80% CP022765_03216	IS110 ssgr IS1111	3809165	3808146	1.02
T78	CP022765_03009	83.75% ISBusp4_aa1	No hit	IS1182	3220243	3218798	1.446
T78	CP022765_01027	54.76% ISHpa1_aa1	No hit	IS1595 ssgr IS1016	1086163	1086942	780
T78	CP022765_00258	49.48% ISHvo5_aa1	44.02% CP022765_02747	IS1595 ssgr ISH4	279269	280057	789
T78	CP022765_00957	100% ISRso11_aa1	100% CP022765_01998	IS3 ssgr IS150	1012466	1012999	534
T78	CP022765_00958	99.64% ISRso11_aa2	100% CP022765_01997	IS3 ssgr IS150	1012996	1013832	837
T78	CP022765_01997	99.64% ISRso11_aa2	100% CP022765_00958	IS3 ssgr IS150	2154047	2153211	837
T78	CP022765_01998	100% ISRso11_aa1	100% CP022765_00957	IS3 ssgr IS150	2154577	2154044	534
T78	CP022765_01178	88.23% IS222_aa1	60.91% CP022765_03004	IS3 ssgr IS3	1244315	1244623	309
T78	CP022765_01179	88.78% IS222_aa2	55.76% CP022765_03005	IS3 ssgr IS3	1244806	1245477	672
T78	CP022765_01025	89.74% ISAisp2_aa2	99.48% CP022765_03005	IS3 ssgr IS51	1083632	1082922	711
T78	CP022765_01132	88.88% ISAisp2_aa2	100% CP022765_03005	IS3 ssgr IS51	1197664	1197218	447
T78	CP022765_01133	81.52% ISAisp2_aa1	100% CP022765_03004	IS3 ssgr IS51	1197939	1197661	279
T78	CP022765_01166	86.38% ISAisp2_aa2	94.94% CP022765_03005	IS3 ssgr IS51	1233274	1232483	792
T78	CP022765_01167	80.43% ISAisp2_aa1	96.73% CP022765_02722	IS3 ssgr IS51	1233549	1233271	279
T78	CP022765_02721	89.34% ISAisp2_aa2	98.28% CP022765_03005	IS3 ssgr IS51	2925299	2924424	876
T78	CP022765_02722	81.52% ISAisp2_aa1	97.82% CP022765_03004	IS3 ssgr IS51	2925574	2925296	279
T78	CP022765_03004	81.52% ISAisp2_aa1	100% CP022765_01133	IS3 ssgr IS51	3213099	3213377	279
T78	CP022765_03005	89.34% ISAisp2_aa2	98.28% CP022765_02721	IS3 ssgr IS51	3213374	3214249	876
T78	CP022765_00271	61.68% ISCro3_aa1	100% CP022765_00513	IS4	298991	300319	1.329
T78	CP022765_00340	61.68% ISCro3_aa1	100% CP022765_00513	IS4	379607	378279	1.329
T78	CP022765_00513	61.68% ISCro3_aa1	100% CP022765_00340	IS4	537036	535708	1.329
T78	CP022765_00680	61.44% ISCro3_aa1	100% CP022765_01263	IS4	709273	710601	1.329
T78	CP022765_01085	61.68% ISCro3_aa1	100% CP022765_02485	IS4	1147261	1145933	1.329

T78	CP022765_01263	61.44% ISCro3_aa1	100% CP022765_00680	IS4	1342005	1343333	1.329
T78	CP022765_01800	61.68% ISCro3_aa1	100% CP022765_03688	IS4	1892160	1893488	1.329
T78	CP022765_02431	61.68% ISCro3_aa1	100% CP022765_03688	IS4	2618614	2617286	1.329
T78	CP022765_02485	61.68% ISCro3_aa1	100% CP022765_01085	IS4	2675071	2673743	1.329
T78	CP022765_02580	61.68% ISCro3_aa1	100% CP022765_03688	IS4	2769071	2770399	1.329
T78	CP022765_02872	61.68% ISCro3_aa1	100% CP022765_03688	IS4	3077891	3079219	1.329
T78	CP022765_03688	61.68% ISCro3_aa1	100% CP022765_02872	IS4	3917914	3916586	1.329
T78	CP022765_00576	78.03% ISAzo5_aa1	100% CP022765_02683	IS4 ssgr IS50	601287	599962	1.326
T78	CP022765_00852	78.03% ISAzo5_aa1	100% CP022765_02683	IS4 ssgr IS50	908568	909893	1.326
T78	CP022765_02683	78.03% ISAzo5_aa1	100% CP022765_00852	IS4 ssgr IS50	2878563	2877238	1.326
T78	CP022765_02087	97.81% ISRso1_aa1	100% CP022765_03613	IS5	2256197	2257021	825
T78	CP022765_02703	97.44% ISRso1_aa1	98.17% CP022765_03613	IS5	2904925	2905749	825
T78	CP022765_03613	97.81% ISRso1_aa1	100% CP022765_02087	IS5	3832542	3831718	825
T78	CP022765_00254	85.93% ISAau3_aa1	80% CP022765_03415	IS5 ssgr IS5	273094	273363	270
T78	CP022765_00276	99.06% IS1405_aa1	100% CP022765_03415	IS5 ssgr IS5	304859	305824	966
T78	CP022765_00342	99.06% IS1405_aa1	100% CP022765_03415	IS5 ssgr IS5	380732	379767	966
T78	CP022765_00368	94.51% IS1021_aa1	100% CP022765_03465	IS5 ssgr IS5	401585	400599	987
T78	CP022765_01380	99.06% IS1405_aa1	100% CP022765_03415	IS5 ssgr IS5	1470191	1469226	966
T78	CP022765_02324	99.06% IS1405_aa1	100% CP022765_03415	IS5 ssgr IS5	2518793	2517828	966
T78	CP022765_02508	99.06% IS1405_aa1	100% CP022765_03415	IS5 ssgr IS5	2699434	2698469	966
T78	CP022765_02699	94.51% IS1021_aa1	100% CP022765_03465	IS5 ssgr IS5	2902142	2901156	987
T78	CP022765_02700	99.06% IS1405_aa1	100% CP022765_03415	IS5 ssgr IS5	2902300	2903265	966
T78	CP022765_02751	99.06% IS1405_aa1	100% CP022765_03415	IS5 ssgr IS5	2965642	2966607	966
T78	CP022765_03268	99.06% IS1405_aa1	100% CP022765_03415	IS5 ssgr IS5	3484036	3483071	966
T78	CP022765_03415	99.06% IS1405_aa1	100% CP022765_03268	IS5 ssgr IS5	3624607	3625572	966
T78	CP022765_03465	94.51% IS1021_aa1	100% CP022765_02699	IS5 ssgr IS5	3676859	3675873	987
T78	CP022765_01773	100% IS1420_aa1	No hit	IS5 ssgr IS903	1867414	1868370	957
T78	CP022765_00968	47.5% ISTha3_aa2	51.50% CP022765_03665	IS91	1024821	1023895	927
T78	CP022765_03665	43.33% ISMno24_aa2	52.12% CP022765_00968	IS91	3895366	3896355	990
T78	CP022765_00036	38.97% ISKpn25_aa1	48.32% CP022765_00608	ISL3	49801	51246	1.446
T78	CP022765_00608	45.49% ISKpn25_aa1	69.40% CP022765_00609	ISL3	625602	627122	1.521
T78	CP022765_00609	45.06% ISKpn25_aa1	69.40% CP022765_00608	ISL3	6271	628728	1.629
T78	CP022765_00612	44.68% ISSm4_aa3	No hit	ISL3	63232	633501	1.182
T78	CP022765_00613	39.16% ISKpn25_aa3	No hit	ISL3	633501	636485	2.985
T78	CP022765_01931	62.5% ISKpn21_aa1	93.33% CP022765_01932	ISNCY ssgr IS1202	2088553	2088152	402

T78	CP022765_01932	72.27% ISKpn21_aa1	93.33% CP022765_01931	ISNCY ssgr IS1202	2090182	2088707	1.476
T78	CP022765_00189	61.81% ISMpo10_aa3	72.38% CP022765_02685	Tn3	211916	210528	1.389
T78	CP022765_02605	54.43% ISMpo10_aa3	100% CP022765_02745	Tn3	2799117	2797735	1.383
T78	CP022765_02685	55.06% ISMpo10_aa3	97.39% CP022765_02745	Tn3	2880547	2879165	1.383
T78	CP022765_02745	54.43% ISMpo10_aa3	100% CP022765_02605	Tn3	2949445	2948063	1.383
SL3730	CP022784_02996	61.67% ISMno14_aa1	45.80% CP022784_03341	IS110 ssgr IS1111	3230960	3229947	1.014
SL3730	CP022784_03341	88.75% ISBcen4_aa1	45.80% CP022784_02996	IS110 ssgr IS1111	3568251	3567232	1.02
SL3730	CP022784_00955	83.75% ISBusp4_aa1	100% CP022784_02772	IS1182	1029076	1030521	1.446
SL3730	CP022784_02772	83.75% ISBusp4_aa1	100% CP022784_00955	IS1182	2999410	2997965	1.446
SL3730	CP022784_00964	54.76% ISHpa1_aa1	No hit	IS1595 ssgr IS1016	1041065	1041844	780
SL3730	CP022784_00881	100% ISRso11_aa1	100% CP022784_01954	IS3 ssgr IS150	951981	952409	429
SL3730	CP022784_00882	99.64% ISRso11_aa2	100% CP022784_01953	IS3 ssgr IS150	952406	953242	837
SL3730	CP022784_01953	99.64% ISRso11_aa2	100% CP022784_00882	IS3 ssgr IS150	2146490	2145654	837
SL3730	CP022784_01954	100% ISRso11_aa1	100% CP022784_00881	IS3 ssgr IS150	2147020	2146487	534
SL3730	CP022784_01164	88.23% IS222_aa1	60.91% CP022784_01071	IS3 ssgr IS3	1258568	1258876	309
SL3730	CP022784_01165	88.78% IS222_aa2	55.76% CP022784_01070	IS3 ssgr IS3	1259059	1259730	672
SL3730	CP022784_00656	81.52% ISAisp2_aa1	100% CP022784_01071	IS3 ssgr IS51	706843	707121	279
SL3730	CP022784_00657	89.34% ISAisp2_aa2	100% CP022784_01070	IS3 ssgr IS51	707118	707993	876
SL3730	CP022784_00961	81.52% ISAisp2_aa1	100% CP022784_01071	IS3 ssgr IS51	1037865	1038143	279
SL3730	CP022784_00962	89.34% ISAisp2_aa2	100% CP022784_01070	IS3 ssgr IS51	1038140	1039015	876
SL3730	CP022784_01070	89.34% ISAisp2_aa2	100% CP022784_00962	IS3 ssgr IS51	1156414	1155539	876
SL3730	CP022784_01071	81.52% ISAisp2_aa1	100% CP022784_00961	IS3 ssgr IS51	1156689	1156411	279
SL3730	CP022784_01152	86.38% ISAisp2_aa2	94.94% CP022784_01070	IS3 ssgr IS51	1247527	1246736	792
SL3730	CP022784_01153	80.43% ISAisp2_aa1	96.73% CP022784_03088	IS3 ssgr IS51	1247802	1247524	279
SL3730	CP022784_03087	89.34% ISAisp2_aa2	98.28% CP022784_01070	IS3 ssgr IS51	3316330	3315455	876
SL3730	CP022784_03088	81.52% ISAisp2_aa1	97.82% CP022784_01071	IS3 ssgr IS51	3316605	3316327	279
SL3730	CP022784_00278	61.68% ISCro3_aa1	100% CP022784_02669	IS4	299432	30076	1.329
SL3730	CP022784_00348	61.68% ISCro3_aa1	100% CP022784_02669	IS4	382099	380771	1.329
SL3730	CP022784_00468	61.68% ISCro3_aa1	100% CP022784_02669	IS4	498617	497289	1.329
SL3730	CP022784_00587	61.44% ISCro3_aa1	100% CP022784_01250	IS4	621759	623087	1.329
SL3730	CP022784_01022	61.68% ISCro3_aa1	100% CP022784_02669	IS4	1102164	1100836	1.329
SL3730	CP022784_01250	61.44% ISCro3_aa1	100% CP022784_00587	IS4	1356255	1357583	1.329
SL3730	CP022784_01759	61.68% ISCro3_aa1	100% CP022784_03433	IS4	1884288	1885616	1.329
SL3730	CP022784_02340	61.68% ISCro3_aa1	100% CP022784_03433	IS4	2567377	2566049	1.329

SL3730	CP022784_02396	61.68% ISCro3_aa1	99.77% CP022784_02669	IS4	2623834	2622506	1.329
SL3730	CP022784_02423	61.68% ISCro3_aa1	100% CP022784_02669	IS4	2653004	2651676	1.329
SL3730	CP022784_02494	61.68% ISCro3_aa1	100% CP022784_03433	IS4	2719272	2720600	1.329
SL3730	CP022784_02669	61.68% ISCro3_aa1	100% CP022784_02423	IS4	2897316	2898644	1.329
SL3730	CP022784_03433	61.68% ISCro3_aa1	100% CP022784_02494	IS4	3678770	3677442	1.329
SL3730	CP022784_00264	78.03% ISAzo5_aa1	100% CP022784_00921	IS4 ssgr IS50	281734	283059	1.326
SL3730	CP022784_00651	78.03% ISAzo5_aa1	100% CP022784_00921	IS4 ssgr IS50	698992	700317	1.326
SL3730	CP022784_00921	78.03% ISAzo5_aa1	100% CP022784_00651	IS4 ssgr IS50	992425	99375	1.326
SL3730	CP022784_01371	97.81% ISRso1_aa1	100% CP022784_02044	IS5	1486945	1486121	825
SL3730	CP022784_02044	97.81% ISRso1_aa1	100% CP022784_01371	IS5	2248620	2249444	825
SL3730	CP022784_00238	99.37% IS1405_aa1	99.68% CP022784_03213	IS5 ssgr IS5	246687	245722	966
SL3730	CP022784_00283	99.06% IS1405_aa1	100% CP022784_03213	IS5 ssgr IS5	3053	306265	966
SL3730	CP022784_00670	94.51% IS1021_aa1	100% CP022784_03276	IS5 ssgr IS5	724131	725117	987
SL3730	CP022784_00777	94.51% IS1021_aa1	100% CP022784_03276	IS5 ssgr IS5	849119	848133	987
SL3730	CP022784_01369	99.06% IS1405_aa1	100% CP022784_03213	IS5 ssgr IS5	1484436	1483471	966
SL3730	CP022784_01535	99.06% IS1405_aa1	100% CP022784_03213	IS5 ssgr IS5	1650619	1649654	966
SL3730	CP022784_02419	99.06% IS1405_aa1	100% CP022784_03213	IS5 ssgr IS5	2648196	2647231	966
SL3730	CP022784_02527	99.06% IS1405_aa1	100% CP022784_03213	IS5 ssgr IS5	2764699	2765664	966
SL3730	CP022784_02545	99.06% IS1405_aa1	100% CP022784_03213	IS5 ssgr IS5	2783627	2782662	966
SL3730	CP022784_03084	94.51% IS1021_aa1	100% CP022784_03276	IS5 ssgr IS5	3313266	3312280	987
SL3730	CP022784_03093	99.06% IS1405_aa1	100% CP022784_03213	IS5 ssgr IS5	3322059	3321094	966
SL3730	CP022784_03112	94.51% IS1021_aa1	100% CP022784_03276	IS5 ssgr IS5	3335441	3336427	987
SL3730	CP022784_03159	99.06% IS1405_aa1	100% CP022784_03213	IS5 ssgr IS5	3381746	3382711	966
SL3730	CP022784_03213	99.06% IS1405_aa1	100% CP022784_03159	IS5 ssgr IS5	3442755	3441790	966
SL3730	CP022784_03276	94.51% IS1021_aa1	100% CP022784_03112	IS5 ssgr IS5	3505591	3506577	987
SL3730	CP022784_00009	100% IS1420_aa1	100% CP022784_03172	IS5 ssgr IS903	15894	14938	957
SL3730	CP022784_00650	100% IS1420_aa1	100% CP022784_03172	IS5 ssgr IS903	697059	698015	957
SL3730	CP022784_00775	100% IS1420_aa1	100% CP022784_03172	IS5 ssgr IS903	838623	839579	957
SL3730	CP022784_02776	100% IS1420_aa1	100% CP022784_03172	IS5 ssgr IS903	3002810	3001854	957
SL3730	CP022784_02778	100% IS1420_aa1	100% CP022784_03172	IS5 ssgr IS903	3004483	3005439	957
SL3730	CP022784_02817	100% IS1420_aa1	100% CP022784_03172	IS5 ssgr IS903	3044313	3045269	957
SL3730	CP022784_03172	100% IS1420_aa1	100% CP022784_02817	IS5 ssgr IS903	3393058	3394014	957
SL3730	CP022784_00893	47.5% ISTha3_aa2	51.50% CP022784_03410	IS91	96423	963304	927
SL3730	CP022784_01637	40.65% ISShvi3_aa1	51.94% CP022784_01623	IS91	1760726	1759662	1.065
SL3730	CP022784_03410	43.33% ISMno24_aa2	52.12% CP022784_00893	IS91	3656221	3657210	990

SL3730	CP022784_00039	38.97% ISKpn25_aa1	No hit	ISL3	50955	524	1.446
SL3730	CP022784_01890	62.5% ISKpn21_aa1	No hit	ISNCY ssgr IS1202	2080992	2080591	402
SL3730	CP022784_01891	73.76% ISKpn21_aa1	No hit	ISNCY ssgr IS1202	2082225	2081146	1.08
SL3730	CP022784_00196	61.81% ISMpo10_aa3	73.17% CP022784_02520	Tn3	214346	212958	1.389
SL3730	CP022784_01627	75.40% ISPa40_aa4	54.09% CP022784_00196	Tn3	1746966	1747562	597
SL3730	CP022784_02520	55.06% ISMpo10_aa3	73.17% CP022784_00196	Tn3	2749318	2747936	1.383
12D	IS_e546712d_00634	69.14% IS5708_aa1	55.95% IS_e546712d_01048	IS110 ssgr IS1111	666688	667065	378
12D	IS_e546712d_01048	65.76% ISBj4_aa1	55.95% IS_e546712d_00634	IS110 ssgr IS1111	1087131	1088168	1.038
12D	IS_e546712d_01052	98.46% ISRme4_aa2	No hit	IS21	1093348	1092563	786
12D	IS_e546712d_01053	81.27% IS1600_aa1	No hit	IS21	1094864	1093338	1.527
12D	IS_e546712d_00007	98.31% ISRso7_aa1	99.75% IS_e546712d_03200	IS256	10877	9627	1.251
12D	IS_e546712d_00090	98.07% ISRso7_aa1	100% IS_e546712d_03200	IS256	9381	9256	1.251
12D	IS_e546712d_00254	77.07% ISNGR8_aa1	67.42% IS_e546712d_03200	IS256	265431	266222	792
12D	IS_e546712d_00255	98.07% ISRso7_aa1	100% IS_e546712d_03200	IS256	266161	267411	1.251
12D	IS_e546712d_00256	79.47% ISSpwi2_aa1	80.10% IS_e546712d_03200	IS256	267479	268054	576
12D	IS_e546712d_00726	98.07% ISRso7_aa1	100% IS_e546712d_03200	IS256	754129	752879	1.251
12D	IS_e546712d_01289	98.07% ISRso7_aa1	100% IS_e546712d_03200	IS256	1348016	1346766	1.251
12D	IS_e546712d_03170	98.07% ISRso7_aa1	100% IS_e546712d_03200	IS256	3308740	3309990	1.251
12D	IS_e546712d_03200	98.07% ISRso7_aa1	100% IS_e546712d_03170	IS256	3353321	3352071	1.251
12D	IS_e546712d_00630	92.72% ISCte2_aa1	No hit	IS3 ssgr IS3	662905	663237	333
12D	IS_e546712d_00631	92.04% ISCte2_aa2	57.02% IS_e546712d_00747	IS3 ssgr IS3	663234	664103	870
12D	IS_e546712d_01984	79.69% ISPsy11_aa1	100% IS_e546712d_03248	IS3 ssgr IS3	2076991	2077389	399
12D	IS_e546712d_01985	86.84% ISBcen7_aa2	100% IS_e546712d_03247	IS3 ssgr IS3	2077753	2078217	465
12D	IS_e546712d_03247	86.84% ISBcen7_aa2	100% IS_e546712d_01985	IS3 ssgr IS3	3402334	3401870	465
12D	IS_e546712d_03248	79.69% ISPsy11_aa1	100% IS_e546712d_01984	IS3 ssgr IS3	3403096	3402698	399
12D	IS_e546712d_01036	88.05% ISRso16_aa1	No hit	IS3 ssgr IS407	1077702	1077899	198
12D	IS_e546712d_01039	79.27% ISPmar2_aa3	59.23% IS_e546712d_01047	IS3 ssgr IS407	1080964	1080134	831
12D	IS_e546712d_01047	96.35% ISRso16_aa2	59.23% IS_e546712d_01039	IS3 ssgr IS407	1087073	1086660	414
12D	IS_e546712d_00746	100% ISRme15_aa1	88.46% IS_e546712d_02845	IS3 ssgr IS51	771705	772034	330
12D	IS_e546712d_00747	100% ISRme15_aa2	87.13% IS_e546712d_02846	IS3 ssgr IS51	772184	772945	762
12D	IS_e546712d_02845	94.39% ISStma17_aa1	88.46% IS_e546712d_00746	IS3 ssgr IS51	2987280	2987603	324
12D	IS_e546712d_02846	90.46% IS401_aa2	87.13% IS_e546712d_00747	IS3 ssgr IS51	2987600	2988514	915
12D	IS_e546712d_03086	69.32% IS1086_aa1	100% IS_e546712d_03193	IS30	3224782	3225852	1.071
12D	IS_e546712d_03193	69.32% IS1086_aa1	100% IS_e546712d_03086	IS30	3340657	3339587	1.071

12D	IS_e546712d_00203	46.29% ISAcp2_aa1	58.31% IS_e546712d_02552	IS481	211342	213234	1.893
12D	IS_e546712d_00635	80% ISPpu19_aa3	73.33% IS_e546712d_03004	IS66	667355	667179	177
12D	IS_e546712d_00840	80.89% ISBcen19_aa3	100% IS_e546712d_03004	IS66	871563	870034	1.53
12D	IS_e546712d_00841	77.08% ISSal1_aa2	100% IS_e546712d_03005	IS66	871916	871626	291
12D	IS_e546712d_00842	64.94% ISBcen19_aa1	100% IS_e546712d_03006	IS66	872245	871958	288
12D	IS_e546712d_02693	64.94% ISBcen19_aa1	100% IS_e546712d_03006	IS66	2813360	2813647	288
12D	IS_e546712d_02694	77.08% ISSal1_aa2	100% IS_e546712d_03005	IS66	2813689	2813979	291
12D	IS_e546712d_02695	80.89% ISBcen19_aa3	100% IS_e546712d_03004	IS66	2814042	2815571	1.53
12D	IS_e546712d_03004	80.89% ISBcen19_aa3	100% IS_e546712d_02695	IS66	3134054	3132525	1.53
12D	IS_e546712d_03005	77.08% ISSal1_aa2	100% IS_e546712d_02694	IS66	3134407	3134117	291
12D	IS_e546712d_03006	64.94% ISBcen19_aa1	100% IS_e546712d_02693	IS66	3134736	3134449	288
12D	IS_e546712d_02443	49.39% ISWz1_aa1	52.09% IS_e546712d_03403	IS91	2534891	2535787	897
12D	IS_e546712d_02878	45.16% ISShvi3_aa1	46.19% IS_e546712d_02265	IS91	3012494	3011439	1.056
12D	IS_e546712d_03403	48.75% ISWz1_aa1	52.10% IS_e546712d_02443	IS91	3563657	3564643	987
12D	IS_e546712d_00743	77.85% ISIde1_aa1	No hit	ISL3	768235	76953	1.296
12D	IS_e546712d_01597	50.78% ISSm4_aa2	No hit	ISL3	1675920	1676981	1.062
12D	IS_e546712d_00505	52.74% ISMpo10_aa1	53.62% IS_e546712d_00041	Tn3	533286	533801	516
12D	IS_e546712d_00649	87.74% IS1071_aa1	No hit	Tn3	678981	681893	2.913
12D	IS_e546712d_02173	64.08% ISPa38_aa1	No hit	Tn3	2280453	2279863	591
12D	IS_e546712d_02566	51.38% TnShfr1_aa1	No hit	Tn3	2669479	2670783	1.305
12D	IS_e546712d_03040	43.27% TnShfr1_aa1	70.52% IS_e546712d_02554	Tn3	3175362	3174214	1.149
DTP0602	IS_b9bc5a47_01204	85.47% ISRta3_aa1	No hit	IS110	1270313	1270885	573
DTP0602	IS_b9bc5a47_02518	58.03% ISWpi13_aa1	No hit	IS110	2753225	2753584	360
DTP0602	IS_b9bc5a47_00237	45.22% ISArch1_aa1	99.81% IS_b9bc5a47_03116	IS1182	238635	240239	1.605
DTP0602	IS_b9bc5a47_03116	45.06% ISArch1_aa1	99.81% IS_b9bc5a47_00237	IS1182	3397538	3399142	1.605
DTP0602	IS_b9bc5a47_01522	66.72% ISMyca1_aa1	99.13% IS_b9bc5a47_04108	IS1634	1628508	1630244	1.737
DTP0602	IS_b9bc5a47_02433	69.55% ISMasp1_aa1	58.42% IS_b9bc5a47_04108	IS1634	2656238	2657140	903
DTP0602	IS_b9bc5a47_02434	65.48% ISMyca1_aa1	66.66% IS_b9bc5a47_01522	IS1634	2657097	2657981	885
DTP0602	IS_b9bc5a47_04108	66.90% ISMyca1_aa1	99.13% IS_b9bc5a47_01522	IS1634	4471042	4472778	1.737
DTP0602	IS_b9bc5a47_00238	90.41% ISAba30_aa2	99.75% IS_b9bc5a47_03920	IS200/IS605	241524	240301	1.224
DTP0602	IS_b9bc5a47_00239	95.62% ISAba30_aa1	99.27% IS_b9bc5a47_04098	IS200/IS605	241547	24196	414
DTP0602	IS_b9bc5a47_03114	94.89% ISAba30_aa1	100% IS_b9bc5a47_04098	IS200/IS605	3396201	3395788	414
DTP0602	IS_b9bc5a47_03115	90.17% ISAba30_aa2	99.75% IS_b9bc5a47_04097	IS200/IS605	3396224	3397447	1.224
DTP0602	IS_b9bc5a47_03919	94.89% ISAba30_aa1	100% IS_b9bc5a47_04098	IS200/IS605	4267255	4266842	414

DTP0602	IS_b9bc5a47_03920	90.17% ISAba30_aa2	99.75% IS_b9bc5a47_00238	IS200/IS605	4267278	4268501	1.224
DTP0602	IS_b9bc5a47_04097	90.41% ISAba30_aa2	99.75% IS_b9bc5a47_03115	IS200/IS605	4461573	4460350	1.224
DTP0602	IS_b9bc5a47_04098	94.89% ISAba30_aa1	100% IS_b9bc5a47_03919	IS200/IS605	4461596	4462009	414
DTP0602	IS_b9bc5a47_00581	41.57% ISHtu6_aa2	47.28% IS_b9bc5a47_01338	IS200/IS605 ssgr IS1341	604008	605549	1.542
DTP0602	IS_b9bc5a47_01338	57.44% ISHaha8_aa2	48.60% IS_b9bc5a47_03115	IS200/IS605 ssgr IS1341	1418462	1417404	1.059
DTP0602	IS_b9bc5a47_03239	58.15% IS1136_aa2	49.40% IS_b9bc5a47_04097	IS200/IS605 ssgr IS1341	3519167	3518280	888
DTP0602	IS_b9bc5a47_01950	60.83% ISMac3_aa2	No hit	IS21	2097542	2096760	783
DTP0602	IS_b9bc5a47_02785	85.27% ISAzo12_aa1	No hit	IS21	3048185	3049090	906
DTP0602	IS_b9bc5a47_02998	90.93% ISRta2_aa1	No hit	IS256	3267048	3268298	1.251
DTP0602	IS_b9bc5a47_01843	68.17% ISRso13_aa1	No hit	IS4 ssgr IS4	1968040	1966664	1.377
DTP0602	IS_b9bc5a47_00014	79.52% ISAzo5_aa1	100% IS_b9bc5a47_00555	IS4 ssgr IS50	1879	17453	1.338
DTP0602	IS_b9bc5a47_00555	79.52% ISAzo5_aa1	100% IS_b9bc5a47_00014	IS4 ssgr IS50	569936	571273	1.338
DTP0602	IS_b9bc5a47_02461	57.41% ISGvi2_aa1	50.68% IS_b9bc5a47_00555	IS4 ssgr IS50	2691618	2692754	1.137
DTP0602	IS_b9bc5a47_01269	53.36% ISHne2_aa1	No hit	IS481	1341006	1341998	993
DTP0602	IS_b9bc5a47_01768	91.56% ISRme16_aa1	No hit	IS5	1890292	1891305	1.014
DTP0602	IS_b9bc5a47_01370	72.22% ISAli12B_aa2	100% IS_b9bc5a47_02487	IS5 ssgr IS427	1455612	1455265	348
DTP0602	IS_b9bc5a47_01371	79.43% ISAli9_aa1	100% IS_b9bc5a47_02486	IS5 ssgr IS427	1456015	1455680	336
DTP0602	IS_b9bc5a47_02486	79.43% ISAli9_aa1	100% IS_b9bc5a47_01371	IS5 ssgr IS427	2721032	2721367	336
DTP0602	IS_b9bc5a47_02487	72.22% ISAli12B_aa2	100% IS_b9bc5a47_01370	IS5 ssgr IS427	2721435	2721782	348
DTP0602	IS_b9bc5a47_01883	82.56% ISBmu20_aa1	No hit	IS5 ssgr IS5	2009980	2010345	366
DTP0602	IS_b9bc5a47_01928	89.26% ISBmu20_aa1	No hit	IS5 ssgr IS5	2060605	2061249	645
DTP0602	IS_b9bc5a47_00580	69.38% ISCfe1_aa1	No hit	IS607	603428	604027	600
DTP0602	IS_b9bc5a47_03240	64.8% ISCARN56_aa2	49.56% IS_b9bc5a47_04097	IS607	3519586	3519215	372
DTP0602	IS_b9bc5a47_01707	80.36% ISCARN31_aa3	97.55% IS_b9bc5a47_02076	IS630	1831630	1832613	984
DTP0602	IS_b9bc5a47_01779	75.69% ISThsp15_aa1	49.71% IS_b9bc5a47_01953	IS630	1903202	1902120	1.083
DTP0602	IS_b9bc5a47_01860	88.05% ISCARN39_aa1	58.55% IS_b9bc5a47_02033	IS630	1985796	1986281	486
DTP0602	IS_b9bc5a47_01861	90% ISCARN39_aa2	89.83% IS_b9bc5a47_03243	IS630	1986278	1986790	513
DTP0602	IS_b9bc5a47_01953	74.44% ISBmu8_aa1	49.71% IS_b9bc5a47_01779	IS630	2100417	2099299	1.119
DTP0602	IS_b9bc5a47_02033	78.29% ISAzo30_aa1	99.70% IS_b9bc5a47_02076	IS630	2217401	2218435	1.035
DTP0602	IS_b9bc5a47_02076	78.59% ISAzo30_aa1	99.70% IS_b9bc5a47_02033	IS630	2272784	2273818	1.035
DTP0602	IS_b9bc5a47_03243	83.89% ISCARN39_aa2	89.83% IS_b9bc5a47_01861	IS630	3522428	3523258	831
DTP0602	IS_b9bc5a47_01931	81.77% ISBmu30_aa2	50.34% IS_b9bc5a47_03711	IS66	2064112	2065515	1.404
DTP0602	IS_b9bc5a47_02793	92.85% ISBcen14_aa2	77.77% IS_b9bc5a47_03710	IS66	3058122	3058343	222
DTP0602	IS_b9bc5a47_03709	57.36% ISCro1_aa1	No hit	IS66	4048325	4048750	426
DTP0602	IS_b9bc5a47_03710	90.43% IS883_aa2	77.77% IS_b9bc5a47_02793	IS66	4048747	4049094	348

DTP0602	IS_b9bc5a47_03711	78.48% IS883_aa3	50.34% IS_b9bc5a47_01931	IS66	4049137	4050711	1.575
DTP0602	IS_b9bc5a47_00072	45.64% ISWz1_aa1	50.15% IS_b9bc5a47_00583	IS91	79929	80999	1.071
DTP0602	IS_b9bc5a47_00583	49.47% ISMno23_aa1	50.32% IS_b9bc5a47_00072	IS91	607135	606194	942
DTP0602	IS_b9bc5a47_01648	39.54% ISShvi3_aa1	95.69% IS_b9bc5a47_01921	IS91	1765181	1764534	648
DTP0602	IS_b9bc5a47_01921	41.71% ISShvi3_aa1	95.69% IS_b9bc5a47_01648	IS91	2053560	2052322	1.239
DTP0602	IS_b9bc5a47_01954	45.6% ISWz1_aa1	46.28% IS_b9bc5a47_00583	IS91	2101377	2100460	918
DTP0602	IS_b9bc5a47_01956	40.54% ISShvi3_aa1	41.85% IS_b9bc5a47_00583	IS91	2103538	2102306	1.233
DTP0602	IS_b9bc5a47_01649	91.21% ISBte2_aa1	51.49% IS_b9bc5a47_01946	ISKra4 ssgr ISAzba1	1765543	1766487	945
DTP0602	IS_b9bc5a47_01852	83.95% ISBusp5_aa1	50.33% IS_b9bc5a47_01649	ISKra4 ssgr ISAzba1	1978551	1977118	1.434
DTP0602	IS_b9bc5a47_01946	80.71% ISBte1_aa2	51.49% IS_b9bc5a47_01649	ISKra4 ssgr ISAzba1	2093077	2093604	528
DTP0602	IS_b9bc5a47_03389	43.53% ISSm4_aa1	51.77% IS_b9bc5a47_03391	ISL3	3689901	3685915	3.987
DTP0602	IS_b9bc5a47_03391	45.59% ISSm4_aa1	51.77% IS_b9bc5a47_03389	ISL3	3692563	3691082	1.482
DTP0602	IS_b9bc5a47_02063	65.95% ISKpn21_aa1	No hit	ISNCY ssgr IS1202	2255700	2256041	342
DTP0602	IS_b9bc5a47_00233	49.18% TnShfr1_aa1	No hit	Tn3	236444	235458	987
DTP0602	IS_b9bc5a47_00566	54.25% ISMpo10_aa1	50.42% IS_b9bc5a47_03659	Tn3	583165	582581	585
DTP0602	IS_b9bc5a47_00655	53.06% ISSba14_aa1	No hit	Tn3	683362	682706	657
DTP0602	IS_b9bc5a47_01930	76.87% IS882_aa1	No hit	Tn3	2063462	2062992	471
DTP0602	IS_b9bc5a47_01948	97.40% IS882_aa1	No hit	Tn3	2095208	2096149	942
DTP0602	IS_b9bc5a47_02542	82.5% ISMpo10_aa2	No hit	Tn3	2782015	2782554	540
DTP0602	IS_b9bc5a47_03928	45.02% TnShfr1_aa1	No hit	Tn3	4276385	4275414	972

FC1138	IS_fdddf1d6_00089	80.30% IS1600_aa1	100% IS_fdddf1d6_03179	IS21	9223	93747	1.518
FC1138	IS_fdddf1d6_00090	98.46% ISRme4_aa2	100% IS_fdddf1d6_03180	IS21	93737	94522	786
FC1138	IS_fdddf1d6_00393	80.30% IS1600_aa1	100% IS_fdddf1d6_03179	IS21	38831	389827	1.518
FC1138	IS_fdddf1d6_00394	98.46% ISRme4_aa2	100% IS_fdddf1d6_03180	IS21	389817	390602	786
FC1138	IS_fdddf1d6_00785	80.30% IS1600_aa1	100% IS_fdddf1d6_03179	IS21	80376	805277	1.518
FC1138	IS_fdddf1d6_00786	98.46% ISRme4_aa2	100% IS_fdddf1d6_03180	IS21	805267	806052	786
FC1138	IS_fdddf1d6_02223	80.88% IS1600_aa1	99.21% IS_fdddf1d6_02623	IS21	2299083	2300609	1.527
FC1138	IS_fdddf1d6_02224	98.46% ISRme4_aa2	100% IS_fdddf1d6_03180	IS21	2300599	2301384	786
FC1138	IS_fdddf1d6_02622	98.46% ISRme4_aa2	100% IS_fdddf1d6_03180	IS21	2729584	2728799	786
FC1138	IS_fdddf1d6_02623	80.50% IS1600_aa1	99.80% IS_fdddf1d6_03179	IS21	2731091	2729574	1.518
FC1138	IS_fdddf1d6_03179	80.30% IS1600_aa1	100% IS_fdddf1d6_00785	IS21	3329997	3331514	1.518
FC1138	IS_fdddf1d6_03180	98.46% ISRme4_aa2	100% IS_fdddf1d6_02622	IS21	3331504	3332289	786
FC1138	IS_fdddf1d6_00092	60.69% ISMco1_aa2	100% IS_fdddf1d6_02225	IS3 ssgr IS3	95184	95795	612
FC1138	IS_fdddf1d6_00787	60.69% ISMco1_aa2	100% IS_fdddf1d6_02225	IS3 ssgr IS3	80674	806129	612

FC1138	IS_fdddf1d6_02225	60.69% ISMco1_aa2	100% IS_fdddf1d6_00787	IS3 ssgr IS3	2302072	2301461	612
FC1138	IS_fdddf1d6_01418	85.40% ISMlo4_aa3	52.55% IS_fdddf1d6_02225	IS3 ssgr IS51	1458362	1457505	858
FC1138	IS_fdddf1d6_01419	90.42% ISAtu4_aa1	No hit	IS3 ssgr IS51	1458652	1458359	294
FC1138	IS_fdddf1d6_02458	100% ISBvi1_aa1	100% IS_fdddf1d6_03006	IS4 ssgr IS4	2550124	2548925	1.2
FC1138	IS_fdddf1d6_02998	100% ISBvi1_aa1	100% IS_fdddf1d6_03006	IS4 ssgr IS4	3131574	3132773	1.2
FC1138	IS_fdddf1d6_03006	100% ISBvi1_aa1	100% IS_fdddf1d6_02998	IS4 ssgr IS4	3139712	3138513	1.2
FC1138	IS_fdddf1d6_00277	40.74% ISGur11_aa1	55.33% IS_fdddf1d6_02865	IS481	277665	278912	1.248
FC1138	IS_fdddf1d6_00301	52.43% IS481v1_aa1	No hit	IS481	299196	29942	225
FC1138	IS_fdddf1d6_02691	79.38% ISCARN33_aa1	No hit	IS5 ssgr IS427	2798126	2798560	435
FC1138	IS_fdddf1d6_02692	75.21% ISCARN85_aa2	No hit	IS5 ssgr IS427	2798557	2798910	354
FC1138	IS_fdddf1d6_01315	57.89% ISWpi10_aa2	No hit	IS630	1352361	1352083	279
FC1138	IS_fdddf1d6_00286	65.38% ISBcen19_aa1	100% IS_fdddf1d6_03308	IS66	28918	289551	372
FC1138	IS_fdddf1d6_00287	77.08% ISSal1_aa2	100% IS_fdddf1d6_03307	IS66	289593	289883	291
FC1138	IS_fdddf1d6_00288	80.89% ISBcen19_aa3	100% IS_fdddf1d6_03306	IS66	289946	291475	1.53
FC1138	IS_fdddf1d6_01413	80.89% ISBcen19_aa3	100% IS_fdddf1d6_03306	IS66	1455784	1454255	1.53
FC1138	IS_fdddf1d6_01414	77.08% ISSal1_aa2	100% IS_fdddf1d6_03307	IS66	1456137	1455847	291
FC1138	IS_fdddf1d6_01415	65.38% ISBcen19_aa1	100% IS_fdddf1d6_03308	IS66	1456550	1456179	372
FC1138	IS_fdddf1d6_03306	80.89% ISBcen19_aa3	100% IS_fdddf1d6_01413	IS66	3446864	3445335	1.53
FC1138	IS_fdddf1d6_03307	77.08% ISSal1_aa2	100% IS_fdddf1d6_01414	IS66	3447217	3446927	291
FC1138	IS_fdddf1d6_03308	65.38% ISBcen19_aa1	100% IS_fdddf1d6_01415	IS66	3447630	3447259	372
FC1138	IS_fdddf1d6_01433	44.02% ISShvi3_aa1	49.72% IS_fdddf1d6_03188	IS91	1473341	1472277	1.065
FC1138	IS_fdddf1d6_02764	45.71% ISTha3_aa2	50.30% IS_fdddf1d6_03731	IS91	2867645	2868580	936
FC1138	IS_fdddf1d6_03188	45.32% ISShvi3_aa1	49.72% IS_fdddf1d6_01433	IS91	3338252	3337209	1.044
FC1138	IS_fdddf1d6_03731	47.54% ISWz1_aa1	50.30% IS_fdddf1d6_02764	IS91	3895800	3896810	1.011
FC1138	IS_fdddf1d6_01285	53.31% ISSm4_aa2	56.90% IS_fdddf1d6_01772	ISL3	1323402	1324538	1.137
FC1138	IS_fdddf1d6_01772	76.16% ISSm4_aa2	56.82% IS_fdddf1d6_01285	ISL3	1823924	1822860	1.065
FC1138	IS_fdddf1d6_00630	52.27% ISMpo10_aa1	45.34% IS_fdddf1d6_00274	Tn3	642467	642994	528
FC1138	IS_fdddf1d6_01424	77.00% ISSba14_aa1	No hit	Tn3	1461925	1462503	579
FC1138	IS_fdddf1d6_01690	62% ISMpo10_aa2	No hit	Tn3	1738142	1738597	456
FC1138	IS_fdddf1d6_02868	53.14% TnShfr1_aa1	57.14% IS_fdddf1d6_03645	Tn3	2984959	2986263	1.305
SL2729	CP022792_02963	61.67% ISMno14_aa1	45.80% CP022792_03304	IS110 ssgr IS1111	3231194	3230181	1014
SL2729	CP022792_03304	88.75% ISBcen4_aa1	45.80% CP022792_02963	IS110 ssgr IS1111	3566153	3565134	102
SL2729	CP022792_00944	83.75% ISBusp4_aa1	100% CP022792_02742	IS1182	1029166	1030611	1446
SL2729	CP022792_02742	83.75% ISBusp4_aa1	100% CP022792_00944	IS1182	2999634	2998189	1446

SL2729	CP022792_00953	54.76% ISHpa1_aa1	No hit	IS1595 ssgr IS1016	1041156	1041935	780
SL2729	CP022792_00871	100% ISRso11_aa1	100% CP022792_01928	IS3 ssgr IS150	951963	952496	534
SL2729	CP022792_00872	99.64% ISRso11_aa2	100% CP022792_01927	IS3 ssgr IS150	952493	953329	837
SL2729	CP022792_01927	99.64% ISRso11_aa2	100% CP022792_00872	IS3 ssgr IS150	2146690	2145854	837
SL2729	CP022792_01928	100% ISRso11_aa1	100% CP022792_00871	IS3 ssgr IS150	2147220	2146687	534
SL2729	CP022792_01153	88.23% IS222_aa1	60.91% CP022792_01061	IS3 ssgr IS3	1258669	1258977	309
SL2729	CP022792_01154	88.78% IS222_aa2	55.76% CP022792_01060	IS3 ssgr IS3	1259160	1259831	672
SL2729	CP022792_00648	81.52% ISAisp2_aa1	100% CP022792_01061	IS3 ssgr IS51	706921	707199	279
SL2729	CP022792_00649	89.34% ISAisp2_aa2	100% CP022792_00951	IS3 ssgr IS51	707196	708071	876
SL2729	CP022792_00950	81.52% ISAisp2_aa1	100% CP022792_01061	IS3 ssgr IS51	1037956	1038234	279
SL2729	CP022792_00951	89.34% ISAisp2_aa2	100% CP022792_00649	IS3 ssgr IS51	1038231	1039106	876
SL2729	CP022792_01060	89.59% ISAisp2_aa2	100% CP022792_00951	IS3 ssgr IS51	1156296	1155631	666
SL2729	CP022792_01061	81.52% ISAisp2_aa1	100% CP022792_00950	IS3 ssgr IS51	1156782	1156504	279
SL2729	CP022792_01141	86.38% ISAisp2_aa2	94.94% CP022792_00951	IS3 ssgr IS51	1247628	1246837	792
SL2729	CP022792_01142	80.43% ISAisp2_aa1	96.73% CP022792_03055	IS3 ssgr IS51	1247903	1247625	279
SL2729	CP022792_03054	89.34% ISAisp2_aa2	98.28% CP022792_00951	IS3 ssgr IS51	3315355	3314480	876
SL2729	CP022792_03055	81.52% ISAisp2_aa1	97.82% CP022792_01061	IS3 ssgr IS51	3315630	3315352	279
SL2729	CP022792_00272	61.68% ISCro3_aa1	100% CP022792_02639	IS4	299487	300815	1329
SL2729	CP022792_00342	61.68% ISCro3_aa1	100% CP022792_02639	IS4	382162	380834	1329
SL2729	CP022792_00460	61.68% ISCro3_aa1	100% CP022792_02639	IS4	498688	49736	1329
SL2729	CP022792_00579	61.44% ISCro3_aa1	100% CP022792_01238	IS4	621831	623159	1329
SL2729	CP022792_01011	61.68% ISCro3_aa1	100% CP022792_02639	IS4	1102255	1100927	1329
SL2729	CP022792_01238	61.44% ISCro3_aa1	100% CP022792_00579	IS4	1356360	1357688	1329
SL2729	CP022792_01738	61.68% ISCro3_aa1	100% CP022792_03396	IS4	1884475	1885803	1329
SL2729	CP022792_02313	61.68% ISCro3_aa1	100% CP022792_03396	IS4	2567586	2566258	1329
SL2729	CP022792_02368	61.68% ISCro3_aa1	99.77% CP022792_02639	IS4	2624044	2622716	1329
SL2729	CP022792_02395	61.68% ISCro3_aa1	100% CP022792_02639	IS4	2653215	2651887	1329
SL2729	CP022792_02464	61.68% ISCro3_aa1	100% CP022792_03396	IS4	2719495	2720823	1329
SL2729	CP022792_02639	61.68% ISCro3_aa1	100% CP022792_02395	IS4	2897535	2898863	1329
SL2729	CP022792_03396	61.68% ISCro3_aa1	100% CP022792_02464	IS4	3676675	3675347	1329
SL2729	CP022792_00260	78.03% ISAzo5_aa1	100% CP022792_00910	IS4 ssgr IS50	281786	283111	1326
SL2729	CP022792_00643	78.03% ISAzo5_aa1	100% CP022792_00910	IS4 ssgr IS50	69907	700395	1326
SL2729	CP022792_00910	78.03% ISAzo5_aa1	100% CP022792_00643	IS4 ssgr IS50	992515	99384	1326
SL2729	CP022792_01357	97.81% ISRso1_aa1	100% CP022792_02018	IS5	1487056	1486232	825
SL2729	CP022792_02018	97.81% ISRso1_aa1	100% CP022792_01357	IS5	2248824	2249648	825

SL2729	CP022792_00235	99.37% IS1405_aa1	99.68% CP022792_03178	IS5 ssgr IS5	246722	245757	966
SL2729	CP022792_00277	99.06% IS1405_aa1	100% CP022792_03178	IS5 ssgr IS5	305355	30632	966
SL2729	CP022792_00661	94.51% IS1021_aa1	100% CP022792_03240	IS5 ssgr IS5	72421	725196	987
SL2729	CP022792_00768	94.51% IS1021_aa1	100% CP022792_03240	IS5 ssgr IS5	849201	848215	987
SL2729	CP022792_01355	99.06% IS1405_aa1	100% CP022792_03178	IS5 ssgr IS5	1484547	1483582	966
SL2729	CP022792_01521	99.06% IS1405_aa1	100% CP022792_03178	IS5 ssgr IS5	1650733	1649768	966
SL2729	CP022792_02391	99.06% IS1405_aa1	100% CP022792_03178	IS5 ssgr IS5	2648407	2647442	966
SL2729	CP022792_02497	99.06% IS1405_aa1	100% CP022792_03178	IS5 ssgr IS5	2764923	2765888	966
SL2729	CP022792_02515	99.06% IS1405_aa1	100% CP022792_03178	IS5 ssgr IS5	2783850	2782885	966
SL2729	CP022792_03060	99.06% IS1405_aa1	100% CP022792_03178	IS5 ssgr IS5	3321084	3320119	966
SL2729	CP022792_03079	94.51% IS1021_aa1	100% CP022792_03240	IS5 ssgr IS5	3334467	3335453	987
SL2729	CP022792_03126	99.06% IS1405_aa1	100% CP022792_03178	IS5 ssgr IS5	3380774	3381739	966
SL2729	CP022792_03178	99.06% IS1405_aa1	100% CP022792_03126	IS5 ssgr IS5	3440645	3439680	966
SL2729	CP022792_03240	94.51% IS1021_aa1	100% CP022792_03079	IS5 ssgr IS5	3503490	3504476	987
SL2729	CP022792_00008	100% IS1420_aa1	100% CP022792_02786	IS5 ssgr IS903	15895	14939	957
SL2729	CP022792_00642	100% IS1420_aa1	100% CP022792_02786	IS5 ssgr IS903	697137	698093	957
SL2729	CP022792_00766	100% IS1420_aa1	100% CP022792_02786	IS5 ssgr IS903	838705	839661	957
SL2729	CP022792_02746	100% IS1420_aa1	100% CP022792_02786	IS5 ssgr IS903	3003034	3002078	957
SL2729	CP022792_02748	100% IS1420_aa1	100% CP022792_02786	IS5 ssgr IS903	3004707	3005663	957
SL2729	CP022792_02786	100% IS1420_aa1	100% CP022792_02748	IS5 ssgr IS903	3044541	3045497	957
SL2729	CP022792_00882	47.5% ISTha3_aa2	51.50% CP022792_03373	IS91	964318	963392	927
SL2729	CP022792_01619	40.65% ISShvi3_aa1	51.94% CP022792_01605	IS91	1760851	1759787	1065
SL2729	CP022792_03373	43.33% ISMno24_aa2	52.12% CP022792_00882	IS91	3654126	3655115	990
SL2729	CP022792_00038	38.97% ISKpn25_aa1	No hit	ISL3	50959	52404	1446
SL2729	CP022792_01865	62.5% ISKpn21_aa1	93.33% CP022792_01866	ISNCY ssgr IS1202	2081189	2080788	402
SL2729	CP022792_01866	72.27% ISKpn21_aa1	93.33% CP022792_01865	ISNCY ssgr IS1202	2082818	2081343	1476
SL2729	CP022792_00193	61.81% ISMpo10_aa3	73.17% CP022792_02490	Tn3	21438	212992	1389
SL2729	CP022792_01609	75.40% ISPa40_aa4	54.09% CP022792_00193	Tn3	1747091	1747687	597
SL2729	CP022792_02490	55.06% ISMpo10_aa3	73.17% CP022792_00193	Tn3	2749541	2748159	1383
T110	IS_b246579f_01867	95.76% ISBma3_aa1	44.82% IS_b246579f_03953	IS110	1675451	1674246	1206
T110	IS_b246579f_03953	88.75% ISBcen4_aa1	No hit	IS110 ssgr IS1111	3496678	3495659	102
T110	IS_b246579f_03016	49.48% ISHvo5_aa1	100% IS_b246579f_00305	IS1595 ssgr ISH4	2686073	2685285	789
T110	IS_b246579f_00376	100% ISRso11_aa1	No hit	IS3 ssgr IS150	356251	356679	429
T110	IS_b246579f_00377	99.64% ISRso11_aa2	52% IS_b246579f_03078	IS3 ssgr IS150	356676	357512	837

T110	IS_b246579f_01298	88.23% IS222_aa1	100% IS_b246579f_03122	IS3 ssgr IS3	1168531	1168839	309
T110	IS_b246579f_01299	89.23% IS222_aa2	97.75% IS_b246579f_03123	IS3 ssgr IS3	1169022	1169693	672
T110	IS_b246579f_03122	88.23% IS222_aa1	100% IS_b246579f_01298	IS3 ssgr IS3	2787882	2788190	309
T110	IS_b246579f_03123	89.23% IS222_aa2	97.75% IS_b246579f_01299	IS3 ssgr IS3	2788373	2789044	672
T110	IS_b246579f_03352	85.71% ISAtu5_aa3	79.51% IS_b246579f_03873	IS3 ssgr IS407	2982798	2983187	390
T110	IS_b246579f_03603	92.20% ISRso16_aa2	No hit	IS3 ssgr IS407	3207804	3207505	300
T110	IS_b246579f_03873	100% ISRso14_aa1	79.51% IS_b246579f_03352	IS3 ssgr IS407	3428725	3428988	264
T110	IS_b246579f_03874	90.12% ISRso14_aa2	No hit	IS3 ssgr IS407	3429294	3429593	300
T110	IS_b246579f_01283	85.64% ISAisp2_aa2	95.89% IS_b246579f_03078	IS3 ssgr IS51	1156632	1155985	648
T110	IS_b246579f_01284	80.43% ISAisp2_aa1	96.73% IS_b246579f_03079	IS3 ssgr IS51	1156907	1156629	279
T110	IS_b246579f_03078	89.34% ISAisp2_aa2	95.89% IS_b246579f_01283	IS3 ssgr IS51	2741908	2741033	876
T110	IS_b246579f_03079	81.52% ISAisp2_aa1	96.73% IS_b246579f_01284	IS3 ssgr IS51	2742183	2741905	279
T110	IS_b246579f_00553	63.32% ISCro3_aa1	94.73% IS_b246579f_02032	IS4	510175	511512	1338
T110	IS_b246579f_00602	61.68% ISCro3_aa1	100% IS_b246579f_03771	IS4	551138	54981	1329
T110	IS_b246579f_01412	61.91% ISCro3_aa1	99.77% IS_b246579f_03771	IS4	1276649	1275321	1329
T110	IS_b246579f_01439	61.68% ISCro3_aa1	100% IS_b246579f_03771	IS4	1301613	1300285	1329
T110	IS_b246579f_02032	61.68% ISCro3_aa1	100% IS_b246579f_03771	IS4	1815865	1814537	1329
T110	IS_b246579f_02879	61.68% ISCro3_aa1	100% IS_b246579f_03771	IS4	2559406	2560734	1329
T110	IS_b246579f_03220	63.74% ISCro3_aa1	99.62% IS_b246579f_01412	IS4	2868460	2867591	870
T110	IS_b246579f_03771	61.68% ISCro3_aa1	100% IS_b246579f_02879	IS4	3337280	3338608	1329
T110	IS_b246579f_04083	61.68% ISCro3_aa1	99.77% IS_b246579f_03771	IS4	3611405	3612733	1329
T110	IS_b246579f_03219	60% ISCro6_aa1	100% IS_b246579f_00553	IS4 ssgr IS4	2867633	2867124	510
T110	IS_b246579f_01446	97.81% ISRso1_aa1	100% IS_b246579f_03869	IS5	1309456	1308632	825
T110	IS_b246579f_01861	99.11% ISRso1_aa1	99.55% IS_b246579f_03869	IS5	1668968	1668291	678
T110	IS_b246579f_02112	97.81% ISRso1_aa1	100% IS_b246579f_03869	IS5	1887350	1888174	825
T110	IS_b246579f_03038	97.44% ISRso1_aa1	98.17% IS_b246579f_03869	IS5	2707544	2708368	825
T110	IS_b246579f_03088	97.44% ISRso1_aa1	99.63% IS_b246579f_03869	IS5	2752974	2752150	825
T110	IS_b246579f_03108	98.67% ISRso1_aa1	99.11% IS_b246579f_03869	IS5	2775285	2774605	681
T110	IS_b246579f_03109	97.81% ISRso1_aa1	100% IS_b246579f_03869	IS5	2775627	2776451	825
T110	IS_b246579f_03111	95.91% ISRso1_aa1	96.42% IS_b246579f_03869	IS5	2778215	2777622	594
T110	IS_b246579f_03869	97.81% ISRso1_aa1	100% IS_b246579f_03109	IS5	3425442	3426266	825
T110	IS_b246579f_01154	100% IS1421_aa1	100% IS_b246579f_04084	IS5 ssgr IS427	1039854	1040237	384
T110	IS_b246579f_03460	85.44% IS1421_aa3	86.44% IS_b246579f_01154	IS5 ssgr IS427	3076197	3075394	804
T110	IS_b246579f_04084	100% IS1421_aa1	100% IS_b246579f_01154	IS5 ssgr IS427	3612994	3613398	405
T110	IS_b246579f_00298	85.71% ISAau3_aa1	80.28% IS_b246579f_00333	IS5 ssgr IS5	277507	2778	294

T110	IS_b246579f_00333	99.37% IS1405_aa1	100% IS_b246579f_03895	IS5 ssgr IS5	31416	315125	966
T110	IS_b246579f_01551	98.91% IS1405_aa1	100% IS_b246579f_00333	IS5 ssgr IS5	1401885	1402481	597
T110	IS_b246579f_01552	100% IS1405_aa1	100% IS_b246579f_03731	IS5 ssgr IS5	1402439	1402849	411
T110	IS_b246579f_03729	98.86% IS1405_aa1	100% IS_b246579f_03896	IS5 ssgr IS5	3302515	3302790	276
T110	IS_b246579f_03730	100% IS1405_aa1	100% IS_b246579f_01551	IS5 ssgr IS5	3302850	3303110	261
T110	IS_b246579f_03731	100% IS1405_aa1	100% IS_b246579f_01552	IS5 ssgr IS5	3303068	3303478	411
T110	IS_b246579f_03895	100% IS1405_aa1	100% IS_b246579f_00333	IS5 ssgr IS5	3448896	3448267	630
T110	IS_b246579f_03896	98.86% IS1405_aa1	100% IS_b246579f_03729	IS5 ssgr IS5	3449231	3448956	276
T110	IS_b246579f_02048	67.18% ISCARN39_aa2	No hit	IS630	1830426	1830626	201
T110	IS_b246579f_03375	99.69% ISRso17_aa1	No hit	IS701	3002531	3003547	1017
T110	IS_b246579f_00623	46.47% ISShvi3_aa1	58.67% IS_b246579f_01882	IS91	567979	568821	843
T110	IS_b246579f_01037	47.5% ISTha3_aa2	49.76% IS_b246579f_04049	IS91	942807	941881	927
T110	IS_b246579f_00009	36.11% ISSm4_aa4	No hit	ISL3	13188	10171	3018
T110	IS_b246579f_00011	50.42% ISKpn25_aa1	No hit	ISL3	15399	14737	663
T110	IS_b246579f_00012	43.31% ISSm4_aa1	41.43% IS_b246579f_00048	ISL3	16355	15396	960
T110	IS_b246579f_00048	38.97% ISKpn25_aa1	41.85% IS_b246579f_00012	ISL3	51024	52469	1446
T110	IS_b246579f_02241	64.60% ISKpn21_aa1	No hit	ISNCY ssgr IS1202	2014922	2014521	402
T110	IS_b246579f_02242	72.22% ISKpn21_aa1	No hit	ISNCY ssgr IS1202	2015777	2015073	705
T110	IS_b246579f_02243	82.75% ISKpn21_aa1	No hit	ISNCY ssgr IS1202	2016151	2015831	321
T110	IS_b246579f_00229	54.08% ISMpo10_aa3	98.81% IS_b246579f_00313	Tn3	216327	214939	1389
T110	IS_b246579f_00313	54.71% ISMpo10_aa3	98.81% IS_b246579f_00229	Tn3	290865	289804	1062
T110	IS_b246579f_01872	78.44% ISSba14_aa1	46.25% IS_b246579f_03093	Tn3	1679761	1680315	555
T110	IS_b246579f_03014	54.43% ISMpo10_aa3	96.53% IS_b246579f_03092	Tn3	2683861	2682008	1854
T110	IS_b246579f_03093	54.16% ISMpo10_aa3	100% IS_b246579f_03014	Tn3	2757209	2756667	543

T51	NZ_CP022770_00702	70.76% ISPst3_aa2	No hit	IS21	745202	744918	285
T51	NZ_CP022770_01046	92.85% ISXca2_aa2	55.76% NZ_CP022770_02441	IS3 ssgr IS407	1120378	1119863	516
T51	NZ_CP022770_02441	97.33% ISRso16_aa2	55.76% NZ_CP022770_01046	IS3 ssgr IS407	2653502	2652711	792
T51	NZ_CP022770_02638	72.41% ISAtu5_aa1	70% NZ_CP022770_02443	IS3 ssgr IS407	2863320	2863580	261
T51	NZ_CP022770_03034	56.77% IS1421_aa3	No hit	IS5 ssgr IS427	3280022	3279396	627
T51	NZ_CP022770_02440	80.30% ISCARN25_aa1	79.10% NZ_CP022770_02650	IS630	2651989	2652222	234
T51	NZ_CP022770_02650	84.21% ISCARN25_aa1	79.10% NZ_CP022770_02440	IS630	2880280	2880534	255
T51	NZ_CP022770_00452	44.36% ISShvi3_aa1	No hit	IS91	471956	472999	1.044
T51	NZ_CP022770_00798	47.16% ISTha3_aa2	52.59% NZ_CP022770_03193	IS91	844947	844021	927
T51	NZ_CP022770_03193	48.75% ISWz1_aa1	52.76% NZ_CP022770_00798	IS91	3459047	3460036	990

T51	NZ_CP022770_00039	40.71% ISKpn25_aa1	50.76% NZ_CP022770_01038	ISL3	44434	45879	1.446
T51	NZ_CP022770_01038	42.36% ISSm4_aa1	50.76% NZ_CP022770_00039	ISL3	1110391	1111848	1.458
T51	NZ_CP022770_01800	55.89% ISKpn21_aa1	No hit	ISNCY ssgr IS1202	1955905	1955414	492
T51	NZ_CP022770_00191	53.08% ISMpo10_aa3	No hit	Tn3	204521	203145	1.377
T51	NZ_CP022770_00632	53.33% TnShfr1_aa1	No hit	Tn3	664821	666032	1.212
SL3175	NZ_CP022788_00703	73.84% ISPst3_aa2	No hit	IS21	75111	750826	285
SL3175	NZ_CP022788_01672	47.82% ISBrsa1_aa2	46.95% NZ_CP022788_02667	IS3 ssgr IS3	1823430	1822969	462
SL3175	NZ_CP022788_02457	63.25% ISBcen17_aa3	No hit	IS3 ssgr IS3	2699048	2698371	678
SL3175	NZ_CP022788_00201	98.86% ISRso16_aa1	100% NZ_CP022788_02666	IS3 ssgr IS407	219477	219743	267
SL3175	NZ_CP022788_00343	96% ISRso14_aa2	99.63% NZ_CP022788_01368	IS3 ssgr IS407	370936	370109	828
SL3175	NZ_CP022788_00344	98.85% IS407_aa1	100% NZ_CP022788_02460	IS3 ssgr IS407	371226	370963	264
SL3175	NZ_CP022788_00524	98.85% IS407_aa1	100% NZ_CP022788_02460	IS3 ssgr IS407	548792	549055	264
SL3175	NZ_CP022788_00525	97.09% ISRso14_aa2	98.90% NZ_CP022788_00343	IS3 ssgr IS407	549082	549909	828
SL3175	NZ_CP022788_01368	96.36% ISRso14_aa2	99.63% NZ_CP022788_00343	IS3 ssgr IS407	1470988	1470161	828
SL3175	NZ_CP022788_01369	97.70% IS407_aa1	98.85% NZ_CP022788_02460	IS3 ssgr IS407	1471278	1471015	264
SL3175	NZ_CP022788_02315	97.25% ISRso14_aa2	100% NZ_CP022788_00525	IS3 ssgr IS407	2552366	2551818	549
SL3175	NZ_CP022788_02316	94.73% ISRso14_aa2	100% NZ_CP022788_02461	IS3 ssgr IS407	2552647	2552366	282
SL3175	NZ_CP022788_02317	98.85% IS407_aa1	100% NZ_CP022788_02460	IS3 ssgr IS407	2552937	2552674	264
SL3175	NZ_CP022788_02451	98.86% ISRso16_aa1	100% NZ_CP022788_02666	IS3 ssgr IS407	2693792	2694058	267
SL3175	NZ_CP022788_02452	96.95% ISRso16_aa2	100% NZ_CP022788_02667	IS3 ssgr IS407	2694142	2694933	792
SL3175	NZ_CP022788_02460	98.85% IS407_aa1	100% NZ_CP022788_02317	IS3 ssgr IS407	2702176	2702439	264
SL3175	NZ_CP022788_02461	94.73% ISRso14_aa2	100% NZ_CP022788_02316	IS3 ssgr IS407	2702466	2702747	282
SL3175	NZ_CP022788_02462	96.70% ISRso14_aa2	100% NZ_CP022788_01368	IS3 ssgr IS407	2702747	2703295	549
SL3175	NZ_CP022788_02666	98.86% ISRso16_aa1	100% NZ_CP022788_02451	IS3 ssgr IS407	2920836	2921102	267
SL3175	NZ_CP022788_02667	96.95% ISRso16_aa2	100% NZ_CP022788_02452	IS3 ssgr IS407	2921186	2921977	792
SL3175	NZ_CP022788_00150	71.17% ISNGR9_aa2	100% NZ_CP022788_02305	IS5 ssgr IS427	173753	173394	360
SL3175	NZ_CP022788_00151	74.76% ISRel13_aa3	100% NZ_CP022788_02306	IS5 ssgr IS427	174151	173783	369
SL3175	NZ_CP022788_02305	71.17% ISNGR9_aa2	100% NZ_CP022788_00150	IS5 ssgr IS427	2541435	2541076	360
SL3175	NZ_CP022788_02306	74.76% ISRel13_aa3	100% NZ_CP022788_00151	IS5 ssgr IS427	2541833	2541465	369
SL3175	NZ_CP022788_01143	88.79% ISCARN25_aa1	No hit	IS630	1224784	1223765	102
SL3175	NZ_CP022788_00799	47.36% ISMno23_aa1	52.59% NZ_CP022788_03213	IS91	850881	849955	927
SL3175	NZ_CP022788_03213	48.75% ISWz1_aa1	52.59% NZ_CP022788_00799	IS91	3510056	3511045	990
SL3175	NZ_CP022788_00041	41.17% ISSm4_aa1	39.30% NZ_CP022788_02489	ISL3	50634	52079	1446
SL3175	NZ_CP022788_02489	47.26% ISKpn25_aa1	39.30% NZ_CP022788_00041	ISL3	2734856	2737273	2418

SL3175	NZ_CP022788_02491	75.29% ISSm4_aa2	No hit	ISL3	2738490	2739533	1044
SL3175	NZ_CP022788_02492	38.28% ISSm4_aa4	No hit	ISL3	2739526	2742090	2565
SL3175	NZ_CP022788_00009	50.46% ISPa4_aa1	No hit	ISNCY	1582	17595	1776
SL3175	NZ_CP022788_01673	77.27% ISKpn21_aa1	84.84% NZ_CP022788_01674	ISNCY ssgr IS1202	1823865	1823518	348
SL3175	NZ_CP022788_01674	72.27% ISKpn21_aa1	84.84% NZ_CP022788_01673	ISNCY ssgr IS1202	1825674	1824199	1476
SL3175	NZ_CP022788_00624	53.33% TnShfr1_aa1	No hit	Tn3	662868	664115	1248
SL3175	NZ_CP022788_00987	61.32% ISPa38_aa1	66.03% NZ_CP022788_02380	Tn3	1048340	1048930	591
SL3175	NZ_CP022788_02380	52.63% ISMpo10_aa3	66.03% NZ_CP022788_00987	Tn3	2617955	2616576	138
T11	NZ_CP022776_00703	70.76% ISPst3_aa2	No hit	IS21	748068	747784	285
T11	NZ_CP022776_01045	92.85% ISXca2_aa2	55.76% NZ_CP022776_02436	IS3 ssgr IS407	1120819	1120304	516
T11	NZ_CP022776_02436	97.33% ISRso16_aa2	55.76% NZ_CP022776_01045	IS3 ssgr IS407	2653929	2653138	792
T11	NZ_CP022776_02633	72.41% ISAtu5_aa1	70% NZ_CP022776_02438	IS3 ssgr IS407	2863745	2864005	261
T11	NZ_CP022776_03076	56.77% IS1421_aa3	No hit	IS5 ssgr IS427	3332533	3331907	627
T11	NZ_CP022776_02435	80.30% ISCARN25_aa1	79.10% NZ_CP022776_02645	IS630	2652416	2652649	234
T11	NZ_CP022776_02645	84.21% ISCARN25_aa1	79.10% NZ_CP022776_02435	IS630	2880705	2880959	255
T11	NZ_CP022776_00454	45.62% ISShvi3_aa1	43.49% NZ_CP022776_02741	IS91	471808	472731	924
T11	NZ_CP022776_00800	47.16% ISTha3_aa2	52.59% NZ_CP022776_03235	IS91	847813	846887	927
T11	NZ_CP022776_03235	48.75% ISWz1_aa1	52.76% NZ_CP022776_00800	IS91	3508776	3509765	990
T11	NZ_CP022776_00039	40.71% ISKpn25_aa1	50.76% NZ_CP022776_01040	ISL3	44434	45879	1.446
T11	NZ_CP022776_01040	42.36% ISSm4_aa1	50.76% NZ_CP022776_00039	ISL3	1113259	1114716	1.458
T11	NZ_CP022776_01796	55.89% ISKpn21_aa1	No hit	ISNCY ssgr IS1202	1956401	1955910	492
T11	NZ_CP022776_00191	53.08% ISMpo10_aa3	No hit	Tn3	204275	202899	1.377
T11	NZ_CP022776_00633	53.33% TnShfr1_aa1	No hit	Tn3	667687	668898	1.212
T98	NZ_CP022759_00701	73.84% ISPst3_aa2	No hit	IS21	751106	750822	285
T98	NZ_CP022759_01671	47.82% ISBrsa1_aa2	46.95% NZ_CP022759_02665	IS3 ssgr IS3	1823414	1822953	462
T98	NZ_CP022759_02457	63.25% ISBcen17_aa3	No hit	IS3 ssgr IS3	2699031	2698354	678
T98	NZ_CP022759_00201	98.86% ISRso16_aa1	100% NZ_CP022759_02664	IS3 ssgr IS407	219474	21974	267
T98	NZ_CP022759_00343	96% ISRso14_aa2	99.63% NZ_CP022759_01365	IS3 ssgr IS407	370933	370106	828
T98	NZ_CP022759_00344	98.85% IS407_aa1	100% NZ_CP022759_02460	IS3 ssgr IS407	371223	37096	264
T98	NZ_CP022759_00523	98.85% IS407_aa1	100% NZ_CP022759_02460	IS3 ssgr IS407	548788	549051	264
T98	NZ_CP022759_00524	97.09% ISRso14_aa2	98.90% NZ_CP022759_00343	IS3 ssgr IS407	549078	549905	828
T98	NZ_CP022759_01365	96.36% ISRso14_aa2	99.63% NZ_CP022759_00343	IS3 ssgr IS407	1470983	1470156	828
T98	NZ_CP022759_01366	97.70% IS407_aa1	98.85% NZ_CP022759_02460	IS3 ssgr IS407	1471273	1471010	264

T98	NZ_CP022759_02316	97.25% ISRso14_aa2	100% NZ_CP022759_00524	IS3 ssgr IS407	2552350	2551802	549
T98	NZ_CP022759_02317	94.73% ISRso14_aa2	100% NZ_CP022759_02461	IS3 ssgr IS407	2552631	2552350	282
T98	NZ_CP022759_02318	98.85% IS407_aa1	100% NZ_CP022759_02460	IS3 ssgr IS407	2552921	2552658	264
T98	NZ_CP022759_02451	98.86% ISRso16_aa1	100% NZ_CP022759_02664	IS3 ssgr IS407	2693775	2694041	267
T98	NZ_CP022759_02452	96.95% ISRso16_aa2	100% NZ_CP022759_02665	IS3 ssgr IS407	2694125	2694916	792
T98	NZ_CP022759_02460	98.85% IS407_aa1	100% NZ_CP022759_02318	IS3 ssgr IS407	2702159	2702422	264
T98	NZ_CP022759_02461	94.73% ISRso14_aa2	100% NZ_CP022759_02317	IS3 ssgr IS407	2702449	2702730	282
T98	NZ_CP022759_02462	96.70% ISRso14_aa2	100% NZ_CP022759_01365	IS3 ssgr IS407	2702730	2703278	549
T98	NZ_CP022759_02664	98.86% ISRso16_aa1	100% NZ_CP022759_02451	IS3 ssgr IS407	2920824	2921090	267
T98	NZ_CP022759_02665	96.95% ISRso16_aa2	100% NZ_CP022759_02452	IS3 ssgr IS407	2921174	2921965	792
T98	NZ_CP022759_00151	71.17% ISNGR9_aa2	100% NZ_CP022759_02306	IS5 ssgr IS427	17375	173391	360
T98	NZ_CP022759_00152	74.76% ISRel13_aa3	100% NZ_CP022759_02307	IS5 ssgr IS427	174148	17378	369
T98	NZ_CP022759_02306	71.17% ISNGR9_aa2	100% NZ_CP022759_00151	IS5 ssgr IS427	2541419	2541060	360
T98	NZ_CP022759_02307	74.76% ISRel13_aa3	100% NZ_CP022759_00152	IS5 ssgr IS427	2541817	2541449	369
T98	NZ_CP022759_01140	88.79% ISCARN25_aa1	No hit	IS630	1224778	1223759	1.02
T98	NZ_CP022759_00797	47.36% ISMno23_aa1	52.59% NZ_CP022759_03212	IS91	850876	84995	927
T98	NZ_CP022759_03212	48.75% ISWz1_aa1	52.59% NZ_CP022759_00797	IS91	3510043	3511032	990
T98	NZ_CP022759_00041	41.17% ISSm4_aa1	39.30% NZ_CP022759_02489	ISL3	50634	52079	1.446
T98	NZ_CP022759_02489	47.26% ISKpn25_aa1	39.30% NZ_CP022759_00041	ISL3	2734839	2737256	2.418
T98	NZ_CP022759_02491	75.29% ISSm4_aa2	No hit	ISL3	2738473	2739516	1.044
T98	NZ_CP022759_02492	38.28% ISSm4_aa4	No hit	ISL3	2739509	2742628	3.12
T98	NZ_CP022759_00009	50.46% ISPa4_aa1	No hit	ISNCY	1582	17595	1.776
T98	NZ_CP022759_01672	77.27% ISKpn21_aa1	84.84% NZ_CP022759_01673	ISNCY ssgr IS1202	1823849	1823502	348
T98	NZ_CP022759_01673	72.27% ISKpn21_aa1	84.84% NZ_CP022759_01672	ISNCY ssgr IS1202	1825658	1824183	1.476
T98	NZ_CP022759_00623	53.33% TnShfr1_aa1	No hit	Tn3	662864	664111	1.248
T98	NZ_CP022759_00984	61.32% ISPa38_aa1	66.03% NZ_CP022759_02381	Tn3	1048335	1048925	591
T98	NZ_CP022759_02381	52.63% ISMpo10_aa3	66.03% NZ_CP022759_00984	Tn3	2617938	2616559	1.38

T12	CP022774_00704	69.23% ISPst3_aa2	No hit	IS21	75061	750332	279
T12	CP022774_00696	96.45% ISRso14_aa2	66.91% CP022774_02438	IS3 ssgr IS407	743147	742674	474
T12	CP022774_00707	100% ISRso12_aa2	75% CP022774_02438	IS3 ssgr IS407	755068	754364	705
T12	CP022774_01486	100% ISRso12_aa1	88.63% CP022774_02439	IS3 ssgr IS407	1581123	1581389	267
T12	CP022774_01487	94.81% ISRso12_aa2	94.81% CP022774_00707	IS3 ssgr IS407	1581520	1582050	531
T12	CP022774_02438	97.33% ISRso16_aa2	75% CP022774_00707	IS3 ssgr IS407	2635835	2635044	792
T12	CP022774_02439	98.86% ISRso16_aa1	88.63% CP022774_01486	IS3 ssgr IS407	2636185	2635919	267

T12	CP022774_02636	74.71% ISAtu5_aa1	60.49% CP022774_02439	IS3 ssgr IS407	2842027	2842248	222
T12	CP022774_00708	99.63% ISRso1_aa1	96% CP022774_02197	IS5	756061	755237	825
T12	CP022774_02194	94.66% ISRso1_aa1	97.33% CP022774_02197	IS5	2384163	2383936	228
T12	CP022774_02197	96% ISRso1_aa1	97.33% CP022774_02194	IS5	2387395	2387622	228
T12	CP022774_03040	56.77% IS1421_aa3	No hit	IS5 ssgr IS427	3261354	3260728	627
T12	CP022774_02437	80.30% ISCARN25_aa1	81.25% CP022774_02646	IS630	2634373	2634606	234
T12	CP022774_02646	84.21% ISCARN25_aa1	81.25% CP022774_02437	IS630	2858964	2859218	255
T12	CP022774_00292	47.33% ISShvi3_aa1	41.36% CP022774_02180	IS91	310713	309622	1.092
T12	CP022774_00826	47.15% ISMno23_aa1	52.59% CP022774_03232	IS91	875712	874786	927
T12	CP022774_00037	40.42% ISKpn25_aa1	44.62% CP022774_02190	ISL3	4409	45535	1446
T12	CP022774_02190	40.29% ISKpn25_aa1	44.62% CP022774_00037	ISL3	2378392	2380011	162
T12	CP022774_00004	46.61% ISPa4_aa1	No hit	ISNCY	8684	6594	2.091
T12	CP022774_01771	71.81% ISKpn21_aa1	No hit	ISNCY ssgr IS1202	1920963	1919944	1.02
T12	CP022774_00193	50.92% ISThsp9_aa1	No hit	Tn3	206212	204872	1.341
T12	CP022774_00620	53.33% TnShfr1_aa1	No hit	Tn3	653501	654712	1.212
T12	CP022774_01693	77.39% ISPa42_aa2	No hit	Tn3	1808880	1808482	399
SL3022	CP023016_00734	74.19% ISCARN16_aa2	No hit	IS21	778782	778498	285
SL3022	CP023016_00333	85.05% ISGau4_aa1	75.86% CP023016_01607	IS3 ssgr IS407	361277	361543	267
SL3022	CP023016_00334	69.59% ISGau4_aa2	51.93% CP023016_02570	IS3 ssgr IS407	361576	362349	774
SL3022	CP023016_00465	97.81% ISRso14_aa2	95.30% CP023016_00727	IS3 ssgr IS407	484438	483611	828
SL3022	CP023016_00466	98.85% ISRso14_aa1	74.11% CP023016_00333	IS3 ssgr IS407	484728	484465	264
SL3022	CP023016_00727	95.32% ISRso14_aa2	95.30% CP023016_00465	IS3 ssgr IS407	771458	770742	717
SL3022	CP023016_01607	100% ISRso12_aa1	89.41% CP023016_02455	IS3 ssgr IS407	1704923	1705189	267
SL3022	CP023016_01608	95.55% ISRso12_aa2	75.73% CP023016_02456	IS3 ssgr IS407	1705320	1705850	531
SL3022	CP023016_02455	94.56% ISRso16_aa1	89.41% CP023016_01607	IS3 ssgr IS407	2596651	2596929	279
SL3022	CP023016_02456	98.35% ISRso16_aa2	97.32% CP023016_02570	IS3 ssgr IS407	2597013	2597588	576
SL3022	CP023016_02570	97.33% ISRso16_aa2	97.32% CP023016_02456	IS3 ssgr IS407	2720981	2720190	792
SL3022	CP023016_02773	74.71% ISAtu5_aa1	68.35% CP023016_00466	IS3 ssgr IS407	2929846	2930067	222
SL3022	CP023016_00754	38.39% ISGur11_aa1	98.16% CP023016_00774	IS481	794435	796357	1.923
SL3022	CP023016_01556	100% IS1421_aa1	100% CP023016_02462	IS5 ssgr IS427	1641665	1641261	405
SL3022	CP023016_02459	100% IS1421_aa1	100% CP023016_02462	IS5 ssgr IS427	2600780	2601163	384
SL3022	CP023016_02462	100% IS1421_aa1	100% CP023016_01556	IS5 ssgr IS427	2604441	2604845	405
SL3022	CP023016_02569	80.30% ISCARN25_aa1	No hit	IS630	2719468	2719701	234
SL3022	CP023016_00462	44.36% ISShvi3_aa1	No hit	IS91	479846	480886	1.041

SL3022	CP023016_00876	47.15% ISMno23_aa1	52.92% CP023016_03328	IS91	923734	922808	927
SL3022	CP023016_02461	48.40% ISShvi3_aa1	46.45% CP023016_03328	IS91	2604260	2603667	594
SL3022	CP023016_03328	48.75% ISWz1_aa1	53.09% CP023016_00876	IS91	3512231	3513220	990
SL3022	CP023016_00032	40.80% ISSm4_aa1	No hit	ISL3	4126	42705	1.446
SL3022	CP023016_01884	70.30% ISKpn21_aa1	90.18% CP023016_01885	ISNCY ssgr IS1202	2013186	2012182	1.005
SL3022	CP023016_01885	71.39% ISKpn21_aa1	90.18% CP023016_01884	ISNCY ssgr IS1202	2014812	2013340	1.473
SL3022	CP023016_00187	51.95% ISMpo10_aa3	No hit	Tn3	201106	200342	765
SL3022	CP023016_00649	53.33% TnShfr1_aa1	No hit	Tn3	680999	68221	1.212
SL2064	CP022798_00253	49.48% ISHvo5_aa1	No hit	IS1595 ssgr ISH4	270346	271134	789
SL2064	CP022798_00747	70.76% ISPst3_aa2	No hit	IS21	79925	798966	285
SL2064	CP022798_01092	92.85% ISXca2_aa2	55.76% CP022798_02492	IS3 ssgr IS407	1174419	1173904	516
SL2064	CP022798_02492	97.33% ISRso16_aa2	55.76% CP022798_01092	IS3 ssgr IS407	2706612	2705821	792
SL2064	CP022798_02689	72.41% ISAtu5_aa1	70% CP022798_02494	IS3 ssgr IS407	2916430	2916690	261
SL2064	CP022798_03108	56.77% IS1421_aa3	No hit	IS5 ssgr IS427	3352140	3351514	627
SL2064	CP022798_00248	85.93% ISAau3_aa1	No hit	IS5 ssgr IS5	264171	26444	270
SL2064	CP022798_02491	80.30% ISCARN25_aa1	79.10% CP022798_02701	IS630	2705099	2705332	234
SL2064	CP022798_02701	84.21% ISCARN25_aa1	79.10% CP022798_02491	IS630	2933390	2933644	255
SL2064	CP022798_00843	47.16% ISTha3_aa2	52.59% CP022798_03267	IS91	898995	898069	927
SL2064	CP022798_03267	48.75% ISWz1_aa1	52.76% CP022798_00843	IS91	3531169	3532158	990
SL2064	CP022798_00040	40.71% ISKpn25_aa1	50.76% CP022798_01084	ISL3	44434	45879	1.446
SL2064	CP022798_01084	42.36% ISSm4_aa1	50.76% CP022798_00040	ISL3	1164432	1165889	1.458
SL2064	CP022798_01852	55.89% ISKpn21_aa1	No hit	ISNCY ssgr IS1202	2009089	2008598	492
SL2064	CP022798_00189	61.81% ISMpo10_aa3	94.65% CP022798_00261	Tn3	20169	200302	1.389
SL2064	CP022798_00261	53.08% ISMpo10_aa3	94.65% CP022798_00189	Tn3	278862	277486	1.377
SL2064	CP022798_00677	53.33% TnShfr1_aa1	No hit	Tn3	718869	72008	1.212
SL2312	IS_2596d515_00694	69.23% ISPst3_aa2	No hit	IS21	750677	750393	285
SL2312	IS_2596d515_00686	96.45% ISRso14_aa2	66.91% IS_2596d515_02404	IS3 ssgr IS407	743211	742738	474
SL2312	IS_2596d515_00697	100% ISRso12_aa2	75% IS_2596d515_02404	IS3 ssgr IS407	755136	754432	705
SL2312	IS_2596d515_01459	100% ISRso12_aa1	88.63% IS_2596d515_02405	IS3 ssgr IS407	1581258	1581524	267
SL2312	IS_2596d515_01460	94.81% ISRso12_aa2	94.81% IS_2596d515_00697	IS3 ssgr IS407	1581655	1582185	531
SL2312	IS_2596d515_02404	97.33% ISRso16_aa2	75% IS_2596d515_00697	IS3 ssgr IS407	2636009	2635218	792
SL2312	IS_2596d515_02405	98.86% ISRso16_aa1	88.63% IS_2596d515_01459	IS3 ssgr IS407	2636359	2636093	267
SL2312	IS_2596d515_02597	74.71% ISAtu5_aa1	60.49% IS_2596d515_02405	IS3 ssgr IS407	2842216	2842437	222

SL2312	IS_2596d515_00698	99.63% ISRso1_aa1	96% IS_2596d515_02169	IS5	756129	755305	825
SL2312	IS_2596d515_02166	94.66% ISRso1_aa1	97.33% IS_2596d515_02169	IS5	2384321	2384094	228
SL2312	IS_2596d515_02169	96% ISRso1_aa1	97.33% IS_2596d515_02166	IS5	2387553	2387780	228
SL2312	IS_2596d515_02994	56.77% IS1421_aa3	No hit	IS5 ssgr IS427	3261566	3260940	627
SL2312	IS_2596d515_02403	80.30% ISCARN25_aa1	81.25% IS_2596d515_02608	IS630	2634547	2634780	234
SL2312	IS_2596d515_02608	84.21% ISCARN25_aa1	81.25% IS_2596d515_02403	IS630	2859154	2859408	255
SL2312	IS_2596d515_00287	47.33% ISShvi3_aa1	41.36% IS_2596d515_02152	IS91	310745	309654	1.092
SL2312	IS_2596d515_00811	47.15% ISMno23_aa1	52.59% IS_2596d515_03176	IS91	875795	874869	927
SL2312	IS_2596d515_03176	48.75% ISWz1_aa1	52.76% IS_2596d515_00811	IS91	3474858	3475847	990
SL2312	IS_2596d515_00037	40.42% ISKpn25_aa1	44.62% IS_2596d515_02162	ISL3	44097	45542	1.446
SL2312	IS_2596d515_02162	40.29% ISKpn25_aa1	44.62% IS_2596d515_00037	ISL3	2378550	2380169	1.62
SL2312	IS_2596d515_00005	46.61% ISPa4_aa1	No hit	ISNCY	8685	6595	2.091
SL2312	IS_2596d515_01741	71.81% ISKpn21_aa1	No hit	ISNCY ssgr IS1202	1921121	1920102	1.02
SL2312	IS_2596d515_00189	50.92% ISThsp9_aa1	No hit	Tn3	206233	204893	1.341
SL2312	IS_2596d515_00615	53.33% TnShfr1_aa1	No hit	Tn3	653556	654767	1.212
SL2312	IS_2596d515_01666	77.39% ISPa42_aa2	No hit	Tn3	1809028	1808630	399
T101	CP022758_01405	94.04% ISRso8_aa1	No hit	IS3	1793295	1792990	306
T101	CP022758_01402	72.46% ISDet2_aa2	No hit	IS3 ssgr IS407	1791626	1791856	231
T101	CP022758_01403	83.67% ISDet2_aa2	No hit	IS3 ssgr IS407	1791871	1792212	342
T101	CP022758_00362	36.11% ISMva2_aa1	No hit	IS481	447269	44502	2.25
T101	CP022758_01184	90.16% IS1421_aa1	No hit	IS5 ssgr IS427	1545903	1545715	189
T101	CP022758_01393	77.41% ISNGR9_aa2	No hit	IS5 ssgr IS427	1780482	1780105	378
T101	CP022758_00038	63.71% IS1405_aa1	No hit	IS5 ssgr IS5	46547	46945	399
T101	CP022758_01398	92.01% ISRso9_aa1	No hit	IS5 ssgr IS5	1784869	1785699	831
T101	CP022758_01430	82.29% ISBmu20_aa1	No hit	IS5 ssgr IS5	1825689	1825324	366
T101	CP022758_00866	43.24% ISShvi3_aa1	95.16% CP022758_00812	IS91	1127374	1125674	1.701
T101	CP022758_00855	72% ISKpn21_aa1	No hit	ISNCY ssgr IS1202	1110567	1109530	1.038
T101	CP022758_00933	60.56% ISKpn21_aa1	No hit	ISNCY ssgr IS1202	1217736	1218074	339
T101	CP022758_01396	54.43% ISMpo10_aa3	No hit	Tn3	1783735	1782356	1.38
T82	CP022763_00694	69.23% ISPst3_aa2	No hit	IS21	750677	750393	285
T82	CP022763_00687	96.45% ISRso14_aa2	66.91% CP022763_02395	IS3 ssgr IS407	74321	742737	474
T82	CP022763_00697	100% ISRso12_aa2	75% CP022763_02395	IS3 ssgr IS407	755136	754432	705
T82	CP022763_01462	100% ISRso12_aa1	88.63% CP022763_02396	IS3 ssgr IS407	1581219	1581485	267

T82	CP022763_01463	94.81% ISRso12_aa2	94.81% CP022763_00697	IS3 ssgr IS407	1581616	1582146	531
T82	CP022763_02395	97.33% ISRso16_aa2	75% CP022763_00697	IS3 ssgr IS407	2636009	2635218	792
T82	CP022763_02396	98.86% ISRso16_aa1	88.63% CP022763_01462	IS3 ssgr IS407	2636359	2636093	267
T82	CP022763_02587	74.71% ISAtu5_aa1	60.49% CP022763_02396	IS3 ssgr IS407	2842219	2842440	222
T82	CP022763_00698	99.63% ISRso1_aa1	96% CP022763_02164	IS5	756129	755305	825
T82	CP022763_02161	94.66% ISRso1_aa1	97.33% CP022763_02164	IS5	2384313	2384086	228
T82	CP022763_02164	96% ISRso1_aa1	97.33% CP022763_02161	IS5	2387545	2387772	228
T82	CP022763_02982	56.77% IS1421_aa3	No hit	IS5 ssgr IS427	3261564	3260938	627
T82	CP022763_02394	80.30% ISCARN25_aa1	81.25% CP022763_02597	IS630	2634547	2634780	234
T82	CP022763_02597	84.21% ISCARN25_aa1	81.25% CP022763_02394	IS630	2859157	2859411	255
T82	CP022763_00287	47.33% ISShvi3_aa1	41.36% CP022763_02147	IS91	310746	309655	1.092
T82	CP022763_00811	47.15% ISMno23_aa1	52.59% CP022763_03164	IS91	875795	874869	927
T82	CP022763_03164	48.75% ISWz1_aa1	52.76% CP022763_00811	IS91	3474857	3475846	990
T82	CP022763_00037	40.42% ISKpn25_aa1	44.62% CP022763_02157	ISL3	44097	45542	1.446
T82	CP022763_02157	40.29% ISKpn25_aa1	44.62% CP022763_00037	ISL3	2378542	2380161	1.62
T82	CP022763_00005	46.61% ISPa4_aa1	No hit	ISNCY	8685	6595	2.091
T82	CP022763_01743	71.81% ISKpn21_aa1	No hit	ISNCY ssgr IS1202	1921083	1920064	1.02
T82	CP022763_00189	50.92% ISThsp9_aa1	No hit	Tn3	206233	204893	1.341
T82	CP022763_00615	53.33% TnShfr1_aa1	No hit	Tn3	653555	654766	1.212
T82	CP022763_01668	77.39% ISPa42_aa2	No hit	Tn3	1808990	1808592	399
T95	CP022761_00253	49.48% ISHvo5_aa1	No hit	IS1595 ssgr ISH4	270347	271135	789
T95	CP022761_00747	70.76% ISPst3_aa2	No hit	IS21	799251	798967	285
T95	CP022761_01092	92.85% ISXca2_aa2	55.76% CP022761_02484	IS3 ssgr IS407	1174420	1173905	516
T95	CP022761_02484	97.33% ISRso16_aa2	55.76% CP022761_01092	IS3 ssgr IS407	2707500	2706709	792
T95	CP022761_02681	72.41% ISAtu5_aa1	70% CP022761_02486	IS3 ssgr IS407	2917318	2917578	261
T95	CP022761_03099	56.77% IS1421_aa3	No hit	IS5 ssgr IS427	3353028	3352402	627
T95	CP022761_00248	85.93% ISAau3_aa1	No hit	IS5 ssgr IS5	264172	264441	270
T95	CP022761_02483	80.30% ISCARN25_aa1	79.10% CP022761_02692	IS630	2705987	2706220	234
T95	CP022761_02692	84.21% ISCARN25_aa1	79.10% CP022761_02483	IS630	2934278	2934532	255
T95	CP022761_00843	47.16% ISTha3_aa2	52.59% CP022761_03258	IS91	898996	89807	927
T95	CP022761_03258	48.75% ISWz1_aa1	52.76% CP022761_00843	IS91	3532056	3533045	990
T95	CP022761_00040	40.71% ISKpn25_aa1	50.76% CP022761_01084	ISL3	44434	45879	1.446
T95	CP022761_01084	42.36% ISSm4_aa1	50.76% CP022761_00040	ISL3	1164433	1165890	1.458
T95	CP022761_01843	55.89% ISKpn21_aa1	No hit	ISNCY ssgr IS1202	2009977	2009486	492

T95	CP022761_00189	61.81% ISMpo10_aa3	94.65% CP022761_00261	Tn3	201691	200303	1.389
T95	CP022761_00261	53.08% ISMpo10_aa3	94.65% CP022761_00189	Tn3	278863	277487	1.377
T95	CP022761_00677	53.33% TnShfr1_aa1	No hit	Tn3	718869	72008	1.212
12J	IS_75882f3f_01025	79.54% ISPsy11_aa1	100% IS_75882f3f_03646	IS3 ssgr IS3	1079047	1079445	399
12J	IS_75882f3f_01026	85.23% ISBcen7_aa2	100% IS_75882f3f_03647	IS3 ssgr IS3	1079448	1080275	828
12J	IS_75882f3f_01130	79.54% ISPsy11_aa1	100% IS_75882f3f_03646	IS3 ssgr IS3	1196718	1197116	399
12J	IS_75882f3f_01131	85.23% ISBcen7_aa2	100% IS_75882f3f_03647	IS3 ssgr IS3	1197119	1197946	828
12J	IS_75882f3f_02688	85.23% ISBcen7_aa2	100% IS_75882f3f_02803	IS3 ssgr IS3	2785799	2784972	828
12J	IS_75882f3f_02689	79.54% ISPsy11_aa1	100% IS_75882f3f_03646	IS3 ssgr IS3	2786200	2785802	399
12J	IS_75882f3f_02803	85.23% ISBcen7_aa2	100% IS_75882f3f_02688	IS3 ssgr IS3	2899546	2898719	828
12J	IS_75882f3f_02804	79.54% ISPsy11_aa1	100% IS_75882f3f_03646	IS3 ssgr IS3	2899947	2899549	399
12J	IS_75882f3f_03646	79.54% ISPsy11_aa1	100% IS_75882f3f_02804	IS3 ssgr IS3	3788563	3788961	399
12J	IS_75882f3f_03647	85.23% ISBcen7_aa2	100% IS_75882f3f_01131	IS3 ssgr IS3	3788964	3789791	828
12J	IS_75882f3f_02534	80.45% ISPmar2_aa3	No hit	IS3 ssgr IS407	2636791	2637204	414
12J	IS_75882f3f_00007	100% ISRme15_aa2	100% IS_75882f3f_01826	IS3 ssgr IS51	11667	10906	762
12J	IS_75882f3f_00008	100% ISRme15_aa1	100% IS_75882f3f_01827	IS3 ssgr IS51	12146	11817	330
12J	IS_75882f3f_01149	95.32% IS401_aa1	86.79% IS_75882f3f_01827	IS3 ssgr IS51	1215931	1216254	324
12J	IS_75882f3f_01150	97.09% IS401_aa2	85.12% IS_75882f3f_01826	IS3 ssgr IS51	1216251	1216802	552
12J	IS_75882f3f_01826	100% ISRme15_aa2	100% IS_75882f3f_00007	IS3 ssgr IS51	1918623	1917862	762
12J	IS_75882f3f_01827	100% ISRme15_aa1	100% IS_75882f3f_00008	IS3 ssgr IS51	1919102	1918773	330
12J	IS_75882f3f_02073	90.42% ISAtu4_aa1	No hit	IS3 ssgr IS51	2179444	2179151	294
12J	IS_75882f3f_00279	87.25% ISAzo23_aa1	100% IS_75882f3f_01482	IS5 ssgr IS1031	295113	295919	807
12J	IS_75882f3f_00282	87.25% ISAzo23_aa1	100% IS_75882f3f_01482	IS5 ssgr IS1031	298828	299634	807
12J	IS_75882f3f_00839	87.25% ISAzo23_aa1	100% IS_75882f3f_01482	IS5 ssgr IS1031	889798	890604	807
12J	IS_75882f3f_01482	87.25% ISAzo23_aa1	100% IS_75882f3f_00839	IS5 ssgr IS1031	1565260	1564454	807
12J	IS_75882f3f_01162	97.98% ISStma10_aa1	100% IS_75882f3f_02778	IS630	1235138	1236181	1.044
12J	IS_75882f3f_01500	97.98% ISStma10_aa1	100% IS_75882f3f_02778	IS630	1581723	1580680	1.044
12J	IS_75882f3f_01758	97.98% ISStma10_aa1	100% IS_75882f3f_02778	IS630	1851058	1852101	1.044
12J	IS_75882f3f_02651	57.89% ISWpi10_aa2	No hit	IS630	2750455	2750177	279
12J	IS_75882f3f_02778	97.98% ISStma10_aa1	100% IS_75882f3f_01758	IS630	2873504	2874547	1.044
12J	IS_75882f3f_01176	64.94% ISBcen19_aa1	100% IS_75882f3f_03347	IS66	1257464	1257751	288
12J	IS_75882f3f_01177	77.08% ISSal1_aa2	100% IS_75882f3f_03346	IS66	1257793	1258083	291
12J	IS_75882f3f_01178	80.89% ISBcen19_aa3	100% IS_75882f3f_03345	IS66	1258146	1259675	1.53
12J	IS_75882f3f_01494	80.89% ISBcen19_aa3	100% IS_75882f3f_03345	IS66	1577269	1575740	1.53

12J	IS_75882f3f_01495	77.08% ISSal1_aa2	100% IS_75882f3f_03346	IS66	1577622	1577332	291
12J	IS_75882f3f_01496	64.94% ISBcen19_aa1	100% IS_75882f3f_03347	IS66	1577951	1577664	288
12J	IS_75882f3f_03345	80.89% ISBcen19_aa3	100% IS_75882f3f_01494	IS66	3472676	3471147	1.53
12J	IS_75882f3f_03346	77.08% ISSal1_aa2	100% IS_75882f3f_01495	IS66	3473029	3472739	291
12J	IS_75882f3f_03347	64.94% ISBcen19_aa1	100% IS_75882f3f_01496	IS66	3473358	3473071	288
12J	IS_75882f3f_01738	41.36% ISShvi3_aa1	55.49% IS_75882f3f_01746	IS91	1829386	1828193	1.194
12J	IS_75882f3f_01746	45.35% ISShvi3_aa1	81.85% IS_75882f3f_01825	IS91	1835612	1833933	1.68
12J	IS_75882f3f_01825	45.19% ISWz1_aa1	81.85% IS_75882f3f_01746	IS91	1917333	1915633	1.701
12J	IS_75882f3f_02838	49.47% ISMno23_aa1	52.59% IS_75882f3f_03728	IS91	2929174	2930118	945
12J	IS_75882f3f_03218	46.04% ISShvi3_aa1	41.57% IS_75882f3f_00458	IS91	3351051	3349996	1.056
12J	IS_75882f3f_03728	48.25% ISWz1_aa1	51.79% IS_75882f3f_02838	IS91	3876531	3877517	987
12J	IS_75882f3f_00004	41.15% ISSm4_aa1	48.98% IS_75882f3f_03195	ISL3	6281	8413	2.133
12J	IS_75882f3f_00446	40.48% ISSm4_aa1	51.19% IS_75882f3f_03195	ISL3	483006	484619	1.614
12J	IS_75882f3f_01907	50.78% ISSm4_aa2	52.31% IS_75882f3f_02616	ISL3	2011099	2012160	1.062
12J	IS_75882f3f_02616	53.31% ISSm4_aa2	51.49% IS_75882f3f_01907	ISL3	2716578	2717714	1.137
12J	IS_75882f3f_03189	38.92% ISKpn25_aa3	42% IS_75882f3f_00443	ISL3	3325497	3322510	2.988
12J	IS_75882f3f_03190	44.79% ISSm4_aa3	No hit	ISL3	3326681	3325497	1.185
12J	IS_75882f3f_03193	45.34% ISKpn25_aa1	68.14% IS_75882f3f_03195	ISL3	3331763	3330252	1.512
12J	IS_75882f3f_03195	45.27% ISKpn25_aa1	68.14% IS_75882f3f_03193	ISL3	3334044	3332548	1.497
12J	IS_75882f3f_01739	58.62% ISKpn21_aa2	No hit	ISNCY ssgr IS1202	1830062	1829421	642
12J	IS_75882f3f_00513	52.74% ISMpo10_aa1	No hit	Tn3	554472	554987	516
12J	IS_75882f3f_00565	54.13% TnShfr1_aa1	97.92% IS_75882f3f_01593	Tn3	603805	605109	1.305
12J	IS_75882f3f_01486	93.16% ISPsy30_aa3	100% IS_75882f3f_01851	Tn3	1570682	1567653	3.03
12J	IS_75882f3f_01487	93.68% ISPsy30_aa2	100% IS_75882f3f_01852	Tn3	1571272	1570679	594
12J	IS_75882f3f_01593	54.13% TnShfr1_aa1	97.92% IS_75882f3f_00565	Tn3	1674338	1675642	1.305
12J	IS_75882f3f_01645	66.22% IS882_aa1	40.02% IS_75882f3f_01736	Tn3	1719504	1722437	2.934
12J	IS_75882f3f_01646	75.87% ISPa43_aa1	No hit	Tn3	1723499	1722537	963
12J	IS_75882f3f_01683	53.14% TnShfr1_aa1	100% IS_75882f3f_02955	Tn3	1767425	1766121	1.305
12J	IS_75882f3f_01736	75.82% ISPa42_aa1	40.02% IS_75882f3f_01645	Tn3	1824700	1827699	3
12J	IS_75882f3f_01765	78% TnShfr1_aa1	49.82% IS_75882f3f_01595	Tn3	1858689	1857739	951
12J	IS_75882f3f_01851	93.16% ISPsy30_aa3	100% IS_75882f3f_01486	Tn3	1944951	1941922	3.03
12J	IS_75882f3f_01852	93.68% ISPsy30_aa2	100% IS_75882f3f_01487	Tn3	1945541	1944948	594
12J	IS_75882f3f_02563	62.98% ISPa38_aa1	61.49% IS_75882f3f_01852	Tn3	2656480	2655890	591
12J	IS_75882f3f_02955	53.14% TnShfr1_aa1	100% IS_75882f3f_01683	Tn3	3054729	3056033	1.305
12J	IS_75882f3f_03382	41.64% TnShfr1_aa1	71.86% IS_75882f3f_00572	Tn3	3514032	3512884	1.149

ATCC49129_1	IS_8802d314_02215	64.70% IS5708_aa1	No hit	IS110 ssgr IS1111	2307733	2308833	1.101
ATCC49129_1	IS_8802d314_02202	78.15% IS401_aa2	No hit	IS3 ssgr IS51	2295504	2295875	372
ATCC49129_1	IS_8802d314_02203	91.17% ISStma17_aa2	54.25% IS_8802d314_02224	IS3 ssgr IS51	2295900	2296409	510
ATCC49129_1	IS_8802d314_02224	90.32% ISMlo4_aa2	54.25% IS_8802d314_02203	IS3 ssgr IS51	2318700	2318287	414
ATCC49129_1	IS_8802d314_02872	45.18% ISGur11_aa1	73.07% IS_8802d314_02867	IS481	2977128	2979275	2.148
ATCC49129_1	IS_8802d314_02124	61.02% ISAzo11_aa1	45.20% IS_8802d314_02200	IS5 ssgr IS5	2230277	2231365	1.089
ATCC49129_1	IS_8802d314_02200	96.27% ISPa54_aa1	74.23% IS_8802d314_02214	IS5 ssgr IS5	2293261	2294238	978
ATCC49129_1	IS_8802d314_02214	88.88% ISBmu20_aa1	74.23% IS_8802d314_02200	IS5 ssgr IS5	2306495	2307388	894
ATCC49129_1	IS_8802d314_02195	75.72% ISBcen19_aa3	83.87% IS_8802d314_00364	IS66	2290993	2289479	1.515
ATCC49129_1	IS_8802d314_02196	76.57% ISPpu19_aa2	No hit	IS66	2291384	2291055	330
ATCC49129_1	IS_8802d314_02197	61.01% ISBcen19_aa1	No hit	IS66	2291749	2291381	369
ATCC49129_1	IS_8802d314_01654	44.50% ISWz1_aa1	47.25% IS_8802d314_02778	IS91	1723567	1725267	1.701
ATCC49129_1	IS_8802d314_02778	46.07% ISTha3_aa2	50% IS_8802d314_03713	IS91	2877428	2878363	936
ATCC49129_1	IS_8802d314_03713	47.05% ISWz1_aa1	50% IS_8802d314_02778	IS91	3853518	3854528	1.011
ATCC49129_1	IS_8802d314_00007	75.09% ISSm4_aa4	No hit	ISL3	12005	8763	3.243
ATCC49129_1	IS_8802d314_00008	49.33% ISKpn25_aa2	No hit	ISL3	13318	12002	1.317
ATCC49129_1	IS_8802d314_00009	75.74% ISKpn25_aa1	No hit	ISL3	1536	13315	2.046
ATCC49129_1	IS_8802d314_03137	53.08% ISSm4_aa2	No hit	ISL3	3257379	3258419	1.041
ATCC49129_1	IS_8802d314_00579	52.27% ISMpo10_aa1	No hit	Tn3	601531	602058	528
ATCC49129_1	IS_8802d314_01638	71.72% ISPa43_aa1	No hit	Tn3	1707140	1706109	1.032
ATCC49129_1	IS_8802d314_01678	62% ISMpo10_aa2	No hit	Tn3	1740230	1740685	456
ATCC49129_1	IS_8802d314_02136	98.25% ISPa38_aa2	No hit	Tn3	2239281	2239922	642
ATCC49129_2	IS_4adf2139_00370	44.88% ISWz1_aa1	43.47% IS_4adf2139_01318	IS91	438322	436619	1.704
ATCC49129_2	IS_4adf2139_00451	83.33% ISDet2_aa2	No hit	IS3 ssgr IS407	520861	520328	534
A2HRMARDI	CP019911_00124	94.31% ISBcen18_aa1	100% CP019911_03307	IS256	138538	137261	1.278
A2HRMARDI	CP019911_00597	94.31% ISBcen18_aa1	100% CP019911_03307	IS256	610446	609169	1.278
A2HRMARDI	CP019911_00603	94.23% ISBcen18_aa1	100% CP019911_03307	IS256	614017	614799	783
A2HRMARDI	CP019911_00748	94.31% ISBcen18_aa1	100% CP019911_03307	IS256	756248	757525	1.278
A2HRMARDI	CP019911_00816	95.83% ISBcen18_aa1	100% CP019911_01604	IS256	832745	833035	291
A2HRMARDI	CP019911_00853	94.31% ISBcen18_aa1	100% CP019911_03307	IS256	880726	879449	1.278
A2HRMARDI	CP019911_00990	94.31% ISBcen18_aa1	100% CP019911_03307	IS256	1021073	1022350	1.278
A2HRMARDI	CP019911_01020	94.31% ISBcen18_aa1	100% CP019911_03307	IS256	1055624	1054347	1.278

A2HRMARDI	CP019911_01058	94.31% ISBcen18_aa1	100% CP019911_03307	IS256	1089194	1087917	1.278
A2HRMARDI	CP019911_01060	94.31% ISBcen18_aa1	100% CP019911_03307	IS256	1089888	1091165	1.278
A2HRMARDI	CP019911_01247	94.31% ISBcen18_aa1	100% CP019911_03307	IS256	1296362	1295085	1.278
A2HRMARDI	CP019911_01259	94.31% ISBcen18_aa1	100% CP019911_03307	IS256	1307129	1308406	1.278
A2HRMARDI	CP019911_01282	94.31% ISBcen18_aa1	100% CP019911_03307	IS256	1336609	1335332	1.278
A2HRMARDI	CP019911_01399	94.31% ISBcen18_aa1	100% CP019911_03307	IS256	1457777	1456500	1.278
A2HRMARDI	CP019911_01444	94.31% ISBcen18_aa1	100% CP019911_03307	IS256	1526153	1527430	1.278
A2HRMARDI	CP019911_01458	94.31% ISBcen18_aa1	100% CP019911_03307	IS256	1540240	1541517	1.278
A2HRMARDI	CP019911_01516	94.31% ISBcen18_aa1	100% CP019911_03307	IS256	1603298	1604575	1.278
A2HRMARDI	CP019911_01536	94.31% ISBcen18_aa1	100% CP019911_03307	IS256	1622606	1623883	1.278
A2HRMARDI	CP019911_01604	93.83% ISBcen18_aa1	99.52% CP019911_03307	IS256	1698482	1699759	1.278
A2HRMARDI	CP019911_01631	94.31% ISBcen18_aa1	100% CP019911_03307	IS256	1727605	1726328	1.278
A2HRMARDI	CP019911_01786	94.31% ISBcen18_aa1	100% CP019911_03307	IS256	1873865	1875142	1.278
A2HRMARDI	CP019911_01787	94.31% ISBcen18_aa1	100% CP019911_03307	IS256	1876684	1875407	1.278
A2HRMARDI	CP019911_01831	94.31% ISBcen18_aa1	100% CP019911_03307	IS256	1923753	1925030	1.278
A2HRMARDI	CP019911_01949	94.31% ISBcen18_aa1	100% CP019911_03307	IS256	2043254	2044531	1.278
A2HRMARDI	CP019911_02073	94.31% ISBcen18_aa1	100% CP019911_03307	IS256	2183050	2181773	1.278
A2HRMARDI	CP019911_02101	94.31% ISBcen18_aa1	100% CP019911_03307	IS256	2216521	2217798	1.278
A2HRMARDI	CP019911_02161	94.31% ISBcen18_aa1	100% CP019911_03307	IS256	2286379	2287656	1.278
A2HRMARDI	CP019911_02166	94.31% ISBcen18_aa1	100% CP019911_03307	IS256	2291009	2289732	1.278
A2HRMARDI	CP019911_02248	94.31% ISBcen18_aa1	100% CP019911_03307	IS256	2373269	2374546	1.278
A2HRMARDI	CP019911_02399	94.31% ISBcen18_aa1	100% CP019911_03307	IS256	2518482	2519759	1.278
A2HRMARDI	CP019911_02573	94.31% ISBcen18_aa1	100% CP019911_03307	IS256	2688945	2687668	1.278
A2HRMARDI	CP019911_02574	94.31% ISBcen18_aa1	100% CP019911_03307	IS256	2689515	2690792	1.278
A2HRMARDI	CP019911_02801	94.31% ISBcen18_aa1	100% CP019911_03307	IS256	2944599	2945876	1.278
A2HRMARDI	CP019911_02933	94.31% ISBcen18_aa1	100% CP019911_03307	IS256	3152147	3150870	1.278
A2HRMARDI	CP019911_02934	94.73% ISBcen18_aa1	100% CP019911_03307	IS256	3152202	3153344	1.143
A2HRMARDI	CP019911_03016	94.31% ISBcen18_aa1	100% CP019911_03307	IS256	3251351	3250074	1.278
A2HRMARDI	CP019911_03050	94.31% ISBcen18_aa1	100% CP019911_03307	IS256	3289537	3290814	1.278
A2HRMARDI	CP019911_03085	94.31% ISBcen18_aa1	100% CP019911_03307	IS256	3328894	3327617	1.278
A2HRMARDI	CP019911_03113	94.04% ISBcen18_aa1	100% CP019911_03307	IS256	3363669	3362710	960
A2HRMARDI	CP019911_03114	94.31% ISBcen18_aa1	100% CP019911_03307	IS256	3363724	3365001	1.278
A2HRMARDI	CP019911_03183	94.31% ISBcen18_aa1	100% CP019911_03307	IS256	3429155	3430432	1.278
A2HRMARDI	CP019911_03271	94.31% ISBcen18_aa1	100% CP019911_03307	IS256	3557255	3558532	1.278
A2HRMARDI	CP019911_03307	94.31% ISBcen18_aa1	100% CP019911_03271	IS256	3599205	3597928	1.278

A2HRMARDI	CP019911_00122	99.18% ISRso10_aa1	100% CP019911_03309	IS3 ssgr IS2	136133	136522	390
A2HRMARDI	CP019911_00123	96.12% ISRso10_aa2	98.06% CP019911_02575	IS3 ssgr IS2	136765	137253	489
A2HRMARDI	CP019911_00596	96.82% ISRso10_aa2	100% CP019911_03310	IS3 ssgr IS2	609021	60883	192
A2HRMARDI	CP019911_00683	97.5% ISRso10_aa2	100% CP019911_02575	IS3 ssgr IS2	690518	689916	603
A2HRMARDI	CP019911_00684	99.18% ISRso10_aa1	100% CP019911_03309	IS3 ssgr IS2	69115	690761	390
A2HRMARDI	CP019911_00818	99.18% ISRso10_aa1	100% CP019911_03309	IS3 ssgr IS2	833937	834326	390
A2HRMARDI	CP019911_00819	98% ISRso10_aa2	100% CP019911_03310	IS3 ssgr IS2	834569	835171	603
A2HRMARDI	CP019911_01056	99.18% ISRso10_aa1	100% CP019911_03309	IS3 ssgr IS2	1086730	1087119	390
A2HRMARDI	CP019911_01057	97.66% ISRso10_aa3	100% CP019911_02162	IS3 ssgr IS2	1087362	1088096	735
A2HRMARDI	CP019911_01456	99.18% ISRso10_aa1	100% CP019911_03309	IS3 ssgr IS2	1539104	1539493	390
A2HRMARDI	CP019911_01457	96.79% ISRso10_aa2	97.51% CP019911_00123	IS3 ssgr IS2	1539736	1540227	492
A2HRMARDI	CP019911_02162	97.66% ISRso10_aa3	100% CP019911_01057	IS3 ssgr IS2	2288211	2287477	735
A2HRMARDI	CP019911_02163	99.18% ISRso10_aa1	100% CP019911_03309	IS3 ssgr IS2	2288843	2288454	390
A2HRMARDI	CP019911_02249	90.72% ISRso10_aa2	99.59% CP019911_02162	IS3 ssgr IS2	2375200	2374367	834
A2HRMARDI	CP019911_02407	99.18% ISRso10_aa1	100% CP019911_03309	IS3 ssgr IS2	2523230	2523619	390
A2HRMARDI	CP019911_02408	97.5% ISRso10_aa2	100% CP019911_02575	IS3 ssgr IS2	2523862	2524464	603
A2HRMARDI	CP019911_02575	97.5% ISRso10_aa2	100% CP019911_02408	IS3 ssgr IS2	2691646	2691044	603
A2HRMARDI	CP019911_02576	99.18% ISRso10_aa1	100% CP019911_03309	IS3 ssgr IS2	2692278	2691889	390
A2HRMARDI	CP019911_02803	99.18% ISRso10_aa1	100% CP019911_03309	IS3 ssgr IS2	2946195	2946584	390
A2HRMARDI	CP019911_02804	98% ISRso10_aa2	100% CP019911_03310	IS3 ssgr IS2	2946827	2947429	603
A2HRMARDI	CP019911_03051	98.14% ISRso10_aa2	100% CP019911_02162	IS3 ssgr IS2	3291344	3290661	684
A2HRMARDI	CP019911_03052	99.18% ISRso10_aa1	100% CP019911_03309	IS3 ssgr IS2	3291976	3291587	390
A2HRMARDI	CP019911_03309	99.18% ISRso10_aa1	100% CP019911_03052	IS3 ssgr IS2	3600140	3600529	390
A2HRMARDI	CP019911_03310	98% ISRso10_aa2	100% CP019911_02804	IS3 ssgr IS2	3600772	3601374	603
A2HRMARDI	CP019911_00601	98.86% ISRso16_aa1	100% CP019911_03270	IS3 ssgr IS407	612849	613115	267
A2HRMARDI	CP019911_00602	96.95% ISRso16_aa2	100% CP019911_03269	IS3 ssgr IS407	613199	61399	792
A2HRMARDI	CP019911_00814	98.86% ISRso16_aa1	100% CP019911_03270	IS3 ssgr IS407	831571	831837	267
A2HRMARDI	CP019911_00815	96.95% ISRso16_aa2	100% CP019911_03269	IS3 ssgr IS407	831921	832712	792
A2HRMARDI	CP019911_01832	98.86% ISRso16_aa1	100% CP019911_03270	IS3 ssgr IS407	1925562	1925296	267
A2HRMARDI	CP019911_03269	96.95% ISRso16_aa2	100% CP019911_00815	IS3 ssgr IS407	3556649	3555858	792
A2HRMARDI	CP019911_03270	98.86% ISRso16_aa1	100% CP019911_01832	IS3 ssgr IS407	3556999	3556733	267
A2HRMARDI	CP019911_02298	38.76% ISGur11_aa1	No hit	IS481	2415031	2413349	1.683
A2HRMARDI	CP019911_02899	75.80% ISCARN14_aa1	No hit	IS5 ssgr IS1031	3112482	3113633	1.152
A2HRMARDI	CP019911_00807	95.41% ISRso9_aa1	100% CP019911_03186	IS5 ssgr IS5	824239	822917	1.323
A2HRMARDI	CP019911_01079	95.41% ISRso9_aa1	100% CP019911_03186	IS5 ssgr IS5	1113088	1114410	1.323

A2HRMARDI	CP019911_01204	95.41% ISRso9_aa1	100% CP019911_03193	IS5 ssgr IS5	1250324	1251646	1.323
A2HRMARDI	CP019911_01252	95.41% ISRso9_aa1	100% CP019911_03186	IS5 ssgr IS5	1300256	1298934	1.323
A2HRMARDI	CP019911_01361	95.41% ISRso9_aa1	100% CP019911_03186	IS5 ssgr IS5	1419841	1418519	1.323
A2HRMARDI	CP019911_01422	95.41% ISRso9_aa1	100% CP019911_03186	IS5 ssgr IS5	1496805	1495483	1.323
A2HRMARDI	CP019911_01529	95.41% ISRso9_aa1	100% CP019911_03193	IS5 ssgr IS5	1614320	1612998	1.323
A2HRMARDI	CP019911_01830	95.41% ISRso9_aa1	99.77% CP019911_03186	IS5 ssgr IS5	1922391	1923740	1.35
A2HRMARDI	CP019911_01951	95.41% ISRso9_aa1	100% CP019911_03186	IS5 ssgr IS5	2046643	2045321	1.323
A2HRMARDI	CP019911_02088	95.41% ISRso9_aa1	100% CP019911_03186	IS5 ssgr IS5	2203829	2205151	1.323
A2HRMARDI	CP019911_02090	95.41% ISRso9_aa1	100% CP019911_03186	IS5 ssgr IS5	2207312	2205990	1.323
A2HRMARDI	CP019911_02150	95.41% ISRso9_aa1	100% CP019911_03186	IS5 ssgr IS5	2259567	2260889	1.323
A2HRMARDI	CP019911_02199	95.40% ISRso9_aa1	100% CP019911_03193	IS5 ssgr IS5	2321217	2320939	279
A2HRMARDI	CP019911_02200	95.29% ISRso9_aa1	99.70% CP019911_03272	IS5 ssgr IS5	2322260	2321214	1.047
A2HRMARDI	CP019911_02558	95.41% ISRso9_aa1	100% CP019911_03186	IS5 ssgr IS5	2673320	2674642	1.323
A2HRMARDI	CP019911_02580	95.41% ISRso9_aa1	100% CP019911_03193	IS5 ssgr IS5	2699768	2698446	1.323
A2HRMARDI	CP019911_02805	95.41% ISRso9_aa1	100% CP019911_03186	IS5 ssgr IS5	2949890	2948568	1.323
A2HRMARDI	CP019911_03048	95.41% ISRso9_aa1	100% CP019911_03186	IS5 ssgr IS5	3287517	3286195	1.323
A2HRMARDI	CP019911_03186	95.41% ISRso9_aa1	100% CP019911_03048	IS5 ssgr IS5	3433614	3434936	1.323
A2HRMARDI	CP019911_03193	95.41% ISRso9_aa1	100% CP019911_02580	IS5 ssgr IS5	3444014	3445336	1.323
A2HRMARDI	CP019911_03272	96.28% ISRso9_aa1	100% CP019911_03186	IS5 ssgr IS5	3559788	3558379	1.41
A2HRMARDI	CP019911_00121	60.14% ISBmu8_aa1	No hit	IS630	135252	136079	828
A2HRMARDI	CP019911_00125	68.42% ISRso5_aa1	No hit	IS630	138798	139025	228
A2HRMARDI	CP019911_00812	90.26% ISCARN25_aa1	100% CP019911_03257	IS630	829127	830146	1.02
A2HRMARDI	CP019911_00813	91.85% ISCARN25_aa1	100% CP019911_01833	IS630	831444	830632	813
A2HRMARDI	CP019911_00888	90.26% ISCARN25_aa1	100% CP019911_03257	IS630	917735	916716	1.02
A2HRMARDI	CP019911_01097	90.26% ISCARN25_aa1	100% CP019911_03257	IS630	1145026	1146045	1.02
A2HRMARDI	CP019911_01206	90.26% ISCARN25_aa1	100% CP019911_03257	IS630	1253685	1252666	1.02
A2HRMARDI	CP019911_01421	92.36% ISCARN25_aa1	100% CP019911_03305	IS630	1495316	1494882	435
A2HRMARDI	CP019911_01435	90.26% ISCARN25_aa1	100% CP019911_03257	IS630	1510092	1511111	1.02
A2HRMARDI	CP019911_01447	90.26% ISCARN25_aa1	100% CP019911_03257	IS630	1530448	1531467	1.02
A2HRMARDI	CP019911_01476	90.26% ISCARN25_aa1	100% CP019911_03257	IS630	1557684	1558703	1.02
A2HRMARDI	CP019911_01492	90.26% ISCARN25_aa1	100% CP019911_03257	IS630	1575521	1574502	1.02
A2HRMARDI	CP019911_01514	90.26% ISCARN25_aa1	100% CP019911_03257	IS630	1600653	1601672	1.02
A2HRMARDI	CP019911_01515	90.26% ISCARN25_aa1	100% CP019911_03257	IS630	1603101	1602082	1.02
A2HRMARDI	CP019911_01537	83.78% ISCARN25_aa1	99.62% CP019911_03257	IS630	1624725	1623730	996
A2HRMARDI	CP019911_01539	90.26% ISCARN25_aa1	100% CP019911_03257	IS630	1625014	1626033	1.02

A2HRMARDI	CP019911_01569	76.99% ISCARN25_aa1	85.25% CP019911_02871	IS630	1656491	1655622	870
A2HRMARDI	CP019911_01591	90.26% ISCARN25_aa1	100% CP019911_03257	IS630	1678754	1679773	1.02
A2HRMARDI	CP019911_01833	91.85% ISCARN25_aa1	100% CP019911_00813	IS630	1925689	1926501	813
A2HRMARDI	CP019911_02089	91.82% ISCARN25_aa1	100% CP019911_01833	IS630	2205815	2205189	627
A2HRMARDI	CP019911_02871	89.97% ISCARN25_aa1	99.70% CP019911_03257	IS630	3017125	3018144	1.02
A2HRMARDI	CP019911_02908	90.26% ISCARN25_aa1	100% CP019911_03257	IS630	3121832	3120813	1.02
A2HRMARDI	CP019911_02931	92.36% ISCARN25_aa1	100% CP019911_03305	IS630	3150604	3150170	435
A2HRMARDI	CP019911_02975	90.26% ISCARN25_aa1	100% CP019911_03257	IS630	3199038	3200057	1.02
A2HRMARDI	CP019911_03049	90.26% ISCARN25_aa1	100% CP019911_03257	IS630	3287887	3288906	1.02
A2HRMARDI	CP019911_03057	90.26% ISCARN25_aa1	100% CP019911_03257	IS630	3298591	3297572	1.02
A2HRMARDI	CP019911_03257	90.26% ISCARN25_aa1	100% CP019911_03057	IS630	3533618	3534637	1.02
A2HRMARDI	CP019911_03305	92.36% ISCARN25_aa1	100% CP019911_02931	IS630	3597662	3597228	435
A2HRMARDI	CP019911_00275	45% ISTha3_aa2	41.71% CP019911_01168	IS91	296599	297684	1.086
A2HRMARDI	CP019911_00909	47.36% ISMno23_aa1	52.59% CP019911_02015	IS91	936486	937412	927
A2HRMARDI	CP019911_02015	48.75% ISWz1_aa1	52.59% CP019911_00909	IS91	2116016	2117005	990
A2HRMARDI	CP019911_03265	43.54% ISShvi3_aa1	44.88% CP019911_00909	IS91	3543672	3545372	1.701
A2HRMARDI	CP019911_01861	41.07% ISKpn25_aa1	No hit	ISL3	1952815	1954260	1.446
A2HRMARDI	CP019911_02736	53.33% TnShfr1_aa1	No hit	Tn3	2872956	2874179	1.224
PSI07	RPSI07_2797	54.66% ISHpa1_aa1	No hit	IS1595 ssgr IS1016	2926813	2927208	396
PSI07	RPSI07_2798	57.24% ISNme3_aa1	100% RPSI07_2797	IS1595 ssgr IS1016	2927106	2927591	486
PSI07	RPSI07_0358	40.71% ISFsp3_aa2	62.67% RPSI07_2191	IS21	347302	345398	1.905
PSI07	RPSI07_0819	73.84% ISPst3_aa2	No hit	IS21	829257	828973	285
PSI07	RPSI07_2499	94.84% ISRso8_aa1	No hit	IS3	2618476	2618183	294
PSI07	RPSI07_1530	98.36% ISRso10_aa1	100% RPSI07_2490	IS3 ssgr IS2	1558207	1558596	390
PSI07	RPSI07_1531	95.74% ISRso10_aa2	100% RPSI07_2969	IS3 ssgr IS2	1558593	1559441	849
PSI07	RPSI07_1633	98.41% ISRso10_aa2	100% RPSI07_2969	IS3 ssgr IS2	1653511	1653320	192
PSI07	RPSI07_1634	93.30% ISRso10_aa2	93.11% RPSI07_2969	IS3 ssgr IS2	1654169	1653384	786
PSI07	RPSI07_1635	98.34% ISRso10_aa1	100% RPSI07_2490	IS3 ssgr IS2	1654531	1654166	366
PSI07	RPSI07_2489	95.74% ISRso10_aa2	100% RPSI07_2969	IS3 ssgr IS2	2612582	2611734	849
PSI07	RPSI07_2490	98.36% ISRso10_aa1	100% RPSI07_1530	IS3 ssgr IS2	2612968	2612579	390
PSI07	RPSI07_2969	95.74% ISRso10_aa2	100% RPSI07_2489	IS3 ssgr IS2	3109820	3108972	849
PSI07	RPSI07_2970	98.34% ISRso10_aa1	100% RPSI07_2490	IS3 ssgr IS2	3110182	3109817	366
PSI07	RPSI07_1614	89.21% IS222_aa1	No hit	IS3 ssgr IS3	1641677	1642009	333
PSI07	RPSI07_1615	90.65% IS222_aa2	46.89% RPSI07_1859	IS3 ssgr IS3	1642123	1642863	741

PSI07	RPSI07_1859	50.32% ISRosp1_aa2	46.89% RPSI07_1615	IS3 ssgr IS3	1900712	1900146	567
PSI07	RPSI07_0298	98.86% ISRso16_aa1	100% RPSI07_2965	IS3 ssgr IS407	298781	299047	267
PSI07	RPSI07_0299	98.59% ISRso16_aa2	98.59% RPSI07_2495	IS3 ssgr IS407	29908	299298	219
PSI07	RPSI07_0812	94.31% ISRso14_aa2	93.82% RPSI07_1639	IS3 ssgr IS407	823776	823228	549
PSI07	RPSI07_1482	98.86% ISRso16_aa1	100% RPSI07_2965	IS3 ssgr IS407	1512906	1513172	267
PSI07	RPSI07_1483	97.14% ISRso16_aa2	100% RPSI07_2495	IS3 ssgr IS407	1513205	1514047	843
PSI07	RPSI07_1523	98.85% IS407_aa1	100% RPSI07_3376	IS3 ssgr IS407	1549315	1549578	264
PSI07	RPSI07_1524	96.72% ISRso14_aa2	99.63% RPSI07_3375	IS3 ssgr IS407	1549605	1550432	828
PSI07	RPSI07_1603	88.88% ISXca2_aa2	100% RPSI07_1633	IS3 ssgr IS407	1630259	1630585	327
PSI07	RPSI07_1638	98.85% IS407_aa1	100% RPSI07_3376	IS3 ssgr IS407	1656062	1656325	264
PSI07	RPSI07_1639	97.45% ISRso14_aa2	99.27% RPSI07_1524	IS3 ssgr IS407	1656352	1657179	828
PSI07	RPSI07_2494	98.86% ISRso16_aa1	100% RPSI07_2965	IS3 ssgr IS407	2614168	2614434	267
PSI07	RPSI07_2495	97.14% ISRso16_aa2	100% RPSI07_1483	IS3 ssgr IS407	2614467	2615309	843
PSI07	RPSI07_2518	95.81% ISRso16_aa2	98.85% RPSI07_2495	IS3 ssgr IS407	2644325	2643534	792
PSI07	RPSI07_2519	98.86% ISRso16_aa1	100% RPSI07_2965	IS3 ssgr IS407	2644675	2644409	267
PSI07	RPSI07_2731	98.86% ISRso16_aa1	100% RPSI07_2965	IS3 ssgr IS407	2856717	2856983	267
PSI07	RPSI07_2732	97.17% ISRso16_aa2	99.19% RPSI07_2495	IS3 ssgr IS407	2857016	2857816	801
PSI07	RPSI07_2964	96.95% ISRso16_aa2	100% RPSI07_2495	IS3 ssgr IS407	3104758	3103967	792
PSI07	RPSI07_2965	98.86% ISRso16_aa1	100% RPSI07_2731	IS3 ssgr IS407	3105108	3104842	267
PSI07	RPSI07_3375	96.36% ISRso14_aa2	99.63% RPSI07_1524	IS3 ssgr IS407	3512986	3512159	828
PSI07	RPSI07_3376	98.85% IS407_aa1	100% RPSI07_1638	IS3 ssgr IS407	3513276	3513013	264
PSI07	RPSI07_1853	76.92% ISMtsp16_aa1	No hit	IS481	1897249	1896818	432
PSI07	RPSI07_1854	68.71% ISMtsp16_aa1	No hit	IS481	1897770	1897228	543
PSI07	RPSI07_1491	96.26% ISRso1_aa1	53.09% RPSI07_1612	IS5	1517884	1518528	645
PSI07	RPSI07_1612	76.29% ISCARN14_aa1	No hit	IS5 ssgr IS1031	1639822	1641000	1.179
PSI07	RPSI07_0317	82.14% ISBcen20_aa1	No hit	IS5 ssgr IS427	310587	310186	402
PSI07	RPSI07_1294	87.16% ISCARN25_aa1	No hit	IS630	1320419	1319469	951
PSI07	RPSI07_0922	47.36% ISMno23_aa1	52.59% RPSI07_3326	IS91	929012	928086	927
PSI07	RPSI07_3326	48.75% ISWz1_aa1	52.59% RPSI07_0922	IS91	3459349	3460338	990
PSI07	RPSI07_0044	40.80% ISSm4_aa1	No hit	ISL3	50641	52086	1.446
PSI07	RPSI07_0010	50.46% ISPa4_aa1	No hit	ISNCY	1582	17595	1.776
PSI07	RPSI07_1861	77.27% ISKpn21_aa1	84.84% RPSI07_1863	ISNCY ssgr IS1202	1904360	1904013	348
PSI07	RPSI07_1863	72.27% ISKpn21_aa1	84.84% RPSI07_1861	ISNCY ssgr IS1202	1906169	1904694	1.476
PSI07	RPSI07_0532	0% newcandidate	not_found	New_Family	520527	521252	726
PSI07	RPSI07_0877	0% newcandidate	not_found	New_Family	887466	887263	204

PSI07	RPSI07_0203	54.71% ISMpo10_aa3	45.67% RPSI07_1637	Tn3	211213	209825	1.389
PSI07	RPSI07_0738	53.33% TnShfr1_aa1	No hit	Tn3	73894	740163	1.224
PSI07	RPSI07_1637	75.93% ISSba14_aa1	45.67% RPSI07_0203	Tn3	1655290	1655868	579
KACC10722	IS_5b99f844_00253	49.48% ISHvo5_aa1	No hit	IS1595 ssgr ISH4	270346	271134	789
KACC10722	IS_5b99f844_00740	70.76% ISPst3_aa2	No hit	IS21	791704	79142	285
KACC10722	IS_5b99f844_01086	92.85% ISXca2_aa2	55.76% IS_5b99f844_02475	IS3 ssgr IS407	1166860	1166345	516
KACC10722	IS_5b99f844_02475	97.33% ISRso16_aa2	55.76% IS_5b99f844_01086	IS3 ssgr IS407	2699461	2698670	792
KACC10722	IS_5b99f844_02673	72.41% ISAtu5_aa1	70% IS_5b99f844_02477	IS3 ssgr IS407	2909278	2909538	261
KACC10722	IS_5b99f844_03091	56.77% IS1421_aa3	No hit	IS5 ssgr IS427	3344970	3344344	627
KACC10722	IS_5b99f844_00248	85.93% ISAau3_aa1	No hit	IS5 ssgr IS5	264171	26444	270
KACC10722	IS_5b99f844_02474	80.30% ISCARN25_aa1	79.10% IS_5b99f844_02684	IS630	2697948	2698181	234
KACC10722	IS_5b99f844_02684	84.21% ISCARN25_aa1	79.10% IS_5b99f844_02474	IS630	2926238	2926492	255
KACC10722	IS_5b99f844_00836	47.16% ISTha3_aa2	52.59% IS_5b99f844_03250	IS91	891449	890523	927
KACC10722	IS_5b99f844_03250	48.75% ISWz1_aa1	52.76% IS_5b99f844_00836	IS91	3521778	3522767	990
KACC10722	IS_5b99f844_00040	40.71% ISKpn25_aa1	50.76% IS_5b99f844_01078	ISL3	44434	45879	1.446
KACC10722	IS_5b99f844_01078	42.36% ISSm4_aa1	50.76% IS_5b99f844_00040	ISL3	1156873	1158330	1.458
KACC10722	IS_5b99f844_01836	55.89% ISKpn21_aa1	No hit	ISNCY ssgr IS1202	2002273	2001782	492
KACC10722	IS_5b99f844_00189	61.81% ISMpo10_aa3	94.65% IS_5b99f844_00261	Tn3	20169	200302	1.389
KACC10722	IS_5b99f844_00261	53.08% ISMpo10_aa3	94.65% IS_5b99f844_00189	Tn3	278862	277486	1.377
KACC10722	IS_5b99f844_00670	53.33% TnShfr1_aa1	No hit	Tn3	711323	712534	1.212

Partial elements

Table S3b. Characterization of Insertion sequences elements found in the chromosome of *Ralstonia* spp. with ISFinder database

Strain	IS N°	Name	IS Family	Subgroups	IS Lenght	Position
GMI1000	1	ISRso6	IS21	-	2064	275155-277218
GMI1000	2	ISRso6	IS21	-	2065	2531573- 2533636
GMI1000	1	IS1090	IS256	-	1250	282798 -284124
GMI1000	1	ISRs07	IS256	-	13220	282785-284128
GMI1000	1	ISBcen17	IS3	IS3	1184	2458038 -2459319
GMI1000	2	ISBcen17	IS3	IS3	1184	1661730- 1663011
GMI1000	3	ISBcen17	IS3	IS3	1191	622252-623533
GMI1000	1	ISRsO11	IS3	IS150	1483	1540157-1541605
GMI1000	2	ISRs011	IS3	IS150	1545	3326411- 3327859
GMI1000	3	ISRs011	IS3	IS150	1505	2613222-2614670
GMI1000	4	ISR011	IS3	IS125	2670	2456643 -2458033
GMI1000	1	ISRs010	IS3	IS2	1377	1538788-1540122
GMI1000	2	ISRs010	IS3	IS2	1284	2001514 -2002848
GMI1000	3	ISRs010	IS3	IS2	1146	2530436-2531572
GMI1000	4	ISRs010	IS3	IS2	155	2533637 -2533838
GMI1000	1	ISRs08	IS3	-	1335	622247-623533
GMI1000	2	ISRs08	IS3	-	1293	1661725-1663011
GMI1000	3	ISRs08	IS3	-	1315	2458033-2459319
GMI1000	1	ISRs014	IS3	IS407	1266	869921 -871154
GMI1000	2	ISRs014	IS3	IS407	1197	1596962-1598195
GMI1000	3	ISRs014	IS3	IS407	1190	2611668 -2612901
GMI1000	1	ISRs012	IS3	IS407	1251	1541741- 1542965
GMI1000	2	ISRs012	IS3	IS407	1114	2910961-2912185
GMI1000	1	ISBph1	IS3	IS3	1180	1661729-1663011
GMI1000	2	ISBph1	IS3	IS3	1180	2458037-2459319
GMI1000	3	ISBph1	IS3	IS3	1180	622251-623533
GMI1000	1	ISRso13	IS4	IS4	1485	3427433 -3428899
GMI1000	2	ISRso13	IS4	IS4	1465	1589424 -1590890
GMI1000	3	ISRso13	IS4	IS4	1465	2047824-2049290
GMI1000	4	ISRso13	IS4	IS4	1461	3498806-3500272
GMI1000	1	ISRso9	IS5	IS5	1483	3695665 -3697147
GMI1000	2	ISRso9	IS5	IS5	1485	3395322-3396804
GMI1000	3	ISRso9	IS5	IS5	1485	3365734-3367216
GMI1000	4	ISRso9	IS5	IS5	1481	883212- 884694
GMI1000	1	ISRs018	IS5	IS5	1227	1820117-1821304
GMI1000	1	ISRs01	IS5	-	830	3612024 -3612907
GMI1000	2	ISRs01	IS5	-	867	231871 -232754
GMI1000	3	ISRs01	IS5	-	830	3460579-3461462
GMI1000	4	ISRs01	IS5	-	849	3483308 -3484191
GMI1000	5	ISRs01	is5	-	827	1059501 -1060384
GMI1000	1	IS1421	IS5	IS427	809	1792206 -1793069
GMI1000	1	IS1405	IS5	IS5	1197	1820273-1821298
GMI1000	1	ISButh4	IS5	IS5	1322	3695757-3697099
GMI1000	2	ISButh4	IS5	IS5	1315	3395414-3396756
GMI1000	3	ISButh4	IS5	IS5	1320	3365826-3367168
GMI1000	4	ISButh4	IS5	IS5	1320	883260-884602
GMI1000	1	ISRs05	IS630	-	1091	3659388-3660546
GMI1000	2	ISRs05	IS630	-	1170	117921 -119079

GMI1000	3	ISRs05	IS630	-	1170	683361 -684519
GMI1000	4	ISRs05	IS630	-	1166	2360114 -2361272
GMI1000	1	ISRs015	ISL3	-	1339	2780073 -2781376
P082	1	ISrso19	IS21	-	1960	1695519- 1697474
P082	2	ISrso19	IS21	-	1958	380817- 382772
P082	3	ISrso19	IS21	-	1942	2854409-2856364
P082	4	ISrso19	IS21	-	1961	2646582 - 2648537
P082	5	ISrso19	IS21	-	1947	1610771 - 1612726
P082	6	ISrso19	IS21	-	1961	1171840- 1173795
P082	1	ISRs010	IS3	IS2	1336	2937657-2938992
P082	2	ISRs010	IS3	IS2	1245	1606603- 1607938
P082	3	ISRs010	IS3	IS2	1224	1605094-1606429
P082	4	ISRs010	IS3	IS2	1338	2643537-2644872
P082	5	ISRs010	IS3	IS2	1267	2922032 - 2923367
P082	6	ISRs010	IS3	IS2	1334	303404-304739
P082	1	IS401	IS3	IS51	1296	1356668-1357986
P082	2	IS401	IS3	IS51	1390	2866276-2867594
P082	3	IS401	IS3	IS51	886	1694605- 1695518
P082	4	IS401	IS3	IS51	1700	1607939-1608677
UY031	1	ISBma3	IS110	-	955	3121893-3123068
UY031	2	ISRs020	IS3	IS3	I285	2305894 -2307180
UY031	1	ISRs010	IS3	IS2	1350	3220488- 3221825
UY031	2	ISRs010	IS3	IS2	1349	489042-490379
UY031	3	ISRs010	IS3	IS2	1350	2300247-2301584
UY031	4	ISRs010	IS3	IS2	1360	1260562 - 1261899
UY031	5	ISRs010	IS3	IS2	1350	2012919-2014256
UY031	6	ISRs010	IS3	IS2	1339	2816320-2817657
UY031	1	ISBxe2	IS3	IS3	298	2296720-2297990
UY031	2	ISBxe2	IS3	IS3	1184	2305910-2307180
UY031	1	ISRs016	IS3	IS407	1143	2698654-2699892
UY031	1	IS1021	IS5	IS5	I220	3404962-3406170
UY031	2	IS1021	IS5	IS5	1216	50549-51757
UY031	3	IS1021	IS5	IS5	I220	3185978- 3187186
UY031	4	IS1021	IS5	IS5	I220	269475- 270683
UY031	5	IS1021	IS5	IS5	1223	308625- 309833
UY031	6	IS1021	IS5	IS5	1220	320157-321365
UY031	7	IS1021	IS5	IS5	1216	428734- 429942
UY031	8	IS1021	IS5	IS5	1221	2809796 -2811004
UY031	9	IS1021	IS5	IS5	1216	2580115-2581323
UY031	10	IS1021	IS5	IS5	1236	2427177-2428385
UY031	11	IS1021	IS5	IS5	1218	2392692-2393900
UY031	12	IS1021	IS5	IS5	I126	1051468 -1052676
UY031	13	IS1021	IS5	IS5	1218	2343629-2344837
UY031	14	IS1021	IS5	IS5	1220	1162776- 1163984
UY031	15	IS1021	IS5	IS5	1218	1257033-1258241
UY031	16	IS1021	IS5	IS5	1220	2151233- 2152441
UY031	17	IS1021	IS5	IS5	1216	2149023-2150231
UY031	18	IS1021	IS5	IS5	1227	2087335 -2088543
UY031	19	IS1021	IS5	IS5	1220	1437951- 1439159

UY031	20	IS1021	IS5	IS5	1216	2089080 -2090288
UY031	21	IS1021	IS5	IS5	1080	1237646-1238854
UY031	22	IS1021	IS5	IS5	1218	1203714- 1204922
UY031	23	IS1021	IS5	IS5	1215	2301602- 2302810
UY031	24	IS1021	IS5	IS5	1128	2388925-2390133
UY031	25	IS1021	IS5	IS5	1075	949013- 950221
UY031	26	IS1021	IS5	IS5	I223	2514722-2515930
UY031	27	IS1021	IS5	IS5	1223	2574230 -2575438
UY031	28	IS1021	IS5	IS5	1220	2707776-2708984
UY031	29	IS1021	IS5	IS5	1215	2732980 - 2734188
UY031	30	IS1021	IS5	IS5	1213	2793777-2794985
UY031	31	IS1021	IS5	IS5	1217	2805791-2806999
UY031	32	IS1021	IS5	IS5	1221	343704-344912
UY031	33	IS1021	IS5	IS5	I225	3252612-3253820
UY031	34	IS1021	IS5	IS5	989	74881-76089
UY031	35	IS1021	IS5	IS5	1213	64638-65846
UY031	36	IS1021	IS5	IS5	990	57487- 58695
UY031	37	IS1021	IS5	IS5	1218	1252906-1254114
UW163	1	ISRs019	IS21	-	1956	3297445-3299400
UW163	2	ISRs019	IS21	-	1956	906378-908333
UW163	3	ISRs019	IS21	-	1871	3283360-3285315
UW163	4	ISRs019	IS21	-	1956	321666-323621
UW163	5	ISRs019	IS21	-	1956	1261637-1263592
UW163	6	ISRs019	IS21	-	1956	1479706-1481661
UW163	7	ISRs019	IS21	-	1956	2054511-2056466
UW163	8	ISRs019	IS21	-	1956	235019-236974
UW163	9	ISRs019	IS21	-	1956	3371413-3373368
UW163	10	ISRs019	IS21	-	1956	309831- 311786
UW163	1	ISRs010	IS3	IS2	1336	28769-30104
UW163	2	ISRs010	IS3	IS2	1580	3368849-3370184
UW163	3	ISRs010	IS3	IS2	1335	302600 -303935
UW163	4	ISRs010	IS3	IS2	1336	2487316-2488651
UW163	5	ISRs010	IS3	IS2	1336	318992- 320327
UW163	1	IS401	IS3	IS51	1350	2493088-2494406
UW163	2	IS401	IS3	IS51	1354	2239337-2240655
UW163	3	IS401	IS3	IS51	1328	2485311-2486629
UW163	4	IS401	IS3	IS51	1304	246886-248204
UW163	5	IS401	IS3	IS51	871	3285316-3286229
IBSBF1503	2	ISRs019	IS21	-	1964	2729776-2731731
IBSBF1503	3	ISRs019	IS21	-	1960	1707852-1709807
IBSBF1503	4	ISRs019	IS21	-	1953	920417-922372II
IBSBF1503	5	ISRs019	IS21	-	1056	511027-512087
IBSBF1503	1	ISRso19	IS21	-	1959	1408482-1410437
IBSBF1503	2	ISRso19	IS21	-	1955	1935156-1937111
IBSBF1503	3	ISRso19	IS21	-	1959	710057-712012
IBSBF1503	1	ISRs010	IS3	IS2	1301	2939646-2940981
IBSBF1503	2	ISRs010	IS3	IS2	1319	2671063-2672398
IBSBF1503	3	ISRs010	IS3	IS2	1337	465685-467020
IBSBF1503	1	IS401	IS3	IS51	1272	736217-737535

IBSBF1503	2	IS401	IS3	IS51	868	426892- 427805
IBSBF1503	1	ISRs011	IS3	IS150	1470	3083874-3085322
RS488	1	ISBma3	IS110	-	1175	3121891 -3123066
RS488	1	ISRs020	IS3	IS3	1213	2296710-2297996
RS488	2	ISRs020	IS3	IS3	1213	2305884-2307170
RS488	1	ISRs010	IS3	IS2	1337	3220488-3221825
RS488	2	ISRs010	IS3	IS2	1337	489037-490374
RS488	3	ISRs010	IS3	IS2	1337	2300237-2301574
RS488	1	ISRs010	IS3	IS2	1345	3108941-3110261
RS488	1	ISBxe2	IS3	IS3	1270	2296710-2297980
RS488	2	ISBxe2	IS3	IS3	1270	2305900-2307170
RS488	1	IS1021	IS5	IS5	1220	3404962 -3406170
RS488	2	IS1021	IS5	IS5	982	50549 -51757
RS488	3	IS1021	IS5	IS5	988	3185977- 3187185
RS488	4	IS1021	IS5	IS5	991	269475-270683
RS488	5	IS1021	IS5	IS5	1091	308625 -309833
RS488	6	IS1021	IS5	IS5	1217	308701-309909
RS488	7	IS1021	IS5	IS5	I225	320231-321439
RS488	8	IS1021	IS5	IS5	1219	2809785-2810993
RS488	9	IS1021	IS5	IS5	1219	2580105-2581313
RS488	10	IS1021	IS5	IS5	1225	-
RS488	11	IS1021	IS5	IS5	1217	-
RS488	12	IS1021	IS5	IS5	1235	-
RS488	13	IS1021	IS5	IS5	1224	-
RS488	14	IS1021	IS5	IS5	1217	-
RS488	15	IS1021	IS5	IS5	1219	-
RS488	16	IS1021	IS5	IS5	1220	-
RS488	17	IS1021	IS5	IS5	1221	-
RS488	18	IS1021	IS5	IS5	1215	-
RS488	19	IS1021	IS5	IS5	1217	-
RS488	20	IS1021	IS5	IS5	1220	-
RS488	21	IS1021	IS5	IS5	1215	-
RS488	22	IS1021	IS5	IS5	1235	-
RS488	23	IS1021	IS5	IS5	1216	-
RS488	24	IS1021	IS5	IS5	1227	-
RS488	25	IS1021	IS5	IS5	1214	-
RS488	26	IS1021	IS5	IS5	1233	-
RS488	27	IS1021	IS5	IS5	1222	-
RS488	28	IS1021	IS5	IS5	1232	-
RS488	29	IS1021	IS5	IS5	1223	-
RS488	30	IS1021	IS5	IS5	1231	-
RS488	31	IS1021	IS5	IS5	1224	-
RS488	32	IS1021	IS5	IS5	1234	-
RS488	33	IS1021	IS5	IS5	1221	-
RS488	34	IS1021	IS5	IS5	1220	-
RS488	35	IS1021	IS5	IS5	1223	-
RS488	36	IS1021	IS5	IS5	1225	-
RS488	37	IS1021	IS5	IS5	1223	-
RS488	38	IS1021	IS5	IS5	1224	-
RS488	39	IS1021	IS5	IS5	1230	-

RS488	40	IS1021	IS5	IS5	1215	-
CFBP2957	1	ISRso7	IS256	-	1344	2505863-2507206
CFBP2957	2	ISRso7	IS256	-	1344	41869-43210
CFBP2957	3	ISRso7	IS256	-	1344	1634792-1636135
CFBP2957	4	ISRso7	IS256	-	1344	3218175-3219518
CFBP2957	5	ISRso7	IS256	-	1344	1170304-1171642
CFBP2957	1	ISRso10	IS3	IS2	1335	1141957-1143292
CFBP2957	2	ISRso10	IS3	IS2	1335	2507449-2508784
CFBP2957	1	ISRso16	IS3	IS407	1238	7054-8292
CFBP2957	2	ISRso16	IS3	IS407	1238	1144490-1145507
CFBP2957	1	IS1090	IS256	-	1343	2505876-2507202
CFBP2957	2	IS1090	IS256	-	1343	3218179-3219514
CFBP2957	3	IS1090	IS256	-	1343	41873-43206
CFBP2957	4	IS1090	IS256	-	1343	1634796-1636122
CFBP2957	5	IS1090	IS256	-	1343	1170308-1171638
UW551	1	ISRso20	IS3	IS3	1287	1211473-1212759
UW551	2	ISRso20	IS3	IS3	1287	1201852-1203138
UW551	1	IS1021	IS3	IS5	1209	9945-11153
UW551	2	IS1021	IS3	IS5	1209	17096-18304
UW551	3	IS1021	IS3	IS5	1209	27338-28546
UW551	4	IS1021	IS3	IS5	1209	34489-35697
UW551	5	IS1021	IS3	IS5	1209	99150-100358
UW551	6	IS1021	IS3	IS5	1209	3235344-3236552
UW551	7	IS1021	IS3	IS5	1209	3197462-3198670
UW551	8	IS1021	IS3	IS5	1209	320637-321845
UW551	9	IS1021	IS3	IS5	1209	3076062-3077270
UW551	10	IS1021	IS3	IS5	1209	698036-699244
UW551	11	IS1021	IS3	IS5	1209	2453336-2454544
UW551	12	IS1021	IS3	IS5	1209	1080649-1081857
UW551	13	IS1021	IS3	IS5	1209	1115133-1116341
UW551	14	IS1021	IS3	IS5	1209	2342029-2343237
UW551	15	IS1021	IS3	IS5	1209	1164195-1165403
UW551	16	IS1021	IS3	IS5	1209	1206734-1207942
UW551	17	IS1021	IS3	IS5	1209	1357037-1358245
UW551	18	IS1021	IS3	IS5	1209	1418723-1419931
UW551	19	IS1021	IS3	IS5	1209	1416978-1418186
UW551	20	IS1021	IS3	IS5	1209	2068071-2069279
UW551	21	IS1021	IS3	IS5	1209	2267159-2268367
UW551	22	IS1021	IS3	IS5	1209	2301091-2302299
UW551	23	IS1021	IS3	IS5	1209	1118900-1120108
UW551	24	IS1021	IS3	IS5	1209	993108-994316
UW551	25	IS1021	IS3	IS5	1209	933600-934808
UW551	26	IS1021	IS3	IS5	1209	2555788-2556996
UW551	27	IS1021	IS3	IS5	1209	801268-802476
UW551	28	IS1021	IS3	IS5	1209	776064-777272
UW551	29	IS1021	IS3	IS5	1209	773112-774320
UW551	30	IS1021	IS3	IS5	1209	714055-715263
UW551	31	IS1021	IS3	IS5	1209	702041-703249
UW551	32	IS1021	IS3	IS5	1209	371712-372920

UW551	33	IS1021	IS3	IS5	1209	3161091-3162299
UW551	34	IS1021	IS3	IS5	1209	3200244-3201452
UW551	35	IS1021	IS3	IS5	1209	3211775-3212983
UW551	36	IS1021	IS3	IS5	1209	253996-255204
UW551	37	IS1021	IS3	IS5	1209	251499-252707
UW551	38	IS1021	IS3	IS5	1209	41427-42635
UW551	39	IS1021	IS3	IS5	1209	3429938-3431146
UW551	40	IS1021	IS3	IS5	1209	3440180-3441388
UW551	41	IS1021	IS3	IS5	1209	3447331-3448539
UW551	42	IS1021	IS3	IS5	1209	3457573-3458781
UW551	43	IS1021	IS3	IS5	1209	3464724-3465932
UW551	44	IS1021	IS3	IS5	1209	2251899-2253107
UW551	45	IS1021	IS3	IS5	910	1-910
UW551	1	ISRso10	IS3	IS5	1335	285990-287327
UW551	2	ISRso10	IS3	IS5	1335	3015625-3016962
UW551	3	ISRso10	IS3	IS5	1335	2245327-2246664
UW551	4	ISRso10	IS3	IS5	1335	1493009-1494346
UW551	5	ISRso10	IS3	IS5	1335	1205379-1206716
UW551	6	ISRso10	IS3	IS5	1335	691384-692721
UW551	1	ISBma3	IS110	-	1458	385967-387142
UW551	1	ISBxe2	IS3	IS3	1287	1211489-1212759
UW551	2	ISBxe2	IS3	IS3	1287	1201852-1203122

RS489	1	ISBma3	IS110	-	1175	3121718-3122893
RS489	1	ISRs020	IS3	IS3	1286	2296729- 2298015
RS489	2	ISRs020	IS3	IS3	1286	2305903 -2307189
RS489	1	ISRs010	IS3	IS2	1337	3220315- 3221652
RS489	2	ISRs010	IS3	IS2	1337	489148-490485
RS489	3	ISRs010	IS3	IS2	1337	1260592-1261929
RS489	1	ISBxe2	IS3	IS3	1270	2296729-2297999
RS489	2	ISBxe2	IS3	IS3	1270	2305919 -2307189
RS489	1	IS1021	IS5	IS5	1220	3404962 -3406170
RS489	2	IS1021	IS5	IS5	982	50549 -51757
RS489	3	IS1021	IS5	IS5	988	3185977- 3187185
RS489	4	IS1021	IS5	IS5	991	269475-270683
RS489	5	IS1021	IS5	IS5	1091	308625 -309833
RS489	6	IS1021	IS5	IS5	1217	308701-309909
RS489	7	IS1021	IS5	IS5	I225	320231-321439
RS489	8	IS1021	IS5	IS5	1227	2809696-2810904
RS489	9	IS1021	IS5	IS5	1229	1051485 -1052693
RS489	10	IS1021	IS5	IS5	1216	2089103 -2090311
RS489	11	IS1021	IS5	IS5	1554	1203746 -1204954
RS489	12	IS1021	IS5	IS5	1216	2793686 -2794894
RS489	13	IS1021	IS5	IS5	1223	3252440 - 3253648
RS489	14	IS1021	IS5	IS5	1203	3185810 -3187018
RS489	15	IS1021	IS5	IS5	1218	2580045-2581253
RS489	16	IS1021	IS5	IS5	I220	2427107 -2428315
RS489	17	IS1021	IS5	IS5	1216	2392660 -2393868
RS489	18	IS1021	IS5	IS5	1226	1162798-1164006
RS489	19	IS1021	IS5	IS5	1221	2149004-2150212
RS489	20	IS1021	IS5	IS5	1218	2087358-2088566

RS489	21	IS1021	IS5	IS5	1221	1438046-1439254
RS489	22	IS1021	IS5	IS5	1081	1252936-1254144
RS489	23	IS1021	IS5	IS5	1204	949050-950258
RS489	24	IS1021	IS5	IS5	1218	305919-307127
RS489	25	IS1021	IS5	IS5	1219	74906-76114
RS489	26	IS1021	IS5	IS5	1219	2514654-2515861
RS489	27	IS1021	IS5	IS5	1211	428815 - 430023
RS489	28	IS1021	IS5	IS5	1203	2343622-2344830
RS489	29	IS1021	IS5	IS5	1224	1257063-1258271
RS489	30	IS1021	IS5	IS5	1232	1237676-1238884
RS489	31	IS1021	IS5	IS5	1311	2301611-2302819
RS489	32	IS1021	IS5	IS5	1220	2388893- 2390101
RS489	33	IS1021	IS5	IS5	1217	2574160-2575368
RS489	34	IS1021	IS5	IS5	1224	2707724-2708932
RS489	35	IS1021	IS5	IS5	1212	2732921 -2734129
RS489	36	IS1021	IS5	IS5	1219	2805691-2806899
RS489	37	IS1021	IS5	IS5	1225	343797-345005
RS489	38	IS1021	IS5	IS5	1213	64667-65875
OE1-1	1	ISBma3	IS110	-	1257	2022580-2024035
OE1-1	1	ISRs011	IS3	IS150	1479	2850246-2851694
OE1-1	1	ISButh1	IS3	IS2	1331	3539419- 3540748
OE1-1	1	ISRs016	IS3	IS407	753	1891844-1892601
OE1-1	1	IS1405	IS5	IS5	1330	3552304-3553477
OE1-1	2	IS1405	IS5	IS5	1173	2581178-2582351
OE1-1	3	IS1405	IS5	IS5	1171	1672257-1673430
OE1-1	4	IS1405	IS5	IS5	1773	2026682-2027855
OE1-1	5	IS1405	IS5	IS5	1182	2126575-2127748
OE1-1	6	IS1405	IS5	IS5	1182	3458874-3460047
OE1-1	7	IS1405	IS5	IS5	1184	3579827-3581000
OE1-1	8	IS1405	IS5	IS5	1181	2954275-2955448
OE1-1	1	ISRs01	IS5	-	885	253452-254335
OE1-1	2	ISRs01	IS5	-	883	2025659-2026542
OE1-1	3	ISRs01	IS5	-	881	3527805-3528688
OE1-1	4	ISRs01	IS5	-	884	2955846-2956729
OE1-1	1	ISRs018	IS5	IS5	964	3579833-3580858
OE1-1	2	ISRs018	IS5	IS5	1407	3458880-3459905
OE1-1	3	ISRs018	IS5	IS5	965	2954281-2955306
OE1-1	4	ISRs018	IS5	IS5	1183	2126581-2127606
OE1-1	5	ISRs018	IS5	IS5	1179	2026688 -2027713
OE1-1	6	ISRs018	IS5	IS5	962	1672263-1673288
OE1-1	7	ISRs018	IS5	IS5	964	2581320-2582345
OE1-1	8	ISRs018	IS5	IS5	1179	3552446-3553471
OE1-1	1	IS1021	IS5	IS5	987	711317-712407
OE1-1	1	ISRs021	ISL3	-	2046	828748-830779
K60	1	ISRs019	IS21	-	1095	3635884-3637839
K60	2	ISRs019	IS21	-	1095	1844205-1846160
K60	3	ISRs019	IS21	-	1095	2475656-2477504
K60	4	ISRs019	IS21	-	569	3126492-3127138
K60	1	ISRme4	IS21	-	1526	3638968-3641436

K60	2	ISRme4	IS21	-	1526	887952-890420
K60	3	ISRme4	IS21	-	1526	2149924-2152392
K60	4	ISRme4	IS21	-	1527	2477504-2479972
K60	5	ISRme4	IS21	-	1526	2628525-2630993
K60	1	ISRs022	IS1595	ISPna2	1065	84932-85983
K60	2	ISRs022	IS1595	ISPna2	1060	3272275- 3273326
K60	3	ISRs022	IS1595	ISPna2	1065	371992-373043
K60	4	ISRs022	IS1595	ISPna2	1071	3601874-3602925
K60	1	IS401	IS3	IS51	1449	3598312-3599630
K60	2	IS401	IS3	IS51	1385	859664-860982
K60	1	ISRs01	IS5	-	841	2125225-2126108
K60	1	ISBma3	IS110	-	1265	211884 -213339
K60	1	ISRs020	IS3	IS3	745	3522105-3522840
K60	1	ISVei4	IS630	-	1128	1222503-1223662
K60	2	ISVei4	IS630	-		1358373-1359451
FJAT-1458	1	ISRs011	IS3	IS150	1473	2253218-2254606
FJAT-1458	2	ISRs011	IS3	IS150	1470	94098- 95546
FJAT-1458	1	IS405	IS5	IS5	1182	3034792-3035965
FJAT-1458	2	IS405	IS5	IS5	1181	222987-224160
FJAT-1458	3	IS405	IS5	IS5	1181	307273-308446
FJAT-1458	4	IS405	IS5	IS5	1180	2742556-2743729
FJAT-1458	5	IS405	IS5	IS5	1181	1426084-1427257
FJAT-1458	6	IS405	IS5	IS5	1182	1914506- 1915679
FJAT-1458	7	IS405	IS5	IS5	1182	1758677-1759850
FJAT-1458	8	IS405	IS5	IS5	1180	1457528-1458701
FJAT-1458	9	IS405	IS5	IS5	1181	3694518-3695691
FJAT-1458	1	ISRs01	IS5	-	884	1997282-1998165
FJAT-1458	2	ISRs01	IS5	-	840	3050734-3051617
FJAT-1458	3	ISRs01	IS5	-	883	277396-278279
FJAT-1458	4	ISRs01	IS5	-	839	274682 -275562
FJAT-1458	1	ISRs018	IS5	IS5	1181	3694524-3695549
FJAT-1458	2	ISRs018	IS5	IS5	1180	1457670-1458695
FJAT-1458	3	ISRs018	IS5	IS5	1152	1758819-1759844
FJAT-1458	4	ISRs018	IS5	IS5	965	1914512-1915537
FJAT-1458	5	ISRs018	IS5	IS5	967	1426090-1427115
FJAT-1458	6	ISRs018	IS5	IS5	1522	2742698- 2743723
FJAT-1458	7	ISRs018	IS5	IS5	958	307279-308304
FJAT-1458	8	ISRs018	IS5	IS5	1183	223129-224154
FJAT-1458	9	ISRs018	IS5	IS5	1182	3034934-3035959
FJAT-1458	1	IS1021	IS5	IS5	1160	3695813-3696903
FJAT-1458	2	IS1021	IS5	IS5	1374	2748070-2749160
FJAT-1458	3	IS1021	IS5	IS5	1217	1265941-1267031
FJAT-1458	4	IS1021	IS5	IS5	1227	1629074-1630164
FJAT-1458	5	IS1021	IS5	IS5	1881	1963521-1964611
FJAT-1458	6	IS1021	IS5	IS5	1214	1930169-1931259
FJAT-1458	7	IS1021	IS5	IS5	1214	1899994-1901084
FJAT-1458	8	IS1021	IS5	IS5	1538	2131208-2132298
FJAT-1458	9	IS1021	IS5	IS5	12014	1622938-1624028
FJAT-1458	10	IS1021	IS5	IS5	988	1432242-1433332
FJAT-1458	11	IS1021	IS5	IS5	1213	2770202-2771292

FJAT-1458	12	IS1021	IS5	IS5	1217	3650565-3651655
FJAT-1458	1	ISRs017	IS701	-	1506	1804596- 1806066
EP1	1	ISBma3	IS110	-	1205	1806215-1807670
EP1	1	ISRs019	IS21	-	1957	2992581-2994536
EP1	1	ISRs011	IS3	IS150	1401	987562-989010
EP1	1	ISBurh1	IS3	IS2	1329	282591-283920
EP1	1	ISRs016	IS3	IS407	657	3465934-3466591
EP1	1	ISRs011	IS3	IS150	1480	2700400-2701848
EP1	1	IS1405	IS5	IS5	1183	269862-271035
EP1	2	IS1405	IS5	IS5	1177	1802395-1803568
EP1	3	IS1405	IS5	IS5	1182	1702505- 1703678
EP1	4	IS1405	IS5	IS5	1184	2717510-2718683
EP1	5	IS1405	IS5	IS5	1183	2972531-2973704
EP1	6	IS1405	IS5	IS5	1181	363292-364465
EP1	7	IS1405	IS5	IS5	1181	883808-884981
EP1	1	ISRs01	IS5	-	841	2950773 -2951656
EP1	2	ISRs01	IS5	-	956	3695386-3696269
EP1	3	ISRs01	IS5	-	886	1803708-1804591
EP1	4	ISRs01	IS5	-	885	294651-295534
EP1	5	ISRs01	IS5	-	573	879259-879832
EP1	6	ISRs01	IS5	-	316	875371-875689
EP1	1	ISRs018	IS5	IS5	1411	363434-364459
EP1	2	ISRs018	IS5	IS5	965	883950-884975
EP1	3	ISRs018	IS5	IS5	960	2972537-2973562
EP1	4	ISRs018	IS5	IS5	1185	2717516-2718541
EP1	5	ISRs018	IS5	IS5	1178	1702647 -1703672
EP1	6	ISRs018	IS5	IS5	1020	1802537-1803562
EP1	7	ISRs018	IS5	IS5	1544	269868-270893
EP1	1	IS1021	IS5	IS5	1255	3239649-3240739
CQPS- 1	1	ISRs011	IS3	IS150	1468	2390432-2391880
CQPS- 1	2	ISRs011	IS3	IS150	1455	2502314- 2503762
CQPS- 1	1	ISRs014	IS3	IS407	1170	1445207-1446440
CQPS- 1	2	ISRs014	IS3	IS407	1173	1266536- 1267769
CQPS- 1	3	ISRs014	IS3	IS407	1174	982476-983709
CQPS- 1	1	ISButh1	IS3	IS2	1329	2286663-2287992
CQPS- 1	1	ISRs016	IS3	IS407	265	978742-979399
CQPS- 1	1	IS1405	IS5	IS5	1183	605415 -606588
CQPS- 1	2	IS1405	IS5	IS5	1178	3142781-3143954
CQPS- 1	3	IS1405	IS5	IS5	1182	1033104- 1034277
CQPS- 1	4	IS1405	IS5	IS5	1188	1268263- 1269436
CQPS- 1	5	IS1405	IS5	IS5	1184	1717974-1719147
CQPS- 1	6	IS1405	IS5	IS5	1182	1691834-1693007
CQPS- 1	7	IS1405	IS5	IS5	1180	2275684-2276857
CQPS- 1	8	IS1405	IS5	IS5	1180	429523-430696
CQPS- 1	9	IS1405	IS5	IS5	1183	3822102-3823274
CQPS- 1	10	IS1405	IS5	IS5	1182	479128-480300
CQPS- 1	1	IS1421	IS5	IS427	872	361314-362175
CQPS- 1	1	ISRs01	IS5	-	870	38916-390051
CQPS- 1	2	ISRs01	IS5	-	1211	3192321-3193204

CQPS- 1	3	ISRs01	IS5	-	883	1709711-1710594
CQPS- 1	1	ISRs018	IS5	IS5	979	429665 -430690
CQPS- 1	2	ISRs018	IS5	IS5	976	479269-480294
CQPS- 1	3	ISRs018	IS5	IS5	1183	2275690-2276715
CQPS- 1	4	ISRs018	IS5	IS5	1575	1691976-1693001
CQPS- 1	5	ISRs018	IS5	IS5	1671	1717980-1719005
CQPS- 1	6	ISRs018	IS5	IS5	964	1268269-1269294
CQPS- 1	7	ISRs018	IS5	IS5	1183	1033110-1034135
CQPS- 1	8	ISRs018	IS5	IS5	1185	3142923-3143948
CQPS- 1	9	ISRs018	IS5	IS5	1185	605421-606446
CQPS- 1	10	ISRs018	IS5	IS5	968	3822243-3823268
CQPS- 1	1	IS1021	IS5	IS5	1328	490477-491567
CQPS- 1	2	IS1021	IS5	IS5	1257	752793-753883
CQPS- 1	2	ISRs017	IS701	-	1495	1261845- 1263315
FJAT- 91	2	ISRs011	IS3	IS150	1458	201893-203341
FJAT- 91	3	ISRs011	IS3	IS150	1463	3833086- 3834534
FJAT- 91	4	ISRs011	IS3	IS150	1455	1716524-1717969
FJAT- 91	1	IS1405	IS5	IS5	1183	1318441- 1319614
FJAT- 91	2	IS1405	IS5	IS5	1181	3395328- 3396501
FJAT-91	3	IS1405	IS5	IS5	1181	3831825-3832998
FJAT-91	4	IS1405	IS5	IS5	1183	243914-245087
FJAT-91	5	IS1405	IS5	IS5	1183	281284-282457
FJAT-91	6	IS1405	IS5	IS5	1183	3490298 -3491471
FJAT-91	7	IS1405	IS5	IS5	1182	899168-900341
FJAT-91	8	IS1405	IS5	IS5	1183	2365570-2366743
FJAT-91	9	IS1405	IS5	IS5	1182	1054611- 1055784
FJAT-91	10	IS1405	IS5	IS5	1183	2823548-2824721
FJAT-91	11	IS1405	IS5	IS5	1180	855139-856312
FJAT-91	12	IS1405	IS5	IS5	1182	291453-292626
FJAT-91	13	IS1405	IS5	IS5	1184	3809361-3810534
FJAT-91	1	IS1421	IS5	IS427	869	578824-579687
FJAT-91	1	ISRs01	IS5	-	887	896810-897693
FJAT-91	2	ISRs01	IS5	-	886	3520459-3521342
FJAT-91	3	ISRs01	IS5	-	835	3524388-3525268
FJAT-91	1	ISRs018	IS5	IS5	1183	3809427 -3810392
FJAT-91	2	ISRs018	IS5	IS5	1879	291595 -292620
FJAT-91	3	ISRs018	IS5	IS5	1979	855281-856306
FJAT-91	4	ISRs018	IS5	IS5	1182	2823554 -2824579
FJAT-91	5	ISRs018	IS5	IS5	1267	1054753-1055778
FJAT-91	6	ISRs018	IS5	IS5	1181	2365576-2366601
FJAT-91	7	ISRs018	IS5	IS5	1454	899174-900199
FJAT-91	8	ISRs018	IS5	IS5	963	3490440-3491465
FJAT-91	9	ISRs018	IS5	IS5	1355	281290-282315
FJAT-91	10	ISRs018	IS5	IS5	1458	243920 -244945
FJAT-91	11	ISRs018	IS5	IS5	1182	3831967-3832992
FJAT-91	12	ISRs018	IS5	IS5	1180	1318583-1319608
FJAT-91	13	ISRs018	IS5	IS5	1133	3395336 -3396359
FJAT-91	1	IS1021	IS5	IS5	1221	300074-301163
FJAT-91	2	IS1021	IS5	IS5	1214	3394116-3395206

FJAT-91	3	IS1021	IS5	IS5	1214	89832-90922
FJAT-91	4	IS1021	IS5	IS5	1216	3746766-3747856
FJAT-91	5	IS1021	IS5	IS5	1216	242702-243792
FJAT-91	6	IS1021	IS5	IS5	1211	290241-291331
FJAT-91	7	IS1021	IS5	IS5	1220	298862 -299952
FJAT-91	8	IS1021	IS5	IS5	1215	2939655-2940745
FJAT-91	9	IS1021	IS5	IS5	1413	1294695-1295785
FJAT-91	10	IS1021	IS5	IS5	1213	2552596-2553686
FJAT-91	11	IS1021	IS5	IS5	1215	588363-589453
FJAT-91	12	IS1021	IS5	IS5	1215	3521899-3522989
FJAT-91	13	IS1021	IS5	IS5	1218	292748-293838
FJAT-91	14	IS1021	IS5	IS5	1216	245209-246299
FJAT-91	15	IS1021	IS5	IS5	1215	116749-117839
FJAT-91	16	IS1021	IS5	IS5	1052	3802196-3803286
FJAT-91	17	IS1021	IS5	IS5	1217	3826521-3827611
FJAT-91	1	ISRs017	IS701	-	1481	1900573-1902043
FQY_4	1	ISBma3	IS110	-	1266	3080038-3081494
FQY_4	2	ISBma3	IS110	-	1264	662044- 663499
FQY_4	1	ISBma2	IS1182	-	1440	2291365-2292701
FQY_4	2	ISRs011	IS3	IS150	1531	1552091-1553539
FQY_4	3	ISRs011	IS3	IS150	1473	3348267-3349715
FQY_4	1	ISRs016	IS3	IS407	333	457847-458504
FQY_4	1	IS1405	IS5	IS5	1184	3291867-3293040
FQY_4	2	IS1405	IS5	IS5	1184	240918-242091
FQY_4	3	IS1405	IS5	IS5	1186	3085605-3086778
FQY_4	4	IS1405	IS5	IS5	1181	869603-870776
FQY_4	5	IS1405	IS5	IS5	1178	2031259-2032432
FQY_4	6	IS1405	IS5	IS5	1184	2253824-2254997
FQY_4	7	IS1405	IS5	IS5	1180	845787-846960
FQY_4	8	IS1405	IS5	IS5	1187	277026-278199
FQY_4	1	IS1421	IS5	IS427	884	3263369-3264226
FQY_4	2	IS1421	IS5	IS427	1011	209645-210502
FQY_4	3	IS1421	IS5	IS427	1015	127260-128117
FQY_4	4	IS1421	IS5	IS427	527	3691902-3692413
FQY_4	1	ISRs01	IS5	-	843	867245- 868128
FQY_4	2	ISRs01	IS5	-	882	875707-876589
FQY_4	3	ISRs01	IS5	-	1073	1969099-1969545
FQY_4	1	ISRs018	IS5	IS5	1182	277168 -278193
FQY_4	2	ISRs018	IS5	IS5	1095	3291873-3292898
FQY_4	3	ISRs018	IS5	IS5	2022	845929-846954
FQY_4	4	ISRs018	IS5	IS5	1179	2253830 -2254855
FQY_4	5	ISRs018	IS5	IS5	1183	2031265-2032290
FQY_4	6	ISRs018	IS5	IS5	1455	869609-870634
FQY_4	7	ISRs018	IS5	IS5	1181	3085747-3086772
FQY_4	8	ISRs018	IS5	IS5	1185	240924-241949
FQY_4	1	IS1021	IS5	IS5	983	3070708-3071797
FQY_4	2	IS1021	IS5	IS5	1214	2788431-2789521
FQY_4	3	IS1021	IS5	IS5	1216	2804164- 2805254
FQY_4	4	IS1021	IS5	IS5	1000	303016-304105
FQY_4	5	IS1021	IS5	IS5	1127	2647259-2648127

FQY_4	1	ISVei4	IS630	-	1168	1435817-1436896
RSCM	1	ISRs07	IS256	-	1300	2229265-2230603
RSCM	2	ISRs07	IS256	-	1300	3133378-3134716
RSCM	3	ISRs07	IS256	-	1305	417573-418911
RSCM	4	ISRs07	IS256	-	1300	282707-284045
RSCM	5	ISRs07	IS256	-	1195	3157289-3158508
RSCM	6	ISRs07	IS256	-	1191	1088222-1089441
RSCM	1	IS1090	IS256	-	1284	2229261-2230590
RSCM	2	IS1090	IS256	-	1286	3133391-3134720
RSCM	3	IS1090	IS256	-	1291	417569-418898
RSCM	4	IS1090	IS256	-	1291	282720-284049
RSCM	5	IS1090	IS256	-	1230	3157285-3158514
RSCM	6	IS1090	IS256	-	1235	1088218-1089447
RSCM	1	ISRs011	IS3	IS150	1443	1099365-1100813
RSCM	2	ISRs011	IS3	IS150	1479	2970628-2972076
RSCM	3	ISRs011	IS3	IS150	1475	3385248-3386696
RSCM	4	ISRs011	IS3	IS150	1481	1915378-1916826
RSCM	1	ISRs014	IS3	IS407	1239	13952-15185
RSCM	2	ISRs014	IS3	IS407	1270	350563-351796
RSCM	3	ISRs014	IS3	IS407	1286	611041-612274
RSCM	4	ISRs014	IS3	IS407	1195	1237962-1239195
RSCM	5	ISRs014	IS3	IS407	1443	209171-209838
RSCM	6	ISRs014	IS3	IS407	736	3211139-3211806
RSCM	7	ISRs014	IS3	IS407	535	211133-211701
RSCM	8	ISRs014	IS3	IS407	542	3209276-3209844
RSCM	9	ISRs014	IS3	IS407	531	1904317-1904885
RSCM	1	IS401	IS3	IS51	1320	981598-982916
RSCM	2	IS401	IS3	IS51	1342	2729839-2731157
RSCM	3	IS401	IS3	IS51	1368	1474241-1475559
RSCM	4	IS401	IS3	IS51	1340	1806930-1808248
RSCM	5	IS401	IS3	IS51	1404	1913811-1915129
RSCM	6	IS401	IS3	IS51	1375	1315849-1317167
RSCM	7	IS401	IS3	IS51	1341	1240145-1241463
RSCM	8	IS401	IS3	IS51	1394	2594248-2595566
RSCM	9	IS401	IS3	IS51	1409	1081977-1083295
RSCM	10	IS401	IS3	IS51	1354	943309-944627
RSCM	11	IS401	IS3	IS51	1386	211706-213019
RSCM	1	ISButh1	IS3	IS2	652	1313063-1313709
RSCM	2	ISButh1	IS3	IS2	653	2573730-2574376
RSCM	3	ISButh1	IS3	IS2	655	3083319-3083965
RSCM	4	ISButh1	IS3	IS2	652	3372943-3373589
RSCM	5	ISButh1	IS3	IS2	680	1315037-1315689
RSCM	6	ISButh1	IS3	IS2	678	2571750-2572402
RSCM	7	ISButh1	IS3	IS2	682	3077624-3078276
RSCM	8	ISButh1	IS3	IS2	680	3370963-3371615
RSCM	1	ISRs011	IS3	IS150	1484	736054-737502
RSCM	1	IS1405	IS5	IS5	1182	990714-991887
RSCM	2	IS1405	IS5	IS5	1186	1085979-1087152
RSCM	3	IS1405	IS5	IS5	1183	1208231-1209404
RSCM	4	IS1405	IS5	IS5	1183	1503376-1504549

RSCM	5	IS1405	IS5	IS5	1183	1674171-1675344
RSCM	6	IS1405	IS5	IS5	1183	1894185-1895358
RSCM	7	IS1405	IS5	IS5	1183	1755881-1757054
RSCM	8	IS1405	IS5	IS5	1183	2191872-2193045
RSCM	9	IS1405	IS5	IS5	1183	1512948-1514121
RSCM	10	IS1405	IS5	IS5	1183	1210529-1211702
RSCM	11	IS1405	IS5	IS5	1181	1089451-1090624
RSCM	12	IS1405	IS5	IS5	1183	2756163-2757336
RSCM	13	IS1405	IS5	IS5	1183	630516- 631689
RSCM	14	IS1405	IS5	IS5	1183	3158518- 3159691
RSCM	15	IS1405	IS5	IS5	1181	352809-353982
RSCM	16	IS1405	IS5	IS5	1185	36219-37392
RSCM	17	IS1405	IS5	IS5	1181	3756736-3757909
RSCM	18	IS1405	IS5	IS5	1183	2747971-2749144
RSCM	1	IS1421	IS5	IS427	861	2746251-2747108
RSCM	2	IS1421	IS5	IS427	868	12904-13730
RSCM	3	IS1421	IS5	IS427	873	1011396-1012222
RSCM	4	IS1421	IS5	IS427	860	2728454-2729280
RSCM	5	IS1421	IS5	IS427	860	2062753-2063579
RSCM	6	IS1421	IS5	IS427	870	2072577-2073403
RSCM	7	IS1421	IS5	IS427	872	2081286-2082112
RSCM	8	IS1421	IS5	IS427	862	932917-933743
RSCM	9	IS1421	IS5	IS427	862	642793-643619
RSCM	10	IS1421	IS5	IS427	860	5638-6464
RSCM	11	IS1421	IS5	IS427	870	1310425-1311286
RSCM	12	IS1421	IS5	IS427	870	3342705-3343531
RSCM	13	IS1421	IS5	IS427	860	3642869-3643689
RSCM	14	IS1421	IS5	IS427	870	1464719-1465544
RSCM	1	ISRs01	IS5	-	838	2727567-2728450
RSCM	2	ISRs01	IS5	-	884	3162458-3163341
RSCM	3	ISRs01	IS5	-	882	976110-976993
RSCM	4	ISRs01	IS5	-	878	1757375-1758258
RSCM	5	ISRs01	IS5	-	885	3645118-3646001
RSCM	6	ISRs01	IS5		866	1811710-1812585
RSCM	1	ISRs018	IS5	IS5	1189	2714128-2715153
RSCM	2	ISRs018	IS5	IS5	1182	2748113-2749138
RSCM	3	ISRs018	IS5	IS5	1113	3756878-3757903
RSCM	4	ISRs018	IS5	IS5	968	36361-37386
RSCM	5	ISRs018	IS5	IS5	1123	352951-353976
RSCM	6	ISRs018	IS5	IS5	1347	3158524-3159549
RSCM	7	ISRs018	IS5	IS5	1514	630658-631683
RSCM	8	ISRs018	IS5	IS5	1116	2756169-2757194
RSCM	9	ISRs018	IS5	IS5	1287	1089593-1090618
RSCM	10	ISRs018	IS5	IS5	966	1210671-1211696
RSCM	11	ISRs018	IS5	IS5	1100	1513090-1514115
RSCM	12	ISRs018	IS5	IS5	1184	2191878-2192903
RSCM	13	ISRs018	IS5	IS5	1217	1756023-1757048
RSCM	14	ISRs018	IS5	IS5	1189	1894327-1895352
RSCM	15	ISRs018	IS5	IS5	966	1674177-1675202
RSCM	16	ISRs018	IS5	IS5	1037	1503382-1504407
RSCM	17	ISRs018	IS5	IS5	1037	1208237-1209262

RSCM	18	ISRs018	IS5	IS5	968	1085985-1087010
RSCM	19	ISRs018	IS5	IS5	1124	990720-991745
RSCM	1	IS1021	IS5	IS5	987	314752-315599
RSCM	2	IS1021	IS5	IS5	1222	3398550-3399397
RSCM	3	IS1021	IS5	IS5	983	3353025-3353872
RSCM	4	IS1021	IS5	IS5	986	2957172 -2958019
RSCM	5	IS1021	IS5	IS5	1216	2867814-2868661
RSCM	6	IS1021	IS5	IS5	1224	1216476-1217323
RSCM	7	IS1021	IS5	IS5	987	2502526-2503373
RSCM	8	IS1021	IS5	IS5	1213	1321836- 1322683
RSCM	9	IS1021	IS5	IS5	1124	1679642-1680489
RSCM	10	IS1021	IS5	IS5	1214	1675467-1676314
RSCM	11	IS1021	IS5	IS5	1214	1444843-1445690
RSCM	12	IS1021	IS5	IS5	987	1354383-1355230
RSCM	13	IS1021	IS5	IS5	1214	983453-984300
RSCM	2	ISRs017	IS701	-	1483	215107- 216577
RSCM	3	ISRs017	IS701	-	1477	3317054-3318524
RSCM	4	ISRs017	IS701	-	1476	3160983-3162453
RSCM	5	ISRs017	IS701	-	1479	1758263-1759733
RSCM	6	ISRs017	IS701	-	1483	1897784-1899254
RSCM	7	ISRs017	IS701	-	1480	1810235-1811705
RSCM	8	ISRs017	IS701	-	1477	985748-987218
RSCM	9	ISRs017	IS701	-	1163	1353165-1354268
RSCM	10	ISRs017	IS701	-	379	1355471-1355846
RSCM	2	ISPa38	Tn3	-	595	654841-655436
RSCM	3	ISPa38	Tn3	-	934	653440-654374
RSCM	1	TnShfr1	Tn3	-	3174	650579-653608
RSCM	1	TnAs3	Tn3	-	2955	650575-653074
RSCM	1	ISShes11	Tn3	-	2972	650575-653074
RSCM	1	TnAs2	Tn3	-	2972	650575-653072
RSCM	1	ISSod9	Tn3	-	2,977	650579-653595
RSCM	1	TnAs1	Tn3	-	2969	650776-651989
T60	2	ISRs011	IS3	IS150	1448	951887-953335
T60	1	ISRs011	IS3	IS150	1526	2145779-2147227
T60	1	IS1405	IS5	IS5	1173	245592-246765
T60	2	IS1405	IS5	IS5	1173	3441846-3443019
T60	3	IS1405	IS5	IS5	1173	305266-306439
T60	4	IS1405	IS5	IS5	1173	3321146-3322319
T60	5	IS1405	IS5	IS5	1173	2782691-2783864
T60	6	IS1405	IS5	IS5	1173	2647254-2648427
T60	7	IS1405	IS5	IS5	1173	1649591 - 1650764
T60	8	IS1405	IS5	IS5	1173	1483406-1484579
T60	9	IS1405	IS5	IS5	1173	2764804-2765977
T60	10	IS1405	IS5	IS5	1173	3381877-3383050
T60	1	IS1420	IS5	IS930	1130	696994-698124
T60	2	IS1420	IS5	IS930	1130	3001980-3003110
T60	3	IS1420	IS5	IS930	1130	838561-839691
T60	4	IS1420	IS5	IS930	1130	3004543-3005673
T60	5	IS1420	IS5	IS930	1130	3044377-3045507
T60	6	IS1420	IS5	IS930	1130	3393135-3394265

T60	7	IS1420	IS5	IS930	1130	14886-16016
T60	1	ISRs01	IS5	-	883	1486182-1487065
T60	2	ISRs01	IS5	-	883	2248737-2249620
T60	1	ISRs018	IS5	IS5	1025	3381883-3382908
T60	2	ISRs018	IS5	IS5	1025	2764810-2765835
T60	3	ISRs018	IS5	IS5	1025	1483548-1484573
T60	4	ISRs018	IS5	IS5	1025	1649733-1650758
T60	5	ISRs018	IS5	IS5	1025	2647396-2648421
T60	6	ISRs018	IS5	IS5	1025	2782833-2783858
T60	7	ISRs018	IS5	IS5	1025	3321288-3322313
T60	8	ISRs018	IS5	IS5	1025	305272-306297
T60	9	ISRs018	IS5	IS5	1025	3441988-3443013
T60	10	ISRs018	IS5	IS5	1025	245734-246759
SL3882	2	ISRs011	IS3	IS150	1465	1066524-1067972
SL3882	1	ISRs011	IS3	IS150	1532	2163754-2165202
SL3882	1	IS1405	IS5	IS5	1185	320361-321534
SL3882	2	IS1405	IS5	IS5	1185	2730086-2731259
SL3882	3	IS1405	IS5	IS5	1182	1540964-1542137
SL3882	4	IS1405	IS5	IS5	1184	964777-965950
SL3882	5	IS1405	IS5	IS5	1183	2995208-2996381
SL3882	6	IS1405	IS5	IS5	1184	454598-455771
SL3882	7	IS1405	IS5	IS5	1183	3611092-3612265
SL3882	8	IS1405	IS5	IS5	1181	3714676-3715849
SL3882	9	IS1405	IS5	IS5	1185	379765-380938
SL3882	1	IS1420	IS5	IS903	1130	1708616-1709746
SL3882	1	ISRs01	IS5	-	883	3819074-3819957
SL3882	2	ISRs01	IS5	-	885	1308724-1309607
SL3882	3	ISRs01	IS5	-	886	2287258-2288141
SL3882	4	ISRs01	IS5	-	881	3249109-3249992
SL3882	5	ISRs01	IS5	-		2934507-2935390
SL3882	1	ISRs018	IS5	IS5	967	3714682-3715707
SL3882	2	ISRs018	IS5	IS5	1184	3611098-3612123
SL3882	3	ISRs018	IS5	IS5	1170	454740-455765
SL3882	4	ISRs018	IS5	IS5	1331	2995214-2996239
SL3882	5	ISRs018	IS5	IS5	969	964919-965944
SL3882	6	ISRs018	IS5	IS5	1182	1541106-1542131
SL3882	7	ISRs018	IS5	IS5	1179	2730228-2731253
SL3882	8	ISRs018	IS5	IS5	966	379771-380796
SL3882	9	ISRs018	IS5	IS5	1177	320503-321528
SL3882	1	IS1021	IS5	IS5	1215	2550330-2551420
SL3882	2	IS1021	IS5	IS5	1076	2296186-2297276
SL3882	3	IS1021	IS5	IS5	990	356031-357121
SL3882	4	IS1021	IS5	IS5	986	3662409-3663499
SL3882	5	IS1021	IS5	IS5	990	2530522-2531612
SL3882	6	IS1021	IS5	IS5	1203	667560-668650
T42	2	ISRs011	IS3	IS150	1484	951887-953335
T42	1	ISRs011	IS3	IS150	1528	2184327-2185775
T42	1	IS1405	IS5	IS5	1186	245592-246765
T42	2	IS1405	IS5	IS5	1182	3441846-3443019

T42	3	IS1405	IS5	IS5	1181	305266-306439
T42	4	IS1405	IS5	IS5	1182	3321146-3322319
T42	5	IS1405	IS5	IS5	1183	2782691-2783864
T42	6	IS1405	IS5	IS5	1178	2647254-2648427
T42	7	IS1405	IS5	IS5	1181	1649591-1650764
T42	8	IS1405	IS5	IS5	1183	1483406-1484579
T42	9	IS1405	IS5	IS5	1184	2764804-2765977
T42	10	IS1405	IS5	IS5	1183	3381877-3383050
T42	1	IS1420	IS5	IS930	1130	696994-698124
T42	2	IS1420	IS5	IS930	1130	3001980-3003110
T42	3	IS1420	IS5	IS930	1130	838561-839691
T42	4	IS1420	IS5	IS930	1130	3004543-3005673
T42	5	IS1420	IS5	IS930	1130	3044377-3045507
T42	6	IS1420	IS5	IS930	1130	3393135-3394265
T42	7	IS1420	IS5	IS930	1130	14886-16016
T42	1	ISRs01	IS5	-	886	1486182-1487065
T42	2	ISRs01	IS5	-	883	2248737-2249620
T42	1	ISRs018	IS5	IS5	1183	3381883-3382908
T42	2	ISRs018	IS5	IS5	1330	2764810-2765835
T42	3	ISRs018	IS5	IS5	1182	1483548-1484573
T42	4	ISRs018	IS5	IS5	964	1649733-1650758
T42	5	ISRs018	IS5	IS5	1181	2647396-2648421
T42	6	ISRs018	IS5	IS5	1184	2782833-2783858
T42	7	ISRs018	IS5	IS5	1181	3321288-3322313
T42	8	ISRs018	IS5	IS5	964	3322313-306297
T42	9	ISRs018	IS5	IS5	1207	3441988-3443013
T42	10	ISRs018	IS5	IS5	1178	245734-246759
T42	1	IS1021	IS5	IS5	1217	3312457-3313547
T42	2	IS1021	IS5	IS5	1354	724099-725189
T42	3	IS1021	IS5	IS5	1213	848174-849264
T42	4	IS1021	IS5	IS5	1216	3335549-3336639
T42	5	IS1021	IS5	IS5	1213	3505710-3506800
SL3300	1	ISRs06	IS21	-	2089	275149-277212
SL3300	1	ISBp1	IS3	IS407	1197	574765-575962
SL3300	2	ISRs011	IS5	IS150	1448	967750-969198
SL3300	1	IS1420	IS5	IS903	1130	2914013-2915143
SL3300	2	IS1420	IS5	IS903	1130	2848029-2849159
SL3300	3	IS1420	IS5	IS903	1130	2785280-2786410
SL3300	1	IS1405	IS5	IS5	1130	313373-314546
SL3300	2	IS1405	IS5	IS5	1130	2665235-2666408
SL3300	3	IS1405	IS5	IS5	1130	1505062-1506235
SL3300	4	IS1405	IS5	IS5	1130	1274691-1275864
SL3300	5	IS1405	IS5	IS5	1130	2908889-2910062
SL3300	6	IS1405	IS5	IS5	1130	854275-855448
SL3300	7	IS1405	IS5	IS5	1130	3462417-3463590
SL3300	1	ISRs01	IS5	-	883	2266721-2267604
SL3300	2	ISRs01	IS5	-	883	2793738-2794621
SL3300	3	ISRs01	IS5	-	883	283496-284379
SL3300	4	ISRs01	IS5	-	883	280782-281662
SL3300	1	ISRs018	IS5	IS5	1197	3462423-3463448

SL3300	2	ISRs018	IS5	IS5	1197	854417-855442
SL3300	3	ISRs018	IS5	IS5	1197	2908895-2909920
SL3300	4	ISRs018	IS5	IS5	1197	1274833-1275858
SL3300	5	ISRs018	IS5	IS5	1197	1505204-1506229
SL3300	6	ISRs018	IS5	IS5	1197	2665377-2666402
SL3300	7	ISRs018	IS5	IS5	1197	313379-314404
SL3300	1	IS1021	IS5	IS5	1197	3514742-3515832
SL3300	2	IS1021	IS5	IS5	1197	2791173-2792263
SL3300	3	IS1021	IS5	IS5	1197	981683-982773
SL3300	4	IS1021	IS5	IS5	1197	2911524-2912614
SL3300	5	IS1021	IS5	IS5	1197	3478690-3479780
SL3822	2	ISRs011	IS3	IS150	1465	967750-969198
SL3822	1	ISBp1	IS3	IS407	1112	574765- 575962
SL3822	1	IS1420	IS5	IS930	1132	2914013-2915143
SL3822	2	IS1420	IS5	IS930	1135	2848029-2849159
SL3822	3	IS1420	IS5	IS930	1135	2785280-2786410
SL3822	1	IS1405	IS5	IS5	1182	313373-314546
SL3822	2	IS1405	IS5	IS5	1179	2665235-2666408
SL3822	3	IS1405	IS5	IS5	1181	1505062- 1506235
SL3822	4	IS1405	IS5	IS5	1185	1274691-1275864
SL3822	5	IS1405	IS5	IS5	1183	2908889-2910062
SL3822	6	IS1405	IS5	IS5	1186	854275-855448
SL3822	7	IS1405	IS5	IS5	1183	3462417-3463590
SL3822	1	ISRs01	IS5	-	881	2266721-2267604
SL3822	2	ISRs01	IS5	-	842	2793738-2794621
SL3822	3	ISRs01	IS5	-	884	283496-284379
SL3822	4	ISRs01	IS5	-	838	280782-281662
SL3822	1	ISRs018	IS5	IS5	1183	3462423-3463448
SL3822	2	ISRs018	IS5	IS5	1033	854417-855442
SL3822	3	ISRs018	IS5	IS5	1181	2908895-2909920
SL3822	4	ISRs018	IS5	IS5	1324	1274833-1275858
SL3822	5	ISRs018	IS5	IS5	1185	1505204-1506229
SL3822	6	ISRs018	IS5	IS5	1184	2665377- 2666402
SL3822	7	ISRs018	IS5	IS5	967	313379- 314404
SL3822	1	IS1021	IS5	IS5	1611	3514742-3515832
SL3822	2	IS1021	IS5	IS5	1215	2791173-2792263
SL3822	3	IS1021	IS5	IS5	985	981683-982773
SL3822	4	IS1021	IS5	IS5	1216	2911524-2912614
SL3822	5	IS1021	IS5	IS5	1218	3478690-3479780
SL3822	1	ISRs011	IS5	IS150	1448	2163754-2165202
HA4-1	1	ISRme9	IS21	-	2362	3684120-3686804
HA4-1	2	ISRme9	IS21	-	2365	3222252-3224936
HA4-1	1	ISBma3	IS110	-	1267	3095232-3096688
HA4-1	2	ISBma3	IS110	-	1261	1148337-1149793
HA4-1	3	ISBma3	IS110	-	265	3278047-3279503
HA4-1	1	ISBcen18	IS256	-	1306	2371831-2373196
HA4-1	2	ISRso11	IS3	IS150	1466	1625070-1626518
HA4-1	3	ISRso11	IS3	IS150	1470	495865- 497313
HA4-1	4	ISRso11	IS3	IS150	52	628032-629480

HA4-1	1	ISRso12	IS3	IS407	2234	2541181-2542065
HA4-1	2	ISRso12	IS3	IS407	368	2542928-2543272
HA4-1	1	IS1420	IS5	IS903	1134	3147330-3148460
HA4-1	2	IS1420	IS5	IS903	616	2430700- 2431314
HA4-1	1	IS1421	IS5	IS427	874	2104783-2105646
HA4-1	2	IS1421	IS5	IS427	868	2542065-2542928
HA4-1	3	IS1421	IS5	IS427	869	716705-717568
HA4-1	4	IS1421	IS5	IS427	870	921240-922099
HA4-1	5	IS1421	IS5	IS427	870	2462547-2463410
HA4-1	6	IS1421	IS5	IS427	873	3201396-3202259
HA4-1	7	IS1421	IS5	IS427	870	2866063-2866925
HA4-1	8	IS1421	IS5	IS427	806	1232330-1233189
HA4-1	1	ISRs01	IS5	-	841	3849873-3850756
HA4-1	2	ISRs01	IS5	-	842	2120614-2121497
HA4-1	3	ISRs01	IS5	-	887	3698871-3699754
HA4-1	4	ISRs01	IS5	-	843	2446257-2447140
HA4-1	5	ISRs01	IS5	-	888	1233197-1234080
HA4-1	6	ISRs01	IS5	-	845	2514097-2514983
HA4-1	7	ISRs01	IS5	-	849	1223575-1224461
HA4-1	8	ISRs01	IS5	-	830	2516088-2516958
HA4-1	1	IS1021	IS5	IS5	990	3192034-3193124
HA4-1	2	IS1021	IS5	IS5	1212	1117908-1118998
HA4-1	3	IS1021	IS5	IS5	1219	2493662-2494752
HA4-1	4	IS1021	IS5	IS5	1215	2460021-2461111
HA4-1	5	IS1021	IS5	IS5	1214	2109183-2110273
HA4-1	6	IS1021	IS5	IS5	1214	2099059-2100149
KACC10709	2	ISRs011	IS3	IS150	1481	2885654-2887102
KACC10709	1	ISRs010	IS3	IS2	880	3002381-3003355
KACC10709	2	ISRs010	IS3	IS2	440	3004721-3005079
KACC10709	1	IS1420	IS5	IS903	1131	12673-13803
KACC10709	2	IS1420	IS5	IS903	1127	3370737-3371867
KACC10709	3	IS1420	IS5	IS903	1131	3009628-3010758
KACC10709	4	IS1420	IS5	IS903	1129	2924019-2925149
KACC10709	5	IS1420	IS5	IS903	1130	1671898-1673028
KACC10709	6	IS1420	IS5	IS903	1130	1752142-1753272
KACC10709	7	IS1420	IS5	IS903	1132	786219-787349
KACC10709	8	IS1420	IS5	IS903	1128	2922584-2923714
KACC10709	9	IS1420	IS5	IS903	1130	420029-421159
KACC10709	10	IS1420	IS5	IS903	1131	3236553-3237683
KACC10709	11	IS1420	IS5	IS903	1131	682520-683649
KACC10709	12	IS1420	IS5	IS903	1121	665866-666996
KACC10709	1	IS1405	IS5	IS5	1182	152332-153505
KACC10709	2	IS1405	IS5	IS5	1182	2925544- 2926717
KACC10709	3	IS1405	IS5	IS5	1190	76909-78082
KACC10709	4	IS1405	IS5	IS5	1181	644222-645395
KACC10709	5	IS1405	IS5	IS5	1183	462604 -463777
KACC10709	6	IS1405	IS5	IS5	1182	3454621-3455794
KACC10709	7	IS1405	IS5	IS5	1182	367832-369005
KACC10709	8	IS1405	IS5	IS5	1182	3008151-3009324
KACC10709	9	IS1405	IS5	IS5	1184	2950619-2951792

KACC10709	10	IS1405	IS5	IS5	1180	866868-868041
KACC10709	11	IS1405	IS5	IS5	1181	1841261-1842434
KACC10709	1	IS1421	IS5	IS427	863	3239269-3240132
KACC10709	2	ISRs01	IS5	-	884	2948874-2949757
KACC10709	1	ISRs018	IS5	IS5	1183	462746-463771
KACC10709	2	ISRs018	IS5	IS5	965	644364-645389
KACC10709	3	ISRs018	IS5	IS5	961	77051-78076
KACC10709	4	ISRs018	IS5	IS5	965	867010-868035
KACC10709	5	ISRs018	IS5	IS5	962	2925686-2926711
KACC10709	6	ISRs018	IS5	IS5	1328	2950761-2951786
KACC10709	7	ISRs018	IS5	IS5	1485	3008293 -3009318
KACC10709	8	ISRs018	IS5	IS5	965	367838-368863
KACC10709	9	ISRs018	IS5	IS5	1182	152338-153363
KACC10709	10	ISRs018	IS5	IS5	1180	3454763-3455788
KACC10709	11	ISRs018	IS5	IS5	1181	1841267-1842292
KACC10709	1	IS1021	IS5	IS5	1215	145592-146681
KACC10709	2	IS1021	IS5	IS5	1216	243093-244182
KACC10709	3	IS1021	IS5	IS5	1215	3235073-3236162
KACC10709	4	IS1021	IS5	IS5	1216	31051-32140
KACC10709	1	ISRs017	IS701	-	1470	2024184-2025654
CMR15	2	ISBma2	IS1182	-	1525	906989-908325
CMR15	3	ISBma2	IS1182	-	1484	526869- 528205
CMR15	4	ISBma2	IS1182	-	1574	2042987-2044323
CMR15	5	ISBma2	IS1182	-	1576	2457599-2458935
CMR15	6	ISBma2	IS1182	-	1489	1151672-1153010
CMR15	1	ISRs01	IS5	-	732	235599-236294
CMR15	1	ISBdo1	IS1182	-	1445	3561674-3563054
CMR15	2	ISBdo1	IS1182	-	1484	943399-944779
CMR15	3	ISBdo1	IS1182	-	1449	2025322-2026702
CMR15	4	ISBdo1	IS1182	-	1484	2017452-2018832
CMR15	5	ISBdo1	IS1182	-	1514	2993613-2994993
CMR15	6	ISBdo1	IS1182	-	1486	914681-916061
CMR15	7	ISBdo1	IS1182	-	1482	993049-994429
CMR15	8	ISBdo1	IS1182	-	1630	2627696-2629076
CMR15	9	ISBdo1	IS1182	-	1449	741975- 743356
CMR15	10	ISBdo1	IS1182	-	1572	1019312-1020692
CMR15	11	ISBdo1	IS1182	-	1443	2855409-2856789
CMR15	12	ISBdo1	IS1182	-	1129	333971-335351
CMR15	13	ISBdo1	IS1182	-	1588	2077989-2079369
CMR15	14	ISBdo1	IS1182	-	1479	3572759-3574139
CMR15	15	ISBdo1	IS1182	-	1446	2292143 -2292728
CMR15	1	ISBma2	IS1182	-	1440	2291365-2292701
RS 476	1	ISRso6	IS21	-	2079	275212-277275
RS 476	2	ISRs06	IS21	-	2068	2531580-2533643
RS 476	1	IS1090	IS256	-	1256	282792-284118
RS 476	1	ISRs07	IS256	-	1346	282779- 284122
RS 476	1	ISRs011	IS3	IS150	1473	1540156-1541604
RS 476	2	ISRs011	IS3	IS150	1458	3326422-3327870
RS 476	3	ISRs011	IS3	IS150	1481	2613229-2614677

RS 476	4	ISRs011	IS3	IS150	1423	2456650-2458040
RS 476	1	ISRs010	IS3	IS2	1345	1538787-1540121
RS 476	2	ISRs010	IS3	IS2	1351	2001513-2002847
RS 476	3	ISRs010	IS3	IS2	1189	2530443-2531579
RS 476	1	ISRs08	IS3	-	1286	622242-623528
RS 476	2	ISRs08	IS3	-	1372	1661724-1663010
RS 476	3	ISRs08	IS3	-	1384	2458040-2459326
RS 476	1	ISRs014	IS3	IS407	1288	869917-871150
RS 476	2	ISRs014	IS3	IS407	1229	1596960-1598193
RS 476	3	ISRs014	IS3	IS407	1242	2611675- 2612908
RS 476	1	ISRs012	IS3	IS407	1233	1541740-1542964
RS 476	2	ISRs012	IS3	IS407	1223	2910971-2912195
RS 476	1	ISBps1	IS3	IS3	1220	2458040-2459326
RS 476	2	ISBps1	IS3	IS3	1222	1661724-1663010
RS 476	3	ISBps1	IS3	IS3	1214	622242 -623528
RS 476	1	ISBph1	IS3	IS3	1176	2458044-2459326
RS 476	2	ISBph1	IS3	IS3	1182	1661728-1663010
RS 476	3	ISBph1	IS3	IS3	1184	622246- 623528
RS 476	1	ISRs013	IS4	IS4	1465	3427443-3428909
RS 476	2	ISRs013	IS4	IS4	1465	1589423-1590889
RS 476	3	ISRs013	IS4	IS4	1465	2047829-2049295
RS 476	4	ISRs013	IS4	IS4	1463	3498816-3500282
RS 476	1	ISRs09	IS5	IS5	1480	3695674-3697156
RS 476	2	ISRs09	IS5	IS5	1482	3395332-3396814
RS 476	3	ISRs09	IS5	IS5	1486	3365744-3367226
RS 476	4	ISRs09	IS5	IS5	1482	883208-884690
RS 476	1	ISRso1	IS5	-	885	3612034- 3612917
RS 476	2	ISRso1	IS5	-	885	231865-232748
RS 476	3	ISRso1	IS5	-	841	3460589-3461472
RS 476	4	ISRso1	IS5	-	863	3483318-3484201
RS 476	5	ISRso1	IS5	-	837	1059497-1060380
RS 476	1	IS1421	IS5	IS427	871	1792205-1793068
RS 476	1	ISBma1	IS5	IS5	966	1820272-1821297
RS 476	1	ISRs05	IS630	-	1165	3659397-3660555
RS 476	2	ISRs05	IS630	-	1178	117915- 119073
RS 476	3	ISRs05	IS630	-	1166	683356-684514
RS 476	4	ISRs05	IS630	-	1166	2360121- 2361279
RS 476	5	ISRs05	IS630	-	1166	127826-128984
RS 476	1	ISBma1	ISL3	-	1216	2780173-2781395
RS 476	1	ISRs015	ISL3	-	1302	2780092-2781395
CRMRs218	2	ISRso6	IS21	-	2064	2531702-2533765
CRMRs218	1	ISRs07	IS256	-	1291	282842-284185
CRMRs218	1	ISRso13	IS4	IS4	1487	3427745-3429211
CRMRs218	2	ISRso13	IS4	IS4	1465	1589337-1590803
CRMRs218	3	ISRso13	IS4	IS4	1480	2047828-2049294
CRMRs218	4	ISRso13	IS4	IS4	1472	3499117- 3500583
CRMRs218	1	ISRs018	IS5	IS5	1196	1820104-1821291
CRMRs218	1	ISRs05	IS630	-	1170	3659744-3660902
CRMRs218	2	ISRs05	IS630	-	1174	117973-119131
CRMRs218	3	ISRs05	IS630	-	1168	683393-684551

CRMRs218	4	ISRs05	IS630	-	1173	2360231-2361389
CRMRs218	5	ISRs05	IS630	-	1170	127898-129056
CRMRs218	1	IS1090	IS256	-	1335	282846-284181
CRMRs218	1	ISRs08	IS3	-	1546	622307-623593
CRMRs218	2	ISRs08	IS3	-	1375	1661631-1662917
CRMRs218	3	ISRs08	IS3	-	1296	2458168-2459454
CRMRs218	1	ISRso12	IS3	IS407	1238	1541669-1542893
CRMRs218	2	ISRso12	IS3	IS407	1515	2911228-2912452
CRMRs218	1	ISRs010	IS3	IS2	1350	1538712-1540049
CRMRs218	2	ISRs010	IS3	IS2	1357	2001524-2002861
CRMRs218	3	ISRs010	IS3	IS2	1230	2530565-2531701
CRMRs218	1	ISBps1	IS3	IS3	1286	2458168-2459454
CRMRs218	2	ISBps1	IS3	IS3	1286	1661631-1662917
CRMRs218	3	ISBps1	IS3	IS3	1286	622307-623593
CRMRs218	1	ISBph1	IS3	IS3	1282	2458172-2459454
CRMRs218	2	ISBph1	IS3	IS3	1282	1661635-1662917
CRMRs218	3	ISBph1	IS3	IS3	1282	622311-623593
CRMRs218	1	ISRso11	IS3	IS150	1455	1540084-1541532
CRMRs218	2	ISRso11	IS3	IS150	1462	3326675-3328123
CRMRs218	3	ISRso11	IS3	IS150	1479	2613387-2614835
CRMRs218	4	ISRso11	IS3	IS150	2076	2456778-2458168
CRMRs218	1	ISRs014	IS3	IS407	1239	869936-871169
CRMRs218	2	ISRs014	IS3	IS407	1237	1596875-1598108
CRMRs218	3	ISRs014	IS3	IS407	1243	2611833-2613066
CRMRs218	1	ISRs010	IS3	IS2	1334	833868 -835202
CRMRs218	1	ISRso9	IS5	IS5	1499	3696008-3697490
CRMRs218	2	ISRso9	IS5	IS5	1494	3395612-3397094
CRMRs218	3	ISRso9	IS5	IS5	1498	3366023-3367505
CRMRs218	4	ISRso9	IS5	IS5	1500	883223-884705
CRMRs218	1	ISRs01	IS5	-	883	231921-232804
CRMRs218	2	ISRs01	IS5	-	883	3612350-3613233
CRMRs218	3	ISRs01	IS5	-	883	3460893-3461776
CRMRs218	4	ISRs01	IS5	-	883	3483623-3484504
CRMRs218	5	ISRs01	IS5	-	883	1059490-1060373
CRMRs218	1	IS1421	IS5	IS427	863	1792193-1793056
CRMRs218	1	IS1405	IS5	IS5	1025	1820260-1821285
CRMRs218	1	ISRs015	ISL3	-	1315	2780341-2781644
CRMRs218	1	ISBma1	ISL3	-	1222	2780422-2781644
YC40-M	1	ISBma3	IS110	-	1263	716098-717554
YC40-M	2	ISBma3	IS110	-	1260	620294-621750
YC40-M	3	ISBma3	IS110	-	1263	3157364-3158819
YC40-M	4	ISBma3	IS110	-	1263	2847096-2848551
YC40-M	5	ISBma3	IS110	-	1259	1748373-1749828
YC40-M	2	ISRs011	IS3	IS150	1479	2149851-2151299
YC40-M	3	ISRs011	IS3	IS150	1474	348727-350175
YC40-M	1	ISRs016	IS3	IS407	300	3387088-3387745
YC40-M	1	ISRs016	IS3	IS407	300	3387088-3387745
YC40-M	1	ISRs011	IS3	IS150	I457	2282929-2284377
YC40-M	1	IS1405	IS5	IS5	1191	3603513-3604686
YC40-M	2	IS1405	IS5	IS5	1180	3333207-3334380

YC40-M	3	IS1405	IS5	IS5	1181	405394- 406567
YC40-M	4	IS1405	IS5	IS5	1180	2863082-2864255
YC40-M	5	IS1405	IS5	IS5	1177	615010- 616183
YC40-M	6	IS1405	IS5	IS5	1179	2915222-2916395
YC40-M	7	IS1405	IS5	IS5	1180	968160-969333
YC40-M	8	IS1405	IS5	IS5	1180	2646789-2647962
YC40-M	9	IS1405	IS5	IS5	1185	1669895-1671068
YC40-M	10	IS1405	IS5	IS5	1178	1449890-1451063
YC40-M	11	IS1405	IS5	IS5	1186	2974416-2975589
YC40-M	12	IS1405	IS5	IS5	1180	3567405-3568578
YC40-M	1	IS1421	IS5	IS427	453	26135-26998
YC40-M	2	IS1421	IS5	IS427	453	3680540-3681397
YC40-M	3	IS1421	IS5	IS427	455	434215-435072
YC40-M	4	IS1421	IS5	IS427	453	3635102-3635959
YC40-M	5	IS1421	IS5	IS427	453	3719784- 3720641
YC40-M	6	IS1421	IS5	IS427	453	2957997- 2958854
YC40-M	7	IS1421	IS5	IS427	453	2921280-2922133
YC40-M	8	IS1421	IS5	IS427	453	3176367-3176522
YC40-M	1	ISRs01	IS5	-	838	2917870-2918753
YC40-M	2	ISRs01	IS5	-	842	1732860-1733743
YC40-M	1	ISRs018	IS5	IS5	1180	2863224-2864249
YC40-M	2	ISRs018	IS5	IS5	1138	3567411-3568436
YC40-M	3	ISRs018	IS5	IS5	965	2974422-2975447
YC40-M	4	ISRs018	IS5	IS5	1182	1450032-1451057
YC40-M	5	ISRs018	IS5	IS5	1025	1670037 -1671062
YC40-M	6	ISRs018	IS5	IS5	1182	2646931-2647956
YC40-M	7	ISRs018	IS5	IS5	964	968166-969191
YC40-M	8	ISRs018	IS5	IS5	1025	2915364-2916389
YC40-M	9	ISRs018	IS5	IS5	1179	615016-616041
YC40-M	10	ISRs018	IS5	IS5	1086	405536-406561
YC40-M	11	ISRs018	IS5	IS5	1098	3333349-3334374
YC40-M	12	ISRs018	IS5	IS5	1179	3603655-3604680
YC40-M	1	IS1021	IS5	IS5	1328	32685-33774
YC40-M	2	IS1021	IS5	IS5	1329	629991-631080
YC40-M	3	IS1021	IS5	IS5	2100	2845258-2846347
YC40-M	4	IS1021	IS5	IS5	1290	1056438- 1057528
YC40-M	5	IS1021	IS5	IS5	1214	2965219-2966308
YC40-M	6	IS1021	IS5	IS5	1214	3541476-3542565
YC40-M	7	IS1021	IS5	IS5	1216	2966837-2967927
YC40-M	8	IS1021	IS5	IS5	1216	913871-914961
YC40-M	9	IS1021	IS5	IS5	1215	1758509-1759599
YC40-M	10	IS1021	IS5	IS5	1214	898138-899228
YC40-M	11	IS1021	IS5	IS5	669	1746647-1747738
YC40-M	1	ISVei4	IS630	-	1132	2266806-2267885
SN82F48	1	ISBmu3	IS21	-	1550	14216-16912
SN82F48	2	ISMca5	IS256	-	1320	2760590-2761975
SN82F48	3	ISMca5	IS256	-	1320	1019500 -1020885
SN82F48	4	ISMca5	IS256	-	1318	1027631-1029016
SN82F48	5	ISMca5	IS256	-	1321	1714730- 1716115
SN82F48	6	ISMca5	IS256	-	1321	2746756-2748141

SN82F48	7	ISMca5	IS256	-	1327	2894359-2895744
SN82F48	8	ISMca5	IS256	-	1323	453839-455224
SN82F48	9	ISMca5	IS256	-	1324	211747-213132
SN82F48	1	ISCsp1	IS256	-	1264	3230173- 3231262
SN82F48	2	ISCsp1	IS256	-	1267	2760688-2761777
SN82F48	3	ISCsp1	IS256	-	1265	1019698-1020787
SN82F48	4	ISCsp1	IS256	-	1262	1027829-1028918
SN82F48	5	ISCsp1	IS256	-	1647	1714828-1715917
SN82F48	6	ISCsp1	IS256	-	1269	2746954-2748043
SN82F48	7	ISCsp1	IS256	-	1267	2894557-2895646
SN82F48	8	ISCsp1	IS256	-	1262	453937- 455026
SN82F48	1	ISPa54	IS5	IS5	980	392733-393699
SN82F48	1	ISPa40	Tn3	-	1173	1643396-1644786
SN82F48	2	ISPa40	Tn3	-	1183	1642419-1643398
SN83A39	2	ISBmu3	IS21	-	1550	2115854-2116350
SN83A39	1	IS408	IS21	-	1549	14218 -16367
SN83A39	1	ISButh3	IS256	-	1328	2450973-2452321
SN83A39	2	ISButh3	IS256	-	1328	3378132-3379270
SN83A39	3	ISButh3	IS256	-	1328	3366141-3366824
SN83A39	1	ISRso11	IS3	IS150	1466	817379-818827
SEPPX05	1	ISRs019	IS21	-	1877	3909574-3911529
SEPPX05	2	ISRs019	IS21	-	1883	335764-337719
SEPPX05	3	ISRs019	IS21	-	1884	358691-360646
SEPPX05	4	ISRs019	IS21	-	1882	3408257-3410212
SEPPX05	5	ISRs019	IS21	-	1880	592307-594262
SEPPX05	6	ISRs019	IS21	-	1880	608199-610154
SEPPX05	7	ISRs019	IS21	-	1881	3094221-3096176
SEPPX05	8	ISRs019	IS21	-	1882	2910413-2912368
SEPPX05	9	ISRs019	IS21	-	1880	2845464-2847419
SEPPX05	10	ISRs019	IS21	-	1883	1195175-1197130
SEPPX05	11	ISRs019	IS21	-	1884	1552523-1554478
SEPPX05	12	ISRs019	IS21	-	1883	2214559-2216514
SEPPX05	13	ISRs019	IS21	-	1884	2135600-2137555
SEPPX05	14	ISRs019	IS21	-	1884	2417247-2419202
SEPPX05	15	ISRs019	IS21	-	1878	2560166 -2562121
SEPPX05	16	ISRs019	IS21	-	1875	2655217-2657172
SEPPX05	17	ISRs019	IS21	-	1883	874759-876714
SEPPX05	18	ISRs019	IS21	-	1880	3137743-3139698
SEPPX05	19	ISRs019	IS21	-	1878	3202726-3204681
SEPPX05	20	ISRs019	IS21	-	1875	3338870-3340825
SEPPX05	21	ISRs019	IS21	-	1878	3518472-3520427
SEPPX05	22	ISRs019	IS21	-	1877	1451007-1452962
SEPPX05	23	ISRs019	IS21	-	1881	2053534 -2055489
SEPPX05	27	ISRs019	IS21	-	1880B	1849568-1851523
SEPPX05	25	ISRs019	IS21	-	1882	2487692 -2489647
SEPPX05	26	ISRs019	IS21	-	1881	484117 -486072
SEPPX05	27	ISRs019	IS21	-	1878	3670654-3672609
SEPPX05	28	ISRs019	IS21	-	1881	79818-81773
SEPPX05	29	ISRs019	IS21	-	2026	2147731-2149685

SEPPX05	30	ISRs019	IS21	-	1878	1332238- 1334193
SEPPX05	31	ISRs019	IS21	-	1854	1218634-1220483
SEPPX05	32	ISRs019	IS21	-	1565	1949030-1950695
SEPPX05	33	ISRs019	IS21	-	1320	1131702-1133121
SEPPX05	34	ISRs019	IS21	-	1148	3271799-3272996
SEPPX05	35	ISRs019	IS21	-	803	3268349-3269114
SEPPX05	36	ISRs019	IS21	-	569	1127260-1127800
SEPPX05	37	ISRs019	IS21	-	459	1439995 -1440499
SEPPX05	1	ISRme9	IS21	-	2360	3269115 -3271799
SEPPX05	2	ISRme9	IS21	-	2731	1441616-1444300
SEPPX05	3	ISRme9	IS21	-	2380	1929865-1932549
SEPPX05	4	ISRme9	IS21	-	2368	2339897-2342581
SEPPX05	5	ISRme9	IS21	-	2331	1199859-1202543
SEPPX05	6	ISRme9	IS21	-	2373	3103470-3106154
SEPPX05	7	ISRme9	IS21	-	2325	3514485-3517169
SEPPX05	8	ISRme9	IS21	-	2330	347147-349831
SEPPX05	9	ISRme9	IS21	-	2333	2904984-2907640
SEPPX05	10	ISRme9	IS21	-	2003	1127801-1130055
SEPPX05	1	ISRs011	IS3	IS150	1458	3770228-3771676
SEPPX05	2	ISRs011	IS3	IS150	1420	3908150- 3909573
SEPPX05	3	ISRs011	IS3	IS150	869	3139699-3140603
SEPPX05	4	ISRs011	IS3	IS150	571	2847420-2847969
SEPPX05	1	ISRs014	IS3	IS407	1168	3521968-3523201
SEPPX05	2	ISRs014	IS3	IS407	1169	2219631-2220864
SEPPX05	3	ISRs014	IS3	IS407	1163	1942034 -1943267
SEPPX05	4	ISRs014	IS3	IS407	1173	2574338-2575571
SEPPX05	5	ISRs014	IS3	IS407	1168	955762-956995
SEPPX05	6	ISRs014	IS3	IS407	1170	3261826-3263059
SEPPX05	7	ISRs014	IS3	IS407	1094	1220858-1221574
SEPPX05	8	ISRs014	IS3	IS407	683	2145328-2145968
SEPPX05	9	ISRs014	IS3	IS407	678	601120-601760
SEPPX05	10	ISRs014	IS3	IS407	676	596025-596665
SEPPX05	11	ISRs014	IS3	IS407	517	2147179-2147729
SEPPX05	12	ISRs014	IS3	IS407	517	594264-594814
SEPPX05	1	ISButh1	IS3	IS2	1330	2222653-2223982
SEPPX05	2	ISButh1	IS3	IS2	1330	3875973-3877302
SEPPX05	3	ISButh1	IS3	IS2	1327	916870-918199
SEPPX05	4	ISButh1	IS3	IS2	1083	3507271-3508583
SEPPX05	5	ISButh1	IS3	IS2	1080	3147527- 3148839
SEPPX05	6	ISButh1	IS3	IS2	1075	1504338-1505650
SEPPX05	7	ISButh1	IS3	IS2	1078	2030922-2032234
SEPPX05	8	ISButh1	IS3	IS2	1078	2139941-2141253
SEPPX05	9	ISButh1	IS3	IS2	1076	1454417-1455729
SEPPX05	10	ISButh1	IS3	IS2	1080	2572419-2573731
SEPPX05	11	ISButh1	IS3	IS2	1080	1215602-1216914
SEPPX05	12	ISButh1	IS3	IS2	1080	2907657-2908969
SEPPX05	13	ISButh1	IS3	IS2	1076	3513077-3514389
SEPPX05	14	ISButh1	IS3	IS2	1079	72139-73451
SEPPX05	15	ISButh1	IS3	IS2	1080	1307252-1308361
SEPPX05	16	ISButh1	IS3	IS2	748	357878- 358688
SEPPX05	1	ISRs016	IS3	IS407	658	1692252-1692910

SEPPX05	1	IS1405	IS5	IS5	1033	1496440-1497493
SEPPX05	2	IS1405	IS5	IS5	596	1554528-1555128
SEPPX05	3	IS1405	IS5	IS5	598	720497-721097
SEPPX05	4	IS1405	IS5	IS5	732	3204960-3205533
SEPPX05	5	IS1405	IS5	IS5	735	1550143 -1550716
SEPPX05	6	IS1405	IS5	IS5	736	722947-723520
SEPPX05	7	IS1405	IS5	IS5	533	486073-486611
SEPPX05	1	IS1421	IS5	IS407	931	878861-879724
SEPPX05	2	IS1421	IS5	IS407	871	1401002-1401865
SEPPX05	3	IS1421	IS5	IS407	871	958147-959006
SEPPX05	4	IS1421	IS5	IS407	870	663693-664552
SEPPX05	5	IS1421	IS5	IS407	872	3796778-3797637
SEPPX05	6	IS1421	IS5	IS407	888	1446075-1446934
SEPPX05	7	IS1421	IS5	IS407	901	1915302-1916161
SEPPX05	8	IS1421	IS5	IS407	870	3063471-3064330
SEPPX05	9	IS1421	IS5	IS407	872	1912022-1912885
SEPPX05	10	IS1421	IS5	IS407	870	880632-881495q
SEPPX05	1	IS881	IS5	IS5	1250	721066-722760
SEPPX05	2	IS881	IS5	IS5	1269	486580-488274
SEPPX05	3	IS881	IS5	IS5	1313	1550903-1552382
SEPPX05	4	IS881	IS5	IS5	1264	3201147-3202704
SEPPX05	1	ISRs018	IS5	IS5	1000	1496462-1497487
SEPPX05	2	ISRs018	IS5	IS5	692	722947-723514
SEPPX05	3	ISRs018	IS5	IS5	704	1550149-1550716
SEPPX05	4	ISRs018	IS5	IS5	693	3204960 -3205527
SEPPX05	2	ISRs017	IS701	-	1481	2404239-2405709
SEPPX05	1	ISRs017	IS701	-	1132	489002-490472
SEPPX05	1	ISRs015	ISL3	-	1322	1459217-1460520
SEPPX05	2	ISRs015	ISL3	-	1316	2438480-2439783
SEPPX05	3	ISRs015	ISL3	-	1321	2156869-2158172
SEPPX05	4	ISRs015	ISL3	-	1320	1910492-1911795
SEPPX05	5	ISRs015	ISL3	-	1322	1479560-1480863
SEPPX05	6	ISRs015	ISL3	-	1320	590397 -591700
SEPPX05	7	ISRs015	ISL3	-	1320	422407- 423710
SEPPX05	1	ISBma1	ISL3	-	1219	1459298 -1460520
SEPPX05	2	ISBma1	ISL3	-	1224	2438480-2439702
SEPPX05	3	ISBma1	ISL3	-	1225	2156869-2158091
SEPPX05	4	ISBma1	ISL3	-	1223	1910573-1911795
SEPPX05	5	ISBma1	ISL3	-	1219	1479560-1480782
SEPPX05	6	ISBma1	ISL3	-	1217	590397-591619
Rs-10-244	2	ISBma2	IS1182	-	1440	906989-908325
Rs-10-244	3	ISBma2	IS1182	-	1484	526869- 528205
Rs-10-244	4	ISBma2	IS1182	-	1574	2042987-2044323
Rs-10-244	5	ISBma2	IS1182	-	1576	2457599-2458935
Rs-10-244	6	ISBma2	IS1182	-	1489	1151672-1153010
Rs-10-244	1	ISBdo1	IS1182	-	1445	3561674-3563054
Rs-10-244	2	ISBdo1	IS1182	-	1484	943399-944779
Rs-10-244	3	ISBdo1	IS1182	-	1449	2025322-2026702
Rs-10-244	4	ISBdo1	IS1182	-	1484	2017452-2018832
Rs-10-244	5	ISBdo1	IS1182	-	1514	2993613-2994993

Rs-10-244	6	ISBdo1	IS1182	-	1486	914681-916061
Rs-10-244	7	ISBdo1	IS1182	-	1482	993049-994429
Rs-10-244	8	ISBdo1	IS1182	-	1630	2627696-2629076
Rs-10-244	9	ISBdo1	IS1182	-	1449	741975- 743356
Rs-10-244	10	ISBdo1	IS1182	-	1572	1019312-1020692
Rs-10-244	11	ISBdo1	IS1182	-	1443	2855409-2856789
Rs-10-244	12	ISBdo1	IS1182	-	1129	333971-335351
Rs-10-244	13	ISBdo1	IS1182	-	1588	2077989-2079369
Rs-10-244	14	ISBdo1	IS1182	-	1479	3572759-3574139
Rs-10-244	15	ISBdo1	IS1182	-	1446	2292143 -2292728
Rs-10-244	1	ISRs01	IS5	-	732	235599-236294
Rs-10-244	1	ISRs01	IS5	-	886	3659942-3660825
Rs-09-161	1	ISButh1	IS3	IS2	970	2354094-2355064
Rs-09-161	1	ISRs012	IS3	IS407	422	2044436-2044858
Rs-09-161	2	ISRs012	IS3	IS407	422	925912-926334
Rs-09-161	1	ISRs016	IS3	IS407	1148	2652695-2653933
Rs-09-161	1	ISRs018	IS5	IS5	975	3615571-3616546
Rs-09-161	1	IS1405	IS5	IS5	973	3615574-3616546
SL3103	2	ISRs011	IS3	IS150	1473	926902-928346
SL3103	1	ISRs010	IS3	IS2	970	231197- 232171
SL3103	2	ISRs010	IS3	IS2	360	229473-229831
SL3103	1	IS1420	IS5	IS903	1130	209027-210157
SL3103	2	IS1420	IS5	IS903	1130	3230782-3231912
SL3103	3	IS1420	IS5	IS903	1130	315784-316914
SL3103	4	IS1420	IS5	IS903	1130	2541245-2542375
SL3103	5	IS1420	IS5	IS903	1131	1471134- 1472264
SL3103	6	IS1420	IS5	IS903	1130	1897633 -1898763
SL3103	7	IS1420	IS5	IS903	1130	1551378-1552508
SL3103	8	IS1420	IS5	IS903	1130	2437560-2438690
SL3103	9	IS1420	IS5	IS903	1130	2829985-2831115
SL3103	10	IS1420	IS5	IS903	1130	3359229-3360359
SL3103	11	IS1420	IS5	IS903	1130	19492-20622
SL3103	12	IS1420	IS5	IS903	1130	9252-10382
SL3103	1	IS1405	IS5	IS5	1184	314216-315389
SL3103	2	IS1405	IS5	IS5	1183	3093303- 3094476
SL3103	3	IS1405	IS5	IS5	1184	2581038-2582211
SL3103	4	IS1405	IS5	IS5	1183	2882128-2883301
SL3103	5	IS1405	IS5	IS5	1181	2606922 -2608095
SL3103	6	IS1405	IS5	IS5	1184	13545-14718
SL3103	7	IS1405	IS5	IS5	1182	210461-211634
SL3103	8	IS1405	IS5	IS5	1185	289071-290244
SL3103	9	IS1405	IS5	IS5	1180	3167710-3168883
SL3103	10	IS1405	IS5	IS5	1184	1381967-1383140
SL3103	11	IS1405	IS5	IS5	1183	3275250-3276423
SL3103	1	IS1421	IS5	IS427	861	6803-7666
SL3103	2	IS1421	IS5	IS427	874	2594328-2595186
SL3103	1	ISRs01	IS5	-	877	3256317-3257200
SL3103	2	ISRs01	IS5	-	881	248558-249441
SL3103	3	ISRs01	IS5	-	887	291106-291989

SL3103	4	ISRs01	IS5	-	885	1780369-1781252
SL3103	1	ISRs018	IS5	IS5	965	2606928-2607953
SL3103	2	ISRs018	IS5	IS5	976	2882270-2883295
SL3103	3	ISRs018	IS5	IS5	1051	3275256-3276281
SL3103	4	ISRs018	IS5	IS5	1020	2581044- 2582069
SL3103	5	ISRs018	IS5	IS5	985	1382109-1383134
SL3103	6	ISRs018	IS5	IS5	981	3093445-3094470
SL3103	7	ISRs018	IS5	IS5	979	3167852-3168877
SL3103	8	ISRs018	IS5	IS5	967	314222-315247
SL3103	9	ISRs018	IS5	IS5	1087	289077-290102
SL3103	10	ISRs018	IS5	IS5	965	210467-211492
SL3103	1	IS1021	IS5	IS5	1217	820682-821771
SL3103	2	IS1021	IS5	IS5	1214	2605361-2606450
SL3103	3	IS1021	IS5	IS5	1214	25789-26878
SL3103	4	IS1021	IS5	IS5	876	3100126-3100877
SL3103	1	ISRs017	IS701	-	1481	3031294-3032764
T117	2	ISRs011	IS3	IS150	1371	964393-965841
T117	1	ISRs016	IS3	IS407	1219	2693695-2694932
T117	1	IS1405	IS5	IS5	1182	288574-289747
T117	2	IS1405	IS5	IS5	1181	1399619-1400792
T117	3	IS1405	IS5	IS5	1036	1273729-1274902
T117	4	IS1405	IS5	IS5	1184	1210383 -1211556
T117	5	IS1405	IS5	IS5	1182	2541702-2542875
T117	6	IS1405	IS5	IS5	1182	3412734-3413907
T117	7	IS1405	IS5	IS5	1182	346765-347938
T117	1	ISRs01	IS5	-	882	3620256-3621139
T117	2	ISRs01	IS5	-	884	1798437-1799320
T117	3	ISRs01	IS5	-	842	1271371- 1272254
T117	1	ISRs018	IS5	IS5	1183	3412740-3413765
T117	2	ISRs018	IS5	IS5	1184	2541708-2542733
T117	3	ISRs018	IS5	IS5	1333	1210525-1211550
T117	4	ISRs018	IS5	IS5	978	1273871-1274896
T117	5	ISRs018	IS5	IS5	1181	1399625-1400650
T117	6	ISRs018	IS5	IS5	960	346771-347796
T117	7	ISRs018	IS5	IS5	1183	288716-289741
T117	1	IS1021	IS5	IS5	1216	1274907-1275997
T117	2	IS1021	IS5	IS5	1614	3464051-3465141
T117	3	IS1021	IS5	IS5	1486	2978737-2979598
SL2330	1	ISBma3	IS110	-	1260	1701774-1703229
SL2330	1	ISRs011	IS3	IS150	1468	383766-385214
SL2330	1	ISRs014	IS3	IS407	1176	3459400-3460633
SL2330	1	ISRs016	IS3	IS407	212	3236599-3237256
SL2330	1	ISRs011	IS3	IS150	1481	1900821-1902269
SL2330	1	IS1405	IS5	IS5	1185	6009-7182
SL2330	2	IS1405	IS5	IS5	1183	341719- 342892
SL2330	1	IS1421	IS5	IS427	871	1068024-1068883
SL2330	1	ISRs01	IS5	-	842	2736087-2736970
SL2330	2	ISRs01	IS5	-	884	2780769-2781652
SL2330	3	ISRs01	IS5	-	886	1915028-1915911

SL2330	4	ISRs01	IS5	-	886	1337037-1337920
SL2330	5	ISRs01	IS5	-	885	2804239-2805122
SL2330	6	ISRs01	IS5	-	886	3454663-3455546
SL2330	7	ISRs01	IS5	-	841	1695821-1696704
SL2330	8	ISRs01	IS5	-	1042	2781761-2782645
SL2330	9	ISRs01	IS5	-	840	2806123-2807006
SL2330	1	ISRs018	IS5	IS5	1179	341725-342750
SL2330	2	ISRs018	IS5	IS5	1166	6015-7040
SL2330	2	ISRs017	IS701	-	1081	3455551 -3457021
SL3755	1	ISBma3	IS110	-	1271	1678305-1679760
SL3755	1	ISRs011	IS3	IS150	1470	352332-353780
SL3755	1	ISRs014	IS3	IS407	1170	3573322-3574555
SL3755	1	ISRs016	IS3	IS407	212	3332137-3332794
SL3755	1	ISRs011	IS3	IS150	1451	2145623-2147071
SL3755	1	IS1405	IS5	IS5	1181	310285-311458
SL3755	2	IS1405	IS5	IS5	1181	287238-288411
SL3755	3	IS1405	IS5	IS5	1185	2745570-2746743
SL3755	1	ISRs01	IS5	-	843	2833364-2834247
SL3755	2	ISRs01	IS5	-	887	1891512-1892395
SL3755	3	ISRs01	IS5	-	883	1314316-1315199
SL3755	4	ISRs01	IS5	-	887	2901509-2902392
SL3755	5	ISRs01	IS5	-	885	3568585-3569468
SL3755	6	ISRs01	IS5	-	885	2878040-2878923
SL3755	7	ISRs01	IS5	-	840	1672353-1673236
SL3755	8	ISRs01	IS5	-	1041	2900516-2901400
SL3755	9	ISRs01	IS5	-	777	264312-265194
SL3755	10	ISRs01	IS5	-	841	2903393-2904276
SL3755	1	ISRs018	IS5	IS5	1268	2745576-2746601
SL3755	2	ISRs018	IS5	IS5	1251	287244-288269
SL3755	3	ISRs018	IS5	IS5	1183	310291-311316
SL3755	2	ISRs017	IS701	-	1482	3128613-3130083
T78	2	ISRs011	IS3	IS150	1468	1012415-1013863
T78	1	IS1420	IS5	IS903	1131	1867294-1868424
T78	1	IS1405	IS5	IS5	1185	304793-305966
T78	2	IS1405	IS5	IS5	1180	3482929-3484102
T78	3	IS1405	IS5	IS5	1181	2698327-2699500
T78	4	IS1405	IS5	IS5	1181	2517686-2518859
T78	5	IS1405	IS5	IS5	1181	1469084-1470257
T78	6	IS1405	IS5	IS5	1184	2902234-2903407
T78	7	IS1405	IS5	IS5	1185	2965576-2966749
T78	8	IS1405	IS5	IS5	1183	379625- 380798
T78	9	IS1405	IS5	IS5	1182	3624541-3625714
T78	1	ISRs01	IS5	-	883	3831702-3832585
T78	2	ISRs01	IS5	-	883	2256154-2257037
T78	3	ISRs01	IS5	-	840	2904882-2905765
T78	1	ISRs018	IS5	IS5	1181	3624547-3625572
T78	2	ISRs018	IS5	IS5	1170	379767-380792
T78	3	ISRs018	IS5	IS5	1331	2965582-2966607
T78	4	ISRs018	IS5	IS5	1316	2902240-2903265

T78	5	ISRs018	IS5	IS5	1183	1469226-1470251
T78	6	ISRs018	IS5	IS5	1335	2517828-2518853
T78	7	ISRs018	IS5	IS5	1181	2698469- 2699494
T78	8	ISRs018	IS5	IS5	963	3483071-3484096
T78	9	ISRs018	IS5	IS5	970	304799-305824
T78	1	IS1021	IS5	IS5	1183	2901139-2902229
T78	2	IS1021	IS5	IS5	1618	3675856-3676946
T78	3	IS1021	IS5	IS5	1228	400582-401672
T78	1	ISRs017	IS701	-	1482	3569473-3570943
SL3730	2	ISRs011	IS3	IS150	1487	951826-953273
SL3730	1	ISRs011	IS3	IS150	1477	2145823-2147271
SL3730	1	IS1405	IS5	IS5	1185	245580-246753
SL3730	2	IS1405	IS5	IS5	1187	3441648-3442821
SL3730	3	IS1405	IS5	IS5	1186	305234-306407
SL3730	4	IS1405	IS5	IS5	1187	3320952-3322125
SL3730	5	IS1405	IS5	IS5	1183	2782520-2783693
SL3730	6	IS1405	IS5	IS5	1183	2647089-2648262
SL3730	7	IS1405	IS5	IS5	1183	1649512-1650685
SL3730	8	IS1405	IS5	IS5	1182	1483329-1484502
SL3730	1	IS1420	IS5	IS903	1131	696939-698069
SL3730	2	IS1420	IS5	IS903	1133	3001800-3002930
SL3730	3	IS1420	IS5	IS903	1128	838503-839633
SL3730	4	IS1420	IS5	IS903	1129	3004363-3005493
SL3730	5	IS1420	IS5	IS903	1131	3044193-3045323
SL3730	6	IS1420	IS5	IS903	1130	3392938- 3394068
SL3730	7	IS1420	IS5	IS903	1127	14884-16014
SL3730	1	ISRs01	IS5	-	825	1486105-1486988
SL3730	2	ISRs01	IS5	-	884	2248577-2249460
SL3730	1	ISRs018	IS5	IS5	1182	3381686-3382711
SL3730	2	ISRs018	IS5	IS5	1333	2764639-2765664
SL3730	3	ISRs018	IS5	IS5	1330	1483471-1484496
SL3730	4	ISRs018	IS5	IS5	964	1649654-1650679
SL3730	5	ISRs018	IS5	IS5	1184	2647231-2648256
SL3730	6	ISRs018	IS5	IS5	1179	2782662-2783687
SL3730	7	ISRs018	IS5	IS5	1183	3321094-3322119
SL3730	8	ISRs018	IS5	IS5	965	305240-306265
SL3730	1	IS1021	IS5	IS5	1219	3312263-3313353
SL3730	2	IS1021	IS5	IS5	1354	724044-725134
SL3730	3	IS1021	IS5	IS5	1217	848116-849206
SL3730	4	IS1021	IS5	IS5	1216	3335354-3336444
SL3730	5	IS1021	IS5	IS5	1213	3505504-3506594
12D	1	ISRme4	IS21	-	1629	1092500-1094966
12D	1	ISRs07	IS256	-	1337	266108-267451
12D	2	ISRs07	IS256	-	1344	3352031-3353374
12D	3	ISRs07	IS256	-	1344	1346726-1348069
12D	4	ISRs07	IS256	-	1344	752839-754182
12D	5	ISRs07	IS256	-	1344	3308687-3310030
12D	6	ISRs07	IS256	-	1344	92520-93863
12D	7	ISRs07	IS256	-	1306	9590-10926

12D	1	IS1090	IS256	-	1351	9591-10917
12D	2	IS1090	IS256	-	1350	266121-267447
12D	3	IS1090	IS256	-	1349	3352035-3353361
12D	4	IS1090	IS256	-	1344	1346730-1348056
12D	5	IS1090	IS256	-	1349	752843-754169
12D	6	IS1090	IS256	-	1347	3308700-3310026
12D	7	IS1090	IS256	-	1344	92524-93850
12D	1	ISMca2	IS3	IS3	1999	662847-664142
12D	1	ISCte2	IS3	IS3	1999	662841-664142
12D	1	ISPsy42	-	-	3057	1567621-1570675
DTP0602	1	ISAba30	IS200/IS605	IS605	1790	4460307-4462083
DTP0602	2	ISAba30	IS200/IS605	IS605	1788	4266768-4268544
DTP0602	3	ISAba30	IS200/IS605	IS605	1788	3395714-3397473
DTP0602	4	ISAba30	IS200/IS605	IS605	1782	240278-242034
DTP0602	1	ISMca5	IS256	-	3947	3230075-3231460
DTP0602	1	ISRta2	IS256	-	1250	3267123-3268282
DTP0602	1	ISRme1	IS5	-	1014	1890291-1891285
DTP0602	1	ISRme16	IS5	-	1014	1890292-1891285
SL2729	2	ISRs011	IS3	IS150	1502	951912-953360
SL2729	1	ISRs016	IS3	IS407	1140	2635202-2636439
SL2729	1	IS1405	IS5	IS5	1186	245615-246788
SL2729	2	IS1405	IS5	IS5	1186	3439538-3440711
SL2729	3	IS1405	IS5	IS5	1184	305289-306462
SL2729	4	IS1405	IS5	IS5	1182	3319977- 3321150
SL2729	5	IS1405	IS5	IS5	1184	2782743-2783916
SL2729	6	IS1405	IS5	IS5	1182	2647300-2648473
SL2729	7	IS1405	IS5	IS5	1184	1649626-1650799
SL2729	8	IS1405	IS5	IS5	1174	1483440 -1484613
SL2729	9	IS1405	IS5	IS5	1183	2764857-2766030
SL2729	10	IS1405	IS5	IS5	1184	3380708-3381881
SL2729	1	IS1420	IS5	IS903	1130	697017-698147
SL2729	2	IS1420	IS5	IS903	1131	3002024-3003154
SL2729	3	IS1420	IS5	IS903	1134	838585-839715
SL2729	4	IS1420	IS5	IS903	1128	3004587-3005717
SL2729	5	IS1420	IS5	IS903	1132	3044421-3045551
SL2729	6	IS1420	IS5	IS903	1134	14885-16015
SL2729	1	ISRs01	IS5	-	882	1486216-1487099
SL2729	2	ISRs01	IS5	-	883	2248781-2249664
SL2729	1	ISRs018	IS5	IS5	1181	3380714-3381739
SL2729	2	ISRs018	IS5	IS5	1331	2764863-2765888
SL2729	3	ISRs018	IS5	IS5	1184	1483582-1484607
SL2729	4	ISRs018	IS5	IS5	962	1649768-1650793
SL2729	5	ISRs018	IS5	IS5	1184	2647442-2648467
SL2729	6	ISRs018	IS5	IS5	1185	2782885- 2783910
SL2729	7	ISRs018	IS5	IS5	1182	3320119-3321144
SL2729	8	ISRs018	IS5	IS5	967	305295-306320
SL2729	9	ISRs018	IS5	IS5	1215	3439680-3440705
SL2729	10	ISRs018	IS5	IS5	1179	245757 -246782
SL2729	1	IS1021	IS5	IS5	1356	724123-725213

SL2729	2	IS1021	IS5	IS5	1213	848198-849288
T110	1	ISRso17	IS701		1471	3002470-3003939
T110	1	ISRso11	IS3	IS150	1449	356097-357542
T110	1	ISRso14	IS3	IS407	1218	3428665-3429881
T110	1	IS1405	IS5	IS5	1174	314094-315266
T110	2	IS1405	IS5	IS5	1174	1401819-1402991
T110	3	IS1405	IS5	IS5	1174	3448126-3449297
T110	4	IS1405	IS5	IS5	1174	3302449-3303619
T110	1	IS1421	IS5	IS427	860	3612943-3613802
T110	2	IS1421	IS5	IS427	860	1039803-1040661
T110	1	ISRso1	IS5		884	2707501-2708384
T110	2	ISRso1	IS5		884	1887307-1888190
T110	3	ISRso1	IS5		884	1308616-1309499
T110	4	ISRso1	IS5		884	2775584-2776467
T110	5	ISRso1	IS5		884	3425399-3426282
T110	6	ISRso1	IS5		884	2752134-2753017
T110	7	ISRso1	IS5		884	1668275-1669157
T110	8	ISRso1	IS5		885	2774591-2775475
T110	9	ISRso1	IS5		884	2777468-2778351
T110	1	ISRso18	IS5	IS5	1026	314100-315125
T110	2	ISRso18	IS5	IS5	1026	3448267-3449291
T110	3	ISRso18	IS5	IS5	1026	1401825-1402849
T110	4	ISRso18	IS5	IS5	1026	3302455-3303478
T110	1	ISBma3	IS110		1459	1674225-1675678
T110	1	ISRso16	IS3	IS407	662	3207574-3208231
RS_T51	1	IS1421	IS5	IS427	924	9055-9846
RS_T51	1	ISRs016	IS3	IS407	1145	2653122-2654360
SL3175	2	ISRs016	IS3	IS407	1220	2920756-2921993
SL3175	3	ISRs016	IS3	IS407	511	219397-219985
SL3175	1	ISRs014	IS3	IS407	1210	548724-549957
SL3175	2	ISRs014	IS3	IS407	1172	370061-371294
SL3175	3	ISRs014	IS3	IS407	1283	2551770-2553005
SL3175	4	ISRs014	IS3	IS407	1117	1470113-1471346
SL3175	5	ISRs014	IS3	IS407	1186	2702108-2703343
SL3175	1	IS407	IS3	IS407	1200	548758-549824
SL3175	2	IS407	IS3	IS407	1328	370194- 371260
SL3175	3	IS407	IS3	IS407	1403	2551903-2552971
SL3175	4	IS407	IS3	IS407	1477	1470246-1471312
SL3175	1	ISRs011	IS3	IS150	1467	352692-354140
SL3175	1	IS407	IS3	IS407	1200	548758-549824
SL3175	2	IS407	IS3	IS407	1328	370194- 371260
SL3175	3	IS407	IS3	IS407	1403	2551903-2552971
SL3175	4	IS407	IS3	IS407	1477	1470246-1471312
T11	1	IS1421	IS5	IS427	911	9055-9846
T11	1	ISRs016	IS3	IS407	1219	2693712-2694949
T98	2	ISRs016	IS3	IS407	1219	2920744-2921981

T98	3	ISRs016	IS3	IS407	508	219394-219982
T98	1	ISRs014	IS3	IS407	1188	548720-549953
T98	2	ISRs014	IS3	IS407	1118	370058-371291
T98	3	ISRs014	IS3	IS407	1189	2551754-2552989
T98	4	ISRs014	IS3	IS407	1118	1470108-1471341
T98	5	ISRs014	IS3	IS407	1117	2702091-2703326
T98	1	IS407	IS3	IS407	1199	548754-549820
T98	2	IS407	IS3	IS407	1328	370191-371257
T98	3	IS407	IS3	IS407	1606	2551887-2552955
T98	4	IS407	IS3	IS407	1120	1470241-1471307
T98	1	ISRs011	IS3	IS150	1483	2153180-2154628
T12	-	-	-	-	-	-
SL3022						
SL2064	1	IS1421	IS5	IS427	848	9055-9846
SL2064	1	ISRs016	IS3	IS407	1144	2635163-2636400
SL2312	1	ISRs012	IS3	IS407	1055	754405-755292
SL2312	2	ISRs012	IS3	IS407	1066	1581180-1582049
SL2312	1	ISRs014	IS3	IS407	671	742673-743457
SL2312	1	ISRs016	IS3	IS407	1145	2705805 -2707043
SL2312	1	ISRs01	IS5	-	883	755289-756172
T101	1	ISRs012	IS3	IS407	732	754351-755238
T101	2	ISRs012	IS3	IS407	1064	1581115-1581984
T101	1	ISRs016	IS3	IS407	1147	2706693-2707931
T101	1	ISRs01	IS5	-	882	755235-756118
T82	1	ISRs012	IS3	IS407	949	754405-755292
T82	2	ISRs012	IS3	IS407	1064	1581141-1582010
T82	1	ISRs014	IS3	IS407	672	742672- 743456
T82	1	ISRs011	IS3	IS150	1448	2145779-2147227
T82	1	ISRs01	IS5	-	883	755289-756172
T95	1	IS1421	IS5	IS427	912	9055-9846
T95	1	ISRs016	IS3	IS407	1146	2635202-2636439
12J	2	ISPsy42	-	-	3067	1941890-1944944
12J	1	ISRme15	IS3	IS51	1404	1917829-1919153
12J	2	ISRme15	IS3	IS51	1420	10873 -12197
12J	1	ISPsy30	Tn3	-	3027	1941890-1944924
12J	2	ISPsy30	Tn3	-	3027	1567621-1570655
12J	1	ISStma10	IS630	-	1054	1235090- 1236194
12J	2	ISStma10	IS630	-	1077	1851010-1852114
12J	3	ISStma10	IS630	-	1071	1580667-1581771
12J	4	ISStma10	IS630	-	1076	2873456-2874560
12J	1	IS401	IS3	IS51	1177	1215881-1216766
12J	1	TnAs2	Tn3	-	1690	1882687-1884174
12J	1	TnAs3	Tn3	-	1696	1882684-1884118

12J	1	ISStma11	ISL3	-	1071	1882687-1884067
ACTC49129_1						
ACTC49129_2						
A2- HR	1	ISBcen18	IS256	-	1315	3150831-3152196
A2- HR	2	ISBcen18	IS256	-	1314	756199-757564
A2- HR	3	ISBcen18	IS256	-	1314	2687629-2688994
A2- HR	4	ISBcen18	IS256	-	1315	1021024-1022389
A2- HR	5	ISBcen18	IS256	-	1317	1089839-1091204
A2- HR	6	ISBcen18	IS256	-	1317	1307080-1308445
A2- HR	7	ISBcen18	IS256	-	1318	2289693-2291058
A2- HR	8	ISBcen18	IS256	-	1316	2181734-2183099
A2- HR	9	ISBcen18	IS256	-	1315	1526104-1527469
A2- HR	10	ISBcen18	IS256	-	1315	1622557-1623922
A2- HR	11	ISBcen18	IS256	-	1320	1875368-1876733
A2- HR	12	ISBcen18	IS256	-	1321	1873816-1875181
A2- HR	13	ISBcen18	IS256	-	1321	2043205-2044570
A2- HR	14	ISBcen18	IS256	-	1319	1456461-1457826
A2- HR	1	ISRme15	IS3	IS51	1421	771654-772978
A2- HR	2	ISRs010	IS3	IS2	1334	2946126-2947460
A2- HR	3	ISRs010	IS3	IS2	1334	3600071-3601405
A2- HR	4	ISRs010	IS3	IS2	1334	2691013-2692347
A2- HR	5	ISRs010	IS3	IS2	1334	2523161-2524495
A2- HR	6	ISRs010	IS3	IS2	1334	689885-691219
A2- HR	7	ISRs010	IS3	IS2	1334	1086661-1087874
A2- HR	8	ISRs010	IS3	IS2	1334	2287699-2288912
A2- HR	9	ISRs010	IS3	IS2	1334	3290876-3292045
A2- HR	10	ISRs010	IS3	IS2	1334	1539035- 1540189
A2- HR	11	ISRs010	IS3	IS2	1334	136064-137218
A2- HR	12	ISRs010	IS3	IS2	1334	2374589-2375113
A2- HR	1	ISRs016	IS3	IS407	1237	3555842-3557079
A2- HR	2	ISRs016	IS3	IS407	1237	612769-614006
A2- HR	3	ISRs016	IS3	IS407	1237	831491-832728
A2- HR	4	ISRs016	IS3	IS407	1237	1925074-1925642
A2- HR	1	ISRs09	IS5	IS5	1294	3286249 -3287543
A2- HR	2	ISRs09	IS5	IS5	1294	2948622-2949916
A2- HR	3	ISRs09	IS5	IS5	1294	1113062 -1114356
A2- HR	4	ISRs09	IS5	IS5	1294	2206044-2207338
A2- HR	5	ISRs09	IS5	IS5	1294	2045375-2046669
A2- HR	1	ISButh4	IS5	IS5	1319	3286247-3287544
A2- HR	2	ISButh4	IS5	IS5	1319	2948620-2949917
A2- HR	3	ISButh4	IS5	IS5	1319	1113061-1114358
A2- HR	4	ISButh4	IS5	IS5	1319	2206042-2207339
A2- HR	5	ISButh4	IS5	IS5	1319	2045373-2046670
A2- HR	6	ISButh4	IS5	IS5	1319	1495535-1496832
A2- HR	7	ISButh4	IS5	IS5	1319	1418571-1419868
A2- HR	8	ISButh4	IS5	IS5	1319	2203802-2205099
A2- HR	9	ISButh4	IS5	IS5	1319	2259540-2260837
A2- HR	10	ISButh4	IS5	IS5	1319	1298986-1300283

A2- HR	11	ISButh4	IS5	IS5	1319	2673293-2674590
A2- HR	12	ISButh4	IS5	IS5	1319	822969-824266
A2- HR	13	ISButh4	IS5	IS5	1319	3433587-3434884
A2- HR	14	ISButh4	IS5	IS5	1319	2698498-2699795
PSI07	2	ISRs010	IS3	IS2	1345	2611703-2613023
PSI07	3	ISRs010	IS3	IS2	1345	1558152-1559472
PSI07	4	ISRs010	IS3	IS2	1345	1653289-1654610
PSI07	1	ISRs016	IS3	IS407	1224	1512826 -1514063
PSI07	2	ISRs016	IS3	IS407	I224	2614088-2615325
PSI07	3	ISRs016	IS3	IS407	1144	3103951 -3105188
PSI07	4	ISRs016	IS3	IS407	1175	2856637-2857875
PSI07	5	ISRs016	IS3	IS407	1224	2643518-2644755
PSI07	6	ISRs016	IS3	IS407	599	298701 -299289
PSI07	1	ISRs014	IS3	IS407	1187	1655994 -1657227
PSI07	2	ISRs014	IS3	IS407	1186	3512111- 3513344
PSI07	3	ISRs014	IS3	IS407	1120	1549247- 1550480
PSI07	4	ISRs014	IS3	IS407	582	823244- 824021
PSI07	1	IS407	IS3	IS407	1066	1656028-1657094
PSI07	2	IS407	IS3	IS407	1066	1549281-1550347
PSI07	3	IS407	IS3	IS407	1066	3512244-3513310
PSI07	1	ISRs020	IS3	IS3	1288	2296720 -2298006
PSI07	1	IS1421	IS5	IS427	653	1641020-1641636
PSI07	1	ISRs01	IS5	-	886	1517841-1518724
KACC10722	1	IS1421	IS5	IS427	775	9055-9846
KACC10722	1	ISRs019	IS21	-	1995	424936-426891

Table S3c. Characterization of Insertion sequences elements found in the chromosome of *Ralstonia* spp. with OASIS

Strain	IS name	ORF Right End	ORF Left End	Family	Group
GMI1000	IS_f1ddcc0a	127828	128994	family "IS630"	group "-"
GMI1000	IS_f1ddcc0a	683357	684523	family "IS630"	group "-"
GMI1000	IS_f1ddcc0a	2360110	2361276	family "IS630"	group "-"
GMI1000	IS_f1ddcc0a	3659384	3660550	family "IS630"	group "-"
GMI1000	IS_f1ddcc0a	117917	119083	family "IS630"	group "-"
GMI1000	IS_f1ddcc0a	275161	277178	family "IS21"	group "-"
GMI1000	IS_f1ddcc0a	2531579	2533596	family "IS21"	group "-"
GMI1000	IS_f1ddcc0a	622263	623517	family "IS3"	group "-"
GMI1000	IS_f1ddcc0a	1661741	1662995	family "IS3"	group "-"
GMI1000	IS_f1ddcc0a	2458049	2459303	family "IS3"	group "-"
GMI1000	IS_f1ddcc0a	869921	871154	family "IS3"	group "IS407"
GMI1000	IS_f1ddcc0a	2611668	2612901	family "IS3"	group "IS407"
GMI1000	IS_f1ddcc0a	1596962	1598195	family "IS3"	group "IS407"
GMI1000	IS_f1ddcc0a	883209	884695	family "IS5"	group "IS5"
GMI1000	IS_f1ddcc0a	3365733	3367219	family "IS5"	group "IS5"
GMI1000	IS_f1ddcc0a	3395321	3396807	family "IS5"	group "IS5"
GMI1000	IS_f1ddcc0a	3695664	3697149	family "IS5"	group "IS5"
GMI1000	IS_f1ddcc0a	1538928	1540122	family "IS3"	group "IS2"
GMI1000	IS_f1ddcc0a	2001514	2002708	family "IS3"	group "IS2"
GMI1000	IS_f1ddcc0a	2530436	2531572	family "IS3"	group "IS2"
GMI1000	IS_f1ddcc0a	2613222	2614670	family "IS3"	group "IS150"
GMI1000	IS_f1ddcc0a	3326411	3327859	family "IS3"	group "IS150"
GMI1000	IS_f1ddcc0a	1540157	1541605	family "IS3"	group "IS150"
GMI1000	IS_f1ddcc0a	2456643	2458033	family "IS3"	group "IS150"
GMI1000	IS_f1ddcc0a	1541741	1542968	family "IS3"	group "IS407"
GMI1000	IS_f1ddcc0a	2910961	2912185	family "IS3"	group "IS407"
GMI1000	IS_f1ddcc0a	1589423	1590891	family "IS4"	group "IS4"
GMI1000	IS_f1ddcc0a	2047823	2049291	family "IS4"	group "IS4"
GMI1000	IS_f1ddcc0a	3498806	3500272	family "IS4"	group "IS4"
GMI1000	IS_f1ddcc0a	3427433	3428899	family "IS4"	group "IS4"
GMI1000	IS_f1ddcc0a	3429678	3432578	family "Tn3"	group ""
GMI1000	IS_f1ddcc0a	3502101	3504838	family "Tn3"	group ""
GMI1000	IS_f1ddcc0a	282790	284123	family "IS256"	group "-"
GMI1000	IS_f1ddcc0a	934789	936735	family "None"	group "None"
GMI1000	IS_f1ddcc0a	2780072	2781377	family "ISL3"	group "-"
GMI1000	IS_f1ddcc0a	3483307	3484192	family "IS5"	group "-"
GMI1000	IS_f1ddcc0a	231870	232755	family "IS5"	group "-"
GMI1000	IS_f1ddcc0a	3612023	3612908	family "IS5"	group "-"
GMI1000	IS_f1ddcc0a	1059500	1060385	family "IS5"	group "-"
GMI1000	IS_f1ddcc0a	3460578	3461463	family "IS5"	group "-"
Po82	IS_b668b82c.1	380846	382732	family "IS21"	group "-"
Po82	IS_b668b82c.1	1610811	1612697	family "IS21"	group "-"
Po82	IS_b668b82c.1	2854449	2856335	family "IS21"	group "-"
Po82	IS_b668b82c.1	1171869	1173755	family "IS21"	group "-"
Po82	IS_b668b82c.1	2646622	2648508	family "IS21"	group "-"
Po82	IS_b668b82c.1	1695548	1697434	family "IS21"	group "-"
Po82	IS_b668b82c.1	2643526	2644876	family "IS3"	group "IS2"
Po82	IS_b668b82c.1	1606602	1607938	family "IS3"	group "IS2"
Po82	IS_b668b82c.1	303404	304739	family "IS3"	group "IS2"
Po82	IS_b668b82c.1	1605094	1606429	family "IS3"	group "IS2"
Po82	IS_b668b82c.1	2922032	2923367	family "IS3"	group "IS2"
Po82	IS_b668b82c.1	2937657	2938992	family "IS3"	group "IS2"
Po82	IS_b668b82c.1	2850260	2851455	family "IS3"	group "IS407"
Po82	IS_b668b82c.1	2924136	2925331	family "IS3"	group "IS407"
Po82	IS_b668b82c.1	1591623	1592817	family "IS3"	group "IS407"
Po82	IS_b668b82c.1	1356668	1357986	family "IS3"	group "IS51"
Po82	IS_b668b82c.1	2866276	2867594	family "IS3"	group "IS51"
Po82	IS_b668b82c.1	1694605	1695518	family "IS3"	group "IS51"
Po82	IS_b668b82c.1	1607939	1608677	family "IS3"	group "IS51"

UY081	IS_cc12ea8f.1	50545	51761	family "IS5"	group "IS5"
UY081	IS_cc12ea8f.1	74877	76093	family "IS5"	group "IS5"
UY081	IS_cc12ea8f.1	269471	270687	family "IS5"	group "IS5"
UY081	IS_cc12ea8f.1	308621	309837	family "IS5"	group "IS5"
UY081	IS_cc12ea8f.1	320153	321369	family "IS5"	group "IS5"
UY081	IS_cc12ea8f.1	343700	344916	family "IS5"	group "IS5"
UY081	IS_cc12ea8f.1	1051464	1052680	family "IS5"	group "IS5"
UY081	IS_cc12ea8f.1	1162772	1163988	family "IS5"	group "IS5"
UY081	IS_cc12ea8f.1	1237642	1238858	family "IS5"	group "IS5"
UY081	IS_cc12ea8f.1	1257029	1258245	family "IS5"	group "IS5"
UY081	IS_cc12ea8f.1	1437947	1439163	family "IS5"	group "IS5"
UY081	IS_cc12ea8f.1	2388921	2390137	family "IS5"	group "IS5"
UY081	IS_cc12ea8f.1	2392688	2393904	family "IS5"	group "IS5"
UY081	IS_cc12ea8f.1	2427173	2428389	family "IS5"	group "IS5"
UY081	IS_cc12ea8f.1	2514718	2515934	family "IS5"	group "IS5"
UY081	IS_cc12ea8f.1	2580111	2581327	family "IS5"	group "IS5"
UY081	IS_cc12ea8f.1	2707772	2708988	family "IS5"	group "IS5"
UY081	IS_cc12ea8f.1	2805787	2807003	family "IS5"	group "IS5"
UY081	IS_cc12ea8f.1	3185974	3187190	family "IS5"	group "IS5"
UY081	IS_cc12ea8f.1	3252608	3253824	family "IS5"	group "IS5"
UY081	IS_cc12ea8f.1	3404958	3406174	family "IS5"	group "IS5"
UY081	IS_cc12ea8f.1	64635	65849	family "IS5"	group "IS5"
UY081	IS_cc12ea8f.1	305840	307054	family "IS5"	group "IS5"
UY081	IS_cc12ea8f.1	2151230	2152444	family "IS5"	group "IS5"
UY081	IS_cc12ea8f.1	1203710	1204924	family "IS5"	group "IS5"
UY081	IS_cc12ea8f.1	2574226	2575439	family "IS5"	group "IS5"
UY081	IS_cc12ea8f.1	2809792	2811005	family "IS5"	group "IS5"
UY081	IS_cc12ea8f.1	949009	950221	family "IS5"	group "IS5"
UY081	IS_cc12ea8f.1	1252902	1254118	family "IS5"	group "IS5"
UY081	IS_cc12ea8f.1	2087335	2088547	family "IS5"	group "IS5"
UY081	IS_cc12ea8f.1	2089076	2090288	family "IS5"	group "IS5"
UY081	IS_cc12ea8f.1	2301602	2302814	family "IS5"	group "IS5"
UY081	IS_cc12ea8f.1	2343625	2344837	family "IS5"	group "IS5"
UY081	IS_cc12ea8f.1	2793773	2794985	family "IS5"	group "IS5"
UY081	IS_cc12ea8f.1	57487	58698	family "IS5"	group "IS5"
UY081	IS_cc12ea8f.1	2149020	2150231	family "IS5"	group "IS5"
UY081	IS_cc12ea8f.1	2732977	2734188	family "IS5"	group "IS5"
UY081	IS_cc12ea8f.1	428734	429942	family "IS5"	group "IS5"
UY081	IS_cc12ea8f.1	2300245	2301594	family "IS3"	group "IS2"
UY081	IS_cc12ea8f.1	3220486	3221825	family "IS3"	group "IS2"
UY081	IS_cc12ea8f.1	489042	490380	family "IS3"	group "IS2"
UY081	IS_cc12ea8f.1	1260562	1261899	family "IS3"	group "IS2"
UY081	IS_cc12ea8f.1	2012919	2014256	family "IS3"	group "IS2"
UY081	IS_cc12ea8f.1	2816320	2817657	family "IS3"	group "IS2"
UY081	IS_cc12ea8f.1	71767	72609	family "IS5"	group "IS1031"
UY081	IS_cc12ea8f.1	855198	856040	family "IS5"	group "IS1031"
UY081	IS_cc12ea8f.1	2344838	2345680	family "IS5"	group "IS1031"
UY081	IS_cc12ea8f.1	3253831	3254673	family "IS5"	group "IS1031"
UY081	IS_cc12ea8f.1	564753	565439	family "None"	group "None"
UW163	IS_dbbfd929	302600	303936	family "IS3"	group "IS2"
UW163	IS_dbbfd929	318991	320327	family "IS3"	group "IS2"
UW163	IS_dbbfd929	2487315	2488651	family "IS3"	group "IS2"
UW163	IS_dbbfd929	3368848	3370184	family "IS3"	group "IS2"
UW163	IS_dbbfd929	28769	30104	family "IS3"	group "IS2"
UW163	IS_dbbfd929	235059	236945	family "IS21"	group "-"
UW163	IS_dbbfd929	1479746	1481632	family "IS21"	group "-"
UW163	IS_dbbfd929	321695	323581	family "IS21"	group "-"
UW163	IS_dbbfd929	1261666	1263552	family "IS21"	group "-"
UW163	IS_dbbfd929	2054540	2056426	family "IS21"	group "-"
UW163	IS_dbbfd929	3371442	3373328	family "IS21"	group "-"
UW163	IS_dbbfd929	309860	311746	family "IS21"	group "-"
UW163	IS_dbbfd929	3297474	3299360	family "IS21"	group "-"
UW163	IS_dbbfd929	906407	908293	family "IS21"	group "-"

UW163	IS_dbbfd929	3283400	3285286	family "IS21"	group "-"
UW163	IS_dbbfd929	246886	248204	family "IS3"	group "IS51"
UW163	IS_dbbfd929	2239337	2240655	family "IS3"	group "IS51"
UW163	IS_dbbfd929	2485311	2486629	family "IS3"	group "IS51"
UW163	IS_dbbfd929	2493088	2494406	family "IS3"	group "IS51"
UW163	IS_dbbfd929	3285316	3286229	family "IS3"	group "IS51"
UW163	IS_dbbfd929	980236	981067	family "IS1595"	group "IS1016"
UW163	IS_dbbfd929	1421537	1422368	family "IS1595"	group "IS1016"
UW163	IS_dbbfd929	2511479	2512310	family "IS1595"	group "IS1016"
UW163	IS_dbbfd929	817989	818575	family "None"	group "None"
UW163	IS_dbbfd929	817348	817921	family "None"	group "None"
UW163	IS_dbbfd929	1434331	1435017	family "None"	group "None"
IBSBF1503	IS_4388f2c8	424976	426862	family "IS21"	group "-"
IBSBF1503	IS_4388f2c8	920457	922343	family "IS21"	group "-"
IBSBF1503	IS_4388f2c8	2729805	2731691	family "IS21"	group "-"
IBSBF1503	IS_4388f2c8	1707892	1709778	family "IS21"	group "-"
IBSBF1503	IS_4388f2c8	511027	512087	family "IS21"	group "-"
IBSBF1503	IS_4388f2c8	426892	427805	family "IS3"	group "IS51"
IBSBF1503	IS_4388f2c8	736622	737535	family "IS3"	group "IS51"
IBSBF1503	IS_4388f2c8	465685	467020	family "IS3"	group "IS2"
IBSBF1503	IS_4388f2c8	2671063	2672398	family "IS3"	group "IS2"
IBSBF1503	IS_4388f2c8	2939646	2940981	family "IS3"	group "IS2"
IBSBF1503	IS_4388f2c8	1549079	1549910	family "IS1595"	group "IS1016"
IBSBF1503	IS_4388f2c8	3105924	3106755	family "IS1595"	group "IS1016"
IBSBF1503	IS_4388f2c8	2633750	2634945	family "IS3"	group "IS407"
IBSBF1503	IS_4388f2c8	2938323	2939518	family "IS3"	group "IS407"
IBSBF1503	IS_4388f2c8	2734685	2735880	family "IS3"	group "IS407"
RS488	CP021652	50545	51761	family "IS5"	group "IS5"
RS488	CP021652	74877	76093	family "IS5"	group "IS5"
RS488	CP021652	269471	270687	family "IS5"	group "IS5"
RS488	CP021652	308621	309837	family "IS5"	group "IS5"
RS488	CP021652	320153	321369	family "IS5"	group "IS5"
RS488	CP021652	343697	344913	family "IS5"	group "IS5"
RS488	CP021652	1051456	1052672	family "IS5"	group "IS5"
RS488	CP021652	1162764	1163980	family "IS5"	group "IS5"
RS488	CP021652	1237634	1238850	family "IS5"	group "IS5"
RS488	CP021652	1257021	1258237	family "IS5"	group "IS5"
RS488	CP021652	1437939	1439155	family "IS5"	group "IS5"
RS488	CP021652	2388911	2390127	family "IS5"	group "IS5"
RS488	CP021652	2392678	2393894	family "IS5"	group "IS5"
RS488	CP021652	2427163	2428379	family "IS5"	group "IS5"
RS488	CP021652	2514708	2515924	family "IS5"	group "IS5"
RS488	CP021652	2580101	2581317	family "IS5"	group "IS5"
RS488	CP021652	2707761	2708977	family "IS5"	group "IS5"
RS488	CP021652	2805776	2806992	family "IS5"	group "IS5"
RS488	CP021652	3185973	3187189	family "IS5"	group "IS5"
RS488	CP021652	3252608	3253824	family "IS5"	group "IS5"
RS488	CP021652	3404958	3406174	family "IS5"	group "IS5"
RS488	CP021652	64635	65849	family "IS5"	group "IS5"
RS488	CP021652	305840	307054	family "IS5"	group "IS5"
RS488	CP021652	2151220	2152434	family "IS5"	group "IS5"
RS488	CP021652	1203702	1204916	family "IS5"	group "IS5"
RS488	CP021652	2574216	2575429	family "IS5"	group "IS5"
RS488	CP021652	2809781	2810994	family "IS5"	group "IS5"
RS488	CP021652	949001	950213	family "IS5"	group "IS5"
RS488	CP021652	1252894	1254110	family "IS5"	group "IS5"
RS488	CP021652	2087325	2088537	family "IS5"	group "IS5"
RS488	CP021652	2089066	2090278	family "IS5"	group "IS5"
RS488	CP021652	2301592	2302804	family "IS5"	group "IS5"
RS488	CP021652	2343615	2344827	family "IS5"	group "IS5"
RS488	CP021652	2793762	2794974	family "IS5"	group "IS5"
RS488	CP021652	57487	58698	family "IS5"	group "IS5"

RS488	CP021652	2149010	2150221	family "IS5"	group "IS5"
RS488	CP021652	2732966	2734177	family "IS5"	group "IS5"
RS488	CP021652	428731	429939	family "IS5"	group "IS5"
RS488	CP021652	855180	856042	family "IS5"	group "IS1031"
RS488	CP021652	3253821	3254683	family "IS5"	group "IS1031"
RS488	CP021652	71758	72618	family "IS5"	group "IS1031"
RS488	CP021652	2344828	2345670	family "IS5"	group "IS1031"
RS488	CP021652	489036	490374	family "IS3"	group "IS2"
RS488	CP021652	1260553	1261891	family "IS3"	group "IS2"
RS488	CP021652	2012908	2014246	family "IS3"	group "IS2"
RS488	CP021652	2816308	2817646	family "IS3"	group "IS2"
RS488	CP021652	3220488	3221826	family "IS3"	group "IS2"
RS488	CP021652	2300237	2301574	family "IS3"	group "IS2"
RS488	CP021652	2296709	2297997	family "IS3"	group "IS3"
RS488	CP021652	2305884	2307170	family "IS3"	group "IS3"
RS488	CP021652	564748	565434	family "None"	group "None"
RS488	CP021652	3389829	3391056	family "None"	group "None"

CFBP2957 No IS(s) found using OASIS Predictor...

UW551	NCTI01000001.1	3173	4033	family "IS5"	group "IS1031"
UW551	NCTI01000001.1	20567	21427	family "IS5"	group "IS1031"
UW551	NCTI01000001.1	3433409	3434269	family "IS5"	group "IS1031"
UW551	NCTI01000001.1	3450802	3451662	family "IS5"	group "IS1031"
UW551	NCTI01000001.1	3468194	3469054	family "IS5"	group "IS1031"
UW551	NCTI01000001.1	250637	251497	family "IS5"	group "IS1031"
UW551	NCTI01000001.1	2649959	2650819	family "IS5"	group "IS1031"
UW551	NCTI01000001.1	1163352	1164194	family "IS5"	group "IS1031"
UW551	NCTI01000001.1	41423	42639	family "IS5"	group "IS5"
UW551	NCTI01000001.1	99146	100362	family "IS5"	group "IS5"
UW551	NCTI01000001.1	251495	252711	family "IS5"	group "IS5"
UW551	NCTI01000001.1	253992	255208	family "IS5"	group "IS5"
UW551	NCTI01000001.1	320633	321849	family "IS5"	group "IS5"
UW551	NCTI01000001.1	371708	372924	family "IS5"	group "IS5"
UW551	NCTI01000001.1	702037	703253	family "IS5"	group "IS5"
UW551	NCTI01000001.1	773108	774324	family "IS5"	group "IS5"
UW551	NCTI01000001.1	801264	802480	family "IS5"	group "IS5"
UW551	NCTI01000001.1	993104	994320	family "IS5"	group "IS5"
UW551	NCTI01000001.1	1080645	1081861	family "IS5"	group "IS5"
UW551	NCTI01000001.1	1115129	1116345	family "IS5"	group "IS5"
UW551	NCTI01000001.1	1118896	1120112	family "IS5"	group "IS5"
UW551	NCTI01000001.1	2068067	2069283	family "IS5"	group "IS5"
UW551	NCTI01000001.1	2267155	2268371	family "IS5"	group "IS5"
UW551	NCTI01000001.1	2342025	2343241	family "IS5"	group "IS5"
UW551	NCTI01000001.1	2453332	2454548	family "IS5"	group "IS5"
UW551	NCTI01000001.1	3161087	3162303	family "IS5"	group "IS5"
UW551	NCTI01000001.1	3200240	3201456	family "IS5"	group "IS5"
UW551	NCTI01000001.1	3211771	3212987	family "IS5"	group "IS5"
UW551	NCTI01000001.1	3235340	3236556	family "IS5"	group "IS5"
UW551	NCTI01000001.1	3429934	3431150	family "IS5"	group "IS5"
UW551	NCTI01000001.1	9942	11156	family "IS5"	group "IS5"
UW551	NCTI01000001.1	17093	18307	family "IS5"	group "IS5"
UW551	NCTI01000001.1	27335	28549	family "IS5"	group "IS5"
UW551	NCTI01000001.1	3197459	3198673	family "IS5"	group "IS5"
UW551	NCTI01000001.1	3440177	3441391	family "IS5"	group "IS5"
UW551	NCTI01000001.1	3447328	3448542	family "IS5"	group "IS5"
UW551	NCTI01000001.1	3457570	3458784	family "IS5"	group "IS5"
UW551	NCTI01000001.1	3464721	3465935	family "IS5"	group "IS5"
UW551	NCTI01000001.1	1357034	1358248	family "IS5"	group "IS5"
UW551	NCTI01000001.1	2301089	2302303	family "IS5"	group "IS5"
UW551	NCTI01000001.1	698035	699248	family "IS5"	group "IS5"
UW551	NCTI01000001.1	933599	934812	family "IS5"	group "IS5"
UW551	NCTI01000001.1	714055	715267	family "IS5"	group "IS5"
UW551	NCTI01000001.1	1164195	1165407	family "IS5"	group "IS5"

UW551	NCTI01000001.1	1416978	1418190	family "IS5"	group "IS5"
UW551	NCTI01000001.1	1418719	1419931	family "IS5"	group "IS5"
UW551	NCTI01000001.1	2251895	2253111	family "IS5"	group "IS5"
UW551	NCTI01000001.1	2555788	2557000	family "IS5"	group "IS5"
UW551	NCTI01000001.1	34486	35697	family "IS5"	group "IS5"
UW551	NCTI01000001.1	776064	777275	family "IS5"	group "IS5"
UW551	NCTI01000001.1	1206734	1207944	family "IS5"	group "IS5"
UW551	NCTI01000001.1	3076062	3077270	family "IS5"	group "IS5"
UW551	NCTI01000001.1	1	915	family "IS5"	group "IS5"
UW551	NCTI01000001.1	248210	249075	family "IS5"	group "IS427"
UW551	NCTI01000001.1	1692064	1692934	family "IS5"	group "IS427"
UW551	NCTI01000001.1	285989	287327	family "IS3"	group "IS2"
UW551	NCTI01000001.1	1493009	1494347	family "IS3"	group "IS2"
UW551	NCTI01000001.1	2245327	2246665	family "IS3"	group "IS2"
UW551	NCTI01000001.1	3015625	3016963	family "IS3"	group "IS2"
UW551	NCTI01000001.1	1205379	1206716	family "IS3"	group "IS2"
UW551	NCTI01000001.1	691384	692722	family "IS3"	group "IS2"
UW551	NCTI01000001.1	1201851	1203139	family "IS3"	group "IS3"
UW551	NCTI01000001.1	1211473	1212759	family "IS3"	group "IS3"
RS489	No IS(s) found using OASIS Predictor...				
OE1-1	IS_fe1a9e3d	38488	39918	family "IS4"	group ""
OE1-1	IS_fe1a9e3d	3819057	3820487	family "IS4"	group ""
OE1-1	IS_fe1a9e3d	30417	31847	family "IS4"	group ""
OE1-1	IS_fe1a9e3d	355303	356733	family "IS4"	group ""
OE1-1	IS_fe1a9e3d	1210256	1211686	family "IS4"	group ""
OE1-1	IS_fe1a9e3d	1884567	1885997	family "IS4"	group ""
OE1-1	IS_fe1a9e3d	3303426	3304856	family "IS4"	group ""
OE1-1	IS_fe1a9e3d	2436151	2437581	family "IS4"	group ""
OE1-1	IS_fe1a9e3d	3502397	3503827	family "IS4"	group ""
OE1-1	IS_fe1a9e3d	847116	848546	family "IS4"	group ""
OE1-1	IS_fe1a9e3d	1153783	1155213	family "IS4"	group ""
OE1-1	IS_fe1a9e3d	253451	254336	family "IS5"	group "-"
OE1-1	IS_fe1a9e3d	2025658	2026543	family "IS5"	group "-"
OE1-1	IS_fe1a9e3d	3527804	3528689	family "IS5"	group "-"
OE1-1	IS_fe1a9e3d	2955845	2956730	family "IS5"	group "-"
OE1-1	IS_fe1a9e3d	946208	947073	family "IS5"	group "IS427"
OE1-1	IS_fe1a9e3d	1397294	1398159	family "IS5"	group "IS427"
OE1-1	IS_fe1a9e3d	2533489	2534354	family "IS5"	group "IS427"
OE1-1	IS_fe1a9e3d	2593066	2593931	family "IS5"	group "IS427"
OE1-1	IS_fe1a9e3d	3720762	3721627	family "IS5"	group "IS427"
OE1-1	IS_fe1a9e3d	285222	286087	family "IS5"	group "IS427"
OE1-1	IS_fe1a9e3d	615884	616749	family "IS5"	group "IS427"
OE1-1	IS_fe1a9e3d	954846	955711	family "IS5"	group "IS427"
OE1-1	IS_fe1a9e3d	3521445	3522310	family "IS5"	group "IS427"
OE1-1	IS_fe1a9e3d	696946	697810	family "IS5"	group "IS427"
OE1-1	IS_fe1a9e3d	923834	924816	family "IS3"	group "IS3"
OE1-1	IS_fe1a9e3d	2535149	2536127	family "IS3"	group "IS3"
OE1-1	IS_fe1a9e3d	1672253	1673434	family "IS5"	group "IS5"
OE1-1	IS_fe1a9e3d	2581174	2582355	family "IS5"	group "IS5"
OE1-1	IS_fe1a9e3d	3458870	3460051	family "IS5"	group "IS5"
OE1-1	IS_fe1a9e3d	3552300	3553481	family "IS5"	group "IS5"
OE1-1	IS_fe1a9e3d	3579823	3581004	family "IS5"	group "IS5"
OE1-1	IS_fe1a9e3d	2026682	2027858	family "IS5"	group "IS5"
OE1-1	IS_fe1a9e3d	2126572	2127748	family "IS5"	group "IS5"
OE1-1	IS_fe1a9e3d	2954271	2955452	family "IS5"	group "IS5"
OE1-1	IS_fe1a9e3d	149225	150581	family "IS110"	group "IS1111"
OE1-1	IS_fe1a9e3d	950001	951826	family "None"	group "None"
OE1-1	IS_fe1a9e3d	3610813	3612576	family "Tn3"	group ""
OE1-1	IS_fe1a9e3d	3535566	3537307	family "Tn3"	group ""
K60	NCTK01000001	65762	67235	family "IS4"	group "IS50"
K60	NCTK01000001	2624351	2625824	family "IS4"	group "IS50"

K60	NCTK01000001	1367717	1369190	family "IS4"	group "IS50"
K60	NCTK01000001	3266742	3268215	family "IS4"	group "IS50"
K60	NCTK01000001	1372623	1374096	family "IS4"	group "IS50"
K60	NCTK01000001	1555223	1556696	family "IS4"	group "IS50"
K60	NCTK01000001	3520280	3521753	family "IS4"	group "IS50"
K60	NCTK01000001	152417	153890	family "IS4"	group "IS50"
K60	NCTK01000001	233590	234707	family "IS630"	group ""
K60	NCTK01000001	862762	863881	family "IS630"	group ""
K60	NCTK01000001	811398	812991	family "IS1182"	group ""
K60	NCTK01000001	2646035	2647628	family "IS1182"	group ""
K60	NCTK01000001	2852777	2854368	family "IS1182"	group ""
K60	NCTK01000001	2417569	2419162	family "IS1182"	group ""
K60	NCTK01000001	1446299	1447892	family "IS1182"	group ""
K60	NCTK01000001	550039	551627	family "IS1182"	group ""
K60	NCTK01000001	222083	223193	family "IS1182"	group ""
K60	NCTK01000001	3732981	3734091	family "IS1182"	group ""
K60	NCTK01000001	1157163	1158273	family "IS1182"	group ""
K60	NCTK01000001	3421940	3423050	family "IS1182"	group ""
K60	NCTK01000001	3620657	3621767	family "IS1182"	group ""
K60	NCTK01000001	388307	389416	family "IS1182"	group ""
K60	NCTK01000001	3000832	3001941	family "IS1182"	group ""
K60	NCTK01000001	1949073	1950184	family "IS1182"	group ""
K60	NCTK01000001	858235	859335	family "IS630"	group ""
K60	NCTK01000001	1840253	1841353	family "IS630"	group ""
K60	NCTK01000001	859664	860982	family "IS3"	group "IS51"
K60	NCTK01000001	3598312	3599630	family "IS3"	group "IS51"
K60	NCTK01000001	887952	890420	family "IS21"	group "-"
K60	NCTK01000001	2149924	2152392	family "IS21"	group "-"
K60	NCTK01000001	3638968	3641436	family "IS21"	group "-"
K60	NCTK01000001	2477504	2479972	family "IS21"	group "-"
K60	NCTK01000001	2628525	2630993	family "IS21"	group "-"
K60	NCTK01000001	892967	893780	family "IS5"	group "IS427"
K60	NCTK01000001	2289673	2290486	family "IS5"	group "IS427"
K60	NCTK01000001	2937243	2938056	family "IS5"	group "IS427"
K60	NCTK01000001	3634459	3635272	family "IS5"	group "IS427"
K60	NCTK01000001	1374429	1375241	family "IS5"	group "IS427"
K60	NCTK01000001	1408948	1409761	family "IS5"	group "IS427"
K60	NCTK01000001	3366080	3366893	family "IS5"	group "IS427"
K60	NCTK01000001	1966243	1967056	family "IS5"	group "IS427"
K60	NCTK01000001	3269275	3270088	family "IS5"	group "IS427"
K60	NCTK01000001	3699133	3699946	family "IS5"	group "IS427"
K60	NCTK01000001	3313683	3314495	family "IS5"	group "IS427"
K60	NCTK01000001	2304968	2306405	family "IS4"	group "IS50"
K60	NCTK01000001	1365589	1367026	family "IS4"	group "IS50"
K60	NCTK01000001	3510336	3511773	family "IS4"	group "IS50"
K60	NCTK01000001	1092515	1093951	family "IS4"	group "IS50"
K60	NCTK01000001	1222500	1224011	family "IS630"	group ""
K60	NCTK01000001	1358024	1359535	family "IS630"	group ""
K60	NCTK01000001	1844245	1846131	family "IS21"	group "-"
K60	NCTK01000001	3635924	3637810	family "IS21"	group "-"
K60	NCTK01000001	2475655	2477504	family "IS21"	group "-"
K60	NCTK01000001	3126535	3127110	family "IS21"	group "-"
K60	NCTK01000001	3783073	3786385	family "None"	group "None"
K60	NCTK01000001	3823341	3826641	family "None"	group "None"
K60	NCTK01000001	873957	875409	family "IS110"	group "IS1111"
K60	NCTK01000001	1599912	1601385	family "IS4"	group "IS50"
K60	NCTK01000001	1356773	1357328	family "IS4"	group "IS50"
K60	NCTK01000001	1758479	1759065	family "None"	group "None"
K60	NCTK01000001	1757838	1758411	family "None"	group "None"
K60	NCTK01000001	1900035	1901770	family "None"	group "None"
K60	NCTK01000001	2431155	2431841	family "None"	group "None"
K60	NCTK01000001	2836264	2837292	family "IS110"	group "IS1111"
K60	NCTK01000001	3503356	3504080	family "IS110"	group "IS1111"
K60	NCTK01000001	3515418	3516275	family "IS3"	group "IS407"

K60	NCTK01000001	3600834	3603135	family "IS701"	group ""
K60	NCTK01000001	84932	85983	family "IS701"	group ""
K60	NCTK01000001	3272275	3273326	family "IS701"	group ""
K60	NCTK01000001	371992	373043	family "IS701"	group ""
K60	NCTK01000001	857231	858041	family "IS701"	group ""
K60	NCTK01000001	2194583	2195393	family "IS701"	group ""
K60	NCTK01000001	2634485	2635295	family "IS701"	group ""
K60	NCTK01000001	1338796	1339606	family "IS701"	group ""
K60	NCTK01000001	1190028	1190840	family "IS701"	group ""
FJAT-1458	IS_9b9ce816	94098	95546	family "IS3"	group "IS150"
FJAT-1458	IS_9b9ce816	2253158	2254606	family "IS3"	group "IS150"
FJAT-1458	IS_9b9ce816	206847	208078	family "IS3"	group "IS51"
FJAT-1458	IS_9b9ce816	3070260	3071491	family "IS3"	group "IS51"
FJAT-1458	IS_9b9ce816	3749432	3750663	family "IS3"	group "IS51"
FJAT-1458	IS_9b9ce816	1182297	1183528	family "IS3"	group "IS51"
FJAT-1458	IS_9b9ce816	1218034	1219138	family "IS3"	group "IS51"
FJAT-1458	IS_9b9ce816	222984	224164	family "IS5"	group "IS5"
FJAT-1458	IS_9b9ce816	3034789	3035969	family "IS5"	group "IS5"
FJAT-1458	IS_9b9ce816	1457525	1458705	family "IS5"	group "IS5"
FJAT-1458	IS_9b9ce816	3694514	3695694	family "IS5"	group "IS5"
FJAT-1458	IS_9b9ce816	307273	308449	family "IS5"	group "IS5"
FJAT-1458	IS_9b9ce816	1426081	1427257	family "IS5"	group "IS5"
FJAT-1458	IS_9b9ce816	1758674	1759850	family "IS5"	group "IS5"
FJAT-1458	IS_9b9ce816	2742553	2743729	family "IS5"	group "IS5"
FJAT-1458	IS_9b9ce816	1914506	1915681	family "IS5"	group "IS5"
FJAT-1458	IS_9b9ce816	271944	274510	family "None"	group "None"
FJAT-1458	IS_9b9ce816	3024215	3026723	family "None"	group "None"
FJAT-1458	IS_9b9ce816	1265938	1267151	family "IS5"	group "IS5"
FJAT-1458	IS_9b9ce816	1622818	1624031	family "IS5"	group "IS5"
FJAT-1458	IS_9b9ce816	1629071	1630284	family "IS5"	group "IS5"
FJAT-1458	IS_9b9ce816	1899874	1901087	family "IS5"	group "IS5"
FJAT-1458	IS_9b9ce816	1930049	1931262	family "IS5"	group "IS5"
FJAT-1458	IS_9b9ce816	2770199	2771412	family "IS5"	group "IS5"
FJAT-1458	IS_9b9ce816	3650562	3651775	family "IS5"	group "IS5"
FJAT-1458	IS_9b9ce816	1432125	1433332	family "IS5"	group "IS5"
FJAT-1458	IS_9b9ce816	1963404	1964611	family "IS5"	group "IS5"
FJAT-1458	IS_9b9ce816	2131208	2132415	family "IS5"	group "IS5"
FJAT-1458	IS_9b9ce816	2747953	2749160	family "IS5"	group "IS5"
FJAT-1458	IS_9b9ce816	3695696	3696903	family "IS5"	group "IS5"
FJAT-1458	IS_9b9ce816	274676	275568	family "IS5"	group "-"
FJAT-1458	IS_9b9ce816	277395	278280	family "IS5"	group "-"
FJAT-1458	IS_9b9ce816	1997281	1998166	family "IS5"	group "-"
FJAT-1458	IS_9b9ce816	3050733	3051618	family "IS5"	group "-"
FJAT-1458	IS_9b9ce816	587633	588480	family "None"	group "None"
FJAT-1458	IS_9b9ce816	1067797	1068626	family "IS1595"	group "IS1016"
FJAT-1458	IS_9b9ce816	1979455	1980885	family "IS4"	group ""
FJAT-1458	IS_9b9ce816	382784	384214	family "IS4"	group ""
FJAT-1458	IS_9b9ce816	574214	575644	family "IS4"	group ""
FJAT-1458	IS_9b9ce816	613958	615388	family "IS4"	group ""
FJAT-1458	IS_9b9ce816	1127602	1129032	family "IS4"	group ""
FJAT-1458	IS_9b9ce816	3939750	3941180	family "IS4"	group ""
FJAT-1458	IS_9b9ce816	301378	302808	family "IS4"	group ""
FJAT-1458	IS_9b9ce816	2820489	2821919	family "IS4"	group ""
FJAT-1458	IS_9b9ce816	3173394	3174824	family "IS4"	group ""
FJAT-1458	IS_9b9ce816	2715219	2716649	family "IS4"	group ""
FJAT-1458	IS_9b9ce816	897309	898739	family "IS4"	group ""
FJAT-1458	IS_9b9ce816	1327976	1329406	family "IS4"	group ""
FJAT-1458	IS_9b9ce816	3032104	3033577	family "IS4"	group "IS50"
EP1	IS_4f0ea434	1293565	1294547	family "IS3"	group "IS3"
EP1	IS_4f0ea434	3027232	3028210	family "IS3"	group "IS3"
EP1	IS_4f0ea434	2924043	2926429	family "Tn3"	group ""
EP1	IS_4f0ea434	2997475	2999861	family "Tn3"	group ""

EP1	IS_4f0ea434	2844223	2846573	family "Tn3"	group ""
EP1	IS_4f0ea434	212859	213361	family "Tn3"	group ""
EP1	IS_4f0ea434	531898	532746	family "None"	group "None"
EP1	IS_4f0ea434	987562	989010	family "IS3"	group "IS150"
EP1	IS_4f0ea434	1056091	1056920	family "IS1595"	group "IS1016"
EP1	IS_4f0ea434	1261762	1262770	family "Tn3"	group ""
EP1	IS_4f0ea434	3239649	3240856	family "IS5"	group "IS5"
EP1	IS_4f0ea434	3254260	3255124	family "IS5"	group "IS427"
EP1	IS_4f0ea434	301029	301894	family "IS5"	group "IS427"
EP1	IS_4f0ea434	2922903	2923768	family "IS5"	group "IS427"
EP1	IS_4f0ea434	3663642	3664507	family "IS5"	group "IS427"
EP1	IS_4f0ea434	1275332	1276197	family "IS5"	group "IS427"
EP1	IS_4f0ea434	102890	103755	family "IS5"	group "IS427"
EP1	IS_4f0ea434	2469708	2470573	family "IS5"	group "IS427"
EP1	IS_4f0ea434	3004973	3005838	family "IS5"	group "IS427"
CQPS- 1	CP016914	75267	76697	family "IS4"	group ""
CQPS- 1	CP016914	181941	183371	family "IS4"	group ""
CQPS- 1	CP016914	1735746	1737176	family "IS4"	group ""
CQPS- 1	CP016914	2255822	2257252	family "IS4"	group ""
CQPS- 1	CP016914	2780565	2781995	family "IS4"	group ""
CQPS- 1	CP016914	3329935	3331365	family "IS4"	group ""
CQPS- 1	CP016914	238410	239840	family "IS4"	group ""
CQPS- 1	CP016914	461852	463282	family "IS4"	group ""
CQPS- 1	CP016914	1454067	1455497	family "IS4"	group ""
CQPS- 1	CP016914	1446441	1447429	family "IS4"	group ""
CQPS- 1	CP016914	389167	390052	family "IS5"	group "-"
CQPS- 1	CP016914	3192320	3193205	family "IS5"	group "-"
CQPS- 1	CP016914	1709710	1710595	family "IS5"	group "-"
CQPS- 1	CP016914	605412	606588	family "IS5"	group "IS5"
CQPS- 1	CP016914	1033101	1034277	family "IS5"	group "IS5"
CQPS- 1	CP016914	1268260	1269436	family "IS5"	group "IS5"
CQPS- 1	CP016914	1691834	1693010	family "IS5"	group "IS5"
CQPS- 1	CP016914	3142781	3143957	family "IS5"	group "IS5"
CQPS- 1	CP016914	429523	430696	family "IS5"	group "IS5"
CQPS- 1	CP016914	1717974	1719147	family "IS5"	group "IS5"
CQPS- 1	CP016914	2275684	2276857	family "IS5"	group "IS5"
CQPS- 1	CP016914	3822102	3823278	family "IS5"	group "IS5"
CQPS- 1	CP016914	479128	480301	family "IS5"	group "IS5"
CQPS- 1	CP016914	432294	435204	family "Tn3"	group ""
CQPS- 1	CP016914	1700527	1703107	family "Tn3"	group ""
CQPS- 1	CP016914	493917	494422	family "Tn3"	group ""
CQPS- 1	CP016914	483912	485361	family "IS91"	group ""
CQPS- 1	CP016914	526220	527669	family "IS91"	group ""
CQPS- 1	CP016914	485359	487355	family "None"	group "None"
CQPS- 1	CP016914	524225	526221	family "None"	group "None"
CQPS- 1	CP016914	488999	490475	family "IS701"	group "-"
CQPS- 1	CP016914	1261845	1263315	family "IS701"	group "-"
CQPS- 1	CP016914	490477	491684	family "IS5"	group "IS5"
CQPS- 1	CP016914	752793	754000	family "IS5"	group "IS5"
CQPS- 1	CP016914	555403	557425	family "IS21"	group ""
CQPS- 1	CP016914	989897	991919	family "IS21"	group ""
CQPS- 1	CP016914	980636	982475	family "IS66"	group ""
CQPS- 1	CP016914	1191469	1193308	family "IS66"	group ""
CQPS- 1	CP016914	982476	983709	family "IS3"	group "IS407"
CQPS- 1	CP016914	1266536	1267769	family "IS3"	group "IS407"
CQPS- 1	CP016914	1445207	1446440	family "IS3"	group "IS407"
CQPS- 1	CP016914	983708	984453	family "None"	group "None"
CQPS- 1	CP016914	1193305	1194048	family "None"	group "None"
CQPS- 1	CP016914	2390432	2391880	family "IS3"	group "IS150"
CQPS- 1	CP016914	2502314	2503762	family "IS3"	group "IS150"
CQPS- 1	CP016914	361316	362171	family "IS5"	group "IS427"
CQPS- 1	CP016914	1586827	1587690	family "IS5"	group "IS427"
CQPS- 1	CP016914	1716088	1716952	family "IS5"	group "IS427"

CQPS- 1	CP016914	3104629	3105493	family "IS5"	group "IS427"
CQPS- 1	CP016914	856754	857619	family "IS5"	group "IS427"
CQPS- 1	CP016914	1214614	1215479	family "IS5"	group "IS427"
CQPS- 1	CP016914	2646773	2647638	family "IS5"	group "IS427"
CQPS- 1	CP016914	2683772	2684637	family "IS5"	group "IS427"
CQPS- 1	CP016914	1980367	1981215	family "None"	group "None"
CQPS- 1	CP016914	2458953	2459782	family "IS1595"	group "IS1016"
FJAT-91	IS_45c6ab84	1293565	1294547	family "IS3"	group "IS3"
FJAT-91	IS_45c6ab84	3027232	3028210	family "IS3"	group "IS3"
FJAT-91	IS_45c6ab84	2924043	2926429	family "Tn3"	group ""
FJAT-91	IS_45c6ab84	2997475	2999861	family "Tn3"	group ""
FJAT-91	IS_45c6ab84	2844223	2846573	family "Tn3"	group ""
FJAT-91	IS_45c6ab84	212859	213361	family "Tn3"	group ""
FJAT-91	IS_45c6ab84	531898	532746	family "None"	group "None"
FJAT-91	IS_45c6ab84	987562	989010	family "IS3"	group "IS150"
FJAT-91	IS_45c6ab84	1056091	1056920	family "IS1595"	group "IS1016"
FJAT-91	IS_45c6ab84	1261762	1262770	family "Tn3"	group ""
FJAT-91	IS_45c6ab84	3239649	3240856	family "IS5"	group "IS5"
FJAT-91	IS_45c6ab84	3254260	3255124	family "IS5"	group "IS427"
FJAT-91	IS_45c6ab84	301029	301894	family "IS5"	group "IS427"
FJAT-91	IS_45c6ab84	2922903	2923768	family "IS5"	group "IS427"
FJAT-91	IS_45c6ab84	3663642	3664507	family "IS5"	group "IS427"
FJAT-91	IS_45c6ab84	1275332	1276197	family "IS5"	group "IS427"
FJAT-91	IS_45c6ab84	102890	103755	family "IS5"	group "IS427"
FJAT-91	IS_45c6ab84	2469708	2470573	family "IS5"	group "IS427"
FJAT-91	IS_45c6ab84	3004973	3005838	family "IS5"	group "IS427"
FQY_4	IS_9e96eacd	208223	210506	family "IS110"	group "IS1111"
FQY_4	IS_9e96eacd	3261947	3264230	family "IS110"	group "IS1111"
FQY_4	IS_9e96eacd	125839	128121	family "IS110"	group "IS1111"
FQY_4	IS_9e96eacd	3691899	3692413	family "IS110"	group "IS1111"
FQY_4	IS_9e96eacd	845784	846963	family "IS5"	group "IS5"
FQY_4	IS_9e96eacd	869600	870779	family "IS5"	group "IS5"
FQY_4	IS_9e96eacd	277023	278199	family "IS5"	group "IS5"
FQY_4	IS_9e96eacd	2031256	2032432	family "IS5"	group "IS5"
FQY_4	IS_9e96eacd	2253821	2254997	family "IS5"	group "IS5"
FQY_4	IS_9e96eacd	3085605	3086781	family "IS5"	group "IS5"
FQY_4	IS_9e96eacd	240918	242094	family "IS5"	group "IS5"
FQY_4	IS_9e96eacd	3291864	3293043	family "IS5"	group "IS5"
FQY_4	IS_9e96eacd	285591	286679	family "IS30"	group "-"
FQY_4	IS_9e96eacd	847880	848964	family "IS30"	group "-"
FQY_4	IS_9e96eacd	302894	304032	family "IS5"	group "IS5"
FQY_4	IS_9e96eacd	3070588	3071724	family "IS5"	group "IS5"
FQY_4	IS_9e96eacd	2788309	2789447	family "IS5"	group "IS5"
FQY_4	IS_9e96eacd	2804238	2805376	family "IS5"	group "IS5"
FQY_4	IS_9e96eacd	2647257	2648053	family "IS5"	group "IS5"
FQY_4	IS_9e96eacd	662044	663501	family "IS110"	group ""
FQY_4	IS_9e96eacd	3080039	3081496	family "IS110"	group ""
FQY_4	IS_9e96eacd	1552090	1553540	family "IS3"	group "IS150"
FQY_4	IS_9e96eacd	736054	737502	family "IS3"	group "IS150"
FQY_4	IS_9e96eacd	3348267	3349715	family "IS3"	group "IS150"
FQY_4	IS_9e96eacd	1149185	1150646	family "IS4"	group "IS50"
FQY_4	IS_9e96eacd	2523574	2525029	family "IS4"	group "IS50"
FQY_4	IS_9e96eacd	1952890	1954345	family "IS4"	group "IS50"
FQY_4	IS_9e96eacd	3408805	3410260	family "IS4"	group "IS50"
FQY_4	IS_9e96eacd	1960186	1962739	family "IS66"	group ""
FQY_4	IS_9e96eacd	2140760	2143311	family "IS66"	group ""
FQY_4	IS_9e96eacd	2777247	2779803	family "IS66"	group ""
FQY_4	IS_9e96eacd	3687179	3689735	family "IS66"	group ""
FQY_4	IS_9e96eacd	3055816	3058374	family "IS66"	group ""
FQY_4	IS_9e96eacd	3461546	3464045	family "IS66"	group ""
FQY_4	IS_9e96eacd	1152358	1154855	family "IS66"	group ""
FQY_4	IS_9e96eacd	1154854	1157351	family "IS66"	group ""

FQY_4	IS_9e96eacd	149396	150752	family "IS110"	group "IS1111"
FQY_4	IS_9e96eacd	875706	876590	family "IS5"	group "-"
FQY_4	IS_9e96eacd	867244	868129	family "IS5"	group "-"
FQY_4	IS_9e96eacd	1435470	1436980	family "IS630"	group ""
FQY_4	IS_9e96eacd	1617336	1618898	family "ISNCY"	group "IS1202"
FQY_4	IS_9e96eacd	1943457	1945138	family "None"	group "None"
FQY_4	IS_9e96eacd	2356555	2358026	family "IS4"	group ""
FQY_4	IS_9e96eacd	1810123	1811553	family "IS4"	group ""
FQY_4	IS_9e96eacd	3155190	3156620	family "IS4"	group ""
FQY_4	IS_9e96eacd	765837	767267	family "IS4"	group ""
FQY_4	IS_9e96eacd	1021385	1022815	family "IS4"	group ""
FQY_4	IS_9e96eacd	1067171	1068601	family "IS4"	group ""
FQY_4	IS_9e96eacd	328603	330037	family "IS4"	group ""
FQY_4	IS_9e96eacd	922984	924416	family "IS4"	group ""
FQY_4	IS_9e96eacd	3390196	3391635	family "IS4"	group ""
FQY_4	IS_9e96eacd	2822161	2823597	family "IS4"	group ""
FQY_4	IS_9e96eacd	991933	993363	family "IS4"	group ""
FQY_4	IS_9e96eacd	1000383	1001813	family "IS4"	group ""
FQY_4	IS_9e96eacd	1023844	1025278	family "IS4"	group ""
FQY_4	IS_9e96eacd	955509	956939	family "IS4"	group ""
FQY_4	IS_9e96eacd	2501870	2503101	family "IS3"	group "IS51"
FQY_4	IS_9e96eacd	2466320	2467426	family "IS3"	group "IS51"
RSCM	CP025985	1805256	1806485	family "IS3"	group "IS51"
RSCM	CP025985	2126401	2127630	family "IS3"	group "IS51"
RSCM	CP025985	2725697	2726926	family "IS3"	group "IS51"
RSCM	CP025985	3376174	3377403	family "IS3"	group "IS51"
RSCM	CP025985	11651	12867	family "IS3"	group "IS51"
RSCM	CP025985	944628	945522	family "IS3"	group "IS51"
RSCM	CP025985	2071814	2072540	family "IS3"	group "IS51"
RSCM	CP025985	36216	37395	family "IS5"	group "IS5"
RSCM	CP025985	352806	353985	family "IS5"	group "IS5"
RSCM	CP025985	630513	631692	family "IS5"	group "IS5"
RSCM	CP025985	1089448	1090627	family "IS5"	group "IS5"
RSCM	CP025985	1210526	1211705	family "IS5"	group "IS5"
RSCM	CP025985	1503373	1504552	family "IS5"	group "IS5"
RSCM	CP025985	1674168	1675347	family "IS5"	group "IS5"
RSCM	CP025985	3158515	3159694	family "IS5"	group "IS5"
RSCM	CP025985	1755879	1757056	family "IS5"	group "IS5"
RSCM	CP025985	1208231	1209407	family "IS5"	group "IS5"
RSCM	CP025985	1894182	1895358	family "IS5"	group "IS5"
RSCM	CP025985	2191869	2193045	family "IS5"	group "IS5"
RSCM	CP025985	2756160	2757336	family "IS5"	group "IS5"
RSCM	CP025985	1085977	1087152	family "IS5"	group "IS5"
RSCM	CP025985	990714	991887	family "IS5"	group "IS5"
RSCM	CP025985	1512948	1514121	family "IS5"	group "IS5"
RSCM	CP025985	2714122	2715298	family "IS5"	group "IS5"
RSCM	CP025985	2747970	2749144	family "IS5"	group "IS5"
RSCM	CP025985	3756736	3757909	family "IS5"	group "IS5"
RSCM	CP025985	204725	207274	family "IS66"	group ""
RSCM	CP025985	647266	649815	family "IS66"	group ""
RSCM	CP025985	938410	940959	family "IS66"	group ""
RSCM	CP025985	1368045	1370594	family "IS66"	group ""
RSCM	CP025985	1900593	1903142	family "IS66"	group ""
RSCM	CP025985	1910694	1913243	family "IS66"	group ""
RSCM	CP025985	2268020	2270569	family "IS66"	group ""
RSCM	CP025985	2585635	2588184	family "IS66"	group ""
RSCM	CP025985	2753025	2755574	family "IS66"	group ""
RSCM	CP025985	3245460	3248009	family "IS66"	group ""
RSCM	CP025985	3333835	3336384	family "IS66"	group ""
RSCM	CP025985	1304795	1307344	family "IS66"	group ""
RSCM	CP025985	2069264	2071813	family "IS66"	group ""
RSCM	CP025985	3079483	3080941	family "IS66"	group ""
RSCM	CP025985	3082095	3083189	family "IS66"	group ""

RSCM	CP025985	943309	944627	family "IS3"	group "IS51"
RSCM	CP025985	981598	982916	family "IS3"	group "IS51"
RSCM	CP025985	1081977	1083295	family "IS3"	group "IS51"
RSCM	CP025985	1240145	1241463	family "IS3"	group "IS51"
RSCM	CP025985	1315849	1317167	family "IS3"	group "IS51"
RSCM	CP025985	1474241	1475559	family "IS3"	group "IS51"
RSCM	CP025985	1806930	1808248	family "IS3"	group "IS51"
RSCM	CP025985	1913811	1915129	family "IS3"	group "IS51"
RSCM	CP025985	2594248	2595566	family "IS3"	group "IS51"
RSCM	CP025985	2729839	2731157	family "IS3"	group "IS51"
RSCM	CP025985	211702	213018	family "IS3"	group "IS51"
RSCM	CP025985	2513235	2514100	family "IS5"	group "IS427"
RSCM	CP025985	3207013	3207878	family "IS5"	group "IS427"
RSCM	CP025985	3727532	3728397	family "IS5"	group "IS427"
RSCM	CP025985	288787	289652	family "IS5"	group "IS427"
RSCM	CP025985	3661313	3662178	family "IS5"	group "IS427"
RSCM	CP025985	2747109	2747969	family "IS5"	group "IS427"
RSCM	CP025985	2715296	2716154	family "IS5"	group "IS427"
RSCM	CP025985	3316078	3316821	family "IS5"	group "IS427"
RSCM	CP025985	314507	315720	family "IS5"	group "IS5"
RSCM	CP025985	1354262	1355475	family "IS5"	group "IS5"
RSCM	CP025985	355141	356350	family "IS5"	group "IS5"
RSCM	CP025985	415401	416610	family "IS5"	group "IS5"
RSCM	CP025985	740841	742050	family "IS5"	group "IS5"
RSCM	CP025985	983332	984541	family "IS5"	group "IS5"
RSCM	CP025985	1216235	1217444	family "IS5"	group "IS5"
RSCM	CP025985	1321595	1322804	family "IS5"	group "IS5"
RSCM	CP025985	1444722	1445931	family "IS5"	group "IS5"
RSCM	CP025985	1675346	1676555	family "IS5"	group "IS5"
RSCM	CP025985	1679401	1680610	family "IS5"	group "IS5"
RSCM	CP025985	2867693	2868902	family "IS5"	group "IS5"
RSCM	CP025985	3352907	3354116	family "IS5"	group "IS5"
RSCM	CP025985	3398429	3399638	family "IS5"	group "IS5"
RSCM	CP025985	2502408	2503614	family "IS5"	group "IS5"
RSCM	CP025985	2957054	2958263	family "IS5"	group "IS5"
RSCM	CP025985	353983	355143	family "IS630"	group "-"
RSCM	CP025985	971450	972610	family "IS630"	group "-"
RSCM	CP025985	984542	985702	family "IS630"	group "-"
RSCM	CP025985	1163431	1164591	family "IS630"	group "-"
RSCM	CP025985	1215074	1216234	family "IS630"	group "-"
RSCM	CP025985	1320434	1321594	family "IS630"	group "-"
RSCM	CP025985	1443564	1444724	family "IS630"	group "-"
RSCM	CP025985	1673010	1674170	family "IS630"	group "-"
RSCM	CP025985	1809049	1810209	family "IS630"	group "-"
RSCM	CP025985	2056950	2058110	family "IS630"	group "-"
RSCM	CP025985	3080938	3082098	family "IS630"	group "-"
RSCM	CP025985	3336385	3337032	family "IS630"	group "-"
RSCM	CP025985	649815	650336	family "IS630"	group "-"
RSCM	CP025985	614484	616142	family "IS1182"	group ""
RSCM	CP025985	903737	905395	family "IS1182"	group ""
RSCM	CP025985	1539634	1541292	family "IS1182"	group ""
RSCM	CP025985	2197369	2199027	family "IS1182"	group ""
RSCM	CP025985	3067639	3069297	family "IS1182"	group ""
RSCM	CP025985	3314425	3316083	family "IS1182"	group ""
RSCM	CP025985	3343568	3345226	family "IS1182"	group ""
RSCM	CP025985	3646128	3647786	family "IS1182"	group ""
RSCM	CP025985	2528719	2530369	family "IS1182"	group ""
RSCM	CP025985	1804136	1805254	family "IS1182"	group ""
RSCM	CP025985	985748	987218	family "IS701"	group "-"
RSCM	CP025985	3317054	3318524	family "IS701"	group "-"
RSCM	CP025985	1897784	1899254	family "IS701"	group "-"
RSCM	CP025985	215107	216577	family "IS701"	group "-"
RSCM	CP025985	1758263	1759733	family "IS701"	group "-"
RSCM	CP025985	1810235	1811705	family "IS701"	group "-"

RSCM	CP025985	3160983	3162453	family "IS701"	group "-"
RSCM	CP025985	1353161	1354268	family "IS701"	group "-"
RSCM	CP025985	1088218	1089468	family "IS256"	group "-"
RSCM	CP025985	3157285	3158535	family "IS256"	group "-"
RSCM	CP025985	417569	418801	family "IS256"	group "-"
RSCM	CP025985	2229261	2230493	family "IS256"	group "-"
RSCM	CP025985	3133488	3134720	family "IS256"	group "-"
RSCM	CP025985	282817	284049	family "IS256"	group "-"
RSCM	CP025985	1099365	1100813	family "IS3"	group "IS150"
RSCM	CP025985	2970628	2972076	family "IS3"	group "IS150"
RSCM	CP025985	3385248	3386696	family "IS3"	group "IS150"
RSCM	CP025985	1915378	1916826	family "IS3"	group "IS150"
RSCM	CP025985	1207024	1208006	family "IS3"	group "IS3"
RSCM	CP025985	2712908	2713886	family "IS3"	group "IS3"
RSCM	CP025985	1313230	1315689	family "IS110"	group "IS1111"
RSCM	CP025985	2571750	2574209	family "IS110"	group "IS1111"
RSCM	CP025985	3370963	3373422	family "IS110"	group "IS1111"
RSCM	CP025985	3077624	3079482	family "IS110"	group "IS1111"
RSCM	CP025985	209829	211136	family "IS110"	group "IS1111"
RSCM	CP025985	3209841	3211148	family "IS110"	group "IS1111"
RSCM	CP025985	1903143	1904320	family "IS110"	group "IS1111"
RSCM	CP025985	3083190	3083798	family "IS110"	group "IS1111"
RSCM	CP025985	3747387	3748760	family "IS4"	group ""
RSCM	CP025985	3755362	3756735	family "IS4"	group ""
RSCM	CP025985	3073929	3075287	family "IS4"	group ""
RSCM	CP025985	778986	780340	family "IS4"	group ""
RSCM	CP025985	1602617	1603971	family "IS4"	group ""
RSCM	CP025985	739476	740840	family "IS4"	group ""
RSCM	CP025985	2614808	2616164	family "IS4"	group ""
RSCM	CP025985	3424076	3425432	family "IS4"	group ""
RSCM	CP025985	2524578	2525933	family "IS4"	group ""
RSCM	CP025985	3615482	3616837	family "IS4"	group ""
RSCM	CP025985	307352	308726	family "IS4"	group ""
RSCM	CP025985	553331	554685	family "IS4"	group ""
RSCM	CP025985	2445450	2446804	family "IS4"	group ""
RSCM	CP025985	3478313	3479667	family "IS4"	group ""
RSCM	CP025985	2213468	2214824	family "IS4"	group ""
RSCM	CP025985	2388885	2390240	family "IS4"	group ""
RSCM	CP025985	1129234	1130588	family "IS4"	group ""
RSCM	CP025985	271030	271914	family "IS5"	group "-"
RSCM	CP025985	3162457	3163342	family "IS5"	group "-"
RSCM	CP025985	976109	976994	family "IS5"	group "-"
RSCM	CP025985	1757374	1758259	family "IS5"	group "-"
RSCM	CP025985	3645117	3646002	family "IS5"	group "-"
RSCM	CP025985	1811709	1812585	family "IS5"	group "-"
RSCM	CP025985	2727566	2728451	family "IS5"	group "-"
RSCM	CP025985	566750	567598	family "None"	group "None"
RSCM	CP025985	1464681	1465545	family "IS5"	group "IS427"
RSCM	CP025985	5637	6502	family "IS5"	group "IS427"
RSCM	CP025985	12866	13731	family "IS5"	group "IS427"
RSCM	CP025985	932916	933781	family "IS5"	group "IS427"
RSCM	CP025985	1011358	1012223	family "IS5"	group "IS427"
RSCM	CP025985	2062752	2063617	family "IS5"	group "IS427"
RSCM	CP025985	2072539	2073404	family "IS5"	group "IS427"
RSCM	CP025985	2081248	2082113	family "IS5"	group "IS427"
RSCM	CP025985	2728453	2729318	family "IS5"	group "IS427"
RSCM	CP025985	3342667	3343532	family "IS5"	group "IS427"
RSCM	CP025985	642799	643650	family "IS5"	group "IS427"
RSCM	CP025985	3642838	3643689	family "IS5"	group "IS427"
RSCM	CP025985	2746251	2747071	family "IS5"	group "IS427"
RSCM	CP025985	1310424	1311251	family "IS5"	group "IS427"
T60	CP022768	5920	7096	family "IS5"	group "IS5"
T60	CP022768	455772	456948	family "IS5"	group "IS5"

T60	CP022768	1542129	1543305	family "IS5"	group "IS5"
T60	CP022768	2731265	2732441	family "IS5"	group "IS5"
T60	CP022768	2996376	2997552	family "IS5"	group "IS5"
T60	CP022768	3612244	3613420	family "IS5"	group "IS5"
T60	CP022768	965942	967115	family "IS5"	group "IS5"
T60	CP022768	3715831	3717004	family "IS5"	group "IS5"
T60	CP022768	380939	382112	family "IS5"	group "IS5"
T60	CP022768	321535	322708	family "IS5"	group "IS5"
T60	CP022768	279221	280928	family "None"	group "None"
T60	CP022768	2912994	2914701	family "None"	group "None"
T60	CP022768	668608	669815	family "IS5"	group "IS5"
T60	CP022768	2531583	2532790	family "IS5"	group "IS5"
T60	CP022768	2551392	2552599	family "IS5"	group "IS5"
T60	CP022768	3663447	3664654	family "IS5"	group "IS5"
T60	CP022768	357088	358295	family "IS5"	group "IS5"
T60	CP022768	2297247	2298454	family "IS5"	group "IS5"
T60	CP022768	375044	376474	family "IS4"	group ""
T60	CP022768	762837	764267	family "IS4"	group ""
T60	CP022768	1924050	1925480	family "IS4"	group ""
T60	CP022768	572127	573557	family "IS4"	group ""
T60	CP022768	1199956	1201386	family "IS4"	group ""
T60	CP022768	2650214	2651644	family "IS4"	group ""
T60	CP022768	2706672	2708102	family "IS4"	group ""
T60	CP022768	2801912	2803342	family "IS4"	group ""
T60	CP022768	3108602	3110032	family "IS4"	group ""
T60	CP022768	3905106	3906536	family "IS4"	group ""
T60	CP022768	1406128	1407558	family "IS4"	group ""
T60	CP022768	454417	455771	family "IS4"	group ""
T60	CP022768	1067688	1069136	family "IS3"	group "IS150"
T60	CP022768	2185505	2186953	family "IS3"	group "IS150"
T60	CP022768	1141205	1142436	family "IS3"	group "IS51"
T60	CP022768	2955207	2956438	family "IS3"	group "IS51"
T60	CP022768	1295836	1296940	family "IS3"	group "IS51"
T60	CP022768	1251265	1252033	family "IS3"	group "IS51"
T60	CP022768	1138325	1138980	family "IS3"	group "IS51"
T60	CP022768	2909930	2912908	family "Tn3"	group ""
T60	CP022768	2978217	2981153	family "Tn3"	group ""
T60	CP022768	2829997	2832840	family "Tn3"	group ""
T60	CP022768	212637	213139	family "Tn3"	group ""
T60	CP022768	585546	586393	family "None"	group "None"
T60	CP022768	1309886	1310771	family "IS5"	group "-"
T60	CP022768	2288435	2289320	family "IS5"	group "-"
T60	CP022768	3250279	3251164	family "IS5"	group "-"
T60	CP022768	3820228	3821113	family "IS5"	group "-"
T60	CP022768	2935680	2936565	family "IS5"	group "-"
SL3882	CP022778	278046	279753	family "None"	group "None"
SL3882	CP022778	2911820	2913527	family "None"	group "None"
SL3882	CP022778	454598	455774	family "IS5"	group "IS5"
SL3882	CP022778	1540964	1542140	family "IS5"	group "IS5"
SL3882	CP022778	2730086	2731262	family "IS5"	group "IS5"
SL3882	CP022778	2995205	2996381	family "IS5"	group "IS5"
SL3882	CP022778	3611089	3612265	family "IS5"	group "IS5"
SL3882	CP022778	964777	965950	family "IS5"	group "IS5"
SL3882	CP022778	3714676	3715849	family "IS5"	group "IS5"
SL3882	CP022778	379765	380938	family "IS5"	group "IS5"
SL3882	CP022778	320361	321534	family "IS5"	group "IS5"
SL3882	CP022778	667443	668650	family "IS5"	group "IS5"
SL3882	CP022778	2530405	2531612	family "IS5"	group "IS5"
SL3882	CP022778	3662292	3663499	family "IS5"	group "IS5"
SL3882	CP022778	355914	357121	family "IS5"	group "IS5"
SL3882	CP022778	2296069	2297276	family "IS5"	group "IS5"
SL3882	CP022778	2550213	2551420	family "IS5"	group "IS5"
SL3882	CP022778	373870	375300	family "IS4"	group ""

SL3882	CP022778	761672	763102	family "IS4"	group ""
SL3882	CP022778	1922881	1924311	family "IS4"	group ""
SL3882	CP022778	570953	572383	family "IS4"	group ""
SL3882	CP022778	1198793	1200223	family "IS4"	group ""
SL3882	CP022778	2649035	2650465	family "IS4"	group ""
SL3882	CP022778	2705493	2706923	family "IS4"	group ""
SL3882	CP022778	2800737	2802167	family "IS4"	group ""
SL3882	CP022778	3107431	3108861	family "IS4"	group ""
SL3882	CP022778	3903951	3905381	family "IS4"	group ""
SL3882	CP022778	1404964	1406394	family "IS4"	group ""
SL3882	CP022778	453243	454597	family "IS4"	group ""
SL3882	CP022778	1066524	1067972	family "IS3"	group "IS150"
SL3882	CP022778	2184327	2185775	family "IS3"	group "IS150"
SL3882	CP022778	1140042	1141273	family "IS3"	group "IS51"
SL3882	CP022778	2954036	2955267	family "IS3"	group "IS51"
SL3882	CP022778	1294673	1295777	family "IS3"	group "IS51"
SL3882	CP022778	1250102	1250870	family "IS3"	group "IS51"
SL3882	CP022778	1137161	1137816	family "IS3"	group "IS51"
SL3882	CP022778	2908756	2911734	family "Tn3"	group ""
SL3882	CP022778	2977046	2979982	family "Tn3"	group ""
SL3882	CP022778	2828823	2831666	family "Tn3"	group ""
SL3882	CP022778	211461	211963	family "Tn3"	group ""
SL3882	CP022778	584372	585219	family "None"	group "None"
SL3882	CP022778	1308723	1309608	family "IS5"	group "-"
SL3882	CP022778	2287257	2288142	family "IS5"	group "-"
SL3882	CP022778	3249108	3249993	family "IS5"	group "-"
SL3882	CP022778	3819073	3819958	family "IS5"	group "-"
SL3882	CP022778	2934506	2935391	family "IS5"	group "-"
T42	CP022772	14886	16016	family "IS5"	group "IS903"
T42	CP022772	696994	698124	family "IS5"	group "IS903"
T42	CP022772	838561	839691	family "IS5"	group "IS903"
T42	CP022772	3001980	3003110	family "IS5"	group "IS903"
T42	CP022772	3004543	3005673	family "IS5"	group "IS903"
T42	CP022772	3044377	3045507	family "IS5"	group "IS903"
T42	CP022772	3393135	3394265	family "IS5"	group "IS903"
T42	CP022772	1483403	1484582	family "IS5"	group "IS5"
T42	CP022772	2647251	2648430	family "IS5"	group "IS5"
T42	CP022772	2782688	2783867	family "IS5"	group "IS5"
T42	CP022772	3321143	3322322	family "IS5"	group "IS5"
T42	CP022772	3381874	3383053	family "IS5"	group "IS5"
T42	CP022772	305266	306442	family "IS5"	group "IS5"
T42	CP022772	1649588	1650764	family "IS5"	group "IS5"
T42	CP022772	2764801	2765977	family "IS5"	group "IS5"
T42	CP022772	3441846	3443022	family "IS5"	group "IS5"
T42	CP022772	245592	246765	family "IS5"	group "IS5"
T42	CP022772	281638	283111	family "IS4"	group "IS50"
T42	CP022772	698922	700395	family "IS4"	group "IS50"
T42	CP022772	992364	993837	family "IS4"	group "IS50"
T42	CP022772	299371	300801	family "IS4"	group ""
T42	CP022772	621715	623145	family "IS4"	group ""
T42	CP022772	1884345	1885775	family "IS4"	group ""
T42	CP022772	380802	382232	family "IS4"	group ""
T42	CP022772	497328	498758	family "IS4"	group ""
T42	CP022772	1100890	1102320	family "IS4"	group ""
T42	CP022772	1356235	1357665	family "IS4"	group ""
T42	CP022772	2566203	2567633	family "IS4"	group ""
T42	CP022772	2651832	2653262	family "IS4"	group ""
T42	CP022772	2719348	2720778	family "IS4"	group ""
T42	CP022772	2897398	2898828	family "IS4"	group ""
T42	CP022772	3677644	3679074	family "IS4"	group ""
T42	CP022772	2622661	2624091	family "IS4"	group ""
T42	CP022772	706835	708066	family "IS3"	group "IS51"
T42	CP022772	1037866	1039097	family "IS3"	group "IS51"

T42	CP022772	1155585	1156816	family "IS3"	group "IS51"
T42	CP022772	3315631	3316862	family "IS3"	group "IS51"
T42	CP022772	1246830	1247937	family "IS3"	group "IS51"
T42	CP022772	848053	849268	family "IS5"	group "IS5"
T42	CP022772	3312336	3313551	family "IS5"	group "IS5"
T42	CP022772	3335545	3336760	family "IS5"	group "IS5"
T42	CP022772	3505706	3506921	family "IS5"	group "IS5"
T42	CP022772	724099	725306	family "IS5"	group "IS5"
T42	CP022772	951887	953335	family "IS3"	group "IS150"
T42	CP022772	2145779	2147227	family "IS3"	group "IS150"
T42	CP022772	1029076	1030755	family "IS1182"	group ""
T42	CP022772	2997975	2999654	family "IS1182"	group ""
T42	CP022772	1486181	1487066	family "IS5"	group "-"
T42	CP022772	2248736	2249621	family "IS5"	group "-"
T42	CP022772	510747	511594	family "None"	group "None"
T42	CP022772	1041086	1041915	family "IS1595"	group "IS1016"
SL3300	CP022786	27488	28668	family "IS5"	group "IS5"
SL3300	CP022786	1476282	1477462	family "IS5"	group "IS5"
SL3300	CP022786	2306965	2308145	family "IS5"	group "IS5"
SL3300	CP022786	3531706	3532886	family "IS5"	group "IS5"
SL3300	CP022786	40841	42017	family "IS5"	group "IS5"
SL3300	CP022786	449104	450280	family "IS5"	group "IS5"
SL3300	CP022786	758923	760099	family "IS5"	group "IS5"
SL3300	CP022786	1357596	1358772	family "IS5"	group "IS5"
SL3300	CP022786	1656380	1657556	family "IS5"	group "IS5"
SL3300	CP022786	2748260	2749440	family "IS5"	group "IS5"
SL3300	CP022786	2817858	2819034	family "IS5"	group "IS5"
SL3300	CP022786	3210051	3211227	family "IS5"	group "IS5"
SL3300	CP022786	1339718	1340882	family "IS5"	group "IS5"
SL3300	CP022786	73754	74969	family "IS5"	group "IS5"
SL3300	CP022786	450278	451493	family "IS5"	group "IS5"
SL3300	CP022786	754679	755894	family "IS5"	group "IS5"
SL3300	CP022786	774465	775680	family "IS5"	group "IS5"
SL3300	CP022786	904533	905748	family "IS5"	group "IS5"
SL3300	CP022786	1081305	1082520	family "IS5"	group "IS5"
SL3300	CP022786	1642457	1643672	family "IS5"	group "IS5"
SL3300	CP022786	2455007	2456222	family "IS5"	group "IS5"
SL3300	CP022786	2465965	2467180	family "IS5"	group "IS5"
SL3300	CP022786	2997499	2998714	family "IS5"	group "IS5"
SL3300	CP022786	3042739	3043954	family "IS5"	group "IS5"
SL3300	CP022786	3547979	3549194	family "IS5"	group "IS5"
SL3300	CP022786	3648134	3649349	family "IS5"	group "IS5"
SL3300	CP022786	3743130	3744345	family "IS5"	group "IS5"
SL3300	CP022786	3768328	3769543	family "IS5"	group "IS5"
SL3300	CP022786	391706	392919	family "IS5"	group "IS5"
SL3300	CP022786	1302258	1303471	family "IS5"	group "IS5"
SL3300	CP022786	3470404	3471617	family "IS5"	group "IS5"
SL3300	CP022786	301527	302734	family "IS5"	group "IS5"
SL3300	CP022786	1300912	1302119	family "IS5"	group "IS5"
SL3300	CP022786	34406	35613	family "IS5"	group "IS5"
SL3300	CP022786	1304400	1305607	family "IS5"	group "IS5"
SL3300	CP022786	1677067	1678274	family "IS5"	group "IS5"
SL3300	CP022786	2774009	2775216	family "IS5"	group "IS5"
SL3300	CP022786	2452560	2453495	family "IS5"	group "IS5"
SL3300	CP022786	143097	143952	family "IS5"	group "IS427"
SL3300	CP022786	753511	754366	family "IS5"	group "IS427"
SL3300	CP022786	3630227	3631082	family "IS5"	group "IS427"
SL3300	CP022786	158145	159000	family "IS5"	group "IS427"
SL3300	CP022786	782200	783055	family "IS5"	group "IS427"
SL3300	CP022786	2990507	2991362	family "IS5"	group "IS427"
SL3300	CP022786	315916	317346	family "IS4"	group ""
SL3300	CP022786	3096308	3097738	family "IS4"	group ""
SL3300	CP022786	1220635	1222065	family "IS4"	group ""

SL3300	CP022786	1500622	1502052	family "IS4"	group ""
SL3300	CP022786	443209	444639	family "IS4"	group ""
SL3300	CP022786	553408	554838	family "IS4"	group ""
SL3300	CP022786	3829701	3831131	family "IS4"	group ""
SL3300	CP022786	1405382	1406812	family "IS4"	group ""
SL3300	CP022786	1557080	1558510	family "IS4"	group ""
SL3300	CP022786	2892294	2893724	family "IS4"	group ""
SL3300	CP022786	2281570	2283000	family "IS4"	group ""
SL3300	CP022786	677795	679225	family "IS4"	group ""
SL3300	CP022786	766513	767744	family "IS3"	group "IS51"
SL3300	CP022786	3040600	3041831	family "IS3"	group "IS51"
SL3300	CP022786	2453496	2454727	family "IS3"	group "IS51"
SL3300	CP022786	3468378	3469609	family "IS3"	group "IS51"
SL3300	CP022786	3004990	3006096	family "IS3"	group "IS51"
SL3300	CP022786	1008371	1009819	family "IS3"	group "IS150"
SL3300	CP022786	2021741	2023189	family "IS3"	group "IS150"
SL3300	CP022786	1048856	1050337	family "IS4"	group "IS50"
SL3300	CP022786	3837947	3839420	family "IS4"	group "IS50"
SL3300	CP022786	1086840	1088519	family "IS1182"	group ""
SL3300	CP022786	3166640	3168319	family "IS1182"	group ""
SL3300	CP022786	1919356	1920241	family "IS5"	group "-"
SL3300	CP022786	1338833	1339718	family "IS5"	group "-"
SL3300	CP022786	2989615	2990500	family "IS5"	group "-"
SL3300	CP022786	566827	567674	family "None"	group "None"
SL3822	CP022780	278722	280610	family "None"	group "None"
SL3822	CP022780	2839308	2841192	family "None"	group "None"
SL3822	CP022780	307478	308908	family "IS4"	group ""
SL3822	CP022780	1903938	1905368	family "IS4"	group ""
SL3822	CP022780	668561	669991	family "IS4"	group ""
SL3822	CP022780	386849	388279	family "IS4"	group ""
SL3822	CP022780	503375	504805	family "IS4"	group ""
SL3822	CP022780	866216	867646	family "IS4"	group ""
SL3822	CP022780	1114689	1116119	family "IS4"	group ""
SL3822	CP022780	1377890	1379320	family "IS4"	group ""
SL3822	CP022780	2584184	2585614	family "IS4"	group ""
SL3822	CP022780	2735902	2737332	family "IS4"	group ""
SL3822	CP022780	2998675	3000105	family "IS4"	group ""
SL3822	CP022780	3798080	3799510	family "IS4"	group ""
SL3822	CP022780	2640642	2642072	family "IS4"	group ""
SL3822	CP022780	1274688	1275867	family "IS5"	group "IS5"
SL3822	CP022780	313372	314547	family "IS5"	group "IS5"
SL3822	CP022780	1505059	1506235	family "IS5"	group "IS5"
SL3822	CP022780	2665232	2666408	family "IS5"	group "IS5"
SL3822	CP022780	2908889	2910065	family "IS5"	group "IS5"
SL3822	CP022780	3462417	3463593	family "IS5"	group "IS5"
SL3822	CP022780	854273	855448	family "IS5"	group "IS5"
SL3822	CP022780	967750	969198	family "IS3"	group "IS150"
SL3822	CP022780	2163754	2165202	family "IS3"	group "IS150"
SL3822	CP022780	2791053	2792266	family "IS5"	group "IS5"
SL3822	CP022780	2911521	2912734	family "IS5"	group "IS5"
SL3822	CP022780	3478687	3479900	family "IS5"	group "IS5"
SL3822	CP022780	3514625	3515832	family "IS5"	group "IS5"
SL3822	CP022780	981566	982773	family "IS5"	group "IS5"
SL3822	CP022780	1049309	1050540	family "IS3"	group "IS51"
SL3822	CP022780	1170612	1171843	family "IS3"	group "IS51"
SL3822	CP022780	2813264	2814495	family "IS3"	group "IS51"
SL3822	CP022780	1267305	1268411	family "IS3"	group "IS51"
SL3822	CP022780	2766110	2767583	family "IS4"	group "IS50"
SL3822	CP022780	2846535	2848008	family "IS4"	group "IS50"
SL3822	CP022780	1166084	1166979	family "IS4"	group "IS50"
SL3822	CP022780	2767584	2770299	family "Tn3"	group ""
SL3822	CP022780	2836446	2839143	family "Tn3"	group ""
SL3822	CP022780	210144	210649	family "Tn3"	group ""

SL3822	CP022780	2914013	2915143	family "IS5"	group "IS903"
SL3822	CP022780	2785280	2786410	family "IS5"	group "IS903"
SL3822	CP022780	2848029	2849159	family "IS5"	group "IS903"
SL3822	CP022780	280776	281668	family "IS5"	group "-"
SL3822	CP022780	283495	284380	family "IS5"	group "-"
SL3822	CP022780	2266720	2267605	family "IS5"	group "-"
SL3822	CP022780	2793737	2794622	family "IS5"	group "-"
SL3822	CP022780	516794	517641	family "None"	group "None"
SL3822	CP022780	1054885	1055714	family "IS1595"	group "IS1016"
HA4-1	CP022481	68154	69584	family "IS4"	group ""
HA4-1	CP022481	3005037	3006467	family "IS4"	group ""
HA4-1	CP022481	394294	395724	family "IS4"	group ""
HA4-1	CP022481	2215898	2217328	family "IS4"	group ""
HA4-1	CP022481	2623378	2624808	family "IS4"	group ""
HA4-1	CP022481	3412912	3414342	family "IS4"	group ""
HA4-1	CP022481	1095325	1096755	family "IS4"	group ""
HA4-1	CP022481	107898	109328	family "IS4"	group ""
HA4-1	CP022481	1357737	1359167	family "IS4"	group ""
HA4-1	CP022481	3031483	3032913	family "IS4"	group ""
HA4-1	CP022481	3085819	3087249	family "IS4"	group ""
HA4-1	CP022481	382889	384104	family "IS5"	group "IS5"
HA4-1	CP022481	1117904	1119119	family "IS5"	group "IS5"
HA4-1	CP022481	1442445	1443660	family "IS5"	group "IS5"
HA4-1	CP022481	2109062	2110277	family "IS5"	group "IS5"
HA4-1	CP022481	2459900	2461115	family "IS5"	group "IS5"
HA4-1	CP022481	2493541	2494756	family "IS5"	group "IS5"
HA4-1	CP022481	3101579	3102794	family "IS5"	group "IS5"
HA4-1	CP022481	313926	315135	family "IS5"	group "IS5"
HA4-1	CP022481	2820816	2822023	family "IS5"	group "IS5"
HA4-1	CP022481	3191917	3193124	family "IS5"	group "IS5"
HA4-1	CP022481	3208342	3209549	family "IS5"	group "IS5"
HA4-1	CP022481	2099059	2100266	family "IS5"	group "IS5"
HA4-1	CP022481	712385	714896	family "IS21"	group ""
HA4-1	CP022481	2105763	2108274	family "IS21"	group ""
HA4-1	CP022481	3202917	3205428	family "IS21"	group ""
HA4-1	CP022481	3471763	3474274	family "IS21"	group ""
HA4-1	CP022481	817371	818835	family "IS3"	group "IS150"
HA4-1	CP022481	1625068	1626520	family "IS3"	group "IS150"
HA4-1	CP022481	495865	497313	family "IS3"	group "IS150"
HA4-1	CP022481	628032	629480	family "IS3"	group "IS150"
HA4-1	CP022481	1148335	1149796	family "IS110"	group ""
HA4-1	CP022481	3095230	3096691	family "IS110"	group ""
HA4-1	CP022481	3278047	3279505	family "IS110"	group ""
HA4-1	CP022481	1712757	1713978	family "IS481"	group "-"
HA4-1	CP022481	2101433	2102654	family "IS481"	group "-"
HA4-1	CP022481	2418124	2422074	family "Tn3"	group ""
HA4-1	CP022481	2494752	2498685	family "Tn3"	group ""
HA4-1	CP022481	2336578	2339470	family "Tn3"	group ""
HA4-1	CP022481	3222310	3224925	family "IS21"	group ""
HA4-1	CP022481	3684131	3686746	family "IS21"	group ""
HA4-1	CP022481	81573	82420	family "None"	group "None"
HA4-1	CP022481	564443	565272	family "IS1595"	group "IS1016"
HA4-1	CP022481	1223574	1224462	family "IS5"	group "-"
HA4-1	CP022481	2514096	2514984	family "IS5"	group "-"
HA4-1	CP022481	1233196	1234081	family "IS5"	group "-"
HA4-1	CP022481	3698870	3699755	family "IS5"	group "-"
HA4-1	CP022481	2516087	2516959	family "IS5"	group "-"
HA4-1	CP022481	2446256	2447141	family "IS5"	group "-"
HA4-1	CP022481	2120613	2121498	family "IS5"	group "-"
HA4-1	CP022481	3849872	3850757	family "IS5"	group "-"
HA4-1	CP022481	2866067	2866921	family "IS5"	group "IS427"
HA4-1	CP022481	3201400	3202255	family "IS5"	group "IS427"
HA4-1	CP022481	2462551	2463406	family "IS5"	group "IS427"

HA4-1	CP022481	716709	717564	family "IS5"	group "IS427"
HA4-1	CP022481	2104787	2105642	family "IS5"	group "IS427"
HA4-1	CP022481	921244	922099	family "IS5"	group "IS427"
HA4-1	CP022481	2542069	2542924	family "IS5"	group "IS427"
HA4-1	CP022481	1232334	1233189	family "IS5"	group "IS427"
KACC10709	IS_3588bd79	30930	32144	family "IS5"	group "IS5"
KACC10709	IS_3588bd79	242972	244186	family "IS5"	group "IS5"
KACC10709	IS_3588bd79	3235069	3236283	family "IS5"	group "IS5"
KACC10709	IS_3588bd79	145588	146802	family "IS5"	group "IS5"
KACC10709	IS_3588bd79	76906	78085	family "IS5"	group "IS5"
KACC10709	IS_3588bd79	2925541	2926720	family "IS5"	group "IS5"
KACC10709	IS_3588bd79	152329	153505	family "IS5"	group "IS5"
KACC10709	IS_3588bd79	644219	645398	family "IS5"	group "IS5"
KACC10709	IS_3588bd79	462604	463780	family "IS5"	group "IS5"
KACC10709	IS_3588bd79	367829	369008	family "IS5"	group "IS5"
KACC10709	IS_3588bd79	2950616	2951795	family "IS5"	group "IS5"
KACC10709	IS_3588bd79	3008148	3009327	family "IS5"	group "IS5"
KACC10709	IS_3588bd79	3454618	3455797	family "IS5"	group "IS5"
KACC10709	IS_3588bd79	866868	868041	family "IS5"	group "IS5"
KACC10709	IS_3588bd79	1841261	1842437	family "IS5"	group "IS5"
KACC10709	IS_3588bd79	140396	142947	family "IS66"	group ""
KACC10709	IS_3588bd79	652228	654779	family "IS66"	group ""
KACC10709	IS_3588bd79	1581414	1583965	family "IS66"	group ""
KACC10709	IS_3588bd79	2386729	2389280	family "IS66"	group ""
KACC10709	IS_3588bd79	2991318	2993869	family "IS66"	group ""
KACC10709	IS_3588bd79	157386	159065	family "IS1182"	group ""
KACC10709	IS_3588bd79	464171	465850	family "IS1182"	group ""
KACC10709	IS_3588bd79	897260	898939	family "IS1182"	group ""
KACC10709	IS_3588bd79	1768429	1770108	family "IS1182"	group ""
KACC10709	IS_3588bd79	2104168	2105847	family "IS1182"	group ""
KACC10709	IS_3588bd79	206861	208291	family "IS4"	group ""
KACC10709	IS_3588bd79	1968176	1969606	family "IS4"	group ""
KACC10709	IS_3588bd79	2674248	2675678	family "IS4"	group ""
KACC10709	IS_3588bd79	2931182	2932612	family "IS4"	group ""
KACC10709	IS_3588bd79	3279277	3280707	family "IS4"	group ""
KACC10709	IS_3588bd79	1416448	1417878	family "IS4"	group ""
KACC10709	IS_3588bd79	3240921	3242351	family "IS4"	group ""
KACC10709	IS_3588bd79	3287347	3288777	family "IS4"	group ""
KACC10709	IS_3588bd79	810393	811823	family "IS4"	group ""
KACC10709	IS_3588bd79	2154872	2156302	family "IS4"	group ""
KACC10709	IS_3588bd79	2847050	2848480	family "IS4"	group ""
KACC10709	IS_3588bd79	564308	565738	family "IS4"	group ""
KACC10709	IS_3588bd79	2713992	2715422	family "IS4"	group ""
KACC10709	IS_3588bd79	868042	869459	family "IS4"	group ""
KACC10709	IS_3588bd79	1337600	1340279	family "None"	group "None"
KACC10709	IS_3588bd79	2510473	2513152	family "None"	group "None"
KACC10709	IS_3588bd79	1340279	1345003	family "Tn3"	group ""
KACC10709	IS_3588bd79	2505761	2510474	family "Tn3"	group ""
KACC10709	IS_3588bd79	2282929	2284377	family "IS3"	group "IS150"
KACC10709	IS_3588bd79	2885654	2887102	family "IS3"	group "IS150"
KACC10709	IS_3588bd79	2948873	2949758	family "IS5"	group "-"
KACC10709	IS_3588bd79	2982550	2983435	family "IS5"	group "-"
KACC10709	IS_3588bd79	3003347	3004733	family "IS110"	group "IS1111"
KACC10709	IS_3588bd79	3393801	3395176	family "IS110"	group "IS1111"
KACC10709	IS_3588bd79	12674	13802	family "IS5"	group "IS903"
KACC10709	IS_3588bd79	420030	421158	family "IS5"	group "IS903"
KACC10709	IS_3588bd79	682521	683649	family "IS5"	group "IS903"
KACC10709	IS_3588bd79	786220	787348	family "IS5"	group "IS903"
KACC10709	IS_3588bd79	1671899	1673027	family "IS5"	group "IS903"
KACC10709	IS_3588bd79	1752143	1753271	family "IS5"	group "IS903"
KACC10709	IS_3588bd79	2922585	2923713	family "IS5"	group "IS903"
KACC10709	IS_3588bd79	2924020	2925148	family "IS5"	group "IS903"
KACC10709	IS_3588bd79	3009629	3010757	family "IS5"	group "IS903"

KACC10709	IS_3588bd79	3236554	3237682	family "IS5"	group "IS903"
KACC10709	IS_3588bd79	3370738	3371866	family "IS5"	group "IS903"
KACC10709	IS_3588bd79	665867	666995	family "IS5"	group "IS903"
KACC10709	IS_3588bd79	3046146	3047785	family "Tn3"	group ""
KACC10709	IS_3588bd79	3005698	3006510	family "Tn3"	group ""
KACC10709	IS_3588bd79	688034	688554	family "Tn3"	group ""
CMR15	IS_8710be73	14490	15662	family "ISAs1"	group ""
CMR15	IS_8710be73	1315139	1316311	family "ISAs1"	group ""
CMR15	IS_8710be73	1486984	1488156	family "ISAs1"	group ""
CMR15	IS_8710be73	2873657	2874829	family "ISAs1"	group ""
CMR15	IS_8710be73	3257341	3258513	family "ISAs1"	group ""
CMR15	IS_8710be73	19248	20420	family "ISAs1"	group ""
CMR15	IS_8710be73	444631	446050	family "IS4"	group ""
CMR15	IS_8710be73	579585	581004	family "IS4"	group ""
CMR15	IS_8710be73	1024596	1026015	family "IS4"	group ""
CMR15	IS_8710be73	1210748	1212167	family "IS4"	group ""
CMR15	IS_8710be73	3315281	3316700	family "IS4"	group ""
CMR15	IS_8710be73	1465552	1466997	family "IS4"	group ""
CMR15	IS_8710be73	2069467	2070912	family "IS4"	group ""
CMR15	IS_8710be73	2780326	2781771	family "IS4"	group ""
CMR15	IS_8710be73	458043	458689	family "None"	group "None"
CMR15	IS_8710be73	2042498	2044829	family "IS1182"	group ""
CMR15	IS_8710be73	526699	528287	family "IS1182"	group ""
CMR15	IS_8710be73	2291195	2292783	family "IS1182"	group ""
CMR15	IS_8710be73	1151497	1153097	family "IS1182"	group ""
CMR15	IS_8710be73	2457429	2459017	family "IS1182"	group ""
CMR15	IS_8710be73	906819	908376	family "IS1182"	group ""
CMR15	IS_8710be73	3561519	3563111	family "IS1182"	group ""
CMR15	IS_8710be73	943344	944932	family "IS1182"	group ""
CMR15	IS_8710be73	2025169	2026757	family "IS1182"	group ""
CMR15	IS_8710be73	914622	916218	family "IS1182"	group ""
CMR15	IS_8710be73	992990	994586	family "IS1182"	group ""
CMR15	IS_8710be73	2627637	2629233	family "IS1182"	group ""
CMR15	IS_8710be73	2993457	2995051	family "IS1182"	group ""
CMR15	IS_8710be73	741920	743509	family "IS1182"	group ""
CMR15	IS_8710be73	1019251	1020851	family "IS1182"	group ""
CMR15	IS_8710be73	2855352	2856944	family "IS1182"	group ""
CMR15	IS_8710be73	333818	335406	family "IS1182"	group ""
CMR15	IS_8710be73	2077934	2079522	family "IS1182"	group ""
CMR15	IS_8710be73	3572704	3574288	family "IS1182"	group ""
CMR15	IS_8710be73	2017395	2018985	family "IS1182"	group ""
CMR15	IS_8710be73	3444294	3445648	family "IS110"	group "IS1111"
CMR15	IS_8710be73	1509920	1511275	family "IS110"	group "IS1111"
CMR15	IS_8710be73	3014617	3015971	family "IS110"	group "IS1111"
CMR15	IS_8710be73	3282580	3283934	family "IS110"	group "IS1111"
RS 476	CP021762	127822	128988	family "IS630"	group "-"
RS 476	CP021762	683352	684518	family "IS630"	group "-"
RS 476	CP021762	2360117	2361283	family "IS630"	group "-"
RS 476	CP021762	3659393	3660559	family "IS630"	group "-"
RS 476	CP021762	117911	119077	family "IS630"	group "-"
RS 476	CP021762	275155	277172	family "IS21"	group "-"
RS 476	CP021762	2531586	2533603	family "IS21"	group "-"
RS 476	CP021762	622258	623512	family "IS3"	group "-"
RS 476	CP021762	1661740	1662994	family "IS3"	group "-"
RS 476	CP021762	2458056	2459310	family "IS3"	group "-"
RS 476	CP021762	869917	871150	family "IS3"	group "IS407"
RS 476	CP021762	2611675	2612908	family "IS3"	group "IS407"
RS 476	CP021762	1596960	1598193	family "IS3"	group "IS407"
RS 476	CP021762	883205	884691	family "IS5"	group "IS5"
RS 476	CP021762	3365743	3367229	family "IS5"	group "IS5"
RS 476	CP021762	3395331	3396817	family "IS5"	group "IS5"
RS 476	CP021762	3695673	3697158	family "IS5"	group "IS5"

RS 476	CP021762	1538927	1540121	family "IS3"	group "IS2"
RS 476	CP021762	2001513	2002707	family "IS3"	group "IS2"
RS 476	CP021762	2530443	2531579	family "IS3"	group "IS2"
RS 476	CP021762	2613229	2614677	family "IS3"	group "IS150"
RS 476	CP021762	3326422	3327870	family "IS3"	group "IS150"
RS 476	CP021762	1540156	1541604	family "IS3"	group "IS150"
RS 476	CP021762	2456650	2458040	family "IS3"	group "IS150"
RS 476	CP021762	1541740	1542967	family "IS3"	group "IS407"
RS 476	CP021762	2910971	2912195	family "IS3"	group "IS407"
RS 476	CP021762	1589422	1590890	family "IS4"	group "IS4"
RS 476	CP021762	2047828	2049296	family "IS4"	group "IS4"
RS 476	CP021762	3498816	3500282	family "IS4"	group "IS4"
RS 476	CP021762	3427443	3428909	family "IS4"	group "IS4"
RS 476	CP021762	3429688	3432588	family "Tn3"	group ""
RS 476	CP021762	3502111	3504848	family "Tn3"	group ""
RS 476	CP021762	282784	284117	family "IS256"	group "-"
RS 476	CP021762	934785	936731	family "None"	group "None"
RS 476	CP021762	2780091	2781396	family "ISL3"	group "-"
RS 476	CP021762	3483317	3484202	family "IS5"	group "-"
RS 476	CP021762	231864	232749	family "IS5"	group "-"
RS 476	CP021762	3612033	3612918	family "IS5"	group "-"
RS 476	CP021762	1059496	1060381	family "IS5"	group "-"
RS 476	CP021762	3460588	3461473	family "IS5"	group "-"

CRMRs218

YC40-M	IS_96ce018c	29165	31716	family "IS66"	group ""
YC40-M	IS_96ce018c	643414	645965	family "IS66"	group ""
YC40-M	IS_96ce018c	923589	926140	family "IS66"	group ""
YC40-M	IS_96ce018c	1740049	1742600	family "IS66"	group ""
YC40-M	IS_96ce018c	2546746	2549297	family "IS66"	group ""
YC40-M	IS_96ce018c	2934854	2937405	family "IS66"	group ""
YC40-M	IS_96ce018c	898017	899154	family "IS5"	group "IS5"
YC40-M	IS_96ce018c	913945	915082	family "IS5"	group "IS5"
YC40-M	IS_96ce018c	1758388	1759525	family "IS5"	group "IS5"
YC40-M	IS_96ce018c	2966716	2967853	family "IS5"	group "IS5"
YC40-M	IS_96ce018c	1056512	1057645	family "IS5"	group "IS5"
YC40-M	IS_96ce018c	2845137	2846274	family "IS5"	group "IS5"
YC40-M	IS_96ce018c	2965292	2966429	family "IS5"	group "IS5"
YC40-M	IS_96ce018c	3541549	3542686	family "IS5"	group "IS5"
YC40-M	IS_96ce018c	32758	33894	family "IS5"	group "IS5"
YC40-M	IS_96ce018c	630064	631200	family "IS5"	group "IS5"
YC40-M	IS_96ce018c	1746526	1747665	family "IS5"	group "IS5"
YC40-M	IS_96ce018c	163600	165030	family "IS4"	group ""
YC40-M	IS_96ce018c	2677557	2678987	family "IS4"	group ""
YC40-M	IS_96ce018c	879798	881228	family "IS4"	group ""
YC40-M	IS_96ce018c	2709470	2710900	family "IS4"	group ""
YC40-M	IS_96ce018c	2745894	2747324	family "IS4"	group ""
YC40-M	IS_96ce018c	2778418	2779848	family "IS4"	group ""
YC40-M	IS_96ce018c	583469	584899	family "IS4"	group ""
YC40-M	IS_96ce018c	3054109	3055539	family "IS4"	group ""
YC40-M	IS_96ce018c	1885335	1886765	family "IS4"	group ""
YC40-M	IS_96ce018c	2680018	2681448	family "IS4"	group ""
YC40-M	IS_96ce018c	2633054	2634484	family "IS4"	group ""
YC40-M	IS_96ce018c	2701020	2702450	family "IS4"	group ""
YC40-M	IS_96ce018c	3515546	3516976	family "IS4"	group ""
YC40-M	IS_96ce018c	306811	308241	family "IS4"	group ""
YC40-M	IS_96ce018c	543725	545155	family "IS4"	group ""
YC40-M	IS_96ce018c	1346881	1348312	family "IS4"	group ""
YC40-M	IS_96ce018c	288181	289638	family "IS4"	group "IS50"
YC40-M	IS_96ce018c	1751105	1752560	family "IS4"	group "IS50"
YC40-M	IS_96ce018c	1179867	1181322	family "IS4"	group "IS50"
YC40-M	IS_96ce018c	2551012	2552467	family "IS4"	group "IS50"
YC40-M	IS_96ce018c	2149851	2151299	family "IS3"	group "IS150"

YC40-M	IS_96ce018c	3083874	3085322	family "IS3"	group "IS150"
YC40-M	IS_96ce018c	348727	350175	family "IS3"	group "IS150"
YC40-M	IS_96ce018c	615007	616183	family "IS5"	group "IS5"
YC40-M	IS_96ce018c	1449890	1451066	family "IS5"	group "IS5"
YC40-M	IS_96ce018c	1669895	1671071	family "IS5"	group "IS5"
YC40-M	IS_96ce018c	2915222	2916398	family "IS5"	group "IS5"
YC40-M	IS_96ce018c	2974413	2975589	family "IS5"	group "IS5"
YC40-M	IS_96ce018c	968160	969333	family "IS5"	group "IS5"
YC40-M	IS_96ce018c	2646789	2647962	family "IS5"	group "IS5"
YC40-M	IS_96ce018c	3567405	3568578	family "IS5"	group "IS5"
YC40-M	IS_96ce018c	2863082	2864255	family "IS5"	group "IS5"
YC40-M	IS_96ce018c	3333207	3334383	family "IS5"	group "IS5"
YC40-M	IS_96ce018c	3603513	3604686	family "IS5"	group "IS5"
YC40-M	IS_96ce018c	405394	406570	family "IS5"	group "IS5"
YC40-M	IS_96ce018c	434212	436493	family "IS110"	group "IS1111"
YC40-M	IS_96ce018c	3635099	3637380	family "IS110"	group "IS1111"
YC40-M	IS_96ce018c	3679119	3681400	family "IS110"	group "IS1111"
YC40-M	IS_96ce018c	3719781	3722062	family "IS110"	group "IS1111"
YC40-M	IS_96ce018c	2919859	2922133	family "IS110"	group "IS1111"
YC40-M	IS_96ce018c	2956576	2958007	family "IS110"	group "IS1111"
YC40-M	IS_96ce018c	26141	27001	family "IS110"	group "IS1111"
YC40-M	IS_96ce018c	2957997	2958848	family "IS110"	group "IS1111"
YC40-M	IS_96ce018c	620292	621749	family "IS110"	group ""
YC40-M	IS_96ce018c	1748373	1749830	family "IS110"	group ""
YC40-M	IS_96ce018c	2847094	2848551	family "IS110"	group ""
YC40-M	IS_96ce018c	3157362	3158819	family "IS110"	group ""
YC40-M	IS_96ce018c	716097	717553	family "IS110"	group ""
YC40-M	IS_96ce018c	2808403	2811001	family "Tn3"	group ""
YC40-M	IS_96ce018c	2884517	2887084	family "Tn3"	group ""
YC40-M	IS_96ce018c	2896761	2897844	family "IS30"	group "-"
YC40-M	IS_96ce018c	2972413	2973496	family "IS30"	group "-"
YC40-M	IS_96ce018c	3558906	3559989	family "IS30"	group "-"
YC40-M	IS_96ce018c	557144	557991	family "None"	group "None"
YC40-M	IS_96ce018c	2917869	2918754	family "IS5"	group "-"
YC40-M	IS_96ce018c	1732859	1733744	family "IS5"	group "-"
SN82F48	IS_98f194dd	211743	213132	family "IS256"	group ""
SN82F48	IS_98f194dd	453835	455224	family "IS256"	group ""
SN82F48	IS_98f194dd	1019500	1020889	family "IS256"	group ""
SN82F48	IS_98f194dd	1027631	1029020	family "IS256"	group ""
SN82F48	IS_98f194dd	1714726	1716115	family "IS256"	group ""
SN82F48	IS_98f194dd	2746756	2748145	family "IS256"	group ""
SN82F48	IS_98f194dd	2760586	2761975	family "IS256"	group ""
SN82F48	IS_98f194dd	2894359	2895748	family "IS256"	group ""
SN82F48	IS_98f194dd	3230071	3231460	family "IS256"	group ""
SN82F48	IS_98f194dd	327544	328991	family "ISL3"	group ""
SN82F48	IS_98f194dd	379415	380862	family "ISL3"	group ""
SN82F48	IS_98f194dd	724326	725773	family "ISL3"	group ""
SN82F48	IS_98f194dd	825842	827289	family "ISL3"	group ""
SN82F48	IS_98f194dd	846150	847597	family "ISL3"	group ""
SN82F48	IS_98f194dd	3346620	3348067	family "ISL3"	group ""
SN82F48	IS_98f194dd	3185668	3187115	family "ISL3"	group ""
SN82F48	IS_98f194dd	1221151	1222598	family "ISL3"	group ""
SN82F48	IS_98f194dd	1227726	1229173	family "ISL3"	group ""
SN82F48	IS_98f194dd	1420372	1421819	family "ISL3"	group ""
SN82F48	IS_98f194dd	1622424	1623871	family "ISL3"	group ""
SN82F48	IS_98f194dd	1665429	1666876	family "ISL3"	group ""
SN82F48	IS_98f194dd	869938	871385	family "ISL3"	group ""
SN82F48	IS_98f194dd	1578977	1580424	family "ISL3"	group ""
SN82F48	IS_98f194dd	1582289	1583736	family "ISL3"	group ""
SN82F48	IS_98f194dd	1658199	1659646	family "ISL3"	group ""
SN82F48	IS_98f194dd	3088472	3089919	family "ISL3"	group ""
SN82F48	IS_98f194dd	757335	759737	family "IS66"	group ""
SN82F48	IS_98f194dd	1258977	1261379	family "IS66"	group ""

SN82F48	IS_98f194dd	1430959	1433361	family "IS66"	group ""
SN82F48	IS_98f194dd	3496165	3498567	family "IS66"	group ""
SN82F48	IS_98f194dd	2677484	2679886	family "IS66"	group ""
SN82F48	IS_98f194dd	832069	834479	family "IS21"	group "-"
SN82F48	IS_98f194dd	853637	856047	family "IS21"	group "-"
SN82F48	IS_98f194dd	3385279	3386700	family "IS91"	group ""
SN83A39	IS_dabb08e8	7317	8862	family "IS5"	group "IS5"
SN83A39	IS_dabb08e8	2121704	2123245	family "IS5"	group "IS5"
SN83A39	IS_dabb08e8	8918	10363	family "IS5"	group "IS5"
SN83A39	IS_dabb08e8	2120203	2121648	family "IS5"	group "IS5"
SN83A39	IS_dabb08e8	1017366	1018873	family "IS91"	group ""
SEPPX05	IS_67e77903	347158	349773	family "IS21"	group ""
SEPPX05	IS_67e77903	1199870	1202485	family "IS21"	group ""
SEPPX05	IS_67e77903	1441674	1444289	family "IS21"	group ""
SEPPX05	IS_67e77903	1929876	1932491	family "IS21"	group ""
SEPPX05	IS_67e77903	2339955	2342570	family "IS21"	group ""
SEPPX05	IS_67e77903	3103528	3106143	family "IS21"	group ""
SEPPX05	IS_67e77903	3269126	3271741	family "IS21"	group ""
SEPPX05	IS_67e77903	3514543	3517158	family "IS21"	group ""
SEPPX05	IS_67e77903	2905012	2907640	family "IS21"	group ""
SEPPX05	IS_67e77903	1127801	1130062	family "IS21"	group ""
SEPPX05	IS_67e77903	418893	420323	family "IS4"	group ""
SEPPX05	IS_67e77903	2178278	2179708	family "IS4"	group ""
SEPPX05	IS_67e77903	770889	772319	family "IS4"	group ""
SEPPX05	IS_67e77903	777644	779074	family "IS4"	group ""
SEPPX05	IS_67e77903	1539012	1540442	family "IS4"	group ""
SEPPX05	IS_67e77903	3775504	3776934	family "IS4"	group ""
SEPPX05	IS_67e77903	857970	859400	family "IS4"	group ""
SEPPX05	IS_67e77903	2343425	2344855	family "IS4"	group ""
SEPPX05	IS_67e77903	3564188	3565618	family "IS4"	group ""
SEPPX05	IS_67e77903	1399375	1400805	family "IS4"	group ""
SEPPX05	IS_67e77903	1562986	1564416	family "IS4"	group ""
SEPPX05	IS_67e77903	2284247	2285677	family "IS4"	group ""
SEPPX05	IS_67e77903	1495009	1496439	family "IS4"	group ""
SEPPX05	IS_67e77903	1391315	1392734	family "IS4"	group ""
SEPPX05	IS_67e77903	610154	611474	family "IS4"	group ""
SEPPX05	IS_67e77903	2052343	2053533	family "IS4"	group ""
SEPPX05	IS_67e77903	422407	423710	family "ISL3"	group "-"
SEPPX05	IS_67e77903	590397	591700	family "ISL3"	group "-"
SEPPX05	IS_67e77903	1459217	1460520	family "ISL3"	group "-"
SEPPX05	IS_67e77903	1479560	1480863	family "ISL3"	group "-"
SEPPX05	IS_67e77903	1910492	1911795	family "ISL3"	group "-"
SEPPX05	IS_67e77903	2156869	2158172	family "ISL3"	group "-"
SEPPX05	IS_67e77903	2438480	2439783	family "ISL3"	group "-"
SEPPX05	IS_67e77903	956245	956996	family "IS3"	group "IS407"
SEPPX05	IS_67e77903	2574337	2575088	family "IS3"	group "IS407"
SEPPX05	IS_67e77903	3261825	3262576	family "IS3"	group "IS407"
SEPPX05	IS_67e77903	3522451	3523202	family "IS3"	group "IS407"
SEPPX05	IS_67e77903	1942517	1943267	family "IS3"	group "IS407"
SEPPX05	IS_67e77903	2220114	2220864	family "IS3"	group "IS407"
SEPPX05	IS_67e77903	1220858	1221575	family "IS3"	group "IS407"
SEPPX05	IS_67e77903	596025	596666	family "IS3"	group "IS407"
SEPPX05	IS_67e77903	601120	601761	family "IS3"	group "IS407"
SEPPX05	IS_67e77903	2145327	2145968	family "IS3"	group "IS407"
SEPPX05	IS_67e77903	601621	605834	family "Tn3"	group ""
SEPPX05	IS_67e77903	2141254	2145467	family "Tn3"	group ""
SEPPX05	IS_67e77903	596526	599699	family "Tn3"	group ""
SEPPX05	IS_67e77903	1136381	1136883	family "Tn3"	group ""
SEPPX05	IS_67e77903	663692	664557	family "IS5"	group "IS427"
SEPPX05	IS_67e77903	958142	959007	family "IS5"	group "IS427"
SEPPX05	IS_67e77903	1446070	1446935	family "IS5"	group "IS427"
SEPPX05	IS_67e77903	1915301	1916166	family "IS5"	group "IS427"

SEPPX05	IS_67e77903	3063466	3064331	family "IS5"	group "IS427"
SEPPX05	IS_67e77903	3796777	3797642	family "IS5"	group "IS427"
SEPPX05	IS_67e77903	878865	879720	family "IS5"	group "IS427"
SEPPX05	IS_67e77903	1401006	1401861	family "IS5"	group "IS427"
SEPPX05	IS_67e77903	880636	881491	family "IS5"	group "IS427"
SEPPX05	IS_67e77903	1912026	1912881	family "IS5"	group "IS427"
SEPPX05	IS_67e77903	721169	723520	family "IS5"	group "IS5"
SEPPX05	IS_67e77903	1550143	1552494	family "IS5"	group "IS5"
SEPPX05	IS_67e77903	486683	488460	family "IS5"	group "IS5"
SEPPX05	IS_67e77903	3201220	3202725	family "IS5"	group "IS5"
SEPPX05	IS_67e77903	3204680	3205533	family "IS5"	group "IS5"
SEPPX05	IS_67e77903	1496920	1497493	family "IS5"	group "IS5"
SEPPX05	IS_67e77903	921917	925074	family "None"	group "None"
SEPPX05	IS_67e77903	1162492	1165649	family "None"	group "None"
SEPPX05	IS_67e77903	1444619	1445484	family "IS5"	group "IS427"
SEPPX05	IS_67e77903	1825217	1826082	family "IS5"	group "IS427"
SEPPX05	IS_67e77903	3281595	3282460	family "IS5"	group "IS427"
SEPPX05	IS_67e77903	1378311	1379176	family "IS5"	group "IS427"
SEPPX05	IS_67e77903	2451964	2452829	family "IS5"	group "IS427"
SEPPX05	IS_67e77903	1778908	1779773	family "IS5"	group "IS427"
SEPPX05	IS_67e77903	1814961	1815826	family "IS5"	group "IS427"
SEPPX05	IS_67e77903	959009	959874	family "IS5"	group "IS427"
SEPPX05	IS_67e77903	2765925	2766790	family "IS5"	group "IS427"
SEPPX05	IS_67e77903	1020004	1021395	family "IS110"	group "IS1111"
SEPPX05	IS_67e77903	2578263	2579640	family "IS110"	group "IS1111"
SEPPX05	IS_67e77903	2134771	2135599	family "IS110"	group "IS1111"
SEPPX05	IS_67e77903	607644	608198	family "IS110"	group "IS1111"
SEPPX05	IS_67e77903	2137556	2138110	family "IS110"	group "IS1111"
SEPPX05	IS_67e77903	1216931	1218635	family "IS66"	group ""
SEPPX05	IS_67e77903	1944623	1946327	family "IS66"	group ""
SEPPX05	IS_67e77903	1846047	1846878	family "IS1595"	group "IS1016"
SEPPX05	IS_67e77903	3836778	3837609	family "IS1595"	group "IS1016"
SEPPX05	IS_67e77903	1900573	1902043	family "IS701"	group "-"
SEPPX05	IS_67e77903	2404239	2405709	family "IS701"	group "-"
SEPPX05	IS_67e77903	3507279	3508590	family "IS3"	group "IS2"
SEPPX05	IS_67e77903	2572412	2573723	family "IS3"	group "IS2"
SEPPX05	IS_67e77903	1454425	1455736	family "IS3"	group "IS2"
SEPPX05	IS_67e77903	2907650	2908961	family "IS3"	group "IS2"
SEPPX05	IS_67e77903	3147535	3148846	family "IS3"	group "IS2"
SEPPX05	IS_67e77903	3513070	3514381	family "IS3"	group "IS2"
SEPPX05	IS_67e77903	72147	73458	family "IS3"	group "IS2"
SEPPX05	IS_67e77903	1215610	1216921	family "IS3"	group "IS2"
SEPPX05	IS_67e77903	1504331	1505642	family "IS3"	group "IS2"
SEPPX05	IS_67e77903	2030930	2032241	family "IS3"	group "IS2"
SEPPX05	IS_67e77903	2139934	2141245	family "IS3"	group "IS2"
SEPPX05	IS_67e77903	1307252	1308366	family "IS3"	group "IS2"
SEPPX05	IS_67e77903	1941201	1942033	family "IS3"	group "IS2"
SEPPX05	IS_67e77903	357878	358690	family "IS3"	group "IS2"
SEPPX05	IS_67e77903	338696	339420	family "IS3"	group "IS2"
SEPPX05	IS_67e77903	1943268	1943768	family "IS3"	group "IS2"
SEPPX05	IS_67e77903	3876024	3877304	family "IS3"	group "IS2"
SEPPX05	IS_67e77903	2222704	2223983	family "IS3"	group "IS2"
SEPPX05	IS_67e77903	916921	918200	family "IS3"	group "IS2"
SEPPX05	IS_67e77903	3770228	3771657	family "IS3"	group "IS150"
SEPPX05	IS_67e77903	3908150	3909573	family "IS3"	group "IS150"
SEPPX05	IS_67e77903	3139699	3140584	family "IS3"	group "IS150"
SEPPX05	IS_67e77903	2847420	2847969	family "IS3"	group "IS150"
SEPPX05	IS_67e77903	3268421	3269241	family "IS21"	group "-"
SEPPX05	IS_67e77903	81008	81701	family "IS21"	group "-"
SEPPX05	IS_67e77903	335836	336529	family "IS21"	group "-"
SEPPX05	IS_67e77903	358763	359456	family "IS21"	group "-"
SEPPX05	IS_67e77903	485307	486000	family "IS21"	group "-"
SEPPX05	IS_67e77903	592379	593072	family "IS21"	group "-"
SEPPX05	IS_67e77903	608271	608964	family "IS21"	group "-"

SEPPX05	IS_67e77903	875949	876642	family "IS21"	group "-"
SEPPX05	IS_67e77903	1195247	1195940	family "IS21"	group "-"
SEPPX05	IS_67e77903	1219718	1220411	family "IS21"	group "-"
SEPPX05	IS_67e77903	1332310	1333003	family "IS21"	group "-"
SEPPX05	IS_67e77903	1451079	1451772	family "IS21"	group "-"
SEPPX05	IS_67e77903	1552595	1553288	family "IS21"	group "-"
SEPPX05	IS_67e77903	1850758	1851451	family "IS21"	group "-"
SEPPX05	IS_67e77903	2053606	2054299	family "IS21"	group "-"
SEPPX05	IS_67e77903	2136790	2137483	family "IS21"	group "-"
SEPPX05	IS_67e77903	2148921	2149614	family "IS21"	group "-"
SEPPX05	IS_67e77903	2215749	2216442	family "IS21"	group "-"
SEPPX05	IS_67e77903	2417319	2418012	family "IS21"	group "-"
SEPPX05	IS_67e77903	2487764	2488457	family "IS21"	group "-"
SEPPX05	IS_67e77903	2560238	2560931	family "IS21"	group "-"
SEPPX05	IS_67e77903	2655289	2655982	family "IS21"	group "-"
SEPPX05	IS_67e77903	2846654	2847347	family "IS21"	group "-"
SEPPX05	IS_67e77903	2911603	2912296	family "IS21"	group "-"
SEPPX05	IS_67e77903	3095411	3096104	family "IS21"	group "-"
SEPPX05	IS_67e77903	3137815	3138508	family "IS21"	group "-"
SEPPX05	IS_67e77903	3202798	3203491	family "IS21"	group "-"
SEPPX05	IS_67e77903	3338942	3339635	family "IS21"	group "-"
SEPPX05	IS_67e77903	3409447	3410140	family "IS21"	group "-"
SEPPX05	IS_67e77903	3518544	3519237	family "IS21"	group "-"
SEPPX05	IS_67e77903	3670726	3671419	family "IS21"	group "-"
SEPPX05	IS_67e77903	3910764	3911457	family "IS21"	group "-"
Rs-10-244	IS_78626702	201297	202528	family "IS3"	group "IS51"
Rs-10-244	IS_78626702	3348656	3349541	family "IS5"	group "-"
Rs-10-244	IS_78626702	3611808	3612693	family "IS5"	group "-"
Rs-09-161	IS_ef62fa1c	3404695	3406457	family "Tn3"	group ""
Rs-09-161	IS_ef62fa1c	3659941	3660826	family "IS5"	group "-"
SL3103	CP022790	9252	10382	family "IS5"	group "IS903"
SL3103	CP022790	19492	20622	family "IS5"	group "IS903"
SL3103	CP022790	209027	210157	family "IS5"	group "IS903"
SL3103	CP022790	315784	316914	family "IS5"	group "IS903"
SL3103	CP022790	1471134	1472264	family "IS5"	group "IS903"
SL3103	CP022790	1551378	1552508	family "IS5"	group "IS903"
SL3103	CP022790	1897633	1898763	family "IS5"	group "IS903"
SL3103	CP022790	2437560	2438690	family "IS5"	group "IS903"
SL3103	CP022790	2541245	2542375	family "IS5"	group "IS903"
SL3103	CP022790	2829985	2831115	family "IS5"	group "IS903"
SL3103	CP022790	3230782	3231912	family "IS5"	group "IS903"
SL3103	CP022790	3359229	3360359	family "IS5"	group "IS903"
SL3103	CP022790	2559438	2560568	family "IS5"	group "IS903"
SL3103	CP022790	210457	211638	family "IS5"	group "IS5"
SL3103	CP022790	289067	290248	family "IS5"	group "IS5"
SL3103	CP022790	3167706	3168887	family "IS5"	group "IS5"
SL3103	CP022790	3275246	3276427	family "IS5"	group "IS5"
SL3103	CP022790	13545	14721	family "IS5"	group "IS5"
SL3103	CP022790	1381964	1383140	family "IS5"	group "IS5"
SL3103	CP022790	2606918	2608099	family "IS5"	group "IS5"
SL3103	CP022790	2882124	2883305	family "IS5"	group "IS5"
SL3103	CP022790	314212	315393	family "IS5"	group "IS5"
SL3103	CP022790	2581034	2582215	family "IS5"	group "IS5"
SL3103	CP022790	3093303	3094479	family "IS5"	group "IS5"
SL3103	CP022790	15508	18059	family "IS66"	group ""
SL3103	CP022790	821992	824543	family "IS66"	group ""
SL3103	CP022790	1641288	1643839	family "IS66"	group ""
SL3103	CP022790	2571654	2574205	family "IS66"	group ""
SL3103	CP022790	3102847	3105398	family "IS66"	group ""
SL3103	CP022790	1894858	1896115	family "IS66"	group ""
SL3103	CP022790	2599879	2601350	family "IS66"	group ""

SL3103	CP022790	25668	26882	family "IS5"	group "IS5"
SL3103	CP022790	820678	821892	family "IS5"	group "IS5"
SL3103	CP022790	2605357	2606571	family "IS5"	group "IS5"
SL3103	CP022790	3100005	3100880	family "IS5"	group "IS5"
SL3103	CP022790	229819	231205	family "IS110"	group "IS1111"
SL3103	CP022790	3335920	3337295	family "IS110"	group "IS1111"
SL3103	CP022790	248557	249442	family "IS5"	group "-"
SL3103	CP022790	291105	291990	family "IS5"	group "-"
SL3103	CP022790	1780368	1781253	family "IS5"	group "-"
SL3103	CP022790	3256316	3257201	family "IS5"	group "-"
SL3103	CP022790	256494	260257	family "None"	group "None"
SL3103	CP022790	1887470	1891233	family "None"	group "None"
SL3103	CP022790	1884475	1887471	family "None"	group "None"
SL3103	CP022790	308322	309752	family "IS4"	group ""
SL3103	CP022790	3451053	3452483	family "IS4"	group ""
SL3103	CP022790	381478	382908	family "IS4"	group ""
SL3103	CP022790	392755	394185	family "IS4"	group ""
SL3103	CP022790	1254794	1256224	family "IS4"	group ""
SL3103	CP022790	549041	550471	family "IS4"	group ""
SL3103	CP022790	1807377	1808807	family "IS4"	group ""
SL3103	CP022790	3442983	3444413	family "IS4"	group ""
SL3103	CP022790	509299	510729	family "IS4"	group ""
SL3103	CP022790	1054937	1056367	family "IS4"	group ""
SL3103	CP022790	2413086	2414516	family "IS4"	group ""
SL3103	CP022790	2686577	2688007	family "IS4"	group ""
SL3103	CP022790	2356611	2358041	family "IS4"	group ""
SL3103	CP022790	278130	280098	family "None"	group "None"
SL3103	CP022790	352667	354157	family "IS3"	group "IS150"
SL3103	CP022790	926900	928348	family "IS3"	group "IS150"
SL3103	CP022790	522718	523565	family "None"	group "None"
SL3103	CP022790	995115	995944	family "IS1595"	group "IS1016"
SL3103	CP022790	1105390	1107069	family "IS1182"	group ""
SL3103	CP022790	1454292	1455971	family "IS1182"	group ""
SL3103	CP022790	2327131	2328810	family "IS1182"	group ""
SL3103	CP022790	2786472	2788151	family "IS1182"	group ""
SL3103	CP022790	2594323	2595187	family "IS5"	group "IS427"
SL3103	CP022790	6807	7662	family "IS5"	group "IS427"
T117	CP022755	1399616	1400795	family "IS5"	group "IS5"
T117	CP022755	2541699	2542878	family "IS5"	group "IS5"
T117	CP022755	3412731	3413910	family "IS5"	group "IS5"
T117	CP022755	1210383	1211559	family "IS5"	group "IS5"
T117	CP022755	1273726	1274902	family "IS5"	group "IS5"
T117	CP022755	346765	347939	family "IS5"	group "IS5"
T117	CP022755	288574	289747	family "IS5"	group "IS5"
T117	CP022755	340870	342300	family "IS4"	group ""
T117	CP022755	661151	662581	family "IS4"	group ""
T117	CP022755	2160656	2162086	family "IS4"	group ""
T117	CP022755	420238	421668	family "IS4"	group ""
T117	CP022755	536764	538194	family "IS4"	group ""
T117	CP022755	1097903	1099333	family "IS4"	group ""
T117	CP022755	1423955	1425385	family "IS4"	group ""
T117	CP022755	2668618	2670048	family "IS4"	group ""
T117	CP022755	2864606	2866036	family "IS4"	group ""
T117	CP022755	3706616	3708046	family "IS4"	group ""
T117	CP022755	1328699	1330129	family "IS4"	group ""
T117	CP022755	1480431	1481861	family "IS4"	group ""
T117	CP022755	737422	738895	family "IS4"	group "IS50"
T117	CP022755	1298448	1299921	family "IS4"	group "IS50"
T117	CP022755	3621218	3622691	family "IS4"	group "IS50"
T117	CP022755	964393	965841	family "IS3"	group "IS150"
T117	CP022755	1900821	1902269	family "IS3"	group "IS150"
T117	CP022755	1225688	1229546	family "Tn3"	group ""
T117	CP022755	1294589	1298447	family "Tn3"	group ""

T117	CP022755	1271370	1272255	family "IS5"	group "-"
T117	CP022755	3620255	3621140	family "IS5"	group "-"
T117	CP022755	1798436	1799321	family "IS5"	group "-"
T117	CP022755	1274904	1276117	family "IS5"	group "IS5"
T117	CP022755	3463934	3465141	family "IS5"	group "IS5"
T117	CP022755	2978620	2979830	family "IS5"	group "IS5"
T117	CP022755	550183	551030	family "None"	group "None"
T117	CP022755	1038098	1038927	family "IS1595"	group "IS1016"
T117	CP022755	1251497	1252728	family "IS3"	group "IS51"
T117	CP022755	2778349	2779453	family "IS3"	group "IS51"
T117	CP022755	2813959	2814727	family "IS3"	group "IS51"
T117	CP022755	1035030	1035685	family "IS3"	group "IS51"
SL2330	CP022794	6006	7185	family "IS5"	group "IS5"
SL2330	CP022794	341716	342897	family "IS5"	group "IS5"
SL2330	CP022794	309946	311653	family "None"	group "None"
SL2330	CP022794	2713400	2715107	family "None"	group "None"
SL2330	CP022794	322016	322881	family "IS5"	group "IS427"
SL2330	CP022794	3104289	3105154	family "IS5"	group "IS427"
SL2330	CP022794	3114545	3115410	family "IS5"	group "IS427"
SL2330	CP022794	1195914	1196779	family "IS5"	group "IS427"
SL2330	CP022794	537917	539347	family "IS4"	group ""
SL2330	CP022794	577664	579094	family "IS4"	group ""
SL2330	CP022794	1328686	1330116	family "IS4"	group ""
SL2330	CP022794	3365141	3366571	family "IS4"	group ""
SL2330	CP022794	2587759	2589189	family "IS4"	group ""
SL2330	CP022794	1842185	1843615	family "IS4"	group ""
SL2330	CP022794	1303702	1305132	family "IS4"	group ""
SL2330	CP022794	3641034	3642464	family "IS4"	group ""
SL2330	CP022794	2895857	2897287	family "IS4"	group ""
SL2330	CP022794	1196778	1197985	family "IS3"	group "IS3"
SL2330	CP022794	2816505	2817710	family "IS3"	group "IS3"
SL2330	CP022794	2710474	2713151	family "Tn3"	group ""
SL2330	CP022794	2799295	2801972	family "Tn3"	group ""
SL2330	CP022794	242415	242929	family "Tn3"	group ""
SL2330	CP022794	2769639	2770870	family "IS3"	group "IS51"
SL2330	CP022794	3453345	3454576	family "IS3"	group "IS51"
SL2330	CP022794	1184005	1185243	family "IS3"	group "IS51"
SL2330	CP022794	3031294	3032764	family "IS701"	group "-"
SL2330	CP022794	3455551	3457021	family "IS701"	group "-"
SL2330	CP022794	11937	16672	family "ISL3"	group ""
SL2330	CP022794	383766	385214	family "IS3"	group "IS150"
SL2330	CP022794	551336	552184	family "None"	group "None"
SL2330	CP022794	2781763	2782645	family "IS5"	group "-"
SL2330	CP022794	1695823	1696702	family "IS5"	group "-"
SL2330	CP022794	2804238	2805123	family "IS5"	group "-"
SL2330	CP022794	1337039	1337918	family "IS5"	group "-"
SL2330	CP022794	1915027	1915912	family "IS5"	group "-"
SL2330	CP022794	2780771	2781650	family "IS5"	group "-"
SL2330	CP022794	3454662	3455547	family "IS5"	group "-"
SL2330	CP022794	2736086	2736971	family "IS5"	group "-"
SL2330	CP022794	2806122	2807007	family "IS5"	group "-"
SL3755	CP022782	210990	213940	family "Tn3"	group ""
SL3755	CP022782	283682	286574	family "Tn3"	group ""
SL3755	CP022782	287235	288414	family "IS5"	group "IS5"
SL3755	CP022782	2745570	2746747	family "IS5"	group "IS5"
SL3755	CP022782	310282	311458	family "IS5"	group "IS5"
SL3755	CP022782	506480	507910	family "IS4"	group ""
SL3755	CP022782	1305965	1307395	family "IS4"	group ""
SL3755	CP022782	3480308	3481738	family "IS4"	group ""
SL3755	CP022782	546224	547654	family "IS4"	group ""
SL3755	CP022782	2606764	2608194	family "IS4"	group ""
SL3755	CP022782	1818713	1820143	family "IS4"	group ""

SL3755	CP022782	3754944	3756374	family "IS4"	group ""
SL3755	CP022782	1280981	1282411	family "IS4"	group ""
SL3755	CP022782	2993147	2994577	family "IS4"	group ""
SL3755	CP022782	1174031	1175268	family "IS3"	group "IS3"
SL3755	CP022782	2913745	2914980	family "IS3"	group "IS3"
SL3755	CP022782	2727307	2730222	family "Tn3"	group ""
SL3755	CP022782	2880971	2883866	family "Tn3"	group ""
SL3755	CP022782	2803771	2806548	family "Tn3"	group ""
SL3755	CP022782	3128613	3130083	family "IS701"	group "-"
SL3755	CP022782	3569473	3570943	family "IS701"	group "-"
SL3755	CP022782	264311	265195	family "IS5"	group "-"
SL3755	CP022782	2903392	2904277	family "IS5"	group "-"
SL3755	CP022782	1672352	1673237	family "IS5"	group "-"
SL3755	CP022782	2900516	2901398	family "IS5"	group "-"
SL3755	CP022782	1314315	1315200	family "IS5"	group "-"
SL3755	CP022782	1891511	1892396	family "IS5"	group "-"
SL3755	CP022782	2901508	2902393	family "IS5"	group "-"
SL3755	CP022782	3568584	3569469	family "IS5"	group "-"
SL3755	CP022782	2833363	2834248	family "IS5"	group "-"
SL3755	CP022782	2878039	2878924	family "IS5"	group "-"
SL3755	CP022782	352332	353780	family "IS3"	group "IS150"
SL3755	CP022782	519896	520744	family "None"	group "None"
T25	CP023014	284736	286443	family "None"	group "None"
T25	CP023014	2737946	2739653	family "None"	group "None"
T25	CP023014	1170066	1171303	family "IS3"	group "IS3"
T25	CP023014	2842175	2843410	family "IS3"	group "IS3"
T25	CP023014	1895331	1896216	family "IS5"	group "-"
T25	CP023014	3497293	3498178	family "IS5"	group "-"
T25	CP023014	2806475	2807360	family "IS5"	group "-"
T25	CP023014	1310328	1311213	family "IS5"	group "-"
T25	CP023014	2829941	2830826	family "IS5"	group "-"
T25	CP023014	2760629	2761514	family "IS5"	group "-"
T25	CP023014	1669025	1669909	family "IS5"	group "-"
T25	CP023014	2828950	2829831	family "IS5"	group "-"
T25	CP023014	2831825	2832710	family "IS5"	group "-"
T25	CP023014	2735020	2737697	family "Tn3"	group ""
T25	CP023014	2809626	2812303	family "Tn3"	group ""
T25	CP023014	216591	217105	family "Tn3"	group ""
T25	CP023014	315628	316803	family "IS5"	group "IS5"
T25	CP023014	2782390	2783565	family "IS5"	group "IS5"
T25	CP023014	1675007	1676183	family "IS5"	group "IS5"
T25	CP023014	357677	359125	family "IS3"	group "IS150"
T25	CP023014	525221	526069	family "None"	group "None"
T25	CP023014	2612330	2613797	family "IS4"	group ""
T25	CP023014	511804	513236	family "IS4"	group ""
T25	CP023014	551547	552977	family "IS4"	group ""
T25	CP023014	1301979	1303409	family "IS4"	group ""
T25	CP023014	3408942	3410372	family "IS4"	group ""
T25	CP023014	1822562	1823992	family "IS4"	group ""
T25	CP023014	1276999	1278429	family "IS4"	group ""
T25	CP023014	2921559	2922991	family "IS4"	group ""
T25	CP023014	3682121	3683553	family "IS4"	group ""
T25	CP023014	3138731	3139603	family "IS5"	group "IS427"
T25	CP023014	3128485	3129350	family "IS5"	group "IS427"
T25	CP023014	1678892	1679756	family "IS5"	group "IS427"
T78	CP022765	298898	300328	family "IS4"	group ""
T78	CP022765	709180	710610	family "IS4"	group ""
T78	CP022765	1892067	1893497	family "IS4"	group ""
T78	CP022765	535699	537129	family "IS4"	group ""
T78	CP022765	1341912	1343342	family "IS4"	group ""
T78	CP022765	2617277	2618707	family "IS4"	group ""
T78	CP022765	3916577	3918007	family "IS4"	group ""

T78	CP022765	1145924	1147354	family "IS4"	group ""
T78	CP022765	2673734	2675164	family "IS4"	group ""
T78	CP022765	2768978	2770408	family "IS4"	group ""
T78	CP022765	3077798	3079228	family "IS4"	group ""
T78	CP022765	378270	379624	family "IS4"	group ""
T78	CP022765	1469081	1470261	family "IS5"	group "IS5"
T78	CP022765	2698324	2699504	family "IS5"	group "IS5"
T78	CP022765	3624537	3625717	family "IS5"	group "IS5"
T78	CP022765	379625	380801	family "IS5"	group "IS5"
T78	CP022765	2517683	2518859	family "IS5"	group "IS5"
T78	CP022765	2902234	2903410	family "IS5"	group "IS5"
T78	CP022765	2965573	2966749	family "IS5"	group "IS5"
T78	CP022765	3482926	3484102	family "IS5"	group "IS5"
T78	CP022765	304793	305967	family "IS5"	group "IS5"
T78	CP022765	400465	401672	family "IS5"	group "IS5"
T78	CP022765	3675739	3676946	family "IS5"	group "IS5"
T78	CP022765	2901022	2902229	family "IS5"	group "IS5"
T78	CP022765	908443	909916	family "IS4"	group "IS50"
T78	CP022765	2877215	2878688	family "IS4"	group "IS50"
T78	CP022765	599939	601412	family "IS4"	group "IS50"
T78	CP022765	1012415	1013863	family "IS3"	group "IS150"
T78	CP022765	2153180	2154628	family "IS3"	group "IS150"
T78	CP022765	2904881	2905766	family "IS5"	group "-"
T78	CP022765	2256153	2257038	family "IS5"	group "-"
T78	CP022765	3831701	3832586	family "IS5"	group "-"
T78	CP022765	2878689	2882546	family "Tn3"	group ""
T78	CP022765	2947587	2951444	family "Tn3"	group ""
T78	CP022765	2797259	2799896	family "Tn3"	group ""
T78	CP022765	2924406	2925637	family "IS3"	group "IS51"
T78	CP022765	3213036	3214267	family "IS3"	group "IS51"
T78	CP022765	1232508	1233612	family "IS3"	group "IS51"
T78	CP022765	1197234	1198002	family "IS3"	group "IS51"
T78	CP022765	1083052	1083707	family "IS3"	group "IS51"
T78	CP022765	549118	549965	family "None"	group "None"
T78	CP022765	625297	629063	family "ISL3"	group ""
T78	CP022765	1086120	1086949	family "IS1595"	group "IS1016"
T78	CP022765	1867294	1868424	family "IS5"	group "IS903"
SL3730	CP022784	14884	16014	family "IS5"	group "IS903"
SL3730	CP022784	696939	698069	family "IS5"	group "IS903"
SL3730	CP022784	838503	839633	family "IS5"	group "IS903"
SL3730	CP022784	3001800	3002930	family "IS5"	group "IS903"
SL3730	CP022784	3004363	3005493	family "IS5"	group "IS903"
SL3730	CP022784	3044193	3045323	family "IS5"	group "IS903"
SL3730	CP022784	3392938	3394068	family "IS5"	group "IS903"
SL3730	CP022784	1483326	1484505	family "IS5"	group "IS5"
SL3730	CP022784	2647086	2648265	family "IS5"	group "IS5"
SL3730	CP022784	2782517	2783696	family "IS5"	group "IS5"
SL3730	CP022784	3320949	3322128	family "IS5"	group "IS5"
SL3730	CP022784	3381677	3382856	family "IS5"	group "IS5"
SL3730	CP022784	305234	306410	family "IS5"	group "IS5"
SL3730	CP022784	1649509	1650685	family "IS5"	group "IS5"
SL3730	CP022784	2764630	2765806	family "IS5"	group "IS5"
SL3730	CP022784	3441648	3442824	family "IS5"	group "IS5"
SL3730	CP022784	245580	246753	family "IS5"	group "IS5"
SL3730	CP022784	281609	283082	family "IS4"	group "IS50"
SL3730	CP022784	698867	700340	family "IS4"	group "IS50"
SL3730	CP022784	992300	993773	family "IS4"	group "IS50"
SL3730	CP022784	299339	300769	family "IS4"	group ""
SL3730	CP022784	621666	623096	family "IS4"	group ""
SL3730	CP022784	1884195	1885625	family "IS4"	group ""
SL3730	CP022784	380762	382192	family "IS4"	group ""
SL3730	CP022784	497280	498710	family "IS4"	group ""
SL3730	CP022784	1100827	1102257	family "IS4"	group ""

SL3730	CP022784	1356162	1357592	family "IS4"	group ""
SL3730	CP022784	2566040	2567470	family "IS4"	group ""
SL3730	CP022784	2651667	2653097	family "IS4"	group ""
SL3730	CP022784	2719179	2720609	family "IS4"	group ""
SL3730	CP022784	2897223	2898653	family "IS4"	group ""
SL3730	CP022784	3677433	3678863	family "IS4"	group ""
SL3730	CP022784	2622497	2623927	family "IS4"	group ""
SL3730	CP022784	706780	708011	family "IS3"	group "IS51"
SL3730	CP022784	1037802	1039033	family "IS3"	group "IS51"
SL3730	CP022784	1155521	1156752	family "IS3"	group "IS51"
SL3730	CP022784	3315437	3316668	family "IS3"	group "IS51"
SL3730	CP022784	1246759	1247866	family "IS3"	group "IS51"
SL3730	CP022784	847995	849210	family "IS5"	group "IS5"
SL3730	CP022784	3312142	3313357	family "IS5"	group "IS5"
SL3730	CP022784	3335350	3336565	family "IS5"	group "IS5"
SL3730	CP022784	3505500	3506715	family "IS5"	group "IS5"
SL3730	CP022784	724044	725251	family "IS5"	group "IS5"
SL3730	CP022784	951929	953274	family "IS3"	group "IS150"
SL3730	CP022784	2145622	2146967	family "IS3"	group "IS150"
SL3730	CP022784	1029012	1030691	family "IS1182"	group ""
SL3730	CP022784	2997795	2999474	family "IS1182"	group ""
SL3730	CP022784	1486104	1486989	family "IS5"	group "-"
SL3730	CP022784	2248576	2249461	family "IS5"	group "-"
SL3730	CP022784	510699	511546	family "None"	group "None"
SL3730	CP022784	1041022	1041851	family "IS1595"	group "IS1016"
12D	IS_e546712d	92525	93858	family "IS256"	group "-"
12D	IS_e546712d	266113	267446	family "IS256"	group "-"
12D	IS_e546712d	752844	754177	family "IS256"	group "-"
12D	IS_e546712d	1346731	1348064	family "IS256"	group "-"
12D	IS_e546712d	3308692	3310025	family "IS256"	group "-"
12D	IS_e546712d	3352036	3353369	family "IS256"	group "-"
12D	IS_e546712d	9592	10925	family "IS256"	group "-"
12D	IS_e546712d	870005	872407	family "IS66"	group ""
12D	IS_e546712d	3132496	3134898	family "IS66"	group ""
12D	IS_e546712d	2813198	2815600	family "IS66"	group ""
12D	IS_e546712d	2076952	2078232	family "IS3"	group "IS3"
12D	IS_e546712d	3401855	3403135	family "IS3"	group "IS3"
12D	IS_e546712d	3224332	3225888	family "IS30"	group "-"
12D	IS_e546712d	3339580	3340703	family "IS30"	group "-"
12D	IS_e546712d	678836	682035	family "Tn3"	group "-"
12D	IS_e546712d	768109	769556	family "ISL3"	group ""
12D	IS_e546712d	2987230	2988548	family "IS3"	group "IS51"
DTP0602	IS_b9bc5a47	17435	18918	family "IS4"	group "IS50"
DTP0602	IS_b9bc5a47	569811	571291	family "IS4"	group "IS50"
DTP0602	IS_b9bc5a47	238557	240288	family "IS1182"	group ""
DTP0602	IS_b9bc5a47	3397472	3399179	family "IS1182"	group ""
DTP0602	IS_b9bc5a47	3395707	3397473	family "IS200--IS605"	group ""
DTP0602	IS_b9bc5a47	4460324	4462090	family "IS200--IS605"	group ""
DTP0602	IS_b9bc5a47	240278	242041	family "IS200--IS605"	group ""
DTP0602	IS_b9bc5a47	4266761	4268527	family "IS200--IS605"	group ""
DTP0602	IS_b9bc5a47	1455248	1456109	family "IS5"	group "IS427"
DTP0602	IS_b9bc5a47	2720938	2721799	family "IS5"	group "IS427"
DTP0602	IS_b9bc5a47	1628356	1630267	family "IS1634"	group ""
DTP0602	IS_b9bc5a47	4470894	4472794	family "IS1634"	group ""
DTP0602	IS_b9bc5a47	2217332	2218440	family "IS630"	group ""
DTP0602	IS_b9bc5a47	2272716	2273822	family "IS630"	group ""
DTP0602	IS_b9bc5a47	1831510	1832617	family "IS630"	group ""
DTP0602	IS_b9bc5a47	80000	81303	family "None"	group "None"
DTP0602	IS_b9bc5a47	603316	605616	family "IS607"	group ""
DTP0602	IS_b9bc5a47	1269933	1271260	family "IS110"	group ""
DTP0602	IS_b9bc5a47	1765471	1766489	family "ISKra4"	group "ISAzba1"

DTP0602	IS_b9bc5a47	1985713	1986818	family "IS630"	group ""
DTP0602	IS_b9bc5a47	3522189	3523293	family "IS630"	group ""
DTP0602	IS_b9bc5a47	2094881	2096312	family "Tn3"	group ""
DTP0602	IS_b9bc5a47	2656117	2658056	family "IS1634"	group ""
DTP0602	IS_b9bc5a47	2691268	2692796	family "IS4"	group "IS50"
DTP0602	IS_b9bc5a47	3266929	3268457	family "IS256"	group ""
FC1138	IS_fdddf1d6	804131	807395	family "IS3"	group "IS3"
FC1138	IS_fdddf1d6	2299463	2302727	family "IS3"	group "IS3"
FC1138	IS_fdddf1d6	92601	94595	family "IS3"	group "IS3"
FC1138	IS_fdddf1d6	388681	390637	family "IS3"	group "IS3"
FC1138	IS_fdddf1d6	3330368	3332324	family "IS3"	group "IS3"
FC1138	IS_fdddf1d6	2728764	2730720	family "IS3"	group "IS3"
FC1138	IS_fdddf1d6	94558	95836	family "IS3"	group "IS3"
FC1138	IS_fdddf1d6	289102	291504	family "IS66"	group ""
FC1138	IS_fdddf1d6	1454226	1456628	family "IS66"	group ""
FC1138	IS_fdddf1d6	3445306	3447708	family "IS66"	group ""
FC1138	IS_fdddf1d6	2548739	2550191	family "IS4"	group "IS4"
FC1138	IS_fdddf1d6	3131507	3132959	family "IS4"	group "IS4"
FC1138	IS_fdddf1d6	3138327	3139779	family "IS4"	group "IS4"
FC1138	IS_fdddf1d6	2798093	2798926	family "IS5"	group "IS427"
FC1138	IS_fdddf1d6	3895525	3897231	family "IS91"	group "-"
T110	IS_b246579f	1168496	1169703	family "IS3"	group "IS3"
T110	IS_b246579f	2787847	2789052	family "IS3"	group "IS3"
T110	IS_b246579f	1887306	1888191	family "IS5"	group "-"
T110	IS_b246579f	3425398	3426283	family "IS5"	group "-"
T110	IS_b246579f	2752133	2753018	family "IS5"	group "-"
T110	IS_b246579f	1308615	1309500	family "IS5"	group "-"
T110	IS_b246579f	2775583	2776468	family "IS5"	group "-"
T110	IS_b246579f	2707500	2708385	family "IS5"	group "-"
T110	IS_b246579f	1668274	1669158	family "IS5"	group "-"
T110	IS_b246579f	2774591	2775473	family "IS5"	group "-"
T110	IS_b246579f	2777467	2778352	family "IS5"	group "-"
T110	IS_b246579f	314092	315267	family "IS5"	group "IS5"
T110	IS_b246579f	3448126	3449300	family "IS5"	group "IS5"
T110	IS_b246579f	1401818	1402992	family "IS5"	group "IS5"
T110	IS_b246579f	3302449	3303621	family "IS5"	group "IS5"
T110	IS_b246579f	356097	357542	family "IS3"	group "IS150"
T110	IS_b246579f	510063	511530	family "IS4"	group ""
T110	IS_b246579f	549801	551231	family "IS4"	group ""
T110	IS_b246579f	3337187	3338617	family "IS4"	group ""
T110	IS_b246579f	1300276	1301706	family "IS4"	group ""
T110	IS_b246579f	2559312	2560744	family "IS4"	group ""
T110	IS_b246579f	1814528	1815958	family "IS4"	group ""
T110	IS_b246579f	3611310	3612744	family "IS4"	group ""
T110	IS_b246579f	1275312	1276742	family "IS4"	group ""
T110	IS_b246579f	2867121	2868557	family "IS4"	group ""
T110	IS_b246579f	3002470	3003939	family "IS701"	group "-"
T110	IS_b246579f	3612938	3613803	family "IS5"	group "IS427"
T110	IS_b246579f	1039798	1040662	family "IS5"	group "IS427"
SL2729	CP022792	14885	16015	family "IS5"	group "IS903"
SL2729	CP022792	697017	698147	family "IS5"	group "IS903"
SL2729	CP022792	838585	839715	family "IS5"	group "IS903"
SL2729	CP022792	3002024	3003154	family "IS5"	group "IS903"
SL2729	CP022792	3004587	3005717	family "IS5"	group "IS903"
SL2729	CP022792	3044421	3045551	family "IS5"	group "IS903"
SL2729	CP022792	1483437	1484616	family "IS5"	group "IS5"
SL2729	CP022792	2647297	2648476	family "IS5"	group "IS5"
SL2729	CP022792	2782740	2783919	family "IS5"	group "IS5"
SL2729	CP022792	3319974	3321153	family "IS5"	group "IS5"
SL2729	CP022792	3380705	3381884	family "IS5"	group "IS5"
SL2729	CP022792	305289	306465	family "IS5"	group "IS5"

SL2729	CP022792	1649623	1650799	family "IS5"	group "IS5"
SL2729	CP022792	2764854	2766030	family "IS5"	group "IS5"
SL2729	CP022792	3439538	3440714	family "IS5"	group "IS5"
SL2729	CP022792	245615	246788	family "IS5"	group "IS5"
SL2729	CP022792	281661	283134	family "IS4"	group "IS50"
SL2729	CP022792	698945	700418	family "IS4"	group "IS50"
SL2729	CP022792	992390	993863	family "IS4"	group "IS50"
SL2729	CP022792	299394	300824	family "IS4"	group ""
SL2729	CP022792	621738	623168	family "IS4"	group ""
SL2729	CP022792	1884382	1885812	family "IS4"	group ""
SL2729	CP022792	380825	382255	family "IS4"	group ""
SL2729	CP022792	497351	498781	family "IS4"	group ""
SL2729	CP022792	1100918	1102348	family "IS4"	group ""
SL2729	CP022792	1356267	1357697	family "IS4"	group ""
SL2729	CP022792	2566249	2567679	family "IS4"	group ""
SL2729	CP022792	2651878	2653308	family "IS4"	group ""
SL2729	CP022792	2719402	2720832	family "IS4"	group ""
SL2729	CP022792	2897442	2898872	family "IS4"	group ""
SL2729	CP022792	3675338	3676768	family "IS4"	group ""
SL2729	CP022792	2622707	2624137	family "IS4"	group ""
SL2729	CP022792	706858	708089	family "IS3"	group "IS51"
SL2729	CP022792	1037893	1039124	family "IS3"	group "IS51"
SL2729	CP022792	1155613	1156845	family "IS3"	group "IS51"
SL2729	CP022792	3314462	3315693	family "IS3"	group "IS51"
SL2729	CP022792	1246860	1247966	family "IS3"	group "IS51"
SL2729	CP022792	848077	849292	family "IS5"	group "IS5"
SL2729	CP022792	3334376	3335591	family "IS5"	group "IS5"
SL2729	CP022792	3503399	3504614	family "IS5"	group "IS5"
SL2729	CP022792	724123	725330	family "IS5"	group "IS5"
SL2729	CP022792	951912	953360	family "IS3"	group "IS150"
SL2729	CP022792	2145823	2147271	family "IS3"	group "IS150"
SL2729	CP022792	1029102	1030781	family "IS1182"	group ""
SL2729	CP022792	2998019	2999698	family "IS1182"	group ""
SL2729	CP022792	1486215	1487100	family "IS5"	group "-"
SL2729	CP022792	2248780	2249665	family "IS5"	group "-"
SL2729	CP022792	510770	511617	family "None"	group "None"
SL2729	CP022792	1041113	1041942	family "IS1595"	group "IS1016"
RS_T51	NZ_CP022770	429169	429815	family "None"	group "None"
RS_T51	NZ_CP022770	3459102	3460475	family "None"	group "None"
SL3175	NZ_CP022788	173383	174214	family "IS5"	group "IS427"
SL3175	NZ_CP022788	2541065	2541896	family "IS5"	group "IS427"
SL3175	NZ_CP022788	2551767	2553008	family "IS3"	group "IS407"
SL3175	NZ_CP022788	548724	549957	family "IS3"	group "IS407"
SL3175	NZ_CP022788	2702108	2703343	family "IS3"	group "IS407"
SL3175	NZ_CP022788	370061	371294	family "IS3"	group "IS407"
SL3175	NZ_CP022788	1470113	1471346	family "IS3"	group "IS407"
SL3175	NZ_CP022788	2693712	2694949	family "IS3"	group "IS407"
SL3175	NZ_CP022788	2920756	2921993	family "IS3"	group "IS407"
SL3175	NZ_CP022788	219397	219985	family "IS3"	group "IS407"
SL3175	NZ_CP022788	445971	446617	family "None"	group "None"
SL3175	NZ_CP022788	3510111	3511479	family "None"	group "None"
T11	NZ_CP022776	428923	429569	family "None"	group "None"
T11	NZ_CP022776	3508831	3510204	family "None"	group "None"
T98	NZ_CP022759	173380	174211	family "IS5"	group "IS427"
T98	NZ_CP022759	2541049	2541880	family "IS5"	group "IS427"
T98	NZ_CP022759	2551751	2552992	family "IS3"	group "IS407"
T98	NZ_CP022759	548720	549953	family "IS3"	group "IS407"
T98	NZ_CP022759	2702091	2703326	family "IS3"	group "IS407"
T98	NZ_CP022759	370058	371291	family "IS3"	group "IS407"
T98	NZ_CP022759	1470108	1471341	family "IS3"	group "IS407"

T98	NZ_CP022759	2693695	2694932	family "IS3"	group "IS407"
T98	NZ_CP022759	2920744	2921981	family "IS3"	group "IS407"
T98	NZ_CP022759	219394	219982	family "IS3"	group "IS407"
T98	NZ_CP022759	445968	446614	family "None"	group "None"
T98	NZ_CP022759	3510098	3511466	family "None"	group "None"
T12	CP022774	3474639	3476012	family "None"	group "None"
SL3022	CP023016	361207	362363	family "IS3"	group "IS407"
SL3022	CP023016	437062	437708	family "None"	group "None"
SL3022	CP023016	2596571	2597821	family "IS3"	group "IS407"
SL3022	CP023016	2720174	2721412	family "IS3"	group "IS407"
SL3022	CP023016	2600729	2601583	family "IS5"	group "IS427"
SL3022	CP023016	1640861	1641716	family "IS5"	group "IS427"
SL3022	CP023016	2604390	2605245	family "IS5"	group "IS427"
SL3022	CP023016	3512286	3513659	family "None"	group "None"
SL2064	CP022798	503511	504157	family "None"	group "None"
SL2064	CP022798	3531224	3532597	family "None"	group "None"
SL2312	IS_2596d515	3474913	3476286	family "None"	group "None"
T101	CP022758	1784744	1786011	family "IS5"	group "IS5"
T82	CP022763	3474912	3476285	family "None"	group "None"
T95	CP022761	503511	504157	family "None"	group "None"
T95	CP022761	3532111	3533484	family "None"	group "None"
12J	IS_75882f3f	10873	12197	family "IS3"	group "IS51"
12J	IS_75882f3f	1917829	1919153	family "IS3"	group "IS51"
12J	IS_75882f3f	295068	295928	family "IS5"	group "IS1031"
12J	IS_75882f3f	298783	299643	family "IS5"	group "IS1031"
12J	IS_75882f3f	889753	890613	family "IS5"	group "IS1031"
12J	IS_75882f3f	1564445	1565305	family "IS5"	group "IS1031"
12J	IS_75882f3f	1196679	1197961	family "IS3"	group "IS3"
12J	IS_75882f3f	2784957	2786239	family "IS3"	group "IS3"
12J	IS_75882f3f	1079008	1080290	family "IS3"	group "IS3"
12J	IS_75882f3f	3788524	3789806	family "IS3"	group "IS3"
12J	IS_75882f3f	2898704	2899986	family "IS3"	group "IS3"
12J	IS_75882f3f	1235079	1236202	family "IS630"	group ""
12J	IS_75882f3f	1580659	1581782	family "IS630"	group ""
12J	IS_75882f3f	2873446	2874568	family "IS630"	group ""
12J	IS_75882f3f	1851001	1852122	family "IS630"	group ""
12J	IS_75882f3f	1257302	1259704	family "IS66"	group ""
12J	IS_75882f3f	1575711	1578113	family "IS66"	group ""
12J	IS_75882f3f	3471118	3473520	family "IS66"	group ""
12J	IS_75882f3f	1568679	1573272	family "Tn3"	group ""
12J	IS_75882f3f	1942948	1947541	family "Tn3"	group ""
12J	IS_75882f3f	3876260	3877810	family "IS91"	group "-"
ATCC49129_1	IS_8802d314	2306354	2307540	family "IS5"	group "IS5"
ATCC49129_1	IS_8802d314	2307651	2309157	family "IS110"	group "IS1111"
A2HRMARDI	CP019911	823617	824362	family "IS5"	group "IS5"
A2HRMARDI	CP019911	1112965	1113710	family "IS5"	group "IS5"
A2HRMARDI	CP019911	1299634	1300379	family "IS5"	group "IS5"
A2HRMARDI	CP019911	1419219	1419964	family "IS5"	group "IS5"
A2HRMARDI	CP019911	1496183	1496928	family "IS5"	group "IS5"
A2HRMARDI	CP019911	2203706	2204451	family "IS5"	group "IS5"
A2HRMARDI	CP019911	2206690	2207435	family "IS5"	group "IS5"
A2HRMARDI	CP019911	2259444	2260189	family "IS5"	group "IS5"
A2HRMARDI	CP019911	2673197	2673942	family "IS5"	group "IS5"
A2HRMARDI	CP019911	2949268	2950013	family "IS5"	group "IS5"

A2HRMARDI	CP019911	3559166	3559911	family "IS5"	group "IS5"
A2HRMARDI	CP019911	3433492	3434236	family "IS5"	group "IS5"
A2HRMARDI	CP019911	1250201	1250946	family "IS5"	group "IS5"
A2HRMARDI	CP019911	2046021	2046766	family "IS5"	group "IS5"
A2HRMARDI	CP019911	2321638	2322383	family "IS5"	group "IS5"
A2HRMARDI	CP019911	2699146	2699891	family "IS5"	group "IS5"
A2HRMARDI	CP019911	3443891	3444636	family "IS5"	group "IS5"
A2HRMARDI	CP019911	1613698	1614442	family "IS5"	group "IS5"
A2HRMARDI	CP019911	3286895	3287639	family "IS5"	group "IS5"
A2HRMARDI	CP019911	1922268	1923013	family "IS5"	group "IS5"
A2HRMARDI	CP019911	1113767	1114450	family "IS5"	group "IS5"
A2HRMARDI	CP019911	1298894	1299577	family "IS5"	group "IS5"
A2HRMARDI	CP019911	1612958	1613641	family "IS5"	group "IS5"
A2HRMARDI	CP019911	2204508	2205191	family "IS5"	group "IS5"
A2HRMARDI	CP019911	2205950	2206633	family "IS5"	group "IS5"
A2HRMARDI	CP019911	2260246	2260929	family "IS5"	group "IS5"
A2HRMARDI	CP019911	2673999	2674682	family "IS5"	group "IS5"
A2HRMARDI	CP019911	2698406	2699089	family "IS5"	group "IS5"
A2HRMARDI	CP019911	2948528	2949211	family "IS5"	group "IS5"
A2HRMARDI	CP019911	3434293	3434976	family "IS5"	group "IS5"
A2HRMARDI	CP019911	3444693	3445376	family "IS5"	group "IS5"
A2HRMARDI	CP019911	822878	823560	family "IS5"	group "IS5"
A2HRMARDI	CP019911	1495444	1496126	family "IS5"	group "IS5"
A2HRMARDI	CP019911	2045282	2045964	family "IS5"	group "IS5"
A2HRMARDI	CP019911	3286156	3286838	family "IS5"	group "IS5"
A2HRMARDI	CP019911	1251003	1251684	family "IS5"	group "IS5"
A2HRMARDI	CP019911	1418481	1419162	family "IS5"	group "IS5"
A2HRMARDI	CP019911	2320900	2321582	family "IS5"	group "IS5"
A2HRMARDI	CP019911	1923070	1923702	family "IS5"	group "IS5"
A2HRMARDI	CP019911	3558575	3559109	family "IS5"	group "IS5"
A2HRMARDI	CP019911	830605	832060	family "IS630"	group ""
A2HRMARDI	CP019911	1925073	1926528	family "IS630"	group ""
A2HRMARDI	CP019911	829288	830173	family "IS630"	group ""
A2HRMARDI	CP019911	916689	917574	family "IS630"	group ""
A2HRMARDI	CP019911	1145187	1146072	family "IS630"	group ""
A2HRMARDI	CP019911	1252639	1253524	family "IS630"	group ""
A2HRMARDI	CP019911	1510253	1511138	family "IS630"	group ""
A2HRMARDI	CP019911	1530609	1531494	family "IS630"	group ""
A2HRMARDI	CP019911	1574475	1575360	family "IS630"	group ""
A2HRMARDI	CP019911	1600814	1601699	family "IS630"	group ""
A2HRMARDI	CP019911	1602055	1602940	family "IS630"	group ""
A2HRMARDI	CP019911	1678915	1679800	family "IS630"	group ""
A2HRMARDI	CP019911	3120786	3121671	family "IS630"	group ""
A2HRMARDI	CP019911	3199199	3200084	family "IS630"	group ""
A2HRMARDI	CP019911	3288048	3288933	family "IS630"	group ""
A2HRMARDI	CP019911	3297545	3298430	family "IS630"	group ""
A2HRMARDI	CP019911	3533779	3534664	family "IS630"	group ""
A2HRMARDI	CP019911	1557845	1558730	family "IS630"	group ""
A2HRMARDI	CP019911	1625175	1626060	family "IS630"	group ""
A2HRMARDI	CP019911	3017286	3018171	family "IS630"	group ""
A2HRMARDI	CP019911	2205190	2205861	family "IS630"	group ""
A2HRMARDI	CP019911	1623926	1624564	family "IS630"	group ""
A2HRMARDI	CP019911	1655710	1656330	family "IS630"	group ""
A2HRMARDI	CP019911	612769	613338	family "IS630"	group ""
A2HRMARDI	CP019911	3556510	3557079	family "IS630"	group ""
A2HRMARDI	CP019911	1494855	1495359	family "IS630"	group ""
A2HRMARDI	CP019911	3150143	3152198	family "IS256"	group ""
A2HRMARDI	CP019911	3597201	3599256	family "IS256"	group ""
A2HRMARDI	CP019911	2043218	2044559	family "IS256"	group ""
A2HRMARDI	CP019911	2289704	2291045	family "IS256"	group ""
A2HRMARDI	CP019911	2518446	2519787	family "IS256"	group ""
A2HRMARDI	CP019911	1054319	1055660	family "IS256"	group ""
A2HRMARDI	CP019911	2687640	2688981	family "IS256"	group ""
A2HRMARDI	CP019911	3289501	3290842	family "IS256"	group ""

A2HRMARDI	CP019911	137233	138574	family "IS256"	group ""
A2HRMARDI	CP019911	1021037	1022378	family "IS256"	group ""
A2HRMARDI	CP019911	1089852	1091193	family "IS256"	group ""
A2HRMARDI	CP019911	1295057	1296398	family "IS256"	group ""
A2HRMARDI	CP019911	1335304	1336645	family "IS256"	group ""
A2HRMARDI	CP019911	1526117	1527458	family "IS256"	group ""
A2HRMARDI	CP019911	2286343	2287684	family "IS256"	group ""
A2HRMARDI	CP019911	2373233	2374574	family "IS256"	group ""
A2HRMARDI	CP019911	3363688	3365029	family "IS256"	group ""
A2HRMARDI	CP019911	3429119	3430460	family "IS256"	group ""
A2HRMARDI	CP019911	609141	610482	family "IS256"	group ""
A2HRMARDI	CP019911	756212	757553	family "IS256"	group ""
A2HRMARDI	CP019911	879421	880762	family "IS256"	group ""
A2HRMARDI	CP019911	1087889	1089230	family "IS256"	group ""
A2HRMARDI	CP019911	1307093	1308434	family "IS256"	group ""
A2HRMARDI	CP019911	1456472	1457813	family "IS256"	group ""
A2HRMARDI	CP019911	1622570	1623911	family "IS256"	group ""
A2HRMARDI	CP019911	1873829	1875170	family "IS256"	group ""
A2HRMARDI	CP019911	1875379	1876720	family "IS256"	group ""
A2HRMARDI	CP019911	2181745	2183086	family "IS256"	group ""
A2HRMARDI	CP019911	2216485	2217826	family "IS256"	group ""
A2HRMARDI	CP019911	2944563	2945904	family "IS256"	group ""
A2HRMARDI	CP019911	1726300	1727641	family "IS256"	group ""
A2HRMARDI	CP019911	1540204	1541545	family "IS256"	group ""
A2HRMARDI	CP019911	1923717	1925058	family "IS256"	group ""
A2HRMARDI	CP019911	3250046	3251387	family "IS256"	group ""
A2HRMARDI	CP019911	3327589	3328930	family "IS256"	group ""
A2HRMARDI	CP019911	1603262	1604603	family "IS256"	group ""
A2HRMARDI	CP019911	2689479	2690820	family "IS256"	group ""
A2HRMARDI	CP019911	3557219	3558560	family "IS256"	group ""
A2HRMARDI	CP019911	1698446	1699787	family "IS256"	group ""
A2HRMARDI	CP019911	3152198	3153386	family "IS256"	group ""
A2HRMARDI	CP019911	3362715	3363673	family "IS256"	group ""
A2HRMARDI	CP019911	614007	614841	family "IS256"	group ""
A2HRMARDI	CP019911	2116071	2117444	family "None"	group "None"
A2HRMARDI	CP019911	2642910	2643556	family "None"	group "None"
A2HRMARDI	CP019911	3112409	3113654	family "IS5"	group "IS1031"
PSI07	IS_523fb345.2	1549247	1550480	family "IS3"	group "IS407"
PSI07	IS_523fb345.2	3512111	3513344	family "IS3"	group "IS407"
PSI07	IS_523fb345.2	1655994	1657227	family "IS3"	group "IS407"
PSI07	IS_523fb345.2	823259	824025	family "IS3"	group "IS407"
PSI07	IS_523fb345.2	1558138	1559473	family "IS3"	group "IS2"
PSI07	IS_523fb345.2	2611702	2613037	family "IS3"	group "IS2"
PSI07	IS_523fb345.2	3108940	3110275	family "IS3"	group "IS2"
PSI07	IS_523fb345.2	1653289	1654624	family "IS3"	group "IS2"
PSI07	IS_523fb345.2	520466	521112	family "None"	group "None"
PSI07	IS_523fb345.2	1517840	1518725	family "IS5"	group "-"
PSI07	IS_523fb345.2	1639780	1641017	family "IS5"	group "IS1031"
PSI07	IS_523fb345.2	2856636	2857926	family "IS3"	group "IS407"
PSI07	IS_523fb345.2	1512826	1514114	family "IS3"	group "IS407"
PSI07	IS_523fb345.2	2614088	2615325	family "IS3"	group "IS407"
PSI07	IS_523fb345.2	3103950	3105189	family "IS3"	group "IS407"
PSI07	IS_523fb345.2	2643518	2644755	family "IS3"	group "IS407"
PSI07	IS_523fb345.2	298701	299289	family "IS3"	group "IS407"
PSI07	IS_523fb345.2	2926770	2927598	family "IS1595"	group "IS1016"
KACC10722	IS_5b99f844	495963	496609	family "None"	group "None"
KACC10722	IS_5b99f844	3521833	3523206	family "None"	group "None"

Table S4a. Characterization of Insertion sequences elements found in the megaplasmid of *Ralstonia* spp. with ISsaga

Strain	ORF Name	% of Similarity Aas	% of AAs Similarity	Identified IS Family	ORF Left End	ORF Right End	ORF Size (bp)
GMI1000	AL646053_00431	100% ISRso6_aa1	100% AL646053_00919	IS21	562934	563977	1.044
GMI1000	AL646053_00432	100% ISRso6_aa2	100% AL646053_00920	IS21	563974	564819	846
GMI1000	AL646053_00919	100% ISRso6_aa1	100% AL646053_00431	IS21	1197017	1198060	1.044
GMI1000	AL646053_00920	100% ISRso6_aa2	100% AL646053_00432	IS21	1198057	1198902	846
GMI1000	AL646053_00459	100% ISRso7_aa1	No hit	IS256	598845	600095	1.251
GMI1000	AL646053_00523	100% ISRso8_aa1	No hit	IS3	687804	688097	294
GMI1000	AL646053_00524	100% ISRso8_aa2	42.08% AL646053_01303	IS3	688094	688984	891
GMI1000	AL646053_00440	100% ISRso10_aa2	93.82% AL646053_00543	IS3 ssgr IS2	578376	577528	849
GMI1000	AL646053_00441	100% ISRso10_aa1	96.12% AL646053_00542	IS3 ssgr IS2	578762	578373	390
GMI1000	AL646053_00542	97.54% ISRso10_aa1	96.12% AL646053_00441	IS3 ssgr IS2	707941	70833	390
GMI1000	AL646053_00543	93.82% ISRso10_aa2	93.82% AL646053_00440	IS3 ssgr IS2	708444	709175	732
GMI1000	AL646053_00484	100% ISRso12_aa1	100% AL646053_01271	IS3 ssgr IS407	625175	625441	267
GMI1000	AL646053_00485	100% ISRso12_aa2	100% AL646053_01303	IS3 ssgr IS407	625591	626295	705
GMI1000	AL646053_00533	100% ISRso16_aa1	87.5% AL646053_01271	IS3 ssgr IS407	699424	69969	267
GMI1000	AL646053_00534	100% ISRso16_aa2	92.41% AL646053_01587	IS3 ssgr IS407	699774	700565	792
GMI1000	AL646053_01186	100% ISRso14_aa1	74.11% AL646053_01271	IS3 ssgr IS407	1547531	1547794	264
GMI1000	AL646053_01187	100% ISRso14_aa2	67.64% AL646053_01587	IS3 ssgr IS407	1548100	1548648	549
GMI1000	AL646053_01271	100% ISRso12_aa1	100% AL646053_00484	IS3 ssgr IS407	1648707	1648973	267
GMI1000	AL646053_01275	100% ISRso12_aa2	100% AL646053_01303	IS3 ssgr IS407	1652963	1653667	705
GMI1000	AL646053_01303	100% ISRso12_aa2	100% AL646053_01275	IS3 ssgr IS407	1683153	1683857	705
GMI1000	AL646053_01584	92.06% ISRso16_aa2	100% AL646053_01587	IS3 ssgr IS407	2047119	2047631	513
GMI1000	AL646053_01587	92.41% ISRso16_aa2	92.41% AL646053_00534	IS3 ssgr IS407	2049261	2048587	675
GMI1000	AL646053_01548	72.26% ISRme10_aa1	No hit	IS30	1992080	1992463	384
GMI1000	AL646053_00446	100% ISRso13_aa1	100% AL646053_01272	IS4 ssgr IS4	581898	583232	1.335
GMI1000	AL646053_01118	100% ISRso13_aa1	100% AL646053_01272	IS4 ssgr IS4	1450099	1448765	1.335
GMI1000	AL646053_01272	100% ISRso13_aa1	100% AL646053_01118	IS4 ssgr IS4	1650320	1648986	1.335
GMI1000	AL646053_00111	99.27% ISRso1_aa1	99.63% AL646053_01302	IS5	148179	147355	825
GMI1000	AL646053_01274	99.63% ISRso1_aa1	100% AL646053_01302	IS5	1652767	1651943	825
GMI1000	AL646053_01302	99.63% ISRso1_aa1	100% AL646053_01274	IS5	1682957	1682133	825
GMI1000	AL646053_00040	100% IS1421_aa1	100% AL646053_00961	IS5 ssgr IS427	45892	46296	405
GMI1000	AL646053_00083	100% IS1421_aa1	100% AL646053_00961	IS5 ssgr IS427	102236	101832	405
GMI1000	AL646053_00282	100% IS1421_aa1	100% AL646053_00961	IS5 ssgr IS427	396332	395928	405
GMI1000	AL646053_00961	100% IS1421_aa1	100% AL646053_00282	IS5 ssgr IS427	1245160	1245564	405
GMI1000	AL646053_00135	100% ISRso9_aa1	100% AL646053_00560	IS5 ssgr IS5	179165	180487	1.323
GMI1000	AL646053_00192	94.51% IS1021_aa1	100% AL646053_01149	IS5 ssgr IS5	255636	25465	987
GMI1000	AL646053_00560	100% ISRso9_aa1	100% AL646053_00135	IS5 ssgr IS5	721452	72013	1.323
GMI1000	AL646053_01105	97.50% IS1405_aa1	70.26% AL646053_01149	IS5 ssgr IS5	1431558	1430593	966
GMI1000	AL646053_01149	94.51% IS1021_aa1	100% AL646053_00192	IS5 ssgr IS5	1487550	1486564	987
GMI1000	AL646053_00202	100% ISRso5_aa1	100% AL646053_01632	IS630	269781	270872	1.092
GMI1000	AL646053_00430	100% ISRso5_aa1	100% AL646053_01632	IS630	562811	562296	516

GMI1000	AL646053_00433	100% ISRso5_aa1	100% AL646053_01632	IS630	565455	564871	585
GMI1000	AL646053_01121	100% ISRso5_aa1	100% AL646053_01632	IS630	1452503	1451412	1.092
GMI1000	AL646053_01632	100% ISRso5_aa1	100% AL646053_01121	IS630	2087388	2088479	1.092
GMI1000	AL646053_00537	79.46% ISPpu19_aa3	No hit	IS66	70424	703791	450
GMI1000	AL646053_00110	100% ISRso17_aa1	100% AL646053_01329	IS701	145895	147256	1.362
GMI1000	AL646053_00136	100% ISRso17_aa1	100% AL646053_01329	IS701	180484	181821	1.338
GMI1000	AL646053_00456	100% ISRso17_aa1	100% AL646053_01329	IS701	591258	589927	1.332
GMI1000	AL646053_01104	100% ISRso17_aa1	100% AL646053_01329	IS701	1429007	1430368	1.362
GMI1000	AL646053_01273	100% ISRso17_aa1	100% AL646053_01329	IS701	1651891	1650530	1.362
GMI1000	AL646053_01301	100% ISRso17_aa1	100% AL646053_01329	IS701	1682081	1680720	1.362
GMI1000	AL646053_01329	100% ISRso17_aa1	100% AL646053_01301	IS701	1712271	1710910	1.362
GMI1000	AL646053_00082	46.07% ISMno24_aa2	40.67% AL646053_00871	IS91	9875	99838	1.089
GMI1000	AL646053_00871	43.95% ISWz1_aa1	43.44% AL646053_01634	IS91	1138814	1140514	1.701
GMI1000	AL646053_01510	100% ISRso15_aa1	No hit	ISL3	1949092	1947872	1.221
GMI1000	AL646053_00886	60.93% ISAba32_aa1	No hit	ISNCY ssgr IS1202	1157633	1157418	216
Po82	RSPO_m01428	62.66% ISPath1_aa1	No hit	IS110 ssgr IS1111	1677899	1677498	402
Po82	RSPO_m00172	53.76% ISHpa1_aa1	No hit	IS1595 ssgr IS1016	188499	189137	639
Po82	RSPO_m00065	99.23% ISRso19_aa2	99.23% RSPO_m01389	IS21	70642	69854	789
Po82	RSPO_m00066	98.22% ISRso19_aa1	100% RSPO_m01388	IS21	71664	70639	1.026
Po82	RSPO_m00405	98.22% ISRso19_aa1	100% RSPO_m01388	IS21	463516	464541	1.026
Po82	RSPO_m00406	99.23% ISRso19_aa2	100% RSPO_m01389	IS21	464538	465326	789
Po82	RSPO_m00549	99.23% ISRso19_aa2	100% RSPO_m01389	IS21	624273	623485	789
Po82	RSPO_m00550	98.22% ISRso19_aa1	100% RSPO_m01388	IS21	625295	62427	1.026
Po82	RSPO_m00930	99.23% ISRso19_aa2	100% RSPO_m01389	IS21	1057188	1056400	789
Po82	RSPO_m00931	98.22% ISRso19_aa1	100% RSPO_m01388	IS21	1058210	1057185	1.026
Po82	RSPO_m01388	98.22% ISRso19_aa1	100% RSPO_m00931	IS21	1634455	1635480	1.026
Po82	RSPO_m01389	99.23% ISRso19_aa2	100% RSPO_m00930	IS21	1635477	1636265	789
Po82	RSPO_m00116	94.57% ISRso10_aa2	100% RSPO_m00780	IS3 ssgr IS2	12508	124193	888
Po82	RSPO_m00117	100% ISRso10_aa1	100% RSPO_m00779	IS3 ssgr IS2	125427	125038	390
Po82	RSPO_m00567	94.57% ISRso10_aa2	100% RSPO_m00780	IS3 ssgr IS2	647108	646221	888
Po82	RSPO_m00568	100% ISRso10_aa1	100% RSPO_m00779	IS3 ssgr IS2	647431	647066	366
Po82	RSPO_m00779	100% ISRso10_aa1	100% RSPO_m00117	IS3 ssgr IS2	877325	877714	390
Po82	RSPO_m00780	94.57% ISRso10_aa2	100% RSPO_m00567	IS3 ssgr IS2	877672	878559	888
Po82	RSPO_m00416	90.13% ISRso16_aa2	No hit	IS3 ssgr IS407	472666	473124	459
Po82	RSPO_m00412	82.14% ISBcen26_aa1	No hit	IS481	469914	469714	201
Po82	RSPO_m00445	98.66% ISRso1_aa1	No hit	IS5	515634	515861	228
Po82	RSPO_m01383	94.84% IS1421_aa1	No hit	IS5 ssgr IS427	1631578	1630928	651
Po82	RSPO_m00295	91.04% ISRso9_aa1	No hit	IS5 ssgr IS5	333903	334541	639
Po82	RSPO_m00384	73.21% ISPsp3_aa1	No hit	IS5 ssgr IS5	448838	448614	225
Po82	RSPO_m00928	54.65% ISAzo11_aa1	No hit	IS5 ssgr IS5	1055205	1055855	651
Po82	RSPO_m00411	86.63% ISRso5_aa1	No hit	IS630	469289	468504	786
Po82	RSPO_m00151	46.59% ISMno24_aa2	43.77% RSPO_m01236	IS91	16063	161703	1.074

Po82	RSPO_m01223	42.93% ISWz1_aa1	50.50% RSPO_m01236	IS91	1409001	1410701	1.701
Po82	RSPO_m01236	41.23% ISShvi3_aa1	50.50% RSPO_m01223	IS91	1432055	1430862	1.194
Po82	RSPO_m00677	0% newcandidate	not_found	New_Family	766594	766749	156
Po82	RSPO_m01231	67.37% ISPa42_aa1	No hit	Tn3	1425541	1427355	1.815
UY031	RSUY_33080	96.05% ISRso7_aa1	No hit	IS256	115125	11612	996
UY031	RSUY_36960	60% ISRme12_aa1	No hit	IS3 ssgr IS150	618015	617395	621
UY031	RSUY_35010	100% ISRso20_aa2	100% RSUY_37850	IS3 ssgr IS3	361692	362534	843
UY031	RSUY_37850	100% ISRso20_aa2	100% RSUY_35010	IS3 ssgr IS3	730907	730065	843
UY031	RSUY_37860	100% ISRso20_aa1	No hit	IS3 ssgr IS3	731248	730952	297
UY031	RSUY_46300	95.87% ISRso20_aa2	95.87% RSUY_37850	IS3 ssgr IS3	1855456	1855061	396
UY031	RSUY_36170	88.37% ISAzo23_aa1	100% RSUY_45980	IS5 ssgr IS1031	522421	521615	807
UY031	RSUY_42800	88.37% ISAzo23_aa1	100% RSUY_45980	IS5 ssgr IS1031	1394620	1395426	807
UY031	RSUY_45980	88.37% ISAzo23_aa1	100% RSUY_42800	IS5 ssgr IS1031	1819925	1819119	807
UY031	RSUY_47360	85.87% IS1421_aa3	No hit	IS5 ssgr IS427	1956587	1957174	588
UY031	RSUY_33040	100% IS1021_aa1	100% RSUY_47200	IS5 ssgr IS5	108733	109719	987
UY031	RSUY_33130	100% IS1021_aa1	100% RSUY_47200	IS5 ssgr IS5	123537	124523	987
UY031	RSUY_33340	100% IS1021_aa1	100% RSUY_47200	IS5 ssgr IS5	154033	155019	987
UY031	RSUY_33470	100% IS1021_aa1	100% RSUY_47200	IS5 ssgr IS5	178644	177658	987
UY031	RSUY_33640	100% IS1021_aa1	100% RSUY_47200	IS5 ssgr IS5	19545	196436	987
UY031	RSUY_33780	100% IS1021_aa1	100% RSUY_47200	IS5 ssgr IS5	219659	218673	987
UY031	RSUY_34140	100% IS1021_aa1	100% RSUY_47200	IS5 ssgr IS5	26619	265204	987
UY031	RSUY_34960	100% IS1021_aa1	100% RSUY_47200	IS5 ssgr IS5	353028	354014	987
UY031	RSUY_35000	100% IS1021_aa1	100% RSUY_47200	IS5 ssgr IS5	361365	360379	987
UY031	RSUY_35150	100% IS1021_aa1	100% RSUY_47200	IS5 ssgr IS5	383536	384522	987
UY031	RSUY_35160	100% IS1021_aa1	100% RSUY_47200	IS5 ssgr IS5	385923	384937	987
UY031	RSUY_35520	100% IS1021_aa1	100% RSUY_47200	IS5 ssgr IS5	435202	434216	987
UY031	RSUY_36180	100% IS1021_aa1	100% RSUY_47200	IS5 ssgr IS5	522545	523531	987
UY031	RSUY_36190	100% IS1021_aa1	100% RSUY_47200	IS5 ssgr IS5	525284	524298	987
UY031	RSUY_36200	100% IS1021_aa1	100% RSUY_47200	IS5 ssgr IS5	526773	525787	987
UY031	RSUY_36270	100% IS1021_aa1	100% RSUY_47200	IS5 ssgr IS5	533439	534425	987
UY031	RSUY_37190	100% IS1021_aa1	100% RSUY_47200	IS5 ssgr IS5	644762	645748	987
UY031	RSUY_37200	100% IS1021_aa1	100% RSUY_47200	IS5 ssgr IS5	646299	647285	987
UY031	RSUY_37250	100% IS1021_aa1	100% RSUY_47200	IS5 ssgr IS5	658073	659059	987
UY031	RSUY_37670	100% IS1021_aa1	100% RSUY_47200	IS5 ssgr IS5	704703	705689	987
UY031	RSUY_37910	100% IS1021_aa1	100% RSUY_47200	IS5 ssgr IS5	736806	737792	987
UY031	RSUY_38030	100% IS1021_aa1	100% RSUY_47200	IS5 ssgr IS5	748639	747653	987
UY031	RSUY_38160	100% IS1021_aa1	100% RSUY_47200	IS5 ssgr IS5	759963	760949	987
UY031	RSUY_39310	100% IS1021_aa1	100% RSUY_47200	IS5 ssgr IS5	929515	928529	987
UY031	RSUY_39340	100% IS1021_aa1	100% RSUY_47200	IS5 ssgr IS5	931259	932245	987
UY031	RSUY_39520	100% IS1021_aa1	100% RSUY_47200	IS5 ssgr IS5	958004	95899	987
UY031	RSUY_40680	100% IS1021_aa1	100% RSUY_47200	IS5 ssgr IS5	1101538	1100552	987
UY031	RSUY_41140	100% IS1021_aa1	100% RSUY_47200	IS5 ssgr IS5	1154714	1153728	987

UY031	RSUY_42210	100% IS1021_aa1	100% RSUY_47200	IS5 ssgr IS5	1316979	1317965	987
UY031	RSUY_42580	100% IS1021_aa1	100% RSUY_47200	IS5 ssgr IS5	1362845	1361859	987
UY031	RSUY_42740	55.85% ISAzo41_aa1	No hit	IS5 ssgr IS5	1388195	1387830	366
UY031	RSUY_42750	100% IS1021_aa1	100% RSUY_47200	IS5 ssgr IS5	1390051	1389065	987
UY031	RSUY_43070	100% IS1021_aa1	100% RSUY_47200	IS5 ssgr IS5	1433213	1434199	987
UY031	RSUY_43970	100% IS1021_aa1	100% RSUY_47200	IS5 ssgr IS5	1554558	1555544	987
UY031	RSUY_44380	100% IS1021_aa1	100% RSUY_47200	IS5 ssgr IS5	1614018	1615004	987
UY031	RSUY_44650	100% IS1021_aa1	100% RSUY_47200	IS5 ssgr IS5	1650256	1649270	987
UY031	RSUY_44670	100% IS1021_aa1	100% RSUY_47200	IS5 ssgr IS5	1652148	1653134	987
UY031	RSUY_44730	100% IS1021_aa1	100% RSUY_47200	IS5 ssgr IS5	1658899	1659885	987
UY031	RSUY_44840	100% IS1021_aa1	100% RSUY_47200	IS5 ssgr IS5	1671006	1671992	987
UY031	RSUY_45210	100% IS1021_aa1	100% RSUY_47200	IS5 ssgr IS5	1710248	1711234	987
UY031	RSUY_45550	100% IS1021_aa1	100% RSUY_47200	IS5 ssgr IS5	1756106	1757092	987
UY031	RSUY_45990	100% IS1021_aa1	100% RSUY_47200	IS5 ssgr IS5	1821093	1820107	987
UY031	RSUY_46370	100% IS1021_aa1	100% RSUY_47200	IS5 ssgr IS5	1864367	1865353	987
UY031	RSUY_46490	85.32% ISBmu20_aa1	66.66% RSUY_44650	IS5 ssgr IS5	1881489	1881142	348
UY031	RSUY_46500	92.85% ISBmu20_aa1	76.81% RSUY_47200	IS5 ssgr IS5	1881705	1881502	204
UY031	RSUY_46520	52.05% ISRso18_aa1	83.87% RSUY_44650	IS5 ssgr IS5	1882016	1881876	141
UY031	RSUY_46720	100% IS1021_aa1	100% RSUY_47200	IS5 ssgr IS5	1896117	1895131	987
UY031	RSUY_46910	100% IS1021_aa1	100% RSUY_47200	IS5 ssgr IS5	1910123	1911109	987
UY031	RSUY_46920	100% IS1021_aa1	100% RSUY_47200	IS5 ssgr IS5	1911336	1912322	987
UY031	RSUY_47110	100% IS1021_aa1	100% RSUY_47200	IS5 ssgr IS5	1931124	1932110	987
UY031	RSUY_47200	100% IS1021_aa1	100% RSUY_47110	IS5 ssgr IS5	1940645	1941631	987
UY031	RSUY_41150	42.93% ISWz1_aa1	39.93% RSUY_47770	IS91	1155315	1157015	1.701
UY031	RSUY_41240	68.37% ISKpn21_aa1	No hit	ISNCY ssgr IS1202	1175026	1174160	867
UW163	CP012940_01302	54.35% ISHpa1_aa1	100% CP012940_01418	IS1595 ssgr IS1016	1662463	1663242	780
UW163	CP012940_01418	54.35% ISHpa1_aa1	100% CP012940_01302	IS1595 ssgr IS1016	1813987	1813208	780
UW163	CP012940_00001	97.34% ISRso19_aa1	100% CP012940_01212	IS21	18	584	567
UW163	CP012940_00002	99.23% ISRso19_aa2	100% CP012940_01211	IS21	581	1369	789
UW163	CP012940_00330	99.23% ISRso19_aa2	100% CP012940_01211	IS21	384831	384043	789
UW163	CP012940_00331	98.18% ISRso19_aa1	100% CP012940_01212	IS21	385823	384828	996
UW163	CP012940_00433	98.18% ISRso19_aa1	100% CP012940_01212	IS21	509722	510717	996
UW163	CP012940_00434	99.23% ISRso19_aa2	100% CP012940_01211	IS21	510714	511502	789
UW163	CP012940_00890	98.18% ISRso19_aa1	100% CP012940_01212	IS21	1131031	1132026	996
UW163	CP012940_00891	99.23% ISRso19_aa2	100% CP012940_01211	IS21	1132023	1132811	789
UW163	CP012940_01211	99.23% ISRso19_aa2	100% CP012940_00891	IS21	1537835	1537047	789
UW163	CP012940_01212	98.18% ISRso19_aa1	100% CP012940_00890	IS21	1538827	1537832	996
UW163	CP012940_00454	96% ISRso10_aa2	100% CP012940_01462	IS3 ssgr IS2	533034	532432	603
UW163	CP012940_00455	100% ISRso10_aa1	100% CP012940_01463	IS3 ssgr IS2	533666	533277	390
UW163	CP012940_01025	96% ISRso10_aa2	100% CP012940_01462	IS3 ssgr IS2	1310383	1309781	603
UW163	CP012940_01026	100% ISRso10_aa1	100% CP012940_01463	IS3 ssgr IS2	1311015	1310626	390
UW163	CP012940_01462	96% ISRso10_aa2	100% CP012940_01025	IS3 ssgr IS2	1871396	1870794	603

UW163	CP012940_01463	100% ISRso10_aa1	100% CP012940_01026	IS3 ssgr IS2	1872028	1871639	390
UW163	CP012940_01203	90.13% ISRso16_aa2	No hit	IS3 ssgr IS407	1529707	1529249	459
UW163	CP012940_01207	82.14% ISBcen26_aa1	No hit	IS481	1532458	1532658	201
UW163	CP012940_00342	97.53% ISRso1_aa1	No hit	IS5	402679	402927	249
UW163	CP012940_00886	93.67% IS1421_aa1	No hit	IS5 ssgr IS427	1128070	1127474	597
UW163	CP012940_00892	55.29% ISAzo11_aa1	No hit	IS5 ssgr IS5	1133661	1133356	306
UW163	CP012940_01305	89.84% ISRso9_aa1	No hit	IS5 ssgr IS5	1668225	1667821	405
UW163	CP012940_01208	86.63% ISRso5_aa1	No hit	IS630	1533083	1533868	786
UW163	CP012940_00742	42.93% ISWz1_aa1	50.50% CP012940_00755	IS91	905593	907293	1.701
UW163	CP012940_00755	41.23% ISShvi3_aa1	50.50% CP012940_00742	IS91	92864	927447	1.194
UW163	CP012940_01435	46.59% ISMno24_aa2	43.77% CP012940_00755	IS91	1841739	1840666	1.074
UW163	CP012940_00750	67.37% ISPa42_aa1	No hit	Tn3	922126	92394	1.815
UW163	CP012940_00751	85.01% ISPa42_aa1	No hit	Tn3	924253	925137	885
IBSBF1503	RALBFv3_22575	53.76% ISHpa1_aa1	No hit	IS1595 ssgr IS1016	1830536	1829898	639
IBSBF1503	RALBFv3_18595	99.23% ISRso19_aa2	99.61% RALBFv3_22995	IS21	710919	710131	789
IBSBF1503	RALBFv3_18600	97.33% ISRso19_aa1	99.40% RALBFv3_20940	IS21	711932	710916	1.017
IBSBF1503	RALBFv3_20760	94.82% ISRso19_aa2	94.82% RALBFv3_20935	IS21	1360452	1360634	183
IBSBF1503	RALBFv3_20770	98.22% ISRso19_aa1	100% RALBFv3_22990	IS21	1361935	1362951	1.017
IBSBF1503	RALBFv3_20775	96.84% ISRso19_aa2	96.84% RALBFv3_22995	IS21	1362948	1363232	285
IBSBF1503	RALBFv3_20935	99.23% ISRso19_aa2	99.23% RALBFv3_18595	IS21	1409344	1408556	789
IBSBF1503	RALBFv3_20940	97.92% ISRso19_aa1	99.70% RALBFv3_22990	IS21	1410357	1409341	1.017
IBSBF1503	RALBFv3_21355	98.22% ISRso19_aa1	100% RALBFv3_22990	IS21	1517816	1518832	1.017
IBSBF1503	RALBFv3_21360	100% ISRso19_aa2	100% RALBFv3_20935	IS21	1518829	1519275	447
IBSBF1503	RALBFv3_22990	98.22% ISRso19_aa1	100% RALBFv3_21355	IS21	1935236	1936252	1.017
IBSBF1503	RALBFv3_22995	99.61% ISRso19_aa2	99.61% RALBFv3_18595	IS21	1936249	1937037	789
IBSBF1503	RALBFv3_20725	90.58% ISAtu5_aa1	No hit	IS3 ssgr IS407	1350066	1350326	261
IBSBF1503	RALBFv3_20730	76.09% ISDet2_aa2	65.73% RALBFv3_20900	IS3 ssgr IS407	1350365	1351189	825
IBSBF1503	RALBFv3_20900	90.13% ISRso16_aa2	65.73% RALBFv3_20730	IS3 ssgr IS407	1401214	1400756	459
IBSBF1503	RALBFv3_20735	96.71% IS401_aa2	71.79% RALBFv3_16055	IS3 ssgr IS51	1352302	1351388	915
IBSBF1503	RALBFv3_20740	95.32% IS401_aa1	No hit	IS3 ssgr IS51	1352622	1352299	324
IBSBF1503	RALBFv3_16515	98.66% ISRso1_aa1	No hit	IS5	178149	178376	228
IBSBF1503	RALBFv3_16030	77.23% IS1421_aa2	No hit	IS5 ssgr IS427	37954	3831	357
IBSBF1503	RALBFv3_18625	94.54% IS1421_aa1	No hit	IS5 ssgr IS427	714779	715108	330
IBSBF1503	RALBFv3_23540	69.45% IS1421_aa3	No hit	IS5 ssgr IS427	2065828	2065056	773
IBSBF1503	RALBFv3_18590	55.29% ISAzo11_aa1	No hit	IS5 ssgr IS5	709281	709586	306
IBSBF1503	RALBFv3_22020	91.04% ISRso9_aa1	No hit	IS5 ssgr IS5	1684947	1684525	423
IBSBF1503	RALBFv3_20920	83.16% ISRso5_aa1	No hit	IS630	1404168	1405293	1.126
IBSBF1503	RALBFv3_19270	41.23% ISShvi3_aa1	50.50% RALBFv3_19325	IS91	914366	915559	1.194
IBSBF1503	RALBFv3_19325	42.93% ISWz1_aa1	50.50% RALBFv3_19270	IS91	93742	93572	1.701
IBSBF1503	RALBFv3_19280	0% newcandidate	not_found	New_Family	917882	917544	339
IBSBF1503	RALBFv3_19285	66.99% ISPa42_aa1	No hit	Tn3	92088	917869	3.012

RS488	CP021653_00098	96.05% ISRso7_aa1	No hit	IS256	115125	11612	996
RS488	CP021653_00488	60% ISRme12_aa1	No hit	IS3 ssgr IS150	618006	617386	621
RS488	CP021653_00290	100% ISRso20_aa1	100% CP021653_00579	IS3 ssgr IS3	36145	361638	189
RS488	CP021653_00291	100% ISRso20_aa2	100% CP021653_00578	IS3 ssgr IS3	361683	362525	843
RS488	CP021653_00578	100% ISRso20_aa2	100% CP021653_00291	IS3 ssgr IS3	730899	730057	843
RS488	CP021653_00579	100% ISRso20_aa1	100% CP021653_00290	IS3 ssgr IS3	73124	730944	297
RS488	CP021653_01423	95.87% ISRso20_aa2	95.87% CP021653_00578	IS3 ssgr IS3	1855458	1855063	396
RS488	CP021653_00408	88.37% ISAzo23_aa1	100% CP021653_01391	IS5 ssgr IS1031	522412	521606	807
RS488	CP021653_01071	88.37% ISAzo23_aa1	100% CP021653_01391	IS5 ssgr IS1031	1394613	1395419	807
RS488	CP021653_01391	88.37% ISAzo23_aa1	100% CP021653_01071	IS5 ssgr IS1031	1819927	1819121	807
RS488	CP021653_01529	85.87% IS1421_aa3	No hit	IS5 ssgr IS427	1956553	1957176	624
RS488	CP021653_00094	100% IS1021_aa1	100% CP021653_01513	IS5 ssgr IS5	108733	109719	987
RS488	CP021653_00103	100% IS1021_aa1	100% CP021653_01513	IS5 ssgr IS5	123537	124523	987
RS488	CP021653_00124	100% IS1021_aa1	100% CP021653_01513	IS5 ssgr IS5	154033	155019	987
RS488	CP021653_00135	100% IS1021_aa1	100% CP021653_01513	IS5 ssgr IS5	178635	177649	987
RS488	CP021653_00152	100% IS1021_aa1	100% CP021653_01513	IS5 ssgr IS5	195441	196427	987
RS488	CP021653_00166	100% IS1021_aa1	100% CP021653_01513	IS5 ssgr IS5	21965	218664	987
RS488	CP021653_00203	100% IS1021_aa1	100% CP021653_01513	IS5 ssgr IS5	266181	265195	987
RS488	CP021653_00285	100% IS1021_aa1	100% CP021653_01513	IS5 ssgr IS5	353019	354005	987
RS488	CP021653_00289	100% IS1021_aa1	100% CP021653_01513	IS5 ssgr IS5	361356	36037	987
RS488	CP021653_00305	100% IS1021_aa1	100% CP021653_01513	IS5 ssgr IS5	383527	384513	987
RS488	CP021653_00306	100% IS1021_aa1	100% CP021653_01513	IS5 ssgr IS5	385914	384928	987
RS488	CP021653_00342	100% IS1021_aa1	100% CP021653_01513	IS5 ssgr IS5	435193	434207	987
RS488	CP021653_00409	100% IS1021_aa1	100% CP021653_01513	IS5 ssgr IS5	522536	523522	987
RS488	CP021653_00410	100% IS1021_aa1	100% CP021653_01513	IS5 ssgr IS5	525275	524289	987
RS488	CP021653_00412	100% IS1021_aa1	100% CP021653_01513	IS5 ssgr IS5	526764	525778	987
RS488	CP021653_00419	100% IS1021_aa1	100% CP021653_01513	IS5 ssgr IS5	53343	534416	987
RS488	CP021653_00512	100% IS1021_aa1	100% CP021653_01513	IS5 ssgr IS5	644753	645739	987
RS488	CP021653_00513	100% IS1021_aa1	100% CP021653_01513	IS5 ssgr IS5	64629	647276	987
RS488	CP021653_00518	100% IS1021_aa1	100% CP021653_01513	IS5 ssgr IS5	658064	65905	987
RS488	CP021653_00560	100% IS1021_aa1	100% CP021653_01513	IS5 ssgr IS5	704695	705681	987
RS488	CP021653_00584	100% IS1021_aa1	100% CP021653_01513	IS5 ssgr IS5	736798	737784	987
RS488	CP021653_00595	100% IS1021_aa1	100% CP021653_01513	IS5 ssgr IS5	748632	747646	987
RS488	CP021653_00608	100% IS1021_aa1	100% CP021653_01513	IS5 ssgr IS5	759956	760942	987
RS488	CP021653_00722	100% IS1021_aa1	100% CP021653_01513	IS5 ssgr IS5	929508	928522	987
RS488	CP021653_00725	100% IS1021_aa1	100% CP021653_01513	IS5 ssgr IS5	931252	932238	987
RS488	CP021653_00743	100% IS1021_aa1	100% CP021653_01513	IS5 ssgr IS5	957997	958983	987
RS488	CP021653_00859	100% IS1021_aa1	100% CP021653_01513	IS5 ssgr IS5	1101531	1100545	987
RS488	CP021653_00905	100% IS1021_aa1	100% CP021653_01513	IS5 ssgr IS5	1154707	1153721	987
RS488	CP021653_01012	100% IS1021_aa1	100% CP021653_01513	IS5 ssgr IS5	1316972	1317958	987
RS488	CP021653_01049	100% IS1021_aa1	100% CP021653_01513	IS5 ssgr IS5	1362838	1361852	987
RS488	CP021653_01065	55.85% ISAzo41_aa1	No hit	IS5 ssgr IS5	1388188	1387823	366
RS488	CP021653_01066	100% IS1021_aa1	100% CP021653_01513	IS5 ssgr IS5	1390044	1389058	987

RS488	CP021653_01098	100% IS1021_aa1	100% CP021653_01513	IS5 ssgr IS5	1433206	1434192	987
RS488	CP021653_01188	100% IS1021_aa1	100% CP021653_01513	IS5 ssgr IS5	1554560	1555546	987
RS488	CP021653_01230	100% IS1021_aa1	100% CP021653_01513	IS5 ssgr IS5	1614020	1615006	987
RS488	CP021653_01257	100% IS1021_aa1	100% CP021653_01513	IS5 ssgr IS5	1650258	1649272	987
RS488	CP021653_01259	100% IS1021_aa1	100% CP021653_01513	IS5 ssgr IS5	1652150	1653136	987
RS488	CP021653_01265	100% IS1021_aa1	100% CP021653_01513	IS5 ssgr IS5	1658901	1659887	987
RS488	CP021653_01276	100% IS1021_aa1	100% CP021653_01513	IS5 ssgr IS5	1671008	1671994	987
RS488	CP021653_01314	100% IS1021_aa1	100% CP021653_01513	IS5 ssgr IS5	1710250	1711236	987
RS488	CP021653_01348	100% IS1021_aa1	100% CP021653_01513	IS5 ssgr IS5	1756108	1757094	987
RS488	CP021653_01392	100% IS1021_aa1	100% CP021653_01513	IS5 ssgr IS5	1821095	1820109	987
RS488	CP021653_01430	100% IS1021_aa1	100% CP021653_01513	IS5 ssgr IS5	1864369	1865355	987
RS488	CP021653_01442	85.32% ISBmu20_aa1	66.66% CP021653_01257	IS5 ssgr IS5	1881491	1881144	348
RS488	CP021653_01443	92.85% ISBmu20_aa1	76.81% CP021653_01513	IS5 ssgr IS5	1881707	1881504	204
RS488	CP021653_01445	52.05% ISRso18_aa1	83.87% CP021653_01257	IS5 ssgr IS5	1882018	1881878	141
RS488	CP021653_01465	100% IS1021_aa1	100% CP021653_01513	IS5 ssgr IS5	1896119	1895133	987
RS488	CP021653_01484	100% IS1021_aa1	100% CP021653_01513	IS5 ssgr IS5	1910125	1911111	987
RS488	CP021653_01485	100% IS1021_aa1	100% CP021653_01513	IS5 ssgr IS5	1911338	1912324	987
RS488	CP021653_01504	100% IS1021_aa1	100% CP021653_01513	IS5 ssgr IS5	1931126	1932112	987
RS488	CP021653_01513	100% IS1021_aa1	100% CP021653_01504	IS5 ssgr IS5	1940647	1941633	987
RS488	CP021653_00906	42.93% ISWz1_aa1	39.93% CP021653_01570	IS91	1155308	1157008	1.701
RS488	CP021653_00915	68.37% ISKpn21_aa1	No hit	ISNCY ssgr IS1202	1175019	1174153	867
UW551	B7R79_18235	57.97% ISPath1_aa1	No hit	IS110 ssgr IS1111	470359	470751	393
UW551	B7R79_21885	72.41% ISBdo1_aa1	No hit	IS1182	1406430	1406631	202
UW551	B7R79_19975	66.10% IS1326_aa1	No hit	IS21	912619	91226	360
UW551	B7R79_17445	96.05% ISRso7_aa1	No hit	IS256	267966	269218	1.253
UW551	B7R79_16480	95.87% ISRso20_aa2	No hit	IS3 ssgr IS3	55776	5536	417
UW551	B7R79_18415	100% ISRso20_aa3	95.87% B7R79_16480	IS3 ssgr IS3	514272	515365	1.094
UW551	B7R79_19970	100% ISRso20_aa3	95.87% B7R79_16480	IS3 ssgr IS3	91223	911047	1.184
UW551	B7R79_16570	76% ISRso1_aa1	60.34% B7R79_24170	IS5	75075	74857	219
UW551	B7R79_16320	88.37% ISAzo23_aa1	100% B7R79_24170	IS5 ssgr IS1031	20225	19419	807
UW551	B7R79_19005	88.37% ISAzo23_aa1	100% B7R79_24170	IS5 ssgr IS1031	67525	674444	807
UW551	B7R79_22480	88.37% ISAzo23_aa1	100% B7R79_24170	IS5 ssgr IS1031	1574368	1575174	807
UW551	B7R79_24170	88.37% ISAzo23_aa1	100% B7R79_22480	IS5 ssgr IS1031	1999658	1998852	807
UW551	B7R79_16510	78.14% ISAzba7_aa3	No hit	IS5 ssgr IS427	64419	63614	806
UW551	B7R79_16605	78.14% ISAzba7_aa3	No hit	IS5 ssgr IS427	84205	834	806
UW551	B7R79_16740	85.87% IS1421_aa3	No hit	IS5 ssgr IS427	109969	110502	534
UW551	B7R79_17440	78.14% ISAzba7_aa3	No hit	IS5 ssgr IS427	267629	266824	806
UW551	B7R79_17470	78.14% ISAzba7_aa3	No hit	IS5 ssgr IS427	277757	278562	806
UW551	B7R79_17930	78.14% ISAzba7_aa3	No hit	IS5 ssgr IS427	407481	406676	806
UW551	B7R79_18265	78.14% ISAzba7_aa3	No hit	IS5 ssgr IS427	48063	479825	806
UW551	B7R79_18405	77.48% ISAzba7_aa3	No hit	IS5 ssgr IS427	512878	512073	806
UW551	B7R79_18675	78.14% ISAzba7_aa3	No hit	IS5 ssgr IS427	590511	591316	806

UW551	B7R79_19655	81.31% IS1421_aa2	No hit	IS5 ssgr IS427	829508	830312	805
UW551	B7R79_21630	84.61% IS1421_aa3	No hit	IS5 ssgr IS427	1334434	1333630	805
UW551	B7R79_22460	78.14% ISAzba7_aa3	No hit	IS5 ssgr IS427	1570866	1571671	806
UW551	B7R79_22475	78.14% ISAzba7_aa3	No hit	IS5 ssgr IS427	1574000	1573195	806
UW551	B7R79_23485	84.61% IS1421_aa3	No hit	IS5 ssgr IS427	1833601	1832797	805
UW551	B7R79_16325	100% IS1021_aa1	100% B7R79_24175	IS5 ssgr IS5	21393	20407	987
UW551	B7R79_16515	100% IS1021_aa1	100% B7R79_24175	IS5 ssgr IS5	64666	65652	987
UW551	B7R79_16610	100% IS1021_aa1	100% B7R79_24175	IS5 ssgr IS5	84452	85438	987
UW551	B7R79_16660	100% IS1021_aa1	100% B7R79_24175	IS5 ssgr IS5	93973	94959	987
UW551	B7R79_17425	100% IS1021_aa1	100% B7R79_24175	IS5 ssgr IS5	261574	26256	987
UW551	B7R79_17465	100% IS1021_aa1	100% B7R79_24175	IS5 ssgr IS5	276378	277364	987
UW551	B7R79_17580	100% IS1021_aa1	100% B7R79_24175	IS5 ssgr IS5	306873	307859	987
UW551	B7R79_17625	100% IS1021_aa1	100% B7R79_24175	IS5 ssgr IS5	331476	33049	987
UW551	B7R79_17710	100% IS1021_aa1	100% B7R79_24175	IS5 ssgr IS5	348282	349268	987
UW551	B7R79_17785	100% IS1021_aa1	100% B7R79_24175	IS5 ssgr IS5	372491	371505	987
UW551	B7R79_17975	100% IS1021_aa1	100% B7R79_24175	IS5 ssgr IS5	419022	418036	987
UW551	B7R79_18385	100% IS1021_aa1	100% B7R79_24175	IS5 ssgr IS5	505859	506845	987
UW551	B7R79_18410	100% IS1021_aa1	100% B7R79_24175	IS5 ssgr IS5	514196	51321	987
UW551	B7R79_18485	100% IS1021_aa1	100% B7R79_24175	IS5 ssgr IS5	536366	537352	987
UW551	B7R79_18490	100% IS1021_aa1	100% B7R79_24175	IS5 ssgr IS5	538753	537767	987
UW551	B7R79_18665	100% IS1021_aa1	100% B7R79_24175	IS5 ssgr IS5	588032	587046	987
UW551	B7R79_19010	100% IS1021_aa1	100% B7R79_24175	IS5 ssgr IS5	675374	67636	987
UW551	B7R79_19020	100% IS1021_aa1	100% B7R79_24175	IS5 ssgr IS5	678113	677127	987
UW551	B7R79_19130	100% IS1021_aa1	100% B7R79_24175	IS5 ssgr IS5	707482	706496	987
UW551	B7R79_19175	100% IS1021_aa1	100% B7R79_24175	IS5 ssgr IS5	714148	715134	987
UW551	B7R79_19635	100% IS1021_aa1	100% B7R79_24175	IS5 ssgr IS5	825745	826731	987
UW551	B7R79_19640	100% IS1021_aa1	100% B7R79_24175	IS5 ssgr IS5	827282	828268	987
UW551	B7R79_19680	100% IS1021_aa1	100% B7R79_24175	IS5 ssgr IS5	839056	840042	987
UW551	B7R79_19885	100% IS1021_aa1	100% B7R79_24175	IS5 ssgr IS5	885686	886672	987
UW551	B7R79_20005	100% IS1021_aa1	100% B7R79_24175	IS5 ssgr IS5	917787	918773	987
UW551	B7R79_20060	100% IS1021_aa1	100% B7R79_24175	IS5 ssgr IS5	92962	928634	987
UW551	B7R79_20125	100% IS1021_aa1	100% B7R79_24175	IS5 ssgr IS5	940944	94193	987
UW551	B7R79_20705	100% IS1021_aa1	100% B7R79_24175	IS5 ssgr IS5	1110489	1109503	987
UW551	B7R79_20720	100% IS1021_aa1	100% B7R79_24175	IS5 ssgr IS5	1112233	1113219	987
UW551	B7R79_20815	100% IS1021_aa1	100% B7R79_24175	IS5 ssgr IS5	1138978	1139964	987
UW551	B7R79_21410	100% IS1021_aa1	100% B7R79_24175	IS5 ssgr IS5	1282509	1281523	987
UW551	B7R79_21635	100% IS1021_aa1	100% B7R79_24175	IS5 ssgr IS5	1335682	1334696	987
UW551	B7R79_22185	100% IS1021_aa1	100% B7R79_24175	IS5 ssgr IS5	1497943	1498929	987
UW551	B7R79_22440	56.36% ISAzo41_aa1	No hit	IS5 ssgr IS5	1568050	1567578	473
UW551	B7R79_22450	100% IS1021_aa1	100% B7R79_24175	IS5 ssgr IS5	1569799	1568813	987
UW551	B7R79_22620	100% IS1021_aa1	100% B7R79_24175	IS5 ssgr IS5	1612961	1613947	987
UW551	B7R79_23105	100% IS1021_aa1	100% B7R79_24175	IS5 ssgr IS5	1734302	1735288	987
UW551	B7R79_23325	100% IS1021_aa1	100% B7R79_24175	IS5 ssgr IS5	1793758	1794744	987

UW551	B7R79_23460	100% IS1021_aa1	100% B7R79_24175	IS5 ssgr IS5	1829994	1829008	987
UW551	B7R79_23500	100% IS1021_aa1	100% B7R79_24175	IS5 ssgr IS5	1835789	1836775	987
UW551	B7R79_23520	100% IS1021_aa1	100% B7R79_24175	IS5 ssgr IS5	1838636	1839622	987
UW551	B7R79_23590	100% IS1021_aa1	100% B7R79_24175	IS5 ssgr IS5	1850743	1851729	987
UW551	B7R79_23775	100% IS1021_aa1	100% B7R79_24175	IS5 ssgr IS5	1889984	1890970	987
UW551	B7R79_23955	100% IS1021_aa1	100% B7R79_24175	IS5 ssgr IS5	1935841	1936827	987
UW551	B7R79_24175	100% IS1021_aa1	100% B7R79_23955	IS5 ssgr IS5	2000826	1999840	987
UW551	B7R79_21640	42.93% ISWz1_aa1	39.93% B7R79_16935	IS91	1336283	1337983	1.701
UW551	B7R79_21685	70.07% ISKpn21_aa1	No hit	ISNCY ssgr IS1202	1356398	1355128	1.271
UW551	B7R79_20945	40.57% ISAzs34_aa2	40.51% B7R79_21440	ISNCY ssgr IS892	1168230	1169167	938
RS489	CDC59_17220	57.97% ISPath1_aa1	No hit	IS110 ssgr IS1111	317589	317981	393
RS489	CDC59_20750	89.28% ISBdo1_aa1	No hit	IS1182	1225465	1225654	190
RS489	CDC59_23195	49.55% ISLsp2_aa1	No hit	IS1595 ssgr ISPna2	1856973	1855591	1.383
RS489	CDC59_16070	37.73% ISFsp3_aa2	45.01% CDC59_23165	IS21	33141	31508	1.634
RS489	CDC59_18840	63.85% ISGdi17_aa1	No hit	IS21	731628	731269	360
RS489	CDC59_16430	98.60% ISRso7_aa1	No hit	IS256	115168	116419	1.252
RS489	CDC59_17400	98.90% ISRso20_aa3	99.17% CDC59_18835	IS3 ssgr IS3	361498	362591	1.094
RS489	CDC59_18835	99.23% ISRso20_aa3	99.17% CDC59_17400	IS3 ssgr IS3	731239	730056	1.184
RS489	CDC59_23190	97.93% ISRso20_aa2	95.87% CDC59_18835	IS3 ssgr IS3	1855512	1855096	417
RS489	CDC59_23550	81.25% ISRso1_aa1	60.34% CDC59_23030	IS5	1921770	1921579	192
RS489	CDC59_17980	88.37% ISAzo23_aa1	100% CDC59_23030	IS5 ssgr IS1031	522455	521649	807
RS489	CDC59_21360	88.37% ISAzo23_aa1	100% CDC59_23030	IS5 ssgr IS1031	1394581	1395387	807
RS489	CDC59_23030	88.37% ISAzo23_aa1	100% CDC59_21360	IS5 ssgr IS1031	1819971	1819165	807
RS489	CDC59_16425	85.82% IS1421_aa3	99.62% CDC59_16900	IS5 ssgr IS427	114831	114026	806
RS489	CDC59_16455	85.82% IS1421_aa3	99.62% CDC59_16900	IS5 ssgr IS427	124957	125762	806
RS489	CDC59_16900	86.19% IS1421_aa3	99.62% CDC59_23590	IS5 ssgr IS427	254728	253923	806
RS489	CDC59_17250	85.82% IS1421_aa3	99.62% CDC59_16900	IS5 ssgr IS427	327863	327058	806
RS489	CDC59_17390	85.82% IS1421_aa3	99.62% CDC59_16900	IS5 ssgr IS427	360104	359299	806
RS489	CDC59_17660	85.07% IS1421_aa3	98.88% CDC59_16900	IS5 ssgr IS427	437757	438562	806
RS489	CDC59_18520	92.13% IS1421_aa3	81.34% CDC59_16900	IS5 ssgr IS427	648536	649339	804
RS489	CDC59_20485	85.82% IS1421_aa3	99.62% CDC59_16900	IS5 ssgr IS427	1153447	1152642	806
RS489	CDC59_21335	85.82% IS1421_aa3	99.62% CDC59_16900	IS5 ssgr IS427	1391079	1391884	806
RS489	CDC59_21355	85.82% IS1421_aa3	99.62% CDC59_16900	IS5 ssgr IS427	1394213	1393408	806
RS489	CDC59_22350	85.82% IS1421_aa3	99.62% CDC59_16900	IS5 ssgr IS427	1655111	1654306	806
RS489	CDC59_23220	85.82% IS1421_aa3	99.62% CDC59_16900	IS5 ssgr IS427	1864156	1863351	806
RS489	CDC59_23280	96.05% IS1421_aa3	89.61% CDC59_23590	IS5 ssgr IS427	1879209	1878718	492
RS489	CDC59_23290	85.82% IS1421_aa3	99.62% CDC59_16900	IS5 ssgr IS427	1879794	1880599	806
RS489	CDC59_23590	85.82% IS1421_aa3	99.62% CDC59_16900	IS5 ssgr IS427	1930918	1930113	806
RS489	CDC59_23730	85.31% IS1421_aa3	80.22% CDC59_21355	IS5 ssgr IS427	1956676	1957209	534
RS489	CDC59_16410	99.08% IS1021_aa1	100% CDC59_23225	IS5 ssgr IS5	108776	109762	987
RS489	CDC59_16450	98.78% IS1021_aa1	100% CDC59_23490	IS5 ssgr IS5	123578	124564	987
RS489	CDC59_16555	98.78% IS1021_aa1	100% CDC59_23490	IS5 ssgr IS5	154118	155104	987

RS489	CDC59_16600	98.47% IS1021_aa1	99.69% CDC59_23645	IS5 ssgr IS5	178728	177742	987
RS489	CDC59_16685	98.47% IS1021_aa1	99.69% CDC59_23645	IS5 ssgr IS5	195534	19652	987
RS489	CDC59_16760	98.47% IS1021_aa1	99.69% CDC59_23645	IS5 ssgr IS5	219742	218756	987
RS489	CDC59_16945	99.08% IS1021_aa1	100% CDC59_23225	IS5 ssgr IS5	266225	265239	987
RS489	CDC59_17370	98.47% IS1021_aa1	99.69% CDC59_23645	IS5 ssgr IS5	353084	35407	987
RS489	CDC59_17395	98.78% IS1021_aa1	100% CDC59_23645	IS5 ssgr IS5	361422	360436	987
RS489	CDC59_17455	69.64% ISPsp3_aa1	60% CDC59_23225	IS5 ssgr IS5	381429	381205	225
RS489	CDC59_17470	99.08% IS1021_aa1	100% CDC59_23225	IS5 ssgr IS5	383588	384574	987
RS489	CDC59_17475	98.47% IS1021_aa1	99.69% CDC59_23645	IS5 ssgr IS5	385975	384989	987
RS489	CDC59_17650	98.78% IS1021_aa1	100% CDC59_23645	IS5 ssgr IS5	435277	434291	987
RS489	CDC59_17985	98.78% IS1021_aa1	100% CDC59_23490	IS5 ssgr IS5	522579	523565	987
RS489	CDC59_17995	99.08% IS1021_aa1	100% CDC59_23225	IS5 ssgr IS5	525318	524332	987
RS489	CDC59_18005	98.47% IS1021_aa1	99.69% CDC59_23645	IS5 ssgr IS5	526807	525821	987
RS489	CDC59_18045	98.78% IS1021_aa1	100% CDC59_23490	IS5 ssgr IS5	533467	534453	987
RS489	CDC59_18500	98.78% IS1021_aa1	100% CDC59_23490	IS5 ssgr IS5	644773	645759	987
RS489	CDC59_18505	98.78% IS1021_aa1	100% CDC59_23490	IS5 ssgr IS5	64631	647296	987
RS489	CDC59_18545	98.78% IS1021_aa1	100% CDC59_23490	IS5 ssgr IS5	658083	659069	987
RS489	CDC59_18750	98.78% IS1021_aa1	100% CDC59_23490	IS5 ssgr IS5	704691	705677	987
RS489	CDC59_18870	98.78% IS1021_aa1	100% CDC59_23645	IS5 ssgr IS5	736797	737783	987
RS489	CDC59_18925	98.47% IS1021_aa1	99.69% CDC59_23645	IS5 ssgr IS5	748632	747646	987
RS489	CDC59_18990	98.78% IS1021_aa1	100% CDC59_23490	IS5 ssgr IS5	759949	760935	987
RS489	CDC59_19570	98.47% IS1021_aa1	99.69% CDC59_23645	IS5 ssgr IS5	929469	928483	987
RS489	CDC59_19580	99.08% IS1021_aa1	100% CDC59_23225	IS5 ssgr IS5	931213	932199	987
RS489	CDC59_19675	98.47% IS1021_aa1	99.69% CDC59_23645	IS5 ssgr IS5	957964	95895	987
RS489	CDC59_20265	98.78% IS1021_aa1	100% CDC59_23645	IS5 ssgr IS5	1101523	1100537	987
RS489	CDC59_20490	98.78% IS1021_aa1	100% CDC59_23645	IS5 ssgr IS5	1154695	1153709	987
RS489	CDC59_21050	99.08% IS1021_aa1	100% CDC59_23225	IS5 ssgr IS5	1316953	1317939	987
RS489	CDC59_21230	99.08% IS1021_aa1	100% CDC59_23225	IS5 ssgr IS5	1362796	1361810	987
RS489	CDC59_21315	56.36% ISAzo41_aa1	No hit	IS5 ssgr IS5	1388237	1387792	446
RS489	CDC59_21325	98.47% IS1021_aa1	99.69% CDC59_23645	IS5 ssgr IS5	1390013	1389027	987
RS489	CDC59_21500	98.78% IS1021_aa1	100% CDC59_23490	IS5 ssgr IS5	1433165	1434151	987
RS489	CDC59_21975	98.47% IS1021_aa1	99.69% CDC59_23645	IS5 ssgr IS5	1554539	1555525	987
RS489	CDC59_22190	98.78% IS1021_aa1	100% CDC59_23490	IS5 ssgr IS5	1614035	1615021	987
RS489	CDC59_22325	98.78% IS1021_aa1	100% CDC59_23490	IS5 ssgr IS5	1650290	1649304	987
RS489	CDC59_22345	98.78% IS1021_aa1	100% CDC59_23490	IS5 ssgr IS5	1652182	1653168	987
RS489	CDC59_22385	99.08% IS1021_aa1	100% CDC59_23225	IS5 ssgr IS5	1658933	1659919	987
RS489	CDC59_22455	98.47% IS1021_aa1	99.69% CDC59_23645	IS5 ssgr IS5	1671033	1672019	987
RS489	CDC59_22635	98.47% IS1021_aa1	99.69% CDC59_23645	IS5 ssgr IS5	1710247	1711233	987
RS489	CDC59_22810	98.47% IS1021_aa1	99.69% CDC59_23645	IS5 ssgr IS5	1756115	1757101	987
RS489	CDC59_23035	98.78% IS1021_aa1	100% CDC59_23490	IS5 ssgr IS5	1821139	1820153	987
RS489	CDC59_23225	99.08% IS1021_aa1	100% CDC59_22385	IS5 ssgr IS5	1864403	1865389	987
RS489	CDC59_23295	88.48% ISBmu20_aa1	72.02% CDC59_23595	IS5 ssgr IS5	1882059	1881176	884
RS489	CDC59_23395	98.47% IS1021_aa1	99.69% CDC59_23645	IS5 ssgr IS5	1896152	1895166	987

RS489	CDC59_23490	98.78% IS1021_aa1	100% CDC59_23035	IS5 ssgr IS5	1910160	1911146	987
RS489	CDC59_23495	98.47% IS1021_aa1	99.69% CDC59_23645	IS5 ssgr IS5	1911373	1912359	987
RS489	CDC59_23595	98.47% IS1021_aa1	99.69% CDC59_23645	IS5 ssgr IS5	1931165	1932151	987
RS489	CDC59_23645	98.78% IS1021_aa1	100% CDC59_20490	IS5 ssgr IS5	1940693	1941679	987
RS489	CDC59_20495	42.39% ISWz1_aa1	42.45% CDC59_23930	IS91	1155296	1156996	1.701
RS489	CDC59_20540	70.07% ISKpn21_aa1	No hit	ISNCY ssgr IS1202	1175401	1174131	1.271
RS489	CDC59_21240	38.36% ISAzs34_aa2	37.74% CDC59_21400	ISNCY ssgr IS892	1367012	1365933	1.08
OE1	CP009763_00500	95.51% ISBma3_aa1	44.33% CP009763_00147	IS110	636803	635598	1.206
OE1	CP009763_00147	88.75% ISBcen4_aa1	No hit	IS110 ssgr IS1111	192609	193628	1.02
OE1	CP009763_01209	100% ISRso11_aa1	No hit	IS3 ssgr IS150	1595473	1596006	534
OE1	CP009763_01210	99.28% ISRso11_aa2	52.38% CP009763_00469	IS3 ssgr IS150	1596003	1596839	837
OE1	CP009763_00462	98.36% ISRso10_aa1	No hit	IS3 ssgr IS2	602159	602548	390
OE1	CP009763_00463	93.00% ISRso10_aa2	43.06% CP009763_01444	IS3 ssgr IS2	602662	603381	720
OE1	CP009763_01441	92.85% ISRso16_aa2	99.20% CP009763_01444	IS3 ssgr IS407	1890389	1890901	513
OE1	CP009763_01444	92.41% ISRso16_aa2	99.20% CP009763_01441	IS3 ssgr IS407	1892531	1891857	675
OE1	CP009763_00468	81.52% ISAisp2_aa1	No hit	IS3 ssgr IS51	606179	606457	279
OE1	CP009763_00469	89.34% ISAisp2_aa2	52.38% CP009763_01210	IS3 ssgr IS51	606454	607329	876
OE1	CP009763_00340	61.68% ISCro3_aa1	No hit	IS4	449065	450393	1.329
OE1	CP009763_01367	78.03% ISAzo5_aa1	No hit	IS4 ssgr IS50	1788844	1787519	1.326
OE1	CP009763_00556	98.54% ISRso1_aa1	99.27% CP009763_00777	IS5	74796	748784	825
OE1	CP009763_00647	99.27% ISRso1_aa1	100% CP009763_00777	IS5	857025	857849	825
OE1	CP009763_00777	99.27% ISRso1_aa1	100% CP009763_00647	IS5	1031855	1031031	825
OE1	CP009763_00511	86.06% IS1421_aa1	100% CP009763_00554	IS5 ssgr IS427	653847	65344	408
OE1	CP009763_00554	86.06% IS1421_aa1	100% CP009763_00511	IS5 ssgr IS427	746849	746442	408
OE1	CP009763_00157	99.37% IS1405_aa1	100% CP009763_00501	IS5 ssgr IS5	205724	206689	966
OE1	CP009763_00338	99.37% IS1405_aa1	100% CP009763_00501	IS5 ssgr IS5	446643	445678	966
OE1	CP009763_00474	99.37% IS1405_aa1	100% CP009763_00501	IS5 ssgr IS5	610244	611209	966
OE1	CP009763_00501	99.37% IS1405_aa1	100% CP009763_00474	IS5 ssgr IS5	637323	638288	966
OE1	CP009763_00648	100% ISRso17_aa1	100% CP009763_01329	IS701	857931	859262	1.332
OE1	CP009763_01329	100% ISRso17_aa1	100% CP009763_00648	IS701	1740675	1742006	1.332
OE1	CP009763_00780	43.40% ISWz1_aa1	44.13% CP009763_01490	IS91	1034662	1036362	1.701
OE1	CP009763_00794	71.39% ISKpn21_aa1	77.5% CP009763_00793	ISNCY ssgr IS1202	1051518	1050043	1.476
K60	NCTK01000002_00366	95.01% ISBma3_aa1	99.75% NCTK01000002_00645	IS110	436889	435684	1.206
K60	NCTK01000002_00586	95.51% ISBma3_aa1	100% NCTK01000002_00645	IS110	70647	705265	1.206
K60	NCTK01000002_00645	95.51% ISBma3_aa1	100% NCTK01000002_00586	IS110	764248	765453	1.206
K60	NCTK01000002_01481	70.23% ISMmg1_aa1	No hit	IS110 ssgr IS1111	1857845	1857330	516
K60	NCTK01000002_01482	70.73% ISSpi6_aa1	No hit	IS110 ssgr IS1111	1858120	1857797	324
K60	NCTK01000002_00708	91.76% ISPosp3_aa1	No hit	IS1595 ssgr ISSod11	83532	835583	264
K60	NCTK01000002_00478	92.75% ISRso19_aa2	47.10% NCTK01000002_00689	IS21	579569	57915	420
K60	NCTK01000002_00479	81.81% ISRso19_aa1	No hit	IS21	579936	579601	336
K60	NCTK01000002_00480	94.63% ISRso19_aa1	44.38% NCTK01000002_00690	IS21	580559	579876	684

K60	NCTK01000002_00689	98.08% ISRme4_aa2	40.63% NCTK01000002_00698	IS21	815984	815199	786
K60	NCTK01000002_00690	81.08% IS1600_aa1	44.38% NCTK01000002_00480	IS21	8175	815974	1.527
K60	NCTK01000002_00698	85.14% ISCARN95_aa2	40.63% NCTK01000002_00689	IS21	827007	82624	768
K60	NCTK01000002_00699	82.88% ISCARN95_aa1	No hit	IS21	828144	82702	1.125
K60	NCTK01000002_01501	94.64% ISRso20_aa2	56.56% NCTK01000002_01000	IS3 ssgr IS3	1876759	1877601	843
K60	NCTK01000002_00486	98.31% IS401_aa2	99.66% NCTK01000002_01000	IS3 ssgr IS51	587288	586287	1.002
K60	NCTK01000002_00487	96.26% IS401_aa1	100% NCTK01000002_00999	IS3 ssgr IS51	587608	587285	324
K60	NCTK01000002_00999	96.26% IS401_aa1	100% NCTK01000002_00487	IS3 ssgr IS51	1303891	1304214	324
K60	NCTK01000002_01000	97.03% IS401_aa2	99.66% NCTK01000002_00486	IS3 ssgr IS51	1304211	1305125	915
K60	NCTK01000002_00463	76.99% ISAzo5_aa1	98.84% NCTK01000002_00696	IS4 ssgr IS50	559202	56053	1.329
K60	NCTK01000002_00671	82.55% ISAzo5_aa1	100% NCTK01000002_01484	IS4 ssgr IS50	793114	79341	297
K60	NCTK01000002_00672	78.17% ISAzo5_aa1	100% NCTK01000002_01483	IS4 ssgr IS50	79341	794159	750
K60	NCTK01000002_00691	82.16% ISAzo5_aa1	100% NCTK01000002_00695	IS4 ssgr IS50	818346	819605	1.26
K60	NCTK01000002_00695	82.16% ISAzo5_aa1	100% NCTK01000002_00691	IS4 ssgr IS50	824277	823018	1.26
K60	NCTK01000002_00696	77.23% ISAzo5_aa1	98.84% NCTK01000002_00463	IS4 ssgr IS50	824487	825803	1.317
K60	NCTK01000002_00994	81.15% ISAzo5_aa1	99.39% NCTK01000002_00695	IS4 ssgr IS50	1296898	1295897	1.002
K60	NCTK01000002_01267	80.91% ISAzo5_aa1	94.71% NCTK01000002_00695	IS4 ssgr IS50	1612132	1610939	1.194
K60	NCTK01000002_01479	70.75% ISAzo5_aa1	99.09% NCTK01000002_00463	IS4 ssgr IS50	1856260	1856715	456
K60	NCTK01000002_01483	78.17% ISAzo5_aa1	100% NCTK01000002_00672	IS4 ssgr IS50	1858996	1858247	750
K60	NCTK01000002_01484	79.67% ISAzo5_aa1	98.55% NCTK01000002_01500	IS4 ssgr IS50	1859448	1858954	495
K60	NCTK01000002_01500	77.38% ISAzo5_aa1	83.03% NCTK01000002_01267	IS4 ssgr IS50	1876583	1875258	1.326
K60	NCTK01000002_00396	62.5% ISMtsp16_aa1	No hit	IS481	469432	469202	231
K60	NCTK01000002_00579	96.71% ISRso1_aa1	No hit	IS5	698912	698088	825
K60	NCTK01000002_00163	65.28% ISCaa13_aa2	100% NCTK01000002_00966	IS5 ssgr IS427	198648	198283	366
K60	NCTK01000002_00610	65.28% ISCaa13_aa2	100% NCTK01000002_00966	IS5 ssgr IS427	729492	729127	366
K60	NCTK01000002_00721	64.46% ISCaa13_aa2	100% NCTK01000002_00610	IS5 ssgr IS427	852543	852908	366
K60	NCTK01000002_00966	65.28% ISCaa13_aa2	100% NCTK01000002_00163	IS5 ssgr IS427	1258053	1257688	366
K60	NCTK01000002_00483	61.32% ISAzo11_aa1	100% NCTK01000002_00485	IS5 ssgr IS5	582604	583674	1.071
K60	NCTK01000002_00485	61.32% ISAzo11_aa1	100% NCTK01000002_00483	IS5 ssgr IS5	586342	585272	1.071
K60	NCTK01000002_00659	85.96% ISRso9_aa1	No hit	IS5 ssgr IS5	779148	779336	189
K60	NCTK01000002_00660	96.26% ISRso9_aa1	No hit	IS5 ssgr IS5	779379	779783	405
K60	NCTK01000002_01360	94.89% ISRso18_aa1	82.75% NCTK01000002_01356	IS5 ssgr IS5	1715525	1715962	438
K60	NCTK01000002_00024	77.87% IS1420_aa1	100% NCTK01000002_01551	IS5 ssgr IS903	24786	24151	636
K60	NCTK01000002_01551	77.87% IS1420_aa1	100% NCTK01000002_00024	IS5 ssgr IS903	1927524	1926889	636
K60	NCTK01000002_00075	85.63% ISAzo9_aa2	100% NCTK01000002_00141	IS630	79877	81004	1.128
K60	NCTK01000002_00117	89.67% ISCARN25_aa1	98.94% NCTK01000002_01301	IS630	130151	13117	1.02
K60	NCTK01000002_00138	91.95% ISAzo32_aa1	99.69% NCTK01000002_00464	IS630	156693	15767	978
K60	NCTK01000002_00141	85.63% ISAzo9_aa2	100% NCTK01000002_00075	IS630	160519	161646	1.128
K60	NCTK01000002_00459	85.63% ISAzo9_aa2	99.73% NCTK01000002_01271	IS630	546198	547325	1.128
K60	NCTK01000002_00464	91.95% ISAzo32_aa1	99.69% NCTK01000002_00138	IS630	560812	561789	978
K60	NCTK01000002_00476	85.63% ISAzo9_aa2	100% NCTK01000002_01271	IS630	57748	578607	1.128
K60	NCTK01000002_00692	90.97% ISCARN25_aa1	100% NCTK01000002_01300	IS630	82141	821844	435
K60	NCTK01000002_00874	80.77% ISRso5_aa1	44.59% NCTK01000002_00967	IS630	1114866	1113790	1.077

K60	NCTK01000002_00967	85.36% ISAzo9_aa2	99.73% NCTK01000002_00141	IS630	1259632	1258505	1.128
K60	NCTK01000002_01119	85.63% ISAzo9_aa2	100% NCTK01000002_01271	IS630	1449050	1450177	1.128
K60	NCTK01000002_01271	85.63% ISAzo9_aa2	100% NCTK01000002_01119	IS630	1614481	1615608	1.128
K60	NCTK01000002_01300	90.97% ISCARN25_aa1	100% NCTK01000002_00692	IS630	1648068	1647634	435
K60	NCTK01000002_01301	88.88% ISCARN25_aa1	98.94% NCTK01000002_00117	IS630	1648640	1648065	576
K60	NCTK01000002_01499	85.71% IS885_aa1	98.35% NCTK01000002_00464	IS630	1875251	1874685	567
K60	NCTK01000002_00592	66.90% ISCARN42_aa1	No hit	IS701	711714	711217	498
K60	NCTK01000002_00680	47.27% ISMno24_aa2	100% NCTK01000002_01494	IS91	804972	806045	1.074
K60	NCTK01000002_00688	46.59% ISMno24_aa2	97.49% NCTK01000002_01494	IS91	812807	813886	1.08
K60	NCTK01000002_00858	42.93% ISWz1_aa1	40.75% NCTK01000002_00021	IS91	1089626	1091332	1.707
K60	NCTK01000002_01494	47.27% ISMno24_aa2	100% NCTK01000002_00680	IS91	1870595	1871668	1.074
K60	NCTK01000002_00482	44.57% ISXc4_aa2	94.87% NCTK01000002_00486	Tn3	581877	582659	783
FJAT1458	CP016555_00182	88.75% ISBcen4_aa1	100% CP016555_00294	IS110 ssgr IS1111	199908	200927	1.02
FJAT1458	CP016555_00291	89.05% ISBcen4_aa1	97.08% CP016555_00182	IS110 ssgr IS1111	337338	337784	447
FJAT1458	CP016555_00294	87.14% ISBcen4_aa1	100% CP016555_00182	IS110 ssgr IS1111	3389	339535	636
FJAT1458	CP016555_00613	61.97% ISMno14_aa1	99.70% CP016555_01315	IS110 ssgr IS1111	742766	741753	1.014
FJAT1458	CP016555_01310	61.67% ISMno14_aa1	100% CP016555_01315	IS110 ssgr IS1111	1661854	1662867	1.014
FJAT1458	CP016555_01315	61.67% ISMno14_aa1	100% CP016555_01310	IS110 ssgr IS1111	1666446	1665433	1.014
FJAT1458	CP016555_00305	83.75% ISBusp4_aa1	No hit	IS1182	348365	34692	1.446
FJAT1458	CP016555_00257	50.44% ISLsp2_aa1	No hit	IS1595 ssgr ISPna2	303254	304531	1.278
FJAT1458	CP016555_00290	95.74% ISRso10_aa1	No hit	IS3 ssgr IS2	336983	337363	381
FJAT1458	CP016555_00295	93.41% ISRso10_aa2	44.70% CP016555_01314	IS3 ssgr IS2	340028	340759	732
FJAT1458	CP016555_01070	78.68% ISRso20_aa1	No hit	IS3 ssgr IS3	1359477	1359677	201
FJAT1458	CP016555_00612	80.13% ISDet2_aa2	65.64% CP016555_01314	IS3 ssgr IS407	741385	740885	501
FJAT1458	CP016555_00614	77.58% ISNmu2_aa2	No hit	IS3 ssgr IS407	743006	742797	210
FJAT1458	CP016555_00615	89.41% ISAtu5_aa1	No hit	IS3 ssgr IS407	743305	743045	261
FJAT1458	CP016555_01314	93.82% ISRso16_aa2	65.64% CP016555_00612	IS3 ssgr IS407	1665086	1664547	540
FJAT1458	CP016555_00323	81.52% ISAisp2_aa1	100% CP016555_00393	IS3 ssgr IS51	368163	368441	279
FJAT1458	CP016555_00324	89.34% ISAisp2_aa2	100% CP016555_00392	IS3 ssgr IS51	368438	369313	876
FJAT1458	CP016555_00392	89.34% ISAisp2_aa2	100% CP016555_00324	IS3 ssgr IS51	451444	450569	876
FJAT1458	CP016555_00393	81.52% ISAisp2_aa1	100% CP016555_00323	IS3 ssgr IS51	451719	451441	279
FJAT1458	CP016555_00935	81.52% ISAisp2_aa1	97.82% CP016555_00393	IS3 ssgr IS51	1164462	1164740	279
FJAT1458	CP016555_00936	89.34% ISAisp2_aa2	98.28% CP016555_00392	IS3 ssgr IS51	1164737	1165612	876
FJAT1458	CP016555_00106	61.68% ISCro3_aa1	No hit	IS4	108915	110243	1.329
FJAT1458	CP016555_00258	82.75% ISBcen26_aa1	No hit	IS481	305026	304676	351
FJAT1458	CP016555_00178	97.44% ISRso1_aa1	100% CP016555_00774	IS5	196738	195704	1.035
FJAT1458	CP016555_00464	98.54% ISRso1_aa1	96.61% CP016555_00178	IS5	573221	574654	1.434
FJAT1458	CP016555_00774	97.44% ISRso1_aa1	100% CP016555_00178	IS5	94446	943636	825
FJAT1458	CP016555_00776	99.25% IS1421_aa1	No hit	IS5 ssgr IS427	946921	946517	405
FJAT1458	CP016555_00103	94.51% IS1021_aa1	100% CP016555_01534	IS5 ssgr IS5	105028	106014	987
FJAT1458	CP016555_00165	99.06% IS1405_aa1	100% CP016555_01545	IS5 ssgr IS5	177455	17842	966
FJAT1458	CP016555_00167	94.51% IS1021_aa1	100% CP016555_01534	IS5 ssgr IS5	180657	179671	987

FJAT1458	CP016555_00210	99.06% IS1405_aa1	100% CP016555_01545	IS5 ssgr IS5	242597	243562	966
FJAT1458	CP016555_00292	99.06% IS1405_aa1	100% CP016555_01545	IS5 ssgr IS5	337789	338754	966
FJAT1458	CP016555_00302	99.06% IS1405_aa1	100% CP016555_01545	IS5 ssgr IS5	344455	34542	966
FJAT1458	CP016555_00308	94.51% IS1021_aa1	100% CP016555_01534	IS5 ssgr IS5	352565	351579	987
FJAT1458	CP016555_00309	99.06% IS1405_aa1	100% CP016555_01545	IS5 ssgr IS5	352723	353688	966
FJAT1458	CP016555_00387	99.06% IS1405_aa1	100% CP016555_01545	IS5 ssgr IS5	443679	442714	966
FJAT1458	CP016555_00518	99.06% IS1405_aa1	100% CP016555_01545	IS5 ssgr IS5	634964	635929	966
FJAT1458	CP016555_00610	94.51% IS1021_aa1	100% CP016555_01534	IS5 ssgr IS5	738451	739437	987
FJAT1458	CP016555_00773	99.37% IS1405_aa1	100% CP016555_00777	IS5 ssgr IS5	943413	942448	966
FJAT1458	CP016555_00777	99.37% IS1405_aa1	100% CP016555_00773	IS5 ssgr IS5	947047	948012	966
FJAT1458	CP016555_00803	99.06% IS1405_aa1	100% CP016555_01545	IS5 ssgr IS5	981837	982802	966
FJAT1458	CP016555_01044	99.06% IS1405_aa1	100% CP016555_01545	IS5 ssgr IS5	1312700	1313665	966
FJAT1458	CP016555_01048	99.06% IS1405_aa1	100% CP016555_01545	IS5 ssgr IS5	1316095	1315130	966
FJAT1458	CP016555_01064	94.51% IS1021_aa1	100% CP016555_01534	IS5 ssgr IS5	1353817	1352831	987
FJAT1458	CP016555_01159	94.51% IS1021_aa1	100% CP016555_01534	IS5 ssgr IS5	1470748	1471734	987
FJAT1458	CP016555_01231	99.06% IS1405_aa1	100% CP016555_01545	IS5 ssgr IS5	1554265	1553300	966
FJAT1458	CP016555_01232	94.51% IS1021_aa1	100% CP016555_01534	IS5 ssgr IS5	1555456	1554470	987
FJAT1458	CP016555_01420	94.51% IS1021_aa1	100% CP016555_01534	IS5 ssgr IS5	1775833	1776819	987
FJAT1458	CP016555_01455	94.51% IS1021_aa1	100% CP016555_01534	IS5 ssgr IS5	1818778	1817792	987
FJAT1458	CP016555_01458	94.51% IS1021_aa1	100% CP016555_01534	IS5 ssgr IS5	1822467	1823453	987
FJAT1458	CP016555_01495	94.51% IS1021_aa1	100% CP016555_01534	IS5 ssgr IS5	1879312	1880298	987
FJAT1458	CP016555_01534	94.51% IS1021_aa1	100% CP016555_01495	IS5 ssgr IS5	1923613	1922627	987
FJAT1458	CP016555_01545	99.06% IS1405_aa1	100% CP016555_01231	IS5 ssgr IS5	1938242	1939207	966
FJAT1458	CP016555_00260	81.35% ISCARN39_aa3	No hit	IS630	307395	306859	537
FJAT1458	CP016555_00627	43.40% ISWz1_aa1	44.13% CP016555_01363	IS91	757187	755487	1.701
FJAT1458	CP016555_00486	100% ISRso21_aa2	100% CP016555_01329	ISL3	600772	602193	1.422
FJAT1458	CP016555_00538	100% ISRso21_aa2	100% CP016555_01329	ISL3	655204	653783	1.422
FJAT1458	CP016555_00802	100% ISRso21_aa2	100% CP016555_01329	ISL3	980261	981682	1.422
FJAT1458	CP016555_00941	100% ISRso21_aa2	100% CP016555_01329	ISL3	1171476	1170055	1.422
FJAT1458	CP016555_01329	100% ISRso21_aa2	100% CP016555_00941	ISL3	1682057	1680636	1.422
FJAT1458	CP016555_00605	72.27% ISKpn21_aa1	70% CP016555_00606	ISNCY ssgr IS1202	73329	734765	1.476
FJAT1458	CP016555_00606	62.5% ISAba32_aa1	70% CP016555_00605	ISNCY ssgr IS1202	734908	735123	216
FJAT1458	CP016555_00222	50.25% ISMpo10_aa3	No hit	Tn3	256539	255166	1.374
EP1	CP015116_00708	95.51% ISBma3_aa1	100% CP015116_00728	IS110	877461	876256	1.206
EP1	CP015116_00728	95.51% ISBma3_aa1	100% CP015116_00708	IS110	902496	901291	1.206
EP1	CP015116_00286	88.75% ISBcen4_aa1	No hit	IS110 ssgr IS1111	351722	352741	1.02
EP1	CP015116_00632	50.44% ISLsp2_aa1	No hit	IS1595 ssgr ISPna2	805608	806885	1.278
EP1	CP015116_01461	100% ISRso11_aa1	No hit	IS3 ssgr IS150	1881652	1882185	534
EP1	CP015116_01462	99.28% ISRso11_aa2	52.38% CP015116_00672	IS3 ssgr IS150	1882182	1883018	837
EP1	CP015116_00665	98.36% ISRso10_aa1	No hit	IS3 ssgr IS2	839239	839628	390
EP1	CP015116_00666	93.00% ISRso10_aa2	43.06% CP015116_00089	IS3 ssgr IS2	839742	840461	720
EP1	CP015116_00086	92.85% ISRso16_aa2	99.20% CP015116_00089	IS3 ssgr IS407	112851	113363	513

EP1	CP015116_00089	92.41% ISRso16_aa2	99.20% CP015116_00086	IS3 ssgr IS407	114993	114319	675
EP1	CP015116_00671	81.52% ISAisp2_aa1	No hit	IS3 ssgr IS51	843259	843537	279
EP1	CP015116_00672	89.34% ISAisp2_aa2	52.38% CP015116_01462	IS3 ssgr IS51	843534	844409	876
EP1	CP015116_00476	61.68% ISCro3_aa1	No hit	IS4	606996	608324	1.329
EP1	CP015116_00012	78.03% ISAzo5_aa1	No hit	IS4 ssgr IS50	12642	11317	1.326
EP1	CP015116_00633	82.75% ISBcen26_aa1	No hit	IS481	80738	80703	351
EP1	CP015116_01647	38.76% ISGur11_aa1	No hit	IS481	2090078	2088396	1.683
EP1	CP015116_00785	98.54% ISRso1_aa1	99.27% CP015116_01025	IS5	1014830	1015654	825
EP1	CP015116_00879	99.27% ISRso1_aa1	100% CP015116_01025	IS5	1123947	1124771	825
EP1	CP015116_01025	99.27% ISRso1_aa1	100% CP015116_00879	IS5	1316078	1315254	825
EP1	CP015116_00739	86.06% IS1421_aa1	100% CP015116_00783	IS5 ssgr IS427	91954	919133	408
EP1	CP015116_00783	86.06% IS1421_aa1	100% CP015116_00739	IS5 ssgr IS427	1013719	1013312	408
EP1	CP015116_00296	99.37% IS1405_aa1	100% CP015116_01332	IS5 ssgr IS5	364837	365802	966
EP1	CP015116_00677	99.37% IS1405_aa1	100% CP015116_01332	IS5 ssgr IS5	847324	848289	966
EP1	CP015116_00729	99.37% IS1405_aa1	100% CP015116_01332	IS5 ssgr IS5	903016	903981	966
EP1	CP015116_00782	99.37% IS1405_aa1	100% CP015116_01332	IS5 ssgr IS5	1012847	1011882	966
EP1	CP015116_01332	99.37% IS1405_aa1	100% CP015116_00782	IS5 ssgr IS5	1713141	1714106	966
EP1	CP015116_00635	81.35% ISCARN39_aa3	No hit	IS630	809749	809213	537
EP1	CP015116_00682	74.13% ISRm2_aa2	No hit	IS66	855343	855696	354
EP1	CP015116_00683	59.09% ISAeh1_aa2	No hit	IS66	855729	857285	1.557
EP1	CP015116_00880	100% ISRso17_aa1	No hit	IS701	1124853	1126184	1.332
EP1	CP015116_01028	43.40% ISWz1_aa1	78.46% CP015116_01016	IS91	1318885	1320585	1.701
EP1	CP015116_01042	71.39% ISKpn21_aa1	77.5% CP015116_01041	ISNCY ssgr IS1202	1335741	1334266	1.476
EP1	CP015116_00597	50.25% ISMpo10_aa3	No hit	Tn3	758893	75752	1.374
EP1	CP015116_01017	65.81% ISPa43_aa2	No hit	Tn3	1301175	1304159	2.985
CQPS-1	CP016915_01614	53.92% ISNme3_aa1	No hit	IS1595 ssgr IS1016	2012868	2013212	345
CQPS-1	CP016915_00213	100% ISRso11_aa1	No hit	IS3 ssgr IS150	249272	2497	429
CQPS-1	CP016915_00214	99.28% ISRso11_aa2	52.38% CP016915_01625	IS3 ssgr IS150	249697	250533	837
CQPS-1	CP016915_01098	97.72% ISButh1_aa1	84.16% CP016915_01632	IS3 ssgr IS2	1330375	1330776	402
CQPS-1	CP016915_01099	93.88% ISButh1_aa2	75.93% CP016915_01631	IS3 ssgr IS2	1330773	1331609	837
CQPS-1	CP016915_01631	93.00% ISRso10_aa2	77.17% CP016915_01099	IS3 ssgr IS2	2028380	2027661	720
CQPS-1	CP016915_01632	98.31% ISRso10_aa1	84.16% CP016915_01098	IS3 ssgr IS2	2028882	2028427	456
CQPS-1	CP016915_00459	92.85% ISRso16_aa2	99.20% CP016915_00462	IS3 ssgr IS407	548203	548715	513
CQPS-1	CP016915_00462	92.41% ISRso16_aa2	99.20% CP016915_00459	IS3 ssgr IS407	550345	549671	675
CQPS-1	CP016915_01144	100% ISRso12_aa1	82.85% CP016915_00463	IS3 ssgr IS407	1388669	1388935	267
CQPS-1	CP016915_01145	100% ISRso12_aa2	72.05% CP016915_00462	IS3 ssgr IS407	1389085	1389789	705
CQPS-1	CP016915_01625	89.34% ISAisp2_aa2	52.38% CP016915_00214	IS3 ssgr IS51	2024589	2023714	876
CQPS-1	CP016915_01626	81.52% ISAisp2_aa1	No hit	IS3 ssgr IS51	2024864	2024586	279
CQPS-1	CP016915_00257	98.90% ISRso1_aa1	100% CP016915_01309	IS5	298678	297854	825
CQPS-1	CP016915_01082	96.96% ISRso1_aa1	96.96% CP016915_01309	IS5	1318279	1318079	201
CQPS-1	CP016915_01093	98.90% ISRso1_aa1	100% CP016915_01309	IS5	1328159	1327335	825
CQPS-1	CP016915_01309	98.90% ISRso1_aa1	100% CP016915_01093	IS5	1590588	1591412	825

CQPS-1	CP016915_01440	99.27% ISRso1_aa1	99.63% CP016915_01309	IS5	1765612	1764788	825
CQPS-1	CP016915_01535	96.59% ISRso1_aa1	95.45% CP016915_01440	IS5	1875521	1875150	372
CQPS-1	CP016915_00189	86.06% IS1421_aa1	100% CP016915_00906	IS5 ssgr IS427	206283	20669	408
CQPS-1	CP016915_00605	86.06% IS1421_aa1	100% CP016915_00906	IS5 ssgr IS427	71061	711017	408
CQPS-1	CP016915_00881	86.06% IS1421_aa1	100% CP016915_00906	IS5 ssgr IS427	1070308	1069901	408
CQPS-1	CP016915_00906	86.06% IS1421_aa1	100% CP016915_00881	IS5 ssgr IS427	1095459	1095866	408
CQPS-1	CP016915_00013	99.37% IS1405_aa1	100% CP016915_01619	IS5 ssgr IS5	14491	13526	966
CQPS-1	CP016915_00039	99.37% IS1405_aa1	100% CP016915_01619	IS5 ssgr IS5	31809	32774	966
CQPS-1	CP016915_00081	99.37% IS1405_aa1	100% CP016915_01619	IS5 ssgr IS5	81699	80734	966
CQPS-1	CP016915_00342	99.37% IS1405_aa1	100% CP016915_01619	IS5 ssgr IS5	397999	397034	966
CQPS-1	CP016915_00689	99.37% IS1405_aa1	100% CP016915_01619	IS5 ssgr IS5	823693	824658	966
CQPS-1	CP016915_01459	94.51% IS1021_aa1	71.80% CP016915_01619	IS5 ssgr IS5	1789988	1790974	987
CQPS-1	CP016915_01619	99.37% IS1405_aa1	100% CP016915_00689	IS5 ssgr IS5	2020683	2019718	966
CQPS-1	CP016915_01643	99.05% IS1420_aa1	No hit	IS5 ssgr IS903	2039416	2040372	957
CQPS-1	CP016915_00192	100% ISRso17_aa1	100% CP016915_01439	IS701	207768	209099	1.332
CQPS-1	CP016915_00258	100% ISRso17_aa1	100% CP016915_01439	IS701	300135	298804	1.332
CQPS-1	CP016915_00875	99.69% ISRso17_aa1	99.69% CP016915_01439	IS701	1062747	1061731	1.017
CQPS-1	CP016915_01439	100% ISRso17_aa1	100% CP016915_00258	IS701	1764706	1763375	1.332
CQPS-1	CP016915_01292	71.39% ISKpn21_aa1	77.5% CP016915_01293	ISNCY ssgr IS1202	1570940	1572415	1.476
CQPS-1	CP016915_00059	50.25% ISMpo10_aa3	No hit	Tn3	50834	52207	1.374

FJAT91	IS_9cef4912_00708	95.51% ISBma3_aa1	100% IS_9cef4912_00728	IS110	877461	876256	1.206
FJAT91	IS_9cef4912_00728	95.51% ISBma3_aa1	100% IS_9cef4912_00708	IS110	902496	901291	1.206
FJAT91	IS_9cef4912_00286	88.75% ISBcen4_aa1	No hit	IS110 ssgr IS1111	351722	352741	1.02
FJAT91	IS_9cef4912_00632	50.44% ISLsp2_aa1	No hit	IS1595 ssgr ISPna2	805608	806885	1.278
FJAT91	IS_9cef4912_01461	100% ISRso11_aa1	No hit	IS3 ssgr IS150	1881652	1882185	534
FJAT91	IS_9cef4912_01462	99.28% ISRso11_aa2	52.38% IS_9cef4912_00672	IS3 ssgr IS150	1882182	1883018	837
FJAT91	IS_9cef4912_00665	98.36% ISRso10_aa1	No hit	IS3 ssgr IS2	839239	839628	390
FJAT91	IS_9cef4912_00666	93.00% ISRso10_aa2	43.06% IS_9cef4912_00089	IS3 ssgr IS2	839742	840461	720
FJAT91	IS_9cef4912_00086	92.85% ISRso16_aa2	99.20% IS_9cef4912_00089	IS3 ssgr IS407	112851	113363	513
FJAT91	IS_9cef4912_00089	92.41% ISRso16_aa2	99.20% IS_9cef4912_00086	IS3 ssgr IS407	114993	114319	675
FJAT91	IS_9cef4912_00671	81.52% ISAisp2_aa1	No hit	IS3 ssgr IS51	843259	843537	279
FJAT91	IS_9cef4912_00672	89.34% ISAisp2_aa2	52.38% IS_9cef4912_01462	IS3 ssgr IS51	843534	844409	876
FJAT91	IS_9cef4912_00476	61.68% ISCro3_aa1	No hit	IS4	606996	608324	1.329
FJAT91	IS_9cef4912_00012	78.03% ISAzo5_aa1	No hit	IS4 ssgr IS50	12642	11317	1.326
FJAT91	IS_9cef4912_00633	82.75% ISBcen26_aa1	No hit	IS481	80738	80703	351
FJAT91	IS_9cef4912_01647	38.76% ISGur11_aa1	No hit	IS481	2090078	2088396	1.683
FJAT91	IS_9cef4912_00785	98.54% ISRso1_aa1	99.27% IS_9cef4912_01025	IS5	1014830	1015654	825
FJAT91	IS_9cef4912_00879	99.27% ISRso1_aa1	100% IS_9cef4912_01025	IS5	1123947	1124771	825
FJAT91	IS_9cef4912_01025	99.27% ISRso1_aa1	100% IS_9cef4912_00879	IS5	1316078	1315254	825
FJAT91	IS_9cef4912_00739	86.06% IS1421_aa1	100% IS_9cef4912_00783	IS5 ssgr IS427	91954	919133	408
FJAT91	IS_9cef4912_00783	86.06% IS1421_aa1	100% IS_9cef4912_00739	IS5 ssgr IS427	1013719	1013312	408
FJAT91	IS_9cef4912_00296	99.37% IS1405_aa1	100% IS_9cef4912_01332	IS5 ssgr IS5	364837	365802	966

FJAT91	IS_9cef4912_00677	99.37% IS1405_aa1	100% IS_9cef4912_01332	IS5 ssgr IS5	847324	848289	966
FJAT91	IS_9cef4912_00729	99.37% IS1405_aa1	100% IS_9cef4912_01332	IS5 ssgr IS5	903016	903981	966
FJAT91	IS_9cef4912_00782	99.37% IS1405_aa1	100% IS_9cef4912_01332	IS5 ssgr IS5	1012847	1011882	966
FJAT91	IS_9cef4912_01332	99.37% IS1405_aa1	100% IS_9cef4912_00782	IS5 ssgr IS5	1713141	1714106	966
FJAT91	IS_9cef4912_00635	81.35% ISCARN39_aa3	No hit	IS630	809749	809213	537
FJAT91	IS_9cef4912_00682	74.13% ISRm2_aa2	No hit	IS66	855343	855696	354
FJAT91	IS_9cef4912_00683	59.09% ISAeh1_aa2	No hit	IS66	855729	857285	1.557
FJAT91	IS_9cef4912_00880	100% ISRso17_aa1	No hit	IS701	1124853	1126184	1.332
FJAT91	IS_9cef4912_01028	43.40% ISWz1_aa1	78.46% IS_9cef4912_01016	IS91	1318885	1320585	1.701
FJAT91	IS_9cef4912_01042	71.39% ISKpn21_aa1	77.5% IS_9cef4912_01041	ISNCY ssgr IS1202	1335741	1334266	1.476
FJAT91	IS_9cef4912_00597	50.25% ISMpo10_aa3	No hit	Tn3	758893	75752	1.374
FJAT91	IS_9cef4912_01017	65.81% ISPa43_aa2	No hit	Tn3	1301175	1304159	2.985
FQY-4	CP004013_00542	95.76% ISBma3_aa1	100% CP004013_01655	IS110	681257	682462	1.206
FQY-4	CP004013_01655	95.76% ISBma3_aa1	100% CP004013_00542	IS110	2064693	2063488	1.206
FQY-4	CP004013_00360	88.75% ISBcen4_aa1	100% CP004013_00404	IS110 ssgr IS1111	462061	461042	1.02
FQY-4	CP004013_00404	88.75% ISBcen4_aa1	100% CP004013_00360	IS110 ssgr IS1111	523146	522127	1.02
FQY-4	CP004013_00604	82.67% IS1383_aa1	72.72% CP004013_00404	IS110 ssgr IS1111	745807	744806	1.002
FQY-4	CP004013_00097	94.07% ISBcen18_aa1	No hit	IS256	111329	112606	1.278
FQY-4	CP004013_00359	97.14% ISRso10_aa2	44.49% CP004013_01630	IS3 ssgr IS2	460508	459816	693
FQY-4	CP004013_00361	94.68% ISRso10_aa1	No hit	IS3 ssgr IS2	462416	462036	381
FQY-4	CP004013_00578	98.86% ISSod2_aa1	62.12% CP004013_01634	IS3 ssgr IS407	722394	72266	267
FQY-4	CP004013_00579	84.79% ISAs22_aa3	58.42% CP004013_01630	IS3 ssgr IS407	722687	723481	795
FQY-4	CP004013_01630	93.30% ISRso16_aa2	100% CP004013_01633	IS3 ssgr IS407	2042143	2042817	675
FQY-4	CP004013_01633	93.65% ISRso16_aa2	100% CP004013_01630	IS3 ssgr IS407	2044285	2043773	513
FQY-4	CP004013_01556	89.34% ISAisp2_aa2	42.15% CP004013_01630	IS3 ssgr IS51	1945616	1944741	876
FQY-4	CP004013_01557	81.52% ISAisp2_aa1	No hit	IS3 ssgr IS51	1945891	1945613	279
FQY-4	CP004013_00098	89.55% ISHar5_aa1	59.25% CP004013_00252	IS30	112878	112615	264
FQY-4	CP004013_00252	65.95% IS1382_aa1	100% CP004013_00832	IS30	343171	342155	1.017
FQY-4	CP004013_00832	65.95% IS1382_aa1	100% CP004013_00252	IS30	1034671	1033619	1.053
FQY-4	CP004013_00476	61.68% ISCro3_aa1	No hit	IS4	6097	611028	1.329
FQY-4	CP004013_01013	76.86% ISAzo5_aa1	No hit	IS4 ssgr IS50	1258418	1259725	1.308
FQY-4	CP004013_00622	96.96% ISRso1_aa1	96.96% CP004013_01014	IS5	76597	76577	201
FQY-4	CP004013_01014	97.81% ISRso1_aa1	95.45% CP004013_01104	IS5	1259797	1260621	825
FQY-4	CP004013_01104	96.59% ISRso1_aa1	95.45% CP004013_01014	IS5	1367477	1367136	342
FQY-4	CP004013_00044	100% IS1421_aa1	100% CP004013_01009	IS5 ssgr IS427	5178	52184	405
FQY-4	CP004013_00094	100% IS1421_aa1	100% CP004013_01009	IS5 ssgr IS427	109255	109659	405
FQY-4	CP004013_00362	100% IS1421_aa1	100% CP004013_01177	IS5 ssgr IS427	463236	462832	405
FQY-4	CP004013_00382	100% IS1421_aa1	100% CP004013_01177	IS5 ssgr IS427	494166	493762	405
FQY-4	CP004013_00565	61.34% ISNeu3_aa3	48.78% CP004013_01009	IS5 ssgr IS427	706376	706759	384
FQY-4	CP004013_00686	100% IS1421_aa1	100% CP004013_01009	IS5 ssgr IS427	8446	845004	405
FQY-4	CP004013_01009	100% IS1421_aa1	100% CP004013_00686	IS5 ssgr IS427	1254659	1255063	405
FQY-4	CP004013_01177	100% IS1421_aa1	100% CP004013_00382	IS5 ssgr IS427	1464255	1464659	405

FQY-4	CP004013_00055	99.06% IS1405_aa1	100% CP004013_01378	IS5 ssgr IS5	62441	63406	966
FQY-4	CP004013_00093	94.81% IS1021_aa1	100% CP004013_01543	IS5 ssgr IS5	109051	108065	987
FQY-4	CP004013_00378	94.51% IS1021_aa1	100% CP004013_01558	IS5 ssgr IS5	480639	479653	987
FQY-4	CP004013_00557	99.06% IS1405_aa1	99.37% CP004013_01378	IS5 ssgr IS5	700152	701117	966
FQY-4	CP004013_00562	98.03% IS1405_aa1	99.34% CP004013_01378	IS5 ssgr IS5	702883	702224	660
FQY-4	CP004013_00700	94.81% IS1021_aa1	100% CP004013_01543	IS5 ssgr IS5	863352	862366	987
FQY-4	CP004013_00735	94.51% IS1021_aa1	100% CP004013_01558	IS5 ssgr IS5	911034	91202	987
FQY-4	CP004013_01011	94.51% IS1021_aa1	100% CP004013_01558	IS5 ssgr IS5	1257409	1256423	987
FQY-4	CP004013_01162	99.06% IS1405_aa1	100% CP004013_01378	IS5 ssgr IS5	1452429	1453394	966
FQY-4	CP004013_01346	94.81% IS1021_aa1	100% CP004013_01543	IS5 ssgr IS5	1678521	1679507	987
FQY-4	CP004013_01354	94.51% IS1021_aa1	100% CP004013_01558	IS5 ssgr IS5	1687125	1688111	987
FQY-4	CP004013_01355	94.51% IS1021_aa1	100% CP004013_01558	IS5 ssgr IS5	1689546	1688560	987
FQY-4	CP004013_01378	99.06% IS1405_aa1	100% CP004013_01162	IS5 ssgr IS5	1727522	1728487	966
FQY-4	CP004013_01470	94.51% IS1021_aa1	100% CP004013_01558	IS5 ssgr IS5	1840249	1841235	987
FQY-4	CP004013_01543	94.81% IS1021_aa1	100% CP004013_01346	IS5 ssgr IS5	1928049	1929035	987
FQY-4	CP004013_01558	94.51% IS1021_aa1	100% CP004013_01470	IS5 ssgr IS5	1947035	1946049	987
FQY-4	CP004013_00355	56.95% ISRm2_aa1	100% CP004013_01475	IS66	456507	456989	483
FQY-4	CP004013_00356	73.27% ISRm2_aa2	100% CP004013_01474	IS66	456986	457339	354
FQY-4	CP004013_00357	59.58% ISAeh1_aa2	100% CP004013_01473	IS66	457372	458928	1.557
FQY-4	CP004013_00589	59.58% ISAeh1_aa2	100% CP004013_01473	IS66	734415	732859	1.557
FQY-4	CP004013_00590	73.27% ISRm2_aa2	100% CP004013_01474	IS66	734801	734448	354
FQY-4	CP004013_00591	56.95% ISRm2_aa1	100% CP004013_01475	IS66	73528	734798	483
FQY-4	CP004013_00836	59.58% ISAeh1_aa2	100% CP004013_01473	IS66	1042700	1041144	1.557
FQY-4	CP004013_00837	73.27% ISRm2_aa2	100% CP004013_01474	IS66	1043086	1042733	354
FQY-4	CP004013_00838	56.95% ISRm2_aa1	100% CP004013_01475	IS66	1043565	1043083	483
FQY-4	CP004013_01473	59.58% ISAeh1_aa2	100% CP004013_00836	IS66	1846003	1844447	1.557
FQY-4	CP004013_01474	73.27% ISRm2_aa2	100% CP004013_00837	IS66	1846389	1846036	354
FQY-4	CP004013_01475	56.95% ISRm2_aa1	100% CP004013_00838	IS66	1846868	1846386	483
FQY-4	CP004013_00855	43.40% ISWz1_aa1	40.54% CP004013_01680	IS91	1061609	1059909	1.701
FQY-4	CP004013_00833	73.76% ISKpn21_aa1	No hit	ISNCY ssgr IS1202	1036350	1034761	1.59
FQY-4	CP004013_00567	0% newcandidate	not_found	New_Family	709418	709263	156
RSCM	CP025986_00285	61.67% ISMno14_aa1	100% CP025986_01553	IS110 ssgr IS1111	347436	348449	1.014
RSCM	CP025986_00293	61.67% ISMno14_aa1	100% CP025986_01553	IS110 ssgr IS1111	354974	353961	1.014
RSCM	CP025986_00375	82.67% IS1383_aa1	46.82% CP025986_01553	IS110 ssgr IS1111	461398	460397	1.002
RSCM	CP025986_01124	61.67% ISMno14_aa1	100% CP025986_01553	IS110 ssgr IS1111	1299996	1298983	1.014
RSCM	CP025986_01500	61.67% ISMno14_aa1	100% CP025986_01553	IS110 ssgr IS1111	1776679	1775666	1.014
RSCM	CP025986_01553	61.67% ISMno14_aa1	100% CP025986_01500	IS110 ssgr IS1111	1840850	1839837	1.014
RSCM	CP025986_00684	83.02% ISBusp4_aa1	100% CP025986_01692	IS1182	818176	819627	1.452
RSCM	CP025986_00704	83.02% ISBusp4_aa1	100% CP025986_01692	IS1182	84568	844229	1.452
RSCM	CP025986_01037	83.02% ISBusp4_aa1	100% CP025986_01692	IS1182	1197684	1196233	1.452
RSCM	CP025986_01186	82.06% ISBusp4_aa1	100% CP025986_01692	IS1182	1379056	1380363	1.308
RSCM	CP025986_01356	83.02% ISBusp4_aa1	100% CP025986_01692	IS1182	1602148	1603599	1.452

RSCM	CP025986_01551	83.02% ISBusp4_aa1	100% CP025986_01692	IS1182	1837161	1838612	1.452
RSCM	CP025986_01636	83.02% ISBusp4_aa1	100% CP025986_01692	IS1182	1924523	1923072	1.452
RSCM	CP025986_01692	83.02% ISBusp4_aa1	100% CP025986_01636	IS1182	2009066	2007615	1.452
RSCM	CP025986_01350	96.15% ISRso7_aa1	No hit	IS256	1596676	1595426	1.251
RSCM	CP025986_01763	100% ISRso11_aa1	No hit	IS3 ssgr IS150	2092500	2093033	534
RSCM	CP025986_01764	99.28% ISRso11_aa2	52.38% CP025986_01367	IS3 ssgr IS150	2093030	2093866	837
RSCM	CP025986_00705	98.36% ISRso10_aa1	83.73% CP025986_01555	IS3 ssgr IS2	845874	846263	390
RSCM	CP025986_00706	93.00% ISRso10_aa2	78.81% CP025986_01552	IS3 ssgr IS2	846377	847096	720
RSCM	CP025986_01501	89.33% ISButh1_aa2	100% CP025986_01554	IS3 ssgr IS2	1776946	1776710	237
RSCM	CP025986_01502	97.72% ISButh1_aa1	100% CP025986_01555	IS3 ssgr IS2	1777344	1776943	402
RSCM	CP025986_01552	94.87% ISButh1_aa2	78.81% CP025986_00706	IS3 ssgr IS2	1839337	1838984	354
RSCM	CP025986_01554	89.33% ISButh1_aa2	100% CP025986_01501	IS3 ssgr IS2	1841117	1840881	237
RSCM	CP025986_01555	97.72% ISButh1_aa1	100% CP025986_01502	IS3 ssgr IS2	1841515	1841114	402
RSCM	CP025986_00286	93.25% ISRso16_aa2	74.35% CP025986_01499	IS3 ssgr IS407	349184	349723	540
RSCM	CP025986_01118	81.5% ISXca1_aa2	74.85% CP025986_01123	IS3 ssgr IS407	1294227	1293601	627
RSCM	CP025986_01123	100% ISRso14_aa2	74.85% CP025986_01118	IS3 ssgr IS407	1298669	1298121	549
RSCM	CP025986_01125	100% ISRso14_aa2	No hit	IS3 ssgr IS407	1300245	1300027	219
RSCM	CP025986_01126	100% ISRso14_aa1	74.11% CP025986_01505	IS3 ssgr IS407	1300535	1300272	264
RSCM	CP025986_01499	100% ISRso12_aa2	74.35% CP025986_00286	IS3 ssgr IS407	1775388	1774804	585
RSCM	CP025986_01505	100% ISRso12_aa1	74.11% CP025986_01126	IS3 ssgr IS407	1780162	1779896	267
RSCM	CP025986_00140	89.34% ISAisp2_aa2	100% CP025986_01367	IS3 ssgr IS51	195306	194431	876
RSCM	CP025986_00141	81.52% ISAisp2_aa1	100% CP025986_01366	IS3 ssgr IS51	195581	195303	279
RSCM	CP025986_00271	81.52% ISAisp2_aa1	100% CP025986_01366	IS3 ssgr IS51	335462	33574	279
RSCM	CP025986_00272	89.34% ISAisp2_aa2	100% CP025986_01367	IS3 ssgr IS51	335737	336612	876
RSCM	CP025986_01187	89.34% ISAisp2_aa2	100% CP025986_01367	IS3 ssgr IS51	1381235	1380360	876
RSCM	CP025986_01188	81.52% ISAisp2_aa1	100% CP025986_01366	IS3 ssgr IS51	1381510	1381232	279
RSCM	CP025986_01189	99.06% IS401_aa1	100% CP025986_01760	IS3 ssgr IS51	1382067	1382390	324
RSCM	CP025986_01190	98.68% IS401_aa2	100% CP025986_01761	IS3 ssgr IS51	1382387	1383301	915
RSCM	CP025986_01366	81.52% ISAisp2_aa1	100% CP025986_01188	IS3 ssgr IS51	1621349	1621627	279
RSCM	CP025986_01367	89.34% ISAisp2_aa2	100% CP025986_01187	IS3 ssgr IS51	1621624	1622499	876
RSCM	CP025986_01760	99.06% IS401_aa1	100% CP025986_01189	IS3 ssgr IS51	2090234	2090557	324
RSCM	CP025986_01761	98.68% IS401_aa2	100% CP025986_01190	IS3 ssgr IS51	2090554	2091468	915
RSCM	CP025986_00573	61.68% ISCro3_aa1	100% CP025986_01139	IS4	685646	686974	1.329
RSCM	CP025986_00663	61.68% ISCro3_aa1	100% CP025986_01139	IS4	789881	788553	1.329
RSCM	CP025986_00776	61.68% ISCro3_aa1	100% CP025986_01139	IS4	91629	914962	1.329
RSCM	CP025986_00978	61.68% ISCro3_aa1	100% CP025986_01139	IS4	1138877	1137549	1.329
RSCM	CP025986_01139	61.68% ISCro3_aa1	100% CP025986_00978	IS4	1325345	1324017	1.329
RSCM	CP025986_00067	98.54% ISRso1_aa1	99.63% CP025986_01808	IS5	96715	95891	825
RSCM	CP025986_00100	98.66% ISRso1_aa1	99.11% CP025986_01808	IS5	13637	135693	678
RSCM	CP025986_01023	98.90% ISRso1_aa1	100% CP025986_01808	IS5	1182709	1181885	825
RSCM	CP025986_01176	98.90% ISRso1_aa1	100% CP025986_01808	IS5	1367731	1366907	825
RSCM	CP025986_01306	98.90% ISRso1_aa1	100% CP025986_01808	IS5	1530837	1530013	825
RSCM	CP025986_01335	98.90% ISRso1_aa1	100% CP025986_01808	IS5	1561237	1562061	825

RSCM	CP025986_01564	98.84% ISRso1_aa1	100% CP025986_00067	IS5	1848221	1848742	522
RSCM	CP025986_01576	96.96% ISRso1_aa1	96.96% CP025986_01564	IS5	1857798	1857998	201
RSCM	CP025986_01621	98.90% ISRso1_aa1	100% CP025986_01808	IS5	1901652	1902476	825
RSCM	CP025986_01808	98.90% ISRso1_aa1	100% CP025986_01621	IS5	2144887	2144063	825
RSCM	CP025986_00370	86.06% IS1421_aa1	100% CP025986_01758	IS5 ssgr IS427	456999	457406	408
RSCM	CP025986_00376	100% IS1421_aa1	100% CP025986_01336	IS5 ssgr IS427	462334	46193	405
RSCM	CP025986_00479	86.06% IS1421_aa1	100% CP025986_01758	IS5 ssgr IS427	585107	585514	408
RSCM	CP025986_00913	86.06% IS1421_aa1	100% CP025986_01758	IS5 ssgr IS427	1060882	1060475	408
RSCM	CP025986_01336	100% IS1421_aa1	100% CP025986_00376	IS5 ssgr IS427	1562889	1562485	405
RSCM	CP025986_01385	86.06% IS1421_aa1	100% CP025986_01758	IS5 ssgr IS427	1638920	1639327	408
RSCM	CP025986_01398	87.15% IS1421_aa1	100% CP025986_01758	IS5 ssgr IS427	1649800	1649429	372
RSCM	CP025986_01406	86.06% IS1421_aa1	100% CP025986_01758	IS5 ssgr IS427	1655778	1656185	408
RSCM	CP025986_01758	86.06% IS1421_aa1	100% CP025986_01406	IS5 ssgr IS427	2087881	2087474	408
RSCM	CP025986_00076	99.37% IS1405_aa1	100% CP025986_01656	IS5 ssgr IS5	110849	111814	966
RSCM	CP025986_00122	91.76% IS1021_aa1	100% CP025986_01806	IS5 ssgr IS5	160963	161949	987
RSCM	CP025986_00138	91.76% IS1021_aa1	100% CP025986_01806	IS5 ssgr IS5	192657	191671	987
RSCM	CP025986_00218	99.37% IS1405_aa1	100% CP025986_01656	IS5 ssgr IS5	275239	276204	966
RSCM	CP025986_00260	100% IS1405_aa1	100% CP025986_01656	IS5 ssgr IS5	323947	323672	276
RSCM	CP025986_00304	91.76% IS1021_aa1	100% CP025986_01806	IS5 ssgr IS5	368539	367553	987
RSCM	CP025986_00374	99.37% IS1405_aa1	100% CP025986_01656	IS5 ssgr IS5	459559	458594	966
RSCM	CP025986_00424	99.37% IS1405_aa1	100% CP025986_01656	IS5 ssgr IS5	518757	517792	966
RSCM	CP025986_00454	91.76% IS1021_aa1	100% CP025986_01806	IS5 ssgr IS5	548052	549038	987
RSCM	CP025986_00500	91.76% IS1021_aa1	100% CP025986_01806	IS5 ssgr IS5	610012	609026	987
RSCM	CP025986_00572	99.37% IS1405_aa1	100% CP025986_01656	IS5 ssgr IS5	685485	68452	966
RSCM	CP025986_00656	99.37% IS1405_aa1	100% CP025986_01656	IS5 ssgr IS5	780827	781792	966
RSCM	CP025986_00688	99.37% IS1405_aa1	100% CP025986_01656	IS5 ssgr IS5	829836	828871	966
RSCM	CP025986_00709	99.37% IS1405_aa1	100% CP025986_01656	IS5 ssgr IS5	84869	847725	966
RSCM	CP025986_00715	91.76% IS1021_aa1	100% CP025986_01806	IS5 ssgr IS5	854777	853791	987
RSCM	CP025986_00980	99.37% IS1405_aa1	100% CP025986_01656	IS5 ssgr IS5	1140087	1139122	966
RSCM	CP025986_01140	91.76% IS1021_aa1	100% CP025986_01806	IS5 ssgr IS5	1326484	1325498	987
RSCM	CP025986_01193	99.37% IS1405_aa1	100% CP025986_01656	IS5 ssgr IS5	1385556	1384591	966
RSCM	CP025986_01228	91.76% IS1021_aa1	100% CP025986_01806	IS5 ssgr IS5	1430591	1429605	987
RSCM	CP025986_01304	91.76% IS1021_aa1	100% CP025986_01806	IS5 ssgr IS5	1528158	1527172	987
RSCM	CP025986_01338	91.76% IS1021_aa1	100% CP025986_01806	IS5 ssgr IS5	1580039	1579053	987
RSCM	CP025986_01345	99.37% IS1405_aa1	100% CP025986_01656	IS5 ssgr IS5	1588387	1587422	966
RSCM	CP025986_01346	99.37% IS1405_aa1	100% CP025986_01656	IS5 ssgr IS5	1588714	1589679	966
RSCM	CP025986_01349	91.76% IS1021_aa1	100% CP025986_01806	IS5 ssgr IS5	1594375	1595361	987
RSCM	CP025986_01418	99.37% IS1405_aa1	100% CP025986_01656	IS5 ssgr IS5	1667735	1668700	966
RSCM	CP025986_01491	91.76% IS1021_aa1	100% CP025986_01806	IS5 ssgr IS5	1766292	1765306	987
RSCM	CP025986_01503	91.76% IS1021_aa1	100% CP025986_01806	IS5 ssgr IS5	1777674	1778660	987
RSCM	CP025986_01592	99.37% IS1405_aa1	100% CP025986_01656	IS5 ssgr IS5	1871016	1870051	966
RSCM	CP025986_01656	99.37% IS1405_aa1	100% CP025986_01592	IS5 ssgr IS5	1959948	1958983	966
RSCM	CP025986_01806	91.76% IS1021_aa1	100% CP025986_01503	IS5 ssgr IS5	2141446	2142432	987

RSCM	CP025986_00760	80.88% ISRso5_aa1	100% CP025986_01492	IS630	901128	90221	1.083
RSCM	CP025986_01128	80.88% ISRso5_aa1	100% CP025986_01492	IS630	1301715	1302797	1.083
RSCM	CP025986_01492	80.88% ISRso5_aa1	100% CP025986_01128	IS630	1766456	1767538	1.083
RSCM	CP025986_00261	58.21% ISRm2_aa1	100% CP025986_01559	IS66	324065	324529	465
RSCM	CP025986_00262	75% ISBmu30_aa1	100% CP025986_01558	IS66	324529	324879	351
RSCM	CP025986_00263	58.13% ISAeh1_aa2	100% CP025986_01557	IS66	324911	326482	1.572
RSCM	CP025986_00290	58.21% ISRm2_aa1	100% CP025986_01559	IS66	35149	351954	465
RSCM	CP025986_00291	75% ISBmu30_aa1	100% CP025986_01558	IS66	351954	352304	351
RSCM	CP025986_00292	58.13% ISAeh1_aa2	100% CP025986_01557	IS66	352336	353907	1.572
RSCM	CP025986_01119	58.21% ISRm2_aa1	100% CP025986_01559	IS66	1294565	1295029	465
RSCM	CP025986_01120	75% ISBmu30_aa1	100% CP025986_01558	IS66	1295029	1295379	351
RSCM	CP025986_01121	58.13% ISAeh1_aa2	100% CP025986_01557	IS66	1295411	1296982	1.572
RSCM	CP025986_01413	58.21% ISRm2_aa1	100% CP025986_01559	IS66	1661394	1661858	465
RSCM	CP025986_01414	75% ISBmu30_aa1	100% CP025986_01558	IS66	1661858	1662208	351
RSCM	CP025986_01415	58.13% ISAeh1_aa2	100% CP025986_01557	IS66	1662240	1663811	1.572
RSCM	CP025986_01557	58.13% ISAeh1_aa2	100% CP025986_01415	IS66	1843645	1842074	1.572
RSCM	CP025986_01558	75% ISBmu30_aa1	100% CP025986_01414	IS66	1844027	1843677	351
RSCM	CP025986_01559	58.21% ISRm2_aa1	100% CP025986_01413	IS66	1844491	1844027	465
RSCM	CP025986_00066	100% ISRso17_aa1	100% CP025986_01807	IS701	95839	94478	1.362
RSCM	CP025986_00078	100% ISRso17_aa1	100% CP025986_01807	IS701	111992	113353	1.362
RSCM	CP025986_00220	100% ISRso17_aa1	100% CP025986_01807	IS701	276382	277743	1.362
RSCM	CP025986_00714	100% ISRso17_aa1	100% CP025986_01229	IS701	853312	853686	375
RSCM	CP025986_00716	100% ISRso17_aa1	100% CP025986_01504	IS701	854937	855884	948
RSCM	CP025986_01229	100% ISRso17_aa1	100% CP025986_01807	IS701	1431535	1430666	870
RSCM	CP025986_01305	100% ISRso17_aa1	100% CP025986_01807	IS701	1528553	1529914	1.362
RSCM	CP025986_01334	100% ISRso17_aa1	100% CP025986_01807	IS701	1561158	1559797	1.362
RSCM	CP025986_01504	100% ISRso17_aa1	100% CP025986_00716	IS701	1778870	1779817	948
RSCM	CP025986_01563	100% ISRso17_aa1	100% CP025986_01807	IS701	1846277	1847638	1.362
RSCM	CP025986_01620	100% ISRso17_aa1	100% CP025986_01807	IS701	1900165	1901526	1.362
RSCM	CP025986_01807	100% ISRso17_aa1	100% CP025986_01620	IS701	2144011	2142650	1.362
RSCM	CP025986_00780	43.47% ISTha3_aa2	No hit	IS91	920285	919179	1.107
RSCM	CP025986_01184	43.40% ISWz1_aa1	78.46% CP025986_01129	IS91	1376304	1378004	1.701
RSCM	CP025986_01191	72.61% ISKpn21_aa1	No hit	ISNCY ssgr IS1202	1384344	1383298	1.047
RSCM	CP025986_01130	65.71% ISPa43_aa2	46.03% CP025986_01181	Tn3	1304669	1307653	2.985
RSCM	CP025986_01179	82.07% ISPa38_aa1	94.39% CP025986_01182	Tn3	1370128	1369778	351
RSCM	CP025986_01181	94.62% TnShfr1_aa4	45.52% CP025986_01130	Tn3	1374630	1371667	2.964
RSCM	CP025986_01182	87.56% ISPa38_aa1	94.39% CP025986_01179	Tn3	1374787	1375344	558
T60	CP022769_00426	88.75% ISBcen4_aa1	100% CP022769_00539	IS110 ssgr IS1111	545948	546967	1.02
T60	CP022769_00477	88.75% ISBcen4_aa1	100% CP022769_00539	IS110 ssgr IS1111	616743	615724	1.02
T60	CP022769_00539	88.75% ISBcen4_aa1	100% CP022769_00426	IS110 ssgr IS1111	684334	685353	1.02
T60	CP022769_01573	61.67% ISMno14_aa1	100% CP022769_01578	IS110 ssgr IS1111	2021824	2022837	1.014
T60	CP022769_01578	61.67% ISMno14_aa1	100% CP022769_01573	IS110 ssgr IS1111	2026416	2025403	1.014

T60	CP022769_01306	83.75% ISBusp4_aa1	No hit	IS1182	1667911	1666466	1.446
T60	CP022769_00505	50.44% ISLsp2_aa1	No hit	IS1595 ssgr ISPna2	650248	651525	1.278
T60	CP022769_00476	100% ISRso10_aa2	93.41% CP022769_00540	IS3 ssgr IS2	615347	614499	849
T60	CP022769_00478	97.87% ISRso10_aa1	96.82% CP022769_00538	IS3 ssgr IS2	617098	616718	381
T60	CP022769_00538	95.74% ISRso10_aa1	96.82% CP022769_00478	IS3 ssgr IS2	683979	684359	381
T60	CP022769_00540	93.41% ISRso10_aa2	93.41% CP022769_00476	IS3 ssgr IS2	685847	686578	732
T60	CP022769_01332	78.68% ISRso20_aa1	No hit	IS3 ssgr IS3	1716646	1716846	201
T60	CP022769_01574	93.82% ISRso16_aa2	45.66% CP022769_00476	IS3 ssgr IS407	2023184	2023723	540
T60	CP022769_01117	81.52% ISAisp2_aa1	97.82% CP022769_01292	IS3 ssgr IS51	1428122	1428400	279
T60	CP022769_01118	89.34% ISAisp2_aa2	98.28% CP022769_01291	IS3 ssgr IS51	1428397	1429272	876
T60	CP022769_01291	89.34% ISAisp2_aa2	98.28% CP022769_01118	IS3 ssgr IS51	1650074	1649199	876
T60	CP022769_01292	81.52% ISAisp2_aa1	97.82% CP022769_01117	IS3 ssgr IS51	1650349	1650071	279
T60	CP022769_00350	61.44% ISCro3_aa1	No hit	IS4	458284	456956	1.329
T60	CP022769_00703	78.03% ISAzo5_aa1	100% CP022769_00742	IS4 ssgr IS50	910436	909111	1.326
T60	CP022769_00742	78.03% ISAzo5_aa1	100% CP022769_00703	IS4 ssgr IS50	962895	96157	1.326
T60	CP022769_00506	82.75% ISBcen26_aa1	No hit	IS481	65202	65167	351
T60	CP022769_00628	98.54% ISRso1_aa1	99.27% CP022769_01104	IS5	82378	825213	1.434
T60	CP022769_00722	97.81% ISRso1_aa1	100% CP022769_01311	IS5	936271	935447	825
T60	CP022769_00977	98.66% ISRso1_aa1	100% CP022769_01311	IS5	1247515	1248192	678
T60	CP022769_01104	98.54% ISRso1_aa1	98.54% CP022769_01311	IS5	1418790	1417966	825
T60	CP022769_01116	85.56% ISRso1_aa1	86.59% CP022769_01311	IS5	1427698	1428090	393
T60	CP022769_01311	97.81% ISRso1_aa1	100% CP022769_00722	IS5	1674699	1675523	825
T60	CP022769_00724	99.25% IS1421_aa1	No hit	IS5 ssgr IS427	938732	938328	405
T60	CP022769_00042	94.51% IS1021_aa1	71.80% CP022769_00082	IS5 ssgr IS5	48328	47342	987
T60	CP022769_00043	99.06% IS1405_aa1	100% CP022769_01229	IS5 ssgr IS5	48486	49451	966
T60	CP022769_00082	99.37% IS1405_aa1	99.68% CP022769_01456	IS5 ssgr IS5	100541	99576	966
T60	CP022769_00421	99.06% IS1405_aa1	100% CP022769_01229	IS5 ssgr IS5	542742	543707	966
T60	CP022769_00455	99.06% IS1405_aa1	100% CP022769_01229	IS5 ssgr IS5	588669	589634	966
T60	CP022769_00544	99.06% IS1405_aa1	100% CP022769_01229	IS5 ssgr IS5	689213	688248	966
T60	CP022769_00552	99.06% IS1405_aa1	100% CP022769_01229	IS5 ssgr IS5	693566	692601	966
T60	CP022769_00679	99.06% IS1405_aa1	100% CP022769_01229	IS5 ssgr IS5	883502	884467	966
T60	CP022769_00816	99.06% IS1405_aa1	100% CP022769_01229	IS5 ssgr IS5	1061258	1060293	966
T60	CP022769_00884	99.06% IS1405_aa1	100% CP022769_01229	IS5 ssgr IS5	1142377	1141412	966
T60	CP022769_00890	99.06% IS1405_aa1	100% CP022769_01229	IS5 ssgr IS5	1148235	1149200	966
T60	CP022769_01051	99.06% IS1405_aa1	100% CP022769_01229	IS5 ssgr IS5	1341271	1342236	966
T60	CP022769_01132	98.91% IS1405_aa1	100% CP022769_00082	IS5 ssgr IS5	1438621	1439217	597
T60	CP022769_01133	100% IS1405_aa1	100% CP022769_01456	IS5 ssgr IS5	1439309	1439584	276
T60	CP022769_01229	99.06% IS1405_aa1	100% CP022769_01051	IS5 ssgr IS5	1574825	1575790	966
T60	CP022769_01456	99.06% IS1405_aa1	99.68% CP022769_00082	IS5 ssgr IS5	1865358	1864393	966
T60	CP022769_00163	99.68% IS1420_aa1	100% CP022769_01307	IS5 ssgr IS903	209345	210301	957
T60	CP022769_00458	99.68% IS1420_aa1	100% CP022769_01307	IS5 ssgr IS903	592091	591135	957
T60	CP022769_00471	99.68% IS1420_aa1	100% CP022769_01307	IS5 ssgr IS903	607208	608164	957
T60	CP022769_01307	99.68% IS1420_aa1	100% CP022769_00471	IS5 ssgr IS903	1669345	1668389	957

T60	CP022769_00508	79.66% ISCARN39_aa3	No hit	IS630	654389	653853	537
T60	CP022769_00872	43.40% ISWz1_aa1	44.13% CP022769_01625	IS91	1128491	1130191	1.701
T60	CP022769_00888	62.5% ISAba32_aa1	70% CP022769_00889	ISNCY ssgr IS1202	1145899	1145684	216
T60	CP022769_00889	72.27% ISKpn21_aa1	70% CP022769_00888	ISNCY ssgr IS1202	1147517	1146042	1.476
T60	CP022769_00461	51.46% ISMpo10_aa3	No hit	Tn3	595547	59692	1.374
SL3882	CP022779_00424	88.75% ISBcen4_aa1	100% CP022779_00536	IS110 ssgr IS1111	545965	546984	1.02
SL3882	CP022779_00475	88.75% ISBcen4_aa1	100% CP022779_00536	IS110 ssgr IS1111	616748	615729	1.02
SL3882	CP022779_00536	88.75% ISBcen4_aa1	100% CP022779_00424	IS110 ssgr IS1111	68434	685359	1.02
SL3882	CP022779_01576	61.67% ISMno14_aa1	100% CP022779_01581	IS110 ssgr IS1111	2033292	2034305	1.014
SL3882	CP022779_01581	61.67% ISMno14_aa1	100% CP022779_01576	IS110 ssgr IS1111	2037884	2036871	1.014
SL3882	CP022779_01305	83.75% ISBusp4_aa1	No hit	IS1182	1679391	1677946	1.446
SL3882	CP022779_00502	50.44% ISLsp2_aa1	No hit	IS1595 ssgr ISPna2	650254	651531	1.278
SL3882	CP022779_00474	100% ISRso10_aa2	93.41% CP022779_00537	IS3 ssgr IS2	615352	614504	849
SL3882	CP022779_00476	97.87% ISRso10_aa1	96.82% CP022779_00535	IS3 ssgr IS2	617103	616723	381
SL3882	CP022779_00535	95.74% ISRso10_aa1	96.82% CP022779_00476	IS3 ssgr IS2	683985	684365	381
SL3882	CP022779_00537	93.41% ISRso10_aa2	93.41% CP022779_00474	IS3 ssgr IS2	685853	686584	732
SL3882	CP022779_01331	78.68% ISRso20_aa1	No hit	IS3 ssgr IS3	1728126	1728326	201
SL3882	CP022779_01577	93.82% ISRso16_aa2	45.66% CP022779_00474	IS3 ssgr IS407	2034652	2035191	540
SL3882	CP022779_01118	81.52% ISAisp2_aa1	97.82% CP022779_01291	IS3 ssgr IS51	1439586	1439864	279
SL3882	CP022779_01119	89.34% ISAisp2_aa2	98.28% CP022779_01290	IS3 ssgr IS51	1439861	1440736	876
SL3882	CP022779_01290	89.34% ISAisp2_aa2	98.28% CP022779_01119	IS3 ssgr IS51	1661554	1660679	876
SL3882	CP022779_01291	81.52% ISAisp2_aa1	97.82% CP022779_01118	IS3 ssgr IS51	1661829	1661551	279
SL3882	CP022779_00348	61.44% ISCro3_aa1	No hit	IS4	458302	456974	1.329
SL3882	CP022779_00700	78.03% ISAzo5_aa1	100% CP022779_00739	IS4 ssgr IS50	910425	9091	1.326
SL3882	CP022779_00739	78.03% ISAzo5_aa1	100% CP022779_00700	IS4 ssgr IS50	962884	961559	1.326
SL3882	CP022779_00503	82.75% ISBcen26_aa1	No hit	IS481	652026	651676	351
SL3882	CP022779_00625	98.54% ISRso1_aa1	99.27% CP022779_01105	IS5	823769	825202	1.434
SL3882	CP022779_00719	97.81% ISRso1_aa1	100% CP022779_01310	IS5	93626	935436	825
SL3882	CP022779_00974	98.66% ISRso1_aa1	100% CP022779_01310	IS5	1247504	1248181	678
SL3882	CP022779_01105	98.54% ISRso1_aa1	98.54% CP022779_01310	IS5	1430254	1429430	825
SL3882	CP022779_01117	85.56% ISRso1_aa1	86.59% CP022779_01310	IS5	1439162	1439554	393
SL3882	CP022779_01310	97.81% ISRso1_aa1	100% CP022779_00719	IS5	1686179	1687003	825
SL3882	CP022779_00721	99.25% IS1421_aa1	No hit	IS5 ssgr IS427	938721	938317	405
SL3882	CP022779_00042	94.51% IS1021_aa1	71.80% CP022779_01130	IS5 ssgr IS5	48328	47342	987
SL3882	CP022779_00043	99.06% IS1405_aa1	100% CP022779_01228	IS5 ssgr IS5	48486	49451	966
SL3882	CP022779_00082	99.37% IS1405_aa1	100% CP022779_01130	IS5 ssgr IS5	100559	99594	966
SL3882	CP022779_00419	99.06% IS1405_aa1	100% CP022779_01228	IS5 ssgr IS5	542759	543724	966
SL3882	CP022779_00453	99.06% IS1405_aa1	100% CP022779_01228	IS5 ssgr IS5	588674	589639	966
SL3882	CP022779_00541	99.06% IS1405_aa1	100% CP022779_01228	IS5 ssgr IS5	689219	688254	966
SL3882	CP022779_00549	99.06% IS1405_aa1	100% CP022779_01228	IS5 ssgr IS5	693572	692607	966
SL3882	CP022779_00676	99.06% IS1405_aa1	100% CP022779_01228	IS5 ssgr IS5	883491	884456	966
SL3882	CP022779_00813	99.06% IS1405_aa1	100% CP022779_01228	IS5 ssgr IS5	1061247	1060282	966

SL3882	CP022779_00881	99.06% IS1405_aa1	100% CP022779_01228	IS5 ssgr IS5	1142366	1141401	966
SL3882	CP022779_00887	99.06% IS1405_aa1	100% CP022779_01228	IS5 ssgr IS5	1148224	1149189	966
SL3882	CP022779_01048	99.06% IS1405_aa1	100% CP022779_01228	IS5 ssgr IS5	1341260	1342225	966
SL3882	CP022779_01130	99.37% IS1405_aa1	100% CP022779_00082	IS5 ssgr IS5	1450097	1451062	966
SL3882	CP022779_01228	99.06% IS1405_aa1	100% CP022779_01048	IS5 ssgr IS5	1586305	1587270	966
SL3882	CP022779_01455	99.06% IS1405_aa1	99.68% CP022779_01130	IS5 ssgr IS5	1876838	1875873	966
SL3882	CP022779_00163	99.68% IS1420_aa1	100% CP022779_01306	IS5 ssgr IS903	209362	210318	957
SL3882	CP022779_00456	99.68% IS1420_aa1	100% CP022779_01306	IS5 ssgr IS903	592096	59114	957
SL3882	CP022779_00469	99.68% IS1420_aa1	100% CP022779_01306	IS5 ssgr IS903	607213	608169	957
SL3882	CP022779_01306	99.68% IS1420_aa1	100% CP022779_00469	IS5 ssgr IS903	1680825	1679869	957
SL3882	CP022779_00505	79.66% ISCARN39_aa3	No hit	IS630	654395	653859	537
SL3882	CP022779_00869	43.40% ISWz1_aa1	44.13% CP022779_01627	IS91	1128480	1130180	1.701
SL3882	CP022779_00885	62.5% ISAba32_aa1	70% CP022779_00886	ISNCY ssgr IS1202	1145888	1145673	216
SL3882	CP022779_00886	72.27% ISKpn21_aa1	70% CP022779_00885	ISNCY ssgr IS1202	1147506	1146031	1.476
SL3882	CP022779_00459	51.46% ISMpo10_aa3	No hit	Tn3	595552	596925	1.374
T42	CP022773_00436	88.75% ISBcen4_aa1	45.80% CP022773_01364	IS110 ssgr IS1111	556241	55726	1.02
T42	CP022773_01359	61.67% ISMno14_aa1	100% CP022773_01364	IS110 ssgr IS1111	1731457	1732470	1.014
T42	CP022773_01364	61.67% ISMno14_aa1	100% CP022773_01359	IS110 ssgr IS1111	1736049	1735036	1.014
T42	CP022773_01092	83.75% ISBusp4_aa1	No hit	IS1182	1377550	1376105	1.446
T42	CP022773_01116	78.68% ISRso20_aa1	No hit	IS3 ssgr IS3	1424270	1424470	201
T42	CP022773_01363	93.82% ISRso16_aa2	44.58% CP022773_00907	IS3 ssgr IS407	1734689	1734150	540
T42	CP022773_00906	81.52% ISAisp2_aa1	97.82% CP022773_01078	IS3 ssgr IS51	1137801	1138079	279
T42	CP022773_00907	89.34% ISAisp2_aa2	98.28% CP022773_01077	IS3 ssgr IS51	1138076	1138951	876
T42	CP022773_01077	89.34% ISAisp2_aa2	98.28% CP022773_00907	IS3 ssgr IS51	1359713	1358838	876
T42	CP022773_01078	81.52% ISAisp2_aa1	97.82% CP022773_00906	IS3 ssgr IS51	1359988	1359710	279
T42	CP022773_00002	61.68% ISCro3_aa1	99.77% CP022773_00363	IS4	3517	2189	1.329
T42	CP022773_00363	61.44% ISCro3_aa1	99.77% CP022773_00002	IS4	469758	46843	1.329
T42	CP022773_00516	97.81% ISRso1_aa1	98.54% CP022773_00893	IS5	655716	654892	825
T42	CP022773_00859	98.87% ISRso1_aa1	100% CP022773_00516	IS5	1089654	1090217	564
T42	CP022773_00893	98.54% ISRso1_aa1	98.54% CP022773_00516	IS5	1128469	1127645	825
T42	CP022773_00905	85.56% ISRso1_aa1	86.59% CP022773_00859	IS5	1137377	1137769	393
T42	CP022773_00227	99.25% IS1421_aa1	100% CP022773_01261	IS5 ssgr IS427	300573	300977	405
T42	CP022773_00518	99.25% IS1421_aa1	98.50% CP022773_01261	IS5 ssgr IS427	658176	657772	405
T42	CP022773_01261	99.25% IS1421_aa1	100% CP022773_00227	IS5 ssgr IS427	1592744	1592340	405
T42	CP022773_00031	99.06% IS1405_aa1	100% CP022773_01205	IS5 ssgr IS5	35897	34932	966
T42	CP022773_00043	94.51% IS1021_aa1	100% CP022773_00226	IS5 ssgr IS5	48315	49301	987
T42	CP022773_00046	99.06% IS1405_aa1	100% CP022773_01205	IS5 ssgr IS5	51108	52073	966
T42	CP022773_00081	94.51% IS1021_aa1	100% CP022773_00226	IS5 ssgr IS5	94012	93026	987
T42	CP022773_00086	99.37% IS1405_aa1	100% CP022773_01242	IS5 ssgr IS5	10508	104115	966
T42	CP022773_00167	94.51% IS1021_aa1	100% CP022773_00226	IS5 ssgr IS5	214751	213765	987
T42	CP022773_00169	94.51% IS1021_aa1	100% CP022773_00226	IS5 ssgr IS5	217688	216702	987
T42	CP022773_00226	94.51% IS1021_aa1	100% CP022773_00169	IS5 ssgr IS5	299403	300389	987

T42	CP022773_00254	99.06% IS1405_aa1	100% CP022773_01205	IS5 ssgr IS5	345491	344526	966
T42	CP022773_00294	99.06% IS1405_aa1	100% CP022773_01205	IS5 ssgr IS5	396422	397387	966
T42	CP022773_00466	99.06% IS1405_aa1	100% CP022773_01205	IS5 ssgr IS5	600078	601043	966
T42	CP022773_00474	99.06% IS1405_aa1	100% CP022773_01205	IS5 ssgr IS5	604431	605396	966
T42	CP022773_00674	99.06% IS1405_aa1	100% CP022773_01205	IS5 ssgr IS5	857231	856266	966
T42	CP022773_00680	99.06% IS1405_aa1	100% CP022773_01205	IS5 ssgr IS5	86309	864055	966
T42	CP022773_00840	99.06% IS1405_aa1	100% CP022773_01205	IS5 ssgr IS5	1055248	1056213	966
T42	CP022773_00918	99.37% IS1405_aa1	100% CP022773_01242	IS5 ssgr IS5	1148312	1149277	966
T42	CP022773_01015	99.06% IS1405_aa1	100% CP022773_01205	IS5 ssgr IS5	1284473	1285438	966
T42	CP022773_01205	99.06% IS1405_aa1	100% CP022773_01015	IS5 ssgr IS5	1535432	1536397	966
T42	CP022773_01242	99.37% IS1405_aa1	100% CP022773_00918	IS5 ssgr IS5	1574125	1573160	966
T42	CP022773_00097	100% IS1420_aa1	100% CP022773_00463	IS5 ssgr IS903	116169	117125	957
T42	CP022773_00463	100% IS1420_aa1	100% CP022773_00097	IS5 ssgr IS903	598088	599044	957
T42	CP022773_00662	43.40% ISWz1_aa1	44.13% CP022773_01410	IS91	843345	845045	1.701
T42	CP022773_00678	62.5% ISAba32_aa1	70% CP022773_00679	ISNCY ssgr IS1202	860753	860538	216
T42	CP022773_00679	72.27% ISKpn21_aa1	70% CP022773_00678	ISNCY ssgr IS1202	862371	860896	1.476
SL3300	IS_5d5b941e_00456	88.75% ISBcen4_aa1	100% IS_5d5b941e_00499	IS110 ssgr IS1111	590607	591626	1.02
SL3300	IS_5d5b941e_00499	88.75% ISBcen4_aa1	100% IS_5d5b941e_00456	IS110 ssgr IS1111	650071	65109	1.02
SL3300	IS_5d5b941e_01532	61.67% ISMno14_aa1	100% IS_5d5b941e_01537	IS110 ssgr IS1111	1984110	1985123	1.014
SL3300	IS_5d5b941e_01537	61.67% ISMno14_aa1	100% IS_5d5b941e_01532	IS110 ssgr IS1111	1988702	1987689	1.014
SL3300	IS_5d5b941e_01256	83.75% ISBusp4_aa1	No hit	IS1182	1619956	1618511	1.446
SL3300	IS_5d5b941e_00498	95.74% ISRso10_aa1	No hit	IS3 ssgr IS2	649716	650096	381
SL3300	IS_5d5b941e_00500	93.41% ISRso10_aa2	44.70% IS_5d5b941e_01536	IS3 ssgr IS2	651583	652314	732
SL3300	IS_5d5b941e_01286	78.68% ISRso20_aa1	No hit	IS3 ssgr IS3	1672690	1672890	201
SL3300	IS_5d5b941e_01536	93.82% ISRso16_aa2	44.70% IS_5d5b941e_00500	IS3 ssgr IS407	1987342	1986803	540
SL3300	IS_5d5b941e_01069	81.52% ISAisp2_aa1	97.82% IS_5d5b941e_01242	IS3 ssgr IS51	1379576	1379854	279
SL3300	IS_5d5b941e_01070	89.34% ISAisp2_aa2	98.28% IS_5d5b941e_01241	IS3 ssgr IS51	1379851	1380726	876
SL3300	IS_5d5b941e_01241	89.34% ISAisp2_aa2	98.28% IS_5d5b941e_01070	IS3 ssgr IS51	1602119	1601244	876
SL3300	IS_5d5b941e_01242	81.52% ISAisp2_aa1	97.82% IS_5d5b941e_01069	IS3 ssgr IS51	1602394	1602116	279
SL3300	IS_5d5b941e_00351	61.44% ISCro3_aa1	No hit	IS4	461903	460575	1.329
SL3300	IS_5d5b941e_01453	78.03% ISAzo5_aa1	100% IS_5d5b941e_01527	IS4 ssgr IS50	1875409	1874084	1.326
SL3300	IS_5d5b941e_01527	78.03% ISAzo5_aa1	100% IS_5d5b941e_01453	IS4 ssgr IS50	1976665	1977990	1.326
SL3300	IS_5d5b941e_00398	40.42% ISGur11_aa1	No hit	IS481	523838	521871	1.968
SL3300	IS_5d5b941e_00587	98.54% ISRso1_aa1	99.27% IS_5d5b941e_01054	IS5	787566	788999	1.434
SL3300	IS_5d5b941e_01054	98.54% ISRso1_aa1	99.27% IS_5d5b941e_00587	IS5	1369032	1368208	825
SL3300	IS_5d5b941e_01068	85.56% ISRso1_aa1	86.59% IS_5d5b941e_01054	IS5	1379152	1379544	393
SL3300	IS_5d5b941e_00694	99.25% IS1421_aa1	100% IS_5d5b941e_01432	IS5 ssgr IS427	914584	914988	405
SL3300	IS_5d5b941e_01240	99.25% IS1421_aa1	100% IS_5d5b941e_01432	IS5 ssgr IS427	1600992	1600588	405
SL3300	IS_5d5b941e_01432	99.25% IS1421_aa1	100% IS_5d5b941e_01240	IS5 ssgr IS427	1844917	1844513	405
SL3300	IS_5d5b941e_00030	99.06% IS1405_aa1	100% IS_5d5b941e_01178	IS5 ssgr IS5	34454	33489	966
SL3300	IS_5d5b941e_00044	99.06% IS1405_aa1	100% IS_5d5b941e_01178	IS5 ssgr IS5	48453	49418	966
SL3300	IS_5d5b941e_00083	99.37% IS1405_aa1	100% IS_5d5b941e_01411	IS5 ssgr IS5	100436	99471	966

SL3300	IS_5d5b941e_00104	94.51% IS1021_aa1	100% IS_5d5b941e_01431	IS5 ssgr IS5	128052	127066	987
SL3300	IS_5d5b941e_00156	94.51% IS1021_aa1	100% IS_5d5b941e_01431	IS5 ssgr IS5	198025	199011	987
SL3300	IS_5d5b941e_00166	94.51% IS1021_aa1	100% IS_5d5b941e_01431	IS5 ssgr IS5	214061	213075	987
SL3300	IS_5d5b941e_00167	94.51% IS1021_aa1	100% IS_5d5b941e_01431	IS5 ssgr IS5	215009	215995	987
SL3300	IS_5d5b941e_00478	94.51% IS1021_aa1	100% IS_5d5b941e_01431	IS5 ssgr IS5	619106	61812	987
SL3300	IS_5d5b941e_00479	94.51% IS1021_aa1	100% IS_5d5b941e_01431	IS5 ssgr IS5	620318	619332	987
SL3300	IS_5d5b941e_00485	94.51% IS1021_aa1	100% IS_5d5b941e_01431	IS5 ssgr IS5	634489	635475	987
SL3300	IS_5d5b941e_00504	99.06% IS1405_aa1	100% IS_5d5b941e_01178	IS5 ssgr IS5	654949	653984	966
SL3300	IS_5d5b941e_00511	94.51% IS1021_aa1	100% IS_5d5b941e_01431	IS5 ssgr IS5	658241	659227	987
SL3300	IS_5d5b941e_00640	99.06% IS1405_aa1	100% IS_5d5b941e_01178	IS5 ssgr IS5	847292	848257	966
SL3300	IS_5d5b941e_00835	99.06% IS1405_aa1	100% IS_5d5b941e_01178	IS5 ssgr IS5	1093501	1092536	966
SL3300	IS_5d5b941e_00841	99.06% IS1405_aa1	100% IS_5d5b941e_01178	IS5 ssgr IS5	1099360	1100325	966
SL3300	IS_5d5b941e_01000	99.06% IS1405_aa1	100% IS_5d5b941e_01178	IS5 ssgr IS5	1291525	1292490	966
SL3300	IS_5d5b941e_01063	94.51% IS1021_aa1	100% IS_5d5b941e_01431	IS5 ssgr IS5	1375626	1376612	987
SL3300	IS_5d5b941e_01081	99.37% IS1405_aa1	100% IS_5d5b941e_01411	IS5 ssgr IS5	1390087	1391052	966
SL3300	IS_5d5b941e_01178	99.06% IS1405_aa1	100% IS_5d5b941e_01000	IS5 ssgr IS5	1526012	1526977	966
SL3300	IS_5d5b941e_01257	99.37% IS1405_aa1	100% IS_5d5b941e_01411	IS5 ssgr IS5	1620035	1621000	966
SL3300	IS_5d5b941e_01276	94.51% IS1021_aa1	100% IS_5d5b941e_01431	IS5 ssgr IS5	1663013	1662027	987
SL3300	IS_5d5b941e_01277	94.51% IS1021_aa1	100% IS_5d5b941e_01431	IS5 ssgr IS5	1664225	1663239	987
SL3300	IS_5d5b941e_01278	94.51% IS1021_aa1	100% IS_5d5b941e_01431	IS5 ssgr IS5	1664745	1665731	987
SL3300	IS_5d5b941e_01279	94.63% IS1021_aa1	100% IS_5d5b941e_01431	IS5 ssgr IS5	1666649	1665855	795
SL3300	IS_5d5b941e_01298	94.51% IS1021_aa1	100% IS_5d5b941e_01431	IS5 ssgr IS5	1682323	1683309	987
SL3300	IS_5d5b941e_01411	99.37% IS1405_aa1	100% IS_5d5b941e_01257	IS5 ssgr IS5	1822599	1821634	966
SL3300	IS_5d5b941e_01431	94.51% IS1021_aa1	100% IS_5d5b941e_01298	IS5 ssgr IS5	1844027	1843041	987
SL3300	IS_5d5b941e_00823	43.40% ISWz1_aa1	44.13% IS_5d5b941e_01583	IS91	1079903	1081603	1.701
SL3300	IS_5d5b941e_00406	50.23% ISKpn25_aa3	No hit	ISL3	532903	529943	2.961
SL3300	IS_5d5b941e_00407	65.04% ISKpn25_aa2	No hit	ISL3	534184	532913	1.272
SL3300	IS_5d5b941e_00409	50.25% ISKpn25_aa1	No hit	ISL3	538379	536025	2.355
SL3300	IS_5d5b941e_00836	72.05% ISKpn21_aa1	70% IS_5d5b941e_00837	ISNCY ssgr IS1202	1094220	1095695	1.476
SL3300	IS_5d5b941e_00837	62.5% ISAba32_aa1	70% IS_5d5b941e_00836	ISNCY ssgr IS1202	1095838	1096053	216
SL3822	CP022781_01049	88.75% ISBcen4_aa1	100% CP022781_01213	IS110 ssgr IS1111	1350513	1349494	1.02
SL3822	CP022781_01066	88.75% ISBcen4_aa1	100% CP022781_01213	IS110 ssgr IS1111	1364763	1365782	1.02
SL3822	CP022781_01162	88.75% ISBcen4_aa1	100% CP022781_01213	IS110 ssgr IS1111	1482184	1483203	1.02
SL3822	CP022781_01213	88.75% ISBcen4_aa1	100% CP022781_01162	IS110 ssgr IS1111	1553139	1552120	1.02
SL3822	CP022781_01628	61.67% ISMno14_aa1	100% CP022781_01633	IS110 ssgr IS1111	2085153	2086166	1.014
SL3822	CP022781_01633	61.67% ISMno14_aa1	100% CP022781_01628	IS110 ssgr IS1111	2089745	2088732	1.014
SL3822	CP022781_00284	83.75% ISBusp4_aa1	No hit	IS1182	373412	374857	1.446
SL3822	CP022781_01071	50.44% ISLsp2_aa1	99.52% CP022781_01136	IS1595 ssgr ISPna2	1371333	1370056	1.278
SL3822	CP022781_01136	50.44% ISLsp2_aa1	99.52% CP022781_01071	IS1595 ssgr ISPna2	1450483	1449206	1.278
SL3822	CP022781_01048	93.41% ISRso10_aa2	93.41% CP022781_01163	IS3 ssgr IS2	1349001	1348270	732
SL3822	CP022781_01050	97.87% ISRso10_aa1	98.41% CP022781_01161	IS3 ssgr IS2	1350868	1350488	381
SL3822	CP022781_01065	97.87% ISRso10_aa1	100% CP022781_01161	IS3 ssgr IS2	1364408	1364788	381

SL3822	CP022781_01161	97.87% ISRso10_aa1	100% CP022781_01065	IS3 ssgr IS2	1481829	1482209	381
SL3822	CP022781_01163	100% ISRso10_aa2	93.41% CP022781_01048	IS3 ssgr IS2	1483580	1484428	849
SL3822	CP022781_00257	78.68% ISRso20_aa1	No hit	IS3 ssgr IS3	324673	324473	201
SL3822	CP022781_01632	93.82% ISRso16_aa2	45.66% CP022781_01163	IS3 ssgr IS407	2088385	2087846	540
SL3822	CP022781_00298	81.52% ISAisp2_aa1	97.82% CP022781_00474	IS3 ssgr IS51	390974	391252	279
SL3822	CP022781_00299	89.34% ISAisp2_aa2	98.28% CP022781_00473	IS3 ssgr IS51	391249	392124	876
SL3822	CP022781_00473	89.34% ISAisp2_aa2	98.28% CP022781_00299	IS3 ssgr IS51	615291	614416	876
SL3822	CP022781_00474	81.52% ISAisp2_aa1	97.82% CP022781_00298	IS3 ssgr IS51	615566	615288	279
SL3822	CP022781_01287	61.44% ISCro3_aa1	No hit	IS4	1639624	1640952	1.329
SL3822	CP022781_00082	78.03% ISAzo5_aa1	100% CP022781_01475	IS4 ssgr IS50	100156	101481	1.326
SL3822	CP022781_00169	78.03% ISAzo5_aa1	100% CP022781_01475	IS4 ssgr IS50	213928	215253	1.326
SL3822	CP022781_01475	78.03% ISAzo5_aa1	100% CP022781_00169	IS4 ssgr IS50	1889260	1887935	1.326
SL3822	CP022781_01070	80.95% ISBcen26_aa1	99.13% CP022781_01135	IS481	1369561	1369911	351
SL3822	CP022781_01135	82.75% ISBcen26_aa1	99.13% CP022781_01070	IS481	1448711	1449061	351
SL3822	CP022781_00278	98.17% ISRso1_aa1	98.90% CP022781_00962	IS5	365827	366651	825
SL3822	CP022781_00475	85.56% ISRso1_aa1	86.59% CP022781_00868	IS5	61599	615598	393
SL3822	CP022781_00487	98.54% ISRso1_aa1	98.54% CP022781_00868	IS5	624898	625722	825
SL3822	CP022781_00868	97.81% ISRso1_aa1	98.54% CP022781_00487	IS5	1103154	1103978	825
SL3822	CP022781_00962	98.54% ISRso1_aa1	98.90% CP022781_00278	IS5	1214175	1212742	1.434
SL3822	CP022781_00866	99.25% IS1421_aa1	99.25% CP022781_01067	IS5 ssgr IS427	1100693	1101097	405
SL3822	CP022781_01067	100% IS1421_aa1	99.25% CP022781_00866	IS5 ssgr IS427	1366308	1366712	405
SL3822	CP022781_00042	99.06% IS1405_aa1	100% CP022781_01182	IS5 ssgr IS5	47275	4824	966
SL3822	CP022781_00054	94.51% IS1021_aa1	100% CP022781_01192	IS5 ssgr IS5	61576	62562	987
SL3822	CP022781_00078	94.51% IS1021_aa1	100% CP022781_01192	IS5 ssgr IS5	90473	91459	987
SL3822	CP022781_00084	99.37% IS1405_aa1	100% CP022781_00462	IS5 ssgr IS5	103153	102188	966
SL3822	CP022781_00142	99.06% IS1405_aa1	100% CP022781_01182	IS5 ssgr IS5	180686	179721	966
SL3822	CP022781_00152	99.06% IS1405_aa1	100% CP022781_01182	IS5 ssgr IS5	195549	196514	966
SL3822	CP022781_00361	99.06% IS1405_aa1	100% CP022781_01182	IS5 ssgr IS5	466489	465524	966
SL3822	CP022781_00427	99.06% IS1405_aa1	100% CP022781_01182	IS5 ssgr IS5	565548	566513	966
SL3822	CP022781_00460	94.51% IS1021_aa1	100% CP022781_01192	IS5 ssgr IS5	602823	603809	987
SL3822	CP022781_00462	99.37% IS1405_aa1	100% CP022781_00084	IS5 ssgr IS5	605055	60409	966
SL3822	CP022781_00525	94.51% IS1021_aa1	100% CP022781_01192	IS5 ssgr IS5	669961	668975	987
SL3822	CP022781_00543	99.06% IS1405_aa1	100% CP022781_01182	IS5 ssgr IS5	703631	702666	966
SL3822	CP022781_00703	99.06% IS1405_aa1	100% CP022781_01182	IS5 ssgr IS5	895789	894824	966
SL3822	CP022781_00709	99.06% IS1405_aa1	100% CP022781_01182	IS5 ssgr IS5	901648	902613	966
SL3822	CP022781_00911	99.06% IS1405_aa1	100% CP022781_01182	IS5 ssgr IS5	1154439	1153474	966
SL3822	CP022781_01044	99.06% IS1405_aa1	100% CP022781_01182	IS5 ssgr IS5	1345635	1346600	966
SL3822	CP022781_01182	99.06% IS1405_aa1	100% CP022781_01044	IS5 ssgr IS5	1507970	1507005	966
SL3822	CP022781_01190	94.51% IS1021_aa1	100% CP022781_01192	IS5 ssgr IS5	1524436	1523450	987
SL3822	CP022781_01192	94.51% IS1021_aa1	100% CP022781_01190	IS5 ssgr IS5	1525614	1526600	987
SL3822	CP022781_01511	99.06% IS1405_aa1	99.68% CP022781_00462	IS5 ssgr IS5	1928687	1927722	966
SL3822	CP022781_00280	100% IS1420_aa1	No hit	IS5 ssgr IS903	367815	368771	957
SL3822	CP022781_01068	81.37% ISCARN39_aa1	99.02% CP022781_01133	IS630	1367199	1367510	312

SL3822	CP022781_01133	79.66% ISCARN39_aa3	99.02% CP022781_01068	IS630	1446342	1446878	537
SL3822	CP022781_01061	82.88% ISSal1_aa2	No hit	IS66	1361022	1360678	345
SL3822	CP022781_00721	43.40% ISWz1_aa1	44.13% CP022781_01680	IS91	915534	913834	1.701
SL3822	CP022781_00704	72.27% ISKpn21_aa1	70% CP022781_00705	ISNCY ssgr IS1202	896508	897983	1.476
SL3822	CP022781_00705	62.5% ISAba32_aa1	70% CP022781_00704	ISNCY ssgr IS1202	898126	898341	216
SL3822	CP022781_01106	50.25% ISMpo10_aa3	98.90% CP022781_01170	Tn3	1418048	1419421	1.374
SL3822	CP022781_01170	51.46% ISMpo10_aa3	98.90% CP022781_01106	Tn3	1494035	1495408	1.374
HA4_1	CP022482_00045	95.51% ISBma3_aa1	99.00% CP022482_00428	IS110	52074	50869	1.206
HA4_1	CP022482_00428	95.51% ISBma3_aa1	99.00% CP022482_00045	IS110	559963	561168	1.206
HA4_1	CP022482_00360	88.75% ISBcen4_aa1	100% CP022482_00688	IS110 ssgr IS1111	46868	467661	1.02
HA4_1	CP022482_00688	88.75% ISBcen4_aa1	100% CP022482_00360	IS110 ssgr IS1111	894892	893873	1.02
HA4_1	CP022482_01180	93.43% ISPosp3_aa1	No hit	IS1595 ssgr ISSod11	1500315	1501109	795
HA4_1	CP022482_00025	93.61% ISRme9_aa1	68.99% CP022482_00843	IS21	25799	27352	1.554
HA4_1	CP022482_00026	94.42% ISRme9_aa2	72.91% CP022482_00842	IS21	27361	28119	759
HA4_1	CP022482_00842	85.14% ISCARN95_aa2	72.91% CP022482_00026	IS21	1083589	1082822	768
HA4_1	CP022482_00843	81.85% ISCARN95_aa1	68.99% CP022482_00025	IS21	1085125	1083602	1.524
HA4_1	CP022482_01450	100% ISRso11_aa1	No hit	IS3 ssgr IS150	1840087	1840620	534
HA4_1	CP022482_01451	99.64% ISRso11_aa2	No hit	IS3 ssgr IS150	1840617	1841453	837
HA4_1	CP022482_00359	93.00% ISRso10_aa2	No hit	IS3 ssgr IS2	467168	466437	732
HA4_1	CP022482_00361	95.74% ISRso10_aa1	No hit	IS3 ssgr IS2	469035	468655	381
HA4_1	CP022482_01110	79.41% ISRso20_aa1	No hit	IS3 ssgr IS3	1395186	1394884	303
HA4_1	CP022482_00897	92.85% ISRso16_aa2	No hit	IS3 ssgr IS407	1135023	1134538	486
HA4_1	CP022482_00936	64.57% IST3091_aa1	No hit	IS30	1195999	1195322	678
HA4_1	CP022482_00486	61.68% ISCro3_aa1	No hit	IS4	624874	626202	1.329
HA4_1	CP022482_00376	98.17% ISRso1_aa1	100% CP022482_01222	IS5	483621	484445	825
HA4_1	CP022482_00941	98.17% ISRso1_aa1	100% CP022482_01222	IS5	1201992	1201168	825
HA4_1	CP022482_01222	98.17% ISRso1_aa1	100% CP022482_00941	IS5	1544292	1545116	825
HA4_1	CP022482_00175	99.25% IS1421_aa1	100% CP022482_00938	IS5 ssgr IS427	221805	222209	405
HA4_1	CP022482_00483	100% IS1421_aa1	100% CP022482_00942	IS5 ssgr IS427	623624	62322	405
HA4_1	CP022482_00812	100% IS1421_aa1	100% CP022482_01157	IS5 ssgr IS427	1048246	1047842	405
HA4_1	CP022482_00938	99.25% IS1421_aa1	100% CP022482_00175	IS5 ssgr IS427	1198524	1198120	405
HA4_1	CP022482_00942	100% IS1421_aa1	100% CP022482_00483	IS5 ssgr IS427	1202489	1202893	405
HA4_1	CP022482_01116	100% IS1421_aa1	100% CP022482_01157	IS5 ssgr IS427	1399549	1399953	405
HA4_1	CP022482_01157	100% IS1421_aa1	100% CP022482_01116	IS5 ssgr IS427	1466338	1465934	405
HA4_1	CP022482_00244	94.51% IS1021_aa1	100% CP022482_01408	IS5 ssgr IS5	301266	30028	987
HA4_1	CP022482_00389	94.51% IS1021_aa1	100% CP022482_01408	IS5 ssgr IS5	512547	511561	987
HA4_1	CP022482_00618	94.51% IS1021_aa1	100% CP022482_01408	IS5 ssgr IS5	7926	791614	987
HA4_1	CP022482_00678	94.51% IS1021_aa1	100% CP022482_01408	IS5 ssgr IS5	881756	88077	987
HA4_1	CP022482_00691	94.51% IS1021_aa1	100% CP022482_01408	IS5 ssgr IS5	897091	898077	987
HA4_1	CP022482_00692	94.51% IS1021_aa1	100% CP022482_01408	IS5 ssgr IS5	898303	899289	987
HA4_1	CP022482_00693	94.51% IS1021_aa1	100% CP022482_01408	IS5 ssgr IS5	899515	900501	987
HA4_1	CP022482_00819	94.51% IS1021_aa1	100% CP022482_01408	IS5 ssgr IS5	1056783	1055797	987

HA4_1	CP022482_00963	94.51% IS1021_aa1	100% CP022482_01408	IS5 ssgr IS5	1223958	1224944	987
HA4_1	CP022482_01132	94.51% IS1021_aa1	100% CP022482_01408	IS5 ssgr IS5	1440704	1439718	987
HA4_1	CP022482_01408	94.51% IS1021_aa1	100% CP022482_01132	IS5 ssgr IS5	1785451	1784465	987
HA4_1	CP022482_01068	100% IS1420_aa1	100% CP022482_01453	IS5 ssgr IS903	1347025	1347981	957
HA4_1	CP022482_01453	100% IS1420_aa1	100% CP022482_01068	IS5 ssgr IS903	1844162	1845118	957
HA4_1	CP022482_00002	64.95% ISAeh1_aa2	No hit	IS66	32	2535	666
HA4_1	CP022482_00003	67.14% ISSba7_aa3	No hit	IS66	3689	3231	459
HA4_1	CP022482_00005	74.56% ISBmu30_aa1	No hit	IS66	4462	4109	354
HA4_1	CP022482_00043	43.40% ISWz1_aa1	78.46% CP022482_00008	IS91	49877	48177	1.701
HA4_1	CP022482_01533	72.27% ISKpn21_aa1	70% CP022482_01534	ISNCY ssgr IS1202	1944201	1945676	1.476
HA4_1	CP022482_01534	62.5% ISAba32_aa1	70% CP022482_01533	ISNCY ssgr IS1202	1945818	1946033	216
HA4_1	CP022482_00007	65.81% ISPa43_aa2	46.20% CP022482_00028	Tn3	9467	6483	2.985
HA4_1	CP022482_00028	93.91% TnShfr1_aa4	45.61% CP022482_00007	Tn3	32718	29752	2.967
HA4_1	CP022482_00029	87.02% ISPa38_aa1	No hit	Tn3	32875	33432	558
pHA4_1	CP022483_00056	85.14% ISCARN95_aa2	72.91% CP022483_00082	IS21	55346	54579	768
pHA4_1	CP022483_00057	81.85% ISCARN95_aa1	68.99% CP022483_00081	IS21	56882	55359	1.524
pHA4_1	CP022483_00081	93.61% ISRme9_aa1	68.99% CP022483_00057	IS21	89965	91518	1.554
pHA4_1	CP022483_00082	94.42% ISRme9_aa2	72.91% CP022483_00056	IS21	91527	92285	759
pHA4_1	CP022483_00146	87.15% IS1421_aa1	No hit	IS5 ssgr IS427	137575	137946	372
pHA4_1	CP022483_00024	94.81% IS1021_aa1	No hit	IS5 ssgr IS5	30641	29655	987
pHA4_1	CP022483_00085	59.54% ISAeh1_aa2	100% CP022483_00126	IS66	97788	96235	1.554
pHA4_1	CP022483_00086	74.56% ISBmu30_aa1	100% CP022483_00125	IS66	98174	97821	354
pHA4_1	CP022483_00087	59.06% ISRm2_aa1	100% CP022483_00124	IS66	98647	98171	477
pHA4_1	CP022483_00124	59.06% ISRm2_aa1	100% CP022483_00087	IS66	115023	115499	477
pHA4_1	CP022483_00125	74.56% ISBmu30_aa1	100% CP022483_00086	IS66	115496	115849	354
pHA4_1	CP022483_00126	59.54% ISAeh1_aa2	100% CP022483_00085	IS66	115882	117435	1.554
pHA4_1	CP022483_00144	68.96% ISPa43_aa2	48.05% CP022483_00149	Tn3	136077	133918	2.16
pHA4_1	CP022483_00149	94.62% TnShfr1_aa4	47.33% CP022483_00144	Tn3	142011	139048	2.964
pHA4_1	CP022483_00150	87.56% ISPa38_aa1	No hit	Tn3	142168	142725	558
pHA4_1	CP022483_00151	57.29% ISPa43_aa2	No hit	Tn3	143519	142722	798
KACC10709	CP016905_00711	88.75% ISBcen4_aa1	100% CP016905_01238	IS110 ssgr IS1111	884924	885943	1.02
KACC10709	CP016905_00782	61.67% ISMno14_aa1	100% CP016905_00790	IS110 ssgr IS1111	981623	982636	1.014
KACC10709	CP016905_00790	61.67% ISMno14_aa1	100% CP016905_00782	IS110 ssgr IS1111	988775	987762	1.014
KACC10709	CP016905_01238	88.75% ISBcen4_aa1	100% CP016905_00711	IS110 ssgr IS1111	1545776	1546795	1.02
KACC10709	CP016905_01308	88.08% ISBcen4_aa1	100% CP016905_01238	IS110 ssgr IS1111	1630953	1631990	1.038
KACC10709	CP016905_00529	83.54% ISBusp4_aa1	99.79% CP016905_00755	IS1182	679159	677714	1.446
KACC10709	CP016905_00755	83.75% ISBusp4_aa1	99.79% CP016905_00529	IS1182	943046	941601	1.446
KACC10709	CP016905_01255	82.73% ISBusp4_aa1	100% CP016905_00755	IS1182	1564060	1565316	1.257
KACC10709	CP016905_00094	91.08% ISPosp3_aa1	100% CP016905_01295	IS1595 ssgr ISSod11	124241	123255	987
KACC10709	CP016905_00258	91.08% ISPosp3_aa1	100% CP016905_01295	IS1595 ssgr ISSod11	314105	315091	987
KACC10709	CP016905_01295	91.08% ISPosp3_aa1	100% CP016905_00258	IS1595 ssgr ISSod11	1617491	1618477	987

KACC10709	CP016905_00824	99.64% ISRso11_aa2	100% CP016905_01556	IS3 ssgr IS150	1017492	1016656	837
KACC10709	CP016905_00825	100% ISRso11_aa1	100% CP016905_01555	IS3 ssgr IS150	1018022	1017489	534
KACC10709	CP016905_01555	100% ISRso11_aa1	100% CP016905_00825	IS3 ssgr IS150	1935582	1936115	534
KACC10709	CP016905_01556	99.64% ISRso11_aa2	100% CP016905_00824	IS3 ssgr IS150	1936112	1936948	837
KACC10709	CP016905_00339	95.74% ISRso10_aa1	No hit	IS3 ssgr IS2	43253	43291	381
KACC10709	CP016905_01239	93.41% ISRso10_aa2	45.02% CP016905_00783	IS3 ssgr IS2	1547288	1548019	732
KACC10709	CP016905_00478	79.45% ISRso20_aa1	No hit	IS3 ssgr IS3	625245	625529	285
KACC10709	CP016905_00783	93.25% ISRso16_aa2	44.58% CP016905_01556	IS3 ssgr IS407	982983	983594	612
KACC10709	CP016905_00696	89.75% ISAisp2_aa2	100% CP016905_01429	IS3 ssgr IS51	872355	871504	852
KACC10709	CP016905_00697	81.52% ISAisp2_aa1	100% CP016905_01430	IS3 ssgr IS51	87263	872352	279
KACC10709	CP016905_01270	81.52% ISAisp2_aa1	97.82% CP016905_01430	IS3 ssgr IS51	1582644	1582922	279
KACC10709	CP016905_01271	89.34% ISAisp2_aa2	98.28% CP016905_01429	IS3 ssgr IS51	1582919	1583794	876
KACC10709	CP016905_01364	89.00% ISAisp2_aa2	99.65% CP016905_01429	IS3 ssgr IS51	1700245	1699370	876
KACC10709	CP016905_01365	81.52% ISAisp2_aa1	100% CP016905_01430	IS3 ssgr IS51	1700520	1700242	279
KACC10709	CP016905_01429	89.34% ISAisp2_aa2	99.65% CP016905_01364	IS3 ssgr IS51	1781302	1780427	876
KACC10709	CP016905_01430	81.52% ISAisp2_aa1	100% CP016905_01365	IS3 ssgr IS51	1781577	1781299	279
KACC10709	CP016905_01155	61.68% ISCro3_aa1	No hit	IS4	1451023	1452351	1.329
KACC10709	CP016905_00141	97.81% ISRso1_aa1	100% CP016905_01664	IS5	179536	18036	825
KACC10709	CP016905_00232	98.54% ISRso1_aa1	97.92% CP016905_00445	IS5	289779	288346	1.434
KACC10709	CP016905_00445	97.81% ISRso1_aa1	100% CP016905_01664	IS5	571931	571041	891
KACC10709	CP016905_00468	98.66% ISRso1_aa1	100% CP016905_00706	IS5	602168	602845	678
KACC10709	CP016905_00706	98.66% ISRso1_aa1	100% CP016905_00468	IS5	881405	882082	678
KACC10709	CP016905_01084	97.81% ISRso1_aa1	100% CP016905_01664	IS5	1377593	1378417	825
KACC10709	CP016905_01158	97.81% ISRso1_aa1	100% CP016905_01664	IS5	1453505	1454329	825
KACC10709	CP016905_01324	97.81% ISRso1_aa1	100% CP016905_01664	IS5	1652137	1651313	825
KACC10709	CP016905_01366	88.04% ISRso1_aa1	89.13% CP016905_00706	IS5	1700878	1700552	327
KACC10709	CP016905_01664	97.81% ISRso1_aa1	100% CP016905_01324	IS5	2064553	2063729	825
KACC10709	CP016905_00134	99.25% IS1421_aa1	99.25% CP016905_01431	IS5 ssgr IS427	17389	174294	405
KACC10709	CP016905_00140	100% IS1421_aa1	100% CP016905_01431	IS5 ssgr IS427	179351	178947	405
KACC10709	CP016905_00338	100% IS1421_aa1	100% CP016905_01431	IS5 ssgr IS427	431689	432093	405
KACC10709	CP016905_00444	100% IS1421_aa1	100% CP016905_01431	IS5 ssgr IS427	570788	570384	405
KACC10709	CP016905_00698	100% IS1421_aa1	100% CP016905_01431	IS5 ssgr IS427	872734	873138	405
KACC10709	CP016905_00939	100% IS1421_aa1	100% CP016905_01431	IS5 ssgr IS427	1162686	1163090	405
KACC10709	CP016905_01064	100% IS1421_aa1	100% CP016905_01431	IS5 ssgr IS427	1348640	1349044	405
KACC10709	CP016905_01431	100% IS1421_aa1	100% CP016905_01064	IS5 ssgr IS427	1781681	1782085	405
KACC10709	CP016905_00137	99.06% IS1405_aa1	100% CP016905_01479	IS5 ssgr IS5	176798	175833	966
KACC10709	CP016905_00295	99.06% IS1405_aa1	100% CP016905_01479	IS5 ssgr IS5	388873	389838	966
KACC10709	CP016905_00332	99.06% IS1405_aa1	100% CP016905_01479	IS5 ssgr IS5	42759	428555	966
KACC10709	CP016905_00336	99.06% IS1405_aa1	100% CP016905_01479	IS5 ssgr IS5	430985	43002	966
KACC10709	CP016905_00447	99.06% IS1405_aa1	100% CP016905_01479	IS5 ssgr IS5	573456	572491	966
KACC10709	CP016905_00535	94.81% IS1021_aa1	100% CP016905_01516	IS5 ssgr IS5	681398	682384	987
KACC10709	CP016905_00537	99.06% IS1405_aa1	100% CP016905_00563	IS5 ssgr IS5	683105	68407	966
KACC10709	CP016905_00563	99.06% IS1405_aa1	100% CP016905_00537	IS5 ssgr IS5	718853	719818	966

KACC10709	CP016905_00567	99.06% IS1405_aa1	100% CP016905_01479	IS5 ssgr IS5	722248	721283	966
KACC10709	CP016905_00701	99.37% IS1405_aa1	100% CP016905_01499	IS5 ssgr IS5	876944	875979	966
KACC10709	CP016905_00704	94.81% IS1021_aa1	100% CP016905_01516	IS5 ssgr IS5	880661	879675	987
KACC10709	CP016905_00705	87.5% IS1405_aa1	88.75% CP016905_00701	IS5 ssgr IS5	880818	88133	513
KACC10709	CP016905_00707	99.45% IS1405_aa1	100% CP016905_01479	IS5 ssgr IS5	882113	88267	558
KACC10709	CP016905_00831	94.81% IS1021_aa1	100% CP016905_01516	IS5 ssgr IS5	1023027	1024013	987
KACC10709	CP016905_00920	94.81% IS1021_aa1	100% CP016905_01516	IS5 ssgr IS5	1127861	1128847	987
KACC10709	CP016905_00945	99.06% IS1405_aa1	100% CP016905_01479	IS5 ssgr IS5	1167395	1166430	966
KACC10709	CP016905_00947	94.81% IS1021_aa1	100% CP016905_01516	IS5 ssgr IS5	1169102	1168116	987
KACC10709	CP016905_01017	99.06% IS1405_aa1	100% CP016905_01479	IS5 ssgr IS5	1281103	1282068	966
KACC10709	CP016905_01087	99.06% IS1405_aa1	100% CP016905_01479	IS5 ssgr IS5	1379991	1379026	966
KACC10709	CP016905_01223	94.81% IS1021_aa1	100% CP016905_01516	IS5 ssgr IS5	1526379	1527365	987
KACC10709	CP016905_01242	99.06% IS1405_aa1	100% CP016905_01479	IS5 ssgr IS5	1549458	1550423	966
KACC10709	CP016905_01253	99.06% IS1405_aa1	100% CP016905_01479	IS5 ssgr IS5	1562248	1561283	966
KACC10709	CP016905_01268	94.81% IS1021_aa1	100% CP016905_01516	IS5 ssgr IS5	1579655	1578669	987
KACC10709	CP016905_01299	99.06% IS1405_aa1	100% CP016905_01479	IS5 ssgr IS5	1621712	1620747	966
KACC10709	CP016905_01479	99.06% IS1405_aa1	100% CP016905_01299	IS5 ssgr IS5	1844611	1845576	966
KACC10709	CP016905_01499	99.37% IS1405_aa1	100% CP016905_00701	IS5 ssgr IS5	1868127	1869092	966
KACC10709	CP016905_01516	94.81% IS1021_aa1	100% CP016905_01268	IS5 ssgr IS5	1893166	1892180	987
KACC10709	CP016905_00139	100% IS1420_aa1	100% CP016905_01702	IS5 ssgr IS903	177544	1785	957
KACC10709	CP016905_00163	100% IS1420_aa1	100% CP016905_01702	IS5 ssgr IS903	211482	212438	957
KACC10709	CP016905_00267	100% IS1420_aa1	100% CP016905_01702	IS5 ssgr IS903	358329	357373	957
KACC10709	CP016905_00293	100% IS1420_aa1	100% CP016905_01702	IS5 ssgr IS903	386892	387848	957
KACC10709	CP016905_00443	100% IS1420_aa1	100% CP016905_01702	IS5 ssgr IS903	568981	569937	957
KACC10709	CP016905_00449	100% IS1420_aa1	100% CP016905_01702	IS5 ssgr IS903	576805	577761	957
KACC10709	CP016905_00453	100% IS1420_aa1	100% CP016905_01702	IS5 ssgr IS903	582536	58158	957
KACC10709	CP016905_00558	100% IS1420_aa1	100% CP016905_01702	IS5 ssgr IS903	707422	708378	957
KACC10709	CP016905_00621	100% IS1420_aa1	100% CP016905_01702	IS5 ssgr IS903	77872	779676	957
KACC10709	CP016905_00623	100% IS1420_aa1	100% CP016905_01702	IS5 ssgr IS903	781809	780853	957
KACC10709	CP016905_00683	100% IS1420_aa1	100% CP016905_01702	IS5 ssgr IS903	857514	856558	957
KACC10709	CP016905_00720	100% IS1420_aa1	100% CP016905_01702	IS5 ssgr IS903	896285	897241	957
KACC10709	CP016905_00722	99.68% IS1420_aa1	100% CP016905_01062	IS5 ssgr IS903	900464	899508	957
KACC10709	CP016905_00736	100% IS1420_aa1	100% CP016905_01702	IS5 ssgr IS903	917213	916377	837
KACC10709	CP016905_00737	100% IS1420_aa1	100% CP016905_01702	IS5 ssgr IS903	917334	91829	957
KACC10709	CP016905_00854	100% IS1420_aa1	100% CP016905_01702	IS5 ssgr IS903	1052635	1051679	957
KACC10709	CP016905_00856	100% IS1420_aa1	100% CP016905_01702	IS5 ssgr IS903	1053045	1054001	957
KACC10709	CP016905_00887	100% IS1420_aa1	100% CP016905_01702	IS5 ssgr IS903	1085993	1086847	855
KACC10709	CP016905_00928	100% IS1420_aa1	100% CP016905_01702	IS5 ssgr IS903	1134268	1135224	957
KACC10709	CP016905_00940	100% IS1420_aa1	100% CP016905_01702	IS5 ssgr IS903	1164493	1163537	957
KACC10709	CP016905_00963	100% IS1420_aa1	100% CP016905_01702	IS5 ssgr IS903	1200720	1199764	957
KACC10709	CP016905_01062	99.68% IS1420_aa1	100% CP016905_00722	IS5 ssgr IS903	1347301	1348257	957
KACC10709	CP016905_01221	100% IS1420_aa1	100% CP016905_01702	IS5 ssgr IS903	1524848	1525804	957
KACC10709	CP016905_01254	100% IS1420_aa1	100% CP016905_01702	IS5 ssgr IS903	1563797	1562841	957

KACC10709	CP016905_01301	100% IS1420_aa1	100% CP016905_01702	IS5 ssgr IS903	1625061	1626017	957
KACC10709	CP016905_01336	100% IS1420_aa1	100% CP016905_01702	IS5 ssgr IS903	1674087	1675043	957
KACC10709	CP016905_01347	100% IS1420_aa1	100% CP016905_01702	IS5 ssgr IS903	1683992	1684948	957
KACC10709	CP016905_01501	100% IS1420_aa1	100% CP016905_01702	IS5 ssgr IS903	1869517	1870473	957
KACC10709	CP016905_01611	100% IS1420_aa1	100% CP016905_01702	IS5 ssgr IS903	1992872	1991916	957
KACC10709	CP016905_01683	100% IS1420_aa1	100% CP016905_01702	IS5 ssgr IS903	2113585	2114541	957
KACC10709	CP016905_01702	100% IS1420_aa1	100% CP016905_01683	IS5 ssgr IS903	2133233	2132277	957
KACC10709	CP016905_00252	55.55% ISRm2_aa1	100% CP016905_01191	IS66	309513	309977	465
KACC10709	CP016905_00253	73.27% ISRm2_aa2	100% CP016905_01192	IS66	309974	310327	354
KACC10709	CP016905_00254	60.75% ISPpu13_aa2	100% CP016905_01193	IS66	31036	311949	1.59
KACC10709	CP016905_00341	56.95% ISRm2_aa1	100% CP016905_01353	IS66	433033	433515	483
KACC10709	CP016905_00342	73.27% ISRm2_aa2	100% CP016905_01352	IS66	433512	433865	354
KACC10709	CP016905_00343	59.79% ISAeh1_aa2	100% CP016905_01351	IS66	433898	435454	1.557
KACC10709	CP016905_00569	59.79% ISAeh1_aa2	100% CP016905_01351	IS66	725868	724312	1.557
KACC10709	CP016905_00570	73.27% ISRm2_aa2	100% CP016905_01352	IS66	726254	725901	354
KACC10709	CP016905_00571	56.95% ISRm2_aa1	100% CP016905_01353	IS66	726733	726251	483
KACC10709	CP016905_00692	55.55% ISRm2_aa1	100% CP016905_01191	IS66	867791	868255	465
KACC10709	CP016905_00693	73.27% ISRm2_aa2	100% CP016905_01192	IS66	868252	868605	354
KACC10709	CP016905_00694	60.75% ISPpu13_aa2	100% CP016905_01193	IS66	868638	870227	1.59
KACC10709	CP016905_00784	59.79% ISAeh1_aa2	100% CP016905_01351	IS66	985071	983515	1.557
KACC10709	CP016905_00785	73.27% ISRm2_aa2	100% CP016905_01352	IS66	985457	985104	354
KACC10709	CP016905_00786	56.95% ISRm2_aa1	100% CP016905_01353	IS66	985936	985454	483
KACC10709	CP016905_01191	55.55% ISRm2_aa1	100% CP016905_00692	IS66	1494802	1495266	465
KACC10709	CP016905_01192	73.27% ISRm2_aa2	100% CP016905_00693	IS66	1495263	1495616	354
KACC10709	CP016905_01193	60.75% ISPpu13_aa2	100% CP016905_00694	IS66	1495649	1497238	1.59
KACC10709	CP016905_01305	56.95% ISRm2_aa1	100% CP016905_01353	IS66	1628559	1629041	483
KACC10709	CP016905_01306	73.27% ISRm2_aa2	100% CP016905_01352	IS66	1629038	1629391	354
KACC10709	CP016905_01307	59.79% ISAeh1_aa2	100% CP016905_01351	IS66	1629424	1630980	1.557
KACC10709	CP016905_01351	59.79% ISAeh1_aa2	100% CP016905_01307	IS66	1689719	1688163	1.557
KACC10709	CP016905_01352	73.27% ISRm2_aa2	100% CP016905_01306	IS66	1690105	1689752	354
KACC10709	CP016905_01353	56.95% ISRm2_aa1	100% CP016905_01305	IS66	1690584	1690102	483
KACC10709	CP016905_00001	42.78% ISWz1_aa1	100% CP016905_01632	IS91	26	637	612
KACC10709	CP016905_01632	42.78% ISWz1_aa1	100% CP016905_00001	IS91	2010734	2009061	1.674
KACC10709	CP016905_01637	62.5% ISKpn21_aa1	No hit	ISNCY ssgr IS1202	2016979	2017380	402
KACC10709	CP016905_01635	75.15% ISPa42_aa1	100% CP016905_01705	Tn3	2015335	2012390	2.946
KACC10709	CP016905_01705	75.15% ISPa42_aa1	100% CP016905_01635	Tn3	2140096	2143041	2.946
CMR15	FP885896_00302	91.68% ISBma2_aa1	100% FP885896_00787	IS1182	391145	392629	1.485
CMR15	FP885896_00787	91.68% ISBma2_aa1	100% FP885896_00302	IS1182	997345	995861	1.485
CMR15	FP885896_01007	91.19% ISBma2_aa1	99.71% FP885896_00787	IS1182	1281570	1282850	1.281
CMR15	FP885896_01008	83.65% ISBfun3_aa1	100% FP885896_00787	IS1182	1282801	1283166	366
CMR15	FP885896_01501	98.36% ISRso10_aa1	No hit	IS3 ssgr IS2	1911844	1912233	390
CMR15	FP885896_01502	95.74% ISRso10_aa2	94.44% FP885896_01347	IS3 ssgr IS2	1912230	1913078	849

CMR15	FP885896_00359	38.01% ISGur11_aa1	No hit	IS481	44686	448542	1.683
CMR15	FP885896_00836	75.23% ISAzs36_aa2	No hit	IS481	1057595	1056624	972
CMR15	FP885896_00837	73.29% ISAzs36_aa1	No hit	IS481	1059246	1057585	1.662
CMR15	FP885896_01317	74.31% ISRel12_aa1	No hit	IS481	1677322	1677780	459
CMR15	FP885896_00282	96.71% ISRso1_aa1	100% FP885896_00851	IS5	371778	372602	825
CMR15	FP885896_00720	89.39% ISRso1_aa1	92.42% FP885896_00851	IS5	918138	918338	201
CMR15	FP885896_00721	95.23% ISRso1_aa1	95.38% FP885896_00851	IS5	918398	918616	219
CMR15	FP885896_00722	92.40% ISRso1_aa1	91.13% FP885896_00851	IS5	918655	918882	228
CMR15	FP885896_00849	65.24% ISRso1_aa1	65.97% FP885896_00851	IS5	1074382	1074720	339
CMR15	FP885896_00851	96.71% ISRso1_aa1	100% FP885896_00282	IS5	1076485	1077309	825
CMR15	FP885896_00492	67.85% IS1420_aa1	No hit	IS5 ssgr IS903	608269	607916	354
CMR15	FP885896_00818	43.40% ISWz1_aa1	41.79% FP885896_01547	IS91	1035549	1037249	1.701
CMR15	FP885896_00086	73.41% ISSpu3_aa1	99.68% FP885896_00823	ISAs1	101377	102417	1.041
CMR15	FP885896_00167	73.90% ISSpu3_aa1	99.72% FP885896_00823	ISAs1	213095	212001	1.095
CMR15	FP885896_00679	74.17% ISSpu3_aa1	100% FP885896_00823	ISAs1	86395	862856	1.095
CMR15	FP885896_00794	73.14% ISSpu3_aa1	100% FP885896_00823	ISAs1	1008018	1007692	327
CMR15	FP885896_00795	74.04% ISPlu18_aa1	99.57% FP885896_00823	ISAs1	1008785	1008054	732
CMR15	FP885896_00823	74.17% ISSpu3_aa1	100% FP885896_00679	ISAs1	1042905	1041811	1.095
CMR15	FP885896_00733	51.33% ISSm4_aa2	47.94% FP885896_01230	ISL3	931796	932977	1.182
CMR15	FP885896_01230	51.07% ISSm4_aa2	47.94% FP885896_00733	ISL3	1572288	1573277	990
CMR15	FP885896_00833	68.18% ISKpn21_aa1	81.81% FP885896_00841	ISNCY ssgr IS1202	1051889	1051587	303
CMR15	FP885896_00840	74.10% ISKpn21_aa1	No hit	ISNCY ssgr IS1202	1062202	1061672	531
CMR15	FP885896_00841	70.95% ISKpn21_aa1	81.81% FP885896_00833	ISNCY ssgr IS1202	1062969	1062199	771
CMR15	FP885896_00060	61.76% ISPa40_aa4	63.76% FP885896_00838	Tn3	65834	66064	231
CMR15	FP885896_00838	71.03% ISThsp9_aa1	63.76% FP885896_00060	Tn3	1059802	1059227	576
RS476	CP021763_00428	100% ISRso6_aa1	100% CP021763_00917	IS21	562932	563975	1.044
RS476	CP021763_00429	100% ISRso6_aa2	100% CP021763_00918	IS21	563972	564817	846
RS476	CP021763_00917	100% ISRso6_aa1	100% CP021763_00428	IS21	1197016	1198059	1.044
RS476	CP021763_00918	100% ISRso6_aa2	100% CP021763_00429	IS21	1198056	1198901	846
RS476	CP021763_00456	100% ISRso7_aa1	No hit	IS256	598843	600093	1.251
RS476	CP021763_00520	100% ISRso8_aa1	No hit	IS3	687804	688097	294
RS476	CP021763_00521	100% ISRso8_aa2	42.08% CP021763_01300	IS3	688094	688984	891
RS476	CP021763_00437	100% ISRso10_aa2	93.82% CP021763_00540	IS3 ssgr IS2	578374	577526	849
RS476	CP021763_00438	100% ISRso10_aa1	96.12% CP021763_00539	IS3 ssgr IS2	57876	578371	390
RS476	CP021763_00539	97.54% ISRso10_aa1	96.12% CP021763_00438	IS3 ssgr IS2	707941	70833	390
RS476	CP021763_00540	93.82% ISRso10_aa2	93.82% CP021763_00437	IS3 ssgr IS2	708444	709175	732
RS476	CP021763_01342	78.68% ISRso20_aa1	62.06% CP021763_00520	IS3 ssgr IS3	1738636	1738836	201
RS476	CP021763_00481	100% ISRso12_aa1	100% CP021763_01268	IS3 ssgr IS407	625173	625439	267
RS476	CP021763_00482	100% ISRso12_aa2	100% CP021763_01300	IS3 ssgr IS407	625589	626293	705
RS476	CP021763_00530	100% ISRso16_aa1	87.5% CP021763_01268	IS3 ssgr IS407	699424	69969	267
RS476	CP021763_00531	100% ISRso16_aa2	92.41% CP021763_01585	IS3 ssgr IS407	699774	700565	792
RS476	CP021763_01184	100% ISRso14_aa1	74.11% CP021763_01268	IS3 ssgr IS407	1547521	1547784	264

RS476	CP021763_01185	100% ISRso14_aa2	67.64% CP021763_01585	IS3 ssgr IS407	1548090	1548638	549
RS476	CP021763_01268	100% ISRso12_aa1	100% CP021763_00481	IS3 ssgr IS407	1648698	1648964	267
RS476	CP021763_01272	100% ISRso12_aa2	100% CP021763_01300	IS3 ssgr IS407	1652954	1653658	705
RS476	CP021763_01300	100% ISRso12_aa2	100% CP021763_01272	IS3 ssgr IS407	1683144	1683848	705
RS476	CP021763_01582	92.06% ISRso16_aa2	100% CP021763_01585	IS3 ssgr IS407	2047115	2047627	513
RS476	CP021763_01585	92.41% ISRso16_aa2	92.41% CP021763_00531	IS3 ssgr IS407	2049257	2048583	675
RS476	CP021763_01546	72.26% ISRme10_aa1	No hit	IS30	1992076	1992459	384
RS476	CP021763_00443	100% ISRso13_aa1	100% CP021763_01269	IS4 ssgr IS4	581896	58323	1.335
RS476	CP021763_01116	100% ISRso13_aa1	100% CP021763_01269	IS4 ssgr IS4	1450089	1448755	1.335
RS476	CP021763_01269	100% ISRso13_aa1	100% CP021763_01116	IS4 ssgr IS4	1650311	1648977	1.335
RS476	CP021763_00111	99.27% ISRso1_aa1	99.63% CP021763_01299	IS5	148179	147355	825
RS476	CP021763_01271	99.63% ISRso1_aa1	100% CP021763_01299	IS5	1652758	1651934	825
RS476	CP021763_01299	99.63% ISRso1_aa1	100% CP021763_01271	IS5	1682948	1682124	825
RS476	CP021763_00040	100% IS1421_aa1	100% CP021763_00959	IS5 ssgr IS427	45892	46296	405
RS476	CP021763_00083	100% IS1421_aa1	100% CP021763_00959	IS5 ssgr IS427	102236	101832	405
RS476	CP021763_00281	100% IS1421_aa1	100% CP021763_00959	IS5 ssgr IS427	396329	395925	405
RS476	CP021763_00959	100% IS1421_aa1	100% CP021763_00281	IS5 ssgr IS427	1245159	1245563	405
RS476	CP021763_00135	100% ISRso9_aa1	100% CP021763_00557	IS5 ssgr IS5	179165	180487	1.323
RS476	CP021763_00191	94.51% IS1021_aa1	100% CP021763_01147	IS5 ssgr IS5	255634	254648	987
RS476	CP021763_00557	100% ISRso9_aa1	100% CP021763_00135	IS5 ssgr IS5	721452	72013	1.323
RS476	CP021763_01103	97.50% IS1405_aa1	70.26% CP021763_01147	IS5 ssgr IS5	1431548	1430583	966
RS476	CP021763_01147	94.51% IS1021_aa1	100% CP021763_00191	IS5 ssgr IS5	1487540	1486554	987
RS476	CP021763_00201	100% ISRso5_aa1	100% CP021763_01630	IS630	269779	27087	1.092
RS476	CP021763_00427	100% ISRso5_aa1	100% CP021763_01630	IS630	562809	562294	516
RS476	CP021763_00430	100% ISRso5_aa1	100% CP021763_01630	IS630	565453	564869	585
RS476	CP021763_01119	100% ISRso5_aa1	100% CP021763_01630	IS630	1452493	1451402	1.092
RS476	CP021763_01630	100% ISRso5_aa1	100% CP021763_01119	IS630	2087384	2088475	1.092
RS476	CP021763_00534	79.46% ISPpu19_aa3	No hit	IS66	70424	703791	450
RS476	CP021763_00110	100% ISRso17_aa1	100% CP021763_01326	IS701	145895	147256	1.362
RS476	CP021763_00136	100% ISRso17_aa1	100% CP021763_01326	IS701	180484	181821	1.338
RS476	CP021763_00453	100% ISRso17_aa1	100% CP021763_01326	IS701	591256	589925	1.332
RS476	CP021763_01102	100% ISRso17_aa1	100% CP021763_01326	IS701	1428997	1430358	1.362
RS476	CP021763_01270	100% ISRso17_aa1	100% CP021763_01326	IS701	1651882	1650521	1.362
RS476	CP021763_01298	100% ISRso17_aa1	100% CP021763_01326	IS701	1682072	1680711	1.362
RS476	CP021763_01326	100% ISRso17_aa1	100% CP021763_01298	IS701	1712262	1710901	1.362
RS476	CP021763_00082	46.07% ISMno24_aa2	40.67% CP021763_00869	IS91	9875	99838	1.089
RS476	CP021763_00869	43.95% ISWz1_aa1	43.44% CP021763_01632	IS91	1138813	1140513	1.701
RS476	CP021763_01508	100% ISRso15_aa1	No hit	ISL3	1949088	1947868	1.221
RS476	CP021763_00884	60.93% ISAba32_aa1	No hit	ISNCY ssgr IS1202	1157632	1157417	216
CRMRs218	CP021765_03249	49.48% ISHvo5_aa1	44.65% CP021765_00907	IS1595 ssgr ISH4	3439477	3438689	789
CRMRs218	CP021765_00250	100% ISRso6_aa1	100% CP021765_02381	IS21	275316	276359	1.044
CRMRs218	CP021765_00251	100% ISRso6_aa2	100% CP021765_02382	IS21	276356	277201	846

CRMRs218	CP021765_02381	100% ISRso6_aa1	100% CP021765_00250	IS21	2531806	2532849	1.044
CRMRs218	CP021765_02382	100% ISRso6_aa2	100% CP021765_00251	IS21	2532846	2533691	846
CRMRs218	CP021765_00255	95.43% ISRso7_aa1	No hit	IS256	282895	284145	1.251
CRMRs218	CP021765_00592	100% ISRso8_aa2	100% CP021765_02313	IS3	623236	622346	891
CRMRs218	CP021765_00593	100% ISRso8_aa1	100% CP021765_02314	IS3	623526	623233	294
CRMRs218	CP021765_01590	100% ISRso8_aa2	100% CP021765_02313	IS3	1662560	1661670	891
CRMRs218	CP021765_01591	100% ISRso8_aa1	100% CP021765_02314	IS3	1662850	1662557	294
CRMRs218	CP021765_02313	100% ISRso8_aa2	100% CP021765_01590	IS3	2459097	2458207	891
CRMRs218	CP021765_02314	100% ISRso8_aa1	100% CP021765_01591	IS3	2459387	2459094	294
CRMRs218	CP021765_01474	100% ISRso11_aa2	100% CP021765_03154	IS3 ssgr IS150	1540951	1540115	837
CRMRs218	CP021765_01475	100% ISRso11_aa1	100% CP021765_03155	IS3 ssgr IS150	1541481	1540948	534
CRMRs218	CP021765_02311	100% ISRso11_aa2	100% CP021765_03154	IS3 ssgr IS150	2457645	2456809	837
CRMRs218	CP021765_02312	100% ISRso11_aa1	100% CP021765_03155	IS3 ssgr IS150	2458070	2457642	429
CRMRs218	CP021765_02459	99.64% ISRso11_aa2	99.64% CP021765_03154	IS3 ssgr IS150	2614254	2613418	837
CRMRs218	CP021765_02460	100% ISRso11_aa1	100% CP021765_03155	IS3 ssgr IS150	2614784	2614251	534
CRMRs218	CP021765_03154	100% ISRso11_aa2	100% CP021765_02311	IS3 ssgr IS150	3327542	3326706	837
CRMRs218	CP021765_03155	100% ISRso11_aa1	100% CP021765_02460	IS3 ssgr IS150	3328072	3327539	534
CRMRs218	CP021765_01472	98.36% ISRso10_aa1	100% CP021765_01880	IS3 ssgr IS2	1538781	1539173	393
CRMRs218	CP021765_01473	99.64% ISRso10_aa2	99.64% CP021765_01879	IS3 ssgr IS2	1539170	1540018	849
CRMRs218	CP021765_01879	99.29% ISRso10_aa2	99.64% CP021765_01473	IS3 ssgr IS2	2002403	2001555	849
CRMRs218	CP021765_01880	98.36% ISRso10_aa1	100% CP021765_01472	IS3 ssgr IS2	2002792	2002400	393
CRMRs218	CP021765_02379	98.58% ISRso10_aa2	98.93% CP021765_01473	IS3 ssgr IS2	2531444	2530596	849
CRMRs218	CP021765_02380	98.86% ISRso10_aa1	97.72% CP021765_01880	IS3 ssgr IS2	2531737	2531441	297
CRMRs218	CP021765_02360	89.23% IS222_aa2	57.69% CP021765_02313	IS3 ssgr IS3	2515013	2514342	672
CRMRs218	CP021765_02361	87.25% IS222_aa1	No hit	IS3 ssgr IS3	2515504	2515196	309
CRMRs218	CP021765_00429	96.10% ISRso16_aa2	85.18% CP021765_02755	IS3 ssgr IS407	451724	452023	300
CRMRs218	CP021765_00641	88.09% ISAtu5_aa1	80.23% CP021765_02457	IS3 ssgr IS407	671045	670785	261
CRMRs218	CP021765_00846	98.90% ISRso14_aa2	100% CP021765_02456	IS3 ssgr IS407	870532	869984	549
CRMRs218	CP021765_00847	100% ISRso14_aa1	100% CP021765_02457	IS3 ssgr IS407	871101	870838	264
CRMRs218	CP021765_01476	100% ISRso12_aa1	74.69% CP021765_00641	IS3 ssgr IS407	1541746	1542012	267
CRMRs218	CP021765_01477	100% ISRso12_aa2	100% CP021765_02755	IS3 ssgr IS407	1542162	1542866	705
CRMRs218	CP021765_01531	98.90% ISRso14_aa2	100% CP021765_02456	IS3 ssgr IS407	1597471	1596923	549
CRMRs218	CP021765_01532	100% ISRso14_aa1	100% CP021765_02457	IS3 ssgr IS407	1598040	1597777	264
CRMRs218	CP021765_02456	98.90% ISRso14_aa2	100% CP021765_01531	IS3 ssgr IS407	2612429	2611881	549
CRMRs218	CP021765_02457	100% ISRso14_aa1	100% CP021765_01532	IS3 ssgr IS407	2612998	2612735	264
CRMRs218	CP021765_02755	100% ISRso12_aa2	100% CP021765_01477	IS3 ssgr IS407	2911959	2911255	705
CRMRs218	CP021765_01589	76.34% ISBcen21_aa1	54.54% CP021765_02314	IS3 ssgr IS51	1661244	1661531	288
CRMRs218	CP021765_01522	100% ISRso13_aa1	100% CP021765_03306	IS4 ssgr IS4	1590586	1589345	1.242
CRMRs218	CP021765_01915	100% ISRso13_aa1	100% CP021765_03306	IS4 ssgr IS4	2047952	2049286	1.335
CRMRs218	CP021765_03237	100% ISRso13_aa1	100% CP021765_03306	IS4 ssgr IS4	3427869	3429203	1.335
CRMRs218	CP021765_03306	100% ISRso13_aa1	100% CP021765_03237	IS4 ssgr IS4	3500459	3499125	1.335
CRMRs218	CP021765_00210	95.62% ISRso1_aa1	99.27% CP021765_03417	IS5	231889	232788	900
CRMRs218	CP021765_01026	97.21% ISRso1_aa1	95.21% CP021765_03417	IS5	1060261	1059506	756

CRMRs218	CP021765_03264	96.35% ISRso1_aa1	96.35% CP021765_03417	IS5	3460936	3461760	825
CRMRs218	CP021765_03274	88.15% ISRso1_aa1	89.47% CP021765_03264	IS5	3472497	3473306	810
CRMRs218	CP021765_03284	94.87% ISRso1_aa1	97.84% CP021765_00210	IS5	3483646	3484488	843
CRMRs218	CP021765_03417	95.98% ISRso1_aa1	99.27% CP021765_00210	IS5	3613190	3612366	825
CRMRs218	CP021765_00911	76.56% ISCARN14_aa1	49.26% CP021765_00210	IS5 ssgr IS1031	940716	939538	1.179
CRMRs218	CP021765_00861	100% ISRso9_aa1	100% CP021765_03488	IS5 ssgr IS5	883345	884667	1.323
CRMRs218	CP021765_01754	99.68% ISRso18_aa1	No hit	IS5 ssgr IS5	1821225	1820260	966
CRMRs218	CP021765_03191	100% ISRso9_aa1	100% CP021765_03488	IS5 ssgr IS5	3367383	3366061	1.323
CRMRs218	CP021765_03214	100% ISRso9_aa1	100% CP021765_03488	IS5 ssgr IS5	3396972	3395650	1.323
CRMRs218	CP021765_03488	100% ISRso9_aa1	100% CP021765_03214	IS5 ssgr IS5	3697368	3696046	1.323
CRMRs218	CP021765_00103	100% ISRso5_aa1	100% CP021765_03461	IS630	118044	119135	1.092
CRMRs218	CP021765_00109	99.72% ISRso5_aa1	99.72% CP021765_03461	IS630	127969	12906	1.092
CRMRs218	CP021765_00655	100% ISRso5_aa1	100% CP021765_03461	IS630	683464	684555	1.092
CRMRs218	CP021765_02223	100% ISRso5_aa1	100% CP021765_03461	IS630	2360302	2361393	1.092
CRMRs218	CP021765_03461	100% ISRso5_aa1	100% CP021765_02223	IS630	3660831	3659740	1.092
CRMRs218	CP021765_00053	48.25% ISWz1_aa1	53.14% CP021765_02595	IS91	5836	57371	990
CRMRs218	CP021765_01595	40.16% ISShvi3_aa1	52.96% CP021765_01702	IS91	1670709	1669645	1.065
CRMRs218	CP021765_02595	48.98% ISMno23_aa1	52.10% CP021765_00053	IS91	2752251	2753177	927
CRMRs218	CP021765_02624	100% ISRso15_aa1	No hit	ISL3	2780421	2781641	1.221
CRMRs218	CP021765_02671	77.94% ISSm4_aa2	No hit	ISL3	2824871	2823804	1.068
CRMRs218	CP021765_03464	38.94% ISKpn25_aa1	No hit	ISL3	3666102	3664657	1.446
CRMRs218	CP021765_01894	64.16% ISKpn21_aa1	92.5% CP021765_01895	ISNCY ssgr IS1202	2023666	2023274	393
CRMRs218	CP021765_01895	71.39% ISKpn21_aa1	92.5% CP021765_01894	ISNCY ssgr IS1202	2025295	2023820	1.476
CRMRs218	CP021765_00605	52.74% ISMpo10_aa1	54.92% CP021765_00149	Tn3	636592	637098	507
CRMRs218	CP021765_00909	53.79% ISMpo10_aa3	71.09% CP021765_03311	Tn3	937596	938975	1.38
CRMRs218	CP021765_03240	61.81% ISMpo10_aa3	96.96% CP021765_03311	Tn3	3430707	3432095	1.389
CRMRs218	CP021765_03311	55.34% ISMpo10_aa3	96.96% CP021765_03240	Tn3	3503119	3504507	1.389
YC40M	CP015851_00321	95.76% ISBma3_aa1	100% CP015851_01417	IS110	424574	425779	1.206
YC40M	CP015851_01306	95.76% ISBma3_aa1	100% CP015851_01417	IS110	1667900	1669105	1.206
YC40M	CP015851_01417	95.76% ISBma3_aa1	100% CP015851_01306	IS110	1794105	1795310	1.206
YC40M	CP015851_00806	82.67% IS1383_aa1	100% CP015851_01211	IS110 ssgr IS1111	1024765	1023764	1.002
YC40M	CP015851_01211	82.67% IS1383_aa1	100% CP015851_00806	IS110 ssgr IS1111	1554181	1555182	1.002
YC40M	CP015851_01233	88.75% ISBcen4_aa1	100% CP015851_01278	IS110 ssgr IS1111	1573460	1572441	1.02
YC40M	CP015851_01278	88.75% ISBcen4_aa1	100% CP015851_01233	IS110 ssgr IS1111	1634545	1633526	1.02
YC40M	CP015851_01504	83.75% ISBusp4_aa1	No hit	IS1182	1889376	1887931	1.446
YC40M	CP015851_00197	94.07% ISBcen18_aa1	No hit	IS256	288978	287701	1.278
YC40M	CP015851_01232	97.14% ISRso10_aa2	44.49% CP015851_00346	IS3 ssgr IS2	1571907	1571215	693
YC40M	CP015851_01234	94.68% ISRso10_aa1	No hit	IS3 ssgr IS2	1573815	1573435	381
YC40M	CP015851_00343	93.65% ISRso16_aa2	100% CP015851_00346	IS3 ssgr IS407	444982	445494	513
YC40M	CP015851_00346	93.30% ISRso16_aa2	100% CP015851_00343	IS3 ssgr IS407	447124	44645	675
YC40M	CP015851_01451	98.86% ISSod2_aa1	62.12% CP015851_00347	IS3 ssgr IS407	1834370	1834636	267
YC40M	CP015851_01452	84.79% ISAs22_aa3	58.42% CP015851_00346	IS3 ssgr IS407	1834663	1835457	795

YC40M	CP015851_00418	81.52% ISAisp2_aa1	No hit	IS3 ssgr IS51	539281	539559	279
YC40M	CP015851_00419	89.34% ISAisp2_aa2	42.15% CP015851_00346	IS3 ssgr IS51	539556	540431	876
YC40M	CP015851_00196	89.55% ISHar5_aa1	59.25% CP015851_00976	IS30	287429	287692	264
YC40M	CP015851_00976	65.95% IS1382_aa1	59.25% CP015851_00196	IS30	1261976	1260960	1.017
YC40M	CP015851_01351	61.68% ISCro3_aa1	No hit	IS4	1722548	1723876	1.329
YC40M	CP015851_00972	76.74% ISAzo5_aa1	100% CP015851_01462	IS4 ssgr IS50	1257110	1255848	1.263
YC40M	CP015851_01462	76.74% ISAzo5_aa1	100% CP015851_00972	IS4 ssgr IS50	1846246	1844984	1.263
YC40M	CP015851_00881	96.59% ISRso1_aa1	95.45% CP015851_00971	IS5	1148096	1148437	342
YC40M	CP015851_00971	97.81% ISRso1_aa1	95.45% CP015851_00881	IS5	1255776	1254952	825
YC40M	CP015851_01193	96.96% ISRso1_aa1	96.96% CP015851_00971	IS5	1534018	1534218	201
YC40M	CP015851_00249	100% IS1421_aa1	100% CP015851_01256	IS5 ssgr IS427	347659	347255	405
YC40M	CP015851_00807	100% IS1421_aa1	100% CP015851_01256	IS5 ssgr IS427	1025702	1025298	405
YC40M	CP015851_00975	100% IS1421_aa1	100% CP015851_01256	IS5 ssgr IS427	1260914	1260510	405
YC40M	CP015851_01129	100% IS1421_aa1	100% CP015851_01256	IS5 ssgr IS427	1455344	1454940	405
YC40M	CP015851_01235	99.25% IS1421_aa1	99.25% CP015851_01256	IS5 ssgr IS427	1574635	1574231	405
YC40M	CP015851_01256	100% IS1421_aa1	100% CP015851_01129	IS5 ssgr IS427	1605565	1605161	405
YC40M	CP015851_01438	61.34% ISNeu3_aa3	48.78% CP015851_01256	IS5 ssgr IS427	1818352	1818735	384
YC40M	CP015851_00160	99.06% IS1405_aa1	100% CP015851_01078	IS5 ssgr IS5	231912	232877	966
YC40M	CP015851_00200	94.51% IS1021_aa1	100% CP015851_01525	IS5 ssgr IS5	290388	291374	987
YC40M	CP015851_00238	99.06% IS1405_aa1	100% CP015851_01078	IS5 ssgr IS5	336998	336033	966
YC40M	CP015851_00352	99.06% IS1405_aa1	100% CP015851_01078	IS5 ssgr IS5	454425	45346	966
YC40M	CP015851_00417	94.51% IS1021_aa1	100% CP015851_01525	IS5 ssgr IS5	538137	539123	987
YC40M	CP015851_00431	94.51% IS1021_aa1	100% CP015851_01525	IS5 ssgr IS5	560484	559498	987
YC40M	CP015851_00469	99.06% IS1405_aa1	100% CP015851_01078	IS5 ssgr IS5	607979	608944	966
YC40M	CP015851_00506	94.51% IS1021_aa1	100% CP015851_01525	IS5 ssgr IS5	649525	648539	987
YC40M	CP015851_00598	99.06% IS1405_aa1	100% CP015851_01078	IS5 ssgr IS5	762274	761309	966
YC40M	CP015851_00621	94.51% IS1021_aa1	100% CP015851_01525	IS5 ssgr IS5	799977	800963	987
YC40M	CP015851_00622	89.55% IS1021_aa1	100% CP015851_01525	IS5 ssgr IS5	8024	801327	1.074
YC40M	CP015851_00630	94.51% IS1021_aa1	100% CP015851_01525	IS5 ssgr IS5	811004	810018	987
YC40M	CP015851_00821	99.06% IS1405_aa1	100% CP015851_01078	IS5 ssgr IS5	1035823	1034858	966
YC40M	CP015851_00973	94.51% IS1021_aa1	100% CP015851_01525	IS5 ssgr IS5	1258164	1259150	987
YC40M	CP015851_01076	92.71% IS1021_aa1	100% CP015851_01525	IS5 ssgr IS5	1387200	1386745	456
YC40M	CP015851_01077	93.02% IS1021_aa1	97.09% CP015851_01525	IS5 ssgr IS5	1387732	1387154	579
YC40M	CP015851_01078	99.06% IS1405_aa1	100% CP015851_00821	IS5 ssgr IS5	1387890	1388855	966
YC40M	CP015851_01114	94.51% IS1021_aa1	100% CP015851_01525	IS5 ssgr IS5	1436592	1437578	987
YC40M	CP015851_01224	94.51% IS1021_aa1	100% CP015851_01525	IS5 ssgr IS5	1564654	1565640	987
YC40M	CP015851_01252	94.51% IS1021_aa1	100% CP015851_01525	IS5 ssgr IS5	1592038	1591052	987
YC40M	CP015851_01432	99.06% IS1405_aa1	99.37% CP015851_01078	IS5 ssgr IS5	1813000	1813965	966
YC40M	CP015851_01525	94.51% IS1021_aa1	100% CP015851_01252	IS5 ssgr IS5	1907273	1906287	987
YC40M	CP015851_01226	85.71% ISAzo9_aa2	No hit	IS630	1566716	1566384	333
YC40M	CP015851_01230	85.03% ISAzo9_aa2	No hit	IS630	1570071	1569256	816
YC40M	CP015851_00501	56.95% ISRm2_aa1	100% CP015851_01491	IS66	642906	643388	483
YC40M	CP015851_00502	73.27% ISRm2_aa2	100% CP015851_01492	IS66	643385	643738	354

YC40M	CP015851_00503	59.58% ISAeh1_aa2	100% CP015851_01229	IS66	643771	645327	1.557
YC40M	CP015851_01227	56.95% ISRm2_aa1	100% CP015851_01491	IS66	1566838	1567320	483
YC40M	CP015851_01228	73.27% ISRm2_aa2	100% CP015851_01492	IS66	1567317	1567670	354
YC40M	CP015851_01229	59.58% ISAeh1_aa2	100% CP015851_00503	IS66	1567703	1569259	1.557
YC40M	CP015851_01491	56.95% ISRm2_aa1	100% CP015851_01227	IS66	1878843	1879325	483
YC40M	CP015851_01492	73.27% ISRm2_aa2	100% CP015851_01228	IS66	1879322	1879675	354
YC40M	CP015851_01493	61.53% ISPpu13_aa2	100% CP015851_01229	IS66	1879708	1881264	1.557
YC40M	CP015851_00978	60.93% ISAba32_aa1	68.57% CP015851_00979	ISNCY ssgr IS1202	1263543	1263328	216
YC40M	CP015851_00979	72.27% ISKpn21_aa1	68.57% CP015851_00978	ISNCY ssgr IS1202	1265160	1263685	1.476
YC40M	CP015851_01440	0% newcandidate	not_found	New_Family	1821394	1821239	156
SN82F48	IS_de47452f_01240	98.00% ISRme9_aa2	No hit	IS21	1369799	1369044	756
SN82F48	IS_de47452f_01241	99.22% ISRme9_aa1	No hit	IS21	1371360	1369807	1.554
SN82F48	IS_de47452f_01144	98.05% ISMca5_aa1	100% IS_de47452f_01206	IS256	1255219	1253954	1.266
SN82F48	IS_de47452f_01172	98.05% ISMca5_aa1	100% IS_de47452f_01206	IS256	1289222	1290487	1.266
SN82F48	IS_de47452f_01206	98.05% ISMca5_aa1	100% IS_de47452f_01172	IS256	1329375	1330640	1.266
SN82F48	IS_de47452f_01242	82.73% ISAzo10_aa2	No hit	IS3 ssgr IS3	1372562	1371465	1.098
SN82F48	IS_de47452f_01243	80.55% ISAzo10_aa1	No hit	IS3 ssgr IS3	1372993	1372559	435
SN82F48	IS_de47452f_00323	80.89% ISBcen19_aa3	No hit	IS66	324455	322926	1.53
SN82F48	IS_de47452f_00324	77.08% ISSal1_aa2	96.29% IS_de47452f_01171	IS66	324808	324518	291
SN82F48	IS_de47452f_00325	65.38% ISBcen19_aa1	100% IS_de47452f_01173	IS66	325221	32485	372
SN82F48	IS_de47452f_01173	65.38% ISBcen19_aa1	100% IS_de47452f_00325	IS66	1290994	1290623	372
SN82F48	IS_de47452f_01225	43.64% ISWz1_aa1	38.29% IS_de47452f_00542	IS91	1354640	1352910	1.731
SN82F48	IS_de47452f_00346	77.85% ISIde1_aa1	100% IS_de47452f_01081	ISL3	344631	345926	1.296
SN82F48	IS_de47452f_00596	77.85% ISIde1_aa1	100% IS_de47452f_01081	ISL3	606664	607959	1.296
SN82F48	IS_de47452f_01031	78.08% ISIde1_aa1	99.07% IS_de47452f_01081	ISL3	1116369	1117664	1.296
SN82F48	IS_de47452f_01081	77.85% ISIde1_aa1	100% IS_de47452f_00596	ISL3	1180404	1181699	1.296
SN82F48	IS_de47452f_01132	98.17% ISPa38_aa2	43.59% IS_de47452f_01223	Tn3	1246144	1243178	2.967
SN82F48	IS_de47452f_01133	95.16% TnAs3_aa1	No hit	Tn3	1246707	1246147	561
SN82F48	IS_de47452f_01157	53.14% TnShfr1_aa1	53.42% IS_de47452f_01180	Tn3	1272949	1271645	1.305
SN82F48	IS_de47452f_01180	78.08% TnShfr1_aa1	52.51% IS_de47452f_01157	Tn3	1303797	1302823	975
SN82F48	IS_de47452f_01222	75.55% ISPa43_aa1	No hit	Tn3	1347685	1348644	960
SN82F48	IS_de47452f_01223	65.81% IS882_aa1	43.59% IS_de47452f_01132	Tn3	1351704	1348771	2.934
SN83A39	IS_c6bb695b_01332	81.14% ISSpwi2_aa1	No hit	IS256	1463719	1462619	1.101
SN83A39	IS_c6bb695b_00971	89.41% ISAtu5_aa1	No hit	IS3 ssgr IS407	1082597	1082857	261
SN83A39	IS_c6bb695b_00972	74% ISDet2_aa2	No hit	IS3 ssgr IS407	1082896	1083708	813
SN83A39	IS_c6bb695b_00903	41.66% ISShvi3_aa1	40.39% IS_c6bb695b_00208	IS91	1014832	1016028	1.197
SN83A39	IS_c6bb695b_00902	59.48% ISKpn21_aa2	No hit	ISNCY ssgr IS1202	1014144	1014785	642
SN83A39	IS_c6bb695b_00936	70.50% ISKpn21_aa1	No hit	ISNCY ssgr IS1202	1047445	1048917	1.473
SN83A39	IS_c6bb695b_00886	0% newcandidate	not_found	New_Family	998098	998496	399
SN83A39	IS_c6bb695b_00829	66.49% IS882_aa1	92.23% IS_c6bb695b_00919	Tn3	93949	942417	2.928
SN83A39	IS_c6bb695b_00830	66.44% ISPa43_aa1	87.13% IS_c6bb695b_00920	Tn3	943762	942455	1.308

SN83A39	IS_c6bb695b_00880	78.34% TnShfr1_aa1	47.03% IS_c6bb695b_00895	Tn3	992449	993423	975
SN83A39	IS_c6bb695b_00895	48.91% TnShfr1_aa1	47.03% IS_c6bb695b_00880	Tn3	1009375	1010301	927
SN83A39	IS_c6bb695b_00913	53.84% ISPa43_aa2	No hit	Tn3	1026095	1027708	1.614
SN83A39	IS_c6bb695b_00919	72.59% IS882_aa1	92.23% IS_c6bb695b_00829	Tn3	1030826	1031461	636
SN83A39	IS_c6bb695b_00920	75.87% ISPa43_aa1	87.09% IS_c6bb695b_00830	Tn3	1032523	1031561	963
SEPPX05	IS_12c9bc1e_01115	95.76% ISBma3_aa1	No hit	IS110	1311220	1312425	1.206
SEPPX05	IS_12c9bc1e_00032	99.23% ISRso19_aa2	100% IS_12c9bc1e_01686	IS21	34048	3326	789
SEPPX05	IS_12c9bc1e_00033	97.58% ISRso19_aa1	100% IS_12c9bc1e_01687	IS21	3504	34045	996
SEPPX05	IS_12c9bc1e_00038	97.58% ISRso19_aa1	100% IS_12c9bc1e_01687	IS21	40216	41211	996
SEPPX05	IS_12c9bc1e_00039	99.23% ISRso19_aa2	100% IS_12c9bc1e_01686	IS21	41208	41996	789
SEPPX05	IS_12c9bc1e_00122	97.58% ISRso19_aa1	100% IS_12c9bc1e_01687	IS21	141948	142943	996
SEPPX05	IS_12c9bc1e_00123	99.23% ISRso19_aa2	100% IS_12c9bc1e_01686	IS21	14294	143728	789
SEPPX05	IS_12c9bc1e_00159	99.23% ISRso19_aa2	100% IS_12c9bc1e_01686	IS21	17823	177442	789
SEPPX05	IS_12c9bc1e_00160	92.70% ISRso19_aa1	100% IS_12c9bc1e_01687	IS21	178517	178227	291
SEPPX05	IS_12c9bc1e_00161	94.44% ISRme9_aa2	100% IS_12c9bc1e_01299	IS21	179416	178658	759
SEPPX05	IS_12c9bc1e_00162	93.42% ISRme9_aa1	100% IS_12c9bc1e_01300	IS21	180978	179425	1.554
SEPPX05	IS_12c9bc1e_00163	99.54% ISRso19_aa1	100% IS_12c9bc1e_01687	IS21	181914	181222	693
SEPPX05	IS_12c9bc1e_00320	99.23% ISRso19_aa2	100% IS_12c9bc1e_01686	IS21	363611	362823	789
SEPPX05	IS_12c9bc1e_00321	97.58% ISRso19_aa1	100% IS_12c9bc1e_01687	IS21	364603	363608	996
SEPPX05	IS_12c9bc1e_00398	99.23% ISRso19_aa2	100% IS_12c9bc1e_01686	IS21	462966	462178	789
SEPPX05	IS_12c9bc1e_00399	97.58% ISRso19_aa1	100% IS_12c9bc1e_01687	IS21	463958	462963	996
SEPPX05	IS_12c9bc1e_00405	62.38% ISRme9_aa1	63.46% IS_12c9bc1e_01300	IS21	47111	471457	348
SEPPX05	IS_12c9bc1e_00496	93.42% ISRme9_aa1	100% IS_12c9bc1e_01300	IS21	582244	583797	1.554
SEPPX05	IS_12c9bc1e_00497	94.44% ISRme9_aa2	100% IS_12c9bc1e_01299	IS21	583806	584564	759
SEPPX05	IS_12c9bc1e_00654	93.42% ISRme9_aa1	100% IS_12c9bc1e_01300	IS21	781812	783365	1.554
SEPPX05	IS_12c9bc1e_00655	94.44% ISRme9_aa2	100% IS_12c9bc1e_01299	IS21	783374	784132	759
SEPPX05	IS_12c9bc1e_00678	97.58% ISRso19_aa1	100% IS_12c9bc1e_01687	IS21	804482	805477	996
SEPPX05	IS_12c9bc1e_00679	99.23% ISRso19_aa2	100% IS_12c9bc1e_01686	IS21	805474	806262	789
SEPPX05	IS_12c9bc1e_00723	97.58% ISRso19_aa1	100% IS_12c9bc1e_01687	IS21	838887	839882	996
SEPPX05	IS_12c9bc1e_00724	99.23% ISRso19_aa2	100% IS_12c9bc1e_01686	IS21	839879	840667	789
SEPPX05	IS_12c9bc1e_00773	99.23% ISRso19_aa2	100% IS_12c9bc1e_01686	IS21	905733	904945	789
SEPPX05	IS_12c9bc1e_00774	97.58% ISRso19_aa1	100% IS_12c9bc1e_01687	IS21	906725	90573	996
SEPPX05	IS_12c9bc1e_00900	99.23% ISRso19_aa2	100% IS_12c9bc1e_01686	IS21	1061485	1060697	789
SEPPX05	IS_12c9bc1e_00901	97.58% ISRso19_aa1	100% IS_12c9bc1e_01687	IS21	1062477	1061482	996
SEPPX05	IS_12c9bc1e_00902	93.42% ISRme9_aa1	100% IS_12c9bc1e_01300	IS21	1063023	1064576	1.554
SEPPX05	IS_12c9bc1e_00903	94.44% ISRme9_aa2	100% IS_12c9bc1e_01299	IS21	1064585	1065343	759
SEPPX05	IS_12c9bc1e_00907	99.23% ISRso19_aa2	100% IS_12c9bc1e_01686	IS21	1068184	1067396	789
SEPPX05	IS_12c9bc1e_00908	97.58% ISRso19_aa1	100% IS_12c9bc1e_01687	IS21	1069176	1068181	996
SEPPX05	IS_12c9bc1e_01067	99.23% ISRso19_aa2	100% IS_12c9bc1e_01686	IS21	1265332	1264544	789
SEPPX05	IS_12c9bc1e_01068	97.58% ISRso19_aa1	100% IS_12c9bc1e_01687	IS21	1266324	1265329	996
SEPPX05	IS_12c9bc1e_01155	99.23% ISRso19_aa2	100% IS_12c9bc1e_01686	IS21	1364911	1364123	789
SEPPX05	IS_12c9bc1e_01156	97.58% ISRso19_aa1	100% IS_12c9bc1e_01687	IS21	1365903	1364908	996

SEPPX05	IS_12c9bc1e_01290	97.58% ISRso19_aa1	100% IS_12c9bc1e_01687	IS21	1534916	1535911	996
SEPPX05	IS_12c9bc1e_01291	99.23% ISRso19_aa2	100% IS_12c9bc1e_01686	IS21	1535908	1536696	789
SEPPX05	IS_12c9bc1e_01299	94.44% ISRme9_aa2	100% IS_12c9bc1e_00903	IS21	1545731	1544973	759
SEPPX05	IS_12c9bc1e_01300	93.42% ISRme9_aa1	100% IS_12c9bc1e_00902	IS21	1547293	1545740	1.554
SEPPX05	IS_12c9bc1e_01475	97.58% ISRso19_aa1	100% IS_12c9bc1e_01687	IS21	1750752	1751747	996
SEPPX05	IS_12c9bc1e_01476	99.23% ISRso19_aa2	100% IS_12c9bc1e_01686	IS21	1751744	1752532	789
SEPPX05	IS_12c9bc1e_01562	99.23% ISRso19_aa2	100% IS_12c9bc1e_01686	IS21	1876497	1875709	789
SEPPX05	IS_12c9bc1e_01563	97.58% ISRso19_aa1	100% IS_12c9bc1e_01687	IS21	1877489	1876494	996
SEPPX05	IS_12c9bc1e_01686	99.23% ISRso19_aa2	100% IS_12c9bc1e_01562	IS21	2020862	2020074	789
SEPPX05	IS_12c9bc1e_01687	97.58% ISRso19_aa1	100% IS_12c9bc1e_01563	IS21	2021854	2020859	996
SEPPX05	IS_12c9bc1e_00189	46.20% ISAzs33_aa2	100% IS_12c9bc1e_01640	IS3 ssgr IS150	206395	207216	822
SEPPX05	IS_12c9bc1e_00420	46.20% ISAzs33_aa2	100% IS_12c9bc1e_01640	IS3 ssgr IS150	484062	484883	822
SEPPX05	IS_12c9bc1e_00749	46.20% ISAzs33_aa2	100% IS_12c9bc1e_01640	IS3 ssgr IS150	870905	871726	822
SEPPX05	IS_12c9bc1e_00771	46.20% ISAzs33_aa2	100% IS_12c9bc1e_01640	IS3 ssgr IS150	9043	903479	822
SEPPX05	IS_12c9bc1e_01552	46.57% ISAzs33_aa2	99.63% IS_12c9bc1e_01640	IS3 ssgr IS150	1865481	1864660	822
SEPPX05	IS_12c9bc1e_01640	46.20% ISAzs33_aa2	100% IS_12c9bc1e_00771	IS3 ssgr IS150	1965690	1964869	822
SEPPX05	IS_12c9bc1e_00023	98.48% ISButh1_aa1	100% IS_12c9bc1e_01637	IS3 ssgr IS2	26149	2655	402
SEPPX05	IS_12c9bc1e_00024	93.88% ISButh1_aa2	100% IS_12c9bc1e_01638	IS3 ssgr IS2	26547	27383	837
SEPPX05	IS_12c9bc1e_00051	92.80% ISButh1_aa2	100% IS_12c9bc1e_01293	IS3 ssgr IS2	5522	54384	837
SEPPX05	IS_12c9bc1e_00052	95.48% ISButh1_aa1	100% IS_12c9bc1e_01292	IS3 ssgr IS2	55618	55217	402
SEPPX05	IS_12c9bc1e_00081	95.48% ISButh1_aa1	100% IS_12c9bc1e_01292	IS3 ssgr IS2	99847	100248	402
SEPPX05	IS_12c9bc1e_00082	92.80% ISButh1_aa2	100% IS_12c9bc1e_01293	IS3 ssgr IS2	100245	101081	837
SEPPX05	IS_12c9bc1e_00241	95.48% ISButh1_aa1	100% IS_12c9bc1e_01292	IS3 ssgr IS2	271896	272297	402
SEPPX05	IS_12c9bc1e_00242	92.80% ISButh1_aa2	100% IS_12c9bc1e_01293	IS3 ssgr IS2	272294	27313	837
SEPPX05	IS_12c9bc1e_00323	92.80% ISButh1_aa2	100% IS_12c9bc1e_01293	IS3 ssgr IS2	367892	367056	837
SEPPX05	IS_12c9bc1e_00324	95.48% ISButh1_aa1	100% IS_12c9bc1e_01292	IS3 ssgr IS2	36829	367889	402
SEPPX05	IS_12c9bc1e_00498	92.80% ISButh1_aa2	100% IS_12c9bc1e_01293	IS3 ssgr IS2	586282	585446	837
SEPPX05	IS_12c9bc1e_00499	95.48% ISButh1_aa1	100% IS_12c9bc1e_01292	IS3 ssgr IS2	58668	586279	402
SEPPX05	IS_12c9bc1e_00673	92.80% ISButh1_aa2	100% IS_12c9bc1e_01293	IS3 ssgr IS2	802296	80146	837
SEPPX05	IS_12c9bc1e_00674	95.48% ISButh1_aa1	100% IS_12c9bc1e_01292	IS3 ssgr IS2	802694	802293	402
SEPPX05	IS_12c9bc1e_00775	96.06% ISButh1_aa1	100% IS_12c9bc1e_01292	IS3 ssgr IS2	906799	907206	408
SEPPX05	IS_12c9bc1e_00776	92.80% ISButh1_aa2	100% IS_12c9bc1e_01293	IS3 ssgr IS2	907203	908039	837
SEPPX05	IS_12c9bc1e_00898	91.81% ISButh1_aa2	100% IS_12c9bc1e_01293	IS3 ssgr IS2	1060453	1060109	345
SEPPX05	IS_12c9bc1e_01168	92.80% ISButh1_aa2	100% IS_12c9bc1e_01293	IS3 ssgr IS2	1377862	1377026	837
SEPPX05	IS_12c9bc1e_01169	95.48% ISButh1_aa1	100% IS_12c9bc1e_01292	IS3 ssgr IS2	1378260	1377859	402
SEPPX05	IS_12c9bc1e_01292	95.48% ISButh1_aa1	100% IS_12c9bc1e_01169	IS3 ssgr IS2	1538344	1538745	402
SEPPX05	IS_12c9bc1e_01293	92.80% ISButh1_aa2	100% IS_12c9bc1e_01168	IS3 ssgr IS2	1538742	1539578	837
SEPPX05	IS_12c9bc1e_01637	98.48% ISButh1_aa1	100% IS_12c9bc1e_00023	IS3 ssgr IS2	1962318	1962719	402
SEPPX05	IS_12c9bc1e_01638	93.88% ISButh1_aa2	100% IS_12c9bc1e_00024	IS3 ssgr IS2	1962716	1963552	837
SEPPX05	IS_12c9bc1e_00088	100% ISRso14_aa2	64.89% IS_12c9bc1e_01606	IS3 ssgr IS407	107851	107303	549
SEPPX05	IS_12c9bc1e_00089	100% ISRso14_aa1	100% IS_12c9bc1e_00899	IS3 ssgr IS407	10842	108157	264
SEPPX05	IS_12c9bc1e_01606	93.43% ISRso16_aa2	82.87% IS_12c9bc1e_01610	IS3 ssgr IS407	1936428	1936880	453
SEPPX05	IS_12c9bc1e_01610	93.75% ISRso16_aa2	82.87% IS_12c9bc1e_01606	IS3 ssgr IS407	1939436	1938951	486

SEPPX05	IS_12c9bc1e_00421	63.10% ISCro3_aa1	98.36% IS_12c9bc1e_00793	IS4	485915	484872	1.044
SEPPX05	IS_12c9bc1e_00793	58.27% ISCro6_aa1	98.36% IS_12c9bc1e_00421	IS4 ssgr IS4	926144	926644	501
SEPPX05	IS_12c9bc1e_00694	39.53% ISAzs36_aa1	No hit	IS481	815818	817308	1.491
SEPPX05	IS_12c9bc1e_00695	45.84% ISAzs36_aa2	No hit	IS481	817308	818594	1.287
SEPPX05	IS_12c9bc1e_00653	98.38% ISRso1_aa1	No hit	IS5	780982	781584	603
SEPPX05	IS_12c9bc1e_00656	100% ISRso1_aa1	96.92% IS_12c9bc1e_00707	IS5	784271	784498	228
SEPPX05	IS_12c9bc1e_00707	96.96% ISRso1_aa1	96.92% IS_12c9bc1e_00656	IS5	82659	82639	201
SEPPX05	IS_12c9bc1e_00041	100% IS1421_aa1	100% IS_12c9bc1e_01702	IS5 ssgr IS427	43042	43446	405
SEPPX05	IS_12c9bc1e_00151	100% IS1421_aa1	100% IS_12c9bc1e_01702	IS5 ssgr IS427	174263	173859	405
SEPPX05	IS_12c9bc1e_00155	87.15% IS1421_aa1	100% IS_12c9bc1e_01635	IS5 ssgr IS427	175827	175456	372
SEPPX05	IS_12c9bc1e_00277	100% IS1421_aa1	100% IS_12c9bc1e_01702	IS5 ssgr IS427	31071	310306	405
SEPPX05	IS_12c9bc1e_00296	87.15% IS1421_aa1	100% IS_12c9bc1e_01635	IS5 ssgr IS427	340222	339851	372
SEPPX05	IS_12c9bc1e_00316	100% IS1421_aa1	100% IS_12c9bc1e_01702	IS5 ssgr IS427	361056	36146	405
SEPPX05	IS_12c9bc1e_00383	100% IS1421_aa1	100% IS_12c9bc1e_01702	IS5 ssgr IS427	441898	441494	405
SEPPX05	IS_12c9bc1e_00477	100% IS1421_aa1	100% IS_12c9bc1e_01702	IS5 ssgr IS427	554527	554123	405
SEPPX05	IS_12c9bc1e_00510	100% IS1421_aa1	100% IS_12c9bc1e_01702	IS5 ssgr IS427	595963	596367	405
SEPPX05	IS_12c9bc1e_00615	100% IS1421_aa1	100% IS_12c9bc1e_01702	IS5 ssgr IS427	739173	739577	405
SEPPX05	IS_12c9bc1e_00651	100% IS1421_aa1	100% IS_12c9bc1e_01702	IS5 ssgr IS427	780045	779641	405
SEPPX05	IS_12c9bc1e_00961	100% IS1421_aa1	100% IS_12c9bc1e_01702	IS5 ssgr IS427	1130562	1130158	405
SEPPX05	IS_12c9bc1e_01008	87.15% IS1421_aa1	100% IS_12c9bc1e_01635	IS5 ssgr IS427	1179421	1179792	372
SEPPX05	IS_12c9bc1e_01120	100% IS1421_aa1	100% IS_12c9bc1e_01702	IS5 ssgr IS427	1317506	1317910	405
SEPPX05	IS_12c9bc1e_01607	100% IS1421_aa1	100% IS_12c9bc1e_01702	IS5 ssgr IS427	1936895	1937299	405
SEPPX05	IS_12c9bc1e_01635	87.15% IS1421_aa1	100% IS_12c9bc1e_01008	IS5 ssgr IS427	1961512	1961883	372
SEPPX05	IS_12c9bc1e_01689	98.50% IS1421_aa1	98.50% IS_12c9bc1e_01702	IS5 ssgr IS427	2025059	2024655	405
SEPPX05	IS_12c9bc1e_01700	87.80% IS1421_aa1	100% IS_12c9bc1e_01635	IS5 ssgr IS427	2039865	2040116	252
SEPPX05	IS_12c9bc1e_01702	100% IS1421_aa1	100% IS_12c9bc1e_01607	IS5 ssgr IS427	2040699	2041103	405
SEPPX05	IS_12c9bc1e_00029	99.37% IS1405_aa1	100% IS_12c9bc1e_01566	IS5 ssgr IS5	31796	30831	966
SEPPX05	IS_12c9bc1e_00145	98.81% IS1405_aa1	100% IS_12c9bc1e_01111	IS5 ssgr IS5	167617	168279	663
SEPPX05	IS_12c9bc1e_00147	99.35% IS1405_aa1	100% IS_12c9bc1e_01153	IS5 ssgr IS5	169908	170432	525
SEPPX05	IS_12c9bc1e_00677	98.81% IS1405_aa1	100% IS_12c9bc1e_01111	IS5 ssgr IS5	803587	804249	663
SEPPX05	IS_12c9bc1e_00681	99.35% IS1405_aa1	100% IS_12c9bc1e_01153	IS5 ssgr IS5	80784	808364	525
SEPPX05	IS_12c9bc1e_00778	99.35% IS1405_aa1	100% IS_12c9bc1e_01153	IS5 ssgr IS5	909456	908932	525
SEPPX05	IS_12c9bc1e_00780	98.81% IS1405_aa1	100% IS_12c9bc1e_01111	IS5 ssgr IS5	911747	911085	663
SEPPX05	IS_12c9bc1e_01111	98.81% IS1405_aa1	100% IS_12c9bc1e_00780	IS5 ssgr IS5	1307958	1308620	663
SEPPX05	IS_12c9bc1e_01113	99.35% IS1405_aa1	100% IS_12c9bc1e_01153	IS5 ssgr IS5	1310249	1310773	525
SEPPX05	IS_12c9bc1e_01153	99.35% IS1405_aa1	100% IS_12c9bc1e_01113	IS5 ssgr IS5	1362545	1362021	525
SEPPX05	IS_12c9bc1e_01157	98.14% IS1405_aa1	100% IS_12c9bc1e_01566	IS5 ssgr IS5	1366326	1365988	339
SEPPX05	IS_12c9bc1e_01566	99.37% IS1405_aa1	100% IS_12c9bc1e_00029	IS5 ssgr IS5	1879221	1880186	966
SEPPX05	IS_12c9bc1e_00400	55.50% ISDge13_aa1	No hit	IS6	464837	464103	735
SEPPX05	IS_12c9bc1e_00402	63.54% ISBcen19_aa1	No hit	IS66	469231	469524	294
SEPPX05	IS_12c9bc1e_00403	75.25% ISBcen19_aa2	No hit	IS66	469566	469856	291
SEPPX05	IS_12c9bc1e_00404	78.67% ISBcen19_aa3	No hit	IS66	469919	470998	1.08
SEPPX05	IS_12c9bc1e_00410	54.22% ISRtr5_aa1	No hit	IS66	473975	474424	450

SEPPX05	IS_12c9bc1e_00233	100% ISRso17_aa1	100% IS_12c9bc1e_00657	IS701	262542	263873	1.332
SEPPX05	IS_12c9bc1e_00396	100% ISRso17_aa1	100% IS_12c9bc1e_00657	IS701	460191	45886	1.332
SEPPX05	IS_12c9bc1e_00509	100% ISRso17_aa1	100% IS_12c9bc1e_00657	IS701	594577	595908	1.332
SEPPX05	IS_12c9bc1e_00657	100% ISRso17_aa1	100% IS_12c9bc1e_00509	IS701	78458	785911	1.332
SEPPX05	IS_12c9bc1e_01178	43.40% ISWz1_aa1	44.13% IS_12c9bc1e_01724	IS91	1387513	1385813	1.701
SEPPX05	IS_12c9bc1e_00977	99.75% ISRso15_aa1	100% IS_12c9bc1e_01709	ISL3	1146280	1147500	1.221
SEPPX05	IS_12c9bc1e_01573	99.75% ISRso15_aa1	100% IS_12c9bc1e_01709	ISL3	1888173	1886953	1.221
SEPPX05	IS_12c9bc1e_01709	99.75% ISRso15_aa1	100% IS_12c9bc1e_01573	ISL3	2045108	2046328	1.221
SEPPX05	IS_12c9bc1e_00507	51.46% ISMpo10_aa3	No hit	Tn3	59352	592147	1.374
Rs_10_244	CM002756_1_00483	82.67% IS1383_aa1	No hit	IS110 ssgr IS1111	615365	614364	1.002
Rs_10_244	CM002756_1_00033	87.03% ISRso10_aa2	87.03% CM002756_1_00558	IS3 ssgr IS2	40025	4042	396
Rs_10_244	CM002756_1_00558	93.06% ISRso10_aa3	87.03% CM002756_1_00033	IS3 ssgr IS2	705765	707	1.236
Rs_10_244	CM002756_1_01299	79.41% ISRso20_aa1	No hit	IS3 ssgr IS3	1647215	1647517	303
Rs_10_244	CM002756_1_01029	85.05% ISGau4_aa1	75.86% CM002756_1_01283	IS3 ssgr IS407	1307623	1307889	267
Rs_10_244	CM002756_1_01030	69.56% ISNoc1_aa2	No hit	IS3 ssgr IS407	1307922	1308137	216
Rs_10_244	CM002756_1_01035	76.16% ISGau4_aa2	No hit	IS3 ssgr IS407	1312936	1313523	588
Rs_10_244	CM002756_1_01283	100% ISRso12_aa1	75.86% CM002756_1_01029	IS3 ssgr IS407	1620069	1620335	267
Rs_10_244	CM002756_1_00484	100% IS1421_aa1	No hit	IS5 ssgr IS427	615548	615952	405
Rs_10_244	CM002756_1_00036	91.75% ISRso9_aa1	No hit	IS5 ssgr IS5	42286	41963	324
Rs_10_244	CM002756_1_00073	81.15% ISRso9_aa1	No hit	IS5 ssgr IS5	97625	9784	216
Rs_10_244	CM002756_1_01460	85.93% ISAau3_aa1	No hit	IS5 ssgr IS5	1839554	1839285	270
Rs_10_244	CM002756_1_01489	87.80% ISBmu2_aa1	No hit	IS5 ssgr IS5	1870352	1870101	252
Rs_10_244	CM002756_1_00045	100% ISRso17_aa1	No hit	IS701	56547	55216	1.332
Rs_10_244	CM002756_1_00874	43.40% ISWz1_aa1	44.13% CM002756_1_01593	IS91	1115042	1116742	1.701
Rs_10_244	CM002756_1_00041	100% ISRso15_aa1	No hit	ISL3	51432	50212	1.221
Rs_10_244	CM002756_1_00058	46.77% TnAs1_aa5	No hit	Tn3	80017	7943	588
Rs_09_16	CM002758_01250	77.61% ISRso20_aa1	No hit	IS3 ssgr IS3	1614868	1615086	219
Rs_09_16	CM002758_01489	92.30% ISRso16_aa2	No hit	IS3 ssgr IS407	1913067	1912675	393
Rs_09_16	CM002758_00047	67.17% IS1382_aa1	84.07% CM002758_01451	IS30	78757	77738	1.02
Rs_09_16	CM002758_00109	92.85% ISHar5_aa1	64.74% CM002758_00047	IS30	154189	153692	498
Rs_09_16	CM002758_01451	62.81% IST3091_aa1	84.07% CM002758_00047	IS30	1856587	1857201	615
Rs_09_16	CM002758_00046	99.77% ISRso13_aa1	No hit	IS4 ssgr IS4	74933	76267	1.335
Rs_09_16	CM002758_01034	91.30% ISRso1_aa1	No hit	IS5	1336774	1337148	375
Rs_09_16	CM002758_00048	100% IS1421_aa1	No hit	IS5 ssgr IS427	79709	79305	405
Rs_09_16	CM002758_00039	94.81% IS1021_aa1	71.47% CM002758_00040	IS5 ssgr IS5	51012	50026	987
Rs_09_16	CM002758_00040	99.06% IS1405_aa1	71.47% CM002758_00039	IS5 ssgr IS5	52353	53318	966
Rs_09_16	CM002758_00042	85.71% ISAau3_aa1	80.28% CM002758_00040	IS5 ssgr IS5	60413	6012	294
Rs_09_16	CM002758_00057	96.80% ISButh4_aa1	No hit	IS5 ssgr IS5	89166	902	1.035
Rs_09_16	CM002758_00038	100% ISRso17_aa1	No hit	IS701	44222	45553	1.332
Rs_09_16	CM002758_00804	43.40% ISWz1_aa1	43.44% CM002758_01533	IS91	1058254	1059954	1.701
Rs_09_16	CM002758_00821	62.5% ISAba32_aa1	No hit	ISNCY ssgr IS1202	1082202	1081987	216

SL3103	CP022791_00022	88.75% ISBcen4_aa1	100% CP022791_00342	IS110 ssgr IS1111	27496	26477	1.02
SL3103	CP022791_00342	88.75% ISBcen4_aa1	100% CP022791_00022	IS110 ssgr IS1111	442542	443561	1.02
SL3103	CP022791_00359	88.08% ISBcen4_aa1	100% CP022791_00342	IS110 ssgr IS1111	45886	459897	1.038
SL3103	CP022791_01643	61.67% ISMno14_aa1	100% CP022791_01651	IS110 ssgr IS1111	2081083	2082096	1.014
SL3103	CP022791_01651	61.67% ISMno14_aa1	100% CP022791_01643	IS110 ssgr IS1111	2088235	2087222	1.014
SL3103	CP022791_00405	83.75% ISBusp4_aa1	100% CP022791_01616	IS1182	520164	521609	1.446
SL3103	CP022791_00910	83.75% ISBusp4_aa1	100% CP022791_01616	IS1182	1170684	1172129	1.446
SL3103	CP022791_01275	82.73% ISBusp4_aa1	100% CP022791_01616	IS1182	1627892	1626636	1.257
SL3103	CP022791_01616	83.75% ISBusp4_aa1	100% CP022791_00910	IS1182	2042506	2041061	1.446
SL3103	CP022791_00463	91.08% ISPosp3_aa1	100% CP022791_01235	IS1595 ssgr ISSod11	625635	624649	987
SL3103	CP022791_00631	91.08% ISPosp3_aa1	100% CP022791_01235	IS1595 ssgr ISSod11	817562	818548	987
SL3103	CP022791_01235	91.08% ISPosp3_aa1	100% CP022791_00631	IS1595 ssgr ISSod11	1575675	1574689	987
SL3103	CP022791_00889	99.64% ISRso11_aa2	100% CP022791_01686	IS3 ssgr IS150	1150504	1149668	837
SL3103	CP022791_00890	100% ISRso11_aa1	100% CP022791_01687	IS3 ssgr IS150	1151034	1150501	534
SL3103	CP022791_01686	99.64% ISRso11_aa2	100% CP022791_00889	IS3 ssgr IS150	2116951	2116115	837
SL3103	CP022791_01687	100% ISRso11_aa1	100% CP022791_00890	IS3 ssgr IS150	2117481	2116948	534
SL3103	CP022791_00343	93.41% ISRso10_aa2	45.02% CP022791_01644	IS3 ssgr IS2	444054	444785	732
SL3103	CP022791_00407	95.74% ISRso10_aa1	No hit	IS3 ssgr IS2	522876	523256	381
SL3103	CP022791_01304	79.45% ISRso20_aa1	No hit	IS3 ssgr IS3	1675702	1675986	285
SL3103	CP022791_01644	93.25% ISRso16_aa2	44.58% CP022791_01686	IS3 ssgr IS407	2082443	2083054	612
SL3103	CP022791_01080	81.52% ISAisp2_aa1	100% CP022791_01510	IS3 ssgr IS51	1388429	1388707	279
SL3103	CP022791_01081	89.34% ISAisp2_aa2	100% CP022791_01509	IS3 ssgr IS51	1388704	1389579	876
SL3103	CP022791_01260	89.34% ISAisp2_aa2	98.28% CP022791_01081	IS3 ssgr IS51	1610244	1609369	876
SL3103	CP022791_01261	81.52% ISAisp2_aa1	97.82% CP022791_01510	IS3 ssgr IS51	1610519	1610241	279
SL3103	CP022791_01509	89.75% ISAisp2_aa2	100% CP022791_01081	IS3 ssgr IS51	1914140	1913289	852
SL3103	CP022791_01510	81.52% ISAisp2_aa1	100% CP022791_01080	IS3 ssgr IS51	1914415	1914137	279
SL3103	CP022791_00261	61.68% ISCro3_aa1	100% CP022791_01001	IS4	349903	351231	1.329
SL3103	CP022791_01001	61.68% ISCro3_aa1	100% CP022791_00261	IS4	1290460	1291788	1.329
SL3103	CP022791_00191	97.81% ISRso1_aa1	100% CP022791_01014	IS5	27647	277294	825
SL3103	CP022791_00487	97.81% ISRso1_aa1	100% CP022791_01014	IS5	6493	648476	825
SL3103	CP022791_00489	98.54% ISRso1_aa1	97.92% CP022791_00584	IS5	650834	652267	1.434
SL3103	CP022791_00582	97.81% ISRso1_aa1	100% CP022791_01014	IS5	761073	760249	825
SL3103	CP022791_00584	97.81% ISRso1_aa1	100% CP022791_01014	IS5	764703	763825	879
SL3103	CP022791_00759	97.81% ISRso1_aa1	100% CP022791_01014	IS5	993411	992587	825
SL3103	CP022791_00825	97.81% ISRso1_aa1	100% CP022791_01014	IS5	1089562	1090386	825
SL3103	CP022791_00864	97.81% ISRso1_aa1	100% CP022791_01014	IS5	1125897	1126721	825
SL3103	CP022791_01014	97.81% ISRso1_aa1	100% CP022791_00864	IS5	1307416	1306592	825
SL3103	CP022791_01016	98.66% ISRso1_aa1	100% CP022791_01014	IS5	1311713	1311036	678
SL3103	CP022791_01078	85.56% ISRso1_aa1	89.13% CP022791_01016	IS5	1387138	1387557	420
SL3103	CP022791_00406	100% IS1421_aa1	100% CP022791_01079	IS5 ssgr IS427	522035	522439	405
SL3103	CP022791_00585	99.25% IS1421_aa1	99.25% CP022791_01079	IS5 ssgr IS427	765561	765157	405
SL3103	CP022791_01013	100% IS1421_aa1	100% CP022791_01079	IS5 ssgr IS427	1305975	1305571	405

SL3103	CP022791_01079	100% IS1421_aa1	100% CP022791_01013	IS5 ssgr IS427	1387572	1387976	405
SL3103	CP022791_00025	94.81% IS1021_aa1	100% CP022791_01564	IS5 ssgr IS5	29694	3068	987
SL3103	CP022791_00051	99.06% IS1405_aa1	100% CP022791_01381	IS5 ssgr IS5	68362	67397	966
SL3103	CP022791_00124	99.06% IS1405_aa1	100% CP022791_01381	IS5 ssgr IS5	180855	18182	966
SL3103	CP022791_00194	99.06% IS1405_aa1	100% CP022791_01381	IS5 ssgr IS5	278868	277903	966
SL3103	CP022791_00346	99.06% IS1405_aa1	100% CP022791_01381	IS5 ssgr IS5	446224	447189	966
SL3103	CP022791_00350	99.06% IS1405_aa1	100% CP022791_01381	IS5 ssgr IS5	449619	448654	966
SL3103	CP022791_00388	94.81% IS1021_aa1	100% CP022791_01564	IS5 ssgr IS5	50372	502734	987
SL3103	CP022791_00389	99.06% IS1405_aa1	100% CP022791_01381	IS5 ssgr IS5	503877	504842	966
SL3103	CP022791_00391	94.81% IS1021_aa1	100% CP022791_01564	IS5 ssgr IS5	5051	506086	987
SL3103	CP022791_00587	99.06% IS1405_aa1	100% CP022791_01381	IS5 ssgr IS5	766718	765753	966
SL3103	CP022791_00619	99.06% IS1405_aa1	100% CP022791_01381	IS5 ssgr IS5	806971	807936	966
SL3103	CP022791_00930	94.81% IS1021_aa1	100% CP022791_01564	IS5 ssgr IS5	1195128	1196114	987
SL3103	CP022791_00947	99.06% IS1405_aa1	100% CP022791_01381	IS5 ssgr IS5	1220167	1219202	966
SL3103	CP022791_01071	94.81% IS1021_aa1	100% CP022791_01564	IS5 ssgr IS5	1381838	1380852	987
SL3103	CP022791_01194	99.06% IS1405_aa1	100% CP022791_01381	IS5 ssgr IS5	1532738	1533703	966
SL3103	CP022791_01231	99.06% IS1405_aa1	100% CP022791_01381	IS5 ssgr IS5	1571454	1572419	966
SL3103	CP022791_01350	94.81% IS1021_aa1	100% CP022791_01564	IS5 ssgr IS5	1723173	1724159	987
SL3103	CP022791_01377	99.06% IS1405_aa1	100% CP022791_01381	IS5 ssgr IS5	1760075	1761040	966
SL3103	CP022791_01381	99.06% IS1405_aa1	100% CP022791_01377	IS5 ssgr IS5	1763470	1762505	966
SL3103	CP022791_01386	94.81% IS1021_aa1	100% CP022791_01564	IS5 ssgr IS5	1767588	1768574	987
SL3103	CP022791_01513	94.81% IS1021_aa1	100% CP022791_01564	IS5 ssgr IS5	1920401	1919415	987
SL3103	CP022791_01564	94.81% IS1021_aa1	100% CP022791_01513	IS5 ssgr IS5	1981070	1982056	987
SL3103	CP022791_00013	100% IS1420_aa1	100% CP022791_01598	IS5 ssgr IS903	16135	15179	957
SL3103	CP022791_00033	100% IS1420_aa1	100% CP022791_01598	IS5 ssgr IS903	36101	37057	957
SL3103	CP022791_00046	100% IS1420_aa1	100% CP022791_01598	IS5 ssgr IS903	6546	64504	957
SL3103	CP022791_00068	100% IS1420_aa1	100% CP022791_01598	IS5 ssgr IS903	100475	99519	957
SL3103	CP022791_00169	100% IS1420_aa1	100% CP022791_01598	IS5 ssgr IS903	247046	248002	957
SL3103	CP022791_00325	100% IS1420_aa1	100% CP022791_01598	IS5 ssgr IS903	422825	423781	957
SL3103	CP022791_00352	100% IS1420_aa1	100% CP022791_01598	IS5 ssgr IS903	452968	453924	957
SL3103	CP022791_00387	100% IS1420_aa1	100% CP022791_01598	IS5 ssgr IS903	501118	502074	957
SL3103	CP022791_00415	100% IS1420_aa1	100% CP022791_01598	IS5 ssgr IS903	529971	529015	957
SL3103	CP022791_00426	100% IS1420_aa1	100% CP022791_01598	IS5 ssgr IS903	539866	53891	957
SL3103	CP022791_00452	100% IS1420_aa1	100% CP022791_01598	IS5 ssgr IS903	568494	56945	957
SL3103	CP022791_00560	100% IS1420_aa1	100% CP022791_01598	IS5 ssgr IS903	729128	728172	957
SL3103	CP022791_00583	99.68% IS1420_aa1	99.68% CP022791_01598	IS5 ssgr IS903	762198	761242	957
SL3103	CP022791_00780	100% IS1420_aa1	100% CP022791_01598	IS5 ssgr IS903	1042441	1043397	957
SL3103	CP022791_00799	100% IS1420_aa1	100% CP022791_01598	IS5 ssgr IS903	1062088	1061132	957
SL3103	CP022791_00830	95.25% IS1420_aa1	95.25% CP022791_00583	IS5 ssgr IS903	1092901	1093968	1.068
SL3103	CP022791_00945	100% IS1420_aa1	100% CP022791_01598	IS5 ssgr IS903	1218777	1217821	957
SL3103	CP022791_01192	100% IS1420_aa1	100% CP022791_01598	IS5 ssgr IS903	1530757	1531713	957
SL3103	CP022791_01276	100% IS1420_aa1	100% CP022791_01598	IS5 ssgr IS903	1628155	1629111	957
SL3103	CP022791_01280	100% IS1420_aa1	100% CP022791_01598	IS5 ssgr IS903	1633886	1632930	957

SL3103	CP022791_01351	100% IS1420_aa1	100% CP022791_01598	IS5 ssgr IS903	1725295	1724339	957
SL3103	CP022791_01371	100% IS1420_aa1	100% CP022791_01598	IS5 ssgr IS903	1748643	1749599	957
SL3103	CP022791_01435	100% IS1420_aa1	100% CP022791_01598	IS5 ssgr IS903	1820508	1821464	957
SL3103	CP022791_01437	100% IS1420_aa1	100% CP022791_01598	IS5 ssgr IS903	1823597	1822641	957
SL3103	CP022791_01497	99.68% IS1420_aa1	99.68% CP022791_01598	IS5 ssgr IS903	1899300	1898344	957
SL3103	CP022791_01547	100% IS1420_aa1	100% CP022791_01598	IS5 ssgr IS903	1962249	1961413	837
SL3103	CP022791_01548	100% IS1420_aa1	100% CP022791_01598	IS5 ssgr IS903	1962370	1963326	957
SL3103	CP022791_01562	100% IS1420_aa1	100% CP022791_01598	IS5 ssgr IS903	1979239	1980195	957
SL3103	CP022791_01565	100% IS1420_aa1	100% CP022791_01598	IS5 ssgr IS903	1983437	1982583	855
SL3103	CP022791_01596	100% IS1420_aa1	100% CP022791_01598	IS5 ssgr IS903	2016384	2015428	957
SL3103	CP022791_01598	100% IS1420_aa1	100% CP022791_01596	IS5 ssgr IS903	2016794	2017750	957
SL3103	CP022791_00296	55.55% ISRm2_aa1	100% CP022791_01505	IS66	392794	393258	465
SL3103	CP022791_00297	73.27% ISRm2_aa2	100% CP022791_01506	IS66	393255	393608	354
SL3103	CP022791_00298	60.75% ISPpu13_aa2	100% CP022791_01507	IS66	393641	39523	1.59
SL3103	CP022791_00356	56.95% ISRm2_aa1	100% CP022791_01647	IS66	456466	456948	483
SL3103	CP022791_00357	73.27% ISRm2_aa2	100% CP022791_01646	IS66	456945	457298	354
SL3103	CP022791_00358	59.79% ISAeh1_aa2	100% CP022791_01645	IS66	457331	458887	1.557
SL3103	CP022791_00409	56.95% ISRm2_aa1	100% CP022791_01647	IS66	523379	523861	483
SL3103	CP022791_00410	73.27% ISRm2_aa2	100% CP022791_01646	IS66	523858	524211	354
SL3103	CP022791_00411	59.79% ISAeh1_aa2	100% CP022791_01645	IS66	524244	5258	1.557
SL3103	CP022791_00467	60.75% ISPpu13_aa2	100% CP022791_01507	IS66	62938	627791	1.59
SL3103	CP022791_00468	73.27% ISRm2_aa2	100% CP022791_01506	IS66	629766	629413	354
SL3103	CP022791_00469	55.55% ISRm2_aa1	100% CP022791_01505	IS66	630227	629763	465
SL3103	CP022791_01092	56.95% ISRm2_aa1	100% CP022791_01647	IS66	1398365	1398847	483
SL3103	CP022791_01093	73.27% ISRm2_aa2	100% CP022791_01646	IS66	1398844	1399197	354
SL3103	CP022791_01094	59.79% ISAeh1_aa2	100% CP022791_01645	IS66	1399230	1400786	1.557
SL3103	CP022791_01383	59.79% ISAeh1_aa2	100% CP022791_01645	IS66	1766447	1764891	1.557
SL3103	CP022791_01384	73.27% ISRm2_aa2	100% CP022791_01646	IS66	1766833	1766480	354
SL3103	CP022791_01385	56.95% ISRm2_aa1	100% CP022791_01647	IS66	1767312	1766830	483
SL3103	CP022791_01505	55.55% ISRm2_aa1	100% CP022791_00469	IS66	1909576	1910040	465
SL3103	CP022791_01506	73.27% ISRm2_aa2	100% CP022791_00468	IS66	1910037	1910390	354
SL3103	CP022791_01507	60.75% ISPpu13_aa2	100% CP022791_00467	IS66	1910423	1912012	1.59
SL3103	CP022791_01645	59.79% ISAeh1_aa2	100% CP022791_01383	IS66	2084531	2082975	1.557
SL3103	CP022791_01646	73.27% ISRm2_aa2	100% CP022791_01384	IS66	2084917	2084564	354
SL3103	CP022791_01647	56.95% ISRm2_aa1	100% CP022791_01385	IS66	2085396	2084914	483
SL3103	CP022791_00726	42.78% ISWz1_aa1	100% CP022791_00806	IS91	942785	941112	1.674
SL3103	CP022791_00806	42.78% ISWz1_aa1	100% CP022791_00726	IS91	1074163	1075836	1.674
SL3103	CP022791_00732	62.5% ISKpn21_aa1	No hit	ISNCY ssgr IS1202	949029	94943	402
SL3103	CP022791_00730	75.15% ISPa42_aa1	100% CP022791_00802	Tn3	947385	94444	2.946
SL3103	CP022791_00802	75.15% ISPa42_aa1	100% CP022791_00730	Tn3	1069563	1072508	2.946
T117	CP022756_00427	88.75% ISBcen4_aa1	100% CP022756_00538	IS110 ssgr IS1111	548001	54902	1.02
T117	CP022756_00538	88.75% ISBcen4_aa1	100% CP022756_00427	IS110 ssgr IS1111	686527	687546	1.02

T117	CP022756_01562	61.67% ISMno14_aa1	100% CP022756_01567	IS110 ssgr IS1111	2011053	2012066	1.014
T117	CP022756_01567	61.67% ISMno14_aa1	100% CP022756_01562	IS110 ssgr IS1111	2015645	2014632	1.014
T117	CP022756_01296	83.75% ISBusp4_aa1	No hit	IS1182	1658288	1656843	1.446
T117	CP022756_00505	50.44% ISLsp2_aa1	No hit	IS1595 ssgr ISPna2	652441	653718	1.278
T117	CP022756_00537	95.74% ISRso10_aa1	No hit	IS3 ssgr IS2	686172	686552	381
T117	CP022756_00539	93.41% ISRso10_aa2	44.70% CP022756_01566	IS3 ssgr IS2	68804	688771	732
T117	CP022756_01322	78.68% ISRso20_aa1	No hit	IS3 ssgr IS3	1705883	1706083	201
T117	CP022756_01566	93.82% ISRso16_aa2	44.70% CP022756_00539	IS3 ssgr IS407	2014285	2013746	540
T117	CP022756_00478	81.52% ISAisp2_aa1	100% CP022756_01282	IS3 ssgr IS51	61591	616188	279
T117	CP022756_00479	89.34% ISAisp2_aa2	100% CP022756_01281	IS3 ssgr IS51	616185	61706	876
T117	CP022756_01110	81.52% ISAisp2_aa1	97.82% CP022756_01282	IS3 ssgr IS51	1418517	1418795	279
T117	CP022756_01111	89.34% ISAisp2_aa2	98.28% CP022756_01281	IS3 ssgr IS51	1418792	1419667	876
T117	CP022756_01281	89.34% ISAisp2_aa2	100% CP022756_00479	IS3 ssgr IS51	1640451	1639576	876
T117	CP022756_01282	81.52% ISAisp2_aa1	100% CP022756_00478	IS3 ssgr IS51	1640726	1640448	279
T117	CP022756_00354	61.44% ISCro3_aa1	No hit	IS4	461516	460188	1.329
T117	CP022756_00265	78.03% ISAzo5_aa1	100% CP022756_00721	IS4 ssgr IS50	358264	359589	1.326
T117	CP022756_00645	78.03% ISAzo5_aa1	100% CP022756_00721	IS4 ssgr IS50	851525	8502	1.326
T117	CP022756_00701	78.03% ISAzo5_aa1	100% CP022756_00721	IS4 ssgr IS50	911108	909783	1.326
T117	CP022756_00721	78.03% ISAzo5_aa1	100% CP022756_00701	IS4 ssgr IS50	939033	937708	1.326
T117	CP022756_00506	82.75% ISBcen26_aa1	No hit	IS481	654213	653863	351
T117	CP022756_00045	97.81% ISRso1_aa1	100% CP022756_01301	IS5	49416	5042	1.005
T117	CP022756_00080	97.81% ISRso1_aa1	100% CP022756_00045	IS5	94321	93497	825
T117	CP022756_00624	98.54% ISRso1_aa1	97.26% CP022756_00045	IS5	822955	824388	1.434
T117	CP022756_00720	97.81% ISRso1_aa1	100% CP022756_00045	IS5	936943	936119	825
T117	CP022756_01097	98.54% ISRso1_aa1	98.54% CP022756_00045	IS5	1409185	1408361	825
T117	CP022756_01109	85.56% ISRso1_aa1	86.59% CP022756_00045	IS5	1418093	1418485	393
T117	CP022756_01301	97.81% ISRso1_aa1	100% CP022756_00045	IS5	1665315	1664491	825
T117	CP022756_00723	99.25% IS1421_aa1	No hit	IS5 ssgr IS427	940887	940483	405
T117	CP022756_00042	99.06% IS1405_aa1	100% CP022756_01219	IS5 ssgr IS5	47019	47984	966
T117	CP022756_00044	99.06% IS1405_aa1	100% CP022756_01219	IS5 ssgr IS5	48454	49419	966
T117	CP022756_00084	99.37% IS1405_aa1	100% CP022756_01578	IS5 ssgr IS5	102229	101264	966
T117	CP022756_00165	94.51% IS1021_aa1	71.80% CP022756_01578	IS5 ssgr IS5	213413	212427	987
T117	CP022756_00456	99.06% IS1405_aa1	100% CP022756_01219	IS5 ssgr IS5	590686	591651	966
T117	CP022756_00543	99.06% IS1405_aa1	100% CP022756_01219	IS5 ssgr IS5	691406	690441	966
T117	CP022756_00677	99.06% IS1405_aa1	100% CP022756_01219	IS5 ssgr IS5	884174	885139	966
T117	CP022756_00879	99.06% IS1405_aa1	100% CP022756_01219	IS5 ssgr IS5	1139945	1138980	966
T117	CP022756_00885	99.06% IS1405_aa1	100% CP022756_01219	IS5 ssgr IS5	1145804	1146769	966
T117	CP022756_01044	99.06% IS1405_aa1	100% CP022756_01219	IS5 ssgr IS5	1337953	1338918	966
T117	CP022756_01122	99.37% IS1405_aa1	100% CP022756_01578	IS5 ssgr IS5	1429028	1429993	966
T117	CP022756_01219	99.06% IS1405_aa1	100% CP022756_01044	IS5 ssgr IS5	1565211	1566176	966
T117	CP022756_01445	99.06% IS1405_aa1	99.68% CP022756_01578	IS5 ssgr IS5	1854587	1853622	966
T117	CP022756_01578	99.37% IS1405_aa1	100% CP022756_01122	IS5 ssgr IS5	2026786	2027751	966
T117	CP022756_00508	79.66% ISCARN39_aa3	No hit	IS630	656582	656046	537

T117	CP022756_00867	43.40% ISWz1_aa1	44.13% CP022756_01614	IS91	1126059	1127759	1.701
T117	CP022756_00880	72.27% ISKpn21_aa1	70% CP022756_00881	ISNCY ssgr IS1202	1140664	1142139	1.476
T117	CP022756_00881	62.5% ISAba32_aa1	70% CP022756_00880	ISNCY ssgr IS1202	1142282	1142497	216
T117	CP022756_00468	50.25% ISMpo10_aa3	No hit	Tn3	604629	603256	1.374
SL2330	CP022795_00152	88.75% ISBcen4_aa1	100% CP022795_01138	IS110 ssgr IS1111	195032	196051	1.02
SL2330	CP022795_00419	88.75% ISBcen4_aa1	100% CP022795_01138	IS110 ssgr IS1111	540999	542018	1.02
SL2330	CP022795_00463	88.75% ISBcen4_aa1	100% CP022795_01138	IS110 ssgr IS1111	599848	600867	1.02
SL2330	CP022795_01138	88.75% ISBcen4_aa1	100% CP022795_00463	IS110 ssgr IS1111	1483512	1484531	1.02
SL2330	CP022795_01192	89.93% ISPosp3_aa1	No hit	IS1595 ssgr ISSod11	1540217	1539225	993
SL2330	CP022795_00462	95.74% ISRso10_aa1	No hit	IS3 ssgr IS2	599493	599873	381
SL2330	CP022795_00464	93.00% ISRso10_aa2	No hit	IS3 ssgr IS2	60136	602079	720
SL2330	CP022795_01269	78.68% ISRso20_aa1	No hit	IS3 ssgr IS3	1654069	1654269	201
SL2330	CP022795_00469	81.52% ISAisp2_aa1	97.82% CP022795_01217	IS3 ssgr IS51	604877	605155	279
SL2330	CP022795_00470	89.34% ISAisp2_aa2	98.28% CP022795_01216	IS3 ssgr IS51	605152	606027	876
SL2330	CP022795_01216	89.34% ISAisp2_aa2	98.28% CP022795_00470	IS3 ssgr IS51	1574715	1573840	876
SL2330	CP022795_01217	81.52% ISAisp2_aa1	97.82% CP022795_00469	IS3 ssgr IS51	1574990	1574712	279
SL2330	CP022795_00080	98.90% ISRso1_aa1	99.27% CP022795_00564	IS5	96427	95603	825
SL2330	CP022795_00564	98.54% ISRso1_aa1	99.27% CP022795_00080	IS5	750101	751534	1.434
SL2330	CP022795_01040	96.96% ISRso1_aa1	96.96% CP022795_00564	IS5	1347359	1347559	201
SL2330	CP022795_00525	100% IS1421_aa1	84.74% CP022795_00527	IS5 ssgr IS427	667316	666912	405
SL2330	CP022795_00527	87.15% IS1421_aa1	87.15% CP022795_00525	IS5 ssgr IS427	668769	668398	372
SL2330	CP022795_00076	99.37% IS1405_aa1	100% CP022795_01428	IS5 ssgr IS5	8887	87905	966
SL2330	CP022795_00405	99.37% IS1405_aa1	100% CP022795_01428	IS5 ssgr IS5	522898	521933	966
SL2330	CP022795_00445	99.37% IS1405_aa1	100% CP022795_01428	IS5 ssgr IS5	583403	582438	966
SL2330	CP022795_00528	90.27% IS1021_aa1	79.24% CP022795_01428	IS5 ssgr IS5	669474	669085	390
SL2330	CP022795_00529	90.65% IS1021_aa1	79.43% CP022795_01428	IS5 ssgr IS5	669843	669508	336
SL2330	CP022795_00639	99.37% IS1405_aa1	100% CP022795_01428	IS5 ssgr IS5	835898	836863	966
SL2330	CP022795_00823	99.37% IS1405_aa1	100% CP022795_01428	IS5 ssgr IS5	1071737	1072702	966
SL2330	CP022795_00976	99.37% IS1405_aa1	100% CP022795_01428	IS5 ssgr IS5	1266073	1265108	966
SL2330	CP022795_01000	99.37% IS1405_aa1	100% CP022795_01428	IS5 ssgr IS5	1302227	1303192	966
SL2330	CP022795_01126	99.37% IS1405_aa1	100% CP022795_01428	IS5 ssgr IS5	1466129	1467094	966
SL2330	CP022795_01428	99.37% IS1405_aa1	100% CP022795_01126	IS5 ssgr IS5	1847463	1848428	966
SL2330	CP022795_00812	43.40% ISWz1_aa1	40.92% CP022795_01558	IS91	1058670	1060370	1.701
SL2330	CP022795_00829	62.5% ISAba32_aa1	70% CP022795_00830	ISNCY ssgr IS1202	1079069	1078854	216
SL2330	CP022795_00830	72.48% ISKpn21_aa1	70% CP022795_00829	ISNCY ssgr IS1202	1080686	1079211	1.476
SL3755	CP022783_00151	88.75% ISBcen4_aa1	100% CP022783_01152	IS110 ssgr IS1111	195033	196052	1.02
SL3755	CP022783_00422	88.75% ISBcen4_aa1	100% CP022783_01152	IS110 ssgr IS1111	541904	542923	1.02
SL3755	CP022783_00465	88.75% ISBcen4_aa1	100% CP022783_01152	IS110 ssgr IS1111	599586	600605	1.02
SL3755	CP022783_01152	88.75% ISBcen4_aa1	100% CP022783_00465	IS110 ssgr IS1111	1486781	1487800	1.02
SL3755	CP022783_01208	89.93% ISPosp3_aa1	No hit	IS1595 ssgr ISSod11	1545401	1544409	993
SL3755	CP022783_00464	95.74% ISRso10_aa1	No hit	IS3 ssgr IS2	599231	599611	381

SL3755	CP022783_00466	93.00% ISRso10_aa2	No hit	IS3 ssgr IS2	601098	601817	720
SL3755	CP022783_01288	79.45% ISRso20_aa1	No hit	IS3 ssgr IS3	1659166	1659450	285
SL3755	CP022783_00471	81.52% ISAisp2_aa1	97.82% CP022783_01235	IS3 ssgr IS51	604615	604893	279
SL3755	CP022783_00472	89.34% ISAisp2_aa2	98.28% CP022783_01234	IS3 ssgr IS51	60489	605765	876
SL3755	CP022783_01234	89.34% ISAisp2_aa2	98.28% CP022783_00472	IS3 ssgr IS51	1579897	1579022	876
SL3755	CP022783_01235	81.52% ISAisp2_aa1	97.82% CP022783_00471	IS3 ssgr IS51	1580172	1579894	279
SL3755	CP022783_00079	98.90% ISRso1_aa1	99.27% CP022783_00570	IS5	96422	95598	825
SL3755	CP022783_00570	98.54% ISRso1_aa1	99.27% CP022783_00079	IS5	751502	752935	1.434
SL3755	CP022783_01054	96.96% ISRso1_aa1	96.96% CP022783_00570	IS5	1350632	1350832	201
SL3755	CP022783_00161	100% IS1421_aa1	100% CP022783_00530	IS5 ssgr IS427	207761	208165	405
SL3755	CP022783_00530	100% IS1421_aa1	100% CP022783_00161	IS5 ssgr IS427	669588	669184	405
SL3755	CP022783_00075	99.37% IS1405_aa1	100% CP022783_01140	IS5 ssgr IS5	88865	879	966
SL3755	CP022783_00408	99.37% IS1405_aa1	100% CP022783_01140	IS5 ssgr IS5	523804	522839	966
SL3755	CP022783_00531	90.27% IS1021_aa1	79.24% CP022783_01140	IS5 ssgr IS5	670879	67049	390
SL3755	CP022783_00532	90.65% IS1021_aa1	79.43% CP022783_01140	IS5 ssgr IS5	671248	670913	336
SL3755	CP022783_00645	99.37% IS1405_aa1	100% CP022783_01140	IS5 ssgr IS5	837296	838261	966
SL3755	CP022783_00833	99.37% IS1405_aa1	100% CP022783_01140	IS5 ssgr IS5	1075035	1076000	966
SL3755	CP022783_00989	99.37% IS1405_aa1	100% CP022783_01140	IS5 ssgr IS5	1269336	1268371	966
SL3755	CP022783_01013	99.37% IS1405_aa1	100% CP022783_01140	IS5 ssgr IS5	1305501	1306466	966
SL3755	CP022783_01140	99.37% IS1405_aa1	100% CP022783_01013	IS5 ssgr IS5	1469399	1470364	966
SL3755	CP022783_00822	43.40% ISWz1_aa1	40.92% CP022783_01567	IS91	1061968	1063668	1.701
SL3755	CP022783_00839	62.5% ISAba32_aa1	70% CP022783_00840	ISNCY ssgr IS1202	1082366	1082151	216
SL3755	CP022783_00840	72.48% ISKpn21_aa1	70% CP022783_00839	ISNCY ssgr IS1202	1083983	1082508	1.476
T25	CP023015_00173	88.75% ISBcen4_aa1	100% CP023015_01316	IS110 ssgr IS1111	196171	19719	1.02
T25	CP023015_01237	88.75% ISBcen4_aa1	100% CP023015_01316	IS110 ssgr IS1111	1389893	1388874	1.02
T25	CP023015_01288	88.75% ISBcen4_aa1	100% CP023015_01316	IS110 ssgr IS1111	1447564	1446545	1.02
T25	CP023015_01316	88.75% ISBcen4_aa1	100% CP023015_01288	IS110 ssgr IS1111	1483038	1484057	1.02
T25	CP023015_01375	89.93% ISPosp3_aa1	No hit	IS1595 ssgr ISSod11	1539705	1538713	993
T25	CP023015_01236	93.00% ISRso10_aa2	No hit	IS3 ssgr IS2	1388381	1387662	720
T25	CP023015_01238	95.74% ISRso10_aa1	No hit	IS3 ssgr IS2	1390248	1389868	381
T25	CP023015_01464	79.45% ISRso20_aa1	No hit	IS3 ssgr IS3	1653457	1653741	285
T25	CP023015_01719	92.85% ISRso16_aa2	47.42% CP023015_01236	IS3 ssgr IS407	1954906	1955244	339
T25	CP023015_01722	92.06% ISRso16_aa2	No hit	IS3 ssgr IS407	1956712	1956200	513
T25	CP023015_01230	89.34% ISAisp2_aa2	98.28% CP023015_01403	IS3 ssgr IS51	1384589	1383714	876
T25	CP023015_01231	81.52% ISAisp2_aa1	97.82% CP023015_01404	IS3 ssgr IS51	1384864	1384586	279
T25	CP023015_01403	89.34% ISAisp2_aa2	98.28% CP023015_01230	IS3 ssgr IS51	1574199	1573324	876
T25	CP023015_01404	81.52% ISAisp2_aa1	97.82% CP023015_01231	IS3 ssgr IS51	1574474	1574196	279
T25	CP023015_00093	98.90% ISRso1_aa1	99.27% CP023015_01116	IS5	97576	96752	825
T25	CP023015_00556	96.96% ISRso1_aa1	96.96% CP023015_01116	IS5	642263	642063	201
T25	CP023015_01116	98.54% ISRso1_aa1	99.27% CP023015_00093	IS5	1238001	1236568	1.434
T25	CP023015_01170	99.11% IS1421_aa1	No hit	IS5 ssgr IS427	1319887	1320270	384
T25	CP023015_00086	99.37% IS1405_aa1	100% CP023015_01303	IS5 ssgr IS5	88843	87878	966

T25	CP023015_00090	100% IS1405_aa1	100% CP023015_01303	IS5 ssgr IS5	94736	94326	411
T25	CP023015_00091	98.91% IS1405_aa1	100% CP023015_01303	IS5 ssgr IS5	9529	94694	597
T25	CP023015_00459	99.37% IS1405_aa1	100% CP023015_01303	IS5 ssgr IS5	523527	522562	966
T25	CP023015_00601	99.37% IS1405_aa1	100% CP023015_01303	IS5 ssgr IS5	687372	686407	966
T25	CP023015_00627	99.37% IS1405_aa1	100% CP023015_01303	IS5 ssgr IS5	723467	724432	966
T25	CP023015_00805	99.37% IS1405_aa1	100% CP023015_01303	IS5 ssgr IS5	917682	916717	966
T25	CP023015_01168	90.65% IS1021_aa1	79.43% CP023015_01303	IS5 ssgr IS5	1318228	1318563	336
T25	CP023015_01169	90.27% IS1021_aa1	79.24% CP023015_01303	IS5 ssgr IS5	1318597	1318986	390
T25	CP023015_01303	99.37% IS1405_aa1	100% CP023015_00805	IS5 ssgr IS5	1465659	1466624	966
T25	CP023015_00817	43.40% ISWz1_aa1	40.92% CP023015_01768	IS91	930747	929047	1.701
T25	CP023015_00796	66.91% ISKpn21_aa1	70% CP023015_00798	ISNCY ssgr IS1202	908737	909267	531
T25	CP023015_00797	74.02% ISKpn21_aa1	No hit	ISNCY ssgr IS1202	909267	910211	945
T25	CP023015_00798	62.5% ISAba32_aa1	70% CP023015_00796	ISNCY ssgr IS1202	910353	910568	216
T78	CP022767_00106	85.13% ISAzo9_aa2	No hit	IS630	102609	101482	1.128
T78	CP022767_00102	61.53% ISPpu13_aa2	100% CP022767_00134	IS66	99422	97866	1.557
T78	CP022767_00103	73.27% ISRm2_aa2	100% CP022767_00135	IS66	99808	99455	354
T78	CP022767_00104	56.95% ISRm2_aa1	100% CP022767_00136	IS66	100287	99805	483
T78	CP022767_00134	61.53% ISPpu13_aa2	100% CP022767_00102	IS66	123813	122257	1.557
T78	CP022767_00135	73.27% ISRm2_aa2	100% CP022767_00103	IS66	124199	123846	354
T78	CP022767_00136	58.75% ISRtr5_aa1	100% CP022767_00104	IS66	124677	124396	282
SL3730	CP022785_00446	88.75% ISBcen4_aa1	92.70% CP022785_00502	IS110 ssgr IS1111	556177	557196	1.02
SL3730	CP022785_00502	84.67% ISBcen4_aa1	92.70% CP022785_00446	IS110 ssgr IS1111	620821	621237	417
SL3730	CP022785_01535	61.67% ISMno14_aa1	100% CP022785_01540	IS110 ssgr IS1111	1920034	1921047	1.014
SL3730	CP022785_01540	61.67% ISMno14_aa1	100% CP022785_01535	IS110 ssgr IS1111	1924626	1923613	1.014
SL3730	CP022785_01265	83.75% ISBusp4_aa1	No hit	IS1182	1566139	1564694	1.446
SL3730	CP022785_00501	95.74% ISRso10_aa1	No hit	IS3 ssgr IS2	620466	620846	381
SL3730	CP022785_01288	78.68% ISRso20_aa1	No hit	IS3 ssgr IS3	1612858	1613058	201
SL3730	CP022785_01539	93.82% ISRso16_aa2	44.58% CP022785_01078	IS3 ssgr IS407	1923266	1922727	540
SL3730	CP022785_01077	81.52% ISAisp2_aa1	97.82% CP022785_01251	IS3 ssgr IS51	1326396	1326674	279
SL3730	CP022785_01078	89.34% ISAisp2_aa2	98.28% CP022785_01250	IS3 ssgr IS51	1326671	1327546	876
SL3730	CP022785_01250	89.34% ISAisp2_aa2	98.28% CP022785_01078	IS3 ssgr IS51	1548302	1547427	876
SL3730	CP022785_01251	81.52% ISAisp2_aa1	97.82% CP022785_01077	IS3 ssgr IS51	1548577	1548299	279
SL3730	CP022785_00002	61.68% ISCro3_aa1	99.77% CP022785_00373	IS4	3517	2189	1.329
SL3730	CP022785_00373	61.44% ISCro3_aa1	99.77% CP022785_00002	IS4	469696	468368	1.329
SL3730	CP022785_00580	98.54% ISRso1_aa1	85.67% CP022785_00605	IS5	732438	733871	1.434
SL3730	CP022785_00605	97.81% ISRso1_aa1	85.67% CP022785_00580	IS5	765485	76293	2.556
SL3730	CP022785_00677	97.81% ISRso1_aa1	100% CP022785_00605	IS5	84434	843516	825
SL3730	CP022785_01030	97.81% ISRso1_aa1	100% CP022785_00605	IS5	1277990	1278814	825
SL3730	CP022785_01064	98.54% ISRso1_aa1	98.54% CP022785_00605	IS5	1317065	1316241	825
SL3730	CP022785_01076	85.56% ISRso1_aa1	86.59% CP022785_00605	IS5	1325972	1326364	393
SL3730	CP022785_00085	100% IS1421_aa1	100% CP022785_01435	IS5 ssgr IS427	102835	102452	384

SL3730	CP022785_00234	99.25% IS1421_aa1	100% CP022785_01435	IS5 ssgr IS427	300526	30093	405
SL3730	CP022785_00497	99.25% IS1421_aa1	100% CP022785_01435	IS5 ssgr IS427	617987	617583	405
SL3730	CP022785_00679	99.25% IS1421_aa1	98.50% CP022785_01435	IS5 ssgr IS427	8468	846396	405
SL3730	CP022785_01435	99.25% IS1421_aa1	100% CP022785_00497	IS5 ssgr IS427	1781324	1780920	405
SL3730	CP022785_00031	99.06% IS1405_aa1	100% CP022785_01379	IS5 ssgr IS5	35894	34929	966
SL3730	CP022785_00043	94.51% IS1021_aa1	100% CP022785_00542	IS5 ssgr IS5	48312	49298	987
SL3730	CP022785_00046	99.06% IS1405_aa1	100% CP022785_01379	IS5 ssgr IS5	51105	5207	966
SL3730	CP022785_00081	94.51% IS1021_aa1	100% CP022785_00542	IS5 ssgr IS5	94004	93018	987
SL3730	CP022785_00087	99.37% IS1405_aa1	100% CP022785_01416	IS5 ssgr IS5	105071	104106	966
SL3730	CP022785_00168	94.51% IS1021_aa1	100% CP022785_00542	IS5 ssgr IS5	214737	213751	987
SL3730	CP022785_00170	94.51% IS1021_aa1	100% CP022785_00542	IS5 ssgr IS5	217674	216688	987
SL3730	CP022785_00233	94.51% IS1021_aa1	100% CP022785_00542	IS5 ssgr IS5	299356	300342	987
SL3730	CP022785_00262	99.06% IS1405_aa1	100% CP022785_01379	IS5 ssgr IS5	345438	344473	966
SL3730	CP022785_00304	99.06% IS1405_aa1	100% CP022785_01379	IS5 ssgr IS5	396365	39733	966
SL3730	CP022785_00477	99.06% IS1405_aa1	100% CP022785_01379	IS5 ssgr IS5	60001	600975	966
SL3730	CP022785_00485	99.06% IS1405_aa1	100% CP022785_01379	IS5 ssgr IS5	604362	605327	966
SL3730	CP022785_00504	99.06% IS1405_aa1	100% CP022785_01379	IS5 ssgr IS5	622286	621321	966
SL3730	CP022785_00542	94.51% IS1021_aa1	100% CP022785_00233	IS5 ssgr IS5	660975	659989	987
SL3730	CP022785_00634	99.06% IS1405_aa1	100% CP022785_01379	IS5 ssgr IS5	793058	794023	966
SL3730	CP022785_00840	99.06% IS1405_aa1	100% CP022785_01379	IS5 ssgr IS5	1045838	1044873	966
SL3730	CP022785_00846	99.06% IS1405_aa1	100% CP022785_01379	IS5 ssgr IS5	1051696	1052661	966
SL3730	CP022785_01010	99.06% IS1405_aa1	100% CP022785_01379	IS5 ssgr IS5	1243847	1244812	966
SL3730	CP022785_01089	99.37% IS1405_aa1	100% CP022785_01416	IS5 ssgr IS5	1336907	1337872	966
SL3730	CP022785_01187	99.06% IS1405_aa1	100% CP022785_01379	IS5 ssgr IS5	1473066	1474031	966
SL3730	CP022785_01379	99.06% IS1405_aa1	100% CP022785_01187	IS5 ssgr IS5	1724013	1724978	966
SL3730	CP022785_01416	99.37% IS1405_aa1	100% CP022785_01089	IS5 ssgr IS5	1762705	1761740	966
SL3730	CP022785_00098	100% IS1420_aa1	100% CP022785_00474	IS5 ssgr IS903	116159	117115	957
SL3730	CP022785_00474	100% IS1420_aa1	100% CP022785_00098	IS5 ssgr IS903	59802	598976	957
SL3730	CP022785_00827	43.19% ISWz1_aa1	No hit	IS91	1033112	1033654	543
SL3730	CP022785_00844	62.5% ISAba32_aa1	No hit	ISNCY ssgr IS1202	1049360	1049145	216
SL3730	CP022785_00845	73.76% ISKpn21_aa1	No hit	ISNCY ssgr IS1202	1050582	1049503	1.08
12D	IS_4fd65f85_00460	100% ISRme13_aa1	No hit	IS3 ssgr IS3	50748	507776	297
12D	IS_4fd65f85_00461	100% ISRme13_aa2	No hit	IS3 ssgr IS3	507773	508666	894
12D	IS_4fd65f85_00044	39.36% ISGur11_aa1	No hit	IS481	52457	49908	2.55
12D	IS_4fd65f85_00973	41.36% ISShvi3_aa1	40.83% IS_4fd65f85_00424	IS91	1086897	1085704	1.194
12D	IS_4fd65f85_00974	58.62% ISKpn21_aa2	No hit	ISNCY ssgr IS1202	1087573	1086932	642
12D	IS_4fd65f85_00971	75.82% ISPa42_aa1	37.91% IS_4fd65f85_01004	Tn3	1082211	1085210	3
12D	IS_4fd65f85_01004	93.16% ISPsy30_aa3	37.73% IS_4fd65f85_00971	Tn3	1116128	1113099	3.03
12D	IS_4fd65f85_01005	93.68% ISPsy30_aa2	No hit	Tn3	1116718	1116125	594
DTP0602	IS_79ead2f9_01864	56.26% ISGdi7_aa1	No hit	IS110 ssgr IS1111	2058633	2057485	1.149
DTP0602	IS_79ead2f9_00615	66.26% ISMyca1_aa1	100% IS_79ead2f9_01859	IS1634	657615	655873	1.743

DTP0602	IS_79ead2f9_01098	66.72% ISMyca1_aa1	99.82% IS_79ead2f9_02306	IS1634	1203199	1204935	1.737
DTP0602	IS_79ead2f9_01859	66.26% ISMyca1_aa1	100% IS_79ead2f9_00615	IS1634	2052482	2050740	1.743
DTP0602	IS_79ead2f9_02257	66.72% ISMyca1_aa1	100% IS_79ead2f9_02306	IS1634	2462879	2464615	1.737
DTP0602	IS_79ead2f9_02306	66.72% ISMyca1_aa1	100% IS_79ead2f9_02257	IS1634	2521543	2523279	1.737
DTP0602	IS_79ead2f9_01119	84.87% IS606_aa1	No hit	IS200/IS605	1230204	1229842	363
DTP0602	IS_79ead2f9_02577	57.44% ISHaha8_aa2	No hit	IS200/IS605 ssgr IS1341	2824540	2823482	1.059
DTP0602	IS_79ead2f9_00013	97.67% ISRta2_aa1	95.34% IS_79ead2f9_02333	IS256	16842	17102	261
DTP0602	IS_79ead2f9_02258	86.20% ISBcen18_aa1	No hit	IS256	2465261	2464908	354
DTP0602	IS_79ead2f9_02333	93.15% ISRta2_aa1	95.34% IS_79ead2f9_00013	IS256	2549980	2550990	1.011
DTP0602	IS_79ead2f9_01068	76.84% ISPosp5_aa2	No hit	IS3 ssgr IS3	1164810	1163926	885
DTP0602	IS_79ead2f9_01069	84.04% ISKpn18_aa1	No hit	IS3 ssgr IS3	1165103	1164807	297
DTP0602	IS_79ead2f9_02175	74.44% ISNmu2_aa1	No hit	IS3 ssgr IS407	2373355	2372897	459
DTP0602	IS_79ead2f9_01204	68.17% ISRso13_aa1	No hit	IS4 ssgr IS4	1317599	1316223	1.377
DTP0602	IS_79ead2f9_00230	56.22% ISGvi2_aa1	100% IS_79ead2f9_02074	IS4 ssgr IS50	258212	259603	1.392
DTP0602	IS_79ead2f9_00243	57.41% ISGvi2_aa1	100% IS_79ead2f9_02554	IS4 ssgr IS50	270537	269401	1.137
DTP0602	IS_79ead2f9_00305	56.22% ISGvi2_aa1	100% IS_79ead2f9_02074	IS4 ssgr IS50	334757	333366	1.392
DTP0602	IS_79ead2f9_00657	56% ISGvi2_aa1	99.78% IS_79ead2f9_02225	IS4 ssgr IS50	704902	703511	1.392
DTP0602	IS_79ead2f9_01968	56.22% ISGvi2_aa1	100% IS_79ead2f9_02074	IS4 ssgr IS50	2172835	2174226	1.392
DTP0602	IS_79ead2f9_02074	56.22% ISGvi2_aa1	100% IS_79ead2f9_01968	IS4 ssgr IS50	2283851	2285242	1.392
DTP0602	IS_79ead2f9_02225	55.77% ISGvi2_aa1	99.78% IS_79ead2f9_00657	IS4 ssgr IS50	2427199	2425808	1.392
DTP0602	IS_79ead2f9_02554	57.41% ISGvi2_aa1	100% IS_79ead2f9_00243	IS4 ssgr IS50	2797290	2796154	1.137
DTP0602	IS_79ead2f9_00968	70.83% ISRel13_aa1	No hit	IS5 ssgr IS427	1052986	1052618	369
DTP0602	IS_79ead2f9_00922	83.50% ISBps3_aa1	No hit	IS5 ssgr IS903	998932	998075	858
DTP0602	IS_79ead2f9_00523	74.44% ISBmu8_aa1	100% IS_79ead2f9_00984	IS630	557182	558267	1.086
DTP0602	IS_79ead2f9_00816	76.25% ISThsp15_aa1	96.62% IS_79ead2f9_00902	IS630	891203	892285	1.083
DTP0602	IS_79ead2f9_00902	76.85% ISThsp15_aa1	96.62% IS_79ead2f9_00816	IS630	975401	974421	981
DTP0602	IS_79ead2f9_00950	78.29% ISAzo30_aa1	100% IS_79ead2f9_01183	IS630	1030977	1029943	1.035
DTP0602	IS_79ead2f9_00984	74.44% ISBmu8_aa1	100% IS_79ead2f9_00523	IS630	1071069	1072154	1.086
DTP0602	IS_79ead2f9_01009	74.64% ISThsp15_aa1	98.63% IS_79ead2f9_00816	IS630	1095513	1095289	225
DTP0602	IS_79ead2f9_01010	77.70% ISThsp15_aa1	97.45% IS_79ead2f9_00816	IS630	1096270	1095797	474
DTP0602	IS_79ead2f9_01181	58.46% ISBmu8_aa1	59.06% IS_79ead2f9_00984	IS630	1293030	1293656	627
DTP0602	IS_79ead2f9_01182	68% ISPsy1_aa1	60% IS_79ead2f9_00984	IS630	1293653	1293889	237
DTP0602	IS_79ead2f9_01183	78.29% ISAzo30_aa1	100% IS_79ead2f9_00950	IS630	1294142	1295176	1.035
DTP0602	IS_79ead2f9_01373	47.74% ISTth6_aa1	No hit	IS630	1493159	1493626	468
DTP0602	IS_79ead2f9_00383	60.60% ISEc22_aa1	No hit	IS66	414198	414623	426
DTP0602	IS_79ead2f9_00384	90.43% IS883_aa2	No hit	IS66	41462	414967	348
DTP0602	IS_79ead2f9_00385	78.48% IS883_aa3	No hit	IS66	41501	416584	1.575
DTP0602	IS_79ead2f9_00955	79.87% ISBte1_aa2	No hit	ISKra4 ssgr ISAzba1	1036511	1036981	471
DTP0602	IS_79ead2f9_00900	80.60% ISSm4_aa2	No hit	ISL3	972937	973764	828
DTP0602	IS_79ead2f9_01369	65.98% ISKpn21_aa1	88.74% IS_79ead2f9_01375	ISNCY ssgr IS1202	1491566	1490832	735
DTP0602	IS_79ead2f9_01371	69.75% ISKpn21_aa1	No hit	ISNCY ssgr IS1202	1492626	1492006	621
DTP0602	IS_79ead2f9_01375	73.87% ISKpn21_aa1	88.74% IS_79ead2f9_01369	ISNCY ssgr IS1202	1496746	1495931	816
DTP0602	IS_79ead2f9_01150	53.00% TnAs1_aa2	79.12% IS_79ead2f9_01153	Tn3	1255777	1256343	567

DTP0602	IS_79ead2f9_01153	52.46% TnAs1_aa2	79.12% IS_79ead2f9_01150	Tn3	1257625	1258212	588
DTP0602	IS_79ead2f9_01266	48.20% ISPa42_aa2	No hit	Tn3	1381170	1380043	1.128
FC1138	IS_530769a3_00796	45.83% ISPpr6_aa1	No hit	IS1595 ssgr ISPna2	88338	882292	1.089
FC1138	IS_530769a3_00367	98.46% ISRme4_aa2	100% IS_530769a3_01104	IS21	429992	429207	786
FC1138	IS_530769a3_00368	80.30% IS1600_aa1	99.01% IS_530769a3_01103	IS21	431499	429982	1.518
FC1138	IS_530769a3_00467	98.46% ISRme4_aa2	100% IS_530769a3_01104	IS21	526087	525302	786
FC1138	IS_530769a3_00468	80.88% IS1600_aa1	100% IS_530769a3_01103	IS21	527603	526077	1.527
FC1138	IS_530769a3_00792	98.46% ISRme4_aa2	100% IS_530769a3_01104	IS21	877724	876939	786
FC1138	IS_530769a3_00793	80.88% IS1600_aa1	100% IS_530769a3_01103	IS21	87924	877714	1.527
FC1138	IS_530769a3_01103	80.88% IS1600_aa1	100% IS_530769a3_00793	IS21	1219762	1221288	1.527
FC1138	IS_530769a3_01104	98.46% ISRme4_aa2	100% IS_530769a3_00792	IS21	1221278	1222063	786
FC1138	IS_530769a3_00051	100% ISRme13_aa1	100% IS_530769a3_01699	IS3 ssgr IS3	54177	54473	297
FC1138	IS_530769a3_00052	100% ISRme13_aa2	100% IS_530769a3_01700	IS3 ssgr IS3	5447	55363	894
FC1138	IS_530769a3_00786	85.19% IS222_aa2	52.47% IS_530769a3_00470	IS3 ssgr IS3	869072	869908	837
FC1138	IS_530769a3_01101	60.69% ISMco1_aa2	54.95% IS_530769a3_01700	IS3 ssgr IS3	1219059	1218448	612
FC1138	IS_530769a3_01699	100% ISRme13_aa1	100% IS_530769a3_00051	IS3 ssgr IS3	1837842	1838138	297
FC1138	IS_530769a3_01700	100% ISRme13_aa2	100% IS_530769a3_00052	IS3 ssgr IS3	1838135	1839028	894
FC1138	IS_530769a3_00483	83.52% ISMdi3_aa1	91.30% IS_530769a3_00776	IS3 ssgr IS407	541457	541735	279
FC1138	IS_530769a3_00776	76.74% ISMdi3_aa1	91.30% IS_530769a3_00483	IS3 ssgr IS407	861107	861385	279
FC1138	IS_530769a3_00777	81.66% ISMdi3_aa2	98.07% IS_530769a3_00484	IS3 ssgr IS407	861415	861987	573
FC1138	IS_530769a3_00778	83.33% ISSpwi1_aa3	46.75% IS_530769a3_01700	IS3 ssgr IS407	861965	862261	297
FC1138	IS_530769a3_00470	89.25% ISAisp2_aa2	59.10% IS_530769a3_01700	IS3 ssgr IS51	529291	528479	813
FC1138	IS_530769a3_00471	80.43% ISAisp2_aa1	64.04% IS_530769a3_01699	IS3 ssgr IS51	529629	529351	279
FC1138	IS_530769a3_01702	69.02% IS1086_aa1	No hit	IS30	1840485	1839415	1.071
FC1138	IS_530769a3_00472	100% ISBvi1_aa1	No hit	IS4 ssgr IS4	529819	531018	1.2
FC1138	IS_530769a3_00568	81.13% ISPsp3_aa1	No hit	IS5 ssgr IS5	643773	644753	981
FC1138	IS_530769a3_00791	64.86% ISBcen19_aa1	No hit	IS66	87656	876898	339
FC1138	IS_530769a3_00389	44.88% ISWz1_aa1	43.24% IS_530769a3_01472	IS91	446883	44518	1.704
SL3022	CP023017_01554	88.75% ISBcen4_aa1	No hit	IS110 ssgr IS1111	1938565	1939584	1.02
SL3022	CP023017_01559	50.44% ISLsp2_aa1	No hit	IS1595 ssgr ISPna2	1945134	1943857	1.278
SL3022	CP023017_01423	92.85% ISRso8_aa1	No hit	IS3	1765738	1765433	306
SL3022	CP023017_01553	97.87% ISRso10_aa1	No hit	IS3 ssgr IS2	1938210	1938590	381
SL3022	CP023017_00934	85.05% ISGau4_aa1	100% CP023017_01585	IS3 ssgr IS407	1205911	1206177	267
SL3022	CP023017_00935	73.26% ISGau4_aa2	100% CP023017_00993	IS3 ssgr IS407	1206210	1207031	822
SL3022	CP023017_00993	73.26% ISGau4_aa2	100% CP023017_00935	IS3 ssgr IS407	1284930	1284109	822
SL3022	CP023017_00994	85.05% ISGau4_aa1	100% CP023017_01585	IS3 ssgr IS407	1285229	1284963	267
SL3022	CP023017_01585	85.05% ISGau4_aa1	100% CP023017_00994	IS3 ssgr IS407	1981371	1981637	267
SL3022	CP023017_01586	73.62% ISGau4_aa2	99.26% CP023017_00993	IS3 ssgr IS407	1981670	1982491	822
SL3022	CP023017_01558	80.95% ISBcen26_aa1	No hit	IS481	1943362	1943712	351
SL3022	CP023017_00130	100% IS1421_aa1	100% CP023017_01555	IS5 ssgr IS427	164986	164582	405
SL3022	CP023017_01555	100% IS1421_aa1	100% CP023017_00130	IS5 ssgr IS427	1940110	1940493	384

SL3022	CP023017_00038	63.71% IS1405_aa1	No hit	IS5 ssgr IS5	46547	46945	399
SL3022	CP023017_01556	81.37% ISCARN39_aa1	No hit	IS630	1941000	1941311	312
SL3022	CP023017_01549	82.88% ISSal1_aa2	No hit	IS66	1934869	1934480	390
SL3022	CP023017_00853	43.95% ISWz1_aa1	90.27% CP023017_00799	IS91	1105775	1104075	1.701
SL3022	CP023017_00772	63.15% ISKpn21_aa1	79.66% CP023017_00842	ISNCY ssgr IS1202	997242	997033	210
SL3022	CP023017_00841	72.53% ISKpn21_aa1	86.36% CP023017_00842	ISNCY ssgr IS1202	1080058	1079075	984
SL3022	CP023017_00842	71.39% ISKpn21_aa1	86.36% CP023017_00841	ISNCY ssgr IS1202	1081681	1080209	1.473
SL3022	CP023017_00926	61.97% ISKpn21_aa1	85.91% CP023017_00842	ISNCY ssgr IS1202	1195728	1196066	339
SL3022	CP023017_01597	52.72% ISMpo10_aa3	No hit	Tn3	1993055	1994158	1.104
SL2064	IS_c0f1f2ff_01362	92.85% ISRso8_aa1	No hit	IS3	1695505	1695200	306
SL2064	IS_c0f1f2ff_00906	85.05% ISGau4_aa1	65.21% IS_c0f1f2ff_01328	IS3 ssgr IS407	1146178	1146444	267
SL2064	IS_c0f1f2ff_00907	73.26% ISGau4_aa2	No hit	IS3 ssgr IS407	1146477	1147298	822
SL2064	IS_c0f1f2ff_00038	63.71% IS1405_aa1	No hit	IS5 ssgr IS5	46558	46956	399
SL2064	IS_c0f1f2ff_00836	87.71% ISCARN25_aa1	No hit	IS630	1056873	1057109	237
SL2064	IS_c0f1f2ff_01100	72.72% ISDge1_aa1	No hit	IS701	1408082	1408993	912
SL2064	IS_c0f1f2ff_00823	43.95% ISWz1_aa1	94.48% IS_c0f1f2ff_00789	IS91	1040165	1038465	1.701
SL2064	IS_c0f1f2ff_01270	54.26% ISSm4_aa2	No hit	ISL3	1606362	1605340	1.023
SL2064	IS_c0f1f2ff_00742	63.15% ISKpn21_aa1	83.33% IS_c0f1f2ff_00897	ISNCY ssgr IS1202	924992	924783	210
SL2064	IS_c0f1f2ff_00812	72.53% ISKpn21_aa1	No hit	ISNCY ssgr IS1202	1016060	1015077	984
SL2064	IS_c0f1f2ff_00897	61.97% ISKpn21_aa1	83.33% IS_c0f1f2ff_00742	ISNCY ssgr IS1202	1135991	1136329	339
SL2312	CP022797_01393	94.04% ISRso8_aa1	No hit	IS3	1793326	1793021	306
SL2312	CP022797_01390	72.46% ISDet2_aa2	No hit	IS3 ssgr IS407	1791657	1791887	231
SL2312	CP022797_01391	83.67% ISDet2_aa2	No hit	IS3 ssgr IS407	1791902	1792243	342
SL2312	CP022797_00357	36.11% ISMva2_aa1	No hit	IS481	447277	445028	2.25
SL2312	CP022797_01171	90.16% IS1421_aa1	No hit	IS5 ssgr IS427	1545922	1545734	189
SL2312	CP022797_01381	77.41% ISNGR9_aa2	No hit	IS5 ssgr IS427	1780513	1780136	378
SL2312	CP022797_00038	63.71% IS1405_aa1	No hit	IS5 ssgr IS5	46548	46946	399
SL2312	CP022797_01386	92.01% ISRso9_aa1	No hit	IS5 ssgr IS5	1784900	1785730	831
SL2312	CP022797_01417	84.37% ISAau3_aa1	No hit	IS5 ssgr IS5	1825626	1825357	270
SL2312	CP022797_00854	43.24% ISShvi3_aa1	95.16% CP022797_00801	IS91	1127392	1125692	1.701
SL2312	CP022797_00844	72% ISKpn21_aa1	No hit	ISNCY ssgr IS1202	1110583	1109546	1.038
SL2312	CP022797_00925	60.56% ISKpn21_aa1	No hit	ISNCY ssgr IS1202	1217743	1218081	339
SL2312	CP022797_01384	54.43% ISMpo10_aa3	No hit	Tn3	1783766	1782387	1.38
T101	CP022757_00688	95.40% ISRso14_aa2	65.51% CP022757_02400	IS3 ssgr IS407	743338	742724	615
T101	CP022757_00700	100% ISRso12_aa2	75% CP022757_02400	IS3 ssgr IS407	755082	754378	705
T101	CP022757_01463	100% ISRso12_aa1	88.63% CP022757_02401	IS3 ssgr IS407	1581193	1581459	267
T101	CP022757_01464	94.81% ISRso12_aa2	94.81% CP022757_00700	IS3 ssgr IS407	1581590	1582120	531
T101	CP022757_02400	97.33% ISRso16_aa2	75% CP022757_00700	IS3 ssgr IS407	2635970	2635179	792
T101	CP022757_02401	98.86% ISRso16_aa1	88.63% CP022757_01463	IS3 ssgr IS407	2636320	2636054	267
T101	CP022757_02591	74.71% ISAtu5_aa1	60.49% CP022757_02401	IS3 ssgr IS407	2842177	2842398	222

T101	CP022757_00701	99.63% ISRso1_aa1	96% CP022757_02167	IS5	756075	755251	825
T101	CP022757_02164	94.66% ISRso1_aa1	97.33% CP022757_02167	IS5	2384278	2384051	228
T101	CP022757_02167	96% ISRso1_aa1	97.33% CP022757_02164	IS5	2387510	2387737	228
T101	CP022757_02988	56.77% IS1421_aa3	No hit	IS5 ssgr IS427	3261520	3260894	627
T101	CP022757_02399	80.30% ISCARN25_aa1	81.25% CP022757_02601	IS630	2634508	2634741	234
T101	CP022757_02601	84.21% ISCARN25_aa1	81.25% CP022757_02399	IS630	2859114	2859368	255
T101	CP022757_00286	47.33% ISShvi3_aa1	41.36% CP022757_02149	IS91	310739	309648	1.092
T101	CP022757_00814	47.15% ISMno23_aa1	52.59% CP022757_03170	IS91	87574	874814	927
T101	CP022757_03170	48.75% ISWz1_aa1	52.76% CP022757_00814	IS91	3474806	3475795	990
T101	CP022757_00036	40.42% ISKpn25_aa1	44.62% CP022757_02159	ISL3	44095	4554	1.446
T101	CP022757_02159	40.29% ISKpn25_aa1	44.62% CP022757_00036	ISL3	2378508	2380127	1.62
T101	CP022757_00004	46.61% ISPa4_aa1	No hit	ISNCY	8684	6594	2.091
T101	CP022757_01744	71.81% ISKpn21_aa1	No hit	ISNCY ssgr IS1202	1921052	1920033	1.02
T101	CP022757_00189	50.92% ISThsp9_aa1	No hit	Tn3	206227	204887	1.341
T101	CP022757_00614	53.33% TnShfr1_aa1	No hit	Tn3	653545	654756	1.212
T101	CP022757_01669	77.39% ISPa42_aa2	No hit	Tn3	1808962	1808564	399
T82	CP022764_01393	94.04% ISRso8_aa1	No hit	IS3	1793328	1793023	306
T82	CP022764_01390	72.46% ISDet2_aa2	No hit	IS3 ssgr IS407	1791659	1791889	231
T82	CP022764_01391	83.67% ISDet2_aa2	No hit	IS3 ssgr IS407	1791904	1792245	342
T82	CP022764_00359	36.11% ISMva2_aa1	No hit	IS481	447277	445028	2.25
T82	CP022764_01169	90.16% IS1421_aa1	No hit	IS5 ssgr IS427	1545941	1545753	189
T82	CP022764_01381	77.41% ISNGR9_aa2	No hit	IS5 ssgr IS427	1780515	1780138	378
T82	CP022764_00038	63.71% IS1405_aa1	No hit	IS5 ssgr IS5	46548	46946	399
T82	CP022764_01386	92.01% ISRso9_aa1	No hit	IS5 ssgr IS5	1784902	1785732	831
T82	CP022764_01418	84.37% ISAau3_aa1	No hit	IS5 ssgr IS5	1825628	1825359	270
T82	CP022764_00855	43.24% ISShvi3_aa1	95.16% CP022764_00802	IS91	1127397	1125697	1.701
T82	CP022764_00845	72% ISKpn21_aa1	No hit	ISNCY ssgr IS1202	1110588	1109551	1.038
T82	CP022764_00923	60.56% ISKpn21_aa1	No hit	ISNCY ssgr IS1202	1217759	1218097	339
T82	CP022764_01384	54.43% ISMpo10_aa3	No hit	Tn3	1783768	1782389	1.38
T95	CP022762_01360	92.85% ISRso8_aa1	No hit	IS3	1695523	1695218	306
T95	CP022762_00903	85.05% ISGau4_aa1	65.21% CP022762_01326	IS3 ssgr IS407	1146195	1146461	267
T95	CP022762_00904	73.26% ISGau4_aa2	No hit	IS3 ssgr IS407	1146494	1147315	822
T95	CP022762_00038	63.71% IS1405_aa1	No hit	IS5 ssgr IS5	46558	46956	399
T95	CP022762_00834	87.71% ISCARN25_aa1	No hit	IS630	1056890	1057126	237
T95	CP022762_01097	72.72% ISDge1_aa1	No hit	IS701	1408099	1409010	912
T95	CP022762_00821	43.95% ISWz1_aa1	94.48% CP022762_00787	IS91	1040182	1038482	1.701
T95	CP022762_01267	54.26% ISSm4_aa2	No hit	ISL3	1606380	1605358	1.023
T95	CP022762_00740	63.15% ISKpn21_aa1	83.33% CP022762_00894	ISNCY ssgr IS1202	925009	9248	210
T95	CP022762_00810	72.53% ISKpn21_aa1	No hit	ISNCY ssgr IS1202	1016077	1015094	984
T95	CP022762_00894	61.97% ISKpn21_aa1	83.33% CP022762_00740	ISNCY ssgr IS1202	1136008	1136346	339

12J	IS_93519229_00055	100% ISRme13_aa1	No hit	IS3 ssgr IS3	63686	63982	297
12J	IS_93519229_00056	100% ISRme13_aa2	No hit	IS3 ssgr IS3	63979	64872	894
12J	IS_93519229_00809	39.36% ISGur11_aa1	No hit	IS481	9109	908351	2.55
12J	IS_93519229_00425	87.25% ISAzo23_aa1	No hit	IS5 ssgr IS1031	46718	467986	807
12J	IS_93519229_00570	41.36% ISShvi3_aa1	40.83% IS_93519229_00020	IS91	643962	642769	1.194
12J	IS_93519229_00571	58.62% ISKpn21_aa2	No hit	ISNCY ssgr IS1202	644638	643997	642
12J	IS_93519229_00568	75.82% ISPa42_aa1	No hit	Tn3	639276	642275	3
ATCC49129	IS_4adf2139_00370	44.88% ISWz1_aa1	43.47% IS_4adf2139_01318	IS91	438322	436619	1.704
ATCC49129	IS_4adf2139_00451	83.33% ISDet2_aa2	No hit	IS3 ssgr IS407	520861	520328	534
T51	IS_28b06e31_01363	92.85% ISRso8_aa1	No hit	IS3	1695516	1695211	306
T51	IS_28b06e31_00906	85.05% ISGau4_aa1	65.21% IS_28b06e31_01329	IS3 ssgr IS407	1146187	1146453	267
T51	IS_28b06e31_00907	73.26% ISGau4_aa2	No hit	IS3 ssgr IS407	1146486	1147307	822
T51	IS_28b06e31_00038	63.71% IS1405_aa1	No hit	IS5 ssgr IS5	46568	46966	399
T51	IS_28b06e31_00837	87.71% ISCARN25_aa1	No hit	IS630	1056882	1057118	237
T51	IS_28b06e31_01100	72.72% ISDge1_aa1	No hit	IS701	1408091	1409002	912
T51	IS_28b06e31_00824	43.95% ISWz1_aa1	94.48% IS_28b06e31_00790	IS91	1040174	1038474	1.701
T51	IS_28b06e31_01270	54.26% ISSm4_aa2	No hit	ISL3	1606373	1605351	1.023
T51	IS_28b06e31_00743	63.15% ISKpn21_aa1	83.33% IS_28b06e31_00897	ISNCY ssgr IS1202	925001	924792	210
T51	IS_28b06e31_00813	72.53% ISKpn21_aa1	No hit	ISNCY ssgr IS1202	1016069	1015086	984
T51	IS_28b06e31_00897	61.97% ISKpn21_aa1	83.33% IS_28b06e31_00743	ISNCY ssgr IS1202	1136000	1136338	339
A2HRMARDI	CP019912_00001	94.31% ISBcen18_aa1	100% CP019912_01132	IS256	131	33	1.278
A2HRMARDI	CP019912_00003	95.83% ISBcen18_aa1	100% CP019912_01132	IS256	2306	2596	291
A2HRMARDI	CP019912_00004	94.31% ISBcen18_aa1	100% CP019912_01132	IS256	266	3937	1.278
A2HRMARDI	CP019912_00120	94.31% ISBcen18_aa1	100% CP019912_01132	IS256	143401	142124	1.278
A2HRMARDI	CP019912_00144	94.31% ISBcen18_aa1	100% CP019912_01132	IS256	168231	166954	1.278
A2HRMARDI	CP019912_00146	94.31% ISBcen18_aa1	100% CP019912_01132	IS256	169693	17097	1.278
A2HRMARDI	CP019912_00171	94.31% ISBcen18_aa1	100% CP019912_01132	IS256	198871	197594	1.278
A2HRMARDI	CP019912_00173	94.31% ISBcen18_aa1	100% CP019912_01132	IS256	199573	20085	1.278
A2HRMARDI	CP019912_00186	94.31% ISBcen18_aa1	100% CP019912_01132	IS256	214972	216249	1.278
A2HRMARDI	CP019912_00215	94.31% ISBcen18_aa1	100% CP019912_01132	IS256	249087	250364	1.278
A2HRMARDI	CP019912_00268	94.31% ISBcen18_aa1	100% CP019912_01132	IS256	304471	305748	1.278
A2HRMARDI	CP019912_00437	94.31% ISBcen18_aa1	100% CP019912_01132	IS256	49424	495517	1.278
A2HRMARDI	CP019912_00438	90.27% ISBcen18_aa1	100% CP019912_01132	IS256	495787	495338	450
A2HRMARDI	CP019912_00450	95.38% ISBcen18_aa1	100% CP019912_01132	IS256	50509	504113	978
A2HRMARDI	CP019912_00544	94.31% ISBcen18_aa1	100% CP019912_01132	IS256	607083	605806	1.278
A2HRMARDI	CP019912_00808	95.52% ISBcen18_aa1	100% CP019912_01132	IS256	88298	883225	246
A2HRMARDI	CP019912_00989	94.31% ISBcen18_aa1	100% CP019912_01132	IS256	1067528	1066251	1.278
A2HRMARDI	CP019912_01060	94.31% ISBcen18_aa1	100% CP019912_01132	IS256	1149932	1151209	1.278
A2HRMARDI	CP019912_01132	94.31% ISBcen18_aa1	100% CP019912_01060	IS256	1246762	1245485	1.278
A2HRMARDI	CP019912_00451	96.90% ISRso8_aa1	No hit	IS3	505738	505445	294

A2HRMARDI	CP019912_00118	97.5% ISRso10_aa2	100% CP019912_00441	IS3 ssgr IS2	141173	140571	603
A2HRMARDI	CP019912_00119	99.18% ISRso10_aa1	100% CP019912_01161	IS3 ssgr IS2	141805	141416	390
A2HRMARDI	CP019912_00142	98% ISRso10_aa2	100% CP019912_01162	IS3 ssgr IS2	166003	165401	603
A2HRMARDI	CP019912_00143	99.18% ISRso10_aa1	100% CP019912_01161	IS3 ssgr IS2	166635	166246	390
A2HRMARDI	CP019912_00440	99.18% ISRso10_aa1	100% CP019912_01161	IS3 ssgr IS2	49676	497149	390
A2HRMARDI	CP019912_00441	97.5% ISRso10_aa2	100% CP019912_00118	IS3 ssgr IS2	497392	497994	603
A2HRMARDI	CP019912_00809	98% ISRso10_aa2	100% CP019912_01162	IS3 ssgr IS2	883813	883211	603
A2HRMARDI	CP019912_00810	99.18% ISRso10_aa1	100% CP019912_01161	IS3 ssgr IS2	884445	884056	390
A2HRMARDI	CP019912_00905	98% ISRso10_aa2	100% CP019912_01162	IS3 ssgr IS2	985601	984999	603
A2HRMARDI	CP019912_00906	99.18% ISRso10_aa1	100% CP019912_01161	IS3 ssgr IS2	986233	985844	390
A2HRMARDI	CP019912_00987	99.18% ISRso10_aa1	100% CP019912_01161	IS3 ssgr IS2	1065089	1065478	390
A2HRMARDI	CP019912_00988	98.14% ISRso10_aa2	91.44% CP019912_01162	IS3 ssgr IS2	1065721	1066404	684
A2HRMARDI	CP019912_01161	99.18% ISRso10_aa1	100% CP019912_00987	IS3 ssgr IS2	1291204	1291593	390
A2HRMARDI	CP019912_01162	98% ISRso10_aa2	100% CP019912_00905	IS3 ssgr IS2	1291836	1292438	603
A2HRMARDI	CP019912_00002	96.95% ISRso16_aa2	100% CP019912_01108	IS3 ssgr IS407	1482	2273	792
A2HRMARDI	CP019912_00216	98.73% ISRso16_aa1	100% CP019912_01107	IS3 ssgr IS407	250643	250185	459
A2HRMARDI	CP019912_00403	98.86% ISRso16_aa1	100% CP019912_01107	IS3 ssgr IS407	450121	450387	267
A2HRMARDI	CP019912_00404	97.14% ISRso16_aa2	100% CP019912_01108	IS3 ssgr IS407	45042	451262	843
A2HRMARDI	CP019912_00442	97.14% ISRso16_aa2	100% CP019912_01108	IS3 ssgr IS407	499843	499001	843
A2HRMARDI	CP019912_00443	98.86% ISRso16_aa1	100% CP019912_01107	IS3 ssgr IS407	500142	499876	267
A2HRMARDI	CP019912_00530	98.86% ISRso16_aa1	100% CP019912_01107	IS3 ssgr IS407	59311	593376	267
A2HRMARDI	CP019912_00531	97.04% ISRso16_aa2	100% CP019912_01108	IS3 ssgr IS407	593409	594224	816
A2HRMARDI	CP019912_00532	98.86% ISRso16_aa1	100% CP019912_01107	IS3 ssgr IS407	594302	594568	267
A2HRMARDI	CP019912_00533	97.14% ISRso16_aa2	100% CP019912_01108	IS3 ssgr IS407	594601	595443	843
A2HRMARDI	CP019912_00611	98.86% ISRso16_aa1	100% CP019912_01107	IS3 ssgr IS407	676121	676387	267
A2HRMARDI	CP019912_00806	98.86% ISRso16_aa1	100% CP019912_01107	IS3 ssgr IS407	881786	882052	267
A2HRMARDI	CP019912_00807	97.14% ISRso16_aa2	100% CP019912_01108	IS3 ssgr IS407	882085	882927	843
A2HRMARDI	CP019912_01107	98.86% ISRso16_aa1	100% CP019912_00806	IS3 ssgr IS407	1221699	1221965	267
A2HRMARDI	CP019912_01108	97.14% ISRso16_aa2	100% CP019912_00807	IS3 ssgr IS407	1221998	1222840	843
A2HRMARDI	CP019912_00501	75.80% ISCARN14_aa1	98.55% CP019912_01061	IS5 ssgr IS1031	562831	563982	1.152
A2HRMARDI	CP019912_01061	74.85% ISCARN14_aa1	98.55% CP019912_00501	IS5 ssgr IS1031	1152285	1151056	1.23
A2HRMARDI	CP019912_01163	79.20% ISCARN14_aa1	100% CP019912_00501	IS5 ssgr IS1031	1292484	1293470	987
A2HRMARDI	CP019912_00005	70% ISMno18_aa2	No hit	IS5 ssgr IS427	3966	4184	219
A2HRMARDI	CP019912_00049	95.18% ISRso9_aa1	100% CP019912_01273	IS5 ssgr IS5	59329	58007	1.323
A2HRMARDI	CP019912_00430	95.41% ISRso9_aa1	100% CP019912_00995	IS5 ssgr IS5	484395	483073	1.323
A2HRMARDI	CP019912_00991	95.41% ISRso9_aa1	100% CP019912_00995	IS5 ssgr IS5	1068649	1069971	1.323
A2HRMARDI	CP019912_00995	95.41% ISRso9_aa1	100% CP019912_00991	IS5 ssgr IS5	1071901	1073223	1.323
A2HRMARDI	CP019912_01259	63.71% IS1405_aa1	No hit	IS5 ssgr IS5	1394658	1395056	399
A2HRMARDI	CP019912_01273	95.18% ISRso9_aa1	100% CP019912_00049	IS5 ssgr IS5	1408733	1410055	1.323
A2HRMARDI	CP019912_00116	90.26% ISCARN25_aa1	100% CP019912_00878	IS630	137691	13871	1.02
A2HRMARDI	CP019912_00121	90.26% ISCARN25_aa1	100% CP019912_00878	IS630	14493	143911	1.02
A2HRMARDI	CP019912_00201	90.26% ISCARN25_aa1	100% CP019912_00878	IS630	234886	235905	1.02
A2HRMARDI	CP019912_00212	90.26% ISCARN25_aa1	100% CP019912_00878	IS630	247583	246564	1.02

A2HRMARDI	CP019912_00217	91.85% ISCARN25_aa1	100% CP019912_00436	IS630	250779	251591	813
A2HRMARDI	CP019912_00267	83.84% ISCARN25_aa1	99.23% CP019912_01051	IS630	304037	304465	429
A2HRMARDI	CP019912_00405	91.85% ISCARN25_aa1	100% CP019912_00436	IS630	451325	452137	813
A2HRMARDI	CP019912_00436	91.85% ISCARN25_aa1	100% CP019912_00405	IS630	494154	493342	813
A2HRMARDI	CP019912_00444	91.2% ISCARN25_aa1	100% CP019912_01051	IS630	500243	50062	378
A2HRMARDI	CP019912_00459	89.97% ISCARN25_aa1	99.70% CP019912_00878	IS630	514012	515031	1.02
A2HRMARDI	CP019912_00878	90.26% ISCARN25_aa1	100% CP019912_00212	IS630	952682	953701	1.02
A2HRMARDI	CP019912_00898	82.10% ISCARN25_aa1	93.68% CP019912_01051	IS630	977596	978183	588
A2HRMARDI	CP019912_00899	92.5% ISCARN25_aa1	100% CP019912_00878	IS630	978132	978614	483
A2HRMARDI	CP019912_01051	90.26% ISCARN25_aa1	100% CP019912_00878	IS630	1140151	1139132	1.02
A2HRMARDI	CP019912_01156	72% ISCARN75_aa1	No hit	IS701	1284803	1283283	1.521
A2HRMARDI	CP019912_00125	66.99% ISKpn21_aa1	No hit	ISNCY ssgr IS1202	150609	150974	366
A2HRMARDI	CP019912_00213	65.57% ISKpn21_aa1	No hit	ISNCY ssgr IS1202	247796	248005	210

ORF Name	% of DNA Similarity	% of AAs Similarity (Replicon First hit)	Identified IS Family	ORF Left End	ORF Right End	ORF Size (bp)	ORF Size (aa)
SL2729	CP022793_00433	88.75% ISBcen4_aa1	92.70% CP022793_00486	IS110 ssgr IS1111	556224	557243	1.02
SL2729	CP022793_00486	84.67% ISBcen4_aa1	92.70% CP022793_00433	IS110 ssgr IS1111	620875	621291	417
SL2729	CP022793_01502	61.67% ISMno14_aa1	100% CP022793_01507	IS110 ssgr IS1111	1939401	1940414	1.014
SL2729	CP022793_01507	61.67% ISMno14_aa1	100% CP022793_01502	IS110 ssgr IS1111	1943993	1942980	1.014
SL2729	CP022793_01236	83.75% ISBusp4_aa1	No hit	IS1182	1585492	1584047	1.446
SL2729	CP022793_00485	95.74% ISRso10_aa1	No hit	IS3 ssgr IS2	62052	6209	381
SL2729	CP022793_01259	78.68% ISRso20_aa1	No hit	IS3 ssgr IS3	1632212	1632412	201
SL2729	CP022793_01503	93.82% ISRso16_aa2	44.58% CP022793_01051	IS3 ssgr IS407	1940761	1941300	540
SL2729	CP022793_01050	81.52% ISAisp2_aa1	97.82% CP022793_01222	IS3 ssgr IS51	1345736	1346014	279
SL2729	CP022793_01051	89.34% ISAisp2_aa2	98.28% CP022793_01221	IS3 ssgr IS51	1346011	1346886	876
SL2729	CP022793_01221	89.34% ISAisp2_aa2	98.28% CP022793_01051	IS3 ssgr IS51	1567655	1566780	876
SL2729	CP022793_01222	81.52% ISAisp2_aa1	97.82% CP022793_01050	IS3 ssgr IS51	1567930	1567652	279
SL2729	CP022793_00002	61.68% ISCro3_aa1	99.77% CP022793_00360	IS4	3518	219	1.329
SL2729	CP022793_00360	61.44% ISCro3_aa1	99.77% CP022793_00002	IS4	469739	468411	1.329
SL2729	CP022793_00565	98.54% ISRso1_aa1	85.67% CP022793_00589	IS5	751742	753175	1.434
SL2729	CP022793_00589	97.81% ISRso1_aa1	85.67% CP022793_00565	IS5	78479	782235	2.556
SL2729	CP022793_00659	97.81% ISRso1_aa1	100% CP022793_00589	IS5	86365	862826	825
SL2729	CP022793_01002	97.81% ISRso1_aa1	100% CP022793_00589	IS5	1297330	1298154	825
SL2729	CP022793_01037	98.54% ISRso1_aa1	98.54% CP022793_00589	IS5	1336404	1335580	825
SL2729	CP022793_01049	85.56% ISRso1_aa1	86.59% CP022793_00589	IS5	1345312	1345704	393
SL2729	CP022793_00225	99.25% IS1421_aa1	100% CP022793_01403	IS5 ssgr IS427	300556	30096	405
SL2729	CP022793_00482	99.25% IS1421_aa1	100% CP022793_01403	IS5 ssgr IS427	618041	617637	405
SL2729	CP022793_00661	99.25% IS1421_aa1	98.50% CP022793_01403	IS5 ssgr IS427	866111	865707	405
SL2729	CP022793_01403	99.25% IS1421_aa1	100% CP022793_00482	IS5 ssgr IS427	1800687	1800283	405
SL2729	CP022793_00031	99.06% IS1405_aa1	100% CP022793_01348	IS5 ssgr IS5	35898	34933	966
SL2729	CP022793_00043	94.51% IS1021_aa1	100% CP022793_00526	IS5 ssgr IS5	48316	49302	987
SL2729	CP022793_00046	99.06% IS1405_aa1	100% CP022793_01348	IS5 ssgr IS5	51109	52074	966

SL2729	CP022793_00081	94.51% IS1021_aa1	100% CP022793_00526	IS5 ssgr IS5	94013	93027	987
SL2729	CP022793_00086	99.37% IS1405_aa1	100% CP022793_01384	IS5 ssgr IS5	105081	104116	966
SL2729	CP022793_00167	94.51% IS1021_aa1	100% CP022793_00526	IS5 ssgr IS5	214734	213748	987
SL2729	CP022793_00169	94.51% IS1021_aa1	100% CP022793_00526	IS5 ssgr IS5	217671	216685	987
SL2729	CP022793_00224	94.51% IS1021_aa1	100% CP022793_00526	IS5 ssgr IS5	299386	300372	987
SL2729	CP022793_00252	99.06% IS1405_aa1	100% CP022793_01348	IS5 ssgr IS5	345474	344509	966
SL2729	CP022793_00292	99.06% IS1405_aa1	100% CP022793_01348	IS5 ssgr IS5	396405	39737	966
SL2729	CP022793_00462	99.06% IS1405_aa1	100% CP022793_01348	IS5 ssgr IS5	600061	601026	966
SL2729	CP022793_00470	99.06% IS1405_aa1	100% CP022793_01348	IS5 ssgr IS5	604414	605379	966
SL2729	CP022793_00488	99.06% IS1405_aa1	100% CP022793_01348	IS5 ssgr IS5	62234	621375	966
SL2729	CP022793_00526	94.51% IS1021_aa1	100% CP022793_00224	IS5 ssgr IS5	66103	660044	987
SL2729	CP022793_00617	99.06% IS1405_aa1	100% CP022793_01348	IS5 ssgr IS5	812365	81333	966
SL2729	CP022793_00817	99.06% IS1405_aa1	100% CP022793_01348	IS5 ssgr IS5	1065168	1064203	966
SL2729	CP022793_00823	99.06% IS1405_aa1	100% CP022793_01348	IS5 ssgr IS5	1071027	1071992	966
SL2729	CP022793_00983	99.06% IS1405_aa1	100% CP022793_01348	IS5 ssgr IS5	1263184	1264149	966
SL2729	CP022793_01062	99.37% IS1405_aa1	100% CP022793_01384	IS5 ssgr IS5	1356247	1357212	966
SL2729	CP022793_01159	99.06% IS1405_aa1	100% CP022793_01348	IS5 ssgr IS5	1492415	1493380	966
SL2729	CP022793_01348	99.06% IS1405_aa1	100% CP022793_01159	IS5 ssgr IS5	1743374	1744339	966
SL2729	CP022793_01384	99.37% IS1405_aa1	100% CP022793_01062	IS5 ssgr IS5	1782068	1781103	966
SL2729	CP022793_00097	100% IS1420_aa1	100% CP022793_00459	IS5 ssgr IS903	11617	117126	957
SL2729	CP022793_00459	100% IS1420_aa1	100% CP022793_00097	IS5 ssgr IS903	598071	599027	957
SL2729	CP022793_00805	43.40% ISWz1_aa1	44.13% CP022793_01553	IS91	1051282	1052982	1.701
SL2729	CP022793_00821	62.5% ISAba32_aa1	70% CP022793_00822	ISNCY ssgr IS1202	1068690	1068475	216
SL2729	CP022793_00822	72.27% ISKpn21_aa1	70% CP022793_00821	ISNCY ssgr IS1202	1070308	1068833	1.476
T110	IS_3f40740b_00199	87.92% ISBcen4_aa1	100% IS_3f40740b_01663	IS110 ssgr IS1111	195056	195856	801
T110	IS_3f40740b_00564	91.50% ISBcen4_aa1	100% IS_3f40740b_01662	IS110 ssgr IS1111	540415	540885	471
T110	IS_3f40740b_00565	85.95% ISBcen4_aa1	100% IS_3f40740b_00199	IS110 ssgr IS1111	540894	541433	540
T110	IS_3f40740b_00631	82.5% ISPa49_aa1	100% IS_3f40740b_01662	IS110 ssgr IS1111	598244	598495	252
T110	IS_3f40740b_00632	85.95% ISBcen4_aa1	100% IS_3f40740b_00199	IS110 ssgr IS1111	598504	599043	540
T110	IS_3f40740b_01662	91.50% ISBcen4_aa1	100% IS_3f40740b_00564	IS110 ssgr IS1111	1480469	1480939	471
T110	IS_3f40740b_01663	85.95% ISBcen4_aa1	100% IS_3f40740b_00199	IS110 ssgr IS1111	1480948	1481487	540
T110	IS_3f40740b_01746	90.58% ISPosp3_aa1	No hit	IS1595 ssgr ISSod11	1537131	1536184	948
T110	IS_3f40740b_00629	95.74% ISRso10_aa1	No hit	IS3 ssgr IS2	597671	598051	381
T110	IS_3f40740b_00633	92.30% ISRso10_aa2	No hit	IS3 ssgr IS2	599535	600197	663
T110	IS_3f40740b_02165	92.85% ISRso16_aa2	47.25% IS_3f40740b_01776	IS3 ssgr IS407	1951579	1951917	339
T110	IS_3f40740b_02168	91.96% ISRso16_aa2	No hit	IS3 ssgr IS407	1953384	1952899	486
T110	IS_3f40740b_00638	81.52% ISAisp2_aa1	97.82% IS_3f40740b_01777	IS3 ssgr IS51	603042	60332	279
T110	IS_3f40740b_00639	89.34% ISAisp2_aa2	98.28% IS_3f40740b_01776	IS3 ssgr IS51	603317	604192	876
T110	IS_3f40740b_01776	89.34% ISAisp2_aa2	98.28% IS_3f40740b_00639	IS3 ssgr IS51	1571604	1570729	876
T110	IS_3f40740b_01777	81.52% ISAisp2_aa1	97.82% IS_3f40740b_00638	IS3 ssgr IS51	1571879	1571601	279
T110	IS_3f40740b_00098	98.97% ISRso1_aa1	99.00% IS_3f40740b_00795	IS5	96099	95512	588
T110	IS_3f40740b_00794	98.23% ISRso1_aa1	65.07% IS_3f40740b_00098	IS5	74855	749152	603

T110	IS_3f40740b_00795	100% ISRso1_aa1	99.00% IS_3f40740b_00098	IS5	749202	749507	306
T110	IS_3f40740b_00710	99.50% IS1421_aa3	89.14% IS_3f40740b_00711	IS5 ssgr IS427	665374	664565	810
T110	IS_3f40740b_00711	85.88% IS1421_aa3	88.88% IS_3f40740b_00710	IS5 ssgr IS427	666825	666058	768
T110	IS_3f40740b_00047	81.15% ISRso9_aa1	No hit	IS5 ssgr IS5	44648	44863	216
T110	IS_3f40740b_00091	100% IS1405_aa1	100% IS_3f40740b_01415	IS5 ssgr IS5	88307	87819	489
T110	IS_3f40740b_00092	96.36% IS1405_aa1	97.57% IS_3f40740b_01779	IS5 ssgr IS5	88783	88265	519
T110	IS_3f40740b_00544	99.37% IS1405_aa1	100% IS_3f40740b_01779	IS5 ssgr IS5	522331	521366	966
T110	IS_3f40740b_00712	90.27% IS1021_aa1	79.24% IS_3f40740b_01415	IS5 ssgr IS5	667529	66714	390
T110	IS_3f40740b_00713	90.65% IS1021_aa1	79.43% IS_3f40740b_01415	IS5 ssgr IS5	667898	667563	336
T110	IS_3f40740b_00903	98.86% IS1405_aa1	100% IS_3f40740b_01416	IS5 ssgr IS5	833766	834041	276
T110	IS_3f40740b_00904	100% IS1405_aa1	100% IS_3f40740b_01415	IS5 ssgr IS5	834101	83473	630
T110	IS_3f40740b_01165	99.37% IS1405_aa1	100% IS_3f40740b_01779	IS5 ssgr IS5	1069363	1070328	966
T110	IS_3f40740b_01415	100% IS1405_aa1	100% IS_3f40740b_00904	IS5 ssgr IS5	1262940	1262311	630
T110	IS_3f40740b_01416	98.86% IS1405_aa1	100% IS_3f40740b_00903	IS5 ssgr IS5	1263275	1263000	276
T110	IS_3f40740b_01452	98.91% IS1405_aa1	100% IS_3f40740b_01779	IS5 ssgr IS5	1299402	1299998	597
T110	IS_3f40740b_01453	100% IS1405_aa1	100% IS_3f40740b_01415	IS5 ssgr IS5	1299956	1300366	411
T110	IS_3f40740b_01645	99.37% IS1405_aa1	100% IS_3f40740b_01779	IS5 ssgr IS5	1463105	1464070	966
T110	IS_3f40740b_01779	99.37% IS1405_aa1	100% IS_3f40740b_01645	IS5 ssgr IS5	1575938	1574973	966
T110	IS_3f40740b_01152	43.40% ISWz1_aa1	No hit	IS91	1056309	1058009	1.701
T110	IS_3f40740b_01173	62.5% ISAba32_aa1	70% IS_3f40740b_01175	ISNCY ssgr IS1202	1076690	1076475	216
T110	IS_3f40740b_01174	75.35% ISKpn21_aa1	No hit	ISNCY ssgr IS1202	1077805	1076819	987
T110	IS_3f40740b_01175	66.91% ISKpn21_aa1	70% IS_3f40740b_01173	ISNCY ssgr IS1202	1078305	1077775	531
SL3175	NZ_CP022789_01049	77.77% ISBusp4_aa1	No hit	IS1182	1329146	1328946	201
SL3175	NZ_CP022789_00005	98.85% IS407_aa1	95.40% NZ_CP022789_01443	IS3 ssgr IS407	4696	4959	264
SL3175	NZ_CP022789_00006	97.09% ISRso14_aa2	96.66% NZ_CP022789_01441	IS3 ssgr IS407	4986	5813	828
SL3175	NZ_CP022789_00504	98.86% ISRso16_aa1	72.28% NZ_CP022789_00005	IS3 ssgr IS407	623952	624218	267
SL3175	NZ_CP022789_00505	96.95% ISRso16_aa2	67.51% NZ_CP022789_00006	IS3 ssgr IS407	624302	625093	792
SL3175	NZ_CP022789_01441	98.33% ISRso14_aa2	96.66% NZ_CP022789_00006	IS3 ssgr IS407	1785445	1784897	549
SL3175	NZ_CP022789_01443	98.85% ISRso14_aa1	95.40% NZ_CP022789_00005	IS3 ssgr IS407	1786004	1785741	264
SL3175	NZ_CP022789_01324	91.72% ISBph2_aa1	No hit	IS5	1672068	1671397	672
SL3175	NZ_CP022789_00599	100% IS1421_aa1	74.50% NZ_CP022789_01436	IS5 ssgr IS427	728611	728255	357
SL3175	NZ_CP022789_01437	81.31% IS1421_aa3	No hit	IS5 ssgr IS427	1781512	1782060	549
SL3175	NZ_CP022789_00042	63.71% IS1405_aa1	No hit	IS5 ssgr IS5	47883	48281	399
SL3175	NZ_CP022789_01339	68.53% ISSm4_aa2	No hit	ISL3	1686727	1686305	423
SL3175	NZ_CP022789_00089	45.77% ISPa4_aa1	No hit	ISNCY	104459	106177	1.719
SL3175	NZ_CP022789_00757	65.57% ISKpn21_aa1	83.33% NZ_CP022789_00851	ISNCY ssgr IS1202	931244	931035	210
SL3175	NZ_CP022789_00850	66.01% ISKpn21_aa1	92.72% NZ_CP022789_00851	ISNCY ssgr IS1202	1037726	1037361	366
SL3175	NZ_CP022789_00851	72.27% ISKpn21_aa1	92.72% NZ_CP022789_00850	ISNCY ssgr IS1202	1039352	1037877	1.476
T98	NZ_CP022760_01043	77.77% ISBusp4_aa1	No hit	IS1182	1329155	1328955	201
T98	NZ_CP022760_00005	98.85% IS407_aa1	95.40% NZ_CP022760_01437	IS3 ssgr IS407	4696	4959	264
T98	NZ_CP022760_00006	97.09% ISRso14_aa2	96.66% NZ_CP022760_01435	IS3 ssgr IS407	4986	5813	828

T98	NZ_CP022760_00499	98.86% ISRso16_aa1	72.28% NZ_CP022760_00005	IS3 ssgr IS407	623962	624228	267
T98	NZ_CP022760_00500	96.95% ISRso16_aa2	67.51% NZ_CP022760_00006	IS3 ssgr IS407	624312	625103	792
T98	NZ_CP022760_01435	98.33% ISRso14_aa2	96.66% NZ_CP022760_00006	IS3 ssgr IS407	1785443	1784895	549
T98	NZ_CP022760_01437	98.85% ISRso14_aa1	95.40% NZ_CP022760_00005	IS3 ssgr IS407	1786002	1785739	264
T98	NZ_CP022760_01318	91.72% ISBph2_aa1	No hit	IS5	1672067	1671396	672
T98	NZ_CP022760_00594	100% IS1421_aa1	74.50% NZ_CP022760_01430	IS5 ssgr IS427	728621	728265	357
T98	NZ_CP022760_01431	81.31% IS1421_aa3	No hit	IS5 ssgr IS427	1781510	1782058	549
T98	NZ_CP022760_00042	63.71% IS1405_aa1	No hit	IS5 ssgr IS5	47883	48281	399
T98	NZ_CP022760_01333	68.53% ISSm4_aa2	No hit	ISL3	1686726	1686304	423
T98	NZ_CP022760_00089	45.77% ISPa4_aa1	No hit	ISNCY	104458	106176	1.719
T98	NZ_CP022760_00751	65.57% ISKpn21_aa1	83.33% NZ_CP022760_00844	ISNCY ssgr IS1202	931254	931045	210
T98	NZ_CP022760_00843	66.01% ISKpn21_aa1	92.72% NZ_CP022760_00844	ISNCY ssgr IS1202	1037737	1037372	366
T98	NZ_CP022760_00844	72.27% ISKpn21_aa1	92.72% NZ_CP022760_00843	ISNCY ssgr IS1202	1039363	1037888	1.476
T11	NZ_CP022777_01363	92.85% ISRso8_aa1	No hit	IS3	1694837	1694532	306
T11	NZ_CP022777_00904	85.05% ISGau4_aa1	65.21% NZ_CP022777_01330	IS3 ssgr IS407	1145543	1145809	267
T11	NZ_CP022777_00905	73.26% ISGau4_aa2	No hit	IS3 ssgr IS407	1145842	1146663	822
T11	NZ_CP022777_00038	63.71% IS1405_aa1	No hit	IS5 ssgr IS5	46599	46997	399
T11	NZ_CP022777_00835	87.71% ISCARN25_aa1	No hit	IS630	1056238	1056474	237
T11	NZ_CP022777_01100	72.72% ISDge1_aa1	No hit	IS701	1407444	1408355	912
T11	NZ_CP022777_00822	43.95% ISWz1_aa1	94.48% NZ_CP022777_00789	IS91	1039530	1037830	1.701
T11	NZ_CP022777_01271	54.26% ISSm4_aa2	No hit	ISL3	1605694	1604672	1.023
T11	NZ_CP022777_00741	63.15% ISKpn21_aa1	83.33% NZ_CP022777_00895	ISNCY ssgr IS1202	925031	924822	210
T11	NZ_CP022777_00811	72.53% ISKpn21_aa1	No hit	ISNCY ssgr IS1202	1015425	1014442	984
T11	NZ_CP022777_00895	61.97% ISKpn21_aa1	83.33% NZ_CP022777_00741	ISNCY ssgr IS1202	1135356	1135694	339
T12	CP022775_01458	94.04% ISRso8_aa1	No hit	IS3	1793155	1792850	306
T12	CP022775_01455	72.46% ISDet2_aa2	No hit	IS3 ssgr IS407	1791486	1791716	231
T12	CP022775_01456	83.67% ISDet2_aa2	No hit	IS3 ssgr IS407	1791731	1792072	342
T12	CP022775_00376	36.11% ISMva2_aa1	No hit	IS481	447243	444994	2.25
T12	CP022775_01228	90.16% IS1421_aa1	No hit	IS5 ssgr IS427	1545776	1545588	189
T12	CP022775_01446	77.41% ISNGR9_aa2	No hit	IS5 ssgr IS427	1780342	1779965	378
T12	CP022775_00039	63.71% IS1405_aa1	No hit	IS5 ssgr IS5	46539	46937	399
T12	CP022775_01451	92.01% ISRso9_aa1	No hit	IS5 ssgr IS5	1784729	1785559	831
T12	CP022775_01484	82.29% ISBmu20_aa1	No hit	IS5 ssgr IS5	1825543	1825178	366
T12	CP022775_00893	43.24% ISShvi3_aa1	95.16% CP022775_00839	IS91	1127295	1125595	1.701
T12	CP022775_00883	72% ISKpn21_aa1	No hit	ISNCY ssgr IS1202	1110489	1109452	1.038
T12	CP022775_00965	60.56% ISKpn21_aa1	No hit	ISNCY ssgr IS1202	1217644	1217982	339
T12	CP022775_01449	54.43% ISMpo10_aa3	No hit	Tn3	1783595	1782216	1.38
PSI07	RPSI07_mp0238	97.54% ISRso10_aa1	99.17% RPSI07_mp1440	IS3 ssgr IS2	286062	286451	390
PSI07	RPSI07_mp0239	95.93% ISRso10_aa2	100% RPSI07_mp1439	IS3 ssgr IS2	286409	287296	888
PSI07	RPSI07_mp1439	95.74% ISRso10_aa2	100% RPSI07_mp0239	IS3 ssgr IS2	1745335	1744487	849

PSI07	RPSI07_mp1440	98.34% ISRso10_aa1	99.17% RPSI07_mp0238	IS3 ssgr IS2	1745697	1745332	366
PSI07	RPSI07_mp1606	98.16% ISRso14_aa2	39.82% RPSI07_mp0239	IS3 ssgr IS407	1875941	1875114	828
PSI07	RPSI07_mp1607	98.85% ISRso14_aa1	No hit	IS3 ssgr IS407	1876221	1875958	264
PSI07	RPSI07_mp1457	91.72% ISBph2_aa1	No hit	IS5	1758688	1758017	672
PSI07	RPSI07_mp0668	76.07% ISCARN14_aa1	97.95% RPSI07_mp1599	IS5 ssgr IS1031	82493	823767	1.164
PSI07	RPSI07_mp1599	82.47% ISCARN14_aa1	97.95% RPSI07_mp0668	IS5 ssgr IS1031	1870530	1871414	885
PSI07	RPSI07_mp0250	78.94% ISRel13_aa1	100% RPSI07_mp0251	IS5 ssgr IS427	301799	302011	213
PSI07	RPSI07_mp0251	67.94% ISAli12B_aa3	No hit	IS5 ssgr IS427	30192	302426	507
PSI07	RPSI07_mp0669	98.37% IS1421_aa2	78.68% RPSI07_mp1601	IS5 ssgr IS427	825367	824996	372
PSI07	RPSI07_mp0670	100% IS1421_aa1	74.50% RPSI07_mp1600	IS5 ssgr IS427	825798	825442	357
PSI07	RPSI07_mp1601	81.86% IS1421_aa3	78.68% RPSI07_mp0669	IS5 ssgr IS427	1871729	1872277	549
PSI07	RPSI07_mp0041	62.83% IS1405_aa1	No hit	IS5 ssgr IS5	45319	45717	399
PSI07	RPSI07_mp0963	43.54% ISShvi3_aa1	42.97% RPSI07_mp1792	IS91	1160832	1159132	1.701
PSI07	RPSI07_mp1481	60.83% ISSm4_aa2	No hit	ISL3	1773347	1772925	423
PSI07	RPSI07_mp1482	50% ISSm4_aa2	No hit	ISL3	1773942	1773460	483
PSI07	RPSI07_mp0945	68.31% ISKpn21_aa1	No hit	ISNCY ssgr IS1202	1136708	1136343	366
PSI07	RPSI07_mp0401	0% newcandidate	not_found	New_Family	463818	463543	276
KACC10722	IS_d132e9d0_01360	92.85% ISRso8_aa1	No hit	IS3	1694545	1694240	306
KACC10722	IS_d132e9d0_00903	85.05% ISGau4_aa1	65.21% IS_d132e9d0_01326	IS3 ssgr IS407	1145292	1145558	267
KACC10722	IS_d132e9d0_00904	73.26% ISGau4_aa2	No hit	IS3 ssgr IS407	1145591	1146412	822
KACC10722	IS_d132e9d0_00038	63.71% IS1405_aa1	No hit	IS5 ssgr IS5	46558	46956	399
KACC10722	IS_d132e9d0_00834	87.71% ISCARN25_aa1	No hit	IS630	1055987	1056223	237
KACC10722	IS_d132e9d0_01097	72.72% ISDge1_aa1	No hit	IS701	1407121	1408032	912
KACC10722	IS_d132e9d0_00821	43.95% ISWz1_aa1	94.48% IS_d132e9d0_00787	IS91	1039279	1037579	1.701
KACC10722	IS_d132e9d0_01267	54.26% ISSm4_aa2	No hit	ISL3	1605402	1604380	1.023
KACC10722	IS_d132e9d0_00740	63.15% ISKpn21_aa1	83.33% IS_d132e9d0_00894	ISNCY ssgr IS1202	924106	923897	210
KACC10722	IS_d132e9d0_00810	72.53% ISKpn21_aa1	No hit	ISNCY ssgr IS1202	1015174	1014191	984
KACC10722	IS_d132e9d0_00894	61.97% ISKpn21_aa1	83.33% IS_d132e9d0_00740	ISNCY ssgr IS1202	1135105	1135443	339

Partial elements

Table S4b. Characterization of Insertion sequences elements found in the megaplasmid of *Ralstonia* spp. with ISFinder database

Strain	IS N°	Name	IS Family	Subgroups	IS Lenght	Position
GMI1000	2	ISRs06	IS21	-	2260	1196913-1198976
GMI1000	1	ISRs06	IS21	-	2240	562830-564893
GMI1000	1	IS1090	IS256	-	1452	598805-600131
GMI1000	1	ISRs017	IS256	-	1359	598792-600135
GMI1000	1	ISRs010	IS3	IS2	1518	577497-578831
GMI1000	2	ISRs010	IS3	IS2	1518	707871-709206
GMI1000	1	ISBph1	IS3	IS3	1383	687737-689019
GMI1000	1	ISBps1	IS3	IS3	1321	687737-689023
GMI1000	1	ISRs012	IS3	IS407	1319	625098- 626322
GMI1000	1	ISRs014	IS3	IS407	1283	1547463- 1548696
GMI1000	1	ISBcen17	IS3	IS3	1266	687737- 689018
GMI1000	3	ISRs012	IS3	IS407	1264	1682997-1683884
GMI1000	1	ISRso16	IS3	IS407	1238	699344 -700581
GMI1000	2	ISRs012	IS3	IS407	1133	1652807-1653694
GMI1000	2	ISRso16	IS3	IS407	1070	2048574- 2049331
GMI1000	4	ISRs012	IS3	IS407	444	1648630-1648974
GMI1000	1	ISRs013	IS4	IS4	1467	1448757-1450223
GMI1000	3	ISRs013	IS4	IS4	1467	1648978 -1650444
GMI1000	2	ISRs013	IS4	IS4	1465	581774-583240
GMI1000	1	ISButh4	IS5	IS5	1514	179091-180433
GMI1000	1	ISRs09	IS5	IS5	1483	179043-180525
GMI1000	2	ISRs09	IS5	IS5	1482	720092-721574
GMI1000	1	IS1021	IS5	IS5	1216	1486547-1487637
GMI1000	2	IS1021	IS5	IS5	1216	254633-255723
GMI1000	1	IS1405	IS5	IS5	1182	1430451-1431624
GMI1000	1	ISRs018	IS5	IS5	1182	1430593-1431624
GMI1000	2	ISRs01	IS5	-	917	1651927-1652810
GMI1000	3	IS1421	IS5	IS427	893	395524-396387
GMI1000	3	ISRs01	IS5	-	884	147339-148222
GMI1000	1	ISRs01	IS5	-	883	1682117-1683000
GMI1000	4	IS1421	IS5	IS427	871	101428-102291
GMI1000	5	IS1421	IS5	IS427	871	45837- 46700
GMI1000	2	IS1421	IS5	IS427	870	1245105-1245968
GMI1000	1	IS1421	IS5	IS427	864	716953- 717816
GMI1000	1	ISRso5	IS630	-	1167	269710-270868
GMI1000	2	ISRso5	IS630	-	1167	1451416- 1452574
GMI1000	3	ISRso5	IS630	-	1165	2087317-2088475
GMI1000	4	ISRso5	IS630	-	740	564894-565526
GMI1000	5	ISRso5	IS630	-	640	562300- 562831
GMI1000	3	ISRs017	IS701	-	1473	1680642-1682112
GMI1000	1	ISRs017	IS701	-	1471	145864-147334
GMI1000	2	ISRs017	IS701	-	1471	1710832-1712302
GMI1000	4	ISRs017	IS701	-	1471	1650452-1651922
GMI1000	5	ISRs017	IS701	-	1471	1428976-1430446
GMI1000	6	ISRs017	IS701	-	1471	589849-591319
GMI1000	1	ISRs015	ISL3	-	1319	1947869-1949172
GMI1000	1	ISBma3	ISL3	-	1274	1947869- 1949091
P082	1	ISRs019	IS21	-	1956	463445-465400
P082	2	ISRs019	IS21	-	1956	1056326 -1058281
P082	3	ISRs019	IS21	-	1956	623411-625366
P082	4	ISRs019	IS21	-	1956	1634384- 1636339
P082	5	ISRs019	IS21	-	1956	69780-71735
P082	1	ISRs010	IS3	IS2	1335	877255-878590
P082	2	ISRs010	IS3	IS2	1334	646190-647525
UY031	1	ISRso7	IS256	-	1344	115072-116417
UY031	1	IS1090	IS256	-	1343	115085-116413
UY031	1	ISRso20	IS3	IS3	1287	730025-731311
UY031	2	ISRso20	IS3	IS3	1287	361453-362574
UY031	1	ISBxe2	IS3	IS3	1287	730025-731295
UY031	2	ISBxe2	IS3	IS3	1287	361528-362574
UY031	1	IS1021	IS5	IS5	1209	1894996-1896204

UY031	2	IS1021	IS5	IS5	1209	108646-109854
UY031	3	IS1021	IS5	IS5	1209	123450-124658
UY031	4	IS1021	IS5	IS5	1209	153946-155154
UY031	5	IS1021	IS5	IS5	1209	1819972-1821180
UY031	6	IS1021	IS5	IS5	1209	195363-196571
UY031	7	IS1021	IS5	IS5	1209	352941-354149
UY031	8	IS1021	IS5	IS5	1209	383449-384657
UY031	9	IS1021	IS5	IS5	1209	522458-523666
UY031	10	IS1021	IS5	IS5	1209	533352-534560
UY031	11	IS1021	IS5	IS5	1209	1388930-1390138
UY031	12	IS1021	IS5	IS5	1209	1361724-1362932
UY031	13	IS1021	IS5	IS5	1209	644675-645883
UY031	14	IS1021	IS5	IS5	1209	646212-647420
UY031	15	IS1021	IS5	IS5	1209	657986-659194
UY031	16	IS1021	IS5	IS5	1209	704616-705824
UY031	17	IS1021	IS5	IS5	1209	736719-737927
UY031	18	IS1021	IS5	IS5	1209	759876-761084
UY031	19	IS1021	IS5	IS5	1209	1153593-1154801
UY031	20	IS1021	IS5	IS5	1209	1100417-1101625
UY031	21	IS1021	IS5	IS5	1209	931172-932380
UY031	22	IS1021	IS5	IS5	1209	957917-959125
UY031	23	IS1021	IS5	IS5	1209	928394-929602
UY031	24	IS1021	IS5	IS5	1209	747518-748726
UY031	25	IS1021	IS5	IS5	1209	1316892-1318100
UY031	26	IS1021	IS5	IS5	1209	1433126-1434334
UY031	27	IS1021	IS5	IS5	1209	525652-526860
UY031	28	IS1021	IS5	IS5	1209	524163-525371
UY031	29	IS1021	IS5	IS5	1209	1554471-1555679
UY031	30	IS1021	IS5	IS5	1209	434081-435289
UY031	31	IS1021	IS5	IS5	1209	384802-386010
UY031	32	IS1021	IS5	IS5	1209	1613931-1615139
UY031	33	IS1021	IS5	IS5	1209	360244-361452
UY031	34	IS1021	IS5	IS5	1209	1652061-1653269
UY031	35	IS1021	IS5	IS5	1209	1658812-1660020
UY031	36	IS1021	IS5	IS5	1209	1670919-1672127
UY031	37	IS1021	IS5	IS5	1209	1710161-1711369
UY031	38	IS1021	IS5	IS5	1209	265069-266277
UY031	39	IS1021	IS5	IS5	1209	1756019-1757227
UY031	40	IS1021	IS5	IS5	1209	218538-219746
UY031	41	IS1021	IS5	IS5	1209	177523-178731
UY031	42	IS1021	IS5	IS5	1209	1864280-1865488
UY031	43	IS1021	IS5	IS5	1209	1910036-1911244
UY031	44	IS1021	IS5	IS5	1209	1911249-1912457
UY031	45	IS1021	IS5	IS5	1209	1931037-1932245
UY031	46	IS1021	IS5	IS5	1209	1940558-1941766
UY031	47	IS1021	IS5	IS5	1209	1649135-1650343
UW163	1	ISRso19	IS21	-	1956	1536973-1538928
UW163	2	ISRso19	IS21	-	1956	509621-511576
UW163	3	ISRso19	IS21	-	1956	1130930-1132885
UW163	4	ISRso19	IS21	-	1956	383969-385924
UW163	5	ISRso19	IS21	-	1956	1-1443
UW163	6	ISRso19	IS21	-	1956	1931489-1932001
UW163	1	ISRso10	IS3	IS2	1335	1870763-1872098
UW163	2	ISRso10	IS3	IS2	1335	1309750-1311085
UW163	3	ISRso10	IS3	IS2	1335	532401-533736
IBSBF1503	1	ISRso19	IS21	-	1956	1408482-1410437
IBSBF1503	2	ISRso19	IS21	-	1956	1935156-1937111
IBSBF1503	3	ISRso19	IS21	-	1956	710057-712012
IBSBF1503	5	ISRso19	IS21	-	1956	1361855-1363214
IBSBF1503	1	IS401	IS3	IS51	1316	1351354-1352672
IBSBF1503	4	ISRso19	IS21	-	1013	1517736-1519095
RS488	1	IS1021	IS5	IS5	1217	1894998-1896206
RS488	2	IS1021	IS5	IS5	1217	108646-109854
RS488	3	IS1021	IS5	IS5	1217	123450-124658

RS488	4	IS1021	IS5	IS5	1217	153946-155154
RS488	5	IS1021	IS5	IS5	1217	1819974-1821182
RS488	6	IS1021	IS5	IS5	1217	195354-196562
RS488	7	IS1021	IS5	IS5	1217	352932-354140
RS488	8	IS1021	IS5	IS5	1217	383440-384648
RS488	9	IS1021	IS5	IS5	1217	522449-523657
RS488	10	IS1021	IS5	IS5	1217	533343-534551
RS488	11	IS1021	IS5	IS5	1217	1388923-1390131
RS488	12	IS1021	IS5	IS5	1217	1361717-1362925
RS488	13	IS1021	IS5	IS5	1217	644666-645874
RS488	14	IS1021	IS5	IS5	1217	646203-647411
RS488	15	IS1021	IS5	IS5	1217	657977-659185
RS488	16	IS1021	IS5	IS5	1217	704608-705816
RS488	17	IS1021	IS5	IS5	1217	736711-737919
RS488	18	IS1021	IS5	IS5	1217	759869-761077
RS488	19	IS1021	IS5	IS5	1217	1153586-1154794
RS488	20	IS1021	IS5	IS5	1217	1100410-1101618
RS488	21	IS1021	IS5	IS5	1217	931165-932373
RS488	22	IS1021	IS5	IS5	1217	957910-959118
RS488	23	IS1021	IS5	IS5	1217	928387-929595
RS488	24	IS1021	IS5	IS5	1217	747511-748719
RS488	26	IS1021	IS5	IS5	1217	1433119-1434327
RS488	27	IS1021	IS5	IS5	1217	525643-526851
RS488	28	IS1021	IS5	IS5	1217	524154-525362
RS488	29	IS1021	IS5	IS5	1217	1554473-1555681
RS488	30	IS1021	IS5	IS5	1217	434072-435280
RS488	31	IS1021	IS5	IS5	1217	384793-386001
RS488	32	IS1021	IS5	IS5	1217	1613933-1615141
RS488	33	IS1021	IS5	IS5	1217	360235-361443
RS488	34	IS1021	IS5	IS5	1217	1652063-1653271
RS488	35	IS1021	IS5	IS5	1217	1658814-1660022
RS488	36	IS1021	IS5	IS5	1217	1670921-1672129
RS488	37	IS1021	IS5	IS5	1217	1710163-1711371
RS488	38	IS1021	IS5	IS5	1217	265060-266268
RS488	39	IS1021	IS5	IS5	1217	1756021-1757229
RS488	40	IS1021	IS5	IS5	1217	218529-219737
RS488	41	IS1021	IS5	IS5	1217	177514-178722
RS488	42	IS1021	IS5	IS5	1217	1864282-1865490
RS488	43	IS1021	IS5	IS5	1217	1910038-1911246
RS488	44	IS1021	IS5	IS5	1217	1911251-1912459
RS488	45	IS1021	IS5	IS5	1217	1931039-1932247
RS488	46	IS1021	IS5	IS5	1217	1940560-1941768
RS488	47	IS1021	IS5	IS5	1217	1649137-1650345
RS488	1	IS1421	IS5	IS427	862	648502-649322
RS488	2	IS1421	IS5	IS427	862	1930477-1930760
RS488	4	IS1421	IS5	IS427	862	1153057-1153340
RS488	5	IS1421	IS5	IS427	862	1391230-1391513
RS488	7	IS1421	IS5	IS427	862	254238-254521
RS488	8	IS1421	IS5	IS427	862	1956642-1956986
RS488	3	IS1421	IS5	IS427	829	1863720-1864003
RS488	6	IS1421	IS5	IS427	829	327388-327671
RS488	25	IS1021	IS5	IS5	315	1316885-1316944
UW551	1	ISRso20	IS3	-	1287	911007-912293
UW551	2	ISRso20	IS3		1287	514284-515405
UW551	1	IS1021	IS5	-	1209	1999705-2000913
UW551	2	IS1021	IS5	-	1209	64579-65787
UW551	3	IS1021	IS5	-	1209	84365-85573
UW551	4	IS1021	IS5	-	1209	93886-95094
UW551	5	IS1021	IS5	-	1209	261487-262695
UW551	6	IS1021	IS5	-	1209	276291-277499
UW551	7	IS1021	IS5	-	1209	306786-307994
UW551	8	IS1021	IS5	-	1209	348195-349403
UW551	9	IS1021	IS5	-	1209	1568678-1569886

UW551	10	IS1021	IS5	-	1209	505772-506980
UW551	11	IS1021	IS5	-	1209	536279-537487
UW551	12	IS1021	IS5	-	1209	1334561-1335769
UW551	13	IS1021	IS5	-	1209	675287-676495
UW551	14	IS1021	IS5	-	1209	714061-715269
UW551	15	IS1021	IS5	-	1209	1281388-1282596
UW551	16	IS1021	IS5	-	1209	825658-826866
UW551	17	IS1021	IS5	-	1209	827195-828403
UW551	18	IS1021	IS5	-	1209	838969-840177
UW551	19	IS1021	IS5	-	1209	885599-886807
UW551	20	IS1021	IS5	-	1209	1109368-1110576
UW551	21	IS1021	IS5	-	1209	917700-918908
UW551	22	IS1021	IS5	-	1209	940857-942065
UW551	23	IS1021	IS5	-	1209	928499-929707
UW551	24	IS1021	IS5	-	1209	1112146-1113354
UW551	25	IS1021	IS5	-	1209	1138891-1140099
UW551	26	IS1021	IS5	-	1209	706361-707569
UW551	27	IS1021	IS5	-	1209	676992-678200
UW551	28	IS1021	IS5	-	1209	586911-588119
UW551	29	IS1021	IS5	-	1209	537632-538840
UW551	30	IS1021	IS5	-	1209	513075-514283
UW551	31	IS1021	IS5	-	1209	1497856-1499064
UW551	32	IS1021	IS5	-	1209	417901-419109
UW551	33	IS1021	IS5	-	1209	1612874-1614082
UW551	34	IS1021	IS5	-	1209	371370-372578
UW551	35	IS1021	IS5	-	1209	330355-331563
UW551	36	IS1021	IS5	-	1209	1734215-1735423
UW551	37	IS1021	IS5	-	1209	1793671-1794879
UW551	38	IS1021	IS5	-	1209	1835702-1836910
UW551	39	IS1021	IS5	-	1209	1838549-1839757
UW551	40	IS1021	IS5	-	1209	1850656-1851864
UW551	41	IS1021	IS5	-	1209	1889897-1891105
UW551	42	IS1021	IS5	-	1209	1935754-1936962
UW551	43	IS1021	IS5	-	1209	20272-21480
UW551	44	IS1021	IS5	-	1209	1828873-1830081
UW551	1	ISRso7	IS256	-	1344	267913-269258
UW551	1	IS1421	IS5	-	864	829494-830314
UW551	1	IS1090	IS256	-	1343	267926-269254
UW551	1	ISBxe2	IS3	-	1287	911007-912277
UW551	2	ISBxe2	IS3	-	1287	514359-515405
RS489	1	IS1021	IS5	IS5	1217	1894998-1896206
RS489	2	IS1021	IS5	IS5	1217	108646-109854
RS489	3	IS1021	IS5	IS5	1217	123450-124658
RS489	4	IS1021	IS5	IS5	1217	153946-155154
RS489	5	IS1021	IS5	IS5	1217	1819974-1821182
RS489	6	IS1021	IS5	IS5	1217	195354-196562
RS489	7	IS1021	IS5	IS5	1217	352932-354140
RS489	8	IS1021	IS5	IS5	1217	383440-384648
RS489	9	IS1021	IS5	IS5	1217	522449-523657
RS489	10	IS1021	IS5	IS5	1217	533343-534551
RS489	11	IS1021	IS5	IS5	1217	1388923-1390131
RS489	12	IS1021	IS5	IS5	1217	1361717-1362925
RS489	13	IS1021	IS5	IS5	1217	644666-645874
RS489	14	IS1021	IS5	IS5	1217	646203-647411
RS489	15	IS1021	IS5	IS5	1217	657977-659185
RS489	16	IS1021	IS5	IS5	1217	704608-705816
RS489	17	IS1021	IS5	IS5	1217	736711-737919
RS489	18	IS1021	IS5	IS5	1217	759869-761077
RS489	19	IS1021	IS5	IS5	1217	1153586-1154794
RS489	20	IS1021	IS5	IS5	1217	1100410-1101618
RS489	21	IS1021	IS5	IS5	1217	931165-932373
RS489	22	IS1021	IS5	IS5	1217	957910-959118
RS489	23	IS1021	IS5	IS5	1217	928387-929595

RS489	24	IS1021	IS5	IS5	1217	747511-748719
RS489	25	IS1021	IS5	IS5	1217	1316885-1316944
RS489	26	IS1021	IS5	IS5	1217	1433119-1434327
RS489	27	IS1021	IS5	IS5	1217	525643-526851
RS489	28	IS1021	IS5	IS5	1217	524154-525362
RS489	29	IS1021	IS5	IS5	1217	1554473-1555681
RS489	30	IS1021	IS5	IS5	1217	434072-435280
RS489	31	IS1021	IS5	IS5	1217	384793-386001
RS489	32	IS1021	IS5	IS5	1217	1613933-1615141
RS489	33	IS1021	IS5	IS5	1217	360235-361443
RS489	34	IS1021	IS5	IS5	1217	1652063-1653271
RS489	35	IS1021	IS5	IS5	1217	1658814-1660022
RS489	36	IS1021	IS5	IS5	1217	1670921-1672129
RS489	37	IS1021	IS5	IS5	1217	1710163-1711371
RS489	38	IS1021	IS5	IS5	1217	265060-266268
RS489	39	IS1021	IS5	IS5	1217	1756021-1757229
RS489	40	IS1021	IS5	IS5	1217	218529-219737
RS489	41	IS1021	IS5	IS5	1217	177514-178722
RS489	42	IS1021	IS5	IS5	1217	1864282-1865490
RS489	43	IS1021	IS5	IS5	1217	1910038-1911246
RS489	44	IS1021	IS5	IS5	1217	1911251-1912459
RS489	45	IS1021	IS5	IS5	1217	1931039-1932247
RS489	46	IS1021	IS5	IS5	1217	1940560-1941768
RS489	47	IS1021	IS5	IS5	1217	1649137-1650345
RS489	1	IS1421	IS5	IS427	861	648502-649322
RS489	6	IS1421	IS5	IS427	861	327388-327671
RS489	2	IS1421	IS5	IS427	829	1930477-1930760
RS489	3	IS1421	IS5	IS427	829	1863720-1864003
RS489	4	IS1421	IS5	IS427	829	1153057-1153340
RS489	7	IS1421	IS5	IS427	829	254238-254521
RS489	5	IS1421	IS5	IS427	821	1391230-1391513
RS489	8	IS1421	IS5	IS427	606	1956642-1956986
OE11	1	ISRs010	IS3	IS2	1592	602089-603412
OE11	4	ISRs018	IS5	IS5	1497	205664- 206689
OE11	1	ISRs017	IS701	-	1474	857870-859340
OE11	2	ISRs017	IS701	-	1470	1740614-1742084
OE11	1	ISRso11	IS3	IS150	1460	1595422-1596870
OE11	1	IS1405	IS5	IS5	1301	205658-206831
OE11	2	IS1405	IS5	IS5	1301	610178-611351
OE11	3	IS1405	IS5	IS5	1301	637257- 638430
OE11	1	ISBma3	IS110	-	1266	635577-637032
OE11	4	IS1405	IS5	IS5	1185	445536-446709
OE11	3	ISRs018	IS5	IS5	1184	610184-611209
OE11	1	ISRs018	IS5	IS5	979	445678 -446703
OE11	2	ISRs018	IS5	IS5	979	637263-638288
OE11	1	ISRs016	IS3	IS407	889	1891844-1892601
K60	1	ISRme4	IS21	-	1638	815136-817604
K60	1	ISRs019	IS21	-	1003	578876-580661
K60	1	IS401	IS3	IS51	1355	1303841-1305159
K60	2	IS401	IS3	IS51		586398-587658
K60	3	IS401	IS3	IS51	1260	586398-587658
K60	1	ISRso20	IS3	IS3	1287	1876708-1877641
K60	1	ISRso1	IS5		884	698072 -698955
K60	1	ISBma3	IS110	-	1458	764020-765474
K60	2	ISBma3	IS110	-	1458	705244-706698
K60	3	ISBma3	IS110	-	1458	435663 -436859
K60	1	ISVei4	IS630	-	1516	79862-81021
K60	2	ISVei4	IS630	-	1516	160504-161663
K60	3	ISVei4	IS630	-	1516	546183-547342
K60	4	ISVei4	IS630	-	1516	1258488-1259647
K60	5	ISVei4	IS630	-	1516	1449035-1450194
K60	5	ISVei4	IS630	-	1516	1614466-1615625

K60	6	ISVei4	IS630	-	1516	577465-578624
K60	1	ISRso9	IS5	-	1483	779182-779941
K60	2	ISBxe2	IS3	-	1287	1876853-1877641
FJAT1458	1	ISRso21	ISL3	-	2032	1680625-1682656
FJAT1458	2	ISRso21	ISL3	-	2032	600173-602204
FJAT1458	3	ISRso21	ISL3	-	2032	1170044-1172075
FJAT1458	4	ISRso21	ISL3	-	2032	979662-981693
FJAT1458	5	ISRso21	ISL3	-	2032	653772-655803
FJAT1458	38	IS1021	IS5	IS5	1403	104941-106031
FJAT1458	39	IS1021	IS5	IS5	1403	1922610-1923700
FJAT1458	40	IS1021	IS5	IS5	1403	1817775-1818865
FJAT1458	6	IS1405	IS5	-	1174	946981-948154
FJAT1458	7	IS1405	IS5	-	1174	942306-943479
FJAT1458	8	IS1405	IS5	-	1174	177389-178562
FJAT1458	9	IS1405	IS5	-	1174	337723-338896
FJAT1458	10	IS1405	IS5	-	1174	344389-345562
FJAT1458	11	IS1405	IS5	-	1174	352657-353830
FJAT1458	12	IS1405	IS5	-	1174	1553158-1554331
FJAT1458	13	IS1405	IS5	-	1174	634898-636071
FJAT1458	14	IS1405	IS5	-	1174	1314988-1316161
FJAT1458	15	IS1405	IS5	-	1174	981771-982944
FJAT1458	16	IS1405	IS5	-	1174	1312634-1313807
FJAT1458	17	IS1405	IS5	-	1174	442572-443745
FJAT1458	18	IS1405	IS5	-	1174	1938176-1939349
FJAT1458	49	ISRso16	IS3	IS407	1091	179654-180744
FJAT1458	41	IS1021	IS5	IS5	1091	1554453-1555543
FJAT1458	42	IS1021	IS5	IS5	1091	1352814-1353904
FJAT1458	43	IS1021	IS5	IS5	1091	738364-739454
FJAT1458	44	IS1021	IS5	IS5	1091	1470661-1471751
FJAT1458	45	IS1021	IS5	IS5	1091	351562-352652
FJAT1458	46	IS1021	IS5	IS5	1091	1775746-1776836
FJAT1458	47	IS1021	IS5	IS5	1091	1822380-1823470
FJAT1458	48	IS1021	IS5	IS5	1091	1879225- 1880315
FJAT1458	24	ISRso18	IS5	IS5	1026	1938182-1939207
FJAT1458	25	ISRso18	IS5	IS5	1026	442714-443739
FJAT1458	26	ISRso18	IS5	IS5	1026	1312640-1313665
FJAT1458	27	ISRso18	IS5	IS5	1026	981777-982802
FJAT1458	28	ISRso18	IS5	IS5	1026	1315130-1316155
FJAT1458	29	ISRso18	IS5	IS5	1026	634904-635929
FJAT1458	30	ISRso18	IS5	IS5	1026	1553300-1554325
FJAT1458	31	ISRso18	IS5	IS5	1026	352663-353688
FJAT1458	32	ISRso18	IS5	IS5	1026	344395-345420
FJAT1458	33	ISRso18	IS5	IS5	1026	337729-338754
FJAT1458	34	ISRso18	IS5	IS5	1026	177395-178420
FJAT1458	35	ISRso18	IS5	IS5	1026	242537-243562
FJAT1458	36	ISRso18	IS5	IS5	1026	942448-943473
FJAT1458	37	ISRso18	IS5	IS5	1026	946987-948012
FJAT1458	23	ISRso10	IS3	IS2	974	339816-340789
FJAT1458	20	ISRso1	IS5	-	884	943620-944503
FJAT1458	21	ISRso1	IS5	-	884	195688-196571
FJAT1458	22	ISRso1	IS5	-	884	573787-574662
FJAT1458	19	IS1421	IS5	IS427	864	946113-946976
FJAT1458	50	IS1021	IS5	IS5	657	1664534-1665190
EP1	11	ISRso1	IS5	-	1768	1123904-1124787
EP1	9	ISRso1	IS5	-	1752	1014787-1015662
EP1	10	ISRso1	IS5	-	1752	1315238-1316121
EP1	18	ISBma3	IS110	-	1456	876235-877690
EP1	19	ISRso16	IS3	IS407	1455	901271-902725
EP1	2	ISRso11	IS3	IS150	1449	1881601-1883049
EP1	1	ISRso17	IS701	-	1417	1124792-1126262
EP1	13	ISRso18	IS5	IS5	1312	1713081-1714106
EP1	3	IS1405	IS5	IS5	1174	364771-365944

EP1	4	IS1405	IS5	IS5	1174	847258-848431
EP1	5	IS1405	IS5	IS5	1174	902950-904123
EP1	6	IS1405	IS5	IS5	1174	1011740-1012913
EP1	7	IS1405	IS5	IS5	1174	1713075-1714248
EP1	8	ISRso10	IS3	IS2	1174	839169-840492
EP1	14	ISRso18	IS5	IS5	1026	1011882-1012907
EP1	15	ISRso18	IS5	IS5	1026	902956-903981
EP1	16	ISRso18	IS5	IS5	1026	847264-848289
EP1	17	ISRso18	IS5	IS5	1026	364777-365802
EP1	20	ISRso16	IS3	IS407	758	114306-115063
EP1	12	ISRso1	IS5	-	260	1598300-1598559
CQPS-1	1	ISRso17	IS701	-	1332	207707-209177
CQPS-1	2	ISRso17	IS701	-	1332	1763297-1764767
CQPS-1	3	ISRso17	IS701	-	1332	298726-300196
CQPS-1	4	ISRso17	IS701	-	1331	1061339-1062808
CQPS-1	26	IS1021	IS5	IS5	987	1789901-1790991
CQPS-1	7	IS1405	IS5	IS5	966	31743-32916
CQPS-1	8	IS1405	IS5	IS5	966	823627-824800
CQPS-1	9	IS1405	IS5	IS5	966	396892-398065
CQPS-1	10	IS1405	IS5	IS5	966	80592-81765
CQPS-1	11	IS1405	IS5	IS5	966	2019577-2020749
CQPS-1	12	IS1405	IS5	IS5	966	13385-14557
CQPS-1	20	ISRso18	IS5	IS5	966	13526-14551
CQPS-1	21	ISRso18	IS5	IS5	966	80734-81759
CQPS-1	22	ISRso18	IS5	IS5	966	397034-398059
CQPS-1	23	ISRso18	IS5	IS5	966	823633-824658
CQPS-1	24	ISRso18	IS5	IS5	966	2019718-2020743
CQPS-1	25	ISRso18	IS5	IS5	966	31749-32774
CQPS-1	13	IS1420	IS5	IS903	957	2039296-2040426
CQPS-1	19	ISButh1	IS3	IS2	837	1330317-1331646
CQPS-1	5	ISRso11	IS3	IS150	828	249117-250564
CQPS-1	15	ISRso1	IS5	-	825	1327319-1328202
CQPS-1	16	ISRso1	IS5	-	825	1590545-1591428
CQPS-1	17	ISRso1	IS5	-	825	297838-298721
CQPS-1	18	ISRso1	IS5	-	825	1764772-1765655
CQPS-1	14	ISRso10	IS3	IS2	786	2027630-2028952
CQPS-1	6	ISRso12	IS3	IS407	705	1388599-1389816
CQPS-1	27	ISRso16	IS3	IS407	675	549658-550415
FJAT91	1	IS1405	IS5	IS5	1174	1275730-1276903
FJAT91	2	IS1405	IS5	IS5	1174	583902-585075
FJAT91	3	IS1405	IS5	IS5	1174	439949-441122
FJAT91	4	IS1405	IS5	IS5	1174	1755195-1756368
FJAT91	5	IS1405	IS5	IS5	1174	1833851-1835024
FJAT91	6	IS1405	IS5	IS5	1174	348193-349366
FJAT91	7	IS1405	IS5	IS5	1174	686247-687420
FJAT91	8	IS1421	IS5	IS427	1174	1780674-1781537
FJAT91	28	IS1021	IS5	IS5	987	746657-747746
FJAT91	29	IS1021	IS5	IS5	987	32721-33810
FJAT91	30	IS1021	IS5	IS5	987	1042760-1043850
FJAT91	31	IS1021	IS5	IS5	987	27369-28459
FJAT91	32	IS1021	IS5	IS5	987	1782657-1783747
FJAT91	33	IS1021	IS5	IS5	987	422570-423660
FJAT91	35	IS1021	IS5	IS5	987	792875-793965
FJAT91	36	IS1021	IS5	IS5	987	1057139-1058229
FJAT91	37	IS1021	IS5	IS5	987	745445-746535
FJAT91	38	IS1021	IS5	IS5	987	1555126-1556216
FJAT91	40	IS1021	IS5	IS5	987	1779417-1780507
FJAT91	41	IS1021	IS5	IS5	987	99918-101008
FJAT91	14	ISRso18	IS5	IS5	966	54160-55185
FJAT91	16	ISRso18	IS5	IS5	966	1058376-1059401
FJAT91	17	ISRso18	IS5	IS5	966	1137970-1138995
FJAT91	20	ISRso18	IS5	IS5	966	686253-687278

FJAT91	22	ISRso18	IS5	IS5	966	1833993-1835018
FJAT91	24	ISRso18	IS5	IS5	966	440091-441116
FJAT91	25	ISRso18	IS5	IS5	966	584044-585069
FJAT91	19	ISRso18	IS5	IS5	947	732653-733678
FJAT91	26	ISRso18	IS5	IS5	930	1275872-1276897
FJAT91	15	ISRso18	IS5	IS5	927	903131-904156
FJAT91	39	IS1021	IS5	IS5	923	417706-418796
FJAT91	18	ISRso18	IS5	IS5	918	1171340-1172365
FJAT91	34	IS1021	IS5	IS5	918	1274594-1275684
FJAT91	21	ISRso18	IS5	IS5	897	348199-349224
FJAT91	13	ISRso1	IS5	-	884	1894935-1895810
FJAT91	9	IS1421	IS5	IS427	864	24006 -24869
FJAT91	10	IS1421	IS5	IS427	864	882670 -883533
FJAT91	11	IS1421	IS5	IS427	864	1045329-1046192
FJAT91	12	ISRso1	IS5	-	825	1784359-1785242
FJAT91	27	ISRso10	IS3	IS2	822	37283-38230
FJAT91	42	ISRso16	IS3	IS407	590	676645-677301
FJAT91	23	ISRso18	IS5	IS5	526	1755201-1756226
FQY_4	1	ISBma3	IS110	-	1458	2063467-2064923
FQY_4	2	ISBma3	IS110	-	1458	681028-682482
FQY_4	1	ISBcen18	IS256	-	1372	111280-112645
FQY_4	1	ISRso10	IS3	IS2	1335	459785-460761
FQY_4	1	ISRso16	IS3	IS407	1238	2042073-2042830
FQY_4	1	IS1021	IS5	IS5	1209	862349-863439
FQY_4	2	IS1021	IS5	IS5	1209	1678435-1679524
FQY_4	3	IS1021	IS5	IS5	1209	1927963-1929052
FQY_4	4	IS1021	IS5	IS5	1209	108048-109138
FQY_4	5	IS1021	IS5	IS5	1209	1946032-1947122
FQY_4	6	IS1021	IS5	IS5	1209	1688543-1689633
FQY_4	7	IS1021	IS5	IS5	1209	1256406-1257496
FQY_4	8	IS1021	IS5	IS5	1209	910947-912037
FQY_4	9	IS1021	IS5	IS5	1209	479636-480726
FQY_4	10	IS1021	IS5	IS5	1209	1687038-1688128
FQY_4	11	IS1021	IS5	IS5	1209	1840162-1841252
FQY_4	1	ISRso18	IS5	IS5	1188	1727462-1728487
FQY_4	2	ISRso18	IS5	IS5	1188	1452369-1453394
FQY_4	3	ISRso18	IS5	IS5	1188	62381-63406
FQY_4	4	ISRso18	IS5	IS5	1188	700092-701117
FQY_4	1	IS1405	IS5	IS5	1174	700086-701259
FQY_4	2	IS1405	IS5	IS5	1174	62375-63548
FQY_4	3	IS1405	IS5	IS5	1174	1452363-1453536
FQY_4	4	IS1405	IS5	IS5	1174	1727456-1728629
FQY_4	5	IS1405	IS5	IS5	1174	702432-702949
FQY_4	1	ISRso1	IS5	-	884	1259754-1260637
FQY_4	1	IS1421	IS5	IS427	864	1254604-1255467
FQY_4	2	IS1421	IS5	IS427	864	51725-52588
FQY_4	3	IS1421	IS5	IS427	864	109200-110063
FQY_4	4	IS1421	IS5	IS427	864	844545-845408
FQY_4	5	IS1421	IS5	IS427	864	493358-494221
FQY_4	6	IS1421	IS5	IS427	864	462428-463289
FQY_4	7	IS1421	IS5	IS427	864	1464200-1464714
FQY_4	8	IS1421	IS5	IS427	864	341642-342151
RSCM	1	TnAs2	Tn3	-	2992	1371664 -1374134
RSCM	1	TnAs3	Tn3	-	2992	1371664- 1374132
RSCM	1	ISPa38	Tn3	-	2976	1371664-1374134
RSCM	16	TnAs2	Tn3	-	2964	1371664-1374134
RSCM	17	TnAs3	Tn3	-	2964	1371664-1374132
RSCM	15	TnShfr1	Tn3	-	2931	1371716-1374646
RSCM	14	ISPa38	Tn3	-	2468	1371664-1374134
RSCM	18	ISShes11	Tn3	-	2466	1371664-1374132
RSCM	1	ISRso17	IS701	-	1470	2142572-2144042
RSCM	2	ISRso17	IS701	-	1470	111961-113431

RSCM	3	ISRso17	IS701	-	1470	276351-277821
RSCM	4	ISRso17	IS701	-	1470	1528522-1529992
RSCM	5	ISRso17	IS701	-	1470	1846246-1847716
RSCM	6	ISRso17	IS701	-	1470	1900134 -1901604
RSCM	7	ISRso17	IS701	-	1470	94400-95870
RSCM	8	ISRso17	IS701	-	1470	1559719-1561189
RSCM	9	ISRso17	IS701	-	1470	854858-855962
RSCM	10	ISRso17	IS701	-	1470	1778792-1779895
RSCM	11	ISRso17	IS701	-	1470	1430677-1431566
RSCM	1	ISRso11	IS3	IS150	1374	2092449-2093897
RSCM	13	ISRso11	IS3	IS150	1368	2092449-2093897
RSCM	1	ISRso17	IS701	-	1332	2142572-2144042
RSCM	2	ISRso17	IS701	-	1332	111961-113431
RSCM	3	ISRso17	IS701	-	1332	276351-277821
RSCM	4	ISRso17	IS701	-	1332	1528522-1529992
RSCM	5	ISRso17	IS701	-	1332	1846246-1847716
RSCM	6	ISRso17	IS701	-	1332	1900134-1901604
RSCM	7	ISRso17	IS701	-	1332	94400-95870
RSCM	8	ISRso17	IS701	-	1332	1559719-1561189
RSCM	40	ISRso7	IS256	-	1251	1595391-1596729
RSCM	1	IS401	IS3	IS51	1239	1382017-1383335
RSCM	35	IS401	IS3	IS51	1236	1382017-1383335
RSCM	36	IS401	IS3	IS51	1236	2090184-2091502
RSCM	2	IS401	IS3	IS51	1235	2090184-2091502
RSCM	37	ISRso10	IS3	IS2	1200	845804-847127
RSCM	9	ISRso17	IS701	-	1026	854858-855962
RSCM	10	ISRso17	IS701	-	1026	1778792-1779895
RSCM	1	IS1405	IS5	IS5	967	110783- 111956
RSCM	2	IS1405	IS5	IS5	967	1958841-1960014
RSCM	3	IS1405	IS5	IS5	967	275173- 276346
RSCM	4	IS1405	IS5	IS5	967	1869909-1871082
RSCM	5	IS1405	IS5	IS5	967	1587280-1588453
RSCM	6	IS1405	IS5	IS5	967	780761-781934
RSCM	7	IS1405	IS5	IS5	967	1384449-1385622
RSCM	8	IS1405	IS5	IS5	967	1138980 -1140153
RSCM	9	IS1405	IS5	IS5	967	847583- 848756
RSCM	10	IS1405	IS5	IS5	967	828729- 829902
RSCM	11	IS1405	IS5	IS5	967	684378-685551
RSCM	19	IS1405	IS5	IS5	966	110783-111956
RSCM	20	IS1405	IS5	IS5	966	1958841-1960014
RSCM	21	IS1405	IS5	IS5	966	275173-276346
RSCM	22	IS1405	IS5	IS5	966	1869909-1871082
RSCM	23	IS1405	IS5	IS5	966	1587280-1588453
RSCM	24	IS1405	IS5	IS5	966	780761-781934
RSCM	25	IS1405	IS5	IS5	966	1384449-1385622
RSCM	26	IS1405	IS5	IS5	966	1138980-1140153
RSCM	27	IS1405	IS5	IS5	966	847583-848756
RSCM	28	IS1405	IS5	IS5	966	828729-829902
RSCM	29	IS1405	IS5	IS5	966	684378-685551
RSCM	30	IS1405	IS5	IS5	966	1588648-1589821
RSCM	31	IS1405	IS5	IS5	966	1667669-1668842
RSCM	32	IS1405	IS5	IS5	966	517650-518823
RSCM	33	IS1405	IS5	IS5	966	458452-459625
RSCM	11	ISRso17	IS701	-	828	1430677-1431566
RSCM	41	ISRso1	IS5	-	825	2144047-2144930
RSCM	42	ISRso1	IS5	-	825	1529997-1530880
RSCM	43	ISRso1	IS5	-	825	1366891-1367774
RSCM	44	ISRso1	IS5	-	825	1181869-1182752
RSCM	45	ISRso1	IS5	-	825	1561194-1562077
RSCM	46	ISRso1	IS5	-	825	1901609-1902492
RSCM	47	ISRso1	IS5	-	825	95875-96758
RSCM	48	ISRso1	IS5	-	825	135677-136560
RSCM	38	IS1421	IS5	IS427	804	1562081-1562907
RSCM	39	IS1421	IS5	IS427	804	461532-462352

RSCM	49	ISRso1	IS5	-	525	1848218-1848758
RSCM	12	ISRso17	IS701	-	315	853281-853656
RSCM	34	IS1405	IS5	IS5	309	323530-323980
RSCM	50	ISRso1	IS5	-	303	1847721-1848067
T60	1	ISRso10	IS3	IS2	1335	614468-615444
T60	2	ISRso10	IS3	IS2	1335	685633-686608
T60	3	ISRso10	IS3	IS2	1335	616809-617167
T60	1	ISRso16	IS3	IS407	1238	2023080-2023736
T60	1	IS1021	IS5	IS5	1209	47325-48415
T60	1	ISRso18	IS5	IS5	1188	1574765-1575790
T60	2	ISRso18	IS5	IS5	1188	692601-693626
T60	3	ISRso18	IS5	IS5	1188	1341211-1342236
T60	4	ISRso18	IS5	IS5	1188	1060293-1061318
T60	5	ISRso18	IS5	IS5	1188	1141412-1142437
T60	6	ISRso18	IS5	IS5	1188	883442-884467
T60	7	ISRso18	IS5	IS5	1188	588609-589634
T60	8	ISRso18	IS5	IS5	1188	542682-543707
T60	9	ISRso18	IS5	IS5	1188	48426-49451
T60	10	ISRso18	IS5	IS5	1188	688248-689273
T60	11	ISRso18	IS5	IS5	1188	99576-100601
T60	12	ISRso18	IS5	IS5	1188	1148176-1149200
T60	13	ISRso18	IS5	IS5	1188	1864393-1865418
T60	14	ISRso18	IS5	IS5	1188	1438561-1439584
T60	1	IS1405	IS5	IS5	1174	99434-100607
T60	2	IS1405	IS5	IS5	1174	1864251-1865424
T60	3	IS1405	IS5	IS5	1174	1438555-1439726
T60	4	IS1405	IS5	IS5	1174	48420- 49593
T60	5	IS1405	IS5	IS5	1174	542676-543849
T60	6	IS1405	IS5	IS5	1174	588603-589776
T60	7	IS1405	IS5	IS5	1174	883436-884609
T60	8	IS1405	IS5	IS5	1174	1141270-1142443
T60	9	IS1405	IS5	IS5	1174	1060151-1061324
T60	10	IS1405	IS5	IS5	1174	1341205-1342378
T60	11	IS1405	IS5	IS5	1174	692459-693632
T60	12	IS1405	IS5	IS5	1174	1574759-1575932
T60	13	IS1405	IS5	IS5	1174	688106-689279
T60	14	IS1405	IS5	IS5	1174	1148170-1149342
T60	1	IS1420	IS5	IS903	1131	209225-210355
T60	2	IS1420	IS5	IS903	1131	1668335-1669465
T60	3	IS1420	IS5	IS903	1131	607088-608218
T60	4	IS1420	IS5	IS903	1131	591081-592211
T60	1	ISRso1	IS5	-	884	1417950-1418833
T60	2	ISRso1	IS5	-	884	935431-936314
T60	3	ISRso1	IS5	-	884	1247325-1248208
T60	4	ISRso1	IS5	-	884	1674656-1675539
T60	5	ISRso1	IS5	-	884	824346-825221
T60	1	IS1421	IS5	IS427	864	937924-938787
SL3882	1	ISRso10	IS3	IS2	1335	614473-615449
SL3882	2	ISRso10	IS3	IS2	1335	685639-686614
SL3882	3	ISRso10	IS3	IS2	1335	616814-617172
SL3882	1	ISRso16	IS3	IS407	1238	2034548-2035204
SL3882	1	ISRso18	IS5	IS5	1188	1586245-1587270
SL3882	2	ISRso18	IS5	IS5	1188	692607-693632
SL3882	3	ISRso18	IS5	IS5	1188	1341200-1342225
SL3882	4	ISRso18	IS5	IS5	1188	1060282-1061307
SL3882	5	ISRso18	IS5	IS5	1188	1141401-1142426
SL3882	6	ISRso18	IS5	IS5	1188	883431-884456
SL3882	7	ISRso18	IS5	IS5	1188	588614-589639
SL3882	8	ISRso18	IS5	IS5	1188	542699-543724
SL3882	9	ISRso18	IS5	IS5	1188	688254-689279
SL3882	10	ISRso18	IS5	IS5	1188	99594-100619
SL3882	11	ISRso18	IS5	IS5	1188	1450037-1451062

SL3882	12	ISRso18	IS5	IS5	1188	1148165-1149189
SL3882	13	ISRso18	IS5	IS5	1188	1875873-1876898
SL3882	1	IS1405	IS5	IS5	1174	1450031-1451204
SL3882	2	IS1405	IS5	IS5	1174	99452-100625
SL3882	3	IS1405	IS5	IS5	1174	1875731-1876904
SL3882	4	IS1405	IS5	IS5	1174	48420-49593
SL3882	5	IS1405	IS5	IS5	1174	542693-543866
SL3882	6	IS1405	IS5	IS5	1174	588608-589781
SL3882	7	IS1405	IS5	IS5	1174	883425-884598
SL3882	8	IS1405	IS5	IS5	1174	1141259-1142432
SL3882	9	IS1405	IS5	IS5	1174	1060140-1061313
SL3882	10	IS1405	IS5	IS5	1174	1341194-1342367
SL3882	11	IS1405	IS5	IS5	1174	692465-693638
SL3882	12	IS1405	IS5	IS5	1174	1586239-1587412
SL3882	13	IS1405	IS5	IS5	1174	688112-689285
SL3882	14	IS1405	IS5	IS5	1174	1148159-1149331
SL3882	1	IS1420	IS5	IS903	1131	209242-210372
SL3882	2	IS1420	IS5	IS903	1131	1679815-1680945
SL3882	3	IS1420	IS5	IS903	1131	607093-608223
SL3882	4	IS1420	IS5	IS903	1131	591086-592216
SL3882	1	ISRso1	IS5	-	884	1429414-1430297
SL3882	2	ISRso1	IS5	-	884	935420-936303
SL3882	3	ISRso1	IS5	-	884	1247314-1248197
SL3882	4	ISRso1	IS5	-	884	1686136-1687019
SL3882	5	ISRso1	IS5	-	884	824335-825210
SL3882	1	IS1421	IS5	IS427	864	937913-938776
T42	1	ISRso16	IS3	IS407	1238	1734137-1734793
T42	1	IS1021	IS5	IS5	1209	48228-49318
T42	2	IS1021	IS5	IS5	1209	299316-300406
T42	3	IS1021	IS5	IS5	1209	216685-217775
T42	4	IS1021	IS5	IS5	1209	213748-214838
T42	5	IS1021	IS5	IS5	1209	93009-94099
T42	1	ISRso18	IS5	IS5	1188	34932-35957
T42	2	ISRso18	IS5	IS5	1188	1535372-1536397
T42	3	ISRso18	IS5	IS5	1188	344526-345551
T42	4	ISRso18	IS5	IS5	1188	1284413-1285438
T42	5	ISRso18	IS5	IS5	1188	1055188-1056213
T42	6	ISRso18	IS5	IS5	1188	856266-857291
T42	7	ISRso18	IS5	IS5	1188	863030-864055
T42	8	ISRso18	IS5	IS5	1188	604371-605396
T42	9	ISRso18	IS5	IS5	1188	600018-601043
T42	10	ISRso18	IS5	IS5	1188	396362-397387
T42	11	ISRso18	IS5	IS5	1188	51048-52073
T42	12	ISRso18	IS5	IS5	1188	104115-105140
T42	13	ISRso18	IS5	IS5	1188	1148252-1149277
T42	14	ISRso18	IS5	IS5	1188	1573160-1574185
T42	1	IS1405	IS5	IS5	1174	1573018-1574191
T42	2	IS1405	IS5	IS5	1174	1148246-1149419
T42	3	IS1405	IS5	IS5	1174	103973-105146
T42	4	IS1405	IS5	IS5	1174	34790-35963
T42	5	IS1405	IS5	IS5	1174	51042-52215
T42	6	IS1405	IS5	IS5	1174	396356-397529
T42	7	IS1405	IS5	IS5	1174	600012-601185
T42	8	IS1405	IS5	IS5	1174	604365-605538
T42	9	IS1405	IS5	IS5	1174	863024-864197
T42	10	IS1405	IS5	IS5	1174	856124-857297
T42	11	IS1405	IS5	IS5	1174	1055182-1056355
T42	12	IS1405	IS5	IS5	1174	1284407-1285580
T42	13	IS1405	IS5	IS5	1174	344384-345557
T42	14	IS1405	IS5	IS5	1174	1535366-1536539
T42	1	IS1420	IS5	IS903	1131	116049-117179
T42	2	IS1420	IS5	IS903	1131	597968-599098
T42	1	ISRso1	IS5	-	884	1127629-1128512

T42	2	ISRso1	IS5	-	884	1089350-1090233
T42	3	ISRso1	IS5	-	884	654876-655759
T42	1	IS1421	IS5	IS427	864	657368-658231
T42	2	IS1421	IS5	IS427	864	1591936-1592797
T42	3	IS1421	IS5	IS427	864	102036-102897
T42	4	IS1421	IS5	IS427	864	300524-301381
SL3300	1	ISRso10	IS3	IS2	1335	651371-652344
SL3300	1	ISRso16	IS3	IS407	1238	1986790-1987446
SL3300	1	IS1021	IS5	IS5	1209	1843024-1844114
SL3300	2	IS1021	IS5	IS5	1209	197938-199028
SL3300	3	IS1021	IS5	IS5	1209	214922-216012
SL3300	4	IS1021	IS5	IS5	1209	1663222-1664312
SL3300	5	IS1021	IS5	IS5	1209	1662010-1663100
SL3300	6	IS1021	IS5	IS5	1209	634402-635492
SL3300	7	IS1021	IS5	IS5	1209	658154-659244
SL3300	8	IS1021	IS5	IS5	1209	1375539-1376629
SL3300	9	IS1021	IS5	IS5	1209	619315-620405
SL3300	10	IS1021	IS5	IS5	1209	618103-619193
SL3300	11	IS1021	IS5	IS5	1209	1664658-1665748
SL3300	12	IS1021	IS5	IS5	1209	1682236-1683326
SL3300	13	IS1021	IS5	IS5	1209	213058-214148
SL3300	14	IS1021	IS5	IS5	1209	127049-128139
SL3300	15	IS1021	IS5	IS5	1209	1665868-1666736
SL3300	1	ISRso18	IS5	IS5	1188	33489-34514
SL3300	2	ISRso18	IS5	IS5	1188	1525952-1526977
SL3300	3	ISRso18	IS5	IS5	1188	653984-655009
SL3300	4	ISRso18	IS5	IS5	1188	1291465-1292490
SL3300	5	ISRso18	IS5	IS5	1188	1099300-1100325
SL3300	6	ISRso18	IS5	IS5	1188	847232-848257
SL3300	7	ISRso18	IS5	IS5	1188	48393-49418
SL3300	8	ISRso18	IS5	IS5	1188	1092536-1093561
SL3300	9	ISRso18	IS5	IS5	1188	99471-100496
SL3300	10	ISRso18	IS5	IS5	1188	1619975-1621000
SL3300	11	ISRso18	IS5	IS5	1188	1390027-1391052
SL3300	12	ISRso18	IS5	IS5	1188	1821634-1822659
SL3300	1	IS1405	IS5	IS5	1174	1821492-1822665
SL3300	2	IS1405	IS5	IS5	1174	1390021-1391194
SL3300	3	IS1405	IS5	IS5	1174	1619969-1621142
SL3300	4	IS1405	IS5	IS5	1174	99329-100502
SL3300	5	IS1405	IS5	IS5	1174	48387-49560
SL3300	6	IS1405	IS5	IS5	1174	847226-848399
SL3300	7	IS1405	IS5	IS5	1174	1099294-1100467
SL3300	8	IS1405	IS5	IS5	1174	1291459-1292632
SL3300	9	IS1405	IS5	IS5	1174	653842-655015
SL3300	10	IS1405	IS5	IS5	1174	1525946-1527119
SL3300	11	IS1405	IS5	IS5	1174	33347-34520
SL3300	12	IS1405	IS5	IS5	1174	1092394-1093567
SL3300	1	ISRso1	IS5	-	884	1368192-1369075
SL3300	2	ISRso1	IS5	-	884	788132-789007
SL3300	1	IS1421	IS5	IS427	864	1600184-1601045
SL3300	2	IS1421	IS5	IS427	864	914531-915392
SL3300	3	IS1421	IS5	IS427	864	1844115-1844970
SL3822	1	ISRso10	IS3	IS2	1335	1483483-1484459
SL3822	2	ISRso10	IS3	IS2	1335	1348240-1349213
SL3822	3	ISRso10	IS3	IS2	1335	1364339-1364697
SL3822	4	ISRso10	IS3	IS2	1335	1481760-1482118
SL3822	1	ISRso16	IS3	IS407	1238	2087833-2088489
SL3822	1	IS1021	IS5	IS5	1209	61489-62579
SL3822	2	IS1021	IS5	IS5	1209	90386-91476
SL3822	3	IS1021	IS5	IS5	1209	602736-603826
SL3822	4	IS1021	IS5	IS5	1209	1523433-1524523
SL3822	5	IS1021	IS5	IS5	1209	668958-670048

SL3822	6	IS1021	IS5	IS5	1209	1525527-1526617
SL3822	1	ISRso18	IS5	IS5	1188	179721-180746
SL3822	2	ISRso18	IS5	IS5	1188	465524-466549
SL3822	3	ISRso18	IS5	IS5	1188	702666-703691
SL3822	4	ISRso18	IS5	IS5	1188	1345575-1346600
SL3822	5	ISRso18	IS5	IS5	1188	894824-895849
SL3822	6	ISRso18	IS5	IS5	1188	1153474-1154499
SL3822	7	ISRso18	IS5	IS5	1188	901588-902613
SL3822	8	ISRso18	IS5	IS5	1188	1507005-1508030
SL3822	9	ISRso18	IS5	IS5	1188	565488-566513
SL3822	10	ISRso18	IS5	IS5	1188	195489-196514
SL3822	11	ISRso18	IS5	IS5	1188	47215-48240
SL3822	12	ISRso18	IS5	IS5	1188	102188-103213
SL3822	13	ISRso18	IS5	IS5	1188	604090-605115
SL3822	14	ISRso18	IS5	IS5	1188	1927722-1928747
SL3822	1	IS1405	IS5	IS5	1174	603948-605121
SL3822	2	IS1405	IS5	IS5	1174	102046-103219
SL3822	3	IS1405	IS5	IS5	1174	1927580-1928753
SL3822	4	IS1405	IS5	IS5	1174	47209-48382
SL3822	5	IS1405	IS5	IS5	1174	195483-196656
SL3822	6	IS1405	IS5	IS5	1174	565482-566655
SL3822	7	IS1405	IS5	IS5	1174	1506863-1508036
SL3822	8	IS1405	IS5	IS5	1174	901582-902755
SL3822	9	IS1405	IS5	IS5	1174	1153332-1154505
SL3822	10	IS1405	IS5	IS5	1174	894682-895855
SL3822	11	IS1405	IS5	IS5	1174	1345569-1346742
SL3822	12	IS1405	IS5	IS5	1174	702524-703697
SL3822	13	IS1405	IS5	IS5	1174	465382-466555
SL3822	14	IS1405	IS5	IS5	1174	179579-180752
SL3822	1	IS1420	IS5	IS903	1131	367695-368825
SL3822	1	ISRso1	IS5	-	884	624855-625738
SL3822	2	ISRso1	IS5	-	884	1103111-1103994
SL3822	3	ISRso1	IS5	-	884	1212734-1213609
SL3822	4	ISRso1	IS5	-	884	365784-366667
SL3822	1	IS1421	IS5	IS427	864	1366253-1367116
SL3822	2	IS1421	IS5	IS427	864	1100638-1101501
HA4-1	1	ISSod9	Tn3	-	-	29736-32742
HA4-1	1	TnShfr1	Tn3	-	3705	29810-32734
HA4-1	1	TnAs3	Tn3	-	3672	29749-32216
HA4-1	1	ISRme9	IS21	-	1632	25528-28212
HA4-1	1	ISRme9	IS21	-	1623	139045-141515
HA4-1	1	ISRso11	IS3	IS150	1480	1840036-1841484
HA4-1	1	ISBma3	IS110	-	1263	559734 -561189
HA4-1	2	ISBma3	IS110	-	1262	50848-52304
HA4-1	1	ISRso10	IS3	IS2	1252	466406-467380
HA4-1	1	IS1021	IS5	IS5	1216	1784448- 1785538
HA4-1	2	IS1021	IS5	IS5	1216	1439701-1440791
HA4-1	3	IS1021	IS5	IS5	1216	1055780-1056870
HA4-1	4	IS1021	IS5	IS5	1216	897004-898094
HA4-1	5	IS1021	IS5	IS5	1216	898216-899306
HA4-1	6	IS1021	IS5	IS5	1216	899428- 900518
HA4-1	7	IS1021	IS5	IS5	1216	880753-881843
HA4-1	1	IS1021	IS5	IS5	1215	29638-30727
HA4-1	1	ISPa38	Tn3	-	1214	29749-32214
HA4-1	2	IS1420	IS5	IS903	1132	1844042 -1845172
HA4-1	1	IS1420	IS5	IS903	1116	1346905-1348035
HA4-1	1	ISRs016	IS3	IS407	1081	1132813-1133569
HA4-1	6	IS1421	IS5	IS427	878	1399494-1400357
HA4-1	2	IS1421	IS5	IS427	872	1465530-1466393
HA4-1	3	IS1421	IS5	IS427	872	1047438-1048301
HA4-1	4	IS1421	IS5	IS427	871	1202438-1203297
HA4-1	1	IS1421	IS5	IS427	870	622816-623679
HA4-1	5	IS1421	IS5	IS427	867	221750- 222613

HA4-1	7	IS1421	IS5	IS427	860	1197716- 1198575
HA4-1	2	ISRso1	IS5	-	847	1201152-1202035
HA4-1	3	ISRso1	IS5	-	847	1544249-1545132
HA4-1	1	ISRso1	IS5	-	844	483578-484461
KACC10709	1	ISRso11	IS3	IS150	1450	1935531 -1936979
KACC10709	2	ISRso11	IS3	IS150	1450	1016585-1018141
KACC10709	75	ISRso10	IS3	IS2	1335	1547076-1548049
KACC10709	76	ISRso10	IS3	IS2	1335	432506-432819
KACC10709	77	ISRso10	IS3	IS2	1335	432351-433029
KACC10709	104	ISRso16	IS3	IS407	1238	982879-983460
KACC10709	97	IS1021	IS5	IS5	1209	1892163-1893252
KACC10709	98	IS1021	IS5	IS5	1209	681312-682401
KACC10709	99	IS1021	IS5	IS5	1209	1168099-1169188
KACC10709	100	IS1021	IS5	IS5	1209	1022941-1024030
KACC10709	101	IS1021	IS5	IS5	1209	1127775-1128864
KACC10709	102	IS1021	IS5	IS5	1209	879658-880747
KACC10709	103	IS1021	IS5	IS5	1209	1526293-1527382
KACC10709	78	ISRso18	IS5	IS5	1188	175833-176858
KACC10709	79	ISRso18	IS5	IS5	1188	1868067-1869092
KACC10709	80	ISRso18	IS5	IS5	1188	1844551-1845576
KACC10709	81	ISRso18	IS5	IS5	1188	430020-431045
KACC10709	82	ISRso18	IS5	IS5	1188	572491-573516
KACC10709	83	ISRso18	IS5	IS5	1188	1549398-1550423
KACC10709	84	ISRso18	IS5	IS5	1188	1281043-1282068
KACC10709	85	ISRso18	IS5	IS5	1188	1166430-1167455
KACC10709	86	ISRso18	IS5	IS5	1188	1379026-1380051
KACC10709	87	ISRso18	IS5	IS5	1188	1561283-1562308
KACC10709	88	ISRso18	IS5	IS5	1188	1620747-1621772
KACC10709	89	ISRso18	IS5	IS5	1188	427530-428555
KACC10709	90	ISRso18	IS5	IS5	1188	388813-389838
KACC10709	91	ISRso18	IS5	IS5	1188	721283-722308
KACC10709	92	ISRso18	IS5	IS5	1188	875979-877004
KACC10709	93	ISRso18	IS5	IS5	1188	683045-684070
KACC10709	94	ISRso18	IS5	IS5	1188	718793-719818
KACC10709	95	ISRso18	IS5	IS5	1188	882098-882670
KACC10709	96	ISRso18	IS5	IS5	1188	880758-881214
KACC10709	34	IS1405	IS5	IS5	1174	875837-877010
KACC10709	35	IS1405	IS5	IS5	1174	1868061-1869234
KACC10709	36	IS1405	IS5	IS5	1174	388807-389980
KACC10709	37	IS1405	IS5	IS5	1174	427524-428697
KACC10709	38	IS1405	IS5	IS5	1174	1620605-1621778
KACC10709	39	IS1405	IS5	IS5	1174	1561141-1562314
KACC10709	40	IS1405	IS5	IS5	1174	1378884-1380057
KACC10709	41	IS1405	IS5	IS5	1174	1166288-1167461
KACC10709	42	IS1405	IS5	IS5	1174	1281037-1282210
KACC10709	43	IS1405	IS5	IS5	1174	1549392-1550565
KACC10709	44	IS1405	IS5	IS5	1174	572349-573522
KACC10709	45	IS1405	IS5	IS5	1174	429878-431051
KACC10709	46	IS1405	IS5	IS5	1174	1844545-1845718
KACC10709	47	IS1405	IS5	IS5	1174	175691-176864
KACC10709	48	IS1405	IS5	IS5	1174	683039-684212
KACC10709	49	IS1405	IS5	IS5	1174	721141-722314
KACC10709	50	IS1405	IS5	IS5	1174	718787-719960
KACC10709	51	IS1405	IS5	IS5	1174	882098-882812
KACC10709	52	IS1405	IS5	IS5	1174	880752-881214
KACC10709	3	IS1420	IS5	IS903	1131	2132223-2133353
KACC10709	4	IS1420	IS5	IS903	1131	1991862 -1992992
KACC10709	5	IS1420	IS5	IS903	1131	211362-212492
KACC10709	6	IS1420	IS5	IS903	1131	386772-387902
KACC10709	7	IS1420	IS5	IS903	1131	576685-577815
KACC10709	8	IS1420	IS5	IS903	1131	1562787-1563917
KACC10709	9	IS1420	IS5	IS903	1131	707302-708432

KACC10709	10	IS1420	IS5	IS903	1131	778600-779730
KACC10709	11	IS1420	IS5	IS903	1131	896165-897295
KACC10709	12	IS1420	IS5	IS903	1131	917214-918344
KACC10709	13	IS1420	IS5	IS903	1131	1199710-1200840
KACC10709	14	IS1420	IS5	IS903	1131	1052925-1054055
KACC10709	15	IS1420	IS5	IS903	1131	1085771-1086901
KACC10709	16	IS1420	IS5	IS903	1131	1051625-1052755
KACC10709	17	IS1420	IS5	IS903	1131	1134148-1135278
KACC10709	18	IS1420	IS5	IS903	1131	856504-857634
KACC10709	19	IS1420	IS5	IS903	1131	780799-781929
KACC10709	20	IS1420	IS5	IS903	1131	1624941-1626071
KACC10709	21	IS1420	IS5	IS903	1131	1673967-1675097
KACC10709	22	IS1420	IS5	IS903	1131	1683872-1685002
KACC10709	23	IS1420	IS5	IS903	1131	357319-358449
KACC10709	24	IS1420	IS5	IS903	1131	1869397-1870527
KACC10709	25	IS1420	IS5	IS903	1131	2113465-2114595
KACC10709	26	IS1420	IS5	IS903	1131	899454-900584
KACC10709	27	IS1420	IS5	IS903	1131	1347181-1348311
KACC10709	28	IS1420	IS5	IS903	1131	1524728-1525858
KACC10709	29	IS1420	IS5	IS903	1131	581526-582656
KACC10709	30	IS1420	IS5	IS903	1131	177424-178542
KACC10709	31	IS1420	IS5	IS903	1131	568861-569979
KACC10709	32	IS1420	IS5	IS903	1131	1163495-1164613
KACC10709	33	IS1420	IS5	IS903	1131	916323-917213
KACC10709	53	ISRsy1	IS1595	ISSod11	1090	314068-315153
KACC10709	53	ISRsy1	IS1595	ISSod11	1090	1617454-1618539
KACC10709	54	ISRsy1	IS1595	ISSod11	1090	123194-124278
KACC10709	55	ISRama1	IS1595	ISSod11	1090	314064-315153
KACC10709	56	ISRama1	IS1595	ISSod11	1090	1617450-1618539
KACC10709	57	ISRama1	IS1595	ISSod11	1090	123194-124282
KACC10709	66	ISRso1	IS5	-	884	2063713-2064596
KACC10709	67	ISRso1	IS5	-	884	179493-180376
KACC10709	68	ISRso1	IS5	-	884	1651297-1652180
KACC10709	69	ISRso1	IS5	-	884	601978-602861
KACC10709	70	ISRso1	IS5	-	884	881215-882098
KACC10709	71	ISRso1	IS5	-	884	1377550-1378433
KACC10709	72	ISRso1	IS5	-	884	1453462-1454345
KACC10709	73	ISRso1	IS5	-	884	571025-571908
KACC10709	74	ISRso1	IS5	-	884	288338-289213
KACC10709	58	IS1421	IS5	IS427	864	431634-432497
KACC10709	59	IS1421	IS5	IS427	864	872679-873542
KACC10709	60	IS1421	IS5	IS427	864	1162631-1163494
KACC10709	61	IS1421	IS5	IS427	864	1348585-1349448
KACC10709	62	IS1421	IS5	IS427	864	569980-570843
KACC10709	63	IS1421	IS5	IS427	864	1781626-1782489
KACC10709	64	IS1421	IS5	IS427	864	178543-179406
KACC10709	65	IS1421	IS5	IS427	864	173835-174698
CMR15	1	ISBdo1	IS1182	-	1572	391145-392525
CMR15	2	ISBdo1	IS1182	-	1572	995965-997345
CMR15	3	ISBdo1	IS1182	-	1572	1281681-1283062
CMR15	1	ISRso10	IS3	IS2	1335	1911775-1913109
CMR15	1	ISRso1	IS5	-	884	371735-372618
CMR15	2	ISRso1	IS5	-	884	1076442-1077325
CMR15	3	ISRso1	IS5	-	884	918095-918946
YC40M	2	ISBma3	IS110	-	1702	1667670-1669126
YC40M	3	ISBma3	IS110	-	1663	1793876-1795330
YC40M	1	ISBma3	IS110	-	1653	424344-425800
YC40M	2	ISRso18	IS5	IS5	1528	453460-454485
YC40M	1	ISBcen18	IS256	-	1517	287662-289027
YC40M	4	ISRso18	IS5	IS5	1499	761309-762334
YC40M	3	ISRso18	IS5	IS5	1440	1387830- 1388855
YC40M	3	IS1021	IS5	IS5	1387	559481-560570

YC40M	1	ISRso18	IS5	IS5	1366	336033-337058
YC40M	1	IS1021	IS5	IS5	1215	1906270-1907359
YC40M	2	IS1021	IS5	IS5	1215	810001-811090
YC40M	4	IS1021	IS5	IS5	1215	290302-291391
YC40M	1	IS1405	IS5	IS5	1174	1812934 -1814107
YC40M	2	IS1405	IS5	IS5	1174	231846-233019
YC40M	3	IS1405	IS5	IS5	1174	607913-609086
YC40M	4	IS1405	IS5	IS5	1174	1034716-1035889
YC40M	5	IS1405	IS5	IS5	1174	761167-762340
YC40M	1	ISRs016	IS3	IS407	1074	446437-447194
YC40M	2	IS1421	IS5	IS427	1062	1454536-1455399
YC40M	1	IS1421	IS5	IS427	1061	1604757-1605620
YC40M	3	IS1421	IS5	IS427	1061	346851-347714
YC40M	5	IS1421	IS5	IS427	1061	1260106-1260969
YC40M	4	IS1421	IS5	IS427	1051	1024900-1025757
YC40M	1	ISRs01	IS5	-	884	1254936-1255819
YC40M	6	IS1421	IS5	IS427	870	1573827-1574688
YC40M	1	ISRs010	IS3	IS2	845	1571184-1572160
SN82F48	1	TnAs2	Tn3	-	8654	1243145-1250125
SN82F48	2	ISMca5	IS256	-	-	1289169-1290554
SN82F48	3	ISMca5	IS256	-	-	1329322-1330707
SN82F48	2	ISCsp1	IS256	-	-	1289367-1290456
SN82F48	3	ISCsp1	IS256	-	-	1329520-1330609
SN82F48	2	TnAs2	Tn3	-	-	1347-3343
SN82F48	3	TnAs2	Tn3	-	-	50379-52375
SN82F48	4	TnAs2	Tn3	-	-	48897-50013
SN82F48	5	TnAs2	Tn3	-	-	1-981
SN82F48	2	TnAs3	Tn3	-	-	1345-3342
SN82F48	3	TnAs3	Tn3	-	-	50377-52374
SN82F48	4	TnAs3	Tn3	-	-	1248124-1249830
SN82F48	5	TnAs3	Tn3	-	-	48906-50002
SN82F48	6	TnAs3	Tn3	-	-	1-970
SN82F48	2	ISShes11	Tn3	-	-	1245833-1246545
SN82F48	1	TnShfr1	Tn3	-	2979	1243227-1245646
SN82F48	1	TnAs1	Tn3	-	2973	1243350-1244563
SN82F48	1	ISPa40	Tn3	-	2973	1243365-1244521
SN82F48	1	ISPa38	Tn3	-	2973	1243145-1246144
SN82F48	1	ISSod9	Tn3	-	2973	1243145-1245634
SN82F48	1	TnAs3	Tn3	-	2966	1243145-1246545
SN82F48	1	ISShes11	Tn3	-	2962	1243145-1245747
SN82F48	1	ISRme9	IS21	-	2688	1368951-1371431
SN82F48	1	ISCsp1	IS256	-	1393	1253985-1255074
SN82F48	1	ISMca5	IS256	-	1390	1253887-1255272
SN83A39	1	IS408	IS21	-	2798	14218-16367
SN83A39	1	ISBmu3	IS21	-	2697	14216-16912
SN83A39	2	ISBmu3	IS21	-	2697	2115854-2116350
SN83A39	1	ISButh3	IS256	-	1348	2450973-2452321
SN83A39	2	ISButh3	IS256	-	1348	3378132-3379270
SN83A39	3	ISButh3	IS256	-	1348	3366141-3366824
SEPPX05	5	ISRso19	IS21	-	-	40115-42070
SEPPX05	6	ISRso19	IS21	-	-	2020000-2021955
Rs-09-161	1	ISRso12	IS3	IS407	-	76376-77204
Rs-09-161	1	IS1421	IS5	IS427	-	78901-79760
Rs-09-161	1	ISButh4	IS5	IS5	1483	89148-90193
Rs-09-161	1	ISRso9	IS5	IS5	1483	89148-90145
Rs-09-161	1	ISRso17	IS701	-	1471	44161-45631
Rs-09-161	1	ISRso13	IS4	IS4	1467	74809-76275
Rs-09-161	1	IS1021	IS5	IS5	1209	50009-51099
Rs-09-161	1	ISRso18	IS5	IS5	1188	52293-53318

Rs-09-161	1	IS1405	IS5	IS5	1174	52288-53460
SL3103	5	ISRso1	IS5	-	-	1125854-1126737
SL3103	6	ISRso1	IS5	-	-	992571- 993454
SL3103	7	ISRso1	IS5	-	-	763809-764692
SL3103	8	ISRso1	IS5	-	-	760233- 761116
SL3103	9	ISRso1	IS5	-	-	648460-649343
SL3103	10	ISRso1	IS5	-	-	651400-652275
SL3103	2	ISRso18	IS5	IS5	-	277903-278928
SL3103	3	ISRso18	IS5	IS5	-	1760015-1761040
SL3103	4	ISRso18	IS5	IS5	-	448654-449679
SL3103	5	ISRso18	IS5	IS5	-	1571394-1572419
SL3103	6	ISRso18	IS5	IS5	-	1532678-1533703
SL3103	7	ISRso18	IS5	IS5	-	806911-807936
SL3103	8	ISRso18	IS5	IS5	-	503817-504842
SL3103	9	ISRso18	IS5	IS5	-	446164-447189
SL3103	10	ISRso18	IS5	IS5	-	1762505-1763530
SL3103	11	ISRso18	IS5	IS5	-	180795-181820
SL3103	12	ISRso18	IS5	IS5	-	765753-766778
SL3103	13	ISRso18	IS5	IS5	-	1219202-1220227
SL3103	2	IS1021	IS5	IS5	-	1919398-1920487
SL3103	3	IS1021	IS5	IS5	-	505014-506103
SL3103	4	IS1021	IS5	IS5	-	1380835-1381924
SL3103	5	IS1021	IS5	IS5	-	1195042-1196131
SL3103	6	IS1021	IS5	IS5	-	502717-503806
SL3103	7	IS1021	IS5	IS5	-	1723087-1724176
SL3103	8	IS1021	IS5	IS5	-	1767502-1768591
SL3103	9	IS1021	IS5	IS5	-	1980984-1982073
SL3103	2	ISRso11	IS3	IS150	1368	2116084-2117532
SL3103	1	ISRso11	IS3	IS150	1358	1149637-1151085
SL3103	1	ISRso10	IS3	IS2	1335	443842-444815
SL3103	1	ISRso16	IS3	IS407	1238	2082339-2082920
SL3103	1	IS1021	IS5	IS5	1209	29608-30697
SL3103	1	ISRso18	IS5	IS5	1188	67397-68422
SL3103	1	ISRama1	IS1595	ISSod11	988	1574627-1575716
SL3103	2	ISRama1	IS1595	ISSod11	988	817521-818610
SL3103	3	ISRama1	IS1595	ISSod11	988	624587- 625676
SL3103	2	ISRsy1	IS1595	ISSod11	987	817525-818610
SL3103	3	ISRsy1	IS1595	ISSod11	986	624587-625672
SL3103	1	ISRsy1	IS1595	ISSod11	985	1574627- 1575712
SL3103	1	IS1405	IS5	IS5	966	180789-181962
SL3103	2	IS1405	IS5	IS5	966	1762363-1763536
SL3103	3	IS1405	IS5	IS5	966	446158-447331
SL3103	4	IS1405	IS5	IS5	966	503811-504984
SL3103	5	IS1405	IS5	IS5	966	806905-808078
SL3103	6	IS1405	IS5	IS5	966	1532672-1533845
SL3103	7	IS1405	IS5	IS5	966	1571388-1572561
SL3103	8	IS1405	IS5	IS5	966	448512 -449685
SL3103	9	IS1405	IS5	IS5	966	1760009 -1761182
SL3103	2	IS1421	IS5	IS427	959	1305167-1306030
SL3103	1	IS1420	IS5	IS903	958	35981-37111
SL3103	3	IS1420	IS5	IS903	958	1982529-1983659
SL3103	4	IS1420	IS5	IS903	958	246926-248056
SL3103	5	IS1420	IS5	IS903	958	1822587-1823717
SL3103	6	IS1420	IS5	IS903	958	1724285-1725415
SL3103	7	IS1420	IS5	IS903	958	452848-453978
SL3103	8	IS1420	IS5	IS903	958	1632876-1634006
SL3103	9	IS1420	IS5	IS903	958	500998-502128
SL3103	2	IS1420	IS5	IS903	956	2015374-2016504
SL3103	1	ISRso1	IS5	-	884	276427-277310
SL3103	1	IS1421	IS5	IS427	870	521980-522843
SL3103	3	ISRso1	IS5	-	824	1306576-1307459
SL3103	4	ISRso1	IS5	-	824	1089519-1090402
SL3103	2	ISRso1	IS5	-	822	1311020-1311903

SL3103	3	IS1421	IS5	IS427	804	1387517-1388380
SL3103	4	IS1421	IS5	IS427	804	764753-765610
T117	1	ISRso16	IS3	IS407	1238	2013733-2014389
T117	1	IS1421	IS5	IS427	864	940079-940942
T117	1	ISRso18	IS5	IS5	1188	1565151-1566176
T117	2	ISRso18	IS5	IS5	1188	690441-691466
T117	3	ISRso18	IS5	IS5	1188	1337893-1338918
T117	4	ISRso18	IS5	IS5	1188	1145744-1146769
T117	5	ISRso18	IS5	IS5	1188	1138980-1140005
T117	6	ISRso18	IS5	IS5	1188	884114-885139
T117	7	ISRso18	IS5	IS5	1188	590626-591651
T117	8	ISRso18	IS5	IS5	1188	48394-49419
T117	9	ISRso18	IS5	IS5	1188	46959-47984
T117	10	ISRso18	IS5	IS5	1188	2026726-2027751
T117	11	ISRso18	IS5	IS5	1188	101264-102289
T117	12	ISRso18	IS5	IS5	1188	1428968-1429993
T117	13	ISRso18	IS5	IS5	1188	1853622-1854647
T117	1	ISRso10	IS3	IS2	1335	687826-688801
T117	1	IS1021	IS5	IS5	1209	212410-213500
T117	1	IS1405	IS5	IS5	1174	1428962-1430135
T117	2	IS1405	IS5	IS5	1174	101122-102295
T117	3	IS1405	IS5	IS5	1174	2026720-2027893
T117	4	IS1405	IS5	IS5	1174	1853480-1854653
T117	5	IS1405	IS5	IS5	1174	46953-48126
T117	6	IS1405	IS5	IS5	1174	590620-591793
T117	7	IS1405	IS5	IS5	1174	884108-885281
T117	8	IS1405	IS5	IS5	1174	1138838-1140011
T117	9	IS1405	IS5	IS5	1174	1145738-1146911
T117	10	IS1405	IS5	IS5	1174	1337887-1339060
T117	11	IS1405	IS5	IS5	1174	690299-691472
T117	12	IS1405	IS5	IS5	1174	1565145-1566318
T117	13	IS1405	IS5	IS5	1174	48388-49552
T117	1	ISRso1	IS5	IS427	884	1664475-1665358
T117	2	ISRso1	IS5	IS427	884	1408345-1409228
T117	3	ISRso1	IS5	IS427	884	93481-94364
T117	4	ISRso1	IS5	IS427	884	49553-50436
T117	5	ISRso1	IS5	IS427	884	936103-936986
T117	6	ISRso1	IS5	IS427	884	823521-824396
T117	7	ISRso1	IS5	IS427	884	698431-698521
SL2330	1	ISRso16	IS3	IS407	1238	1954659-1955415
SL2330	2	ISRso16	IS3	IS407	1238	1956494-1956939
SL2330	3	ISRso16	IS3	IS407	1238	1954434-1954618
SL2330	4	ISRso16	IS3	IS407	1238	1956980-1957164
SL2330	5	ISRso16	IS3	IS407	1238	1956941-1956979
SL2330	6	ISRso16	IS3	IS407	1238	1954619-1954657
SL2330	1	IS1405	IS5	IS5	1174	1264966-1266139
SL2330	2	IS1405	IS5	IS5	1174	835832-837005
SL2330	3	IS1405	IS5	IS5	1174	1071671-1072844
SL2330	4	IS1405	IS5	IS5	1174	1302161-1303334
SL2330	5	IS1405	IS5	IS5	1174	582296-583469
SL2330	6	IS1405	IS5	IS5	1174	1466063-1467236
SL2330	7	IS1405	IS5	IS5	1119	521791-522964
SL2330	9	IS1405	IS5	IS5	966	87763-88936
SL2330	8	IS1405	IS5	IS5	966	1847397-1848570
SL2330	1	ISRso18	IS5	IS5	966	87905-88930
SL2330	2	ISRso18	IS5	IS5	966	1847403-1848428
SL2330	3	ISRso18	IS5	IS5	966	521933-522958
SL2330	4	ISRso18	IS5	IS5	966	1466069-1467094
SL2330	5	ISRso18	IS5	IS5	966	582438-583463
SL2330	6	ISRso18	IS5	IS5	966	1302167-1303192
SL2330	7	ISRso18	IS5	IS5	966	1071677-1072702
SL2330	8	ISRso18	IS5	IS5	966	835838-836863

SL2330	9	ISRso18	IS5	IS5	966	1265108-1266133
SL2330	1	ISRsy1	IS1595	ISSod11	962	1539256-1540254
SL2330	1	ISRama1	IS1595	ISSod11	962	1539256-1540258
SL2330	1	ISRso10	IS3	IS2	932	601148-602110
SL2330	1	ISRso1	IS5	-	825	95587-96470
SL2330	2	ISRso1	IS5	-	825	750667-751542
SL2330	10	IS1421	IS5	IS427	804	666508-667371
SL3755	1	ISRso10	IS3	IS2	1335	600886-601848
SL3755	1	ISRso16	IS3	IS407	1238	1958970-1959726
SL3755	2	ISRso18	IS5	IS5	1188	87900-88925
SL3755	3	ISRso18	IS5	IS5	1188	522839-523864
SL3755	4	ISRso18	IS5	IS5	1188	1469339-1470364
SL3755	5	ISRso18	IS5	IS5	1188	1305441-1306466
SL3755	6	ISRso18	IS5	IS5	1188	1074975-1076000
SL3755	7	ISRso18	IS5	IS5	1188	837236-838261
SL3755	8	ISRso18	IS5	IS5	1188	1268371-1269396
SL3755	1	IS1405	IS5	IS5	1174	1268229-1269402
SL3755	2	IS1405	IS5	IS5	1174	837230-838403
SL3755	3	IS1405	IS5	IS5	1174	1074969-1076142
SL3755	4	IS1405	IS5	IS5	1174	1305435-1306608
SL3755	5	IS1405	IS5	IS5	1174	1469333-1470506
SL3755	6	IS1405	IS5	IS5	1174	522697-523870
SL3755	7	IS1405	IS5	IS5	1174	87758-88931
SL3755	1	ISRso1	IS5	-	884	95582-96465
SL3755	2	ISRso1	IS5	-	884	752068-752943
SL3755	1	IS1421	IS5	IS427	864	207706-208569
SL3755	2	IS1421	IS5	IS427	864	668780-669643
T25	2	IS1405	IS5	IS5	-	1465593-1466766
T25	3	IS1405	IS5	IS5	-	522420-523593
T25	4	IS1405	IS5	IS5	-	87736-88909
T25	5	IS1405	IS5	IS5	-	916576-917748
T25	6	IS1405	IS5	IS5	-	686266-687438
T25	7	IS1405	IS5	IS5	-	94185-95356
T25	2	ISRso1	IS5	-	-	1236560-1237435
T25	1	ISRso10	IS3	IS2	1335	1387631-1388593
T25	1	ISRso16	IS3	IS407	1238	1954501-1955257
T25	1	ISRso18	IS5	IS5	1188	87878-88903
T25	2	ISRso18	IS5	IS5	1188	522562-523587
T25	3	ISRso18	IS5	IS5	1188	1465599-1466624
T25	4	ISRso18	IS5	IS5	1188	686407-687432
T25	5	ISRso18	IS5	IS5	1188	916717-917742
T25	6	ISRso18	IS5	IS5	1188	723407-724432
T25	7	ISRso18	IS5	IS5	1188	94326-95350
T25	1	IS1405	IS5	IS5	1174	723401-724574
T25	1	ISRsy1	IS1595	ISSod11	1090	1538744-1539742
T25	1	ISRama1	IS1595	ISSod11	1090	1538744-1539746
T25	1	ISRso1	IS5	-	884	96736-97619
T25	1	IS1421	IS5	IS427	864	1319832-1320694
SL3730	1	ISRso1	IS5	-	884	1316225-1317108
SL3730	2	ISRso1	IS5	-	884	762914-763797
SL3730	3	ISRso1	IS5	-	884	1277947-1278830
SL3730	4	ISRso1	IS5	-	884	843500-844383
SL3730	5	ISRso1	IS5	-	884	733004-733879
SL3730	1	ISRso16	IS3	IS407	1238	1922714-1923370
SL3730	1	IS1021	IS5	IS5	1209	48225-49315
SL3730	2	IS1021	IS5	IS5	1209	299269-300359
SL3730	3	IS1021	IS5	IS5	1209	659972-661062
SL3730	4	IS1021	IS5	IS5	1209	216671-217761
SL3730	5	IS1021	IS5	IS5	1209	213734-214824
SL3730	6	IS1021	IS5	IS5	1209	93001-94091
SL3730	1	ISRso18	IS5	IS5	1188	34929-35954

SL3730	2	ISRso18	IS5	IS5	1188	1723953-1724978
SL3730	3	ISRso18	IS5	IS5	1188	344473-345498
SL3730	4	ISRso18	IS5	IS5	1188	1473006-1474031
SL3730	5	ISRso18	IS5	IS5	1188	621321-622346
SL3730	6	ISRso18	IS5	IS5	1188	1243787-1244812
SL3730	7	ISRso18	IS5	IS5	1188	1051636-1052661
SL3730	8	ISRso18	IS5	IS5	1188	1044873-1045898
SL3730	9	ISRso18	IS5	IS5	1188	792998-794023
SL3730	10	ISRso18	IS5	IS5	1188	604302-605327
SL3730	11	ISRso18	IS5	IS5	1188	599950-600975
SL3730	12	ISRso18	IS5	IS5	1188	396305-397330
SL3730	13	ISRso18	IS5	IS5	1188	51045-52070
SL3730	14	ISRso18	IS5	IS5	1188	104106-105131
SL3730	15	ISRso18	IS5	IS5	1188	1336847-1337872
SL3730	16	ISRso18	IS5	IS5	1188	1761740-1762765
SL3730	1	IS1405	IS5	IS5	1174	1761598-1762771
SL3730	2	IS1405	IS5	IS5	1174	1336841-1338014
SL3730	3	IS1405	IS5	IS5	1174	103964-105137
SL3730	4	IS1405	IS5	IS5	1174	34787-35960
SL3730	5	IS1405	IS5	IS5	1174	51039-52212
SL3730	6	IS1405	IS5	IS5	1174	396299-397472
SL3730	7	IS1405	IS5	IS5	1174	599944-601117
SL3730	8	IS1405	IS5	IS5	1174	604296-605469
SL3730	9	IS1405	IS5	IS5	1174	792992-794165
SL3730	10	IS1405	IS5	IS5	1174	1044731-1045904
SL3730	11	IS1405	IS5	IS5	1174	1051630-1052803
SL3730	12	IS1405	IS5	IS5	1174	1243781-1244954
SL3730	13	IS1405	IS5	IS5	1174	621179-622352
SL3730	14	IS1405	IS5	IS5	1174	1473000-1474173
SL3730	15	IS1405	IS5	IS5	1174	344331-345504
SL3730	16	IS1405	IS5	IS5	1174	1723947-1725120
SL3730	1	IS1420	IS5	IS903	1131	116039-117169
SL3730	2	IS1420	IS5	IS903	1131	597900-599030
SL3730	1	IS1421	IS5	IS427	864	845992-846855
SL3730	2	IS1421	IS5	IS427	864	1780516-1781377
SL3730	3	IS1421	IS5	IS427	864	617179-618040
SL3730	4	IS1421	IS5	IS427	864	300477-301334
SL3730	5	IS1421	IS5	IS427	864	102028-102888
12D	1	ISPsy42	Tn3	-	3030	1113067-1116121
12D	1	ISPsy30	Tn3	-	3030	1113067-1116101
12D	1	ISRme13	IS3	IS3	1288	507419-508706
12D	1	ISBxe2	IS3	IS3	1287	507419-508706
FC1138	1	ISRme4	IS21	-	2469	2728736-2731195
FC1138	2	ISRme4	IS21	-	2469	2298979-2301419
FC1138	3	ISRme4	IS21	-	2469	388206-390665
FC1138	4	ISRme4	IS21	-	2469	3329893-3332352
FC1138	5	ISRme4	IS21	-	2469	92126-94557
FC1138	6	ISRme4	IS21	-	2469	803656-806087
FC1138	1	ISBvi1	IS4	IS4	1455	3138326-3139780
FC1138	2	ISBvi1	IS4	IS4	1455	2548738-2550192
FC1138	3	ISBvi1	IS4	IS4	1455	3131506-3132960
FC1138	1	ISCARN35	IS4	IS4	1455	3138327-3139713
FC1138	2	ISCARN35	IS4	IS4	1455	2548739-2550125
FC1138	3	ISCARN35	IS4	IS4	1455	3131573-3132959
SL3022	1	ISRso10	IS3	IS2	1335	1938141-1938499
SL3022	1	IS1421	IS5	IS427	864	164178-165041
SL3022	2	IS1421	IS5	IS427	864	1940055-1940917
12J	1	ISRme13	IS3	IS3	1288	63625-64912
12J	1	ISBxe2	IS3	IS3	1287	63625-64912

T51	-	-	-	-	-	-
A2-HR	1	ISRso9	IS5	IS5	1483	483127-484421
A2-HR	2	ISRso9	IS5	IS5	1483	1068623-1069917
A2-HR	3	ISRso9	IS5	IS5	1483	1071875-1073169
A2-HR	4	ISRso9	IS5	IS5	1483	1408707-1410001
A2-HR	5	ISRso9	IS5	IS5	1483	58061-59355
A2-HR	1	ISButh4	IS5	IS5	1483	483125-484422
A2-HR	2	ISButh4	IS5	IS5	1483	1068622-1069919
A2-HR	3	ISButh4	IS5	IS5	1483	1071874-1073171
A2-HR	4	ISButh4	IS5	IS5	1483	1408706-1410003
A2-HR	5	ISButh4	IS5	IS5	1483	58059-59356
A2-HR	1	ISBcen18	IS256	-	1372	2611-3976
A2-HR	2	ISBcen18	IS256	-	1372	199524-200889
A2-HR	3	ISBcen18	IS256	-	1372	214923-216288
A2-HR	4	ISBcen18	IS256	-	1372	1245446-1246811
A2-HR	5	ISBcen18	IS256	-	1372	249038-250403
A2-HR	6	ISBcen18	IS256	-	1372	304422-305787
A2-HR	7	ISBcen18	IS256	-	1372	1066212-1067577
A2-HR	8	ISBcen18	IS256	-	1372	494191-495556
A2-HR	9	ISBcen18	IS256	-	1372	605767-607132
A2-HR	10	ISBcen18	IS256	-	1372	197555-198920
A2-HR	11	ISBcen18	IS256	-	1372	169644-171009
A2-HR	12	ISBcen18	IS256	-	1372	166915-168280
A2-HR	13	ISBcen18	IS256	-	1372	142085-143450
A2-HR	14	ISBcen18	IS256	-	1372	1-1359
A2-HR	15	ISBcen18	IS256	-	1372	504074-505154
A2-HR	16	ISBcen18	IS256	-	1372	2303-2565
A2-HR	1	ISRso10	IS3	IS2	1335	984968-986302
A2-HR	2	ISRso10	IS3	IS2	1335	883180-884514
A2-HR	3	ISRso10	IS3	IS2	1335	1291135-1292469
A2-HR	4	ISRso10	IS3	IS2	1335	165370-166704
A2-HR	5	ISRso10	IS3	IS2	1335	496691-498025
A2-HR	6	ISRso10	IS3	IS2	1335	140540-141874
A2-HR	7	ISRso10	IS3	IS2	1335	1065020-1066189
A2-HR	1	ISRso16	IS3	IS407	1238	450041-451278
A2-HR	2	ISRso16	IS3	IS407	1238	594222-595459
A2-HR	3	ISRso16	IS3	IS407	1238	676041-677278
A2-HR	4	ISRso16	IS3	IS407	1238	881706-882943
A2-HR	5	ISRso16	IS3	IS407	1238	498985-500222
A2-HR	6	ISRso16	IS3	IS407	1238	1221619-1222856
A2-HR	7	ISRso16	IS3	IS407	1238	593030-594221
A2-HR	8	ISRso16	IS3	IS407	1238	1361-2289
SL2729	1	IS1021	IS5	IS5	-	48229-49319
SL2729	2	IS1021	IS5	IS5	-	299299-300389
SL2729	3	IS1021	IS5	IS5	-	660027-661117
SL2729	4	IS1021	IS5	IS5	-	216668-217758
SL2729	5	IS1021	IS5	IS5	-	213731-214821
SL2729	6	IS1021	IS5	IS5	-	93010-94100
SL2729	1	ISRso16	IS3	IS407	1238	1940657-1941313
SL2729	1	ISRso18	IS5	IS5	1188	34933-35958
SL2729	2	ISRso18	IS5	IS5	1188	1743314- 1744339
SL2729	3	ISRso18	IS5	IS5	1188	344509-345534
SL2729	4	ISRso18	IS5	IS5	1188	1492355-1493380
SL2729	5	ISRso18	IS5	IS5	1188	621375-622400
SL2729	6	ISRso18	IS5	IS5	1188	1263124-1264149
SL2729	7	ISRso18	IS5	IS5	1188	1070967-1071992
SL2729	8	ISRso18	IS5	IS5	1188	1064203-1065228
SL2729	9	ISRso18	IS5	IS5	1188	812305-813330
SL2729	10	ISRso18	IS5	IS5	1188	604354-605379
SL2729	11	ISRso18	IS5	IS5	1188	600001-601026
SL2729	12	ISRso18	IS5	IS5	1188	396345-397370
SL2729	13	ISRso18	IS5	IS5	1188	51049-52074

SL2729	14	ISRso18	IS5	IS5	1188	104116-105141
SL2729	15	ISRso18	IS5	IS5	1188	1356187-1357212
SL2729	16	ISRso18	IS5	IS5	1188	1781103-1782128
SL2729	1	IS1405	IS5	IS5	1174	1780961-1782134
SL2729	2	IS1405	IS5	IS5	1174	1356181-1357354
SL2729	3	IS1405	IS5	IS5	1174	103974-105147
SL2729	4	IS1405	IS5	IS5	1174	34791-35964
SL2729	5	IS1405	IS5	IS5	1174	51043-52216
SL2729	6	IS1405	IS5	IS5	1174	396339-397512
SL2729	7	IS1405	IS5	IS5	1174	599995-601168
SL2729	8	IS1405	IS5	IS5	1174	604348-605521
SL2729	9	IS1405	IS5	IS5	1174	812299-813472
SL2729	10	IS1405	IS5	IS5	1174	1064061-1065234
SL2729	11	IS1405	IS5	IS5	1174	1070961-1072134
SL2729	12	IS1405	IS5	IS5	1174	1263118-1264291
SL2729	13	IS1405	IS5	IS5	1174	621233-622406
SL2729	14	IS1405	IS5	IS5	1174	1492349-1493522
SL2729	15	IS1405	IS5	IS5	1174	344367-345540
SL2729	16	IS1405	IS5	IS5	1174	1743308-1744481
SL2729	1	IS1420	IS5	IS903	1131	116050-117180
SL2729	2	IS1420	IS5	IS903	1131	597951-599081
SL2729	1	ISRso1	IS5	-	884	1335564-1336447
SL2729	2	ISRso1	IS5	-	884	782219-783102
SL2729	3	ISRso1	IS5	-	884	1297287-1298170
SL2729	4	ISRso1	IS5	-	884	862810-863693
SL2729	5	ISRso1	IS5	-	884	752308-753183
SL2729	1	IS1421	IS5	IS427	864	865303-866166
SL2729	2	IS1421	IS5	IS427	864	1799879-1800740
SL2729	3	IS1421	IS5	IS427	864	617233-618094
SL2729	4	IS1421	IS5	IS427	864	102037-102898
SL2729	5	IS1421	IS5	IS427	864	300507-301364
T110	11	ISRso1	IS5	-	-	748642-749515
T110	12	ISRso10	IS3	IS2	1335	599324-600284
T110	23	ISRso16	IS3	IS407	1238	1951174-1951930
T110	13	ISRso18	IS5	IS5	1188	521366-522391
T110	14	ISRso18	IS5	IS5	1188	1463045-1464070
T110	15	ISRso18	IS5	IS5	1188	1069303-1070328
T110	16	ISRso18	IS5	IS5	1188	1574973-1575998
T110	17	ISRso18	IS5	IS5	1188	87819-88843
T110	18	ISRso18	IS5	IS5	1188	833706-834730
T110	19	ISRso18	IS5	IS5	1188	1262311-1263335
T110	20	ISRso18	IS5	IS5	1188	1299342-1300366
T110	1	IS1405	IS5	IS5	1174	521224-522397
T110	2	IS1405	IS5	IS5	1174	1574832-1576004
T110	3	IS1405	IS5	IS5	1174	1069297-1070469
T110	4	IS1405	IS5	IS5	1174	1463039-1464211
T110	5	IS1405	IS5	IS5	1174	1262170-1263341
T110	6	IS1405	IS5	IS5	1174	833700-834871
T110	7	IS1405	IS5	IS5	1174	1299336-1300507
T110	8	IS1405	IS5	IS5	1174	87678-88849
T110	21	ISRsy1	IS1595	ISSod11	1090	1536171-1537168
T110	22	ISRama1	IS1595	ISSod11	1090	1536171-1537172
T110	10	ISRso1	IS5	-	884	95496-96377
T110	9	IS1421	IS5	IS427	864	664569-665429
PSI07	1	ISRso10	IS3	IS2	1335	1744456-1745776
PSI07	2	ISRso10	IS3	IS2	1335	286007-287327
PSI07	1	ISRso14	IS3	IS407	1234	1875066-1876289
PSI07	1	IS1421	IS3	IS427	864	824990-825816
KACC10722	-	-	-	-	-	-
SL3175	1	ISRso16	IS3	IS407	1238	623872-625109

SL3175	1	IS407	IS3	IS407	1236	4662-5728
SL3175	1	ISRso14	IS3	IS407	1234	1784849-1786072
SL3175	2	ISRso14	IS3	IS407	1234	4628-5861
SL3175	1	ISRs016	IS3	IS407	1143	623872-625109
SL3175	1	IS407	IS3	IS407	1118	4662 -5728
SL3175	2	ISRso14	IS3	IS407	1117	4628-5861
SL3175	1	ISRso14	IS3	IS407	1107	1784849-1786072
SL3175	1	IS1421	IS5	IS427	864	727803-728629
SL3175	1	IS1421	IS5	IS427	803	727803 - 728629
T98	1	ISRso16	IS3	IS407	1238	623882-625119
T98	1	IS407	IS3	IS407	1236	4662-5728
T98	1	ISRso14	IS3	IS407	1234	1784847-1786070
T98	2	ISRso14	IS3	IS407	1234	4628-5861
T98	1	IS1421	IS5	IS427	864	727813-728639
T11	-	-	-	-	-	-
T12	1	ISRso9	IS5	IS5	1483	1784687-1785518

Table S4c. Characterization of Insertion sequences elements found in the megaplasmid of *Ralstonia* spp. with OASIS

Strain	IS name	ORF Right End	ORF Left End	Family	Group
GMI1000	CP025985	1805256	1806485	family "IS3"	group "IS51"
GMI1000	CP025985	2126401	2127630	family "IS3"	group "IS51"
GMI1000	CP025985	2725697	2726926	family "IS3"	group "IS51"
GMI1000	CP025985	3376174	3377403	family "IS3"	group "IS51"
GMI1000	CP025985	11651	12867	family "IS3"	group "IS51"
GMI1000	CP025985	944628	945522	family "IS3"	group "IS51"
GMI1000	CP025985	2071814	2072540	family "IS3"	group "IS51"
GMI1000	CP025985	36216	37395	family "IS5"	group "IS5"
GMI1000	CP025985	352806	353985	family "IS5"	group "IS5"
GMI1000	CP025985	630513	631692	family "IS5"	group "IS5"
GMI1000	CP025985	1089448	1090627	family "IS5"	group "IS5"
GMI1000	CP025985	1210526	1211705	family "IS5"	group "IS5"
GMI1000	CP025985	1503373	1504552	family "IS5"	group "IS5"
GMI1000	CP025985	1674168	1675347	family "IS5"	group "IS5"
GMI1000	CP025985	3158515	3159694	family "IS5"	group "IS5"
GMI1000	CP025985	1755879	1757056	family "IS5"	group "IS5"
GMI1000	CP025985	1208231	1209407	family "IS5"	group "IS5"
GMI1000	CP025985	1894182	1895358	family "IS5"	group "IS5"
GMI1000	CP025985	2191869	2193045	family "IS5"	group "IS5"
GMI1000	CP025985	2756160	2757336	family "IS5"	group "IS5"
GMI1000	CP025985	1085977	1087152	family "IS5"	group "IS5"
GMI1000	CP025985	990714	991887	family "IS5"	group "IS5"
GMI1000	CP025985	1512948	1514121	family "IS5"	group "IS5"
GMI1000	CP025985	2714122	2715298	family "IS5"	group "IS5"
GMI1000	CP025985	2747970	2749144	family "IS5"	group "IS5"
GMI1000	CP025985	3756736	3757909	family "IS5"	group "IS5"
GMI1000	CP025985	204725	207274	family "IS66"	group ""
GMI1000	CP025985	647266	649815	family "IS66"	group ""
GMI1000	CP025985	938410	940959	family "IS66"	group ""
GMI1000	CP025985	1368045	1370594	family "IS66"	group ""
GMI1000	CP025985	1900593	1903142	family "IS66"	group ""
GMI1000	CP025985	1910694	1913243	family "IS66"	group ""
GMI1000	CP025985	2268020	2270569	family "IS66"	group ""
GMI1000	CP025985	2585635	2588184	family "IS66"	group ""
GMI1000	CP025985	2753025	2755574	family "IS66"	group ""
GMI1000	CP025985	3245460	3248009	family "IS66"	group ""
GMI1000	CP025985	3333835	3336384	family "IS66"	group ""
GMI1000	CP025985	1304795	1307344	family "IS66"	group ""
GMI1000	CP025985	2069264	2071813	family "IS66"	group ""
GMI1000	CP025985	3079483	3080941	family "IS66"	group ""
GMI1000	CP025985	3082095	3083189	family "IS66"	group ""
GMI1000	CP025985	943309	944627	family "IS3"	group "IS51"
GMI1000	CP025985	981598	982916	family "IS3"	group "IS51"
GMI1000	CP025985	1081977	1083295	family "IS3"	group "IS51"
GMI1000	CP025985	1240145	1241463	family "IS3"	group "IS51"
GMI1000	CP025985	1315849	1317167	family "IS3"	group "IS51"
GMI1000	CP025985	1474241	1475559	family "IS3"	group "IS51"
GMI1000	CP025985	1806930	1808248	family "IS3"	group "IS51"

GMI1000	CP025985	1913811	1915129	family "IS3"	group "IS51"
GMI1000	CP025985	2594248	2595566	family "IS3"	group "IS51"
GMI1000	CP025985	2729839	2731157	family "IS3"	group "IS51"
GMI1000	CP025985	211702	213018	family "IS3"	group "IS51"
GMI1000	CP025985	2513235	2514100	family "IS5"	group "IS427"
GMI1000	CP025985	3207013	3207878	family "IS5"	group "IS427"
GMI1000	CP025985	3727532	3728397	family "IS5"	group "IS427"
GMI1000	CP025985	288787	289652	family "IS5"	group "IS427"
GMI1000	CP025985	3661313	3662178	family "IS5"	group "IS427"
GMI1000	CP025985	2747109	2747969	family "IS5"	group "IS427"
GMI1000	CP025985	2715296	2716154	family "IS5"	group "IS427"
GMI1000	CP025985	3316078	3316821	family "IS5"	group "IS427"
GMI1000	CP025985	314507	315720	family "IS5"	group "IS5"
GMI1000	CP025985	1354262	1355475	family "IS5"	group "IS5"
GMI1000	CP025985	355141	356350	family "IS5"	group "IS5"
GMI1000	CP025985	415401	416610	family "IS5"	group "IS5"
GMI1000	CP025985	740841	742050	family "IS5"	group "IS5"
GMI1000	CP025985	983332	984541	family "IS5"	group "IS5"
GMI1000	CP025985	1216235	1217444	family "IS5"	group "IS5"
GMI1000	CP025985	1321595	1322804	family "IS5"	group "IS5"
GMI1000	CP025985	1444722	1445931	family "IS5"	group "IS5"
GMI1000	CP025985	1675346	1676555	family "IS5"	group "IS5"
GMI1000	CP025985	1679401	1680610	family "IS5"	group "IS5"
GMI1000	CP025985	2867693	2868902	family "IS5"	group "IS5"
GMI1000	CP025985	3352907	3354116	family "IS5"	group "IS5"
GMI1000	CP025985	3398429	3399638	family "IS5"	group "IS5"
GMI1000	CP025985	2502408	2503614	family "IS5"	group "IS5"
GMI1000	CP025985	2957054	2958263	family "IS5"	group "IS5"
GMI1000	CP025985	353983	355143	family "IS630"	group "-"
GMI1000	CP025985	971450	972610	family "IS630"	group "-"
GMI1000	CP025985	984542	985702	family "IS630"	group "-"
GMI1000	CP025985	1163431	1164591	family "IS630"	group "-"
GMI1000	CP025985	1215074	1216234	family "IS630"	group "-"
GMI1000	CP025985	1320434	1321594	family "IS630"	group "-"
GMI1000	CP025985	1443564	1444724	family "IS630"	group "-"
GMI1000	CP025985	1673010	1674170	family "IS630"	group "-"
GMI1000	CP025985	1809049	1810209	family "IS630"	group "-"
GMI1000	CP025985	2056950	2058110	family "IS630"	group "-"
GMI1000	CP025985	3080938	3082098	family "IS630"	group "-"
GMI1000	CP025985	3336385	3337032	family "IS630"	group "-"
GMI1000	CP025985	649815	650336	family "IS630"	group "-"
GMI1000	CP025985	614484	616142	family "IS1182"	group ""
GMI1000	CP025985	903737	905395	family "IS1182"	group ""
GMI1000	CP025985	1539634	1541292	family "IS1182"	group ""
GMI1000	CP025985	2197369	2199027	family "IS1182"	group ""
GMI1000	CP025985	3067639	3069297	family "IS1182"	group ""
GMI1000	CP025985	3314425	3316083	family "IS1182"	group ""

GMI1000	CP025985	3343568	3345226	family "IS1182"	group ""
GMI1000	CP025985	3646128	3647786	family "IS1182"	group ""
GMI1000	CP025985	2528719	2530369	family "IS1182"	group ""
GMI1000	CP025985	1804136	1805254	family "IS1182"	group ""
GMI1000	CP025985	985748	987218	family "IS701"	group "-"
GMI1000	CP025985	3317054	3318524	family "IS701"	group "-"
GMI1000	CP025985	1897784	1899254	family "IS701"	group "-"
GMI1000	CP025985	215107	216577	family "IS701"	group "-"
GMI1000	CP025985	1758263	1759733	family "IS701"	group "-"
GMI1000	CP025985	1810235	1811705	family "IS701"	group "-"
GMI1000	CP025985	3160983	3162453	family "IS701"	group "-"
GMI1000	CP025985	1353161	1354268	family "IS701"	group "-"
GMI1000	CP025985	1088218	1089468	family "IS256"	group "-"
GMI1000	CP025985	3157285	3158535	family "IS256"	group "-"
GMI1000	CP025985	417569	418801	family "IS256"	group "-"
GMI1000	CP025985	2229261	2230493	family "IS256"	group "-"
GMI1000	CP025985	3133488	3134720	family "IS256"	group "-"
GMI1000	CP025985	282817	284049	family "IS256"	group "-"
GMI1000	CP025985	1099365	1100813	family "IS3"	group "IS150"
GMI1000	CP025985	2970628	2972076	family "IS3"	group "IS150"
GMI1000	CP025985	3385248	3386696	family "IS3"	group "IS150"
GMI1000	CP025985	1915378	1916826	family "IS3"	group "IS150"
GMI1000	CP025985	1207024	1208006	family "IS3"	group "IS3"
GMI1000	CP025985	2712908	2713886	family "IS3"	group "IS3"
GMI1000	CP025985	1313230	1315689	family "IS110"	group "IS1111"
GMI1000	CP025985	2571750	2574209	family "IS110"	group "IS1111"
GMI1000	CP025985	3370963	3373422	family "IS110"	group "IS1111"
GMI1000	CP025985	3077624	3079482	family "IS110"	group "IS1111"
GMI1000	CP025985	209829	211136	family "IS110"	group "IS1111"
GMI1000	CP025985	3209841	3211148	family "IS110"	group "IS1111"
GMI1000	CP025985	1903143	1904320	family "IS110"	group "IS1111"
GMI1000	CP025985	3083190	3083798	family "IS110"	group "IS1111"
GMI1000	CP025985	3747387	3748760	family "IS4"	group ""
GMI1000	CP025985	3755362	3756735	family "IS4"	group ""
GMI1000	CP025985	3073929	3075287	family "IS4"	group ""
GMI1000	CP025985	778986	780340	family "IS4"	group ""
GMI1000	CP025985	1602617	1603971	family "IS4"	group ""
GMI1000	CP025985	739476	740840	family "IS4"	group ""
GMI1000	CP025985	2614808	2616164	family "IS4"	group ""
GMI1000	CP025985	3424076	3425432	family "IS4"	group ""
GMI1000	CP025985	2524578	2525933	family "IS4"	group ""
GMI1000	CP025985	3615482	3616837	family "IS4"	group ""
GMI1000	CP025985	307352	308726	family "IS4"	group ""
GMI1000	CP025985	553331	554685	family "IS4"	group ""
GMI1000	CP025985	2445450	2446804	family "IS4"	group ""
GMI1000	CP025985	3478313	3479667	family "IS4"	group ""
GMI1000	CP025985	2213468	2214824	family "IS4"	group ""
GMI1000	CP025985	2388885	2390240	family "IS4"	group ""
GMI1000	CP025985	1129234	1130588	family "IS4"	group ""

GMI1000	CP025985	271030	271914	family "IS5"	group "-"
GMI1000	CP025985	3162457	3163342	family "IS5"	group "-"
GMI1000	CP025985	976109	976994	family "IS5"	group "-"
GMI1000	CP025985	1757374	1758259	family "IS5"	group "-"
GMI1000	CP025985	3645117	3646002	family "IS5"	group "-"
GMI1000	CP025985	1811709	1812585	family "IS5"	group "-"
GMI1000	CP025985	2727566	2728451	family "IS5"	group "-"
GMI1000	CP025985	566750	567598	family "None"	group "None"
GMI1000	CP025985	1464681	1465545	family "IS5"	group "IS427"
GMI1000	CP025985	5637	6502	family "IS5"	group "IS427"
GMI1000	CP025985	12866	13731	family "IS5"	group "IS427"
GMI1000	CP025985	932916	933781	family "IS5"	group "IS427"
GMI1000	CP025985	1011358	1012223	family "IS5"	group "IS427"
GMI1000	CP025985	2062752	2063617	family "IS5"	group "IS427"
GMI1000	CP025985	2072539	2073404	family "IS5"	group "IS427"
GMI1000	CP025985	2081248	2082113	family "IS5"	group "IS427"
GMI1000	CP025985	2728453	2729318	family "IS5"	group "IS427"
GMI1000	CP025985	3342667	3343532	family "IS5"	group "IS427"
GMI1000	CP025985	642799	643650	family "IS5"	group "IS427"
GMI1000	CP025985	3642838	3643689	family "IS5"	group "IS427"
GMI1000	CP025985	2746251	2747071	family "IS5"	group "IS427"
GMI1000	CP025985	1310424	1311251	family "IS5"	group "IS427"
Po82	CP002820.1	69820	71706	family "IS21"	group "-"
Po82	CP002820.1	463474	465360	family "IS21"	group "-"
Po82	CP002820.1	623451	625337	family "IS21"	group "-"
Po82	CP002820.1	1056366	1058252	family "IS21"	group "-"
Po82	CP002820.1	1634413	1636299	family "IS21"	group "-"
Po82	CP002820.1	124161	125498	family "IS3"	group "IS2"
Po82	CP002820.1	877254	878591	family "IS3"	group "IS2"
Po82	CP002820.1	646190	647525	family "IS3"	group "IS2"
UY031	CP012688.1	108642	109858	family "IS5"	group "IS5"
UY031	CP012688.1	123446	124662	family "IS5"	group "IS5"
UY031	CP012688.1	177519	178735	family "IS5"	group "IS5"
UY031	CP012688.1	218534	219750	family "IS5"	group "IS5"
UY031	CP012688.1	434077	435293	family "IS5"	group "IS5"
UY031	CP012688.1	525648	526864	family "IS5"	group "IS5"
UY031	CP012688.1	646208	647424	family "IS5"	group "IS5"
UY031	CP012688.1	657982	659198	family "IS5"	group "IS5"
UY031	CP012688.1	759872	761088	family "IS5"	group "IS5"
UY031	CP012688.1	928390	929606	family "IS5"	group "IS5"
UY031	CP012688.1	931168	932384	family "IS5"	group "IS5"
UY031	CP012688.1	957913	959129	family "IS5"	group "IS5"
UY031	CP012688.1	1100413	1101629	family "IS5"	group "IS5"
UY031	CP012688.1	1316888	1318104	family "IS5"	group "IS5"
UY031	CP012688.1	1361720	1362936	family "IS5"	group "IS5"
UY031	CP012688.1	1388926	1390142	family "IS5"	group "IS5"
UY031	CP012688.1	1554467	1555683	family "IS5"	group "IS5"
UY031	CP012688.1	1658808	1660024	family "IS5"	group "IS5"
UY031	CP012688.1	1670915	1672131	family "IS5"	group "IS5"

UY031	CP012688.1	1864276	1865492	family "IS5"	group "IS5"
UY031	CP012688.1	1894992	1896208	family "IS5"	group "IS5"
UY031	CP012688.1	1910032	1911248	family "IS5"	group "IS5"
UY031	CP012688.1	1911245	1912461	family "IS5"	group "IS5"
UY031	CP012688.1	1931033	1932249	family "IS5"	group "IS5"
UY031	CP012688.1	1940554	1941770	family "IS5"	group "IS5"
UY031	CP012688.1	153943	155157	family "IS5"	group "IS5"
UY031	CP012688.1	195360	196574	family "IS5"	group "IS5"
UY031	CP012688.1	383446	384660	family "IS5"	group "IS5"
UY031	CP012688.1	524160	525374	family "IS5"	group "IS5"
UY031	CP012688.1	1613928	1615142	family "IS5"	group "IS5"
UY031	CP012688.1	747514	748728	family "IS5"	group "IS5"
UY031	CP012688.1	352937	354149	family "IS5"	group "IS5"
UY031	CP012688.1	360244	361456	family "IS5"	group "IS5"
UY031	CP012688.1	384802	386014	family "IS5"	group "IS5"
UY031	CP012688.1	522458	523670	family "IS5"	group "IS5"
UY031	CP012688.1	644671	645883	family "IS5"	group "IS5"
UY031	CP012688.1	1649131	1650347	family "IS5"	group "IS5"
UY031	CP012688.1	1819972	1821184	family "IS5"	group "IS5"
UY031	CP012688.1	265069	266280	family "IS5"	group "IS5"
UY031	CP012688.1	533349	534560	family "IS5"	group "IS5"
UY031	CP012688.1	704614	705825	family "IS5"	group "IS5"
UY031	CP012688.1	736718	737929	family "IS5"	group "IS5"
UY031	CP012688.1	1153593	1154804	family "IS5"	group "IS5"
UY031	CP012688.1	1433123	1434334	family "IS5"	group "IS5"
UY031	CP012688.1	1652061	1653272	family "IS5"	group "IS5"
UY031	CP012688.1	1710161	1711372	family "IS5"	group "IS5"
UY031	CP012688.1	1756019	1757230	family "IS5"	group "IS5"
UY031	CP012688.1	361451	362577	family "IS3"	group "IS3"
UY031	CP012688.1	730022	731146	family "IS3"	group "IS3"
UY031	CP012688.1	521615	522457	family "IS5"	group "IS1031"
UY031	CP012688.1	1394584	1395426	family "IS5"	group "IS1031"
UY031	CP012688.1	1819119	1819961	family "IS5"	group "IS1031"
UY031	CP012688.1	115077	116412	family "IS256"	group "-"
UW163	CP012940	384009	385895	family "IS21"	group "-"
UW163	CP012940	509650	511536	family "IS21"	group "-"
UW163	CP012940	1130959	1132845	family "IS21"	group "-"
UW163	CP012940	1537013	1538899	family "IS21"	group "-"
UW163	CP012940	1	1443	family "IS21"	group "-"
UW163	CP012940	1931489	1932001	family "IS21"	group "-"
UW163	CP012940	532401	533736	family "IS3"	group "IS2"
UW163	CP012940	1309750	1311085	family "IS3"	group "IS2"
UW163	CP012940	1870763	1872098	family "IS3"	group "IS2"
UW163	CP012940	1662419	1663250	family "IS1595"	group "IS1016"
UW163	CP012940	1813200	1814031	family "IS1595"	group "IS1016"
IBSBF1503	CP012944.1	710653	712012	family "IS21"	group "-"
IBSBF1503	CP012944.1	1409078	1410437	family "IS21"	group "-"
IBSBF1503	CP012944.1	1517736	1519095	family "IS21"	group "-"

IBSBF1503	CP012944.1	1935156	1936515	family "IS21"	group "-"
IBSBF1503	CP012944.1	1361855	1363214	family "IS21"	group "-"
RS488	CP021653	108642	109858	family "IS5"	group "IS5"
RS488	CP021653	123446	124662	family "IS5"	group "IS5"
RS488	CP021653	177510	178726	family "IS5"	group "IS5"
RS488	CP021653	218525	219741	family "IS5"	group "IS5"
RS488	CP021653	434068	435284	family "IS5"	group "IS5"
RS488	CP021653	525639	526855	family "IS5"	group "IS5"
RS488	CP021653	646199	647415	family "IS5"	group "IS5"
RS488	CP021653	657973	659189	family "IS5"	group "IS5"
RS488	CP021653	759865	761081	family "IS5"	group "IS5"
RS488	CP021653	928383	929599	family "IS5"	group "IS5"
RS488	CP021653	931161	932377	family "IS5"	group "IS5"
RS488	CP021653	957906	959122	family "IS5"	group "IS5"
RS488	CP021653	1100406	1101622	family "IS5"	group "IS5"
RS488	CP021653	1316881	1318097	family "IS5"	group "IS5"
RS488	CP021653	1361713	1362929	family "IS5"	group "IS5"
RS488	CP021653	1388919	1390135	family "IS5"	group "IS5"
RS488	CP021653	1554469	1555685	family "IS5"	group "IS5"
RS488	CP021653	1658810	1660026	family "IS5"	group "IS5"
RS488	CP021653	1670917	1672133	family "IS5"	group "IS5"
RS488	CP021653	1864278	1865494	family "IS5"	group "IS5"
RS488	CP021653	1894994	1896210	family "IS5"	group "IS5"
RS488	CP021653	1910034	1911250	family "IS5"	group "IS5"
RS488	CP021653	1911247	1912463	family "IS5"	group "IS5"
RS488	CP021653	1931035	1932251	family "IS5"	group "IS5"
RS488	CP021653	1940556	1941772	family "IS5"	group "IS5"
RS488	CP021653	153943	155157	family "IS5"	group "IS5"
RS488	CP021653	195351	196565	family "IS5"	group "IS5"
RS488	CP021653	383437	384651	family "IS5"	group "IS5"
RS488	CP021653	524151	525365	family "IS5"	group "IS5"
RS488	CP021653	1613930	1615144	family "IS5"	group "IS5"
RS488	CP021653	747507	748721	family "IS5"	group "IS5"
RS488	CP021653	352928	354140	family "IS5"	group "IS5"
RS488	CP021653	360235	361447	family "IS5"	group "IS5"
RS488	CP021653	384793	386005	family "IS5"	group "IS5"
RS488	CP021653	522449	523661	family "IS5"	group "IS5"
RS488	CP021653	644662	645874	family "IS5"	group "IS5"
RS488	CP021653	1649133	1650349	family "IS5"	group "IS5"
RS488	CP021653	1819974	1821186	family "IS5"	group "IS5"
RS488	CP021653	265060	266271	family "IS5"	group "IS5"
RS488	CP021653	533340	534551	family "IS5"	group "IS5"
RS488	CP021653	704606	705817	family "IS5"	group "IS5"
RS488	CP021653	736710	737921	family "IS5"	group "IS5"
RS488	CP021653	1153586	1154797	family "IS5"	group "IS5"
RS488	CP021653	1433116	1434327	family "IS5"	group "IS5"
RS488	CP021653	1652063	1653274	family "IS5"	group "IS5"
RS488	CP021653	1710163	1711374	family "IS5"	group "IS5"
RS488	CP021653	1756021	1757232	family "IS5"	group "IS5"
RS488	CP021653	361442	362568	family "IS3"	group "IS3"

RS488	CP021653	730014	731138	family "IS3"	group "IS3"
RS488	CP021653	521606	522448	family "IS5"	group "IS1031"
RS488	CP021653	1394577	1395419	family "IS5"	group "IS1031"
RS488	CP021653	1819121	1819963	family "IS5"	group "IS1031"
RS488	CP021653	115077	116412	family "IS256"	group "-"
UW551	NCTI01000002.1	19410	20270	family "IS5"	group "IS1031"
UW551	NCTI01000002.1	1998843	1999703	family "IS5"	group "IS1031"
UW551	NCTI01000002.1	1574323	1575183	family "IS5"	group "IS1031"
UW551	NCTI01000002.1	674444	675286	family "IS5"	group "IS1031"
UW551	NCTI01000002.1	20268	21484	family "IS5"	group "IS5"
UW551	NCTI01000002.1	505768	506984	family "IS5"	group "IS5"
UW551	NCTI01000002.1	513071	514287	family "IS5"	group "IS5"
UW551	NCTI01000002.1	706357	707573	family "IS5"	group "IS5"
UW551	NCTI01000002.1	825654	826870	family "IS5"	group "IS5"
UW551	NCTI01000002.1	1999701	2000917	family "IS5"	group "IS5"
UW551	NCTI01000002.1	417897	419112	family "IS5"	group "IS5"
UW551	NCTI01000002.1	1334557	1335772	family "IS5"	group "IS5"
UW551	NCTI01000002.1	1612871	1614086	family "IS5"	group "IS5"
UW551	NCTI01000002.1	64575	65787	family "IS5"	group "IS5"
UW551	NCTI01000002.1	84361	85573	family "IS5"	group "IS5"
UW551	NCTI01000002.1	93882	95094	family "IS5"	group "IS5"
UW551	NCTI01000002.1	261483	262695	family "IS5"	group "IS5"
UW551	NCTI01000002.1	276287	277499	family "IS5"	group "IS5"
UW551	NCTI01000002.1	330355	331567	family "IS5"	group "IS5"
UW551	NCTI01000002.1	348191	349403	family "IS5"	group "IS5"
UW551	NCTI01000002.1	371370	372582	family "IS5"	group "IS5"
UW551	NCTI01000002.1	537632	538844	family "IS5"	group "IS5"
UW551	NCTI01000002.1	586911	588123	family "IS5"	group "IS5"
UW551	NCTI01000002.1	827191	828403	family "IS5"	group "IS5"
UW551	NCTI01000002.1	838965	840177	family "IS5"	group "IS5"
UW551	NCTI01000002.1	940853	942065	family "IS5"	group "IS5"
UW551	NCTI01000002.1	1109368	1110580	family "IS5"	group "IS5"
UW551	NCTI01000002.1	1112142	1113354	family "IS5"	group "IS5"
UW551	NCTI01000002.1	1138887	1140099	family "IS5"	group "IS5"
UW551	NCTI01000002.1	1281388	1282600	family "IS5"	group "IS5"
UW551	NCTI01000002.1	1497852	1499064	family "IS5"	group "IS5"
UW551	NCTI01000002.1	1568678	1569890	family "IS5"	group "IS5"
UW551	NCTI01000002.1	1734211	1735423	family "IS5"	group "IS5"
UW551	NCTI01000002.1	1793667	1794879	family "IS5"	group "IS5"
UW551	NCTI01000002.1	1835698	1836910	family "IS5"	group "IS5"
UW551	NCTI01000002.1	1838545	1839757	family "IS5"	group "IS5"
UW551	NCTI01000002.1	1850652	1851864	family "IS5"	group "IS5"
UW551	NCTI01000002.1	306783	307994	family "IS5"	group "IS5"
UW551	NCTI01000002.1	536276	537487	family "IS5"	group "IS5"
UW551	NCTI01000002.1	676992	678203	family "IS5"	group "IS5"
UW551	NCTI01000002.1	714058	715269	family "IS5"	group "IS5"
UW551	NCTI01000002.1	885597	886807	family "IS5"	group "IS5"
UW551	NCTI01000002.1	928499	929709	family "IS5"	group "IS5"
UW551	NCTI01000002.1	917699	918908	family "IS5"	group "IS5"
UW551	NCTI01000002.1	675287	676495	family "IS5"	group "IS5"

UW551	NCTI01000002.1	1828873	1830085	family "IS5"	group "IS5"
UW551	NCTI01000002.1	1889897	1891105	family "IS5"	group "IS5"
UW551	NCTI01000002.1	1935754	1936962	family "IS5"	group "IS5"
UW551	NCTI01000002.1	1333623	1334487	family "IS5"	group "IS427"
UW551	NCTI01000002.1	479818	480683	family "IS5"	group "IS427"
UW551	NCTI01000002.1	1570813	1571678	family "IS5"	group "IS427"
UW551	NCTI01000002.1	590458	591325	family "IS5"	group "IS427"
UW551	NCTI01000002.1	277704	278569	family "IS5"	group "IS427"
UW551	NCTI01000002.1	1832790	1833654	family "IS5"	group "IS427"
UW551	NCTI01000002.1	83393	84258	family "IS5"	group "IS427"
UW551	NCTI01000002.1	63607	64472	family "IS5"	group "IS427"
UW551	NCTI01000002.1	1573188	1574053	family "IS5"	group "IS427"
UW551	NCTI01000002.1	406669	407534	family "IS5"	group "IS427"
UW551	NCTI01000002.1	266817	267682	family "IS5"	group "IS427"
UW551	NCTI01000002.1	512066	512931	family "IS5"	group "IS427"
UW551	NCTI01000002.1	267918	269253	family "IS256"	group "-"
UW551	NCTI01000002.1	829452	830319	family "IS5"	group "IS427"
RS489	No IS(s) found using OASIS Predictor...				
OE11	CP009763	205655	206834	family "IS5"	group "IS5"
OE11	CP009763	445533	446712	family "IS5"	group "IS5"
OE11	CP009763	610175	611351	family "IS5"	group "IS5"
OE11	CP009763	637257	638430	family "IS5"	group "IS5"
OE11	CP009763	653033	653902	family "IS5"	group "IS427"
OE11	CP009763	746037	746902	family "IS5"	group "IS427"
OE11	CP009763	856981	857861	family "IS5"	group "-"
OE11	CP009763	1031019	1031899	family "IS5"	group "-"
OE11	CP009763	747916	748792	family "IS5"	group "-"
OE11	CP009763	857870	859340	family "IS701"	group "-"
OE11	CP009763	1740614	1742084	family "IS701"	group "-"
OE11	CP009763	1890061	1890769	family "IS3"	group "IS407"
OE11	CP009763	1892153	1892857	family "IS3"	group "IS407"
OE11	CP009763	192550	193906	family "IS110"	group "IS1111"
OE11	CP009763	448953	450421	family "IS4"	group ""
OE11	CP009763	606116	607347	family "IS3"	group "IS51"
OE11	CP009763	1595406	1596886	family "IS3"	group "IS150"
FJAT1458	CP016555	104937	106152	family "IS5"	group "IS5"
FJAT1458	CP016555	179533	180748	family "IS5"	group "IS5"
FJAT1458	CP016555	738360	739575	family "IS5"	group "IS5"
FJAT1458	CP016555	1775742	1776957	family "IS5"	group "IS5"
FJAT1458	CP016555	1879221	1880436	family "IS5"	group "IS5"
FJAT1458	CP016555	351442	352655	family "IS5"	group "IS5"
FJAT1458	CP016555	1822378	1823589	family "IS5"	group "IS5"
FJAT1458	CP016555	1554336	1555543	family "IS5"	group "IS5"
FJAT1458	CP016555	1352697	1353904	family "IS5"	group "IS5"
FJAT1458	CP016555	1470661	1471868	family "IS5"	group "IS5"
FJAT1458	CP016555	1922493	1923700	family "IS5"	group "IS5"
FJAT1458	CP016555	1817658	1818865	family "IS5"	group "IS5"

FJAT1458	CP016555	242528	243707	family "IS5"	group "IS5"
FJAT1458	CP016555	337720	338899	family "IS5"	group "IS5"
FJAT1458	CP016555	352654	353833	family "IS5"	group "IS5"
FJAT1458	CP016555	634895	636074	family "IS5"	group "IS5"
FJAT1458	CP016555	1314985	1316164	family "IS5"	group "IS5"
FJAT1458	CP016555	344389	345565	family "IS5"	group "IS5"
FJAT1458	CP016555	442569	443745	family "IS5"	group "IS5"
FJAT1458	CP016555	981771	982947	family "IS5"	group "IS5"
FJAT1458	CP016555	1553158	1554334	family "IS5"	group "IS5"
FJAT1458	CP016555	1938173	1939349	family "IS5"	group "IS5"
FJAT1458	CP016555	1312634	1313809	family "IS5"	group "IS5"
FJAT1458	CP016555	177389	178563	family "IS5"	group "IS5"
FJAT1458	CP016555	946978	948157	family "IS5"	group "IS5"
FJAT1458	CP016555	942306	943479	family "IS5"	group "IS5"
FJAT1458	CP016555	368100	369331	family "IS3"	group "IS51"
FJAT1458	CP016555	450551	451782	family "IS3"	group "IS51"
FJAT1458	CP016555	1164399	1165630	family "IS3"	group "IS51"
FJAT1458	CP016555	600173	602204	family "ISL3"	group ""
FJAT1458	CP016555	653772	655803	family "ISL3"	group ""
FJAT1458	CP016555	979662	981693	family "ISL3"	group ""
FJAT1458	CP016555	1170044	1172075	family "ISL3"	group ""
FJAT1458	CP016555	1680625	1682656	family "ISL3"	group ""
FJAT1458	CP016555	1661454	1663459	family "IS110"	group "IS1111"
FJAT1458	CP016555	1664843	1666844	family "IS110"	group "IS1111"
FJAT1458	CP016555	741507	742808	family "IS110"	group "IS1111"
FJAT1458	CP016555	108818	110256	family "IS4"	group ""
FJAT1458	CP016555	199849	201205	family "IS110"	group "IS1111"
FJAT1458	CP016555	338897	339813	family "IS110"	group "IS1111"
FJAT1458	CP016555	336558	337912	family "IS3"	group "IS2"
FJAT1458	CP016555	733211	734773	family "ISNCY"	group "IS1202"
FJAT1458	CP016555	734678	735353	family "None"	group "None"
EP1	CP015116	112523	113231	family "IS3"	group "IS407"
EP1	CP015116	114615	115319	family "IS3"	group "IS407"
EP1	CP015116	847254	848435	family "IS5"	group "IS5"
EP1	CP015116	1011736	1012917	family "IS5"	group "IS5"
EP1	CP015116	1713071	1714252	family "IS5"	group "IS5"
EP1	CP015116	364768	365944	family "IS5"	group "IS5"
EP1	CP015116	902950	904126	family "IS5"	group "IS5"
EP1	CP015116	876233	877690	family "IS110"	group ""
EP1	CP015116	901271	902725	family "IS110"	group ""
EP1	CP015116	918733	919588	family "IS5"	group "IS427"
EP1	CP015116	1012914	1013772	family "IS5"	group "IS427"
EP1	CP015116	1123903	1124783	family "IS5"	group "-"
EP1	CP015116	1315242	1316122	family "IS5"	group "-"
EP1	CP015116	1014786	1015662	family "IS5"	group "-"
EP1	CP015116	351663	353019	family "IS110"	group "IS1111"
EP1	CP015116	606884	608352	family "IS4"	group ""
EP1	CP015116	843196	844427	family "IS3"	group "IS51"
EP1	CP015116	1124792	1126262	family "IS701"	group "-"

EP1	CP015116	1881585	1883065	family "IS3"	group "IS150"
CQPS-1	CP016915	31739	32806	family "IS5"	group "IS5"
CQPS-1	CP016915	80702	81769	family "IS5"	group "IS5"
CQPS-1	CP016915	397002	398065	family "IS5"	group "IS5"
CQPS-1	CP016915	823627	824690	family "IS5"	group "IS5"
CQPS-1	CP016915	2019687	2020754	family "IS5"	group "IS5"
CQPS-1	CP016915	13495	14558	family "IS5"	group "IS5"
CQPS-1	CP016915	206230	207095	family "IS5"	group "IS427"
CQPS-1	CP016915	1069496	1070361	family "IS5"	group "IS427"
CQPS-1	CP016915	1095406	1096271	family "IS5"	group "IS427"
CQPS-1	CP016915	710557	711422	family "IS5"	group "IS427"
CQPS-1	CP016915	814411	815275	family "IS5"	group "IS427"
CQPS-1	CP016915	207707	209177	family "IS701"	group "-"
CQPS-1	CP016915	298726	300196	family "IS701"	group "-"
CQPS-1	CP016915	1061339	1062808	family "IS701"	group "-"
CQPS-1	CP016915	1763297	1764767	family "IS701"	group "-"
CQPS-1	CP016915	297837	298722	family "IS5"	group "-"
CQPS-1	CP016915	1327318	1328203	family "IS5"	group "-"
CQPS-1	CP016915	1590544	1591429	family "IS5"	group "-"
CQPS-1	CP016915	1764771	1765656	family "IS5"	group "-"
CQPS-1	CP016915	547875	548583	family "IS3"	group "IS407"
CQPS-1	CP016915	549967	550671	family "IS3"	group "IS407"
CQPS-1	CP016915	249101	250580	family "IS3"	group "IS150"
CQPS-1	CP016915	1789897	1791112	family "IS5"	group "IS5"
CQPS-1	CP016915	2012484	2013365	family "IS1595"	group "IS1016"
CQPS-1	CP016915	2039296	2040426	family "IS5"	group "IS903"
FJAT91	IS_9cef4912	112523	113231	family "IS3"	group "IS407"
FJAT91	IS_9cef4912	114615	115319	family "IS3"	group "IS407"
FJAT91	IS_9cef4912	847254	848435	family "IS5"	group "IS5"
FJAT91	IS_9cef4912	1011736	1012917	family "IS5"	group "IS5"
FJAT91	IS_9cef4912	1713071	1714252	family "IS5"	group "IS5"
FJAT91	IS_9cef4912	364768	365944	family "IS5"	group "IS5"
FJAT91	IS_9cef4912	902950	904126	family "IS5"	group "IS5"
FJAT91	IS_9cef4912	876233	877690	family "IS110"	group ""
FJAT91	IS_9cef4912	901271	902725	family "IS110"	group ""
FJAT91	IS_9cef4912	918733	919588	family "IS5"	group "IS427"
FJAT91	IS_9cef4912	1012914	1013772	family "IS5"	group "IS427"
FJAT91	IS_9cef4912	1123903	1124783	family "IS5"	group "-"
FJAT91	IS_9cef4912	1315242	1316122	family "IS5"	group "-"
FJAT91	IS_9cef4912	1014786	1015662	family "IS5"	group "-"
FJAT91	IS_9cef4912	351663	353019	family "IS110"	group "IS1111"
FJAT91	IS_9cef4912	606884	608352	family "IS4"	group ""
FJAT91	IS_9cef4912	843196	844427	family "IS3"	group "IS51"
FJAT91	IS_9cef4912	1124792	1126262	family "IS701"	group "-"
FJAT91	IS_9cef4912	1881585	1883065	family "IS3"	group "IS150"
FQY_4	No IS(s) found using OASIS Predictor...				

RSCM	CP025986	275170	276349	family "IS5"	group "IS5"
RSCM	CP025986	517647	518826	family "IS5"	group "IS5"
RSCM	CP025986	684375	685554	family "IS5"	group "IS5"
RSCM	CP025986	828726	829905	family "IS5"	group "IS5"
RSCM	CP025986	1384446	1385625	family "IS5"	group "IS5"
RSCM	CP025986	1958838	1960017	family "IS5"	group "IS5"
RSCM	CP025986	110783	111959	family "IS5"	group "IS5"
RSCM	CP025986	458452	459628	family "IS5"	group "IS5"
RSCM	CP025986	847580	848756	family "IS5"	group "IS5"
RSCM	CP025986	1587280	1588456	family "IS5"	group "IS5"
RSCM	CP025986	780761	781934	family "IS5"	group "IS5"
RSCM	CP025986	1138980	1140153	family "IS5"	group "IS5"
RSCM	CP025986	1588648	1589821	family "IS5"	group "IS5"
RSCM	CP025986	1667669	1668842	family "IS5"	group "IS5"
RSCM	CP025986	1869909	1871082	family "IS5"	group "IS5"
RSCM	CP025986	191536	192745	family "IS5"	group "IS5"
RSCM	CP025986	367418	368627	family "IS5"	group "IS5"
RSCM	CP025986	608891	610100	family "IS5"	group "IS5"
RSCM	CP025986	853656	854865	family "IS5"	group "IS5"
RSCM	CP025986	1765171	1766380	family "IS5"	group "IS5"
RSCM	CP025986	547965	549173	family "IS5"	group "IS5"
RSCM	CP025986	1429470	1430678	family "IS5"	group "IS5"
RSCM	CP025986	160877	162084	family "IS5"	group "IS5"
RSCM	CP025986	1527037	1528244	family "IS5"	group "IS5"
RSCM	CP025986	1325363	1326569	family "IS5"	group "IS5"
RSCM	CP025986	1578918	1580124	family "IS5"	group "IS5"
RSCM	CP025986	1777589	1778795	family "IS5"	group "IS5"
RSCM	CP025986	2141361	2142567	family "IS5"	group "IS5"
RSCM	CP025986	1594287	1595387	family "IS5"	group "IS5"
RSCM	CP025986	194413	195644	family "IS3"	group "IS51"
RSCM	CP025986	335399	336630	family "IS3"	group "IS51"
RSCM	CP025986	1380342	1381573	family "IS3"	group "IS51"
RSCM	CP025986	1621286	1622517	family "IS3"	group "IS51"
RSCM	CP025986	323980	326529	family "IS66"	group ""
RSCM	CP025986	351405	353954	family "IS66"	group ""
RSCM	CP025986	1294480	1297029	family "IS66"	group ""
RSCM	CP025986	1661309	1663858	family "IS66"	group ""
RSCM	CP025986	1842027	1844576	family "IS66"	group ""
RSCM	CP025986	585054	585919	family "IS5"	group "IS427"
RSCM	CP025986	1638867	1639732	family "IS5"	group "IS427"
RSCM	CP025986	1649024	1649889	family "IS5"	group "IS427"
RSCM	CP025986	1655725	1656590	family "IS5"	group "IS427"
RSCM	CP025986	2087069	2087934	family "IS5"	group "IS427"
RSCM	CP025986	456946	457811	family "IS5"	group "IS427"
RSCM	CP025986	1060070	1060935	family "IS5"	group "IS427"
RSCM	CP025986	461532	462383	family "IS5"	group "IS427"
RSCM	CP025986	1562087	1562938	family "IS5"	group "IS427"
RSCM	CP025986	105021	105619	family "IS5"	group "IS427"
RSCM	CP025986	914950	916386	family "IS4"	group ""
RSCM	CP025986	1137537	1138973	family "IS4"	group ""
RSCM	CP025986	685553	686983	family "IS4"	group ""

RSCM	CP025986	788544	789974	family "IS4"	group ""
RSCM	CP025986	1324006	1325362	family "IS4"	group ""
RSCM	CP025986	818107	819765	family "IS1182"	group ""
RSCM	CP025986	844091	845749	family "IS1182"	group ""
RSCM	CP025986	1196095	1197753	family "IS1182"	group ""
RSCM	CP025986	1922934	1924592	family "IS1182"	group ""
RSCM	CP025986	2007477	2009135	family "IS1182"	group ""
RSCM	CP025986	1837095	1838747	family "IS1182"	group ""
RSCM	CP025986	1602083	1603733	family "IS1182"	group ""
RSCM	CP025986	1378987	1380341	family "IS1182"	group ""
RSCM	CP025986	854847	855967	family "IS701"	group "-"
RSCM	CP025986	94395	95504	family "IS701"	group "-"
RSCM	CP025986	276717	277825	family "IS701"	group "-"
RSCM	CP025986	1778792	1779900	family "IS701"	group "-"
RSCM	CP025986	2142568	2143676	family "IS701"	group "-"
RSCM	CP025986	112327	113434	family "IS701"	group "-"
RSCM	CP025986	1559716	1560823	family "IS701"	group "-"
RSCM	CP025986	1528888	1529992	family "IS701"	group "-"
RSCM	CP025986	1846612	1847716	family "IS701"	group "-"
RSCM	CP025986	1900500	1901604	family "IS701"	group "-"
RSCM	CP025986	1430677	1431200	family "IS701"	group "-"
RSCM	CP025986	901050	902210	family "IS630"	group "-"
RSCM	CP025986	1301637	1302797	family "IS630"	group "-"
RSCM	CP025986	1766378	1767538	family "IS630"	group "-"
RSCM	CP025986	1382017	1383335	family "IS3"	group "IS51"
RSCM	CP025986	2090184	2091502	family "IS3"	group "IS51"
RSCM	CP025986	1775408	1777402	family "IS110"	group "IS1111"
RSCM	CP025986	1839582	1841573	family "IS110"	group "IS1111"
RSCM	CP025986	1298731	1300038	family "IS110"	group "IS1111"
RSCM	CP025986	347394	348698	family "IS110"	group "IS1111"
RSCM	CP025986	353954	355016	family "IS110"	group "IS1111"
RSCM	CP025986	1848067	1848759	family "IS5"	group "-"
RSCM	CP025986	95874	96415	family "IS5"	group "-"
RSCM	CP025986	1181868	1182409	family "IS5"	group "-"
RSCM	CP025986	1366890	1367431	family "IS5"	group "-"
RSCM	CP025986	1529996	1530537	family "IS5"	group "-"
RSCM	CP025986	1561537	1562078	family "IS5"	group "-"
RSCM	CP025986	1901952	1902493	family "IS5"	group "-"
RSCM	CP025986	2144046	2144587	family "IS5"	group "-"
RSCM	CP025986	135676	136217	family "IS5"	group "-"
RSCM	CP025986	2092433	2093911	family "IS3"	group "IS150"
T60	CP022769	209225	210355	family "IS5"	group "IS903"
T60	CP022769	591081	592211	family "IS5"	group "IS903"
T60	CP022769	607088	608218	family "IS5"	group "IS903"
T60	CP022769	1668335	1669465	family "IS5"	group "IS903"
T60	CP022769	614719	617122	family "IS110"	group "IS1111"

T60	CP022769	683955	686358	family "IS110"	group "IS1111"
T60	CP022769	545877	547258	family "IS110"	group "IS1111"
T60	CP022769	909088	910561	family "IS4"	group "IS50"
T60	CP022769	961547	963020	family "IS4"	group "IS50"
T60	CP022769	1428059	1429290	family "IS3"	group "IS51"
T60	CP022769	1649181	1650412	family "IS3"	group "IS51"
T60	CP022769	2021424	2023429	family "IS110"	group "IS1111"
T60	CP022769	2024813	2026814	family "IS110"	group "IS1111"
T60	CP022769	595083	597254	family "Tn3"	group ""
T60	CP022769	935430	936315	family "IS5"	group "-"
T60	CP022769	1247324	1248209	family "IS5"	group "-"
T60	CP022769	1674655	1675540	family "IS5"	group "-"
T60	CP022769	1417949	1418834	family "IS5"	group "-"
T60	CP022769	824345	825221	family "IS5"	group "-"
T60	CP022769	1438552	1439730	family "IS5"	group "IS5"
T60	CP022769	99430	100607	family "IS5"	group "IS5"
T60	CP022769	1864248	1865425	family "IS5"	group "IS5"
T60	CP022769	48420	49597	family "IS5"	group "IS5"
T60	CP022769	542676	543853	family "IS5"	group "IS5"
T60	CP022769	588603	589780	family "IS5"	group "IS5"
T60	CP022769	692456	693633	family "IS5"	group "IS5"
T60	CP022769	883436	884613	family "IS5"	group "IS5"
T60	CP022769	1341205	1342382	family "IS5"	group "IS5"
T60	CP022769	1574758	1575935	family "IS5"	group "IS5"
T60	CP022769	1141267	1142443	family "IS5"	group "IS5"
T60	CP022769	688102	689279	family "IS5"	group "IS5"
T60	CP022769	1060151	1061324	family "IS5"	group "IS5"
T60	CP022769	1148170	1149347	family "IS5"	group "IS5"
SL3882	CP022779	48417	49596	family "IS5"	group "IS5"
SL3882	CP022779	542690	543869	family "IS5"	group "IS5"
SL3882	CP022779	588605	589784	family "IS5"	group "IS5"
SL3882	CP022779	883422	884601	family "IS5"	group "IS5"
SL3882	CP022779	1141256	1142435	family "IS5"	group "IS5"
SL3882	CP022779	1341191	1342370	family "IS5"	group "IS5"
SL3882	CP022779	688109	689288	family "IS5"	group "IS5"
SL3882	CP022779	692462	693638	family "IS5"	group "IS5"
SL3882	CP022779	1060140	1061316	family "IS5"	group "IS5"
SL3882	CP022779	1586239	1587415	family "IS5"	group "IS5"
SL3882	CP022779	1148155	1149335	family "IS5"	group "IS5"
SL3882	CP022779	99449	100628	family "IS5"	group "IS5"
SL3882	CP022779	1450028	1451207	family "IS5"	group "IS5"
SL3882	CP022779	1875728	1876904	family "IS5"	group "IS5"
SL3882	CP022779	209242	210372	family "IS5"	group "IS903"
SL3882	CP022779	591086	592216	family "IS5"	group "IS903"
SL3882	CP022779	607093	608223	family "IS5"	group "IS903"
SL3882	CP022779	1679815	1680945	family "IS5"	group "IS903"
SL3882	CP022779	614724	617127	family "IS110"	group "IS1111"
SL3882	CP022779	683961	686364	family "IS110"	group "IS1111"
SL3882	CP022779	545894	547275	family "IS110"	group "IS1111"
SL3882	CP022779	909077	910550	family "IS4"	group "IS50"

SL3882	CP022779	961536	963009	family "IS4"	group "IS50"
SL3882	CP022779	1439523	1440754	family "IS3"	group "IS51"
SL3882	CP022779	1660661	1661892	family "IS3"	group "IS51"
SL3882	CP022779	2032892	2034897	family "IS110"	group "IS1111"
SL3882	CP022779	2036281	2038282	family "IS110"	group "IS1111"
SL3882	CP022779	595088	597259	family "Tn3"	group ""
SL3882	CP022779	935419	936304	family "IS5"	group "-"
SL3882	CP022779	1247313	1248198	family "IS5"	group "-"
SL3882	CP022779	1686135	1687020	family "IS5"	group "-"
SL3882	CP022779	1429413	1430298	family "IS5"	group "-"
SL3882	CP022779	824334	825210	family "IS5"	group "-"
T42	CP022773	2179	3611	family "IS4"	group ""
T42	CP022773	468420	469852	family "IS4"	group ""
T42	CP022773	34787	35967	family "IS5"	group "IS5"
T42	CP022773	396352	397532	family "IS5"	group "IS5"
T42	CP022773	1284403	1285583	family "IS5"	group "IS5"
T42	CP022773	1535362	1536542	family "IS5"	group "IS5"
T42	CP022773	51042	52218	family "IS5"	group "IS5"
T42	CP022773	600012	601188	family "IS5"	group "IS5"
T42	CP022773	604365	605541	family "IS5"	group "IS5"
T42	CP022773	856121	857297	family "IS5"	group "IS5"
T42	CP022773	863024	864200	family "IS5"	group "IS5"
T42	CP022773	1055182	1056358	family "IS5"	group "IS5"
T42	CP022773	344384	345557	family "IS5"	group "IS5"
T42	CP022773	1573015	1574195	family "IS5"	group "IS5"
T42	CP022773	103970	105146	family "IS5"	group "IS5"
T42	CP022773	1148246	1149422	family "IS5"	group "IS5"
T42	CP022773	48224	49439	family "IS5"	group "IS5"
T42	CP022773	92888	94103	family "IS5"	group "IS5"
T42	CP022773	213627	214842	family "IS5"	group "IS5"
T42	CP022773	216564	217779	family "IS5"	group "IS5"
T42	CP022773	299316	300523	family "IS5"	group "IS5"
T42	CP022773	116049	117179	family "IS5"	group "IS903"
T42	CP022773	597968	599098	family "IS5"	group "IS903"
T42	CP022773	657372	658227	family "IS5"	group "IS427"
T42	CP022773	102040	102895	family "IS5"	group "IS427"
T42	CP022773	1591940	1592795	family "IS5"	group "IS427"
T42	CP022773	300524	301375	family "IS5"	group "IS427"
T42	CP022773	212972	213555	family "IS5"	group "IS427"
T42	CP022773	1137738	1138969	family "IS3"	group "IS51"
T42	CP022773	1358820	1360051	family "IS3"	group "IS51"
T42	CP022773	1731057	1733062	family "IS110"	group "IS1111"
T42	CP022773	1734446	1736447	family "IS110"	group "IS1111"
T42	CP022773	556182	557538	family "IS110"	group "IS1111"
T42	CP022773	1089345	1090238	family "IS5"	group "-"
T42	CP022773	654875	655760	family "IS5"	group "-"
T42	CP022773	1127628	1128513	family "IS5"	group "-"
SL3300	IS_5d5b941e	33344	34524	family "IS5"	group "IS5"
SL3300	IS_5d5b941e	1525942	1527122	family "IS5"	group "IS5"

SL3300	IS_5d5b941e	48387	49563	family "IS5"	group "IS5"
SL3300	IS_5d5b941e	653839	655015	family "IS5"	group "IS5"
SL3300	IS_5d5b941e	847226	848402	family "IS5"	group "IS5"
SL3300	IS_5d5b941e	1099294	1100470	family "IS5"	group "IS5"
SL3300	IS_5d5b941e	1291459	1292635	family "IS5"	group "IS5"
SL3300	IS_5d5b941e	1092391	1093567	family "IS5"	group "IS5"
SL3300	IS_5d5b941e	1619965	1621145	family "IS5"	group "IS5"
SL3300	IS_5d5b941e	1821489	1822669	family "IS5"	group "IS5"
SL3300	IS_5d5b941e	99326	100502	family "IS5"	group "IS5"
SL3300	IS_5d5b941e	1390021	1391197	family "IS5"	group "IS5"
SL3300	IS_5d5b941e	197934	199149	family "IS5"	group "IS5"
SL3300	IS_5d5b941e	212937	214152	family "IS5"	group "IS5"
SL3300	IS_5d5b941e	617982	619197	family "IS5"	group "IS5"
SL3300	IS_5d5b941e	619194	620409	family "IS5"	group "IS5"
SL3300	IS_5d5b941e	634398	635613	family "IS5"	group "IS5"
SL3300	IS_5d5b941e	1375535	1376750	family "IS5"	group "IS5"
SL3300	IS_5d5b941e	1661889	1663104	family "IS5"	group "IS5"
SL3300	IS_5d5b941e	1663101	1664316	family "IS5"	group "IS5"
SL3300	IS_5d5b941e	126932	128139	family "IS5"	group "IS5"
SL3300	IS_5d5b941e	214922	216129	family "IS5"	group "IS5"
SL3300	IS_5d5b941e	1664658	1665865	family "IS5"	group "IS5"
SL3300	IS_5d5b941e	1682236	1683443	family "IS5"	group "IS5"
SL3300	IS_5d5b941e	1842907	1844114	family "IS5"	group "IS5"
SL3300	IS_5d5b941e	658154	659361	family "IS5"	group "IS5"
SL3300	IS_5d5b941e	1665866	1666740	family "IS5"	group "IS5"
SL3300	IS_5d5b941e	590531	591916	family "IS110"	group "IS1111"
SL3300	IS_5d5b941e	650000	651380	family "IS110"	group "IS1111"
SL3300	IS_5d5b941e	914533	915388	family "IS5"	group "IS427"
SL3300	IS_5d5b941e	1600188	1601043	family "IS5"	group "IS427"
SL3300	IS_5d5b941e	1844115	1844966	family "IS5"	group "IS427"
SL3300	IS_5d5b941e	209557	210140	family "IS5"	group "IS427"
SL3300	IS_5d5b941e	1379513	1380744	family "IS3"	group "IS51"
SL3300	IS_5d5b941e	1601226	1602457	family "IS3"	group "IS51"
SL3300	IS_5d5b941e	1874054	1875540	family "IS4"	group "IS50"
SL3300	IS_5d5b941e	1976540	1978013	family "IS4"	group "IS50"
SL3300	IS_5d5b941e	1983710	1985715	family "IS110"	group "IS1111"
SL3300	IS_5d5b941e	1987099	1989100	family "IS110"	group "IS1111"
SL3300	IS_5d5b941e	1094141	1095703	family "ISNCY"	group "IS1202"
SL3300	IS_5d5b941e	1095608	1096283	family "None"	group "None"
SL3822	IS_5d5b941e	33344	34524	family "IS5"	group "IS5"
SL3822	IS_5d5b941e	1525942	1527122	family "IS5"	group "IS5"
SL3822	IS_5d5b941e	48387	49563	family "IS5"	group "IS5"
SL3822	IS_5d5b941e	653839	655015	family "IS5"	group "IS5"
SL3822	IS_5d5b941e	847226	848402	family "IS5"	group "IS5"
SL3822	IS_5d5b941e	1099294	1100470	family "IS5"	group "IS5"
SL3822	IS_5d5b941e	1291459	1292635	family "IS5"	group "IS5"
SL3822	IS_5d5b941e	1092391	1093567	family "IS5"	group "IS5"
SL3822	IS_5d5b941e	1619965	1621145	family "IS5"	group "IS5"
SL3822	IS_5d5b941e	1821489	1822669	family "IS5"	group "IS5"

SL3822	IS_5d5b941e	99326	100502	family "IS5"	group "IS5"
SL3822	IS_5d5b941e	1390021	1391197	family "IS5"	group "IS5"
SL3822	IS_5d5b941e	197934	199149	family "IS5"	group "IS5"
SL3822	IS_5d5b941e	212937	214152	family "IS5"	group "IS5"
SL3822	IS_5d5b941e	617982	619197	family "IS5"	group "IS5"
SL3822	IS_5d5b941e	619194	620409	family "IS5"	group "IS5"
SL3822	IS_5d5b941e	634398	635613	family "IS5"	group "IS5"
SL3822	IS_5d5b941e	1375535	1376750	family "IS5"	group "IS5"
SL3822	IS_5d5b941e	1661889	1663104	family "IS5"	group "IS5"
SL3822	IS_5d5b941e	1663101	1664316	family "IS5"	group "IS5"
SL3822	IS_5d5b941e	126932	128139	family "IS5"	group "IS5"
SL3822	IS_5d5b941e	214922	216129	family "IS5"	group "IS5"
SL3822	IS_5d5b941e	1664658	1665865	family "IS5"	group "IS5"
SL3822	IS_5d5b941e	1682236	1683443	family "IS5"	group "IS5"
SL3822	IS_5d5b941e	1842907	1844114	family "IS5"	group "IS5"
SL3822	IS_5d5b941e	658154	659361	family "IS5"	group "IS5"
SL3822	IS_5d5b941e	1665866	1666740	family "IS5"	group "IS5"
SL3822	IS_5d5b941e	590531	591916	family "IS110"	group "IS1111"
SL3822	IS_5d5b941e	650000	651380	family "IS110"	group "IS1111"
SL3822	IS_5d5b941e	914533	915388	family "IS5"	group "IS427"
SL3822	IS_5d5b941e	1600188	1601043	family "IS5"	group "IS427"
SL3822	IS_5d5b941e	1844115	1844966	family "IS5"	group "IS427"
SL3822	IS_5d5b941e	209557	210140	family "IS5"	group "IS427"
SL3822	IS_5d5b941e	1379513	1380744	family "IS3"	group "IS51"
SL3822	IS_5d5b941e	1601226	1602457	family "IS3"	group "IS51"
SL3822	IS_5d5b941e	1874054	1875540	family "IS4"	group "IS50"
SL3822	IS_5d5b941e	1976540	1978013	family "IS4"	group "IS50"
SL3822	IS_5d5b941e	1983710	1985715	family "IS110"	group "IS1111"
SL3822	IS_5d5b941e	1987099	1989100	family "IS110"	group "IS1111"
SL3822	IS_5d5b941e	1094141	1095703	family "ISNCY"	group "IS1202"
SL3822	IS_5d5b941e	1095608	1096283	family "None"	group "None"
HA4-1	CP022482	50848	52303	family "IS110"	group ""
HA4-1	CP022482	559734	561189	family "IS110"	group ""
HA4-1	CP022482	622820	623675	family "IS5"	group "IS427"
HA4-1	CP022482	1465534	1466389	family "IS5"	group "IS427"
HA4-1	CP022482	1047442	1048297	family "IS5"	group "IS427"
HA4-1	CP022482	1202438	1203293	family "IS5"	group "IS427"
HA4-1	CP022482	1399498	1400353	family "IS5"	group "IS427"
HA4-1	CP022482	221754	222609	family "IS5"	group "IS427"
HA4-1	CP022482	1197720	1198575	family "IS5"	group "IS427"
HA4-1	CP022482	884643	885227	family "IS5"	group "IS427"
HA4-1	CP022482	300142	301357	family "IS5"	group "IS5"
HA4-1	CP022482	511423	512638	family "IS5"	group "IS5"
HA4-1	CP022482	791476	792691	family "IS5"	group "IS5"
HA4-1	CP022482	880632	881847	family "IS5"	group "IS5"
HA4-1	CP022482	897000	898215	family "IS5"	group "IS5"
HA4-1	CP022482	898212	899427	family "IS5"	group "IS5"
HA4-1	CP022482	899424	900639	family "IS5"	group "IS5"
HA4-1	CP022482	1055659	1056874	family "IS5"	group "IS5"

HA4-1	CP022482	1223867	1225082	family "IS5"	group "IS5"
HA4-1	CP022482	1439580	1440795	family "IS5"	group "IS5"
HA4-1	CP022482	1784327	1785542	family "IS5"	group "IS5"
HA4-1	CP022482	467379	468756	family "IS110"	group "IS1111"
HA4-1	CP022482	893591	894963	family "IS110"	group "IS1111"
HA4-1	CP022482	483577	484462	family "IS5"	group "-"
HA4-1	CP022482	1201151	1202036	family "IS5"	group "-"
HA4-1	CP022482	1544248	1545133	family "IS5"	group "-"
HA4-1	CP022482	1132555	1133262	family "IS3"	group "IS407"
HA4-1	CP022482	1134646	1135349	family "IS3"	group "IS407"
HA4-1	CP022482	1346905	1348035	family "IS5"	group "IS903"
HA4-1	CP022482	1844042	1845172	family "IS5"	group "IS903"
HA4-1	CP022482	624777	626215	family "IS4"	group ""
HA4-1	CP022482	1840036	1841484	family "IS3"	group "IS150"
HA4-1	CP022482	1944122	1945684	family "ISNCY"	group "IS1202"
HA4-1	CP022482	1945589	1946263	family "None"	group "None"
pHA4-1	CP022483	96171	98732	family "IS66"	group ""
pHA4-1	CP022483	114938	117499	family "IS66"	group ""
pHA4-1	CP022483	137482	138355	family "IS5"	group "IS427"
KACC10709	CP016905	123195	124280	family "IS1595"	group "ISSod11"
KACC10709	CP016905	314066	315151	family "IS1595"	group "ISSod11"
KACC10709	CP016905	1617452	1618537	family "IS1595"	group "ISSod11"
KACC10709	CP016905	173839	174694	family "IS5"	group "IS427"
KACC10709	CP016905	178547	179402	family "IS5"	group "IS427"
KACC10709	CP016905	569984	570839	family "IS5"	group "IS427"
KACC10709	CP016905	1162635	1163490	family "IS5"	group "IS427"
KACC10709	CP016905	1829692	1830547	family "IS5"	group "IS427"
KACC10709	CP016905	431638	432493	family "IS5"	group "IS427"
KACC10709	CP016905	872683	873538	family "IS5"	group "IS427"
KACC10709	CP016905	1348589	1349444	family "IS5"	group "IS427"
KACC10709	CP016905	1781630	1782485	family "IS5"	group "IS427"
KACC10709	CP016905	894588	895171	family "IS5"	group "IS427"
KACC10709	CP016905	175688	176867	family "IS5"	group "IS5"
KACC10709	CP016905	429875	431054	family "IS5"	group "IS5"
KACC10709	CP016905	1166285	1167464	family "IS5"	group "IS5"
KACC10709	CP016905	1561138	1562317	family "IS5"	group "IS5"
KACC10709	CP016905	1844542	1845721	family "IS5"	group "IS5"
KACC10709	CP016905	388807	389983	family "IS5"	group "IS5"
KACC10709	CP016905	1281034	1282210	family "IS5"	group "IS5"
KACC10709	CP016905	1378884	1380060	family "IS5"	group "IS5"
KACC10709	CP016905	1549389	1550565	family "IS5"	group "IS5"
KACC10709	CP016905	1620602	1621778	family "IS5"	group "IS5"
KACC10709	CP016905	427524	428699	family "IS5"	group "IS5"
KACC10709	CP016905	683036	684215	family "IS5"	group "IS5"
KACC10709	CP016905	572349	573522	family "IS5"	group "IS5"
KACC10709	CP016905	718784	719963	family "IS5"	group "IS5"
KACC10709	CP016905	721139	722314	family "IS5"	group "IS5"

KACC10709	CP016905	1868058	1869234	family "IS5"	group "IS5"
KACC10709	CP016905	875837	877010	family "IS5"	group "IS5"
KACC10709	CP016905	882098	882815	family "IS5"	group "IS5"
KACC10709	CP016905	724265	726818	family "IS66"	group ""
KACC10709	CP016905	983468	986021	family "IS66"	group ""
KACC10709	CP016905	1628474	1631027	family "IS66"	group ""
KACC10709	CP016905	432948	435500	family "IS66"	group ""
KACC10709	CP016905	1688116	1690668	family "IS66"	group ""
KACC10709	CP016905	310456	311927	family "IS66"	group ""
KACC10709	CP016905	868734	870205	family "IS66"	group ""
KACC10709	CP016905	1495745	1497216	family "IS66"	group ""
KACC10709	CP016905	601977	602862	family "IS5"	group "-"
KACC10709	CP016905	179492	180377	family "IS5"	group "-"
KACC10709	CP016905	571024	571909	family "IS5"	group "-"
KACC10709	CP016905	881214	882099	family "IS5"	group "-"
KACC10709	CP016905	1377549	1378434	family "IS5"	group "-"
KACC10709	CP016905	1453461	1454346	family "IS5"	group "-"
KACC10709	CP016905	1651296	1652181	family "IS5"	group "-"
KACC10709	CP016905	2063712	2064597	family "IS5"	group "-"
KACC10709	CP016905	288338	289214	family "IS5"	group "-"
KACC10709	CP016905	677544	679223	family "IS1182"	group ""
KACC10709	CP016905	941432	943109	family "IS1182"	group ""
KACC10709	CP016905	1563918	1565489	family "IS1182"	group ""
KACC10709	CP016905	681308	682522	family "IS5"	group "IS5"
KACC10709	CP016905	1578535	1579741	family "IS5"	group "IS5"
KACC10709	CP016905	1892046	1893252	family "IS5"	group "IS5"
KACC10709	CP016905	879541	880747	family "IS5"	group "IS5"
KACC10709	CP016905	1022941	1024147	family "IS5"	group "IS5"
KACC10709	CP016905	1127775	1128981	family "IS5"	group "IS5"
KACC10709	CP016905	1167982	1169188	family "IS5"	group "IS5"
KACC10709	CP016905	1526293	1527499	family "IS5"	group "IS5"
KACC10709	CP016905	884085	886233	family "IS110"	group "IS1111"
KACC10709	CP016905	1545628	1547085	family "IS110"	group "IS1111"
KACC10709	CP016905	1631027	1632281	family "IS110"	group "IS1111"
KACC10709	CP016905	1544937	1545562	family "IS110"	group "IS1111"
KACC10709	CP016905	981223	983228	family "IS110"	group "IS1111"
KACC10709	CP016905	987172	989173	family "IS110"	group "IS1111"
KACC10709	CP016905	1016625	1018073	family "IS3"	group "IS150"
KACC10709	CP016905	1935531	1936979	family "IS3"	group "IS150"
KACC10709	CP016905	1582590	1583812	family "IS3"	group "IS51"
KACC10709	CP016905	1780409	1781623	family "IS3"	group "IS51"
KACC10709	CP016905	1699352	1700566	family "IS3"	group "IS51"
KACC10709	CP016905	871512	872676	family "IS3"	group "IS51"
KACC10709	CP016905	2011650	2016363	family "Tn3"	group ""
KACC10709	CP016905	2139068	2143781	family "Tn3"	group ""
KACC10709	CP016905	357319	358449	family "IS5"	group "IS903"
KACC10709	CP016905	576685	577815	family "IS5"	group "IS903"
KACC10709	CP016905	707302	708432	family "IS5"	group "IS903"
KACC10709	CP016905	780799	781929	family "IS5"	group "IS903"

KACC10709	CP016905	856504	857634	family "IS5"	group "IS903"
KACC10709	CP016905	1052925	1054055	family "IS5"	group "IS903"
KACC10709	CP016905	1085771	1086901	family "IS5"	group "IS903"
KACC10709	CP016905	1624941	1626071	family "IS5"	group "IS903"
KACC10709	CP016905	2132223	2133353	family "IS5"	group "IS903"
KACC10709	CP016905	211362	212492	family "IS5"	group "IS903"
KACC10709	CP016905	386772	387902	family "IS5"	group "IS903"
KACC10709	CP016905	778600	779730	family "IS5"	group "IS903"
KACC10709	CP016905	896165	897295	family "IS5"	group "IS903"
KACC10709	CP016905	917214	918344	family "IS5"	group "IS903"
KACC10709	CP016905	1051625	1052755	family "IS5"	group "IS903"
KACC10709	CP016905	1134148	1135278	family "IS5"	group "IS903"
KACC10709	CP016905	1199710	1200840	family "IS5"	group "IS903"
KACC10709	CP016905	1562787	1563917	family "IS5"	group "IS903"
KACC10709	CP016905	1673967	1675097	family "IS5"	group "IS903"
KACC10709	CP016905	1683872	1685002	family "IS5"	group "IS903"
KACC10709	CP016905	1869397	1870527	family "IS5"	group "IS903"
KACC10709	CP016905	1991862	1992992	family "IS5"	group "IS903"
KACC10709	CP016905	2113465	2114595	family "IS5"	group "IS903"
KACC10709	CP016905	1347181	1348311	family "IS5"	group "IS903"
KACC10709	CP016905	1524728	1525858	family "IS5"	group "IS903"
KACC10709	CP016905	581526	582656	family "IS5"	group "IS903"
KACC10709	CP016905	899454	900584	family "IS5"	group "IS903"
KACC10709	CP016905	1163495	1164614	family "IS5"	group "IS903"
KACC10709	CP016905	177424	178542	family "IS5"	group "IS903"
KACC10709	CP016905	568861	569979	family "IS5"	group "IS903"
KACC10709	CP016905	916323	917213	family "IS5"	group "IS903"
KACC10709	CP016905	1450929	1452361	family "IS4"	group ""
CMR15	CP016905	123195	124280	family "IS1595"	group "ISSod11"
CMR15	CP016905	314066	315151	family "IS1595"	group "ISSod11"
CMR15	CP016905	1617452	1618537	family "IS1595"	group "ISSod11"
CMR15	CP016905	173839	174694	family "IS5"	group "IS427"
CMR15	CP016905	178547	179402	family "IS5"	group "IS427"
CMR15	CP016905	569984	570839	family "IS5"	group "IS427"
CMR15	CP016905	1162635	1163490	family "IS5"	group "IS427"
CMR15	CP016905	1829692	1830547	family "IS5"	group "IS427"
CMR15	CP016905	431638	432493	family "IS5"	group "IS427"
CMR15	CP016905	872683	873538	family "IS5"	group "IS427"
CMR15	CP016905	1348589	1349444	family "IS5"	group "IS427"
CMR15	CP016905	1781630	1782485	family "IS5"	group "IS427"
CMR15	CP016905	894588	895171	family "IS5"	group "IS427"
CMR15	CP016905	175688	176867	family "IS5"	group "IS5"
CMR15	CP016905	429875	431054	family "IS5"	group "IS5"
CMR15	CP016905	1166285	1167464	family "IS5"	group "IS5"
CMR15	CP016905	1561138	1562317	family "IS5"	group "IS5"
CMR15	CP016905	1844542	1845721	family "IS5"	group "IS5"
CMR15	CP016905	388807	389983	family "IS5"	group "IS5"
CMR15	CP016905	1281034	1282210	family "IS5"	group "IS5"

CMR15	CP016905	1378884	1380060	family "IS5"	group "IS5"
CMR15	CP016905	1549389	1550565	family "IS5"	group "IS5"
CMR15	CP016905	1620602	1621778	family "IS5"	group "IS5"
CMR15	CP016905	427524	428699	family "IS5"	group "IS5"
CMR15	CP016905	683036	684215	family "IS5"	group "IS5"
CMR15	CP016905	572349	573522	family "IS5"	group "IS5"
CMR15	CP016905	718784	719963	family "IS5"	group "IS5"
CMR15	CP016905	721139	722314	family "IS5"	group "IS5"
CMR15	CP016905	1868058	1869234	family "IS5"	group "IS5"
CMR15	CP016905	875837	877010	family "IS5"	group "IS5"
CMR15	CP016905	882098	882815	family "IS5"	group "IS5"
CMR15	CP016905	724265	726818	family "IS66"	group ""
CMR15	CP016905	983468	986021	family "IS66"	group ""
CMR15	CP016905	1628474	1631027	family "IS66"	group ""
CMR15	CP016905	432948	435500	family "IS66"	group ""
CMR15	CP016905	1688116	1690668	family "IS66"	group ""
CMR15	CP016905	310456	311927	family "IS66"	group ""
CMR15	CP016905	868734	870205	family "IS66"	group ""
CMR15	CP016905	1495745	1497216	family "IS66"	group ""
CMR15	CP016905	601977	602862	family "IS5"	group "-"
CMR15	CP016905	179492	180377	family "IS5"	group "-"
CMR15	CP016905	571024	571909	family "IS5"	group "-"
CMR15	CP016905	881214	882099	family "IS5"	group "-"
CMR15	CP016905	1377549	1378434	family "IS5"	group "-"
CMR15	CP016905	1453461	1454346	family "IS5"	group "-"
CMR15	CP016905	1651296	1652181	family "IS5"	group "-"
CMR15	CP016905	2063712	2064597	family "IS5"	group "-"
CMR15	CP016905	288338	289214	family "IS5"	group "-"
CMR15	CP016905	677544	679223	family "IS1182"	group ""
CMR15	CP016905	941432	943109	family "IS1182"	group ""
CMR15	CP016905	1563918	1565489	family "IS1182"	group ""
CMR15	CP016905	681308	682522	family "IS5"	group "IS5"
CMR15	CP016905	1578535	1579741	family "IS5"	group "IS5"
CMR15	CP016905	1892046	1893252	family "IS5"	group "IS5"
CMR15	CP016905	879541	880747	family "IS5"	group "IS5"
CMR15	CP016905	1022941	1024147	family "IS5"	group "IS5"
CMR15	CP016905	1127775	1128981	family "IS5"	group "IS5"
CMR15	CP016905	1167982	1169188	family "IS5"	group "IS5"
CMR15	CP016905	1526293	1527499	family "IS5"	group "IS5"
CMR15	CP016905	884085	886233	family "IS110"	group "IS1111"
CMR15	CP016905	1545628	1547085	family "IS110"	group "IS1111"
CMR15	CP016905	1631027	1632281	family "IS110"	group "IS1111"
CMR15	CP016905	1544937	1545562	family "IS110"	group "IS1111"
CMR15	CP016905	981223	983228	family "IS110"	group "IS1111"
CMR15	CP016905	987172	989173	family "IS110"	group "IS1111"
CMR15	CP016905	1016625	1018073	family "IS3"	group "IS150"
CMR15	CP016905	1935531	1936979	family "IS3"	group "IS150"
CMR15	CP016905	1582590	1583812	family "IS3"	group "IS51"
CMR15	CP016905	1780409	1781623	family "IS3"	group "IS51"

CMR15	CP016905	1699352	1700566	family "IS3"	group "IS51"
CMR15	CP016905	871512	872676	family "IS3"	group "IS51"
CMR15	CP016905	2011650	2016363	family "Tn3"	group ""
CMR15	CP016905	2139068	2143781	family "Tn3"	group ""
CMR15	CP016905	357319	358449	family "IS5"	group "IS903"
CMR15	CP016905	576685	577815	family "IS5"	group "IS903"
CMR15	CP016905	707302	708432	family "IS5"	group "IS903"
CMR15	CP016905	780799	781929	family "IS5"	group "IS903"
CMR15	CP016905	856504	857634	family "IS5"	group "IS903"
CMR15	CP016905	1052925	1054055	family "IS5"	group "IS903"
CMR15	CP016905	1085771	1086901	family "IS5"	group "IS903"
CMR15	CP016905	1624941	1626071	family "IS5"	group "IS903"
CMR15	CP016905	2132223	2133353	family "IS5"	group "IS903"
CMR15	CP016905	211362	212492	family "IS5"	group "IS903"
CMR15	CP016905	386772	387902	family "IS5"	group "IS903"
CMR15	CP016905	778600	779730	family "IS5"	group "IS903"
CMR15	CP016905	896165	897295	family "IS5"	group "IS903"
CMR15	CP016905	917214	918344	family "IS5"	group "IS903"
CMR15	CP016905	1051625	1052755	family "IS5"	group "IS903"
CMR15	CP016905	1134148	1135278	family "IS5"	group "IS903"
CMR15	CP016905	1199710	1200840	family "IS5"	group "IS903"
CMR15	CP016905	1562787	1563917	family "IS5"	group "IS903"
CMR15	CP016905	1673967	1675097	family "IS5"	group "IS903"
CMR15	CP016905	1683872	1685002	family "IS5"	group "IS903"
CMR15	CP016905	1869397	1870527	family "IS5"	group "IS903"
CMR15	CP016905	1991862	1992992	family "IS5"	group "IS903"
CMR15	CP016905	2113465	2114595	family "IS5"	group "IS903"
CMR15	CP016905	1347181	1348311	family "IS5"	group "IS903"
CMR15	CP016905	1524728	1525858	family "IS5"	group "IS903"
CMR15	CP016905	581526	582656	family "IS5"	group "IS903"
CMR15	CP016905	899454	900584	family "IS5"	group "IS903"
CMR15	CP016905	1163495	1164614	family "IS5"	group "IS903"
CMR15	CP016905	177424	178542	family "IS5"	group "IS903"
CMR15	CP016905	568861	569979	family "IS5"	group "IS903"
CMR15	CP016905	916323	917213	family "IS5"	group "IS903"
CMR15	CP016905	1450929	1452361	family "IS4"	group ""
RS476	CP021763	45841	46696	family "IS5"	group "IS427"
RS476	CP021763	716957	717812	family "IS5"	group "IS427"
RS476	CP021763	1245108	1245963	family "IS5"	group "IS427"
RS476	CP021763	101432	102287	family "IS5"	group "IS427"
RS476	CP021763	395525	396380	family "IS5"	group "IS427"
RS476	CP021763	267324	267907	family "IS5"	group "IS427"
RS476	CP021763	179037	180527	family "IS5"	group "IS5"
RS476	CP021763	720090	721574	family "IS5"	group "IS5"
RS476	CP021763	254510	255725	family "IS5"	group "IS5"
RS476	CP021763	1486416	1487631	family "IS5"	group "IS5"
RS476	CP021763	269704	270870	family "IS630"	group "-"
RS476	CP021763	2087309	2088475	family "IS630"	group "-"
RS476	CP021763	1451402	1452568	family "IS630"	group "-"
RS476	CP021763	564892	565529	family "IS630"	group "-"

RS476	CP021763	562294	562829	family "IS630"	group "-"
RS476	CP021763	562834	564851	family "IS21"	group "-"
RS476	CP021763	1196918	1198935	family "IS21"	group "-"
RS476	CP021763	1448742	1450218	family "IS4"	group "IS4"
RS476	CP021763	1648964	1650440	family "IS4"	group "IS4"
RS476	CP021763	581771	583239	family "IS4"	group "IS4"
RS476	CP021763	1650962	1655645	family "IS5"	group "-"
RS476	CP021763	1681152	1685835	family "IS5"	group "-"
RS476	CP021763	1711342	1712341	family "IS5"	group "-"
RS476	CP021763	145864	146815	family "IS5"	group "-"
RS476	CP021763	590366	591317	family "IS5"	group "-"
RS476	CP021763	1428966	1429917	family "IS5"	group "-"
RS476	CP021763	147338	148223	family "IS5"	group "-"
RS476	CP021763	625433	626320	family "IS5"	group "-"
RS476	CP021763	180524	181380	family "IS5"	group "-"
RS476	CP021763	104002	104862	family "IS5"	group "-"
RS476	CP021763	96043	96917	family "IS5"	group "-"
RS476	CP021763	597122	598011	family "IS5"	group "-"
RS476	CP021763	598795	600128	family "IS256"	group "-"
RS476	CP021763	687753	689007	family "IS3"	group "-"
RS476	CP021763	699344	700581	family "IS3"	group "IS407"
RS476	CP021763	2048570	2049337	family "IS3"	group "IS407"
CRMRs218	No IS(s) found using OASIS Predictor...				
YC40M	CP015851	231842	233023	family "IS5"	group "IS5"
YC40M	CP015851	335887	337068	family "IS5"	group "IS5"
YC40M	CP015851	453314	454495	family "IS5"	group "IS5"
YC40M	CP015851	761163	762344	family "IS5"	group "IS5"
YC40M	CP015851	1387820	1389001	family "IS5"	group "IS5"
YC40M	CP015851	607910	609089	family "IS5"	group "IS5"
YC40M	CP015851	1034715	1035890	family "IS5"	group "IS5"
YC40M	CP015851	1812930	1814111	family "IS5"	group "IS5"
YC40M	CP015851	538107	539261	family "IS5"	group "IS5"
YC40M	CP015851	1590914	1592068	family "IS5"	group "IS5"
YC40M	CP015851	1564624	1565777	family "IS5"	group "IS5"
YC40M	CP015851	799947	801101	family "IS5"	group "IS5"
YC40M	CP015851	1436562	1437713	family "IS5"	group "IS5"
YC40M	CP015851	648401	649555	family "IS5"	group "IS5"
YC40M	CP015851	1258134	1259284	family "IS5"	group "IS5"
YC40M	CP015851	1386607	1387762	family "IS5"	group "IS5"
YC40M	CP015851	801275	802430	family "IS5"	group "IS5"
YC40M	CP015851	290359	291513	family "IS5"	group "IS5"
YC40M	CP015851	1906149	1907303	family "IS5"	group "IS5"
YC40M	CP015851	559361	560514	family "IS5"	group "IS5"
YC40M	CP015851	809880	811034	family "IS5"	group "IS5"
YC40M	CP015851	346855	347710	family "IS5"	group "IS427"
YC40M	CP015851	1454540	1455395	family "IS5"	group "IS427"
YC40M	CP015851	1604761	1605616	family "IS5"	group "IS427"
YC40M	CP015851	1024900	1025751	family "IS5"	group "IS427"

YC40M	CP015851	1260110	1260965	family "IS5"	group "IS427"
YC40M	CP015851	1573831	1574686	family "IS5"	group "IS427"
YC40M	CP015851	182341	182924	family "IS5"	group "IS427"
YC40M	CP015851	424345	425802	family "IS110"	group ""
YC40M	CP015851	1667671	1669128	family "IS110"	group ""
YC40M	CP015851	1793876	1795330	family "IS110"	group ""
YC40M	CP015851	444654	445362	family "IS3"	group "IS407"
YC40M	CP015851	446746	447450	family "IS3"	group "IS407"
YC40M	CP015851	1566754	1569309	family "IS66"	group ""
YC40M	CP015851	642822	645373	family "IS66"	group ""
YC40M	CP015851	1878759	1881310	family "IS66"	group ""
YC40M	CP015851	1023488	1024905	family "IS110"	group "IS1111"
YC40M	CP015851	1554041	1555458	family "IS110"	group "IS1111"
YC40M	CP015851	1255822	1257283	family "IS4"	group "IS50"
YC40M	CP015851	1844961	1846416	family "IS4"	group "IS50"
YC40M	CP015851	1572159	1573536	family "IS110"	group "IS1111"
YC40M	CP015851	1633244	1634616	family "IS110"	group "IS1111"
YC40M	CP015851	1722451	1723889	family "IS4"	group ""
YC40M	CP015851	1818337	1819066	family "IS5"	group "IS427"
YC40M	CP015851	1834322	1835474	family "IS3"	group "IS407"
SN82F48	IS_de47452f	344505	345952	family "ISL3"	group ""
SN82F48	IS_de47452f	606538	607985	family "ISL3"	group ""
SN82F48	IS_de47452f	1180278	1181725	family "ISL3"	group ""
SN82F48	IS_de47452f	1116243	1117690	family "ISL3"	group ""
SN82F48	IS_de47452f	1253883	1255272	family "IS256"	group ""
SN82F48	IS_de47452f	1289169	1290558	family "IS256"	group ""
SN82F48	IS_de47452f	1329322	1330711	family "IS256"	group ""
SN83A39	IS_c6bb695b	939083	944123	family "Tn3"	group ""
SN83A39	IS_c6bb695b	1031594	1032117	family "Tn3"	group ""
SN83A39	IS_c6bb695b	1047378	1048925	family "ISNCY"	group "IS1202"
SEPPX05	IS_12c9bc1e	33226	35112	family "IS21"	group "-"
SEPPX05	IS_12c9bc1e	141876	143762	family "IS21"	group "-"
SEPPX05	IS_12c9bc1e	362789	364675	family "IS21"	group "-"
SEPPX05	IS_12c9bc1e	462144	464030	family "IS21"	group "-"
SEPPX05	IS_12c9bc1e	1060663	1062549	family "IS21"	group "-"
SEPPX05	IS_12c9bc1e	40144	42030	family "IS21"	group "-"
SEPPX05	IS_12c9bc1e	804410	806296	family "IS21"	group "-"
SEPPX05	IS_12c9bc1e	838815	840701	family "IS21"	group "-"
SEPPX05	IS_12c9bc1e	904911	906797	family "IS21"	group "-"
SEPPX05	IS_12c9bc1e	1067362	1069248	family "IS21"	group "-"
SEPPX05	IS_12c9bc1e	1264510	1266396	family "IS21"	group "-"
SEPPX05	IS_12c9bc1e	1364089	1365975	family "IS21"	group "-"
SEPPX05	IS_12c9bc1e	1534844	1536730	family "IS21"	group "-"
SEPPX05	IS_12c9bc1e	1750680	1752566	family "IS21"	group "-"
SEPPX05	IS_12c9bc1e	1875675	1877561	family "IS21"	group "-"
SEPPX05	IS_12c9bc1e	2020040	2021926	family "IS21"	group "-"
SEPPX05	IS_12c9bc1e	177368	178565	family "IS21"	group "-"

SEPPX05	IS_12c9bc1e	181250	182015	family "IS21"	group "-"
SEPPX05	IS_12c9bc1e	173454	174319	family "IS5"	group "IS427"
SEPPX05	IS_12c9bc1e	595907	596772	family "IS5"	group "IS427"
SEPPX05	IS_12c9bc1e	1129753	1130618	family "IS5"	group "IS427"
SEPPX05	IS_12c9bc1e	1936839	1937704	family "IS5"	group "IS427"
SEPPX05	IS_12c9bc1e	553718	554583	family "IS5"	group "IS427"
SEPPX05	IS_12c9bc1e	779236	780101	family "IS5"	group "IS427"
SEPPX05	IS_12c9bc1e	2040643	2041508	family "IS5"	group "IS427"
SEPPX05	IS_12c9bc1e	42991	43846	family "IS5"	group "IS427"
SEPPX05	IS_12c9bc1e	1317455	1318310	family "IS5"	group "IS427"
SEPPX05	IS_12c9bc1e	361000	361865	family "IS5"	group "IS427"
SEPPX05	IS_12c9bc1e	441089	441954	family "IS5"	group "IS427"
SEPPX05	IS_12c9bc1e	309906	310761	family "IS5"	group "IS427"
SEPPX05	IS_12c9bc1e	739122	739977	family "IS5"	group "IS427"
SEPPX05	IS_12c9bc1e	2024255	2025110	family "IS5"	group "IS427"
SEPPX05	IS_12c9bc1e	54355	55666	family "IS3"	group "IS2"
SEPPX05	IS_12c9bc1e	99799	101110	family "IS3"	group "IS2"
SEPPX05	IS_12c9bc1e	271848	273159	family "IS3"	group "IS2"
SEPPX05	IS_12c9bc1e	367027	368338	family "IS3"	group "IS2"
SEPPX05	IS_12c9bc1e	585417	586728	family "IS3"	group "IS2"
SEPPX05	IS_12c9bc1e	801431	802742	family "IS3"	group "IS2"
SEPPX05	IS_12c9bc1e	1376997	1378308	family "IS3"	group "IS2"
SEPPX05	IS_12c9bc1e	1538296	1539607	family "IS3"	group "IS2"
SEPPX05	IS_12c9bc1e	906827	908076	family "IS3"	group "IS2"
SEPPX05	IS_12c9bc1e	26142	27421	family "IS3"	group "IS2"
SEPPX05	IS_12c9bc1e	1962311	1963590	family "IS3"	group "IS2"
SEPPX05	IS_12c9bc1e	168405	170578	family "IS5"	group "IS5"
SEPPX05	IS_12c9bc1e	806337	808510	family "IS5"	group "IS5"
SEPPX05	IS_12c9bc1e	1308746	1310919	family "IS5"	group "IS5"
SEPPX05	IS_12c9bc1e	1361875	1364048	family "IS5"	group "IS5"
SEPPX05	IS_12c9bc1e	908790	910959	family "IS5"	group "IS5"
SEPPX05	IS_12c9bc1e	30686	31289	family "IS5"	group "IS5"
SEPPX05	IS_12c9bc1e	1879728	1880331	family "IS5"	group "IS5"
SEPPX05	IS_12c9bc1e	339446	340311	family "IS5"	group "IS427"
SEPPX05	IS_12c9bc1e	1179332	1180197	family "IS5"	group "IS427"
SEPPX05	IS_12c9bc1e	175051	175916	family "IS5"	group "IS427"
SEPPX05	IS_12c9bc1e	2039776	2040641	family "IS5"	group "IS427"
SEPPX05	IS_12c9bc1e	1961421	1962259	family "IS5"	group "IS427"
SEPPX05	IS_12c9bc1e	178576	181191	family "IS21"	group ""
SEPPX05	IS_12c9bc1e	582031	584646	family "IS21"	group ""
SEPPX05	IS_12c9bc1e	781599	784214	family "IS21"	group ""
SEPPX05	IS_12c9bc1e	1062810	1065425	family "IS21"	group ""
SEPPX05	IS_12c9bc1e	1544891	1547506	family "IS21"	group ""
SEPPX05	IS_12c9bc1e	206035	207244	family "IS3"	group "IS150"
SEPPX05	IS_12c9bc1e	483702	484911	family "IS3"	group "IS150"
SEPPX05	IS_12c9bc1e	870545	871754	family "IS3"	group "IS150"
SEPPX05	IS_12c9bc1e	903451	904660	family "IS3"	group "IS150"
SEPPX05	IS_12c9bc1e	1964841	1966050	family "IS3"	group "IS150"
SEPPX05	IS_12c9bc1e	1864632	1865841	family "IS3"	group "IS150"
SEPPX05	IS_12c9bc1e	262481	263951	family "IS701"	group "-"
SEPPX05	IS_12c9bc1e	458782	460252	family "IS701"	group "-"

SEPPX05	IS_12c9bc1e	784519	785989	family "IS701"	group "-"
SEPPX05	IS_12c9bc1e	594516	595908	family "IS701"	group "-"
SEPPX05	IS_12c9bc1e	1146200	1147503	family "ISL3"	group "-"
SEPPX05	IS_12c9bc1e	1886950	1888253	family "ISL3"	group "-"
SEPPX05	IS_12c9bc1e	2045028	2046331	family "ISL3"	group "-"
SEPPX05	IS_12c9bc1e	1936100	1936763	family "IS3"	group "IS407"
SEPPX05	IS_12c9bc1e	1939103	1939762	family "IS3"	group "IS407"
SEPPX05	IS_12c9bc1e	783985	784752	family "IS5"	group "-"
Rs_10_244	CM002756_1	705702	707024	family "IS3"	group "IS2"
Rs-09-161	CM002758	44161	45631	family "IS701"	group "-"
Rs-09-161	CM002758	52289	53460	family "IS5"	group "IS5"
Rs-09-161	CM002758	74810	76274	family "IS4"	group "IS4"
Rs-09-161	CM002758	87450	88120	family "IS3"	group "IS407"
SL3103	CP022791	15125	16255	family "IS5"	group "IS903"
SL3103	CP022791	35981	37111	family "IS5"	group "IS903"
SL3103	CP022791	64450	65580	family "IS5"	group "IS903"
SL3103	CP022791	99465	100595	family "IS5"	group "IS903"
SL3103	CP022791	246926	248056	family "IS5"	group "IS903"
SL3103	CP022791	452848	453978	family "IS5"	group "IS903"
SL3103	CP022791	500998	502128	family "IS5"	group "IS903"
SL3103	CP022791	528961	530091	family "IS5"	group "IS903"
SL3103	CP022791	538856	539986	family "IS5"	group "IS903"
SL3103	CP022791	568374	569504	family "IS5"	group "IS903"
SL3103	CP022791	728118	729248	family "IS5"	group "IS903"
SL3103	CP022791	1042321	1043451	family "IS5"	group "IS903"
SL3103	CP022791	1061078	1062208	family "IS5"	group "IS903"
SL3103	CP022791	1217767	1218897	family "IS5"	group "IS903"
SL3103	CP022791	1530637	1531767	family "IS5"	group "IS903"
SL3103	CP022791	1628035	1629165	family "IS5"	group "IS903"
SL3103	CP022791	1632876	1634006	family "IS5"	group "IS903"
SL3103	CP022791	1724285	1725415	family "IS5"	group "IS903"
SL3103	CP022791	1748523	1749653	family "IS5"	group "IS903"
SL3103	CP022791	1820388	1821518	family "IS5"	group "IS903"
SL3103	CP022791	1822587	1823717	family "IS5"	group "IS903"
SL3103	CP022791	1962250	1963380	family "IS5"	group "IS903"
SL3103	CP022791	1979119	1980249	family "IS5"	group "IS903"
SL3103	CP022791	1982529	1983659	family "IS5"	group "IS903"
SL3103	CP022791	2015374	2016504	family "IS5"	group "IS903"
SL3103	CP022791	2016674	2017804	family "IS5"	group "IS903"
SL3103	CP022791	422705	423835	family "IS5"	group "IS903"
SL3103	CP022791	1898290	1899420	family "IS5"	group "IS903"
SL3103	CP022791	761188	762318	family "IS5"	group "IS903"
SL3103	CP022791	1092781	1093910	family "IS5"	group "IS903"
SL3103	CP022791	1961359	1962249	family "IS5"	group "IS903"
SL3103	CP022791	26187	28335	family "IS110"	group "IS1111"
SL3103	CP022791	442394	443851	family "IS110"	group "IS1111"
SL3103	CP022791	458934	460188	family "IS110"	group "IS1111"
SL3103	CP022791	441703	442328	family "IS110"	group "IS1111"

SL3103	CP022791	29604	30818	family "IS5"	group "IS5"
SL3103	CP022791	502596	503810	family "IS5"	group "IS5"
SL3103	CP022791	505010	506224	family "IS5"	group "IS5"
SL3103	CP022791	1380714	1381928	family "IS5"	group "IS5"
SL3103	CP022791	1767498	1768712	family "IS5"	group "IS5"
SL3103	CP022791	1919277	1920491	family "IS5"	group "IS5"
SL3103	CP022791	1980980	1982194	family "IS5"	group "IS5"
SL3103	CP022791	1195042	1196248	family "IS5"	group "IS5"
SL3103	CP022791	1723087	1724290	family "IS5"	group "IS5"
SL3103	CP022791	67255	68431	family "IS5"	group "IS5"
SL3103	CP022791	180786	181962	family "IS5"	group "IS5"
SL3103	CP022791	277761	278937	family "IS5"	group "IS5"
SL3103	CP022791	446155	447331	family "IS5"	group "IS5"
SL3103	CP022791	503808	504984	family "IS5"	group "IS5"
SL3103	CP022791	806902	808078	family "IS5"	group "IS5"
SL3103	CP022791	1760006	1761182	family "IS5"	group "IS5"
SL3103	CP022791	1762363	1763539	family "IS5"	group "IS5"
SL3103	CP022791	448512	449685	family "IS5"	group "IS5"
SL3103	CP022791	1532672	1533845	family "IS5"	group "IS5"
SL3103	CP022791	1571388	1572561	family "IS5"	group "IS5"
SL3103	CP022791	1219060	1220236	family "IS5"	group "IS5"
SL3103	CP022791	765611	766784	family "IS5"	group "IS5"
SL3103	CP022791	760232	761117	family "IS5"	group "-"
SL3103	CP022791	1089518	1090403	family "IS5"	group "-"
SL3103	CP022791	1125853	1126738	family "IS5"	group "-"
SL3103	CP022791	1306575	1307460	family "IS5"	group "-"
SL3103	CP022791	992570	993455	family "IS5"	group "-"
SL3103	CP022791	276426	277311	family "IS5"	group "-"
SL3103	CP022791	648459	649344	family "IS5"	group "-"
SL3103	CP022791	763808	764693	family "IS5"	group "-"
SL3103	CP022791	1311019	1311904	family "IS5"	group "-"
SL3103	CP022791	651399	652275	family "IS5"	group "-"
SL3103	CP022791	349810	351240	family "IS4"	group ""
SL3103	CP022791	1290367	1291797	family "IS4"	group ""
SL3103	CP022791	456381	458934	family "IS66"	group ""
SL3103	CP022791	1764844	1767397	family "IS66"	group ""
SL3103	CP022791	523294	525847	family "IS66"	group ""
SL3103	CP022791	2082928	2085481	family "IS66"	group ""
SL3103	CP022791	1398281	1400832	family "IS66"	group ""
SL3103	CP022791	393737	395208	family "IS66"	group ""
SL3103	CP022791	627813	629284	family "IS66"	group ""
SL3103	CP022791	1910519	1911990	family "IS66"	group ""
SL3103	CP022791	520101	521778	family "IS1182"	group ""
SL3103	CP022791	1170621	1172298	family "IS1182"	group ""
SL3103	CP022791	2040892	2042569	family "IS1182"	group ""
SL3103	CP022791	1626467	1628034	family "IS1182"	group ""
SL3103	CP022791	521984	522839	family "IS5"	group "IS427"
SL3103	CP022791	1305171	1306026	family "IS5"	group "IS427"
SL3103	CP022791	1387521	1388376	family "IS5"	group "IS427"

SL3103	CP022791	764759	765610	family "IS5"	group "IS427"
SL3103	CP022791	17249	17832	family "IS5"	group "IS427"
SL3103	CP022791	624589	625674	family "IS1595"	group "ISSod11"
SL3103	CP022791	817523	818608	family "IS1595"	group "ISSod11"
SL3103	CP022791	1574629	1575714	family "IS1595"	group "ISSod11"
SL3103	CP022791	942440	948415	family "Tn3"	group ""
SL3103	CP022791	1068535	1074508	family "Tn3"	group ""
SL3103	CP022791	1149637	1151085	family "IS3"	group "IS150"
SL3103	CP022791	2116084	2117532	family "IS3"	group "IS150"
SL3103	CP022791	1388383	1389597	family "IS3"	group "IS51"
SL3103	CP022791	1913293	1914461	family "IS3"	group "IS51"
SL3103	CP022791	1609351	1610565	family "IS3"	group "IS51"
SL3103	CP022791	2080683	2082688	family "IS110"	group "IS1111"
SL3103	CP022791	2086632	2088633	family "IS110"	group "IS1111"
T117	CP022756	101119	102298	family "IS5"	group "IS5"
T117	CP022756	1428959	1430138	family "IS5"	group "IS5"
T117	CP022756	2026720	2027895	family "IS5"	group "IS5"
T117	CP022756	1853477	1854653	family "IS5"	group "IS5"
T117	CP022756	590617	591796	family "IS5"	group "IS5"
T117	CP022756	690296	691475	family "IS5"	group "IS5"
T117	CP022756	884105	885284	family "IS5"	group "IS5"
T117	CP022756	1138835	1140014	family "IS5"	group "IS5"
T117	CP022756	1145735	1146914	family "IS5"	group "IS5"
T117	CP022756	1337884	1339063	family "IS5"	group "IS5"
T117	CP022756	1565145	1566321	family "IS5"	group "IS5"
T117	CP022756	46953	48126	family "IS5"	group "IS5"
T117	CP022756	48384	49552	family "IS5"	group "IS5"
T117	CP022756	936102	936987	family "IS5"	group "-"
T117	CP022756	49552	50437	family "IS5"	group "-"
T117	CP022756	93480	94365	family "IS5"	group "-"
T117	CP022756	1664474	1665359	family "IS5"	group "-"
T117	CP022756	1408344	1409229	family "IS5"	group "-"
T117	CP022756	823520	824396	family "IS5"	group "-"
T117	CP022756	850177	851650	family "IS4"	group "IS50"
T117	CP022756	937685	939158	family "IS4"	group "IS50"
T117	CP022756	358139	359612	family "IS4"	group "IS50"
T117	CP022756	909760	911233	family "IS4"	group "IS50"
T117	CP022756	547925	549311	family "IS110"	group "IS1111"
T117	CP022756	686456	687837	family "IS110"	group "IS1111"
T117	CP022756	615847	617078	family "IS3"	group "IS51"
T117	CP022756	1639558	1640789	family "IS3"	group "IS51"
T117	CP022756	1418454	1419685	family "IS3"	group "IS51"
T117	CP022756	2010653	2012658	family "IS110"	group "IS1111"
T117	CP022756	2014042	2016043	family "IS110"	group "IS1111"
T117	CP022756	1140585	1142147	family "ISNCY"	group "IS1202"
T117	CP022756	1142052	1142727	family "None"	group "None"
SL2330	CP022795	87759	88939	family "IS5"	group "IS5"

SL2330	CP022795	835829	837009	family "IS5"	group "IS5"
SL2330	CP022795	1071668	1072848	family "IS5"	group "IS5"
SL2330	CP022795	521788	522964	family "IS5"	group "IS5"
SL2330	CP022795	582296	583472	family "IS5"	group "IS5"
SL2330	CP022795	1264966	1266142	family "IS5"	group "IS5"
SL2330	CP022795	1302158	1303334	family "IS5"	group "IS5"
SL2330	CP022795	1847394	1848570	family "IS5"	group "IS5"
SL2330	CP022795	1466063	1467236	family "IS5"	group "IS5"
SL2330	CP022795	194955	196333	family "IS110"	group "IS1111"
SL2330	CP022795	540922	542300	family "IS110"	group "IS1111"
SL2330	CP022795	599777	601149	family "IS110"	group "IS1111"
SL2330	CP022795	1483445	1484813	family "IS110"	group "IS1111"
SL2330	CP022795	604814	606045	family "IS3"	group "IS51"
SL2330	CP022795	1573822	1575053	family "IS3"	group "IS51"
SL3755	CP022783	87755	88931	family "IS5"	group "IS5"
SL3755	CP022783	522694	523870	family "IS5"	group "IS5"
SL3755	CP022783	837230	838406	family "IS5"	group "IS5"
SL3755	CP022783	1074969	1076145	family "IS5"	group "IS5"
SL3755	CP022783	1268229	1269402	family "IS5"	group "IS5"
SL3755	CP022783	1305435	1306608	family "IS5"	group "IS5"
SL3755	CP022783	1469333	1470506	family "IS5"	group "IS5"
SL3755	CP022783	194956	196334	family "IS110"	group "IS1111"
SL3755	CP022783	541827	543205	family "IS110"	group "IS1111"
SL3755	CP022783	599515	600887	family "IS110"	group "IS1111"
SL3755	CP022783	1486714	1488082	family "IS110"	group "IS1111"
SL3755	CP022783	207710	208565	family "IS5"	group "IS427"
SL3755	CP022783	668784	669639	family "IS5"	group "IS427"
SL3755	CP022783	204707	205291	family "IS5"	group "IS427"
SL3755	CP022783	604552	605783	family "IS3"	group "IS51"
SL3755	CP022783	1579004	1580235	family "IS3"	group "IS51"
T25	CP023015	87846	88909	family "IS5"	group "IS5"
T25	CP023015	522530	523593	family "IS5"	group "IS5"
T25	CP023015	723401	724464	family "IS5"	group "IS5"
T25	CP023015	1465593	1466656	family "IS5"	group "IS5"
T25	CP023015	686376	687439	family "IS5"	group "IS5"
T25	CP023015	916686	917749	family "IS5"	group "IS5"
T25	CP023015	94295	95358	family "IS5"	group "IS5"
T25	CP023015	196094	197472	family "IS110"	group "IS1111"
T25	CP023015	1446263	1447641	family "IS110"	group "IS1111"
T25	CP023015	1388592	1389964	family "IS110"	group "IS1111"
T25	CP023015	1482971	1484339	family "IS110"	group "IS1111"
T25	CP023015	1383696	1384927	family "IS3"	group "IS51"
T25	CP023015	1573306	1574537	family "IS3"	group "IS51"
T25	CP023015	908658	910219	family "ISNCY"	group "IS1202"
T25	CP023015	910124	910798	family "None"	group "None"
T25	CP023015	1319836	1320690	family "IS5"	group "IS427"
T25	CP023015	205843	206427	family "IS5"	group "IS427"

T78	CP022767	97820	100019	family "IS66"	group ""
T78	CP022767	122211	124410	family "IS66"	group ""
SL3730	CP022785	2179	3611	family "IS4"	group ""
SL3730	CP022785	468358	469790	family "IS4"	group ""
SL3730	CP022785	34784	35964	family "IS5"	group "IS5"
SL3730	CP022785	396295	397475	family "IS5"	group "IS5"
SL3730	CP022785	621176	622356	family "IS5"	group "IS5"
SL3730	CP022785	1472996	1474176	family "IS5"	group "IS5"
SL3730	CP022785	1723943	1725123	family "IS5"	group "IS5"
SL3730	CP022785	51039	52215	family "IS5"	group "IS5"
SL3730	CP022785	599944	601120	family "IS5"	group "IS5"
SL3730	CP022785	604296	605472	family "IS5"	group "IS5"
SL3730	CP022785	792992	794168	family "IS5"	group "IS5"
SL3730	CP022785	1044728	1045904	family "IS5"	group "IS5"
SL3730	CP022785	1051630	1052806	family "IS5"	group "IS5"
SL3730	CP022785	1243781	1244957	family "IS5"	group "IS5"
SL3730	CP022785	344331	345504	family "IS5"	group "IS5"
SL3730	CP022785	1761595	1762775	family "IS5"	group "IS5"
SL3730	CP022785	103961	105137	family "IS5"	group "IS5"
SL3730	CP022785	1336841	1338017	family "IS5"	group "IS5"
SL3730	CP022785	48221	49436	family "IS5"	group "IS5"
SL3730	CP022785	92880	94095	family "IS5"	group "IS5"
SL3730	CP022785	213613	214828	family "IS5"	group "IS5"
SL3730	CP022785	216550	217765	family "IS5"	group "IS5"
SL3730	CP022785	659851	661066	family "IS5"	group "IS5"
SL3730	CP022785	299269	300476	family "IS5"	group "IS5"
SL3730	CP022785	116039	117169	family "IS5"	group "IS903"
SL3730	CP022785	597900	599030	family "IS5"	group "IS903"
SL3730	CP022785	617183	618038	family "IS5"	group "IS427"
SL3730	CP022785	1780520	1781375	family "IS5"	group "IS427"
SL3730	CP022785	300477	301328	family "IS5"	group "IS427"
SL3730	CP022785	102032	102886	family "IS5"	group "IS427"
SL3730	CP022785	845996	846851	family "IS5"	group "IS427"
SL3730	CP022785	212958	213541	family "IS5"	group "IS427"
SL3730	CP022785	1277946	1278831	family "IS5"	group "-"
SL3730	CP022785	762913	763798	family "IS5"	group "-"
SL3730	CP022785	843499	844384	family "IS5"	group "-"
SL3730	CP022785	1316224	1317109	family "IS5"	group "-"
SL3730	CP022785	733001	733879	family "IS5"	group "-"
SL3730	CP022785	1326333	1327564	family "IS3"	group "IS51"
SL3730	CP022785	1547409	1548640	family "IS3"	group "IS51"
SL3730	CP022785	1919634	1921639	family "IS110"	group "IS1111"
SL3730	CP022785	1923023	1925024	family "IS110"	group "IS1111"
SL3730	CP022785	556118	557474	family "IS110"	group "IS1111"
12D	No IS(s) found using OASIS Predictor...				
DTP0602	IS_79ead2f9	269372	270872	family "IS4"	group "IS50"
DTP0602	IS_79ead2f9	2796125	2797625	family "IS4"	group "IS50"

DTP0602	IS_79ead2f9	703482	704982	family "IS4"	group "IS50"
DTP0602	IS_79ead2f9	2425779	2427279	family "IS4"	group "IS50"
DTP0602	IS_79ead2f9	258132	259632	family "IS4"	group "IS50"
DTP0602	IS_79ead2f9	333337	334837	family "IS4"	group "IS50"
DTP0602	IS_79ead2f9	2172755	2174255	family "IS4"	group "IS50"
DTP0602	IS_79ead2f9	2283771	2285271	family "IS4"	group "IS50"
DTP0602	IS_79ead2f9	557098	558269	family "IS630"	group ""
DTP0602	IS_79ead2f9	1070985	1072154	family "IS630"	group ""
DTP0602	IS_79ead2f9	655798	657706	family "IS1634"	group ""
DTP0602	IS_79ead2f9	2050670	2052561	family "IS1634"	group ""
DTP0602	IS_79ead2f9	1029939	1031046	family "IS630"	group ""
DTP0602	IS_79ead2f9	1294073	1295180	family "IS630"	group ""
DTP0602	IS_79ead2f9	2462731	2464706	family "IS1634"	group ""
DTP0602	IS_79ead2f9	2521395	2523358	family "IS1634"	group ""
DTP0602	IS_79ead2f9	1203051	1205014	family "IS1634"	group ""
DTP0602	IS_79ead2f9	16446	17363	family "IS256"	group ""
DTP0602	IS_79ead2f9	891145	892622	family "IS630"	group ""
DTP0602	IS_79ead2f9	1094952	1096430	family "IS630"	group ""
DTP0602	IS_79ead2f9	974083	975561	family "IS630"	group ""
DTP0602	IS_79ead2f9	2549642	2551216	family "IS256"	group ""
FC1138	IS_530769a3	54114	55403	family "IS3"	group "IS3"
FC1138	IS_530769a3	1837779	1839068	family "IS3"	group "IS3"
FC1138	IS_530769a3	525242	527704	family "IS21"	group "-"
FC1138	IS_530769a3	876879	879341	family "IS21"	group "-"
FC1138	IS_530769a3	1219698	1222096	family "IS21"	group "-"
FC1138	IS_530769a3	429144	431603	family "IS21"	group "-"
FC1138	IS_530769a3	529752	531204	family "IS4"	group "IS4"
FC1138	IS_530769a3	643710	644914	family "IS5"	group "IS5"
FC1138	IS_530769a3	861040	862306	family "IS3"	group "IS407"
FC1138	IS_530769a3	541390	541922	family "IS3"	group "IS407"
SL3022	CP023017	1205841	1207045	family "IS3"	group "IS407"
SL3022	CP023017	1284095	1285299	family "IS3"	group "IS407"
SL3022	CP023017	1981301	1982505	family "IS3"	group "IS407"
SL3022	CP023017	1940049	1940923	family "IS5"	group "IS427"
SL3022	CP023017	164182	165037	family "IS5"	group "IS427"
SL2064	IS_c0f1f2ff	1146108	1147312	family "IS3"	group "IS407"
SL2312	CP022797	1784775	1786042	family "IS5"	group "IS5"
T101	CP022757	3474861	3476234	family "None"	group "None"
T82	CP022764	1784777	1786044	family "IS5"	group "IS5"
T95	CP022762	1146125	1147329	family "IS3"	group "IS407"

12J	IS_93519229	467135	467995	family "IS5"	group "IS1031"
ATCC49129	No IS(s) found using OASIS Predictor...				
T51	IS_28b06e31	1146117	1147321	family "IS3"	group "IS407"
A2HRMARDI	CP019912	1068526	1069271	family "None"	group "None"
A2HRMARDI	CP019912	483773	484518	family "None"	group "None"
A2HRMARDI	CP019912	1071778	1072523	family "None"	group "None"
A2HRMARDI	CP019912	1408610	1409355	family "None"	group "None"
A2HRMARDI	CP019912	58707	59451	family "None"	group "None"
A2HRMARDI	CP019912	1069328	1070011	family "None"	group "None"
A2HRMARDI	CP019912	1072580	1073263	family "None"	group "None"
A2HRMARDI	CP019912	483034	483716	family "None"	group "None"
A2HRMARDI	CP019912	57967	58650	family "None"	group "None"
A2HRMARDI	CP019912	1409412	1410095	family "None"	group "None"
A2HRMARDI	CP019912	140540	141874	family "IS3"	group "IS2"
A2HRMARDI	CP019912	496691	498025	family "IS3"	group "IS2"
A2HRMARDI	CP019912	165370	166704	family "IS3"	group "IS2"
A2HRMARDI	CP019912	883180	884514	family "IS3"	group "IS2"
A2HRMARDI	CP019912	984968	986302	family "IS3"	group "IS2"
A2HRMARDI	CP019912	1291135	1292469	family "IS3"	group "IS2"
A2HRMARDI	CP019912	1065020	1066209	family "IS3"	group "IS2"
A2HRMARDI	CP019912	982	2988	family "IS3"	group "IS407"
A2HRMARDI	CP019912	882015	883179	family "IS3"	group "IS407"
A2HRMARDI	CP019912	498984	499913	family "IS3"	group "IS407"
A2HRMARDI	CP019912	450350	451278	family "IS3"	group "IS407"
A2HRMARDI	CP019912	594531	595459	family "IS3"	group "IS407"
A2HRMARDI	CP019912	676350	677278	family "IS3"	group "IS407"
A2HRMARDI	CP019912	1221928	1222856	family "IS3"	group "IS407"
A2HRMARDI	CP019912	593339	594221	family "IS3"	group "IS407"
A2HRMARDI	CP019912	562762	563999	family "IS5"	group "IS1031"
A2HRMARDI	CP019912	1151250	1152354	family "IS5"	group "IS1031"
A2HRMARDI	CP019912	1292470	1293487	family "IS5"	group "IS1031"
A2HRMARDI	CP019912	977524	978642	family "IS630"	group ""
A2HRMARDI	CP019912	952611	953728	family "IS630"	group ""
A2HRMARDI	CP019912	137619	138738	family "IS630"	group ""
A2HRMARDI	CP019912	143883	145002	family "IS630"	group ""
A2HRMARDI	CP019912	246536	247655	family "IS630"	group ""
A2HRMARDI	CP019912	234814	235933	family "IS630"	group ""
A2HRMARDI	CP019912	513940	515059	family "IS630"	group ""
A2HRMARDI	CP019912	1139106	1140221	family "IS630"	group ""
A2HRMARDI	CP019912	250733	251619	family "IS630"	group ""
A2HRMARDI	CP019912	451279	452165	family "IS630"	group ""
A2HRMARDI	CP019912	493315	494189	family "IS630"	group ""
A2HRMARDI	CP019912	498437	498984	family "IS630"	group ""
SL2729	CP022793	2180	3612	family "IS4"	group ""
SL2729	CP022793	468401	469833	family "IS4"	group ""
SL2729	CP022793	34788	35968	family "IS5"	group "IS5"

SL2729	CP022793	396335	397515	family "IS5"	group "IS5"
SL2729	CP022793	621230	622410	family "IS5"	group "IS5"
SL2729	CP022793	1492345	1493525	family "IS5"	group "IS5"
SL2729	CP022793	1743304	1744484	family "IS5"	group "IS5"
SL2729	CP022793	51043	52219	family "IS5"	group "IS5"
SL2729	CP022793	599995	601171	family "IS5"	group "IS5"
SL2729	CP022793	604348	605524	family "IS5"	group "IS5"
SL2729	CP022793	812299	813475	family "IS5"	group "IS5"
SL2729	CP022793	1064058	1065234	family "IS5"	group "IS5"
SL2729	CP022793	1070961	1072137	family "IS5"	group "IS5"
SL2729	CP022793	1263118	1264294	family "IS5"	group "IS5"
SL2729	CP022793	344367	345540	family "IS5"	group "IS5"
SL2729	CP022793	1780958	1782138	family "IS5"	group "IS5"
SL2729	CP022793	103971	105147	family "IS5"	group "IS5"
SL2729	CP022793	1356181	1357357	family "IS5"	group "IS5"
SL2729	CP022793	48225	49440	family "IS5"	group "IS5"
SL2729	CP022793	92889	94104	family "IS5"	group "IS5"
SL2729	CP022793	213610	214825	family "IS5"	group "IS5"
SL2729	CP022793	216547	217762	family "IS5"	group "IS5"
SL2729	CP022793	659906	661121	family "IS5"	group "IS5"
SL2729	CP022793	299299	300506	family "IS5"	group "IS5"
SL2729	CP022793	116050	117180	family "IS5"	group "IS903"
SL2729	CP022793	597951	599081	family "IS5"	group "IS903"
SL2729	CP022793	617237	618092	family "IS5"	group "IS427"
SL2729	CP022793	102041	102896	family "IS5"	group "IS427"
SL2729	CP022793	1799883	1800738	family "IS5"	group "IS427"
SL2729	CP022793	300507	301358	family "IS5"	group "IS427"
SL2729	CP022793	865307	866162	family "IS5"	group "IS427"
SL2729	CP022793	212955	213538	family "IS5"	group "IS427"
SL2729	CP022793	1297286	1298171	family "IS5"	group "-"
SL2729	CP022793	782218	783103	family "IS5"	group "-"
SL2729	CP022793	862809	863694	family "IS5"	group "-"
SL2729	CP022793	1335563	1336448	family "IS5"	group "-"
SL2729	CP022793	752305	753183	family "IS5"	group "-"
SL2729	CP022793	1345673	1346904	family "IS3"	group "IS51"
SL2729	CP022793	1566762	1567993	family "IS3"	group "IS51"
SL2729	CP022793	1939001	1941006	family "IS110"	group "IS1111"
SL2729	CP022793	1942390	1944391	family "IS110"	group "IS1111"
SL2729	CP022793	556165	557521	family "IS110"	group "IS1111"
T110	IS_3f40740b	602980	604214	family "IS3"	group "IS51"
T110	IS_3f40740b	1570711	1571925	family "IS3"	group "IS51"
T110	IS_3f40740b	194779	196134	family "IS110"	group "IS1111"
T110	IS_3f40740b	597967	599322	family "IS110"	group "IS1111"
T110	IS_3f40740b	540356	541712	family "IS110"	group "IS1111"
T110	IS_3f40740b	1480410	1481766	family "IS110"	group "IS1111"
T110	IS_3f40740b	1299331	1300512	family "IS5"	group "IS5"
T110	IS_3f40740b	1463034	1464214	family "IS5"	group "IS5"
T110	IS_3f40740b	1574827	1576007	family "IS5"	group "IS5"
T110	IS_3f40740b	1069294	1070469	family "IS5"	group "IS5"
T110	IS_3f40740b	833697	834877	family "IS5"	group "IS5"

T110	IS_3f40740b	87678	88853	family "IS5"	group "IS5"
T110	IS_3f40740b	1262170	1263345	family "IS5"	group "IS5"
T110	IS_3f40740b	521224	522397	family "IS5"	group "IS5"
SL3175	NZ_CP022789	4628	5861	family "IS3"	group "IS407"
SL3175	NZ_CP022789	1784849	1786072	family "IS3"	group "IS407"
SL3175	NZ_CP022789	623871	625110	family "IS3"	group "IS407"
T98	NZ_CP022760	4628	5861	family "IS3"	group "IS407"
T98	NZ_CP022760	1784847	1786070	family "IS3"	group "IS407"
T98	NZ_CP022760	623881	625120	family "IS3"	group "IS407"
T11	NZ_CP022777	1145473	1146677	family "IS3"	group "IS407"
T12	CP022775	1784604	1785871	family "IS5"	group "IS5"
PSI07	IS_6244ae36.2	301593	302445	family "IS5"	group "IS427"
KACC1722	IS_d132e9d0	1145222	1146426	family "IS3"	group "IS407"

Table S5. Distribution of IS family in *Ralstonia spp.* genomes

Strain	Family																
	**Total**	**IS110**	**IS1182**	**IS1595**	**IS21**	**IS256**	**IS3**	**IS30**	**IS4**	**IS481**	**IS5**	**IS630**	**IS66**	**IS701**	**ISL3**	**ISNCY**	**Tn3**
12J	47						4				5	5	9		8	1	15
CFBP2957	30					8	11			2	4				2		3
IBSBF1503	53			4	20		13				6	1			2	1	6
K60	165	11	22	7	19		12		28	3	28	22	1	7	4		1
P082	53	1		4	16		17			1	5	1			2		6
RS488	123	1				1	14			1	97				3	2	4
RS489	141	2	1	1	2	1	10			1	115				2	3	3
UW163	55			6	20		14			1	5	1			2		6
UW551	146	2	1		2	1	9			1	121				3	3	3
UY031	119	1				1	11			1	97				3	2	3
CMR15	97	1		3	2		16		11		39	1	6	6	3	2	7
CQPS- 1	95	1	2	1	2		16		11		39	1	6	6	3	1	6
CRMRs218	119			2	6	2	53		8		24	10			6	4	4
EP1	85	5		1	2		9		12	2	33	1	7	2	1	2	8
FJAT- 91	86	5		2	1		11		12	2	33	1	7	2	1	2	7
FJAT-1458	123	8	2	3			17		14	1	60	1			7	5	5
FQY_4	139	11		1			10	5	20		48	1	35		1	4	3
GMI1000	100			2	8	1	33	1	7		24	9		7	4	4	
HA4-1	120	9		3	10	1	12	1	12	3	52		3		1	5	8
KACC10709	204	8	8	5			22		16		107		32		1	1	4
KACC10722	26			1	1		6				3	3		1	4	4	3
OE1-1	77	4		1	1		12		15		33	1		3	1	2	4
YC40-M	155	19	2	1	1	1	14	5	23		65	3	15		1	5	
RS 476	102			1	6	2	36	1	7		24	10	1	7	4	3	
Rs-09-161	49			2			9	6	2	1	13	4		2	2	3	5
Rs-10-244	32	1		1			12			1	8	1		1	2	1	4
RSCM	483	22	26	2	8		95		27		162	20	74	33	1	3	10
SEPPX05	259	3		3	5		60		18	4	61		9	12	11	1	12
SL2330	80	6		3			11		9	1	33	1		2	4	5	5
SL2729	167	10	5	2			23		22		93				2	7	3

SL3103	214	8	8	5			15	15		103		49		1	3	7
SL3300	140	6	3	1			21	17	2	80				4	3	3
SL3730	108	6	2	1			15	18		59					4	3
SL3755	77	6	2				14	9		27	1		2	4	5	7
SL3822	102	8	2	5			19	3	2	48	2	4		4	3	2
SL3882	108	7	2	3			17	14	1	48	1			4	4	7
T110	130	16		3			20	10		61	1		1	4	9	5
T117	87	6	2	2			11	20	2	35	1			1	3	4
T25	75	7		3			12	9		31	1			3	6	3
T42	101	5	2	1			14	18		52				2	4	3
T60	110	7	2	3			19	15	1	50	1			4	4	4
T78	62	2	1	2			7	15		17	1	6		5	2	4
T82	26				1		5		1	9				2	4	4
T95	17			1	1		2			1	2		1	3	4	2
T98	42		1		1		16			8	1			5	7	3
PSI07	67			2	2		38		2	12	1			3	4	3
SL2064	21			1	1		4			2	2		1	3	4	3
SL2312	41				2		8		1	13	4			4	5	4
T101	28						9		1	9	2			2	2	3
T11	21				1		6			2	3		1	3	4	1
T12	29				1		6		1	9	2			2	4	4
SL3022	35	1		1	1		10		2	7	2	1		1	6	3
SL3175	41		1		1		15			8	1			5	7	3
T51	19				1		3			2	3		1	3	4	2
A2- HR	172					62	30		1	32	42		1	1	2	1

Table S6. Impact of Insertion sequences on virulence factors of *Ralstonia solanacearum* Species Complex

**Strain**	**Family**	**Gene**	**Classes**	**IS Coordinates**	
GMI1000	IS21	putative calcium binding hemolysin protein	Overlapping	275259	276302
GMI1000	IS256	probable phenazine biosynthesis phzc/phzf protein	Near	2532717	2533562
GMI1000	IS4 ssgr IS4	yopp/avrrvx-related protein	Truncated	3500148	3498814
GMI1000	IS630	calcium binding hemolysin protein	Truncated	117992	119083
Po82	IS1595 ssgr ISPna2	hemolysin activator translocator	Near	300425	301885
Po82	IS3 ssgr IS51	hemagglutinin-related protein	Near	1356718	1357041
Po82	IS3 ssgr IS2	type III secretion system protein	Near	2816389	2816781
UY031	IS5 ssgr IS5	Putative type III effector protein	Truncated	50636	51622
UY031	IS5 ssgr IS5	Endoglucanase precursor	Near	344825	343839
UY031	IS5 ssgr IS5	RHS Repeat protein	Near	2428298	2427312
UY031	IS5 ssgr IS5	Type III effector protein	Near	3406083	3405097
UY031	IS3 ssgr IS51	Hemagglutinin-related protein	Overlapping	2239387	2239710
RS488	IS5 ssgr IS5	Type III effector protein	Truncated	50636	51622
RS488	IS5 ssgr IS5	filamentous hemagglutinin	Truncated	308712	309698
RS488	IS5 ssgr IS5	filamentous hemagglutinin	Truncated	320244	32123
RS488	IS5 ssgr IS5	Type III effector protein (Skwp5)	Truncated	1162855	1163841
RS488	IS5 ssgr IS5	proline dehydrogenase	Truncated	2793853	2794839
RS488	IS5 ssgr IS5	Type III effector protein RipI	Near	3406083	3405097
RS488	IS3 ssgr IS2	type III effector protein RipJ (Putative acetyltransferase)	Truncated	2816324	2817534
RS489	IS5 ssgr IS5	Type III effector protein RipI	Truncated	50662	51648
RS489	IS5 ssgr IS5	filamentous hemagglutinin	Truncated	308788	309774
RS489	IS5 ssgr IS5	Type III effector protein (Skwp5)	Truncated	1162885	1163871
RS489	IS5 ssgr IS5	proline dehydrogenase	Truncated	2793773	2794759
RS489	IS5 ssgr IS5	Type III effector protein RipI	Truncated	3405902	3404916
OE1	IS5	hemolysin-type protein	Near	2956686	2955862
OE1	IS5 ssgr IS427	type III effector protein AvrRpm1	Near	286034	285627
OE1	IS5 ssgr IS427	hemagglutinin	Truncated	94702	946613
OE1	IS5 ssgr IS427	type III secretion system YopJ family effector PopP2	Near	3579893	3580858
FJAT-1458	IS5 ssgr IS5	type III effector protein RipJ (Putative acetyltransferase)	Truncated	1433245	1432259
CQPS-1	IS21	type III effector protein RipP1	Truncated	555457	556488
CQPS-1	IS3 ssgr IS2	hemolysin	Truncated	2287536	2286700
CQPS-1	IS5 ssgr IS5	subtilase	Truncated	43063	429665

CQPS-1	IS5 ssgr IS5	YOPP/AvrRxv family protein	Truncated	480234	479269
FJAT91	IS110	Type III effector protein (Skwp 4)	Truncated	1807441	1806236
FJAT91	IS1595 ssgr IS1016	hemagglutinin	Truncated	1056134	1056913
RSCM	IS701	non-ribosomal peptide synthetase	Truncated	1758324	1759655
RSCM	IS701	peptide ABC transporter permease	Truncated	3318493	3317132
T110	IS5	hemagglutinin	Near	-	-
T110	IS5	type III effector protein	Truncated	-	-
T110	IS5	type III effector protein	Truncated	-	-
SEPPX05	IS5	type III effector protein CDS	Near	-	-
T117	IS5	Type III effector protein avrA	Overlapping	-	-
SL3175	IS3 ssgr IS407	Type III effector protein	Near	220288	221223
SL3175	IS3 ssgr IS407	Type III effector	Truncated	2696383	2696817
SL3175	ISL3	Metal-dependent hydrolase	Near	2742644	2743358
T25	IS5	type III effector protein CDS	Truncated	-	-
T25	IS5	type III effector protein CDS	Truncated	-	-
T98	IS3 ssgr IS407	Amidohydrolase	Near	550091	551281
T98	IS3 ssgr IS407	Tyrosinase	Near	1473455	1474687
T98	IS3 ssgr IS407	Type III effector	Near	2696366	2696800
SL3022	IS481	Serine hydrolase	Near	797539	798108
SL3022	ISNCY ssgr IS1202	Alpha/beta hydrolase	Near	2015146	2016171
SL2312	IS3 ssgr IS407	Avirulence d protein	Near	2632942	2633811
A2HRMARDI	IS256	Pilus assembly protein PilY	Truncated	1022393	1024894
A2HRMARDI	IS256	Xanthomonas outer protein AQ	Near	1089209	1089194
A2HRMARDI	IS256	Type II secretion system protein GspF	Near	1455081	1456292
A2HRMARDI	IS256	Phenazine biosynthesis protein PhzC/PhzF	Near	1700061	1700918
A2HRMARDI	IS256	Hemagglutinin	Truncated	3153387	3156095
A2HRMARDI	IS5 ssgr IS5	Type III effector protein	Near	2260943	2263089
A2HRMARDI	IS630	Carotenoid oxygenase	Near	1508281	1509843
A2HRMARDI	IS630	Type III effector protein	Near	3196072	3198873
SL3755	IS5	type III effector protein CDS	Truncated	-	-
SL3755	IS5	type III effector protein CDS	Truncated	-	-
